# Supplementary material for: Connectome-constrained networks predict neural activity across the fly visual system
Source: Nature. 2024 Sep 11;634(8036):1132–40. doi: 10.1038/s41586-024-07939-3 (PMC11525180; doi:10.1038/s41586-024-07939-3)
Supplement: Supplementary file 1 — Supplementary Notes 1–5 (describing additional methods and analysis) and Figs. 1–9 (showing additional analysis, including connectome and model parameter statistics, and T4 and T5 motion response mechanisms). [file 41586_2024_7939_MOESM1_ESM.pdf]

nature portfolio

https://doi.org/10.1038/s41586-024-07939-3

Supplementary information

Connectome-constrained networks predict neural activity across the fly visual system

In the format provided by the authors and unedited

Supplementary Information

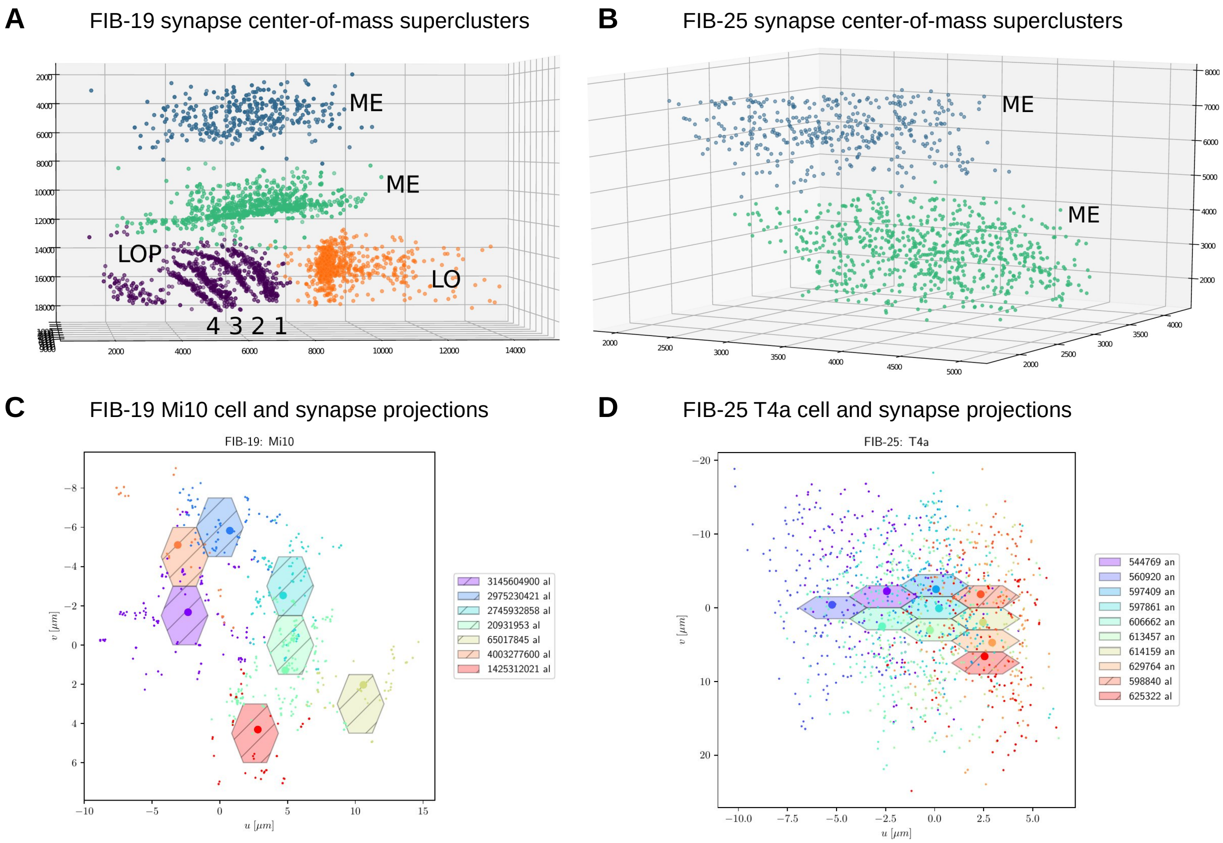

Supplementary Figure 1: **(A)** FIB-19 synapse center-of-mass superclusters. The clusters form two strata in the medulla (ME), and in the lobula (LO) and lobula plate layers (LOP, 1-4) additionally. Each dot corresponds to the center-of-mass of all synapses belonging to the super-cluster. Typically, each diverging arborization of a cell becomes a distinct location, which helps our probabilistic model to project 3D positions of synapses into retinotopic 2D planes, despite the lobula having a different spatial orientation (perpendicular) than the medulla and lobula (Fig. 1a and c). **(B)** FIB-25 synapse center-of-mass superclusters. The clusters form two strata (ME) in the medulla. **(C)** Mi10 cell type in FIB-19 with no pre-annotated lattice positions. The seven cell specimen (hexagons) are recovered by our probabilistic algorithm. Individual synapses and synapse center-of-mass projections are superimposed. **(D)** T4a cell type in FIB-25 with eight pre-annotated (an) and two recovered (al) lattice positions. The projected synapse positions show directional displacement consistent with the direction selectivity of T4a cells (Fig. 3a).

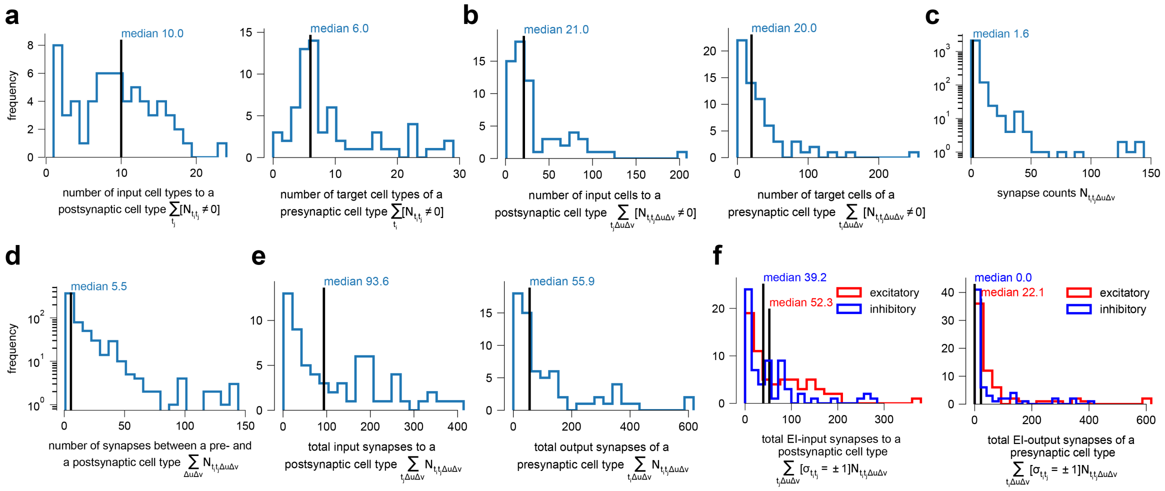

Supplementary Figure 2: **Statistics of derived connectome.** **(a)** (left) Half of the 65 cell types receive input from more than ten other cell types, while the other half receives input from less than ten. (right) Half of the 65 cell types project onto more then six other cell types, while the other half projects onto less than six. **(b)** (left) Half of the 65 cell types receive input from 21 up to 200 cells, while the other half receives input from less than 21 cells. (right) Half of the 65 cell types project output onto 20 up to 200 cells, while the other half projects output onto less than 20 cells. **(c)** Half of the connections are characterized by less than 1.6 synapses while the other half are characterized by 1.6 up to hundreds of synapses. **(d)** A pair of presynaptic and postsynaptic cell type is connected by 5.5 synapses in half of the cases and by more than 5.5 up to hundreds in the other half of the cases. **(e)** (left) Half of the 65 cell types receive input from less than 93.6 synapses and the other half between 93.6 to 400 synapses. (right) Half of the 65 cell types project less than 55.9 synapses and the other half projects between 55.9 to 600 synapses. **(f)** Separating (e) into excitatory and inihibitory synapses, (left) we see that half of the 65 cell types receive excitatory inputs from less than 52.3 synapses and the other half from 52.3 to hundreds. Half of the 65 cell types receive inhibitory inputs from less than 39.2 synapses and the other half from 39.2 to hundreds. (right) Half of the 65 cell types project less than 22.1 excitatory synapses and the other half from 22.1 to hundreds. At least half of the 65 cell types project no inhibitory synapses and the rest project between zero to hundreds.

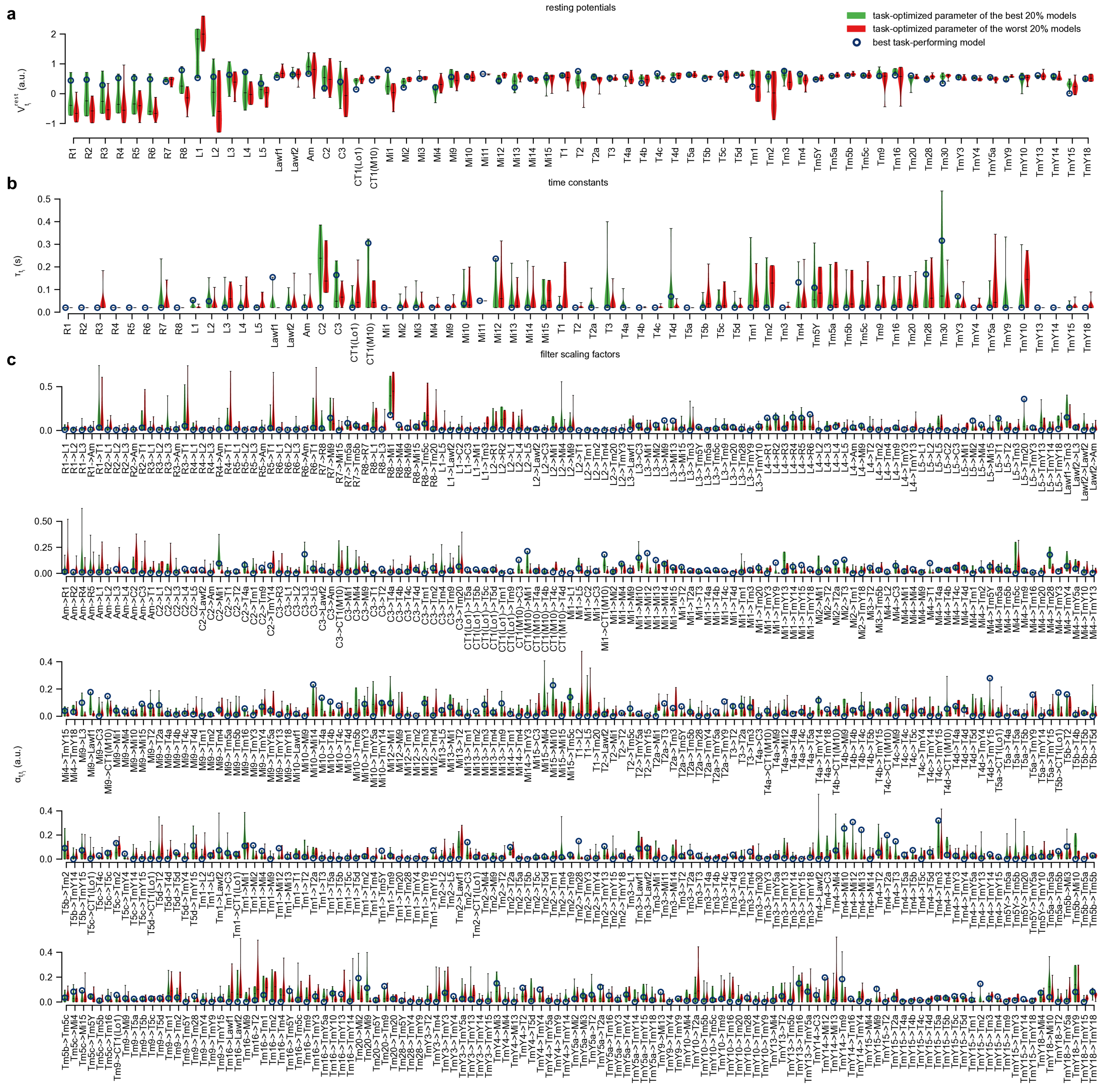

Supplementary Figure 3: **Statistics of learned parameters of best 20% models vs. worst 20% models.** **(a)** Task-optimized resting potentials. **(b)** Task-optimized time constants. **(c)** Task-optimized filter scaling factors.

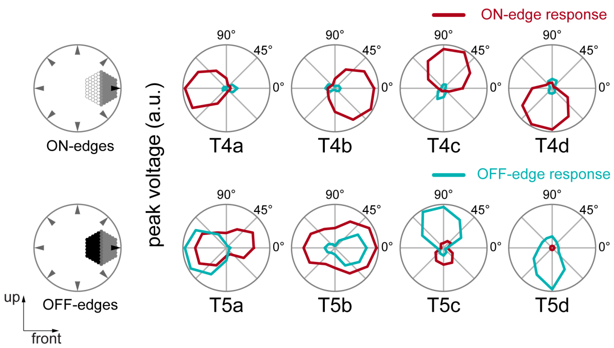

Supplementary Figure 4: **Motion tuning predictions for T4 and T5 subtypes to preferred and null contrast edges in the task-optimal model.**

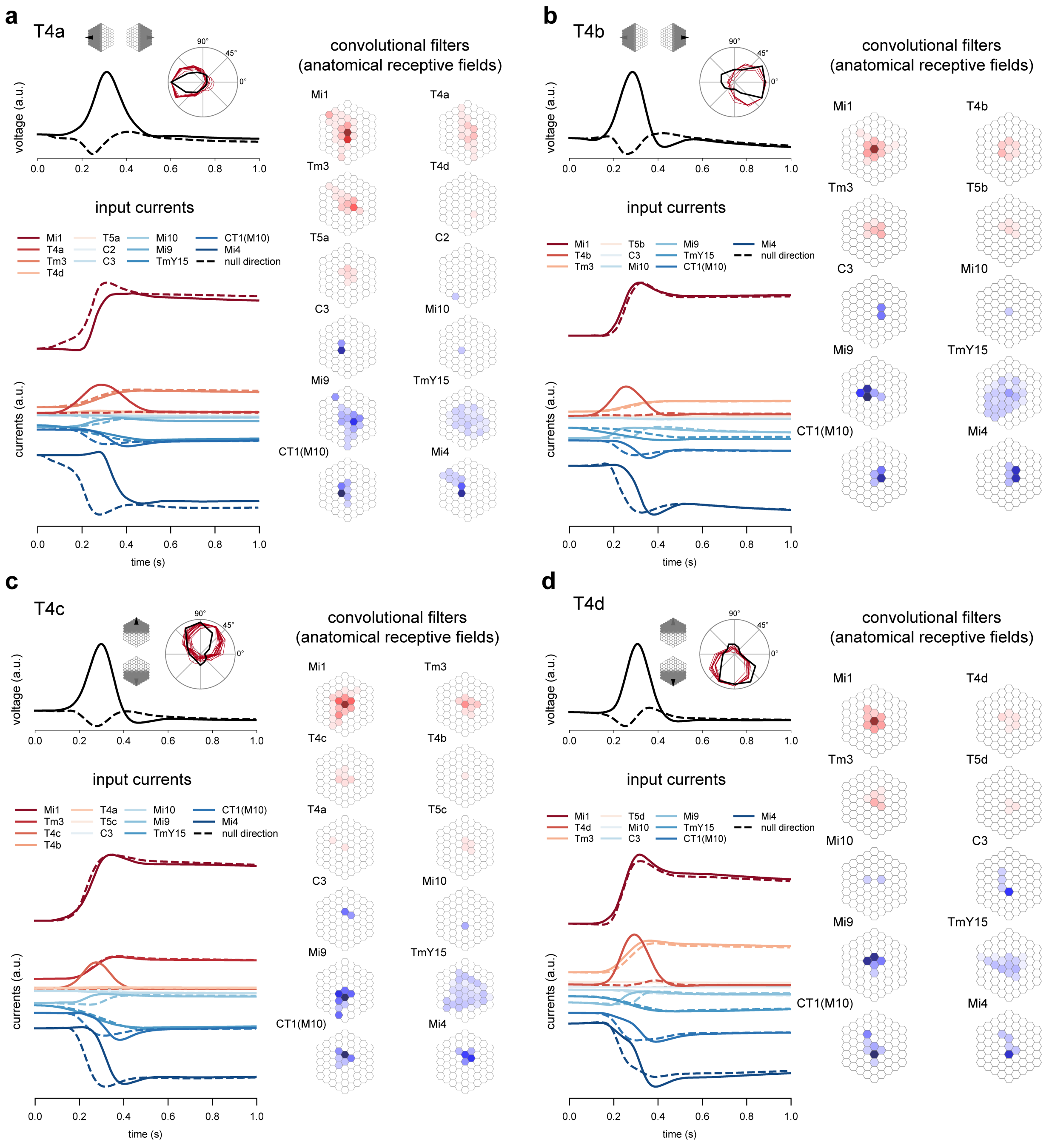

Supplementary Figure 5: **T4 motion detection mechanisms.**

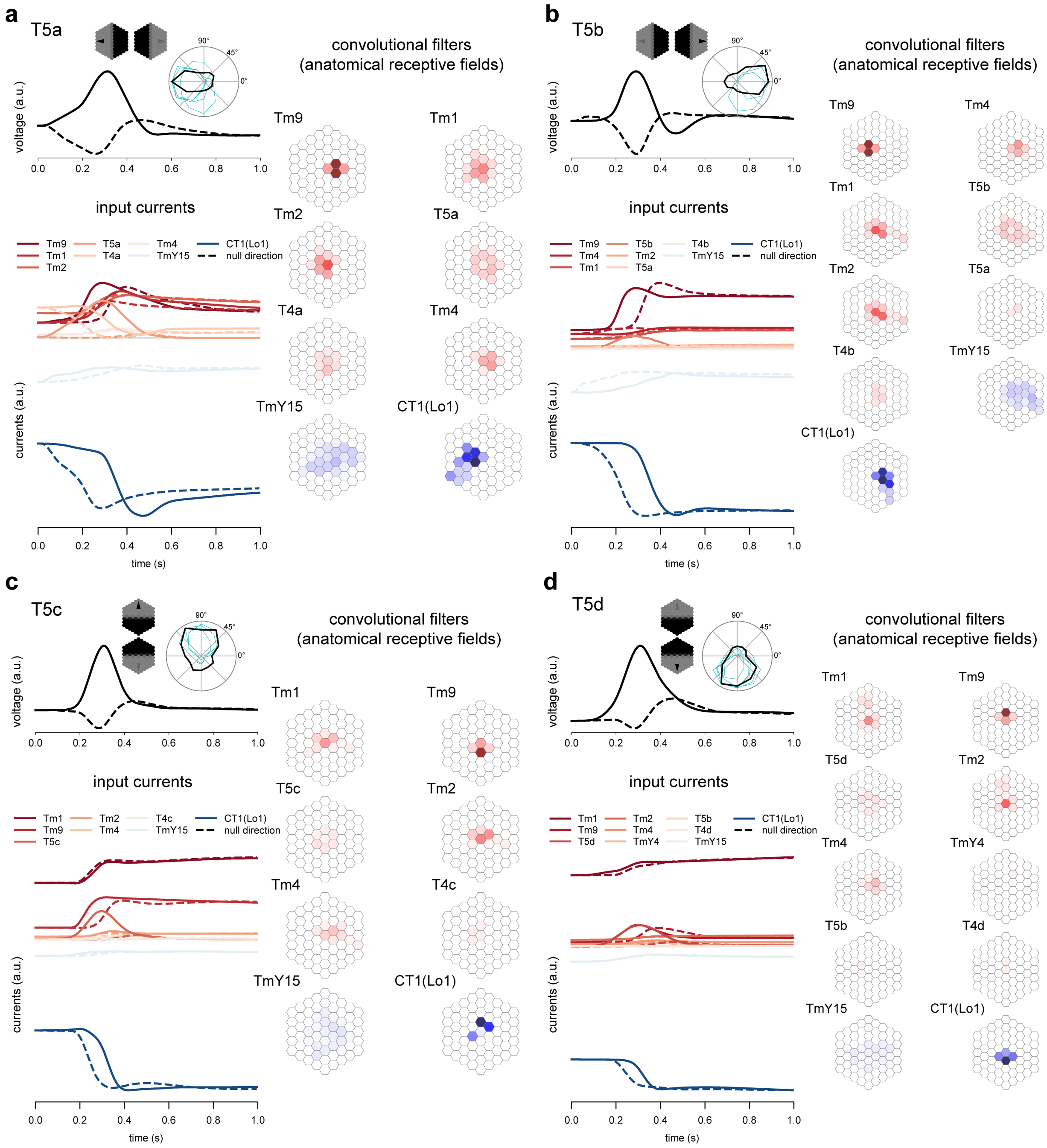

Supplementary Figure 6: **T5 motion detection mechanisms.**

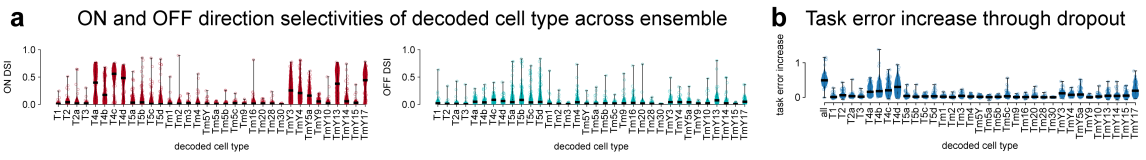

Supplementary Figure 7: **Contribution of all decoded cell types to the motion detection task.** **(a)** Direction selectivity indexes for each decoded cell type and each model from the ensemble of 50 models. (left) Direction selectivity indices from responses to moving ON-edges. (right) Direction selectivity indices from responses to moving OFF-edges. **(b)** Task error increase if all respective decoded cell responses are replaced by their cell type's spatio-temporal averages to the naturalistic input at the decoding stage. This measures the cell type's contribution to the motion detection task. As a baseline, the left-most column shows the per model task error increase if all decoded cell responses are replaced by their cell type's spatio-temporal averages, i.e. no motion can be detected. The decoder attends primarily to the ON-motion selective T4 cell types.

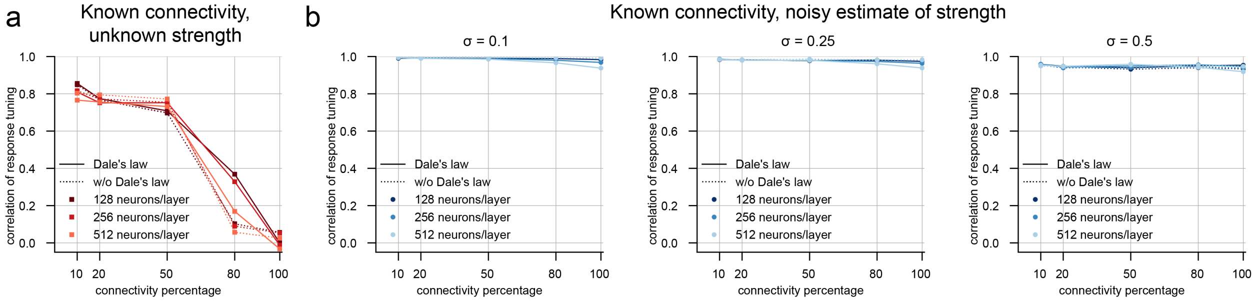

Supplementary Figure 8: **Investigating the role of sparse connectivity with synthetic networks for MNIST handwritten digit recognition.** **(a)** Median hidden-layer response correlation as a function of synthetic network connectivity percentage for task and connectome constrained models that had access to only connectivity information but not connection strength. **(b)** Median hidden-layer response correlation as a function of synthetic network connectivity percentage for task and connectome constrained models with access to noisy estimates of connection strength (multiplicative noise levels of $\sigma = 0.1, \sigma = 0.25$, and $\sigma = 0.5$, respectively). Connectome constrained models were task optimized with a soft (L2) constraint with the noisy connectome measurements.

Supplementary Note 1. Probabilistic model for automatic construction of connectome

The EM-datasets primarily contain the lamina projections, medulla (FIB-25), lobula and lobula plate (FIB-19) cells, and the important cell types of the primary motion detection circuit (T4, T5). In total, they contain 1801 neurons (702 from FIB-25 and 1099 from FIB-19), with hand-annotated positions available for 830 of these neurons (SI Figure 1). To accurately localize the remaining neurons and synapses and to derive cell-type connectivity (Fig. 1b), we build a probabilistic expectation maximization algorithm that takes synaptic connection statistics, projected synapse center-of-mass clusters and existing column annotations into account. We verified the quality of our reconstruction, concluding that even in the absence of 90% of the hand-annotations available to us, we could accurately position the majority of the neurons in our circuit reconstruction (SI Table 1, SI Figure 9). In the absence of ground-truth annotations, we verified the quality of our reconstruction by the *recovery* and *consistency* rates (Table 1). The *recovery* rate is defined as the ratio of reference positions successfully recovered by our algorithm after removing a random proportion of reference positions from the data. For each 10% of the reference positions removed, on average, only 2.5% are not correctly recovered. The *consistency* rate is defined as the fraction of neurons obtaining the same position between evaluations of the algorithm starting with a different fraction of reference positions. For each 10% of the reference positions removed, on average, an additional 3.9% of neurons are not consistently estimated. We found that even just 10% (83 positions) of the available ground truth was sufficient to robustly position the majority of the neurons (64.3%, 534 positions) into the correct columns, and annotate 48% (865 neurons) perfectly consistent.

Probabilistic expectation maximization for unassigned neurons. Each neuron is either annotated in the dataset (in $\mathcal{K}$), assigned to a position (in $\mathcal{A}$) by our algorithm, or still under evaluation (in set $\mathcal{U}$). Iteratively, the EM-expectation step updates the normal distribution ($\mu_{s,t,(y,x)}$, $\sigma_{s,t,(y,x)}$) of expected synapse counts between neurons, while the EM-maximization step updates the positions (y, x) of all neurons not yet assigned to a column (set $\mathcal{U}$).

Synapse center-of-mass as a neuron column position proxy. For offset assignment, we take the center-of-mass of all synapses belonging (pre- or postsynaptic) to a neuron into account. These are generally a more useful hint than the physical location of the cell body, as the cell bodies are mostly positioned on the side of the neuropiles, and not near the column to which the neuron belongs to, but most cells have a majority of synapses in close proximity to their own column. Since many neurons span more than one layer in a neuropile, or even multiple neuropiles, we first group all synapses per neuron into clusters, and then assign the center-of-mass of these clusters to one of up to $N = 5$ super-clusters (approximately matching the medulla, lobula and lobula plate). Clustering is done via k-means with the ideal number of clusters determined by silhouette scores. The super-clusters allow to project 3D synapse coordinates onto a retinotopic 2D hexagonal lattice with a simple projection and affine transformation.

Hybrid cost-model for neuron-position likelihood estimates. Prior knowledge about the normal distribution ($\mu_{s,t,(y,x)}$, $\sigma_{s,t,(y,x)}$) of expected synapse counts between neurons from existing annotations is required to express the probability of any cell specimen c to be located at position (y, x). This metric correlates a neuron to all pre- and postsynaptic neurons it is connected to, of which some already have a fixed, known position. Thereby, the neighbouring neurons with known position (in $\mathcal{K}$ and $\mathcal{A}$) contribute to stabilize the probabilities of unassigned neurons (in set $\mathcal{U}$). For columnar, spatially repeated neurons, we can also

assume that only one neuron per position is present. This prior rapidly discounts the number of possible positions an unassigned neuron can have, each time another neuron becomes assigned (moves from $\mathcal{U}$ to $\mathcal{A}$).

Fusion of separately evaluated datasets. Since FIB-19 and FIB-25 are evaluated separately, we have to combine the estimated parameters of both models to a single, coherent model. The datasets overlap partially, in terms of the neurpoiles and cell types covered, and our method therefore fuses the model by always taking the larger estimated parameter.

$$\mu_{s,t,(y,x)} = \max(\mu^{\text{FIB-19}}_{s,t,(y,x)},\ \mu^{\text{FIB-25}}_{s,t,(y,x)}) \tag{7}$$

This method of model fusion only underestimates the number of synapses between two neurons if they have connections in two different neuropiles, and if each neuropile is exclusively covered by only one dataset.

Pruning spurious synapses. Some automatic annotations, which are not proof read in the FlyEM DVID data, contain a large number of autapses per neuron on most neuron types, arising from wrongly detected synapses in the cell bodies themselves. Additionally, there are many statistically insignificant single synapses left from the assignment algorithm. We imposed the following additional filter on our estimated model parameters, to remove both autapses and spurious connections with less than one synapse on average.

$$\mu_{s,t,(y,x)} = \begin{cases} \emptyset & \text{if } s = t \wedge (y,x) = (0,0) \\ \emptyset & \text{if } \mu_{s,t,(y,x)} < 1 \\ \mu_{s,t,(y,x)} & \text{otherwise} \end{cases} \tag{8}$$

Finally, neuron types without connections and synapses with either missing target or source can be removed. The resulting mean synapse counts $\mu_{s,t,(y,x)}$ form the convolutional filters for our simulation.

Reference positions	1.0	0.9	0.8	0.7	0.6	0.5	0.4	0.3	0.2	0.1	average
Recovery rate	**0.870**	0.849	0.820	0.807	0.790	0.754	0.730	0.707	0.669	0.643	
Recovery rate delta		0.020	0.029	0.013	0.017	0.036	0.024	0.023	0.039	0.025	**0.025**
Consistency rate		0.790	0.728	0.701	0.682	0.638	0.609	0.552	0.546	0.480	
Consistency rate delta			0.062	0.027	0.019	0.044	0.029	0.057	0.006	0.066	**0.039**

Table 1: Recovery and consistency of columnar cell position estimation.

Pos. removed [%]	0	10	20	30	40	50	60	70	80	90	100
Reference positions	830	747	664	581	498	415	332	249	166	83	0
Combined	1801	1459	1305	1346	1248	1152	1229	1081	972	944	0
FIB-25	702	592	573	566	538	509	491	464	445	399	0
FIB-19	1099	867	732	780	710	643	738	617	527	545	0
Combined (annotated)	721	708	694	684	658	643	626	576	566	499	0
FIB-25 (annotated)	393	387	379	375	360	353	339	307	304	272	0
FIB-19 (annotated)	328	321	315	309	298	290	287	269	262	227	0

Table 2: Results of the probabilistic model construction.

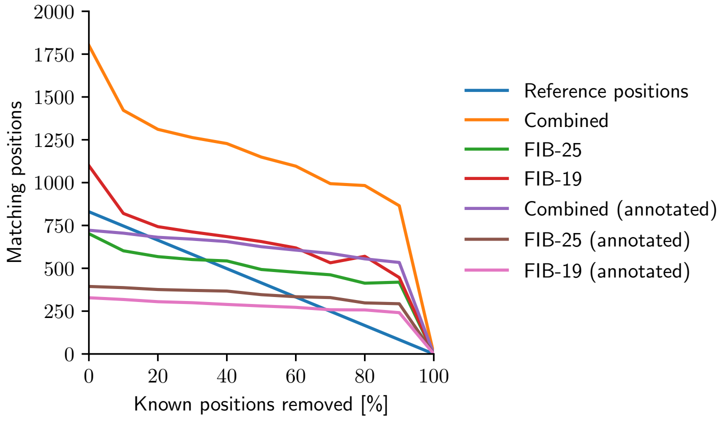

Supplementary Figure 9: Results of the probabilistic model construction.

Supplementary Note 2. Manually constructed connectome components

Lamina and ommatidia model Since neither FIB-19 nor FIB-25 contain the connections of the ommatidia or first neuropile, the lamina [89, 90], we reused and refined the existing hand-crafted model from our previous work [91], which is based on data from Rivera-Alba *et al.* [92] and Tuthill *et al.* [93, 94].

Non-columnar single CT1 cell model While we did in general not model any neurons with large tangential branches, such as Mt, Mt, Pm, Dm, which span many columns and are therefore insufficiently segmented in FIB-19 and FIB-25, we did model the single CT1 cell present in the lobula CT1(Lo1) and medulla CT1(M10) [95]. Because a multi-compartment model with bidirectional electrical synapses resulted often in oscillatory dynamics in earlier modeling attempts and because CT1 terminals were found to act as functionally independent units [96] we modelled CT1 as two anatomically separate cell types CT1(Lo1) and CT1(M10).

Non-columnar periodic cells Because lamina-wide-field neurons, Lawf1 and Lawf2, do not occur in each individual column but more sparsely, we modeled them with an inferred spatial stride to occur more sparsely resulting in 123 cell of each type in our model (there are approx. 140 Lawf2 neurons per optic lobe, and each of approx. 700 columns is innervated by approx. 5 Lawf2 cells [94]).

Hexagonal lattice rendering of connectome For compilation into the hexagonal grid, the convex hull of the filters is filled with ones to remove spatial discontinuities. Although these are considered mostly false positives from the connectome reconstruction [97], this allowed for weak autapses in our hexagonal model that did not affect the tuning predictions.

Additional proofreading We manually proofread filters on the hexagonal lattice and compared them to the reported filters in the literature to ensure overall correspondence. We found that the reconstruction did not fully capture the asymmetry reported in [90] of the T5 anatomical receptive field of Tm9, which we then substituted by a Gaussian at the reported offset column scaled by the reported number of input synapses. For few T4 and T5 inputs the number of input synapses reported in the literature[90] slightly deviated from our reconstruction. To get a better initialization of our filter scale we scaled them to closely match the number of input synapses reported[90].

Supplementary Note 3. Model predictions agree with experimental measurements of neural activity across 26 studies

We compared simulated model responses for each cell type to experimentally reported responses. We found that the 10 models which achieved the best task performance predict neural responses in consensus with experimental measurements across 26 studies[88, 96, 98, 99, 100, 101, 102, 103, 104, 105, 106, 107, 108, 109, 110, 111, 112, 113, 114, 115, 116, 117, 118, 119, 120, 121].

Supplementary Note 4. Models predict motion tuning for TmY3

Amongst models with the best task performance, TmY3, TmY4, and TmY18 are often ON-motion selective (Fig. 2c). As these neurons have yet to be experimentally characterized, we analyzed these prediction in our models. Since TmY3 neurons do not receive inputs from other known motion selective neurons, we were intrigued by the possibility that they might directly compute a motion signal and possibly constitute a parallel pathway to the well-known T4 and T5 neurons. In contrast, TmY4 and TmY18 cells receive inputs from T4 cells, potentially inheriting their motion tuning.

In the model ensemble, we found four distinct clusters for TmY3 (Fig. 4b). In the task-optimal cluster (circular marker) TmY3 responds to ON-edges from front to back or downwards (Fig. 4c). In contrast, in the second cluster (triangular marker), TmY3 is not direction selective. In the third cluster (square marker) TmY3 is direction selective to ON-edges moving from the back to the front. In the fourth cluster (star marker), TmY3 is, again, not direction selective. Together, the ensemble suggests ON-motion sensitivity for TmY3, but different clusters disagree in their predictions for direction and contrast selectivity.

In our connectome data, the strongest input elements of TmY3 by number of synapses are L4, L5, Tm2, Tm3, Mi1, Mi9, and Mi4 (Fig. 4d and e). While none of these input neurons are motion-selective, the asymmetries in their connectivity to TmY3 might allow it to detect motion. We asked if we could better constrain our predictions by asking which clusters also predicted the correct preferred contrast for these input neurons. We found that the first model cluster (Fig. 4b, circular marker), in which TmY3 is tuned to front-back or downwards motion, most accurately captures the known contrast selectivity of all TmY3 input cells (Fig. 4f). In contrast, all three other clusters fail to consistently capture the OFF-selectivity of Mi9. Thus our model proposes TmY3 as a novel candidate motion detector independent of the well-known T4 and T5 motion pathways with the putative mechanism for the task-optimal cluster shown in Extended Data Fig. 5. Examining the mechanism of direction selectivity, we find null direction suppression but no preferred direction enhancement, consistent with previous experimental findings for T4 [88] and T5 [115].

Supplementary Note 5. Investigating the role of sparse connectivity with synthetic networks for MNIST digit recognition

Training feedforward synthetic networks The weight matrix for each layer in Dale's-law-based synthetic networks (DLTrues) is decomposed into three components: binary adjacency matrix, non-negative weight magnitudes, and a sign vector.

$$\begin{aligned} W_{\texttt{DLTrue}} &= C_{\texttt{DLTrue}} \odot W_{\texttt{DLTrue}} \odot [\mathbf{1} \otimes \mathbf{s}_{\texttt{DLTrue}}], \\ \text{where } C_{\texttt{DLTrue}} &= \text{binary adjacency matrix}, \\ W_{\texttt{DLTrue}} &= \text{non-negative weight magnitudes of true network}, \\ \mathbf{s}_{\texttt{DLTrue}} &= \text{sign vector} \end{aligned}$$

By means of projected gradient descent, $W_{\texttt{DLTrue}}$ is enforced to be non-negative and is initialized from the absolute value of the He initialization distribution [122]. Although sign vector $\mathbf{s}_{\texttt{DLTrue}}$ is randomly initialized with equal probability to be either -1 or +1 to represent inhibitory and excitatory synapses respectively, its elements are allowed to assume values in $\mathbb{R}$ over the course of training.

Inducing sparsity Binary adjacency matrix $C_{\texttt{DLTrue}}$ is initialized to be a unit matrix and is later updated according to the desired true network connectivity level. Following the LTH algorithm, a portion of the lowest-magnitude weights, designated to be pruned, were identified from $W_{\texttt{DLTrue}} \odot [\mathbf{1} \otimes \mathbf{s}_{\texttt{DLTrue}}]$. For pruning synapses, the corresponding entries in the adjacency matrix $C_{\texttt{DLTrue}}$ were then set to zero. After each pruning iteration, weight magnitudes were reset back to their original initialization, followed by a final training run post-pruning.

Training with/without sign constraints In addition to a Dale's-law-based sign constraint, we also experimented with networks trained without any sign constraint. No restrictions were imposed on the nature of outgoing synapses i.e., a neuron can have both excitatory and inhibitory outgoing synapses. For true network variants trained without a sign constraint, $\mathbf{1} \otimes \mathbf{s}_{\texttt{DLTrue}}$ was simply replaced by a sign matrix $S_{\texttt{nonDLTrue}}$ initialized in a similar fashion; that is, all entries were initialized to be in $\{-1, +1\}$ with equal probability. We will refer to true networks trained without a sign constraint as `nonDLTrues`.

$$\begin{aligned} W_{\texttt{nonDLTrue}} &= C_{\texttt{nonDLTrue}} \odot W_{\texttt{nonDLTrue}} \odot S_{\texttt{nonDLTrue}}, \\ \text{where } C_{\texttt{nonDLTrue}} &= \text{binary adjacency matrix}, \\ W_{\texttt{nonDLTrue}} &= \text{non-negative weight magnitudes of true network}, \\ S_{\texttt{nonDLTrue}} &= \text{sign matrix} \end{aligned}$$

Training simulated networks As elements in a true network's $\mathbf{s}_{\texttt{DLTrue}}$ or $S_{\texttt{nonDLTrue}}$ are allowed to assume values in $\mathbb{R}$ while training, only the signs of these elements are inherited by the true networks's respective simulated network.

$$
\begin{aligned}
W_{\texttt{DLSimulated}} &= C_{\texttt{DLTrue}} \odot W'_{\texttt{DLSimulated}} \odot \left[\mathbf{1} \otimes \mathbf{s}'_{\texttt{DLTrue}}\right], \\
\text{where } C_{\texttt{DLTrue}} &= \text{binary adjacency matrix from corresponding DLTrue,} \\
W'_{\texttt{DLSimulated}} &= \text{non-negative weight magnitudes of simulated network,} \\
\mathbf{s}'_{\texttt{DLTrue}} &= \text{signs of elements in } \mathbf{s}_{\texttt{DLTrue}}
\end{aligned}
$$

$$
\begin{aligned}
W_{\texttt{nonDLSimulated}} &= C_{\texttt{nonDLTrue}} \odot W'_{\texttt{nonDLSimulated}} \odot S'_{\texttt{nonDLTrue}}, \\
\text{where } C_{\texttt{nonDLTrue}} &= \text{binary adjacency matrix from corresponding NonDLTrue,} \\
W'_{\texttt{nonDLSimulated}} &= \text{non-negative weight magnitudes of simulated network,} \\
S'_{\texttt{nonDLTrue}} &= \text{signs of elements in } S_{\texttt{nonDLTrue}}
\end{aligned}
$$

Extended Data Fig. 8 A shows median hidden-layer tuning correlations for networks trained with and without Dale's law sign constraint for three different architectures.

Training simulated networks that had access to weight magnitudes Three levels of multiplicative noise $\sigma = 0.1, 0.25, 0.5$ were explored, inducing low-noise, medium-noise, and high-noise weight estimates, respectively. Each noise level represents the maximum percentage by which a weight magnitude could be perturbed.

$$
\begin{aligned}
W_{\texttt{Simulated\_Init}} &= X \odot W_{\texttt{True}}, \\
\text{where } X_{ij} &= \text{Uniform}(1-\sigma,\ 1+\sigma)
\end{aligned}
$$

Simulated networks were trained with a Gaussian prior on the weights centered around the noisy initialization. In effect, this additional loss term penalizes trainable weights for deviating from their noisy initialization.

$$
\mathcal{L}_{\texttt{weight\_prior}} = \mathcal{L}_{\texttt{cross\_entropy}} + \lambda \sum \left[W_{\texttt{Simulated}} - W_{\texttt{Simulated\_Init}}\right]^2
$$

Extended Data Fig. 8b shows median hidden-layer tuning correlations for networks with low-, medium-, and high-noise weight perturbations.

References for appendix

[88] Gruntman, E., Romani, S. & Reiser, M. B. Simple integration of fast excitation and offset, delayed inhibition computes directional selectivity in Drosophila. *Nature Neuroscience* **21**, 250–257 (2018).

[89] Takemura, S.-y. *et al.* Synaptic circuits and their variations within different columns in the visual system of Drosophila . *Proceedings of the National Academy of Sciences* **112**, 13711–13716 (2015).

[90] Shinomiya, K. *et al.* Comparisons between the ON- and OFF-edge motion pathways in the Drosophila brain. *Elife* **8**, 2431 (2019).

[91] Tschopp, F. D., Reiser, M. B. & Turaga, S. C. A Connectome Based Hexagonal Lattice Convolutional Network Model of the Drosophila Visual System. *arXiv preprint arXiv:1806.04793* (2018). `1806.04793`.

[92] Rivera-Alba, M. *et al.* Wiring economy and volume exclusion determine neuronal placement in the drosophila brain. *Current Biology* **21**, 2000–2005 (2011).

[93] Tuthill, J. C., Nern, A., Holtz, S. L., Rubin, G. M. & Reiser, M. B. Contributions of the 12 Neuron Classes in the Fly Lamina to Motion Vision. *Neuron* **79**, 128–140 (2013).

[94] Tuthill, J. C., Nern, A., Rubin, G. M. & Reiser, M. B. Wide-field feedback neurons dynamically tune early visual processing. *Neuron* **82**, 887–895 (2014).

[95] Takemura, S.-y. *et al.* The comprehensive connectome of a neural substrate for 'ON' motion detection in Drosophila. *eLife* **6**, 1–16 (2017).

[96] Meier, M. & Borst, A. Extreme Compartmentalization in a Drosophila Amacrine Cell. *Current Biology* **29**, 1545–1550.e2 (2019).

[97] Scheffer, L. K. *et al.* A connectome and analysis of the adult drosophila central brain. *Elife* **9**, e57443 (2020).

[98] Peretz, A. *et al.* The light response of drosophila photoreceptors is accompanied by an increase in cellular calcium: effects of specific mutations. *Neuron* **12**, 1257–1267 (1994).

[99] Reiff, D. F., Plett, J., Mank, M., Griesbeck, O. & Borst, A. Visualizing retinotopic half-wave rectified input to the motion detection circuitry of drosophila. *Nature neuroscience* **13**, 973–978 (2010).

[100] Clark, D. A., Bursztyn, L., Horowitz, M. A., Schnitzer, M. J. & Clandinin, T. R. Defining the computational structure of the motion detector in drosophila. *Neuron* **70**, 1165–1177 (2011).

[101] Freifeld, L., Clark, D. A., Schnitzer, M. J., Horowitz, M. A. & Clandinin, T. R. Gabaergic lateral interactions tune the early stages of visual processing in drosophila. *Neuron* **78**, 1075–1089 (2013).

[102] Silies, M. *et al.* Modular use of peripheral input channels tunes motion-detecting circuitry. *Neuron* **79**, 111–127 (2013).

[103] Maisak, M. S. *et al.* A directional tuning map of Drosophila elementary motion detectors. *Nature* **500**, 212–216 (2013).

[104] Strother, J. A., Nern, A. & Reiser, M. B. Direct observation of on and off pathways in the drosophila visual system. *Current Biology* **24**, 976–983 (2014).

[105] Meier, M. *et al.* Neural circuit components of the drosophila off motion vision pathway. *Current Biology* **24**, 385–392 (2014).

[106] Behnia, R., Clark, D. A., Carter, A. G., Clandinin, T. R. & Desplan, C. Processing properties of ON and OFF pathways for drosophila motion detection. *Nature* **512**, 427–430 (2014).

[107] Fisher, Y. E. *et al.* A Class of Visual Neurons with Wide-Field Properties Is Required for Local Motion Detection. *Current Biology* **25**, 3178–3189 (2015).

[108] Hardie, R. C. & Juusola, M. Phototransduction in drosophila. *Current opinion in neurobiology* **34**, 37–45 (2015).

[109] Leonhardt, A. *et al.* Asymmetry of drosophila on and off motion detectors enhances real-world velocity estimation. *Nature neuroscience* **19**, 706–715 (2016).

[110] Fisher, Y. E., Silies, M. & Clandinin, T. R. Orientation Selectivity Sharpens Motion Detection in Drosophila. *Neuron* **88**, 390–402 (2015).

[111] Yang, H. H. *et al.* Subcellular imaging of voltage and calcium signals reveals neural processing in vivo. *Cell* **166**, 245–257 (2016).

[112] Serbe, E., Meier, M., Leonhardt, A. & Borst, A. Comprehensive characterization of the major presynaptic elements to the drosophila off motion detector. *Neuron* **89**, 829–841 (2016).

[113] Strother, J. A. *et al.* The emergence of directional selectivity in the visual motion pathway of drosophila. *Neuron* **94**, 168–182 (2017).

[114] Arenz, A., Drews, M. S., Richter, F. G., Ammer, G. & Borst, A. The Temporal Tuning of the Drosophila Motion Detectors Is Determined by the Dynamics of Their Input Elements. *Current Biology* **27**, 929–944 (2017).

[115] Gruntman, E., Romani, S. & Reiser, M. B. The computation of directional selectivity in the drosophila off motion pathway. *Elife* **8**, e50706 (2019).

[116] Drews, M. S. *et al.* Dynamic signal compression for robust motion vision in flies. *Curr. Biol.* **30**, 209–221.e8 (2020).

[117] Matulis, C. A., Chen, J., Gonzalez-Suarez, A. D., Behnia, R. & Clark, D. A. Heterogeneous temporal contrast adaptation in drosophila Direction-Selective circuits. *Curr. Biol.* **30**, 222–236.e6 (2020).

[118] Ramos-Traslosheros, G. & Silies, M. The physiological basis for contrast opponency in motion computation in drosophila. *Nature communications* **12**, 1–16 (2021).

[119] Gruntman, E., Reimers, P., Romani, S. & Reiser, M. B. Non-preferred contrast responses in the drosophila motion pathways reveal a receptive field structure that explains a common visual illusion. *Current Biology* **31**, 5286–5298 (2021).

[120] Groschner, L. N., Malis, J. G., Zuidinga, B. & Borst, A. A biophysical account of multiplication by a single neuron. *Nature* **603**, 119–123 (2022).

[121] Ketkar, M. D. *et al.* First-order visual interneurons distribute distinct contrast and luminance information across on and off pathways to achieve stable behavior. *Elife* **11**, e74937 (2022).

[122] He, K., Zhang, X., Ren, S. & Sun, J. Delving deep into rectifiers: Surpassing human-level performance on imagenet classification. In *Proceedings of the IEEE international conference on computer vision*, 1026–1034 (2015).
