# Supplementary material for: Connectome-constrained networks predict neural activity across the fly visual system
Source: Nature. 2024 Sep 11;634(8036):1132–40. doi: 10.1038/s41586-024-07939-3 (PMC11525180; doi:10.1038/s41586-024-07939-3)
Supplement: Supplementary file 3 — This zipped data folder contains seven files: (1) connectome type-to-type filters, average synapse count; (2) source references for the compiled connectome; (3) cell type-to-type connection matrix; (4) single-neuron-resolution connectome for 91 columns; (5) source references to 26 studies reporting neural activity measurements; (6) BibTeX file of 26 neural activity measurement studies; (7) catalogue of neural response predictions for all 64 cell types. [file 41586_2024_7939_MOESM3_ESM.zip › 2023-03-04080C-Supplementary Data/Lappalainen_et_al_all_cell_type_neural_activity_predictions.pdf]

Cell type

1 R1 3

2 R2 10

3 R3 16

4 R4 23

5 R5 30

6 R6 37

7 R7 44

8 R8 50

9 L1 56

10 L2 64

11 L3 71

12 L4 79

13 L5 86

14 Lawf1 94

15 Lawf2 101

16 Am 108

17 C2 116

18 C3 123

19 CT1(Lo1) 131

20 CT1(M10) 139

21 Mi1 147

22 Mi2 154

23 Mi3 160

24 Mi4 167

25 Mi9 174

26 Mi10 181

27 Mi11 189

28 Mi12 195

29 Mi13 202

30 Mi14 208

31 Mi15 215

32 T1 221

33	T2	228
34	T2a	236
35	T3	244
36	T4a	250
37	T4b	258
38	T4c	266
39	T4d	274
40	T5a	282
41	T5b	290
42	T5c	298
43	T5d	306
44	Tm1	314
45	Tm2	322
46	Tm3	330
47	Tm4	338
48	Tm5Y	346
49	Tm5a	353
50	Tm5b	360
51	Tm5c	367
52	Tm9	373
53	Tm16	381
54	Tm20	389
55	Tm28	396
56	Tm30	403
57	TmY3	410
58	TmY4	418
59	TmY5a	426
60	TmY9	434
61	TmY10	441
62	TmY13	448
63	TmY14	455
64	TmY15	463
65	TmY18	470

1 R1

← Cell types

Figures

1 Anatomical receptive fields. . . . 3
2 Anatomical projective fields. . . . 3
3 Clustering of the responses to naturalistic stimuli. . . . 4
4 Responses to flashes. . . . 4
5 Cluster-average responses to single-ommatidium flashes. . . . 4
6 Peak responses to moving edges. . . . 5
7 Peak responses to moving edges from task-optimal models. . . . 5
8 Responses to moving edges from task-optimal models. . . . 6
9 Peak responses to moving bars. . . . 6
10 Peak responses to moving bars from task-optimal models. . . . 7
11 Responses to moving bars from task-optimal models. . . . 7
12 Spatio-temporal receptive field. . . . 8
13 Maximally excitatory stimuli. . . . 8
14 Task-constrained parameters. . . . 9

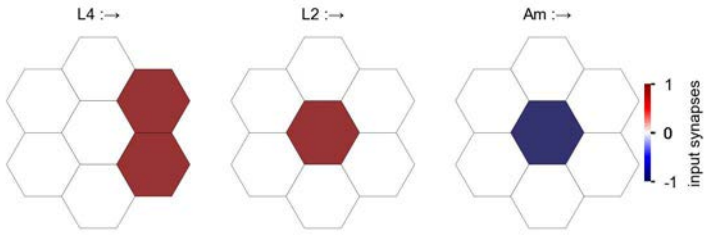

R1 - Figure 1: **Anatomical receptive fields.** Each colored hexagon is an input connection, with the connection strength characterized by the average number of synapses that we count from the EM reconstruction. Red indicates excitatory synapses, blue indicates inhibitory synapses from inferred signs. Filters in the order of their total number of synapses.

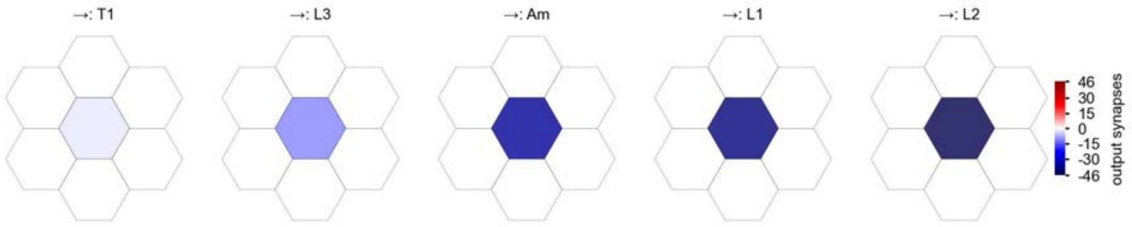

R1 - Figure 2: **Anatomical projective fields.** Each colored hexagon is an output connection, with the connection strength characterized by the average number of synapses that we count from the EM reconstruction. Red indicates excitatory synapses, blue indicates inhibitory synapses from inferred signs. Filters in the order of their total number of synapses.

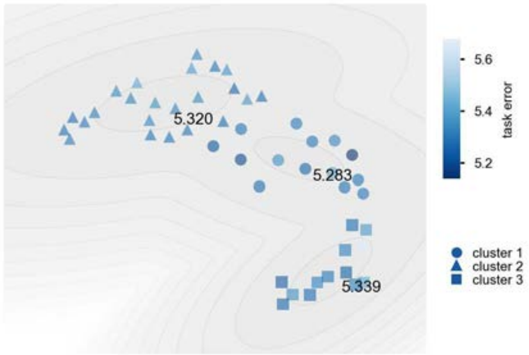

R1 - Figure 3: **Clustering of the responses to naturalistic stimuli.** Clustering of the 50 models based on the cell type responses to naturalistic scenes from the Sintel dataset. Scatterpoints represent individual models colored by their task error.

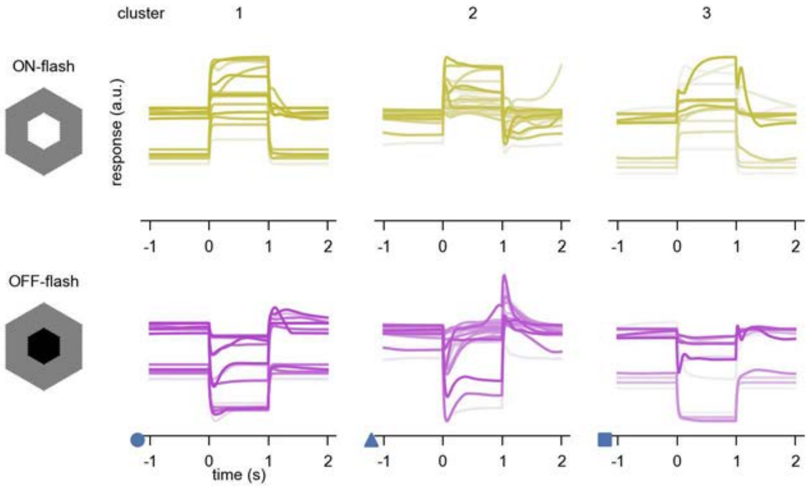

R1 - Figure 4: **Responses to flashes.** The top row shows responses to ON-flashes (yellow), the bottom row shows responses to OFF-flashes (magenta). The responses from the 50 different models that are separated into the different clusters (columns) overlay, with better task-performing models on top. Responses from better task-performing models are more saturated. The circular flashes (1s) cover 6 ommatidia in radius and are presented at time zero. Before and after, a grey-stimulus leads to a stationary state of the network.

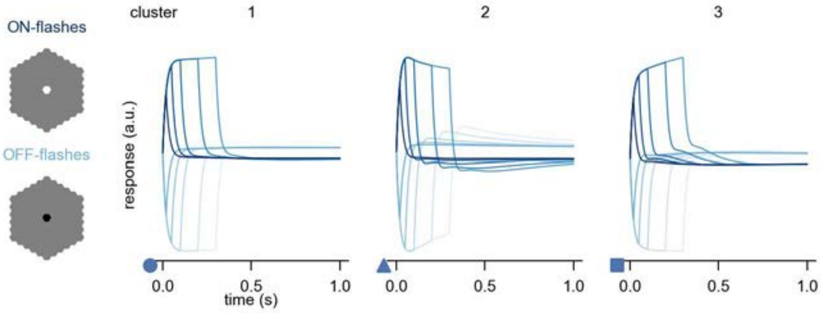

R1 - Figure 5: **Cluster-average responses to single-ommatidium flashes.** Responses to single-ommatidium ON-flashes (dark blue shades) and single-ommatidium OFF-flashes (light blue shades) of 20ms, 50ms, 100ms, 200ms, 300ms duration. The flashes occur at second zero.

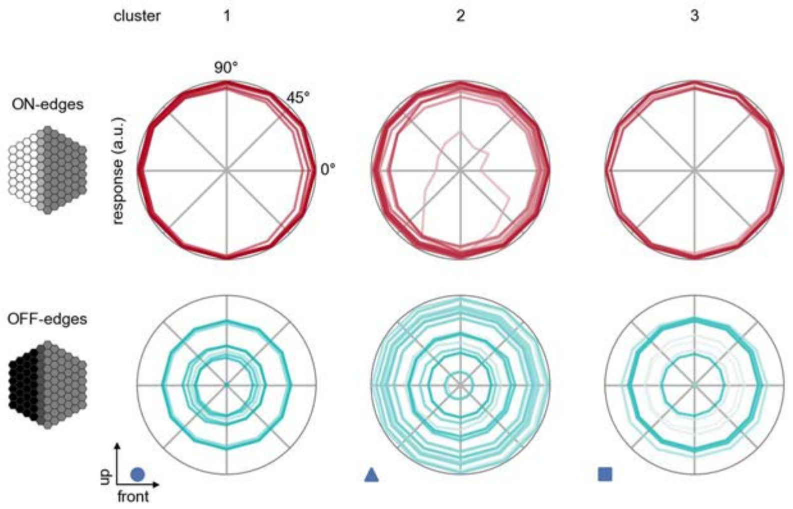

R1 - Figure 6: **Peak responses to moving edges.** The top row shows peak responses to moving ON-edges (red), the bottom row shows peak responses to moving OFF-edges (turqoise). The peak responses are averaged over edge-speeds. Edge-stimuli move in different directions from 0 to 360 degrees. The responses from the different models in the different clusters (columns) overlay. Responses from better task-performing models are more saturated.

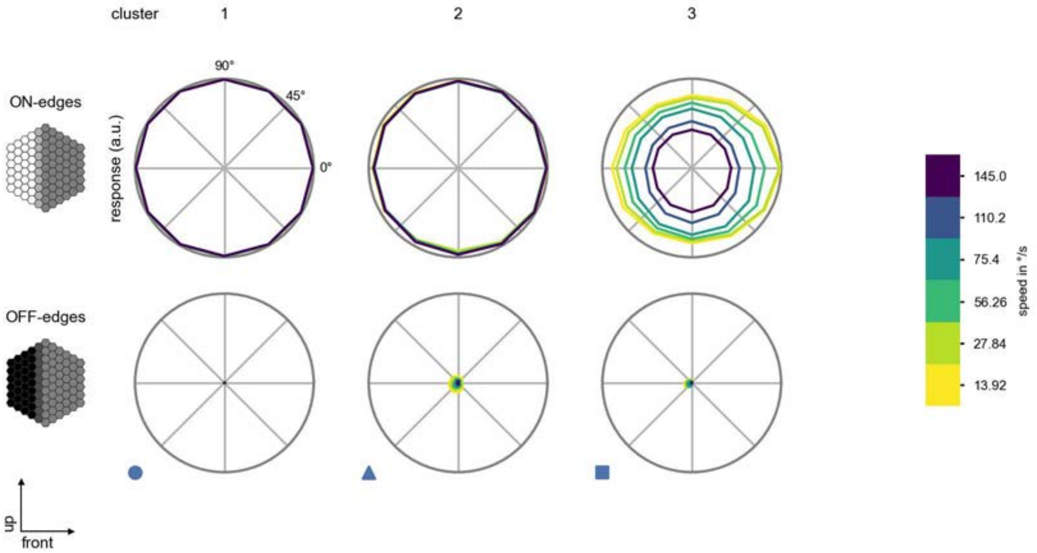

R1 - Figure 7: **Peak responses to moving edges from task-optimal models.** The top row shows peak responses to moving ON-edges, the bottom row shows peak responses to moving OFF-edges of varying speeds from 13.92°/s to 145°/s (yellow to dark blue). The edge-stimuli move in different directions from 0 to 360 degrees and at different speeds. Responses from the task-optimal model in the respective cluster.

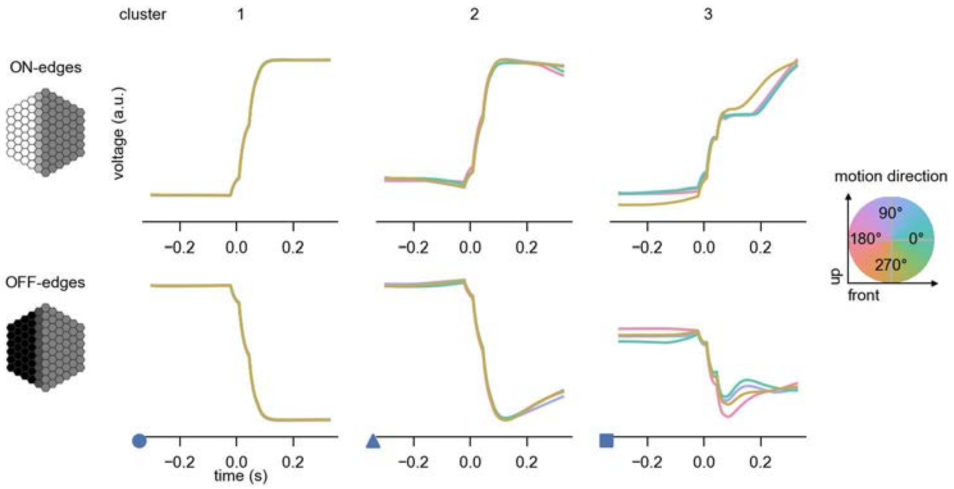

R1 - Figure 8: **Responses to moving edges from task-optimal models.** Responses to moving ON-edges (top row) and to moving OFF-edges (bottom row). Edges move in different directions from 0 to 360 degrees and at different speeds. Responses are from the task-optimal model in the respective cluster. Edges moving at 75.4°/s in all cardinal directions (green 0°, blue 90°, red 180°, yellow 270°) from -22.5 to 22.5° visual angle.

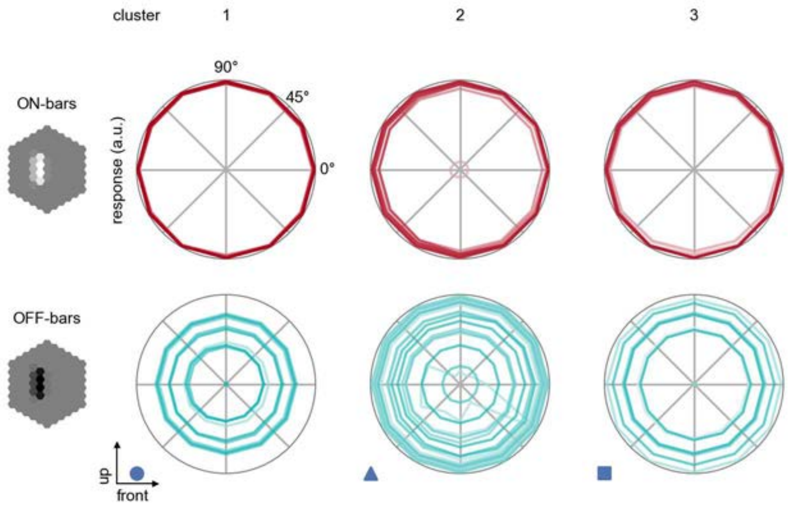

R1 - Figure 9: **Peak responses to moving bars.** The top row shows peak responses to moving ON-bars (red), the bottom row shows peak responses to moving OFF-bars (turqoise). The peak responses are averaged over bar-speeds. Bar-stimuli move in different directions from 0 to 360 degrees. The responses from the different models in the different clusters (columns) overlay. Responses from better task-performing models are more saturated.

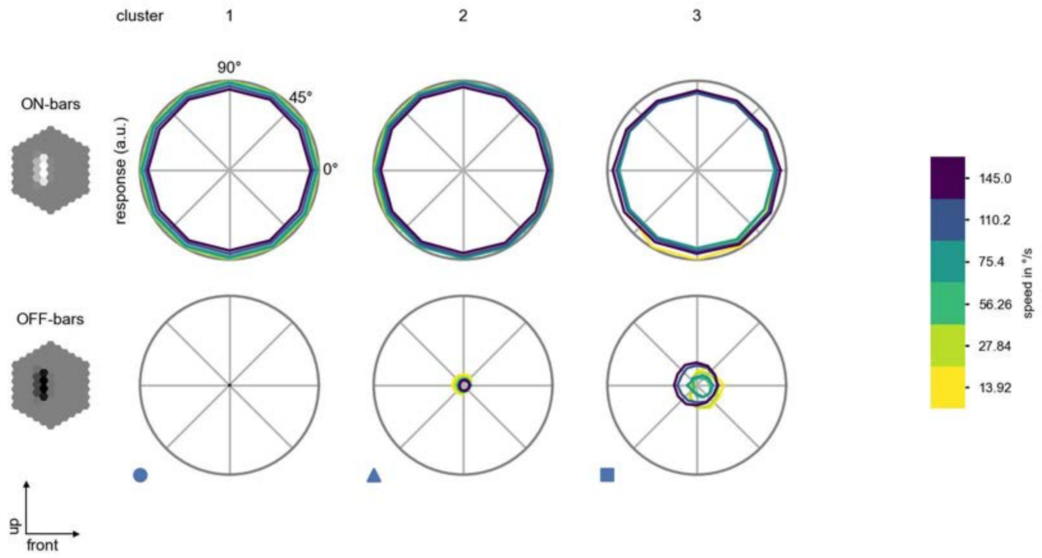

R1 - Figure 10: **Peak responses to moving bars from task-optimal models.** The top row shows peak responses to moving ON-bars, the bottom row shows peak responses to moving OFF-bars of varying speeds from 13.92°/s to 145°/s (yellow to dark blue). The bar-stimuli move in different directions from 0 to 360 degrees and at different speeds. Responses from the task-optimal model in the respective cluster.

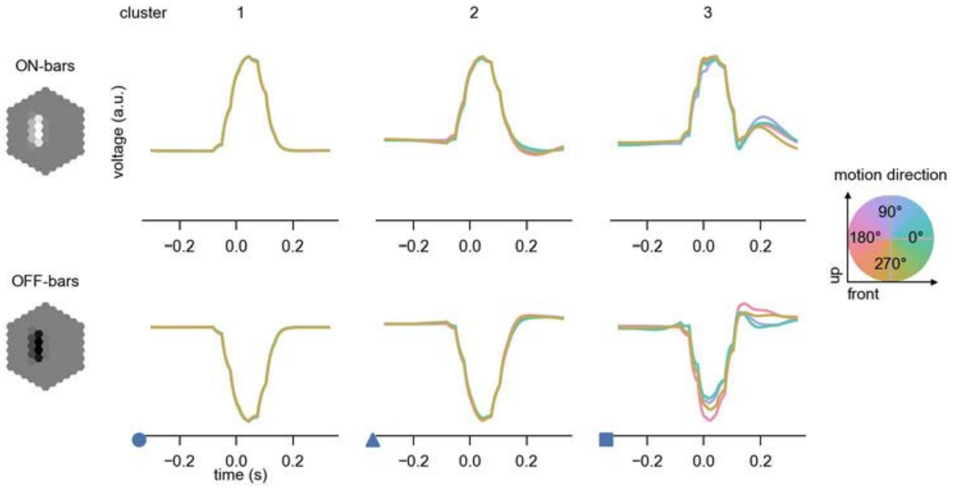

R1 - Figure 11: **Responses to moving bars from task-optimal models.** Responses to moving ON-bars (top row) and to moving OFF-bars (bottom row). Bars move in different directions from 0 to 360 degrees and at different speeds. Responses are from the task-optimal model in the respective cluster. Bars moving at 75.4°/s in all cardinal directions (green 0°, blue 90°, red 180°, yellow 270°) from -22.5 to 22.5° visual angle.

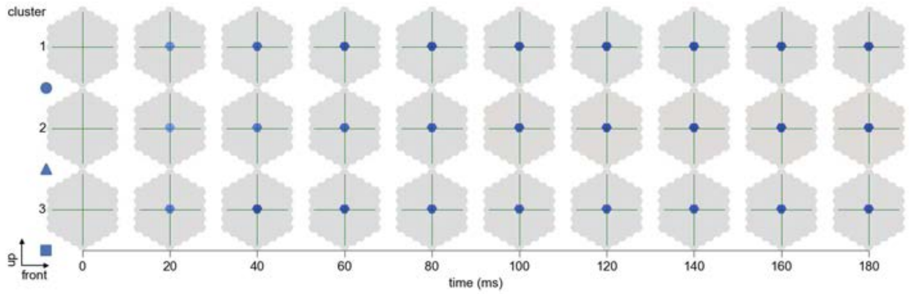

R1 - Figure 12: **Spatio-temporal receptive field.** Responses of the central cell to ON-impulses (5 ms) at single-ommatidium flash locations. The flash occurs at second zero. Responses from the task-optimal model of the respective cluster (rows). Red indicates depolarization, blue indicates hyperpolarization.

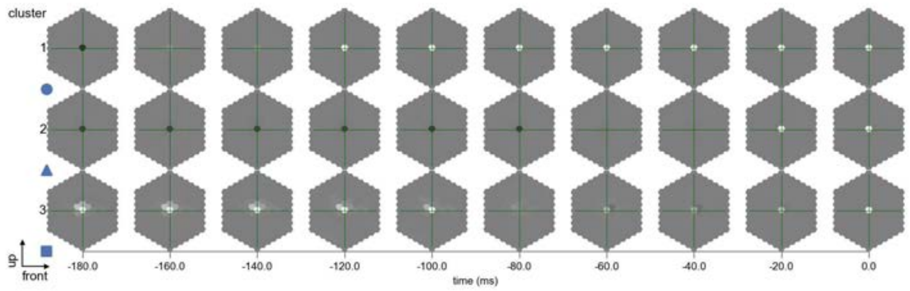

R1 - Figure 13: **Maximally excitatory stimuli.** Each row presents the regularized naturalistic-stimulus from the Sintel dataset that maximizes the cell type's central column response at second zero in the task-optimal model of the respective cluster (rows).

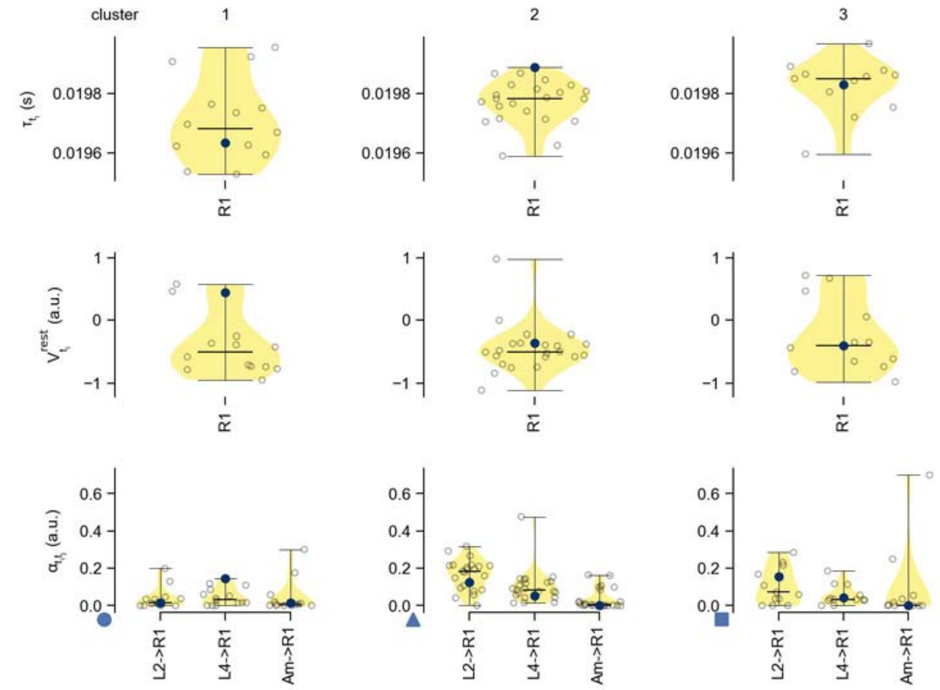

R1 - Figure 14: **Task-constrained parameters.** Each column shows the parameters inferred within the respective cluster. First row: learned time constants of the cell type. Second row: resting potentials of the cell type. Third row: scaling factors for the convolutional filters. The blue scatter represents the parameters from the task-optimal model within the cluster.

2 R2

← Cell types

Figures

1 Anatomical receptive fields. 10
2 Anatomical projective fields. 10
3 Clustering of the responses to naturalistic stimuli. 11
4 Responses to flashes. 11
5 Cluster-average responses to single-ommatidium flashes. 11
6 Peak responses to moving edges. 12
7 Peak responses to moving edges from task-optimal models. 12
8 Responses to moving edges from task-optimal models. 13
9 Peak responses to moving bars. 13
10 Peak responses to moving bars from task-optimal models. 14
11 Responses to moving bars from task-optimal models. 14
12 Spatio-temporal receptive field. 14
13 Maximally excitatory stimuli. 15
14 Task-constrained parameters. 15

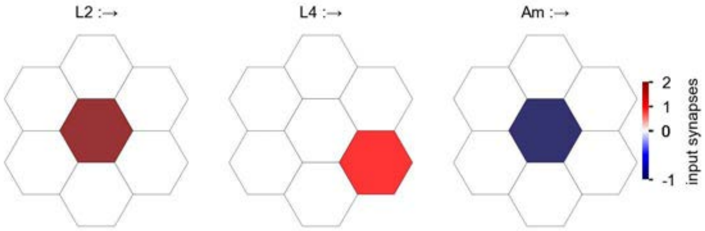

R2 - Figure 1: **Anatomical receptive fields.** Each colored hexagon is an input connection, with the connection strength characterized by the average number of synapses that we count from the EM reconstruction. Red indicates excitatory synapses, blue indicates inhibitory synapses from inferred signs. Filters in the order of their total number of synapses.

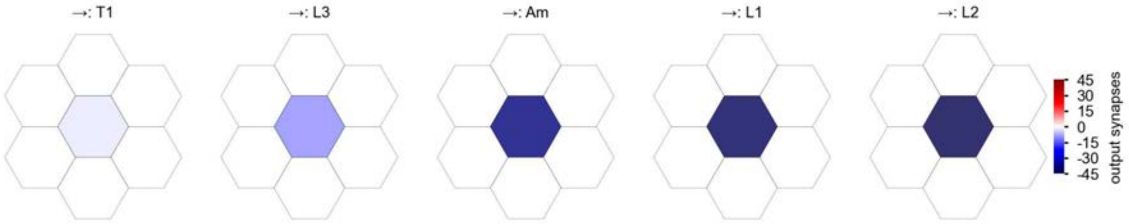

R2 - Figure 2: **Anatomical projective fields.** Each colored hexagon is an output connection, with the connection strength characterized by the average number of synapses that we count from the EM reconstruction. Red indicates excitatory synapses, blue indicates inhibitory synapses from inferred signs. Filters in the order of their total number of synapses.

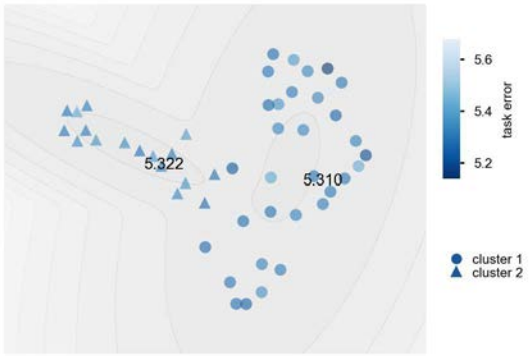

R2 - Figure 3: **Clustering of the responses to naturalistic stimuli.** Clustering of the 50 models based on the cell type responses to naturalistic scenes from the Sintel dataset. Scatterpoints represent individual models colored by their task error.

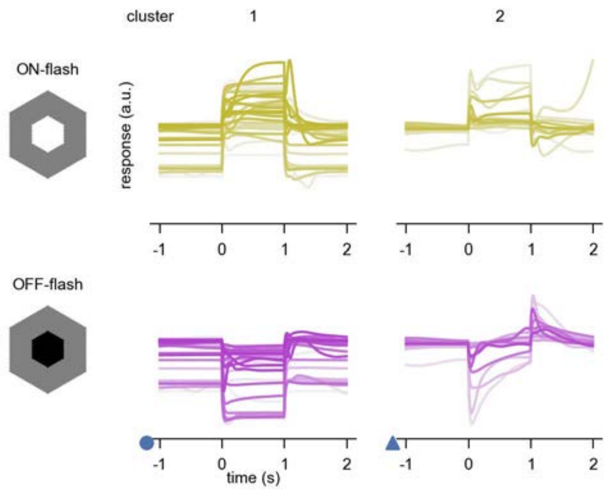

R2 - Figure 4: **Responses to flashes.** The top row shows responses to ON-flashes (yellow), the bottom row shows responses to OFF-flashes (magenta). The responses from the 50 different models that are separated into the different clusters (columns) overlay, with better task-performing models on top. Responses from better task-performing models are more saturated. The circular flashes (1s) cover 6 ommatidia in radius and are presented at time zero. Before and after, a grey-stimulus leads to a stationary state of the network.

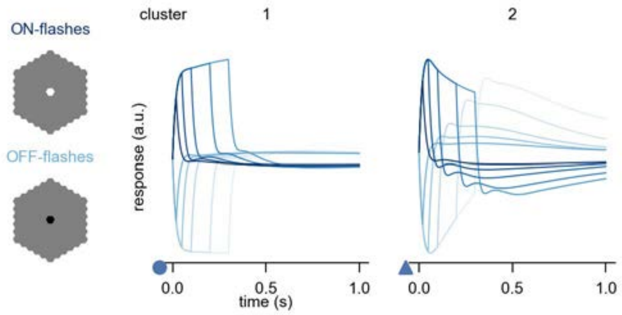

R2 - Figure 5: **Cluster-average responses to single-ommatidium flashes.** Responses to single-ommatidium ON-flashes (dark blue shades) and single-ommatidium OFF-flashes (light blue shades) of 20ms, 50ms, 100ms, 200ms, 300ms duration. The flashes occur at second zero.

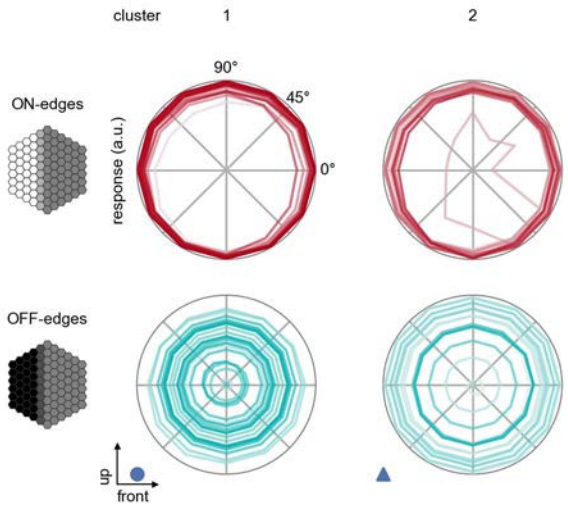

R2 - Figure 6: **Peak responses to moving edges.** The top row shows peak responses to moving ON-edges (red), the bottom row shows peak responses to moving OFF-edges (turqoise). The peak responses are averaged over edge-speeds. Edge-stimuli move in different directions from 0 to 360 degrees. The responses from the different models in the different clusters (columns) overlay. Responses from better task-performing models are more saturated.

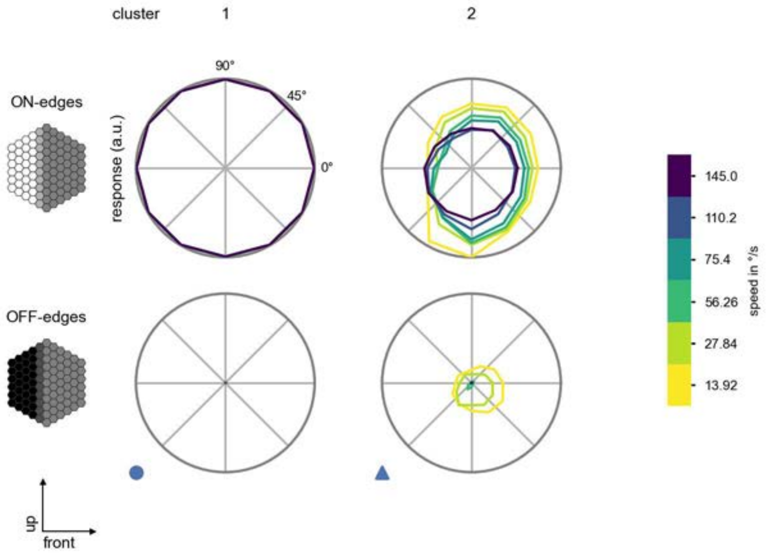

R2 - Figure 7: **Peak responses to moving edges from task-optimal models.** The top row shows peak responses to moving ON-edges, the bottom row shows peak responses to moving OFF-edges of varying speeds from 13.92°/s to 145°/s (yellow to dark blue). The edge-stimuli move in different directions from 0 to 360 degrees and at different speeds. Responses from the task-optimal model in the respective cluster.

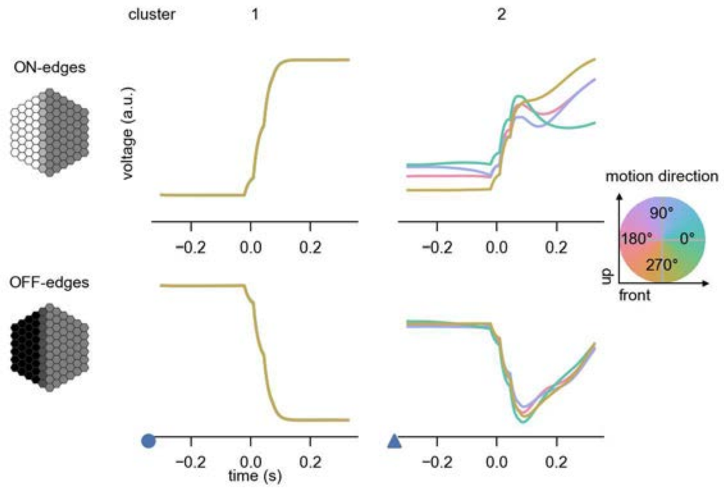

R2 - Figure 8: **Responses to moving edges from task-optimal models.** Responses to moving ON-edges (top row) and to moving OFF-edges (bottom row). Edges move in different directions from 0 to 360 degrees and at different speeds. Responses are from the task-optimal model in the respective cluster. Edges moving at 75.4°/s in all cardinal directions (green 0°, blue 90°, red 180°, yellow 270°) from -22.5 to 22.5° visual angle.

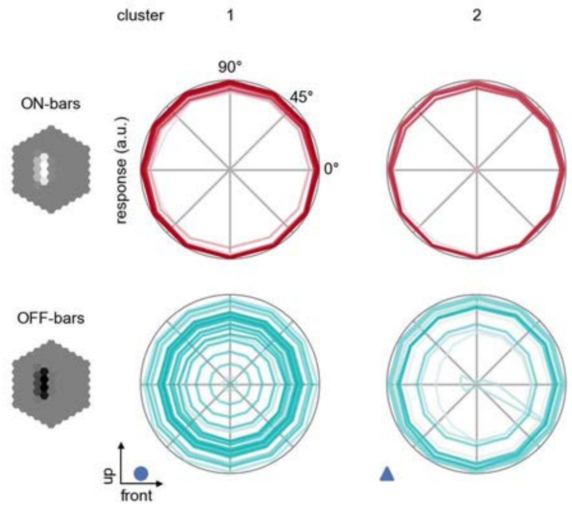

R2 - Figure 9: **Peak responses to moving bars.** The top row shows peak responses to moving ON-bars (red), the bottom row shows peak responses to moving OFF-bars (turqoise). The peak responses are averaged over bar-speeds. Bar-stimuli move in different directions from 0 to 360 degrees. The responses from the different models in the different clusters (columns) overlay. Responses from better task-performing models are more saturated.

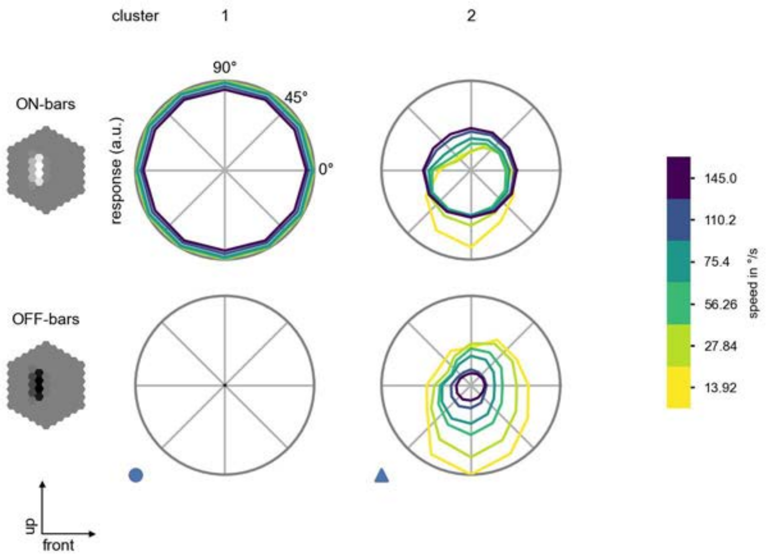

R2 - Figure 10: **Peak responses to moving bars from task-optimal models.** The top row shows peak responses to moving ON-bars, the bottom row shows peak responses to moving OFF-bars of varying speeds from 13.92°/s to 145°/s (yellow to dark blue). The bar-stimuli move in different directions from 0 to 360 degrees and at different speeds. Responses from the task-optimal model in the respective cluster.

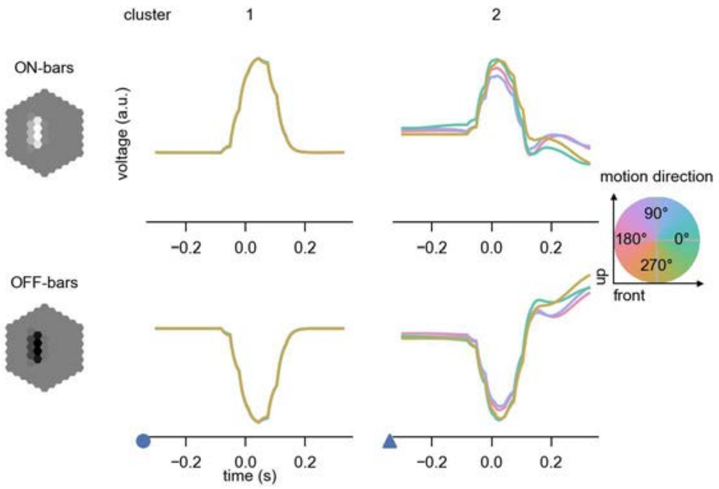

R2 - Figure 11: **Responses to moving bars from task-optimal models.** Responses to moving ON-bars (top row) and to moving OFF-bars (bottom row). Bars move in different directions from 0 to 360 degrees and at different speeds. Responses are from the task-optimal model in the respective cluster. Bars moving at 75.4°/s in all cardinal directions (green 0°, blue 90°, red 180°, yellow 270°) from -22.5 to 22.5° visual angle.

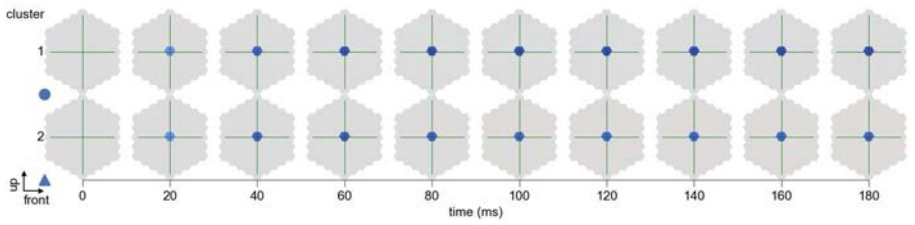

R2 - Figure 12: **Spatio-temporal receptive field.** Responses of the central cell to ON-impulses (5 ms) at single-ommatidium flash locations. The flash occurs at second zero. Responses from the task-optimal model of the respective cluster (rows). Red indicates depolarization, blue indicates hyperpolarization.

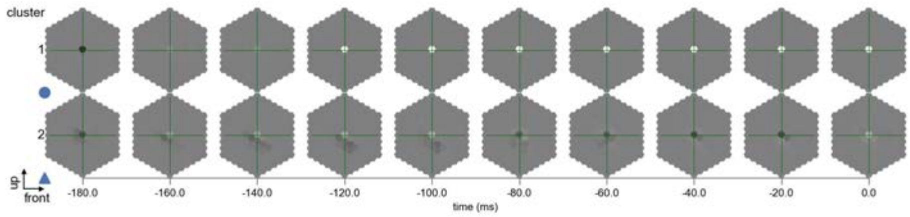

R2 - Figure 13: **Maximally excitatory stimuli.** Each row presents the regularized naturalistic-stimulus from the Sintel dataset that maximizes the cell type's central column response at second zero in the task-optimal model of the respective cluster (rows).

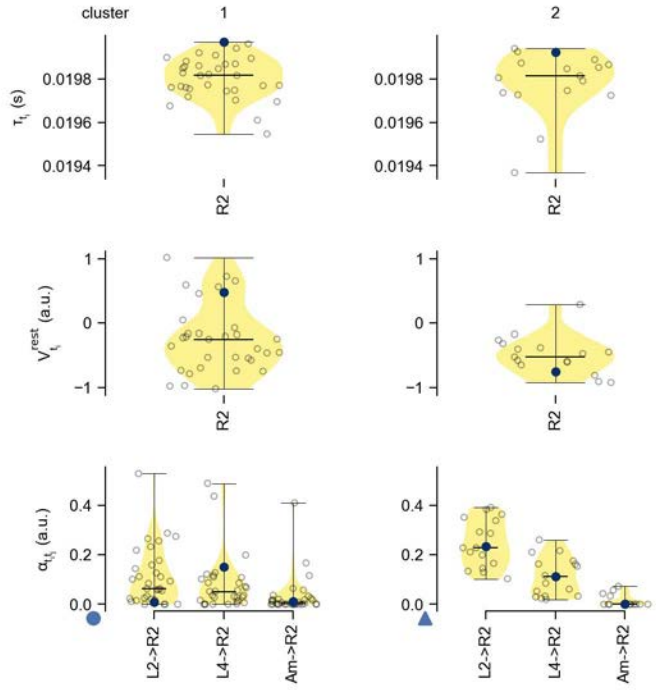

R2 - Figure 14: **Task-constrained parameters.** Each column shows the parameters inferred within the respective cluster. First row: learned time constants of the cell type. Second row: resting potentials of the cell type. Third row: scaling factors for the convolutional filters. The blue scatter represents the parameters from the task-optimal model within the cluster.

3 R3

← Cell types

Figures

1 Anatomical receptive fields. 16
2 Anatomical projective fields. 16
3 Clustering of the responses to naturalistic stimuli. 17
4 Responses to flashes. 17
5 Cluster-average responses to single-ommatidium flashes. 17
6 Peak responses to moving edges. 18
7 Peak responses to moving edges from task-optimal models. 18
8 Responses to moving edges from task-optimal models. 19
9 Peak responses to moving bars. 19
10 Peak responses to moving bars from task-optimal models. 20
11 Responses to moving bars from task-optimal models. 20
12 Spatio-temporal receptive field. 21
13 Maximally excitatory stimuli. 21
14 Task-constrained parameters. 22

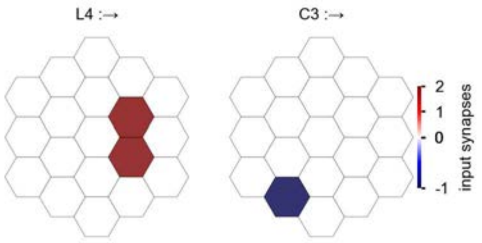

R3 - Figure 1: **Anatomical receptive fields.** Each colored hexagon is an input connection, with the connection strength characterized by the average number of synapses that we count from the EM reconstruction. Red indicates excitatory synapses, blue indicates inhibitory synapses from inferred signs. Filters in the order of their total number of synapses.

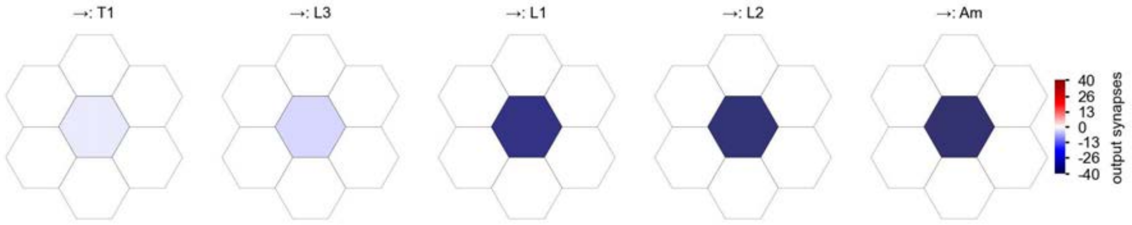

R3 - Figure 2: **Anatomical projective fields.** Each colored hexagon is an output connection, with the connection strength characterized by the average number of synapses that we count from the EM reconstruction. Red indicates excitatory synapses, blue indicates inhibitory synapses from inferred signs. Filters in the order of their total number of synapses.

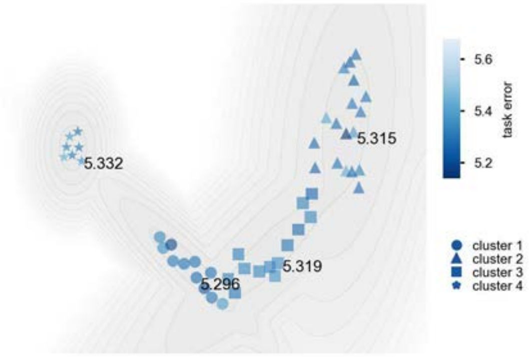

R3 - Figure 3: **Clustering of the responses to naturalistic stimuli.** Clustering of the 50 models based on the cell type responses to naturalistic scenes from the Sintel dataset. Scatterpoints represent individual models colored by their task error.

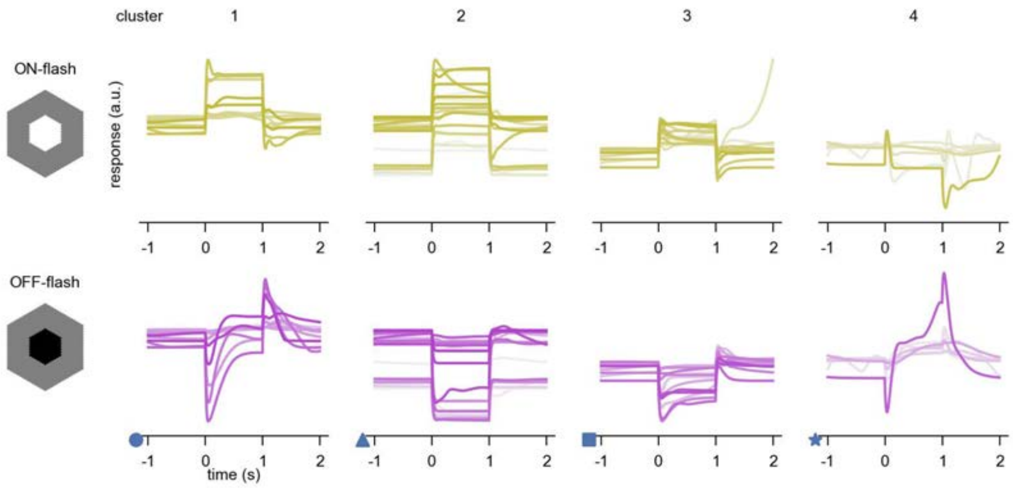

R3 - Figure 4: **Responses to flashes.** The top row shows responses to ON-flashes (yellow), the bottom row shows responses to OFF-flashes (magenta). The responses from the 50 different models that are separated into the different clusters (columns) overlay, with better task-performing models on top. Responses from better task-performing models are more saturated. The circular flashes (1s) cover 6 ommatidia in radius and are presented at time zero. Before and after, a grey-stimulus leads to a stationary state of the network.

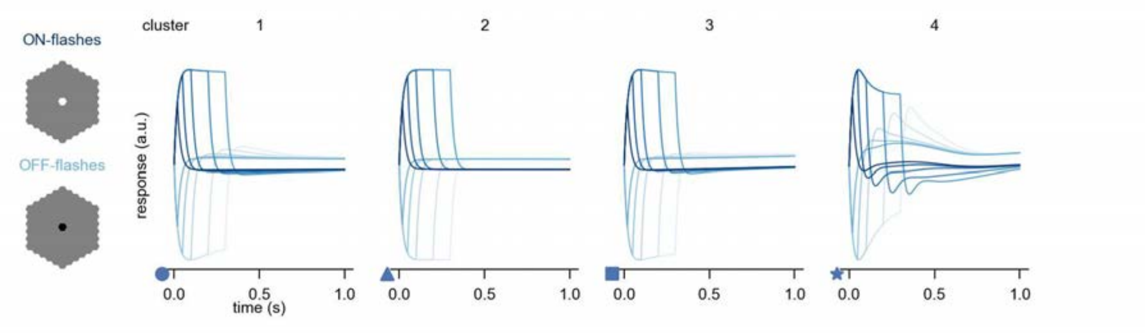

R3 - Figure 5: **Cluster-average responses to single-ommatidium flashes.** Responses to single-ommatidium ON-flashes (dark blue shades) and single-ommatidium OFF-flashes (light blue shades) of 20ms, 50ms, 100ms, 200ms, 300ms duration. The flashes occur at second zero.

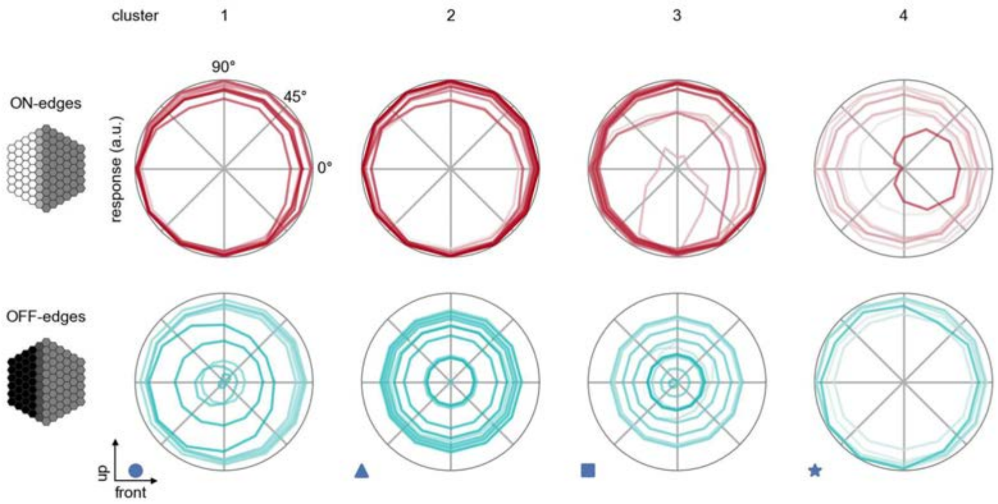

R3 - Figure 6: **Peak responses to moving edges.** The top row shows peak responses to moving ON-edges (red), the bottom row shows peak responses to moving OFF-edges (turqoise). The peak responses are averaged over edge-speeds. Edge-stimuli move in different directions from 0 to 360 degrees. The responses from the different models in the different clusters (columns) overlay. Responses from better task-performing models are more saturated.

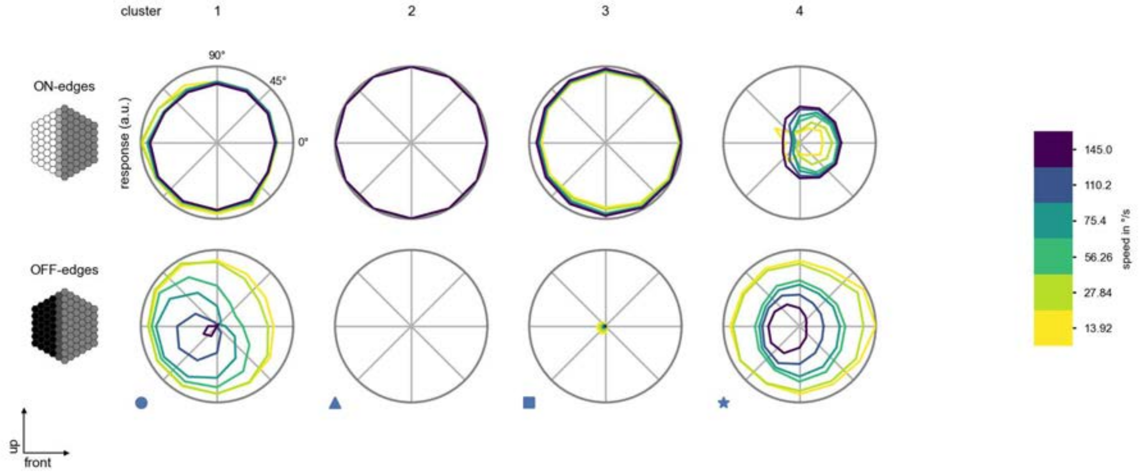

R3 - Figure 7: **Peak responses to moving edges from task-optimal models.** The top row shows peak responses to moving ON-edges, the bottom row shows peak responses to moving OFF-edges of varying speeds from 13.92°/s to 145°/s (yellow to dark blue). The edge-stimuli move in different directions from 0 to 360 degrees and at different speeds. Responses from the task-optimal model in the respective cluster.

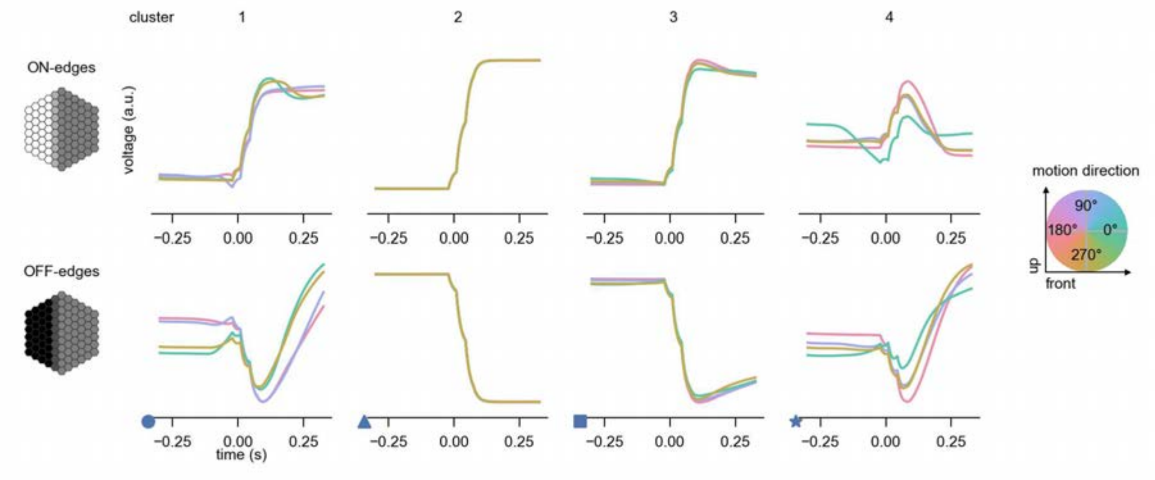

R3 - Figure 8: **Responses to moving edges from task-optimal models.** Responses to moving ON-edges (top row) and to moving OFF-edges (bottom row). Edges move in different directions from 0 to 360 degrees and at different speeds. Responses are from the task-optimal model in the respective cluster. Edges moving at 75.4°/s in all cardinal directions (green 0°, blue 90°, red 180°, yellow 270°) from -22.5 to 22.5° visual angle.

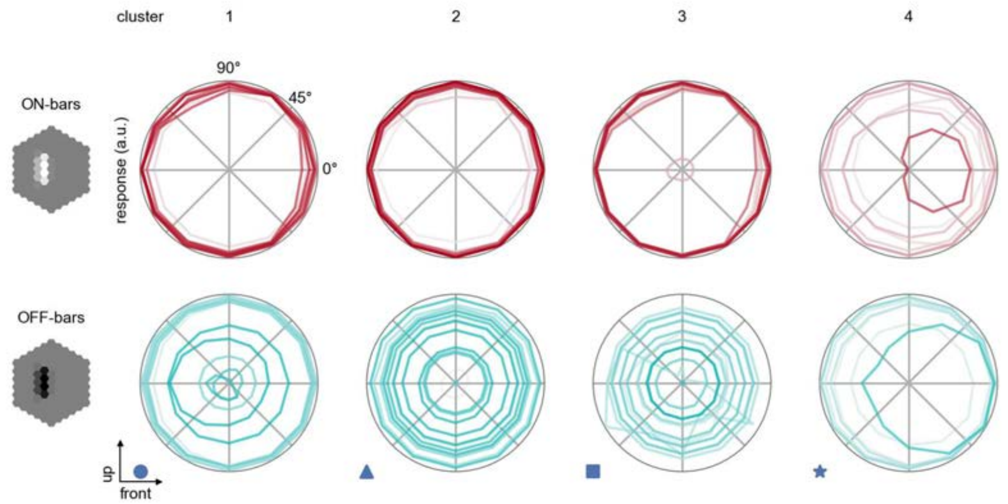

R3 - Figure 9: **Peak responses to moving bars.** The top row shows peak responses to moving ON-bars (red), the bottom row shows peak responses to moving OFF-bars (turqoise). The peak responses are averaged over bar-speeds. Bar-stimuli move in different directions from 0 to 360 degrees. The responses from the different models in the different clusters (columns) overlay. Responses from better task-performing models are more saturated.

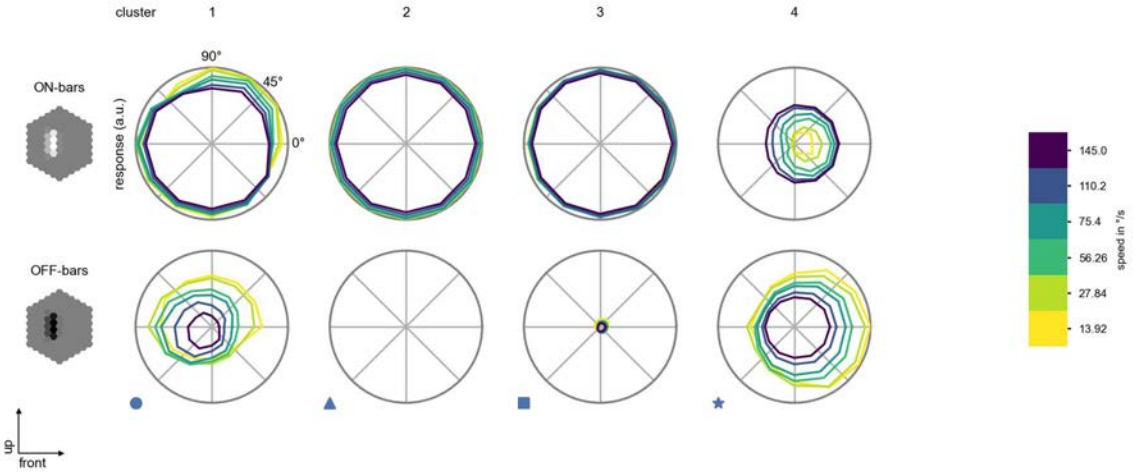

R3 - Figure 10: **Peak responses to moving bars from task-optimal models.** The top row shows peak responses to moving ON-bars, the bottom row shows peak responses to moving OFF-bars of varying speeds from 13.92°/s to 145°/s (yellow to dark blue). The bar-stimuli move in different directions from 0 to 360 degrees and at different speeds. Responses from the task-optimal model in the respective cluster.

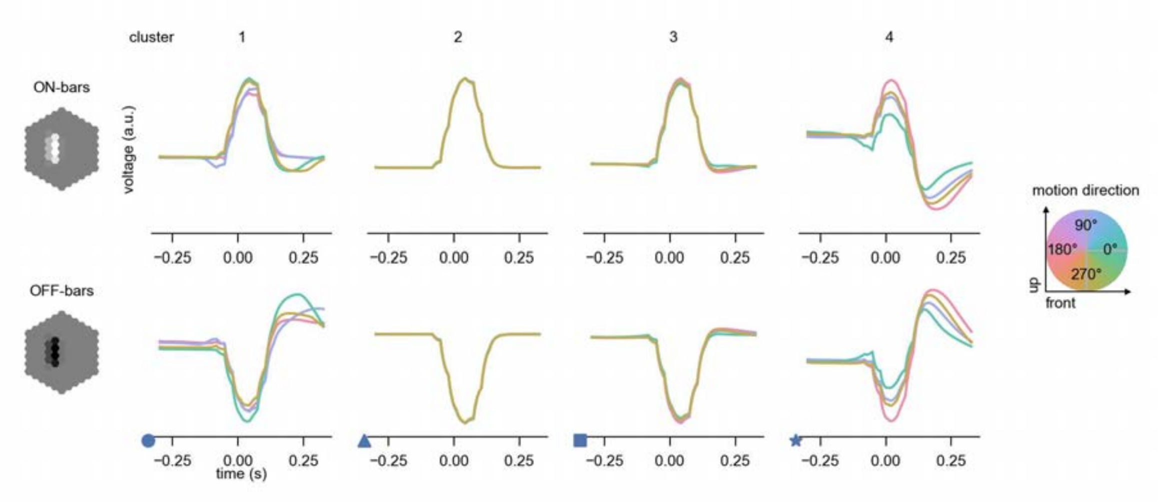

R3 - Figure 11: **Responses to moving bars from task-optimal models.** Responses to moving ON-bars (top row) and to moving OFF-bars (bottom row). Bars move in different directions from 0 to 360 degrees and at different speeds. Responses are from the task-optimal model in the respective cluster. Bars moving at 75.4°/s in all cardinal directions (green 0°, blue 90°, red 180°, yellow 270°) from -22.5 to 22.5° visual angle.

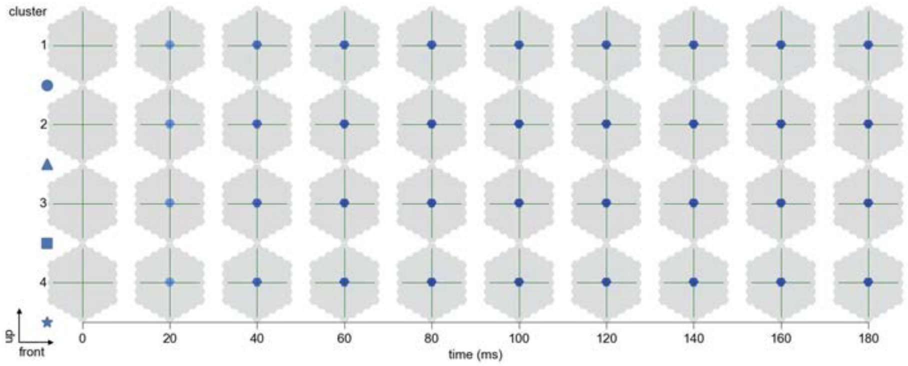

R3 - Figure 12: **Spatio-temporal receptive field.** Responses of the central cell to ON-impulses (5 ms) at single-ommatidium flash locations. The flash occurs at second zero. Responses from the task-optimal model of the respective cluster (rows). Red indicates depolarization, blue indicates hyperpolarization.

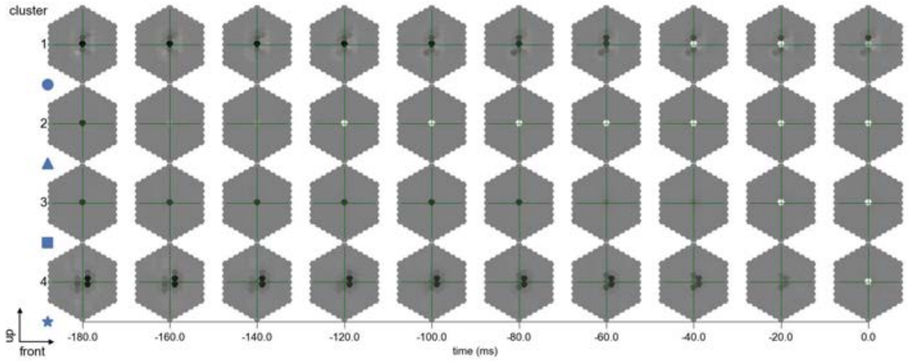

R3 - Figure 13: **Maximally excitatory stimuli.** Each row presents the regularized naturalistic-stimulus from the Sintel dataset that maximizes the cell type's central column response at second zero in the task-optimal model of the respective cluster (rows).

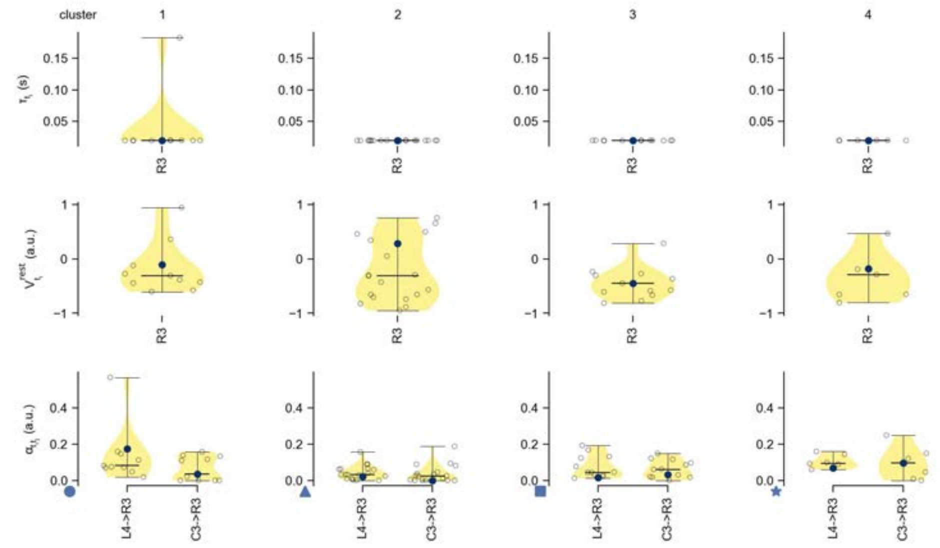

R3 - Figure 14: **Task-constrained parameters.** Each column shows the parameters inferred within the respective cluster. First row: learned time constants of the cell type. Second row: resting potentials of the cell type. Third row: scaling factors for the convolutional filters. The blue scatter represents the parameters from the task-optimal model within the cluster.

4 R4

← Cell types

Figures

1 Anatomical receptive fields. 23
2 Anatomical projective fields. 23
3 Clustering of the responses to naturalistic stimuli. 24
4 Responses to flashes. 24
5 Cluster-average responses to single-ommatidium flashes. 24
6 Peak responses to moving edges. 25
7 Peak responses to moving edges from task-optimal models. 25
8 Responses to moving edges from task-optimal models. 26
9 Peak responses to moving bars. 26
10 Peak responses to moving bars from task-optimal models. 27
11 Responses to moving bars from task-optimal models. 27
12 Spatio-temporal receptive field. 28
13 Maximally excitatory stimuli. 28
14 Task-constrained parameters. 29

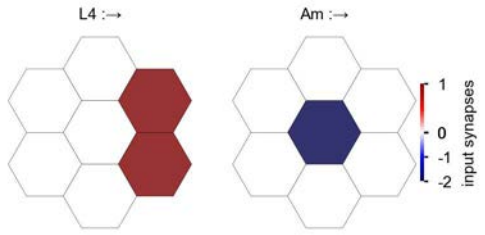

R4 - Figure 1: **Anatomical receptive fields.** Each colored hexagon is an input connection, with the connection strength characterized by the average number of synapses that we count from the EM reconstruction. Red indicates excitatory synapses, blue indicates inhibitory synapses from inferred signs. Filters in the order of their total number of synapses.

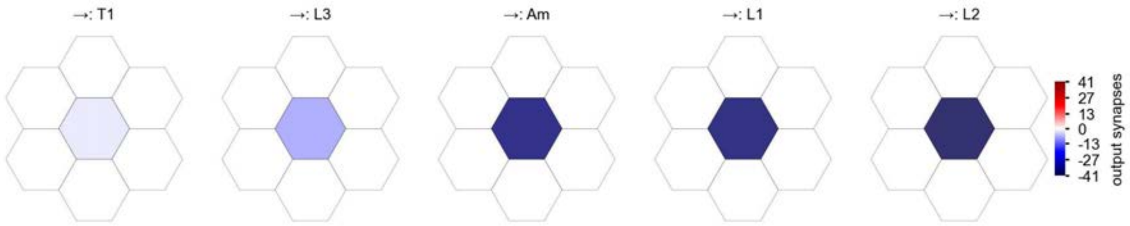

R4 - Figure 2: **Anatomical projective fields.** Each colored hexagon is an output connection, with the connection strength characterized by the average number of synapses that we count from the EM reconstruction. Red indicates excitatory synapses, blue indicates inhibitory synapses from inferred signs. Filters in the order of their total number of synapses.

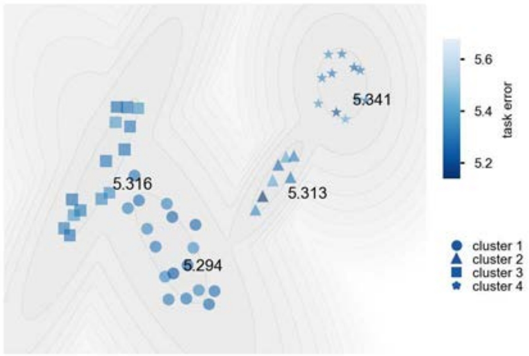

R4 - Figure 3: **Clustering of the responses to naturalistic stimuli.** Clustering of the 50 models based on the cell type responses to naturalistic scenes from the Sintel dataset. Scatterpoints represent individual models colored by their task error.

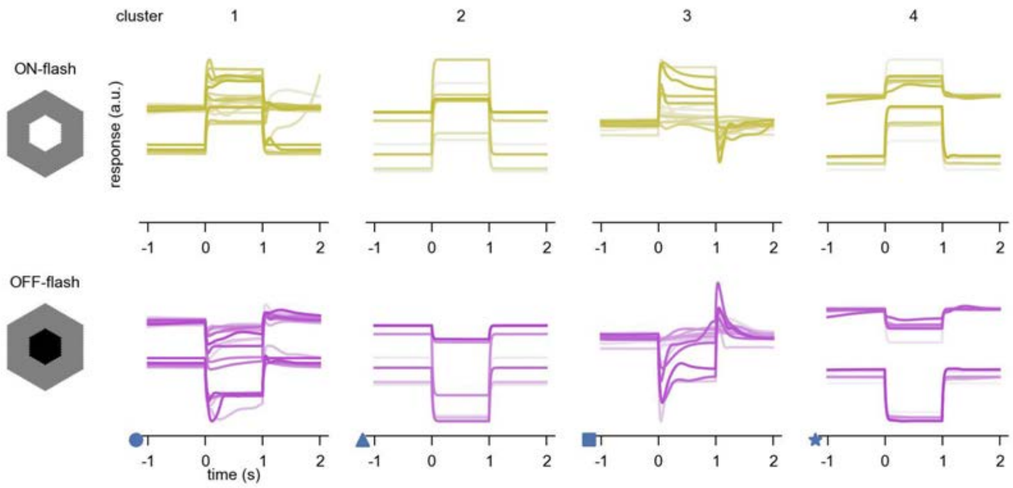

R4 - Figure 4: **Responses to flashes.** The top row shows responses to ON-flashes (yellow), the bottom row shows responses to OFF-flashes (magenta). The responses from the 50 different models that are separated into the different clusters (columns) overlay, with better task-performing models on top. Responses from better task-performing models are more saturated. The circular flashes (1s) cover 6 ommatidia in radius and are presented at time zero. Before and after, a grey-stimulus leads to a stationary state of the network.

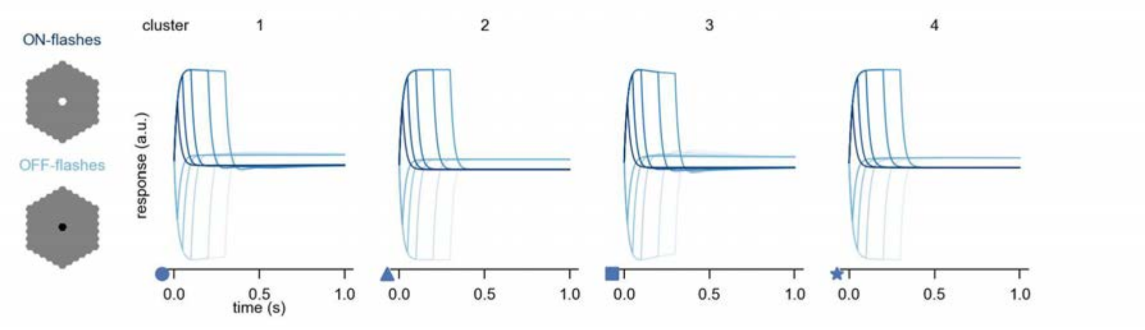

R4 - Figure 5: **Cluster-average responses to single-ommatidium flashes.** Responses to single-ommatidium ON-flashes (dark blue shades) and single-ommatidium OFF-flashes (light blue shades) of 20ms, 50ms, 100ms, 200ms, 300ms duration. The flashes occur at second zero.

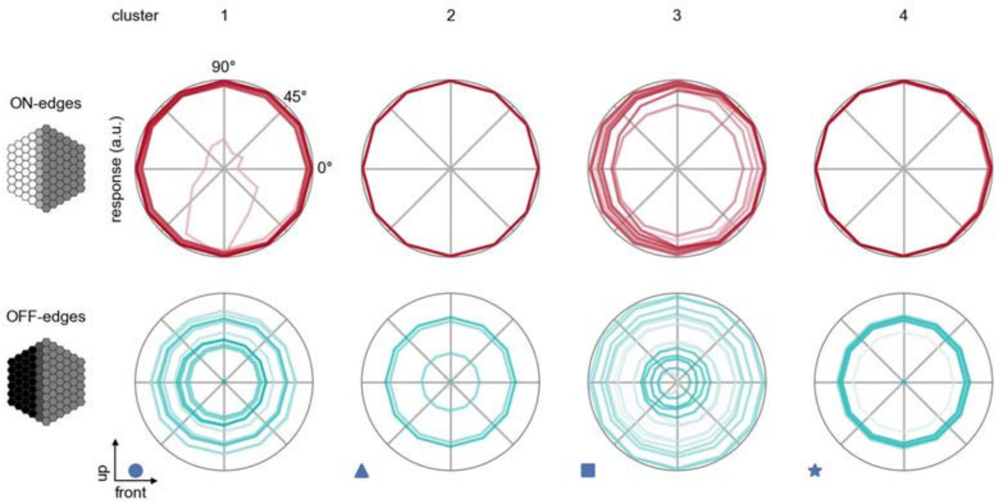

R4 - Figure 6: **Peak responses to moving edges.** The top row shows peak responses to moving ON-edges (red), the bottom row shows peak responses to moving OFF-edges (turqoise). The peak responses are averaged over edge-speeds. Edge-stimuli move in different directions from 0 to 360 degrees. The responses from the different models in the different clusters (columns) overlay. Responses from better task-performing models are more saturated.

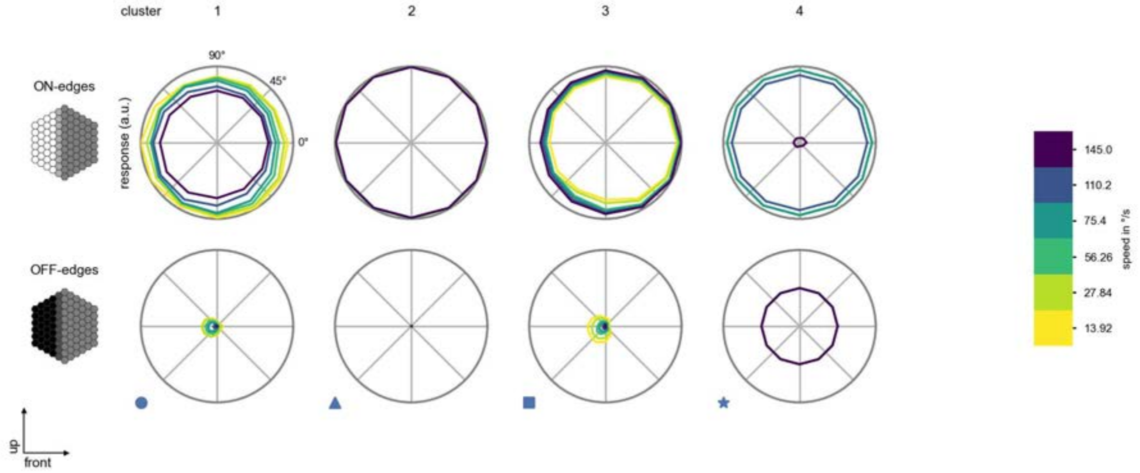

R4 - Figure 7: **Peak responses to moving edges from task-optimal models.** The top row shows peak responses to moving ON-edges, the bottom row shows peak responses to moving OFF-edges of varying speeds from 13.92°/s to 145°/s (yellow to dark blue). The edge-stimuli move in different directions from 0 to 360 degrees and at different speeds. Responses from the task-optimal model in the respective cluster.

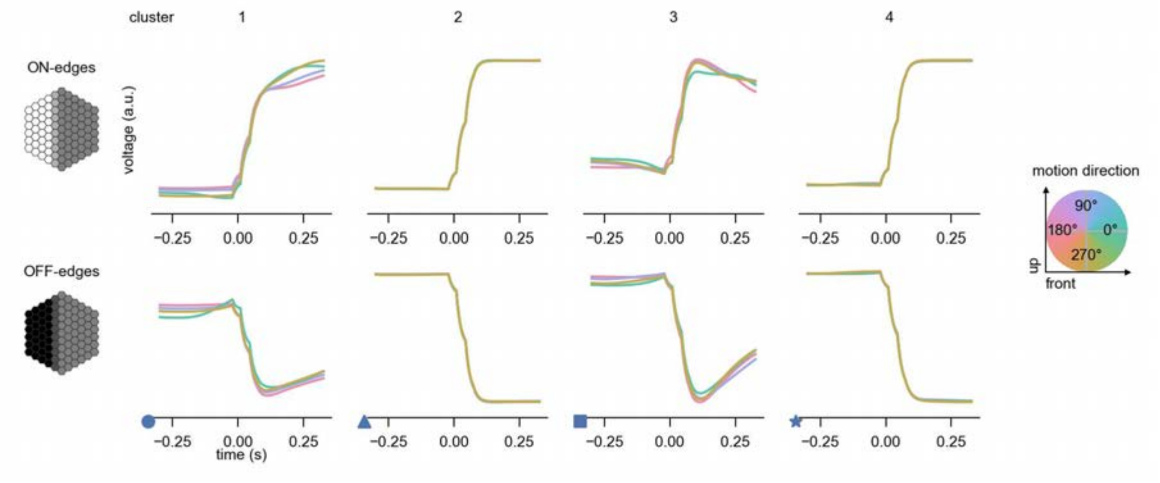

R4 - Figure 8: **Responses to moving edges from task-optimal models.** Responses to moving ON-edges (top row) and to moving OFF-edges (bottom row). Edges move in different directions from 0 to 360 degrees and at different speeds. Responses are from the task-optimal model in the respective cluster. Edges moving at 75.4°/s in all cardinal directions (green 0°, blue 90°, red 180°, yellow 270°) from -22.5 to 22.5° visual angle.

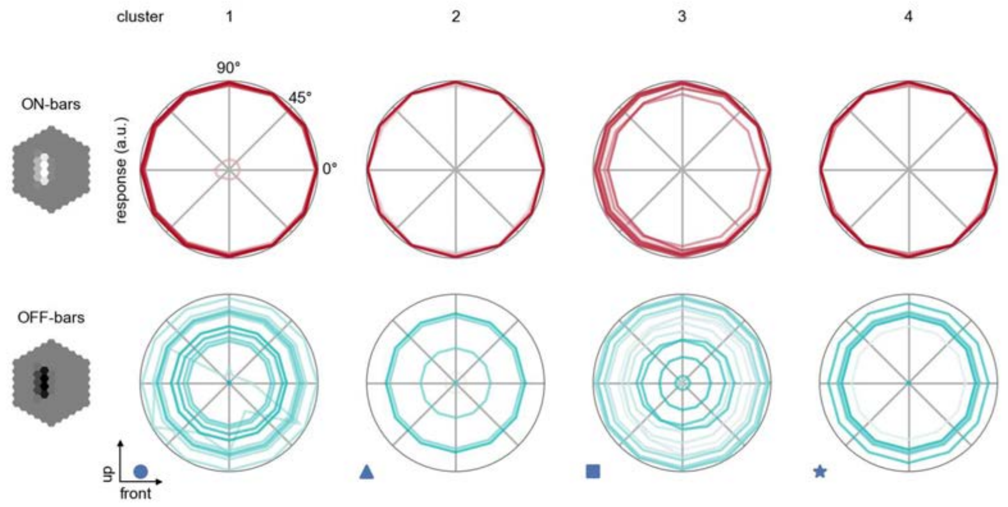

R4 - Figure 9: **Peak responses to moving bars.** The top row shows peak responses to moving ON-bars (red), the bottom row shows peak responses to moving OFF-bars (turqoise). The peak responses are averaged over bar-speeds. Bar-stimuli move in different directions from 0 to 360 degrees. The responses from the different models in the different clusters (columns) overlay. Responses from better task-performing models are more saturated.

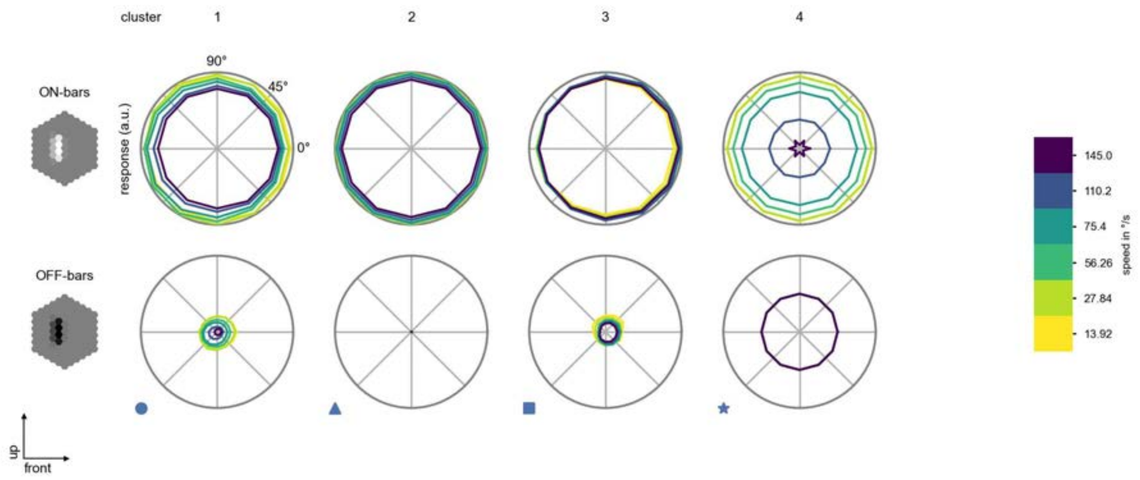

R4 - Figure 10: **Peak responses to moving bars from task-optimal models.** The top row shows peak responses to moving ON-bars, the bottom row shows peak responses to moving OFF-bars of varying speeds from 13.92°/s to 145°/s (yellow to dark blue). The bar-stimuli move in different directions from 0 to 360 degrees and at different speeds. Responses from the task-optimal model in the respective cluster.

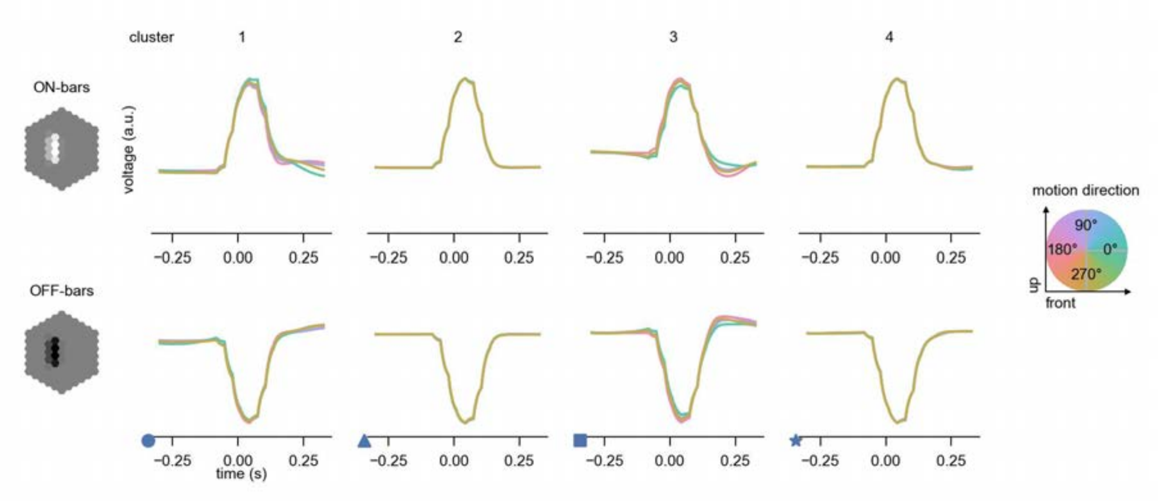

R4 - Figure 11: **Responses to moving bars from task-optimal models.** Responses to moving ON-bars (top row) and to moving OFF-bars (bottom row). Bars move in different directions from 0 to 360 degrees and at different speeds. Responses are from the task-optimal model in the respective cluster. Bars moving at 75.4°/s in all cardinal directions (green 0°, blue 90°, red 180°, yellow 270°) from -22.5 to 22.5° visual angle.

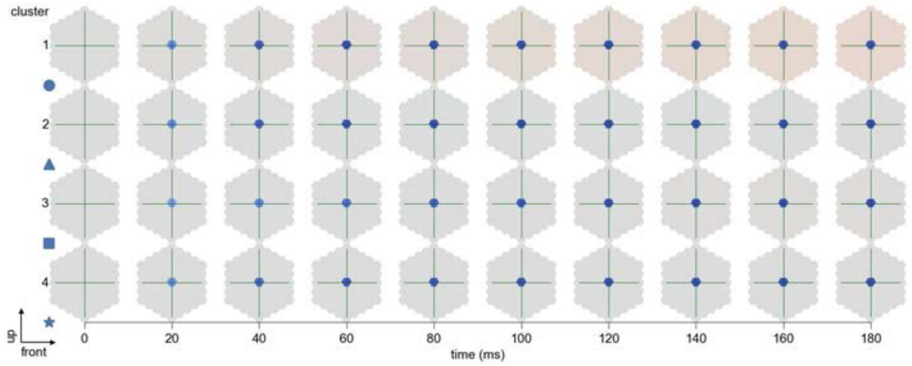

R4 - Figure 12: **Spatio-temporal receptive field.** Responses of the central cell to ON-impulses (5 ms) at single-ommatidium flash locations. The flash occurs at second zero. Responses from the task-optimal model of the respective cluster (rows). Red indicates depolarization, blue indicates hyperpolarization.

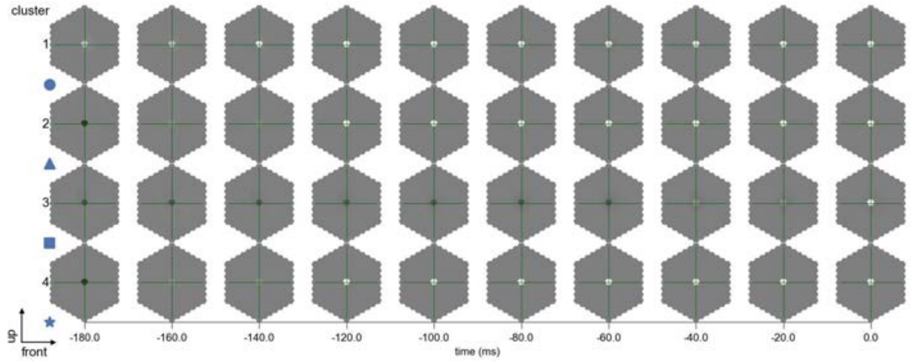

R4 - Figure 13: **Maximally excitatory stimuli.** Each row presents the regularized naturalistic-stimulus from the Sintel dataset that maximizes the cell type's central column response at second zero in the task-optimal model of the respective cluster (rows).

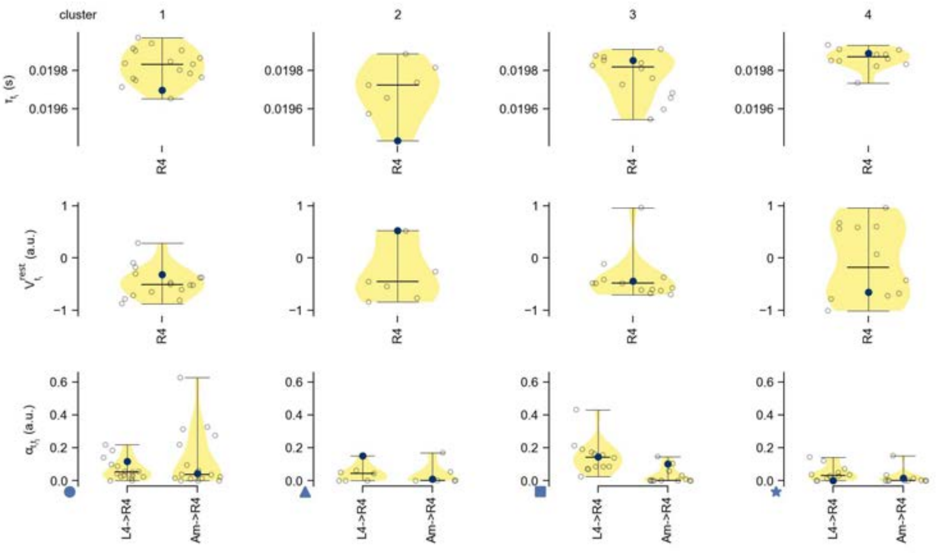

R4 - Figure 14: **Task-constrained parameters.** Each column shows the parameters inferred within the respective cluster. First row: learned time constants of the cell type. Second row: resting potentials of the cell type. Third row: scaling factors for the convolutional filters. The blue scatter represents the parameters from the task-optimal model within the cluster.

5 R5

← Cell types

Figures

1 Anatomical receptive fields. . . . 30
2 Anatomical projective fields. . . . 30
3 Clustering of the responses to naturalistic stimuli. . . . 31
4 Responses to flashes. . . . 31
5 Cluster-average responses to single-ommatidium flashes. . . . 31
6 Peak responses to moving edges. . . . 32
7 Peak responses to moving edges from task-optimal models. . . . 32
8 Responses to moving edges from task-optimal models. . . . 33
9 Peak responses to moving bars. . . . 33
10 Peak responses to moving bars from task-optimal models. . . . 34
11 Responses to moving bars from task-optimal models. . . . 34
12 Spatio-temporal receptive field. . . . 35
13 Maximally excitatory stimuli. . . . 35
14 Task-constrained parameters. . . . 36

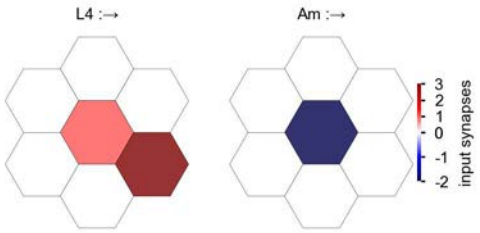

R5 - Figure 1: **Anatomical receptive fields.** Each colored hexagon is an input connection, with the connection strength characterized by the average number of synapses that we count from the EM reconstruction. Red indicates excitatory synapses, blue indicates inhibitory synapses from inferred signs. Filters in the order of their total number of synapses.

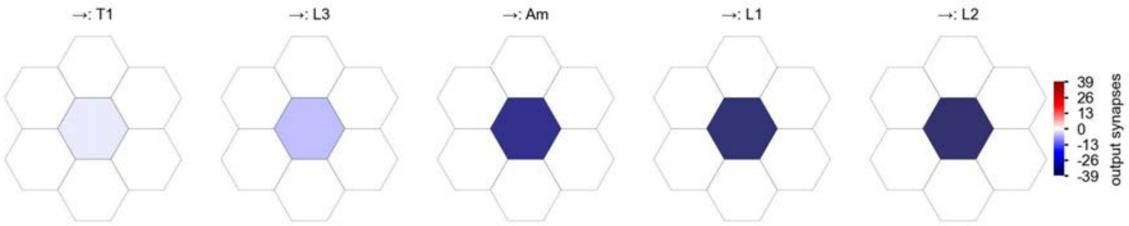

R5 - Figure 2: **Anatomical projective fields.** Each colored hexagon is an output connection, with the connection strength characterized by the average number of synapses that we count from the EM reconstruction. Red indicates excitatory synapses, blue indicates inhibitory synapses from inferred signs. Filters in the order of their total number of synapses.

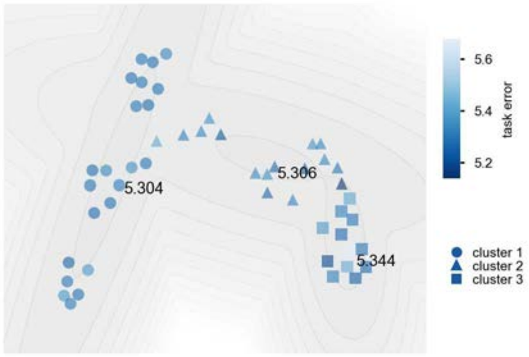

R5 - Figure 3: **Clustering of the responses to naturalistic stimuli.** Clustering of the 50 models based on the cell type responses to naturalistic scenes from the Sintel dataset. Scatterpoints represent individual models colored by their task error.

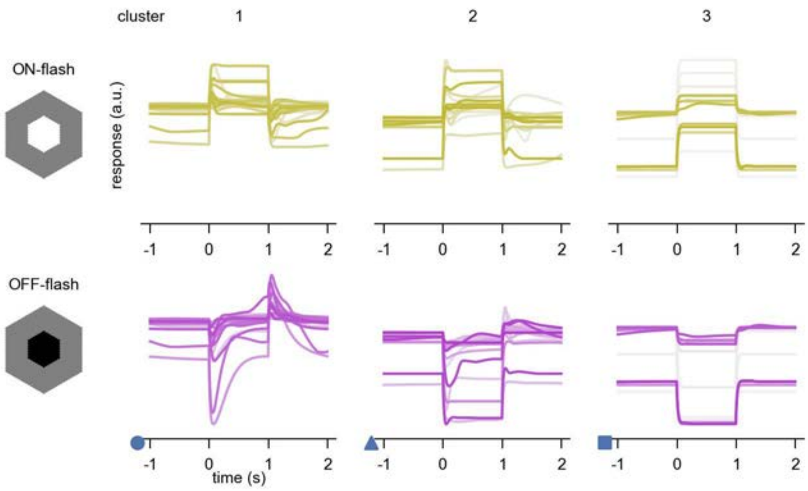

R5 - Figure 4: **Responses to flashes.** The top row shows responses to ON-flashes (yellow), the bottom row shows responses to OFF-flashes (magenta). The responses from the 50 different models that are separated into the different clusters (columns) overlay, with better task-performing models on top. Responses from better task-performing models are more saturated. The circular flashes (1s) cover 6 ommatidia in radius and are presented at time zero. Before and after, a grey-stimulus leads to a stationary state of the network.

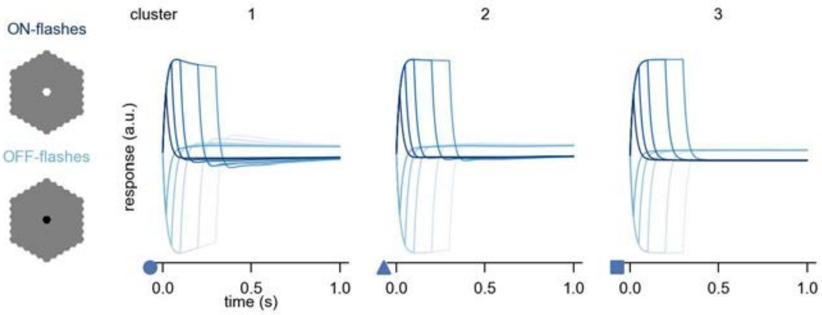

R5 - Figure 5: **Cluster-average responses to single-ommatidium flashes.** Responses to single-ommatidium ON-flashes (dark blue shades) and single-ommatidium OFF-flashes (light blue shades) of 20ms, 50ms, 100ms, 200ms, 300ms duration. The flashes occur at second zero.

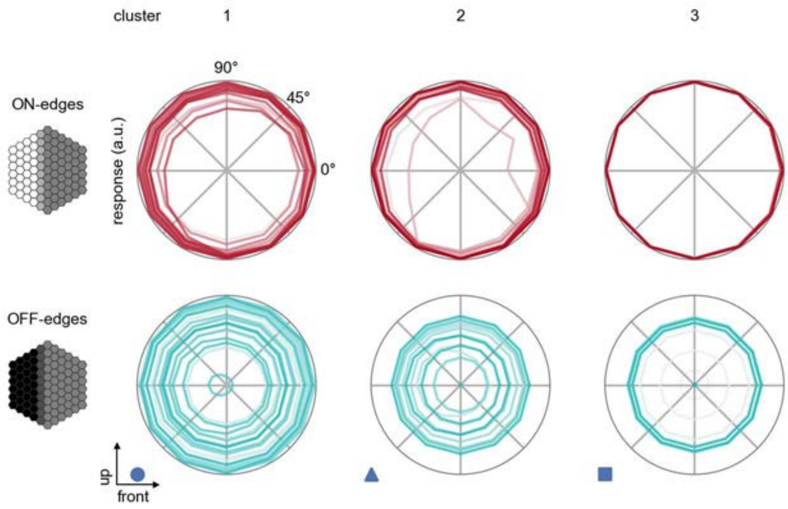

R5 - Figure 6: **Peak responses to moving edges.** The top row shows peak responses to moving ON-edges (red), the bottom row shows peak responses to moving OFF-edges (turqoise). The peak responses are averaged over edge-speeds. Edge-stimuli move in different directions from 0 to 360 degrees. The responses from the different models in the different clusters (columns) overlay. Responses from better task-performing models are more saturated.

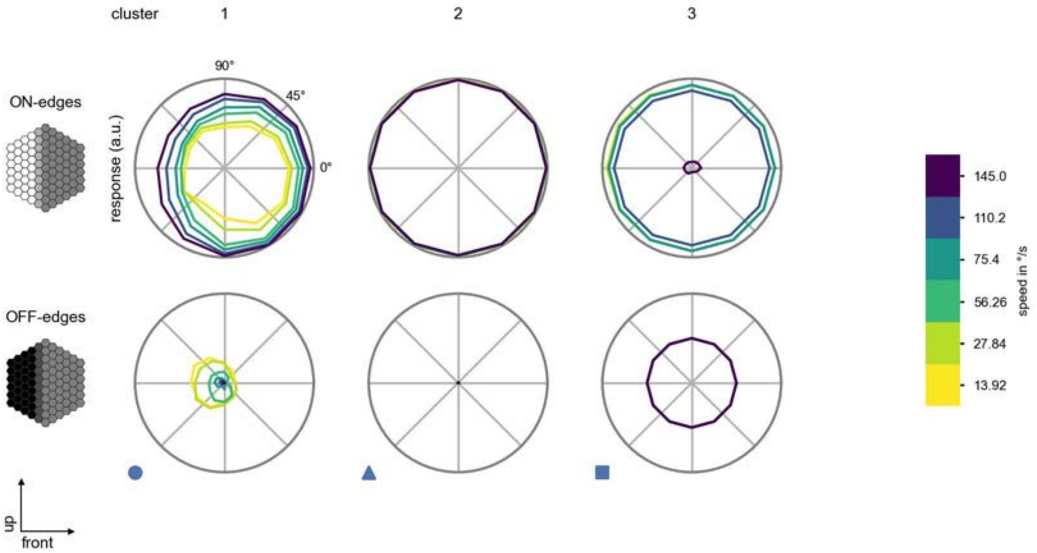

R5 - Figure 7: **Peak responses to moving edges from task-optimal models.** The top row shows peak responses to moving ON-edges, the bottom row shows peak responses to moving OFF-edges of varying speeds from 13.92°/s to 145°/s (yellow to dark blue). The edge-stimuli move in different directions from 0 to 360 degrees and at different speeds. Responses from the task-optimal model in the respective cluster.

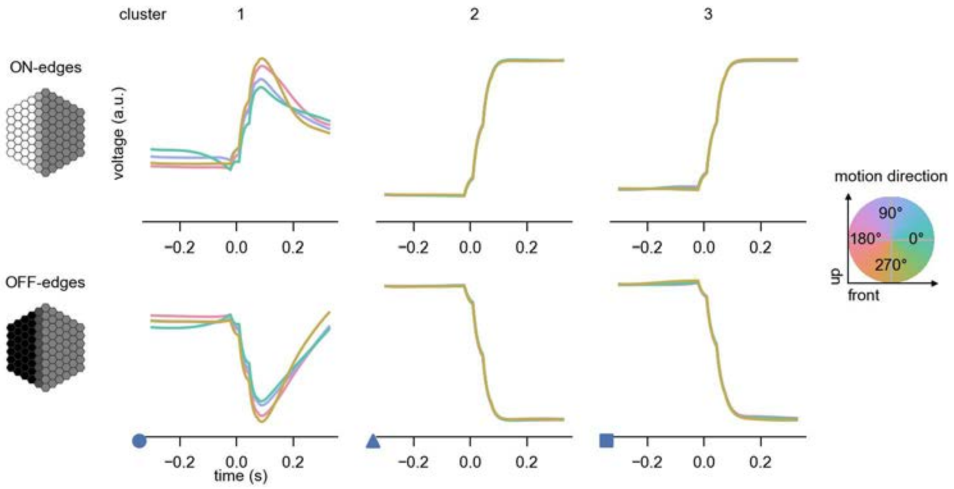

R5 - Figure 8: **Responses to moving edges from task-optimal models.** Responses to moving ON-edges (top row) and to moving OFF-edges (bottom row). Edges move in different directions from 0 to 360 degrees and at different speeds. Responses are from the task-optimal model in the respective cluster. Edges moving at 75.4°/s in all cardinal directions (green 0°, blue 90°, red 180°, yellow 270°) from -22.5 to 22.5° visual angle.

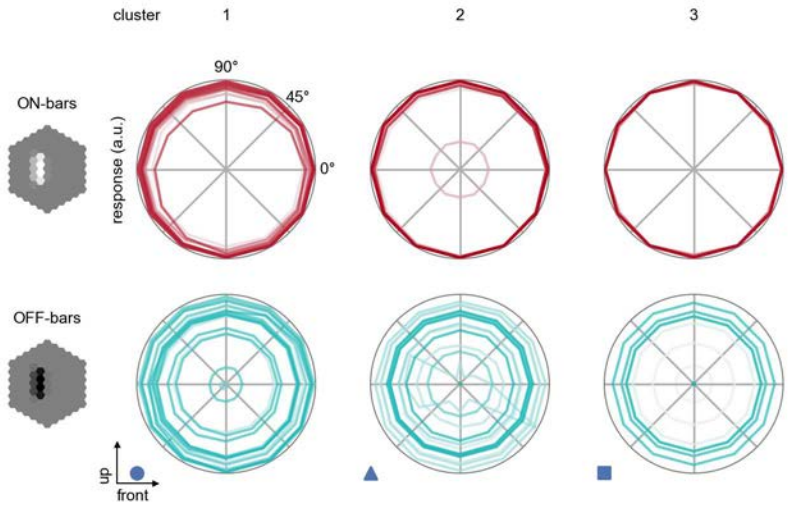

R5 - Figure 9: **Peak responses to moving bars.** The top row shows peak responses to moving ON-bars (red), the bottom row shows peak responses to moving OFF-bars (turqoise). The peak responses are averaged over bar-speeds. Bar-stimuli move in different directions from 0 to 360 degrees. The responses from the different models in the different clusters (columns) overlay. Responses from better task-performing models are more saturated.

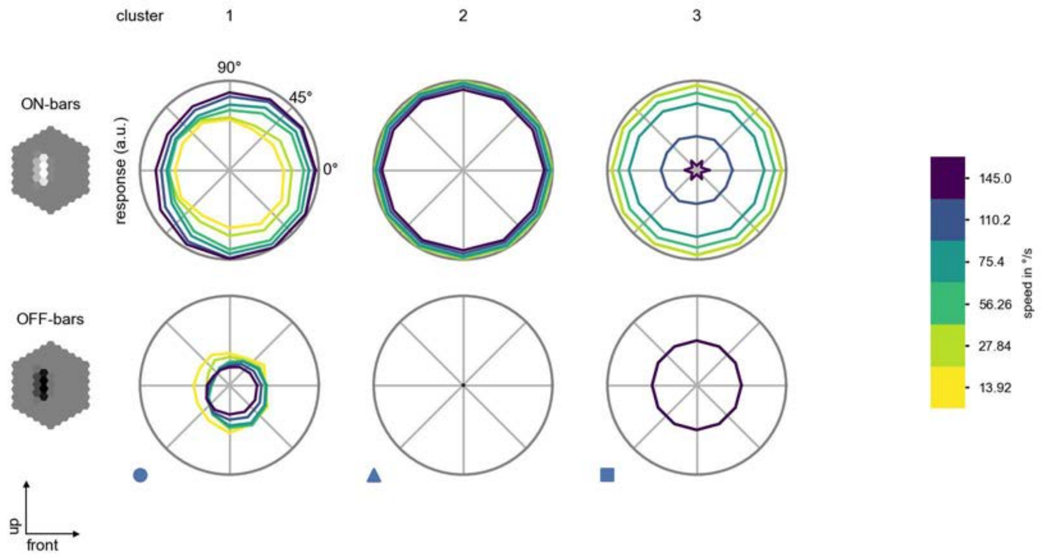

R5 - Figure 10: **Peak responses to moving bars from task-optimal models.** The top row shows peak responses to moving ON-bars, the bottom row shows peak responses to moving OFF-bars of varying speeds from 13.92°/s to 145°/s (yellow to dark blue). The bar-stimuli move in different directions from 0 to 360 degrees and at different speeds. Responses from the task-optimal model in the respective cluster.

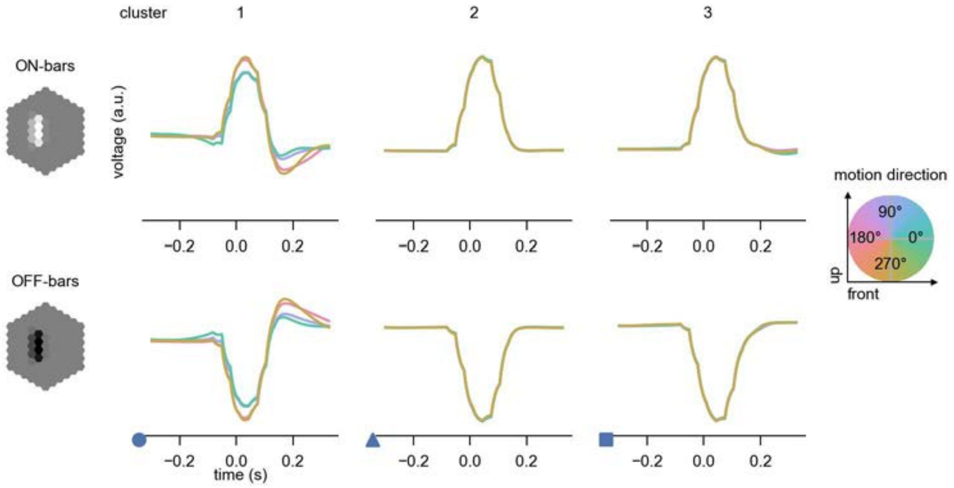

R5 - Figure 11: **Responses to moving bars from task-optimal models.** Responses to moving ON-bars (top row) and to moving OFF-bars (bottom row). Bars move in different directions from 0 to 360 degrees and at different speeds. Responses are from the task-optimal model in the respective cluster. Bars moving at 75.4°/s in all cardinal directions (green 0°, blue 90°, red 180°, yellow 270°) from -22.5 to 22.5° visual angle.

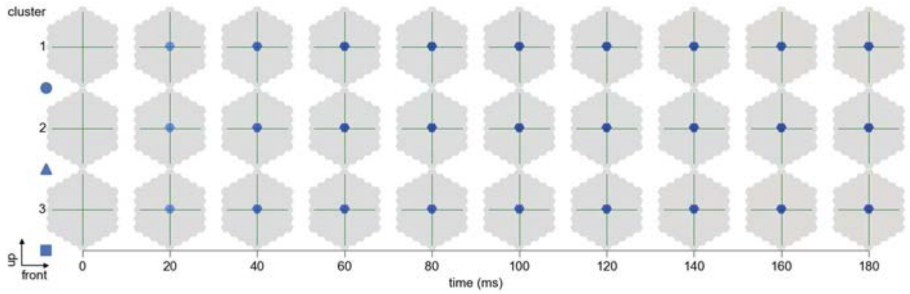

R5 - Figure 12: **Spatio-temporal receptive field.** Responses of the central cell to ON-impulses (5 ms) at single-ommatidium flash locations. The flash occurs at second zero. Responses from the task-optimal model of the respective cluster (rows). Red indicates depolarization, blue indicates hyperpolarization.

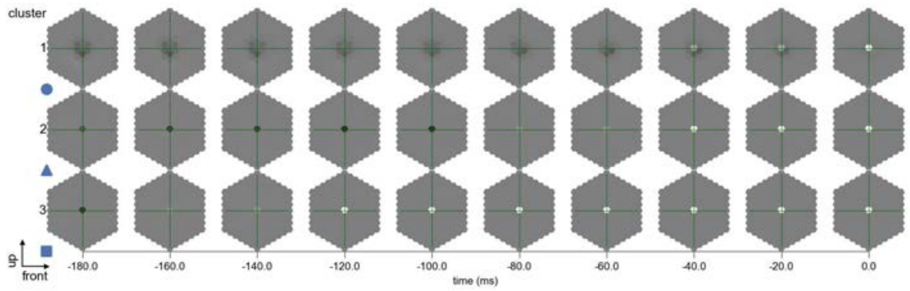

R5 - Figure 13: **Maximally excitatory stimuli.** Each row presents the regularized naturalistic-stimulus from the Sintel dataset that maximizes the cell type's central column response at second zero in the task-optimal model of the respective cluster (rows).

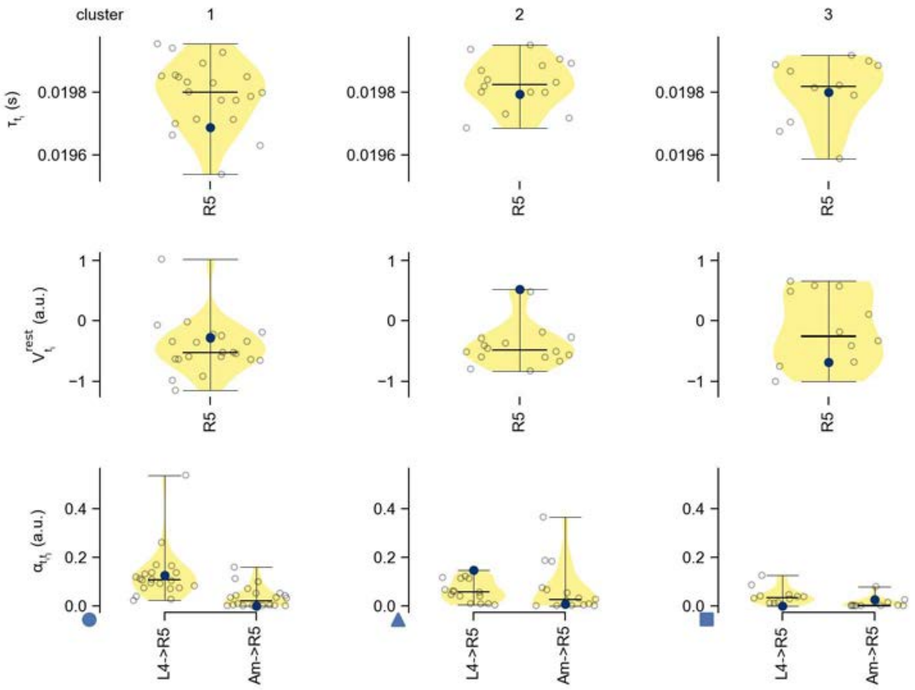

R5 - Figure 14: **Task-constrained parameters.** Each column shows the parameters inferred within the respective cluster. First row: learned time constants of the cell type. Second row: resting potentials of the cell type. Third row: scaling factors for the convolutional filters. The blue scatter represents the parameters from the task-optimal model within the cluster.

6 R6

← Cell types

Figures

1 Anatomical receptive fields. . . . 37
2 Anatomical projective fields. . . . 37
3 Clustering of the responses to naturalistic stimuli. . . . 38
4 Responses to flashes. . . . 38
5 Cluster-average responses to single-ommatidium flashes. . . . 38
6 Peak responses to moving edges. . . . 39
7 Peak responses to moving edges from task-optimal models. . . . 39
8 Responses to moving edges from task-optimal models. . . . 40
9 Peak responses to moving bars. . . . 40
10 Peak responses to moving bars from task-optimal models. . . . 41
11 Responses to moving bars from task-optimal models. . . . 41
12 Spatio-temporal receptive field. . . . 42
13 Maximally excitatory stimuli. . . . 42
14 Task-constrained parameters. . . . 43

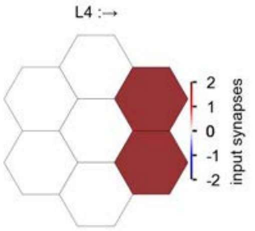

R6 - Figure 1: **Anatomical receptive fields.** Each colored hexagon is an input connection, with the connection strength characterized by the average number of synapses that we count from the EM reconstruction. Red indicates excitatory synapses, blue indicates inhibitory synapses from inferred signs. Filters in the order of their total number of synapses.

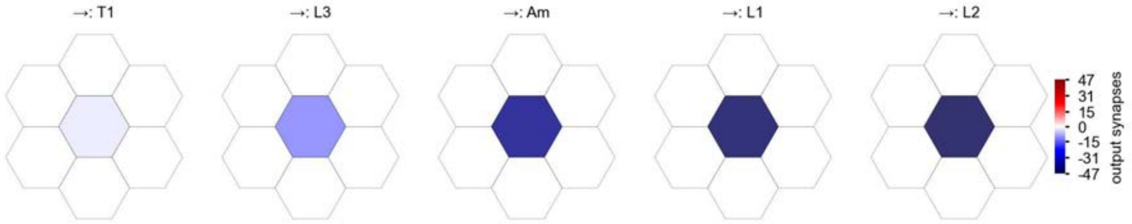

R6 - Figure 2: **Anatomical projective fields.** Each colored hexagon is an output connection, with the connection strength characterized by the average number of synapses that we count from the EM reconstruction. Red indicates excitatory synapses, blue indicates inhibitory synapses from inferred signs. Filters in the order of their total number of synapses.

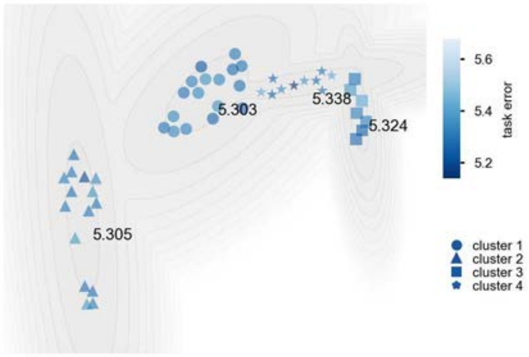

R6 - Figure 3: **Clustering of the responses to naturalistic stimuli.** Clustering of the 50 models based on the cell type responses to naturalistic scenes from the Sintel dataset. Scatterpoints represent individual models colored by their task error.

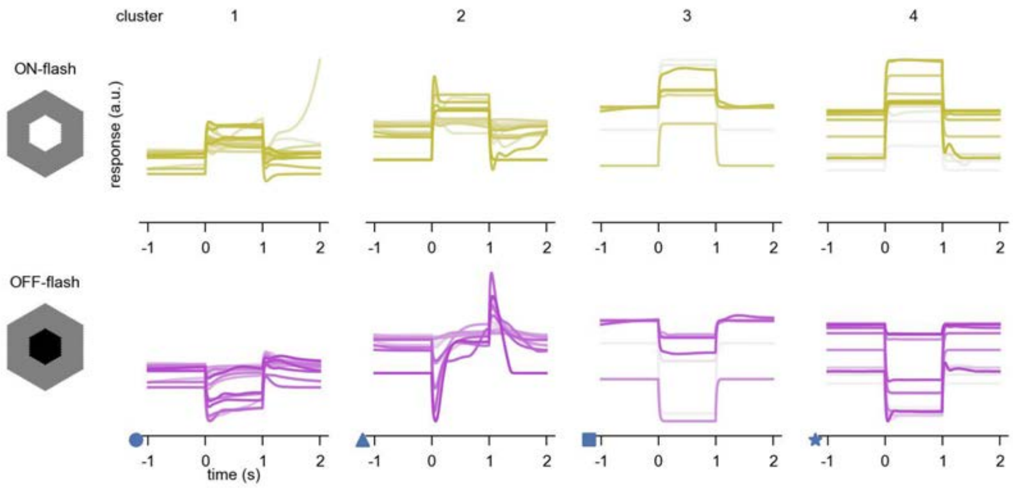

R6 - Figure 4: **Responses to flashes.** The top row shows responses to ON-flashes (yellow), the bottom row shows responses to OFF-flashes (magenta). The responses from the 50 different models that are separated into the different clusters (columns) overlay, with better task-performing models on top. Responses from better task-performing models are more saturated. The circular flashes (1s) cover 6 ommatidia in radius and are presented at time zero. Before and after, a grey-stimulus leads to a stationary state of the network.

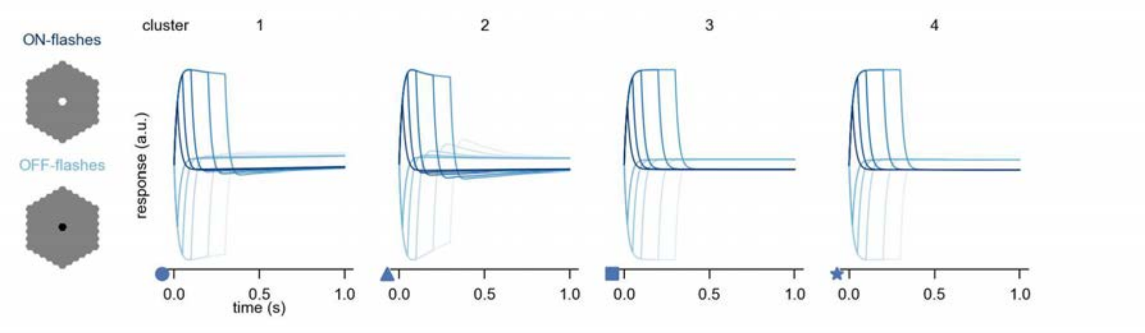

R6 - Figure 5: **Cluster-average responses to single-ommatidium flashes.** Responses to single-ommatidium ON-flashes (dark blue shades) and single-ommatidium OFF-flashes (light blue shades) of 20ms, 50ms, 100ms, 200ms, 300ms duration. The flashes occur at second zero.

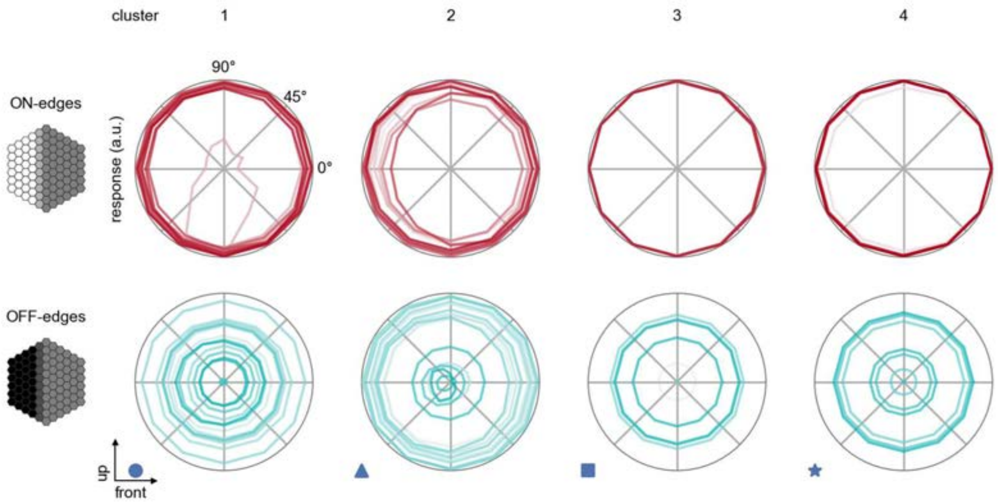

R6 - Figure 6: **Peak responses to moving edges.** The top row shows peak responses to moving ON-edges (red), the bottom row shows peak responses to moving OFF-edges (turqoise). The peak responses are averaged over edge-speeds. Edge-stimuli move in different directions from 0 to 360 degrees. The responses from the different models in the different clusters (columns) overlay. Responses from better task-performing models are more saturated.

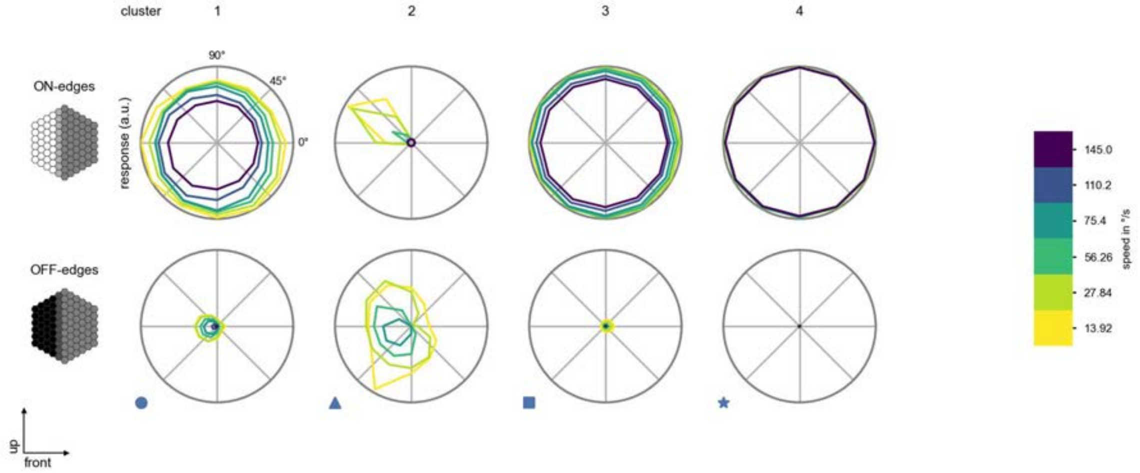

R6 - Figure 7: **Peak responses to moving edges from task-optimal models.** The top row shows peak responses to moving ON-edges, the bottom row shows peak responses to moving OFF-edges of varying speeds from 13.92°/s to 145°/s (yellow to dark blue). The edge-stimuli move in different directions from 0 to 360 degrees and at different speeds. Responses from the task-optimal model in the respective cluster.

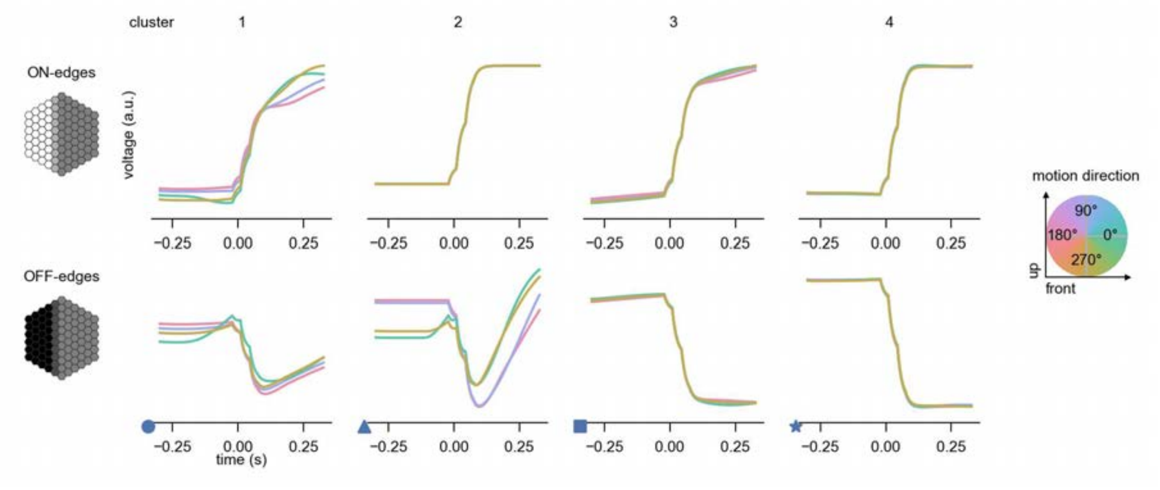

R6 - Figure 8: **Responses to moving edges from task-optimal models.** Responses to moving ON-edges (top row) and to moving OFF-edges (bottom row). Edges move in different directions from 0 to 360 degrees and at different speeds. Responses are from the task-optimal model in the respective cluster. Edges moving at 75.4°/s in all cardinal directions (green 0°, blue 90°, red 180°, yellow 270°) from -22.5 to 22.5° visual angle.

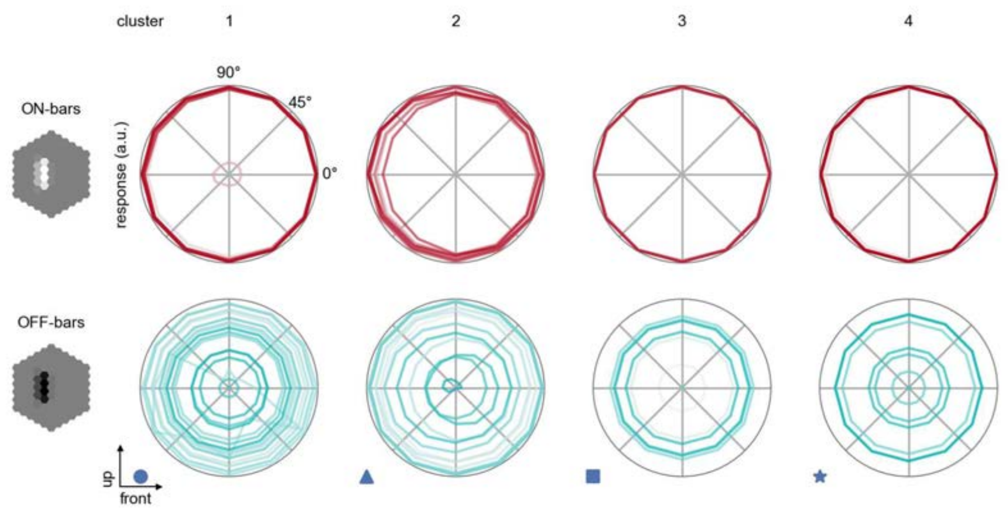

R6 - Figure 9: **Peak responses to moving bars.** The top row shows peak responses to moving ON-bars (red), the bottom row shows peak responses to moving OFF-bars (turqoise). The peak responses are averaged over bar-speeds. Bar-stimuli move in different directions from 0 to 360 degrees. The responses from the different models in the different clusters (columns) overlay. Responses from better task-performing models are more saturated.

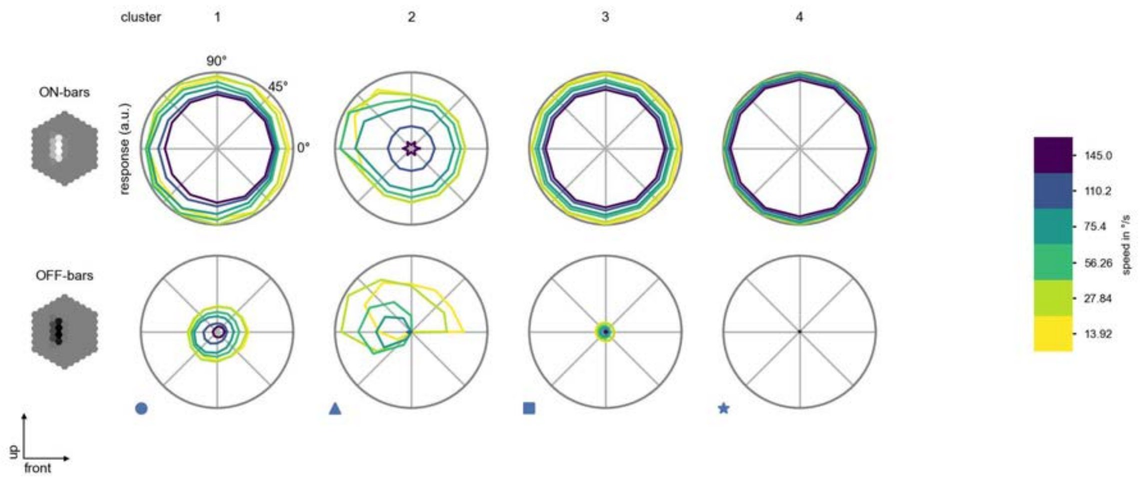

R6 - Figure 10: **Peak responses to moving bars from task-optimal models.** The top row shows peak responses to moving ON-bars, the bottom row shows peak responses to moving OFF-bars of varying speeds from 13.92°/s to 145°/s (yellow to dark blue). The bar-stimuli move in different directions from 0 to 360 degrees and at different speeds. Responses from the task-optimal model in the respective cluster.

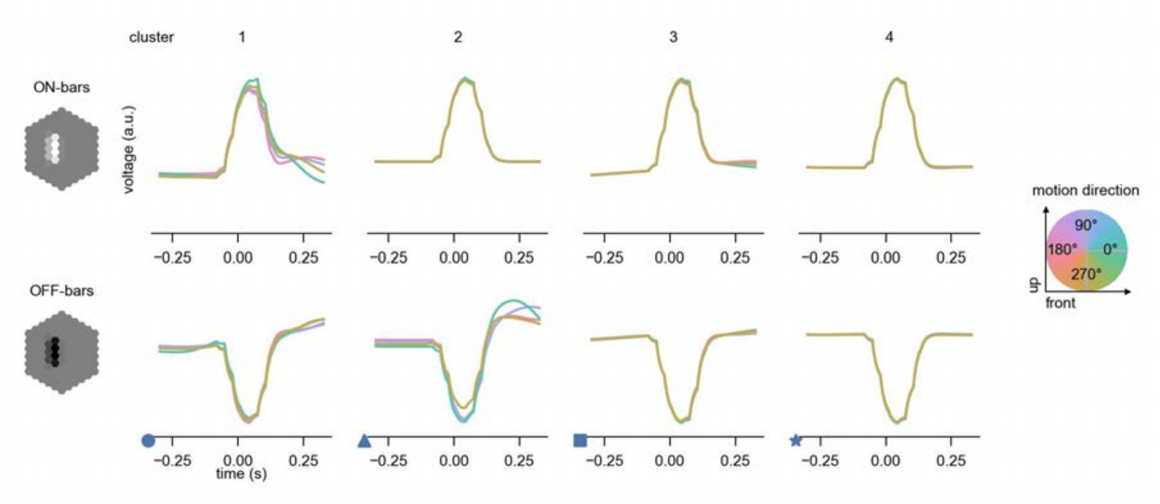

R6 - Figure 11: **Responses to moving bars from task-optimal models.** Responses to moving ON-bars (top row) and to moving OFF-bars (bottom row). Bars move in different directions from 0 to 360 degrees and at different speeds. Responses are from the task-optimal model in the respective cluster. Bars moving at 75.4°/s in all cardinal directions (green 0°, blue 90°, red 180°, yellow 270°) from -22.5 to 22.5° visual angle.

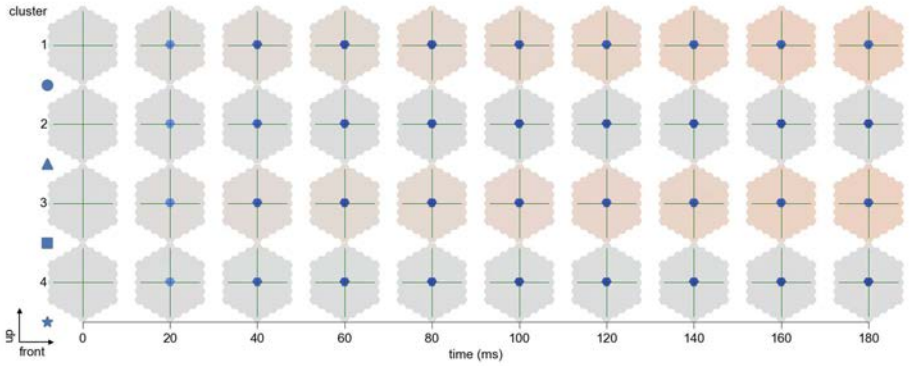

R6 - Figure 12: **Spatio-temporal receptive field.** Responses of the central cell to ON-impulses (5 ms) at single-ommatidium flash locations. The flash occurs at second zero. Responses from the task-optimal model of the respective cluster (rows). Red indicates depolarization, blue indicates hyperpolarization.

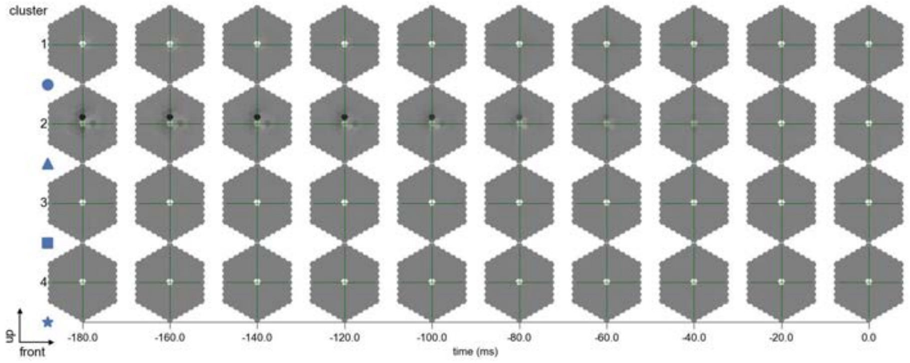

R6 - Figure 13: **Maximally excitatory stimuli.** Each row presents the regularized naturalistic-stimulus from the Sintel dataset that maximizes the cell type's central column response at second zero in the task-optimal model of the respective cluster (rows).

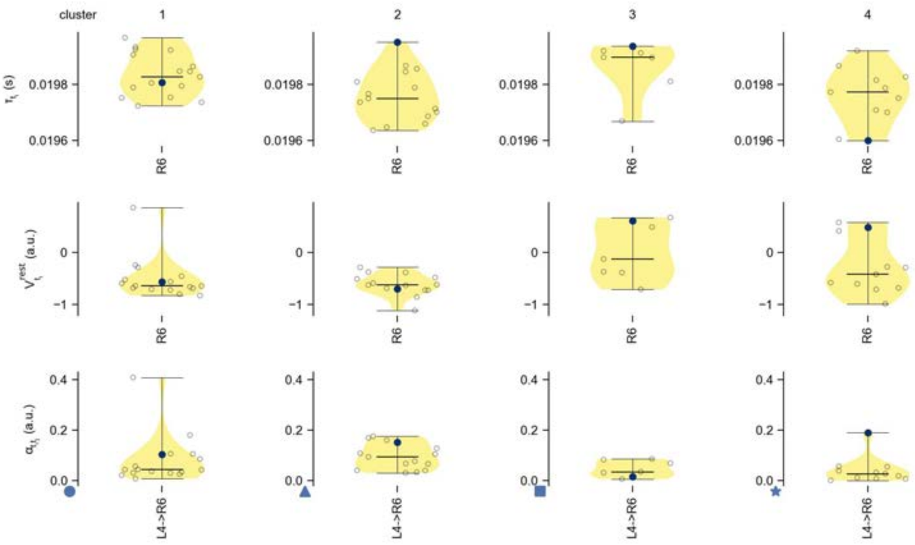

R6 - Figure 14: **Task-constrained parameters.** Each column shows the parameters inferred within the respective cluster. First row: learned time constants of the cell type. Second row: resting potentials of the cell type. Third row: scaling factors for the convolutional filters. The blue scatter represents the parameters from the task-optimal model within the cluster.

7 R7

← Cell types

Figures

1 Anatomical receptive fields. 44
2 Anatomical projective fields. 44
3 Clustering of the responses to naturalistic stimuli. 45
4 Responses to flashes. 45
5 Cluster-average responses to single-ommatidium flashes. 45
6 Peak responses to moving edges. 46
7 Peak responses to moving edges from task-optimal models. 46
8 Responses to moving edges from task-optimal models. 46
9 Peak responses to moving bars. 47
10 Peak responses to moving bars from task-optimal models. 47
11 Responses to moving bars from task-optimal models. 47
12 Spatio-temporal receptive field. 48
13 Maximally excitatory stimuli. 48
14 Task-constrained parameters. 49

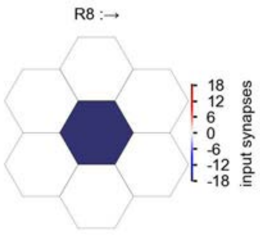

R7 - Figure 1: **Anatomical receptive fields.** Each colored hexagon is an input connection, with the connection strength characterized by the average number of synapses that we count from the EM reconstruction. Red indicates excitatory synapses, blue indicates inhibitory synapses from inferred signs. Filters in the order of their total number of synapses.

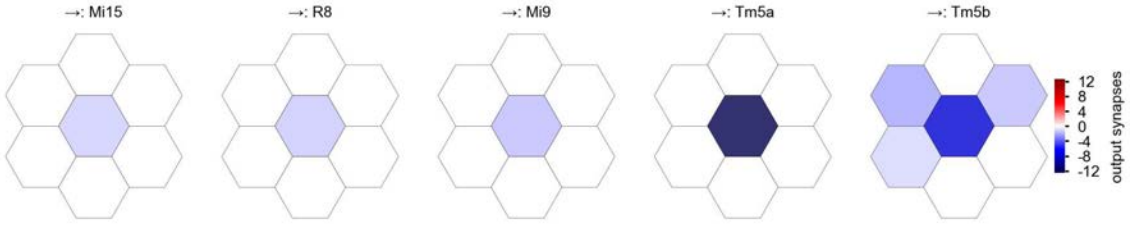

R7 - Figure 2: **Anatomical projective fields.** Each colored hexagon is an output connection, with the connection strength characterized by the average number of synapses that we count from the EM reconstruction. Red indicates excitatory synapses, blue indicates inhibitory synapses from inferred signs. Filters in the order of their total number of synapses.

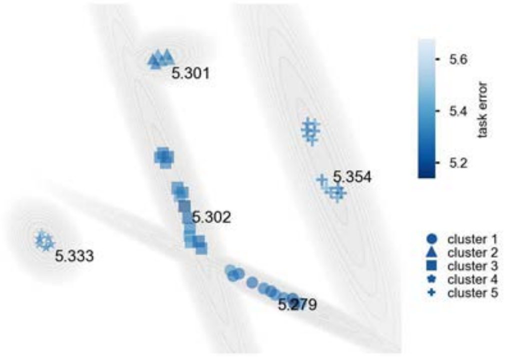

R7 - Figure 3: **Clustering of the responses to naturalistic stimuli.** Clustering of the 50 models based on the cell type responses to naturalistic scenes from the Sintel dataset. Scatterpoints represent individual models colored by their task error.

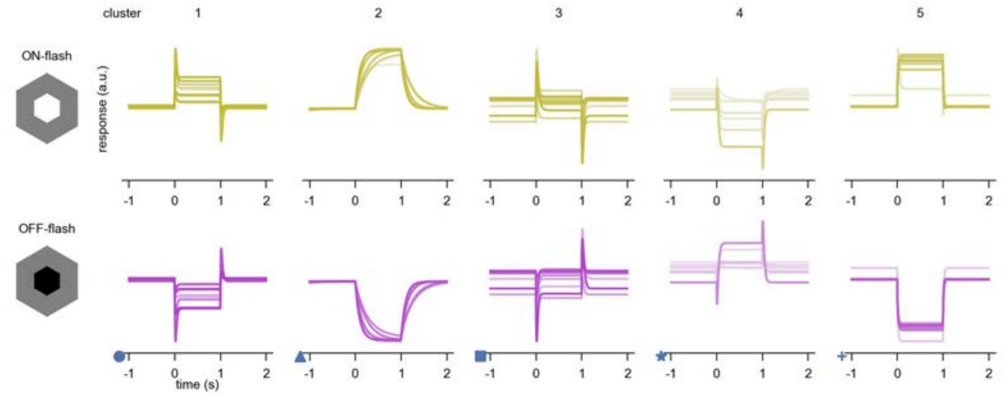

R7 - Figure 4: **Responses to flashes.** The top row shows responses to ON-flashes (yellow), the bottom row shows responses to OFF-flashes (magenta). The responses from the 50 different models that are separated into the different clusters (columns) overlay, with better task-performing models on top. Responses from better task-performing models are more saturated. The circular flashes (1s) cover 6 ommatidia in radius and are presented at time zero. Before and after, a grey-stimulus leads to a stationary state of the network.

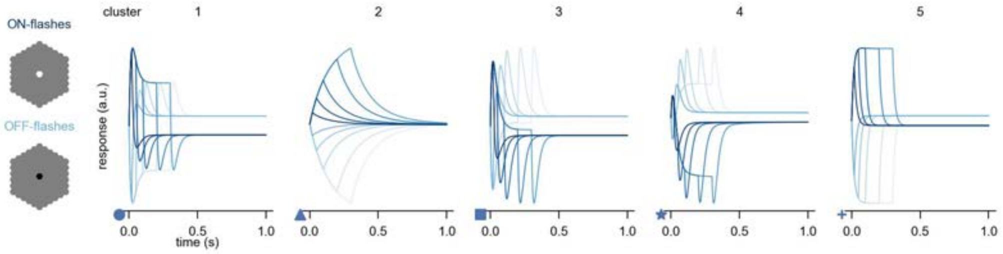

R7 - Figure 5: **Cluster-average responses to single-ommatidium flashes.** Responses to single-ommatidium ON-flashes (dark blue shades) and single-ommatidium OFF-flashes (light blue shades) of 20ms, 50ms, 100ms, 200ms, 300ms duration. The flashes occur at second zero.

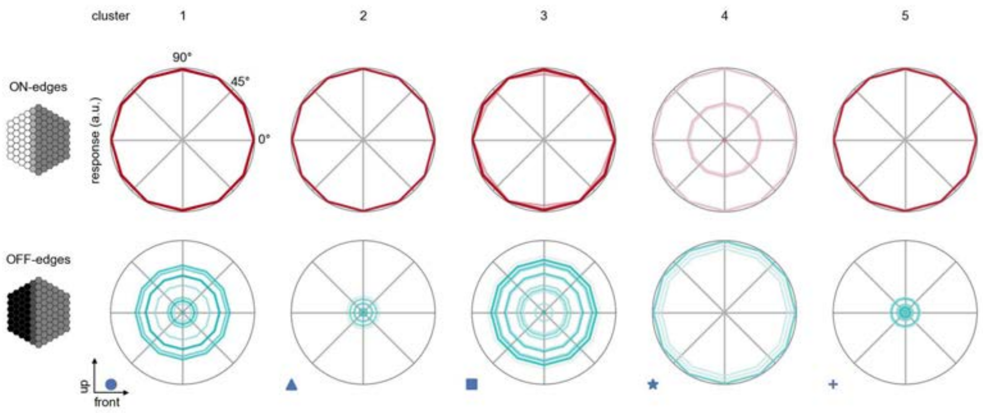

R7 - Figure 6: **Peak responses to moving edges.** The top row shows peak responses to moving ON-edges (red), the bottom row shows peak responses to moving OFF-edges (turqoise). The peak responses are averaged over edge-speeds. Edge-stimuli move in different directions from 0 to 360 degrees. The responses from the different models in the different clusters (columns) overlay. Responses from better task-performing models are more saturated.

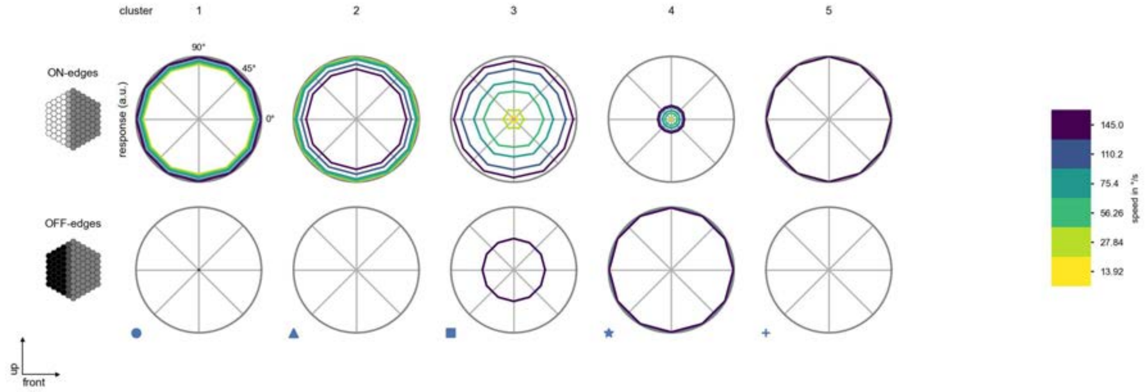

R7 - Figure 7: **Peak responses to moving edges from task-optimal models.** The top row shows peak responses to moving ON-edges, the bottom row shows peak responses to moving OFF-edges of varying speeds from 13.92°/s to 145°/s (yellow to dark blue). The edge-stimuli move in different directions from 0 to 360 degrees and at different speeds. Responses from the task-optimal model in the respective cluster.

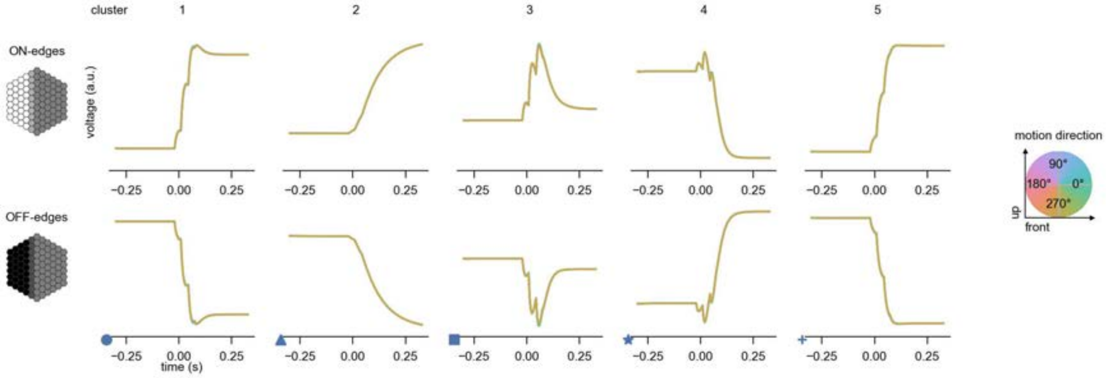

R7 - Figure 8: **Responses to moving edges from task-optimal models.** Responses to moving ON-edges (top row) and to moving OFF-edges (bottom row). Edges move in different directions from 0 to 360 degrees and at different speeds. Responses are from the task-optimal model in the respective cluster. Edges moving at 75.4°/s in all cardinal directions (green 0°, blue 90°, red 180°, yellow 270°) from -22.5 to 22.5° visual angle.

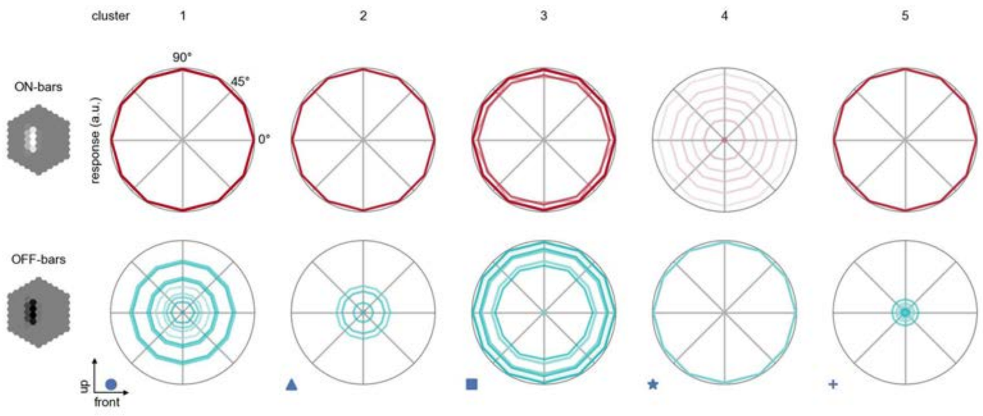

R7 - Figure 9: **Peak responses to moving bars.** The top row shows peak responses to moving ON-bars (red), the bottom row shows peak responses to moving OFF-bars (turqoise). The peak responses are averaged over bar-speeds. Bar-stimuli move in different directions from 0 to 360 degrees. The responses from the different models in the different clusters (columns) overlay. Responses from better task-performing models are more saturated.

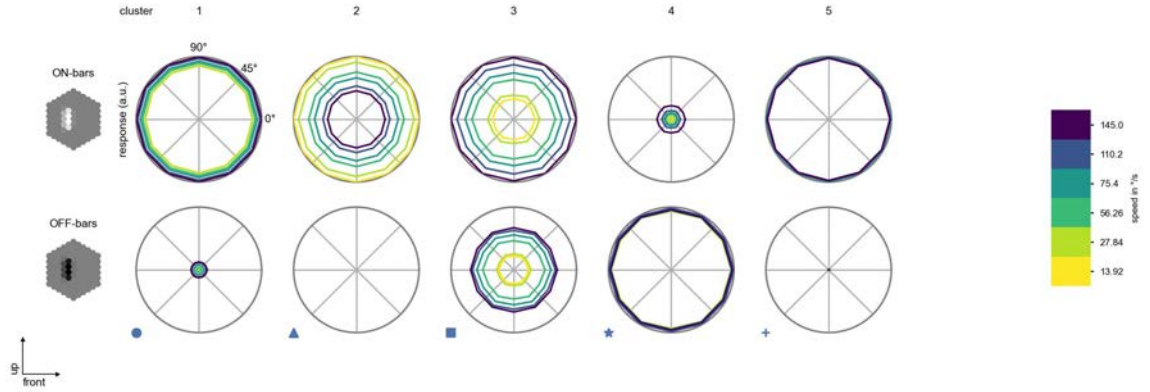

R7 - Figure 10: **Peak responses to moving bars from task-optimal models.** The top row shows peak responses to moving ON-bars, the bottom row shows peak responses to moving OFF-bars of varying speeds from 13.92°/s to 145°/s (yellow to dark blue). The bar-stimuli move in different directions from 0 to 360 degrees and at different speeds. Responses from the task-optimal model in the respective cluster.

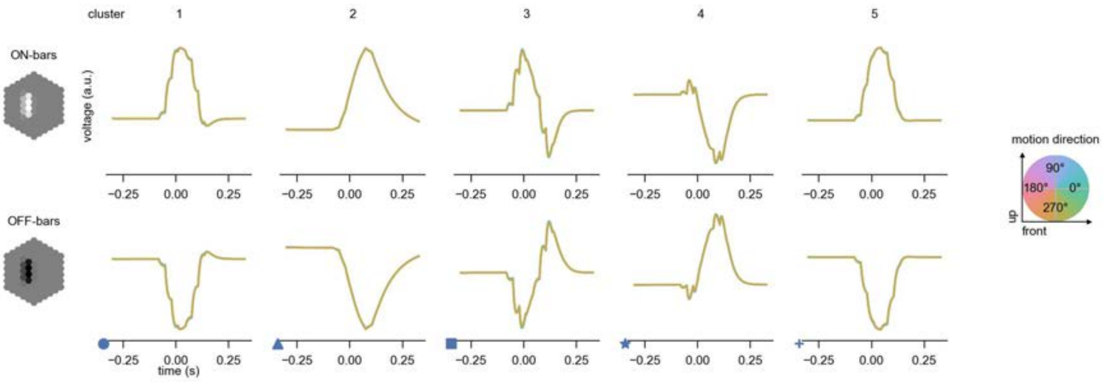

R7 - Figure 11: **Responses to moving bars from task-optimal models.** Responses to moving ON-bars (top row) and to moving OFF-bars (bottom row). Bars move in different directions from 0 to 360 degrees and at different speeds. Responses are from the task-optimal model in the respective cluster. Bars moving at 75.4°/s in all cardinal directions (green 0°, blue 90°, red 180°, yellow 270°) from -22.5 to 22.5° visual angle.

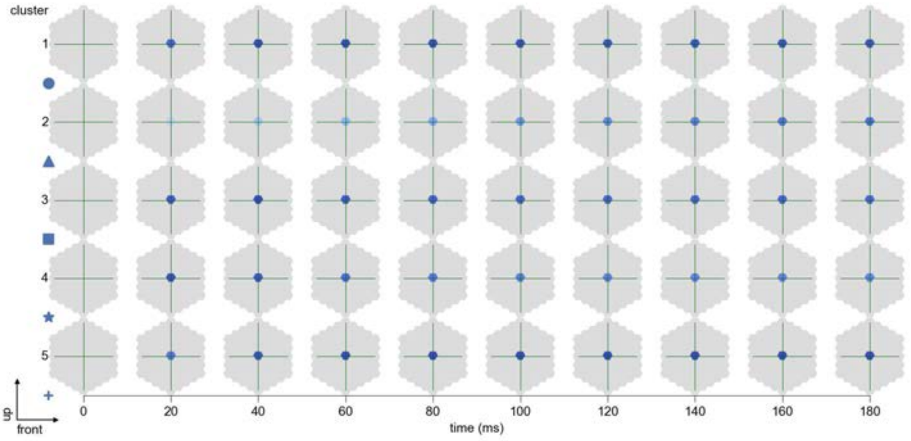

R7 - Figure 12: **Spatio-temporal receptive field.** Responses of the central cell to ON-impulses (5 ms) at single-ommatidium flash locations. The flash occurs at second zero. Responses from the task-optimal model of the respective cluster (rows). Red indicates depolarization, blue indicates hyperpolarization.

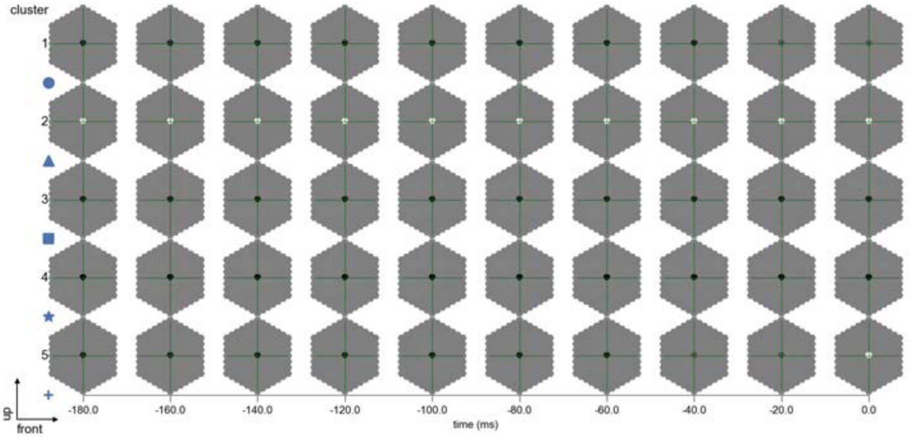

R7 - Figure 13: **Maximally excitatory stimuli.** Each row presents the regularized naturalistic-stimulus from the Sintel dataset that maximizes the cell type's central column response at second zero in the task-optimal model of the respective cluster (rows).

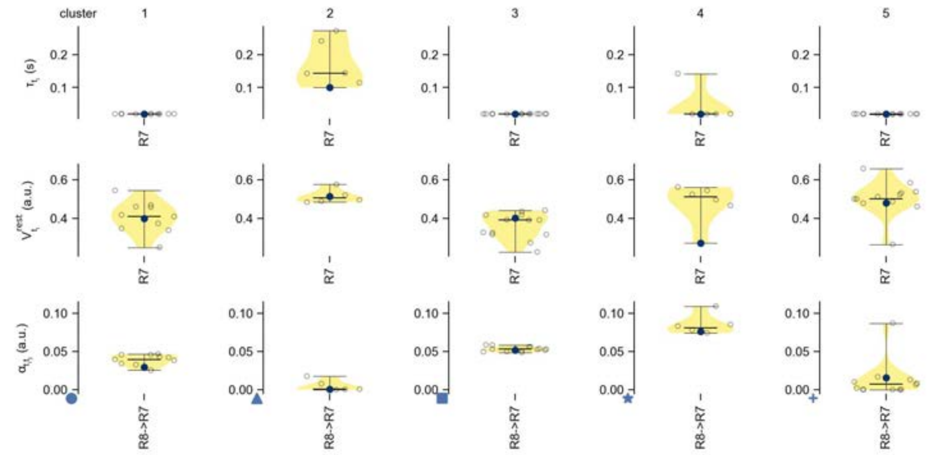

R7 - Figure 14: **Task-constrained parameters.** Each column shows the parameters inferred within the respective cluster. First row: learned time constants of the cell type. Second row: resting potentials of the cell type. Third row: scaling factors for the convolutional filters. The blue scatter represents the parameters from the task-optimal model within the cluster.

8 R8

← Cell types

Figures

1 Anatomical receptive fields. 50
2 Anatomical projective fields. 50
3 Clustering of the responses to naturalistic stimuli. 51
4 Responses to flashes. 51
5 Cluster-average responses to single-ommatidium flashes. 51
6 Peak responses to moving edges. 52
7 Peak responses to moving edges from task-optimal models. 52
8 Responses to moving edges from task-optimal models. 52
9 Peak responses to moving bars. 53
10 Peak responses to moving bars from task-optimal models. 53
11 Responses to moving bars from task-optimal models. 53
12 Spatio-temporal receptive field. 54
13 Maximally excitatory stimuli. 54
14 Task-constrained parameters. 55

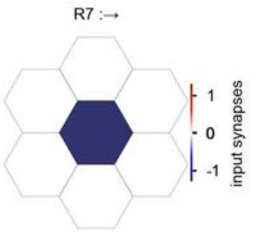

R8 - Figure 1: **Anatomical receptive fields.** Each colored hexagon is an input connection, with the connection strength characterized by the average number of synapses that we count from the EM reconstruction. Red indicates excitatory synapses, blue indicates inhibitory synapses from inferred signs. Filters in the order of their total number of synapses.

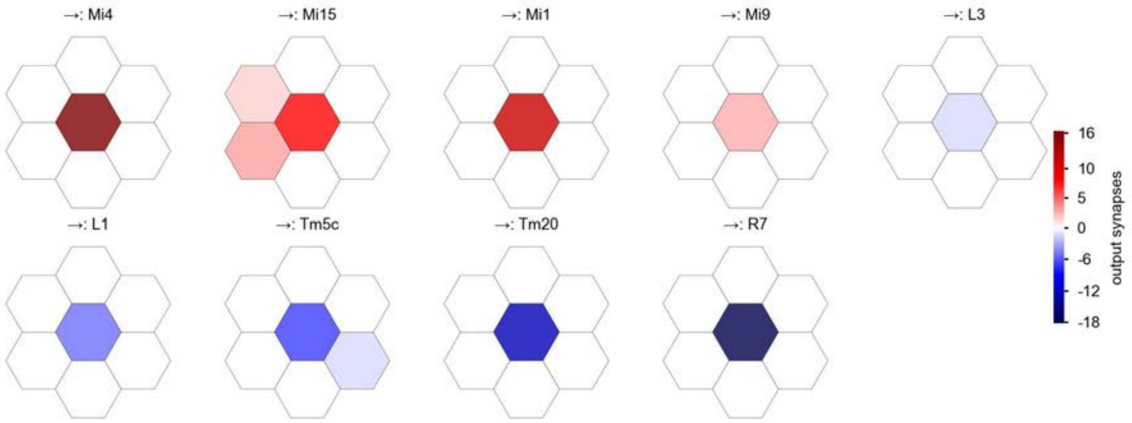

R8 - Figure 2: **Anatomical projective fields.** Each colored hexagon is an output connection, with the connection strength characterized by the average number of synapses that we count from the EM reconstruction. Red indicates excitatory synapses, blue indicates inhibitory synapses from inferred signs. Filters in the order of their total number of synapses.

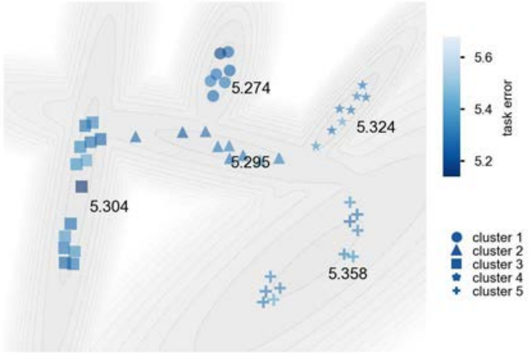

R8 - Figure 3: **Clustering of the responses to naturalistic stimuli.** Clustering of the 50 models based on the cell type responses to naturalistic scenes from the Sintel dataset. Scatterpoints represent individual models colored by their task error.

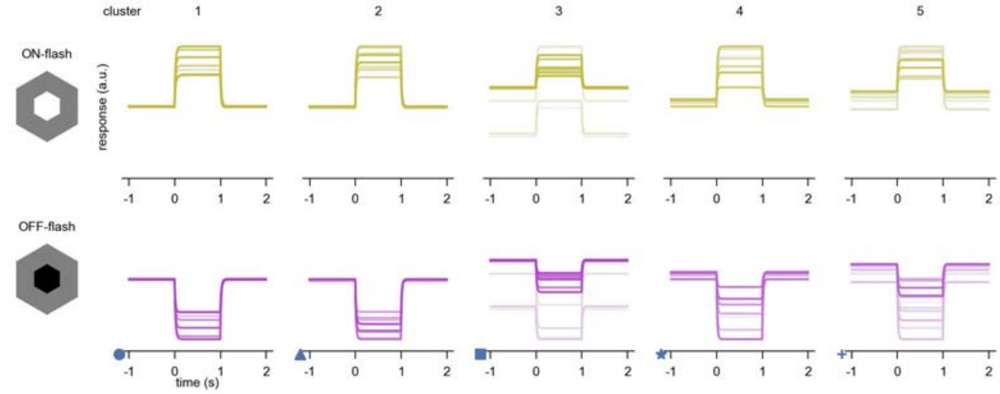

R8 - Figure 4: **Responses to flashes.** The top row shows responses to ON-flashes (yellow), the bottom row shows responses to OFF-flashes (magenta). The responses from the 50 different models that are separated into the different clusters (columns) overlay, with better task-performing models on top. Responses from better task-performing models are more saturated. The circular flashes (1s) cover 6 ommatidia in radius and are presented at time zero. Before and after, a grey-stimulus leads to a stationary state of the network.

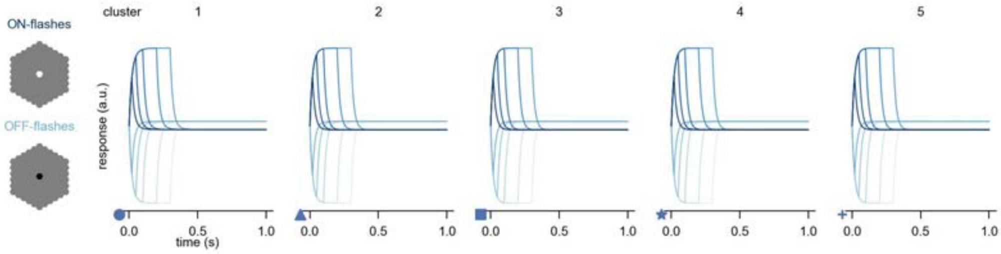

R8 - Figure 5: **Cluster-average responses to single-ommatidium flashes.** Responses to single-ommatidium ON-flashes (dark blue shades) and single-ommatidium OFF-flashes (light blue shades) of 20ms, 50ms, 100ms, 200ms, 300ms duration. The flashes occur at second zero.

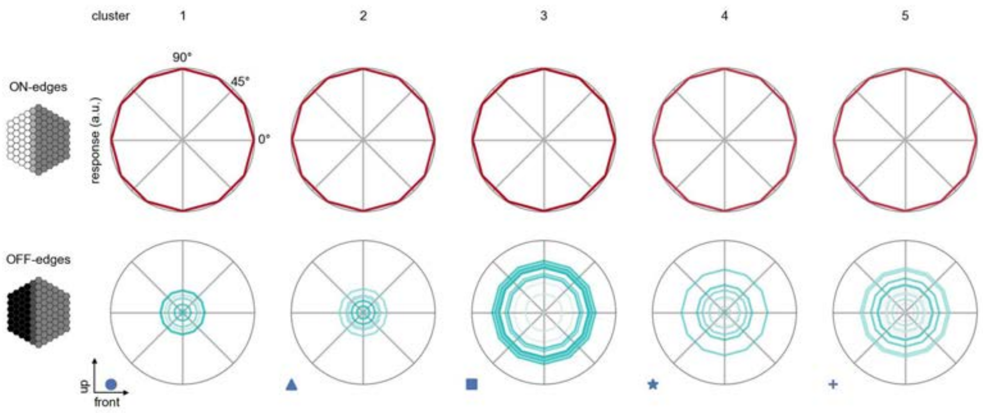

R8 - Figure 6: **Peak responses to moving edges.** The top row shows peak responses to moving ON-edges (red), the bottom row shows peak responses to moving OFF-edges (turqoise). The peak responses are averaged over edge-speeds. Edge-stimuli move in different directions from 0 to 360 degrees. The responses from the different models in the different clusters (columns) overlay. Responses from better task-performing models are more saturated.

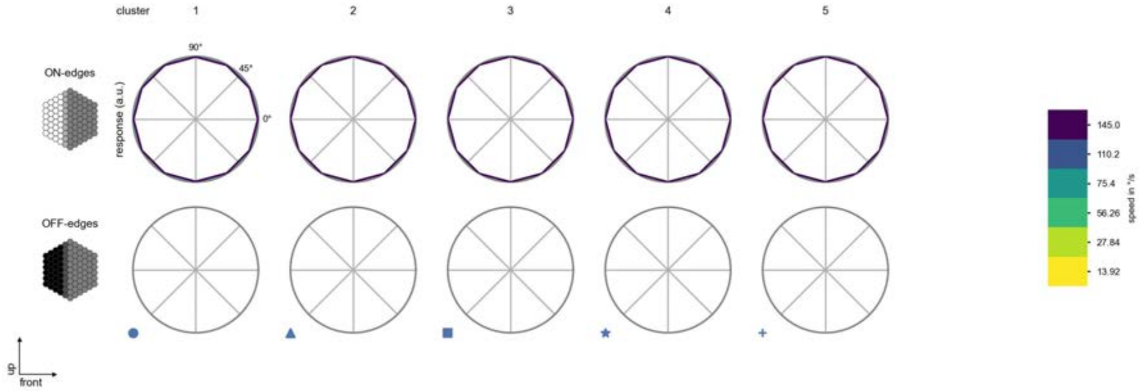

R8 - Figure 7: **Peak responses to moving edges from task-optimal models.** The top row shows peak responses to moving ON-edges, the bottom row shows peak responses to moving OFF-edges of varying speeds from 13.92°/s to 145°/s (yellow to dark blue). The edge-stimuli move in different directions from 0 to 360 degrees and at different speeds. Responses from the task-optimal model in the respective cluster.

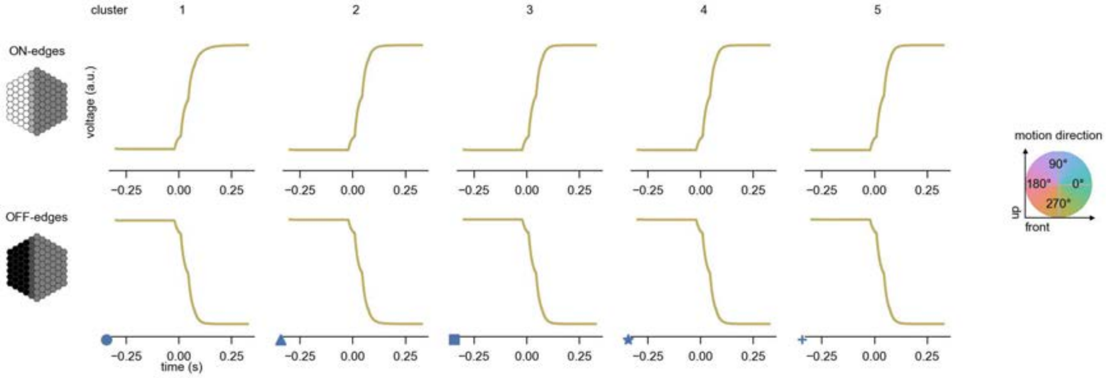

R8 - Figure 8: **Responses to moving edges from task-optimal models.** Responses to moving ON-edges (top row) and to moving OFF-edges (bottom row). Edges move in different directions from 0 to 360 degrees and at different speeds. Responses are from the task-optimal model in the respective cluster. Edges moving at 75.4°/s in all cardinal directions (green 0°, blue 90°, red 180°, yellow 270°) from -22.5 to 22.5° visual angle.

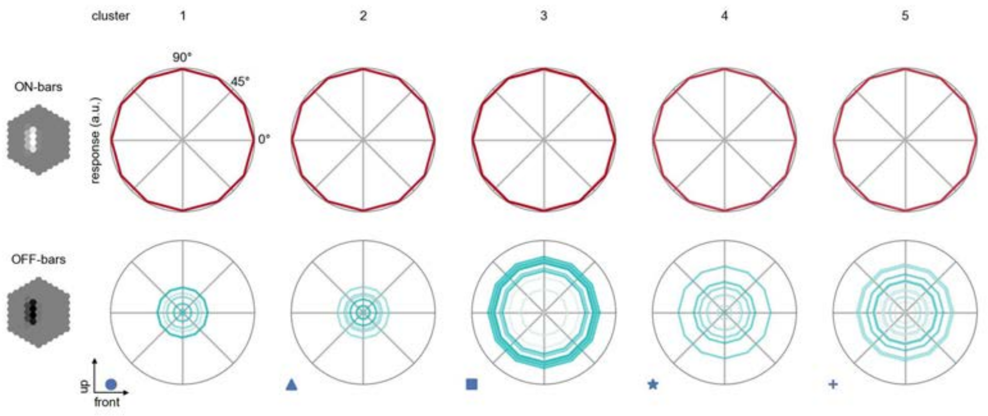

R8 - Figure 9: **Peak responses to moving bars.** The top row shows peak responses to moving ON-bars (red), the bottom row shows peak responses to moving OFF-bars (turqoise). The peak responses are averaged over bar-speeds. Bar-stimuli move in different directions from 0 to 360 degrees. The responses from the different models in the different clusters (columns) overlay. Responses from better task-performing models are more saturated.

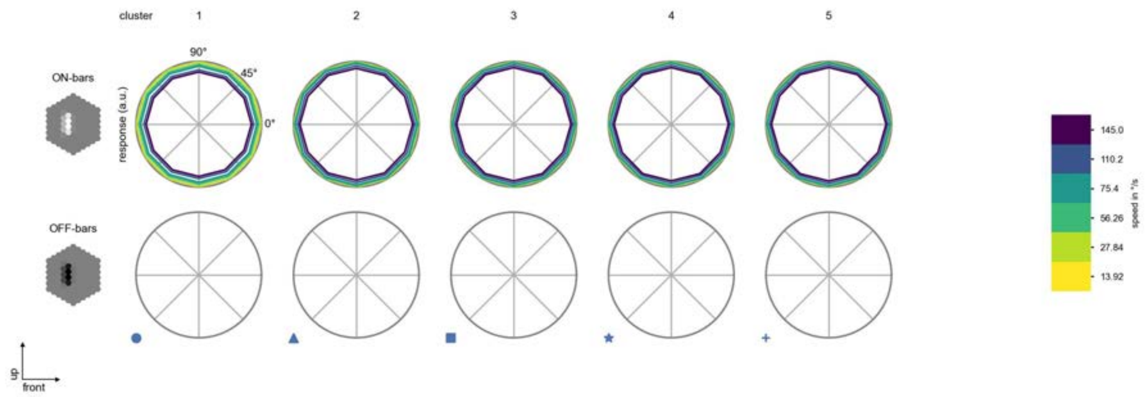

R8 - Figure 10: **Peak responses to moving bars from task-optimal models.** The top row shows peak responses to moving ON-bars, the bottom row shows peak responses to moving OFF-bars of varying speeds from 13.92°/s to 145°/s (yellow to dark blue). The bar-stimuli move in different directions from 0 to 360 degrees and at different speeds. Responses from the task-optimal model in the respective cluster.

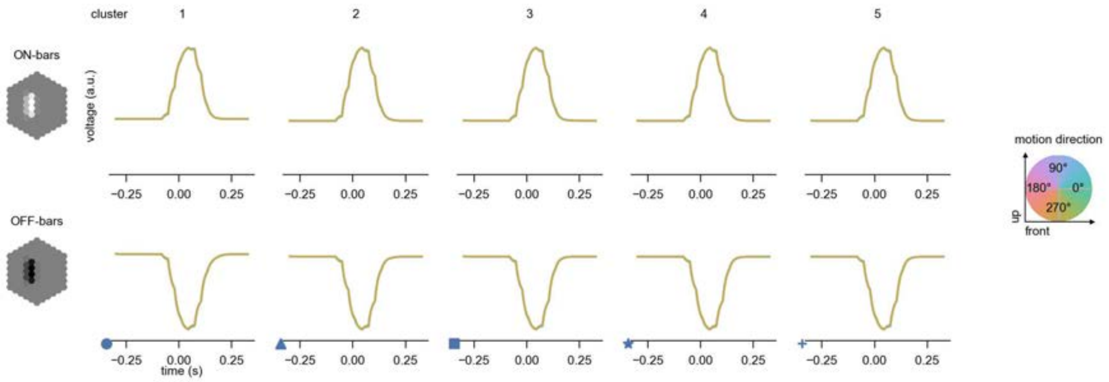

R8 - Figure 11: **Responses to moving bars from task-optimal models.** Responses to moving ON-bars (top row) and to moving OFF-bars (bottom row). Bars move in different directions from 0 to 360 degrees and at different speeds. Responses are from the task-optimal model in the respective cluster. Bars moving at 75.4°/s in all cardinal directions (green 0°, blue 90°, red 180°, yellow 270°) from -22.5 to 22.5° visual angle.

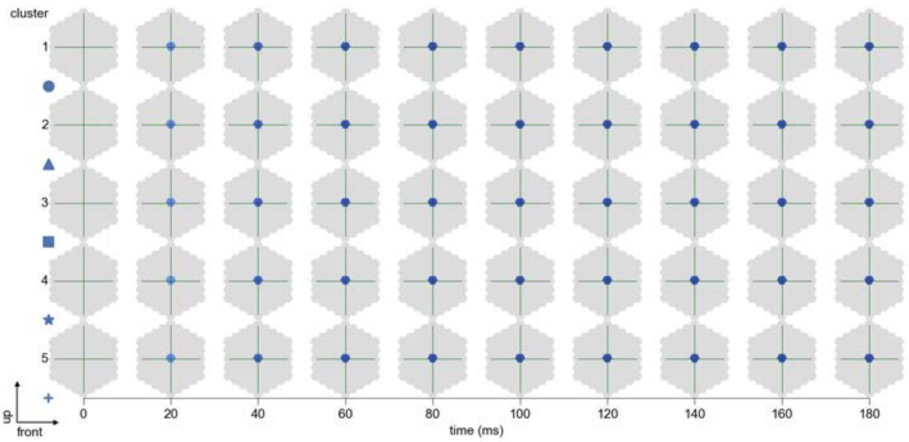

R8 - Figure 12: **Spatio-temporal receptive field.** Responses of the central cell to ON-impulses (5 ms) at single-ommatidium flash locations. The flash occurs at second zero. Responses from the task-optimal model of the respective cluster (rows). Red indicates depolarization, blue indicates hyperpolarization.

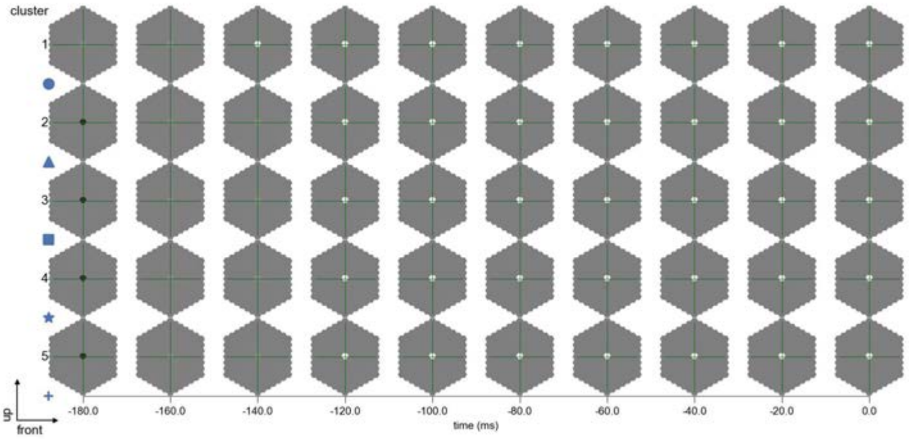

R8 - Figure 13: **Maximally excitatory stimuli.** Each row presents the regularized naturalistic-stimulus from the Sintel dataset that maximizes the cell type's central column response at second zero in the task-optimal model of the respective cluster (rows).

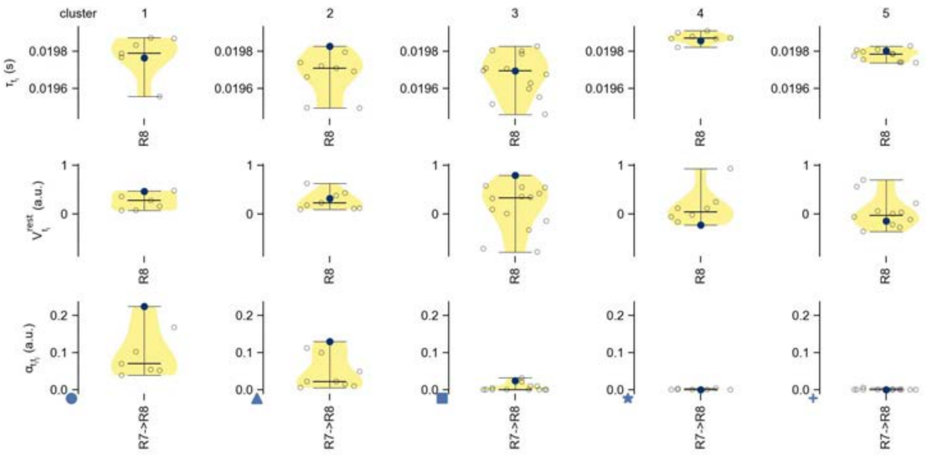

R8 - Figure 14: **Task-constrained parameters.** Each column shows the parameters inferred within the respective cluster. First row: learned time constants of the cell type. Second row: resting potentials of the cell type. Third row: scaling factors for the convolutional filters. The blue scatter represents the parameters from the task-optimal model within the cluster.

9 L1

← Cell types

Figures

1 Anatomical receptive fields. 56
2 Anatomical projective fields. 57
3 Clustering of the responses to naturalistic stimuli. 57
4 Responses to flashes. 58
5 Cluster-average responses to single-ommatidium flashes. 58
6 Peak responses to moving edges. 59
7 Peak responses to moving edges from task-optimal models. 59
8 Responses to moving edges from task-optimal models. 60
9 Peak responses to moving bars. 60
10 Peak responses to moving bars from task-optimal models. 61
11 Responses to moving bars from task-optimal models. 61
12 Spatio-temporal receptive field. 62
13 Maximally excitatory stimuli. 62
14 Task-constrained parameters. 63

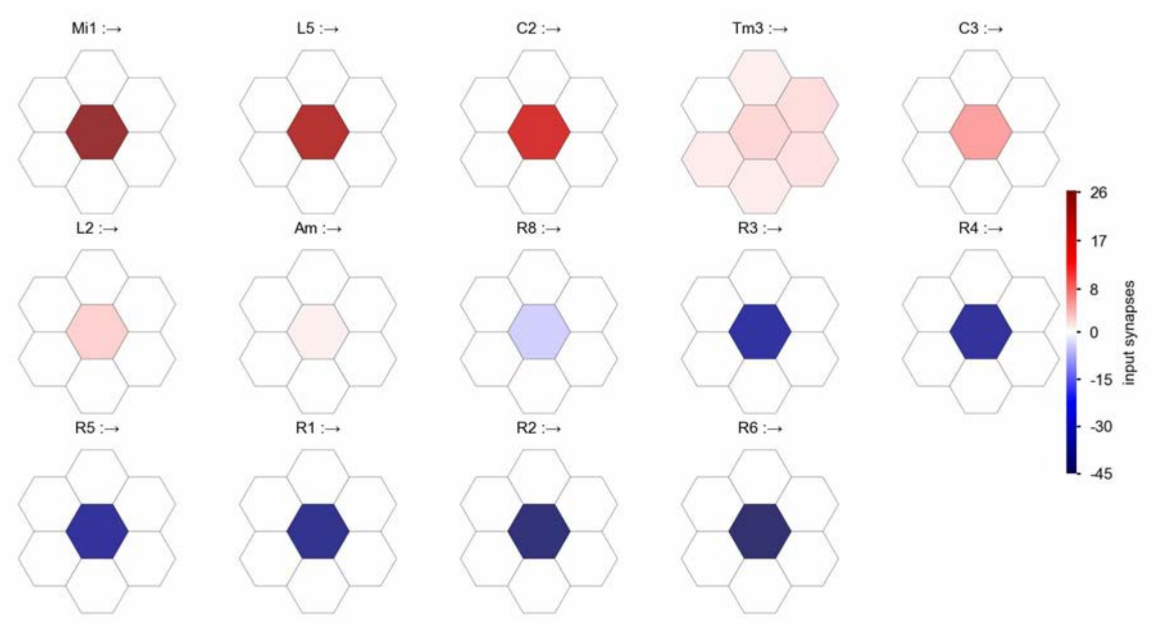

L1 - Figure 1: **Anatomical receptive fields.** Each colored hexagon is an input connection, with the connection strength characterized by the average number of synapses that we count from the EM reconstruction. Red indicates excitatory synapses, blue indicates inhibitory synapses from inferred signs. Filters in the order of their total number of synapses.

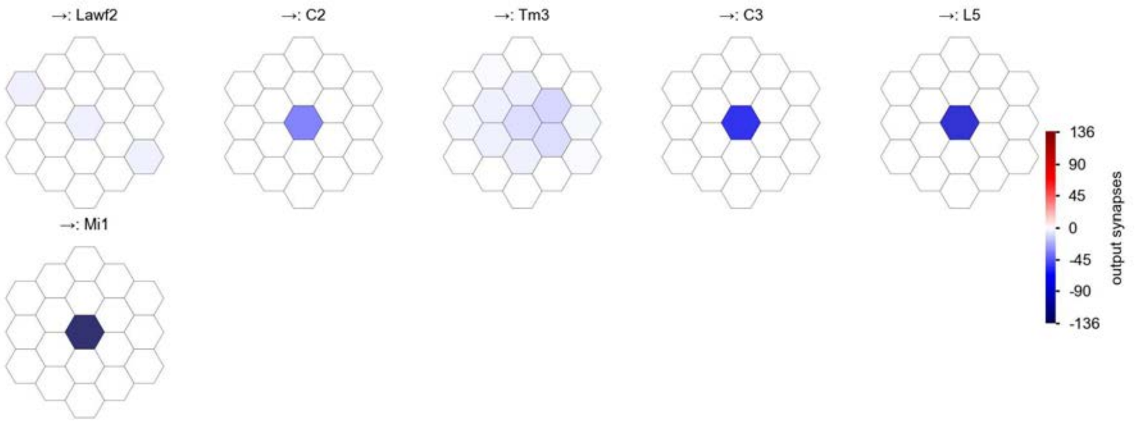

L1 - Figure 2: **Anatomical projective fields.** Each colored hexagon is an output connection, with the connection strength characterized by the average number of synapses that we count from the EM reconstruction. Red indicates excitatory synapses, blue indicates inhibitory synapses from inferred signs. Filters in the order of their total number of synapses.

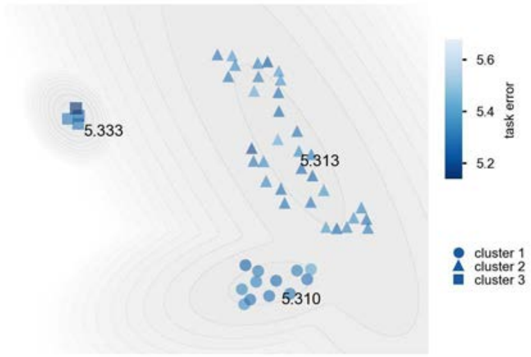

L1 - Figure 3: **Clustering of the responses to naturalistic stimuli.** Clustering of the 50 models based on the cell type responses to naturalistic scenes from the Sintel dataset. Scatterpoints represent individual models colored by their task error.

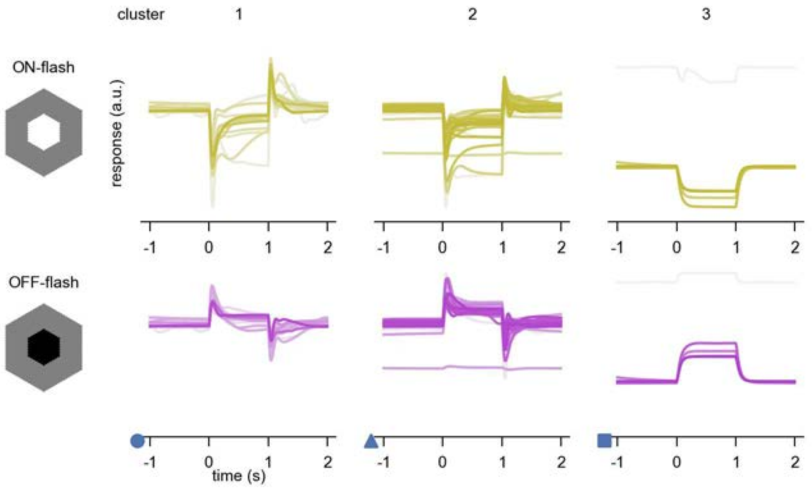

L1 - Figure 4: **Responses to flashes.** The top row shows responses to ON-flashes (yellow), the bottom row shows responses to OFF-flashes (magenta). The responses from the 50 different models that are separated into the different clusters (columns) overlay, with better task-performing models on top. Responses from better task-performing models are more saturated. The circular flashes (1s) cover 6 ommatidia in radius and are presented at time zero. Before and after, a grey-stimulus leads to a stationary state of the network.

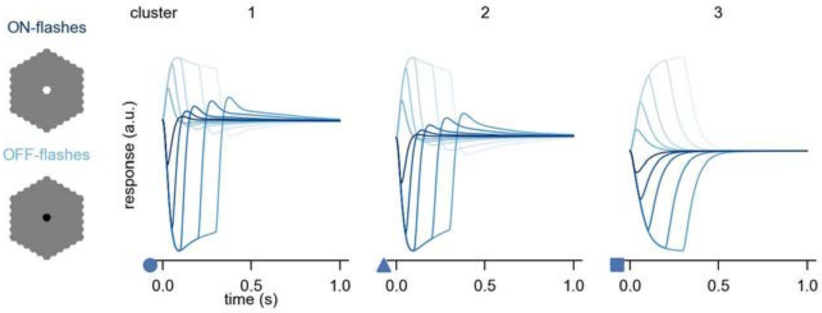

L1 - Figure 5: **Cluster-average responses to single-ommatidium flashes.** Responses to single-ommatidium ON-flashes (dark blue shades) and single-ommatidium OFF-flashes (light blue shades) of 20ms, 50ms, 100ms, 200ms, 300ms duration. The flashes occur at second zero.

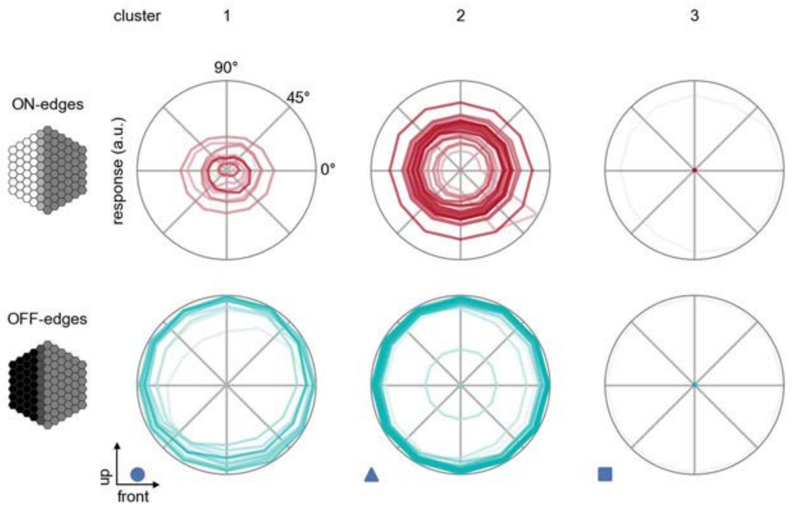

L1 - Figure 6: **Peak responses to moving edges.** The top row shows peak responses to moving ON-edges (red), the bottom row shows peak responses to moving OFF-edges (turqoise). The peak responses are averaged over edge-speeds. Edge-stimuli move in different directions from 0 to 360 degrees. The responses from the different models in the different clusters (columns) overlay. Responses from better task-performing models are more saturated.

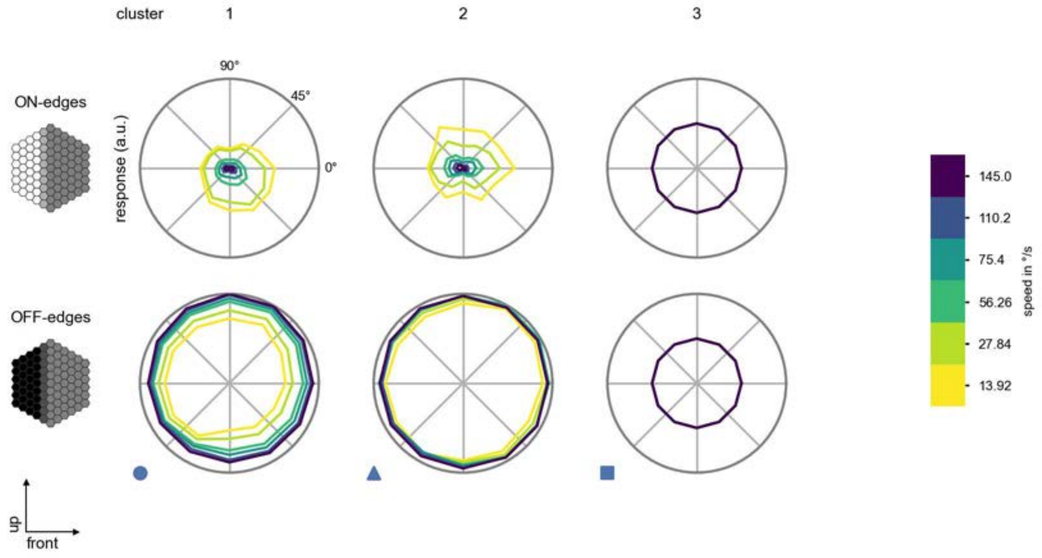

L1 - Figure 7: **Peak responses to moving edges from task-optimal models.** The top row shows peak responses to moving ON-edges, the bottom row shows peak responses to moving OFF-edges of varying speeds from 13.92°/s to 145°/s (yellow to dark blue). The edge-stimuli move in different directions from 0 to 360 degrees and at different speeds. Responses from the task-optimal model in the respective cluster.

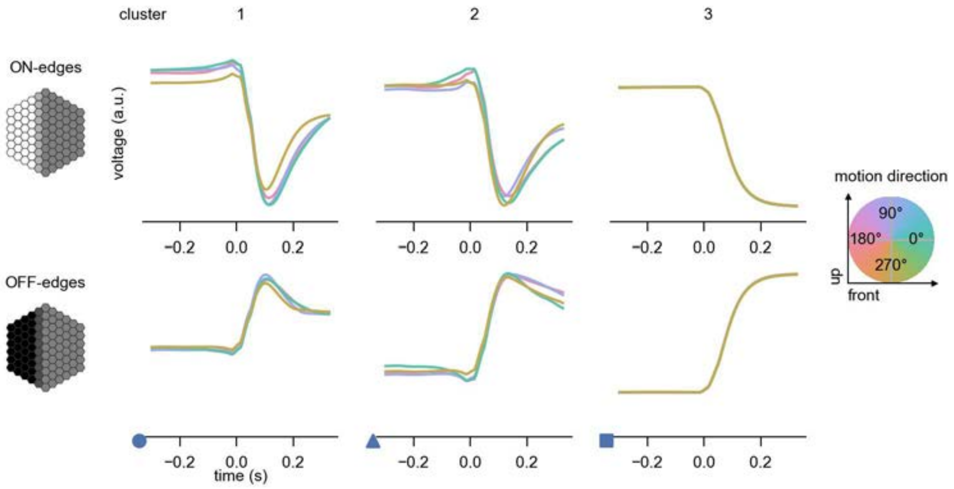

L1 - Figure 8: **Responses to moving edges from task-optimal models.** Responses to moving ON-edges (top row) and to moving OFF-edges (bottom row). Edges move in different directions from 0 to 360 degrees and at different speeds. Responses are from the task-optimal model in the respective cluster. Edges moving at 75.4°/s in all cardinal directions (green 0°, blue 90°, red 180°, yellow 270°) from -22.5 to 22.5° visual angle.

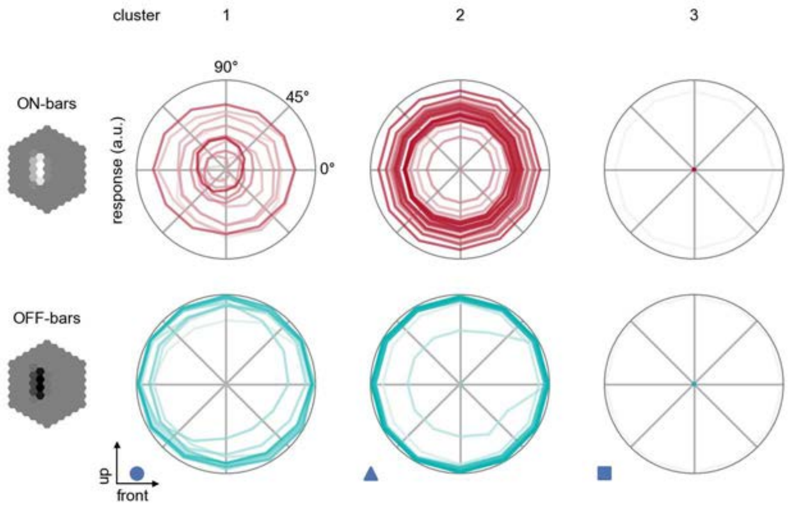

L1 - Figure 9: **Peak responses to moving bars.** The top row shows peak responses to moving ON-bars (red), the bottom row shows peak responses to moving OFF-bars (turqoise). The peak responses are averaged over bar-speeds. Bar-stimuli move in different directions from 0 to 360 degrees. The responses from the different models in the different clusters (columns) overlay. Responses from better task-performing models are more saturated.

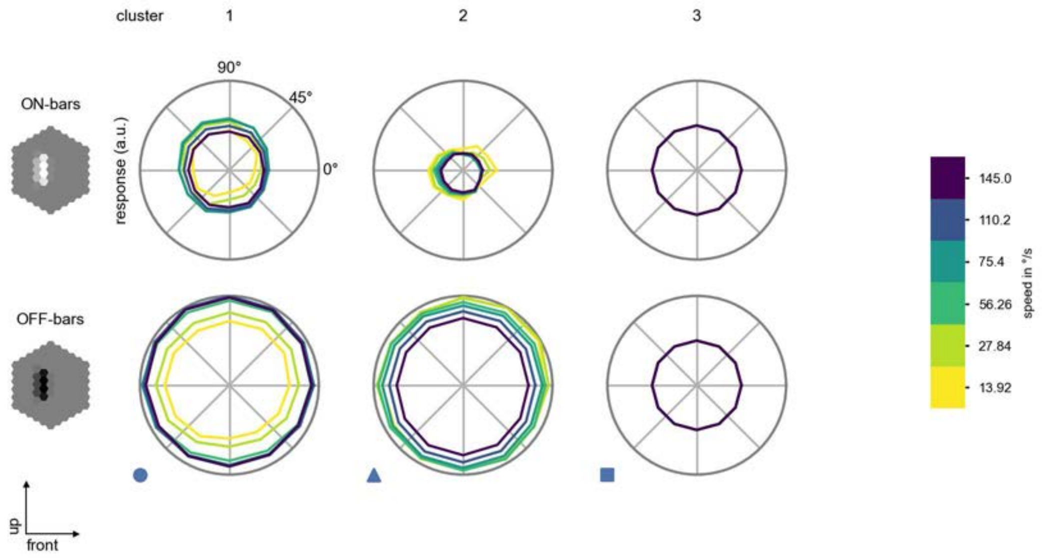

L1 - Figure 10: **Peak responses to moving bars from task-optimal models.** The top row shows peak responses to moving ON-bars, the bottom row shows peak responses to moving OFF-bars of varying speeds from 13.92°/s to 145°/s (yellow to dark blue). The bar-stimuli move in different directions from 0 to 360 degrees and at different speeds. Responses from the task-optimal model in the respective cluster.

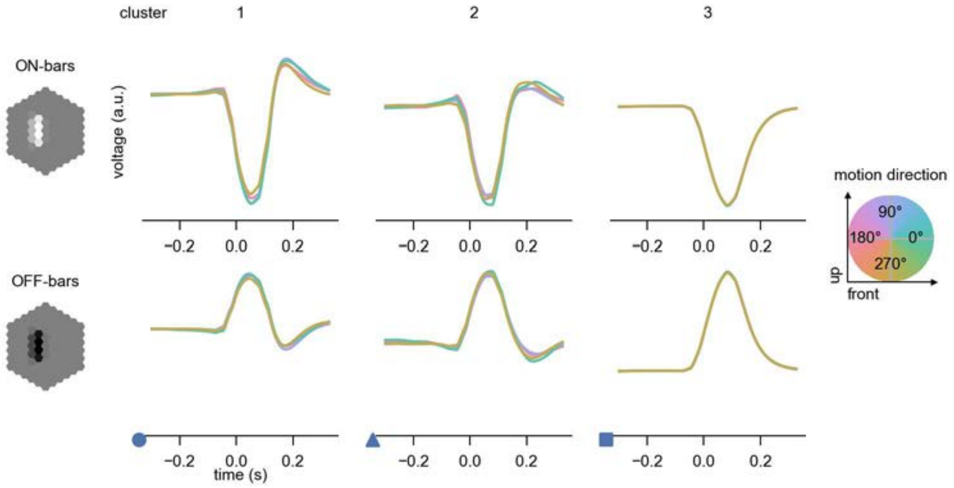

L1 - Figure 11: **Responses to moving bars from task-optimal models.** Responses to moving ON-bars (top row) and to moving OFF-bars (bottom row). Bars move in different directions from 0 to 360 degrees and at different speeds. Responses are from the task-optimal model in the respective cluster. Bars moving at 75.4°/s in all cardinal directions (green 0°, blue 90°, red 180°, yellow 270°) from -22.5 to 22.5° visual angle.

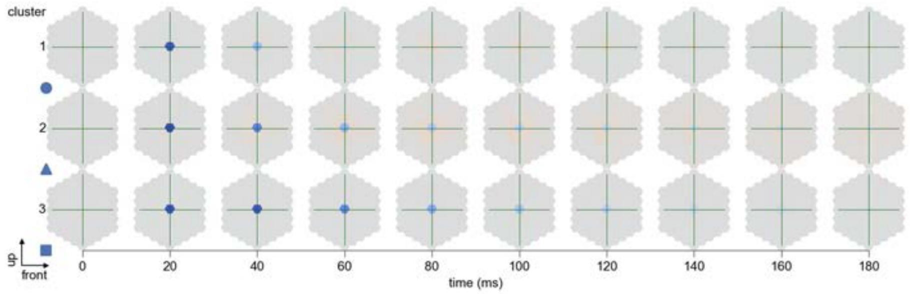

L1 - Figure 12: **Spatio-temporal receptive field.** Responses of the central cell to ON-impulses (5 ms) at single-ommatidium flash locations. The flash occurs at second zero. Responses from the task-optimal model of the respective cluster (rows). Red indicates depolarization, blue indicates hyperpolarization.

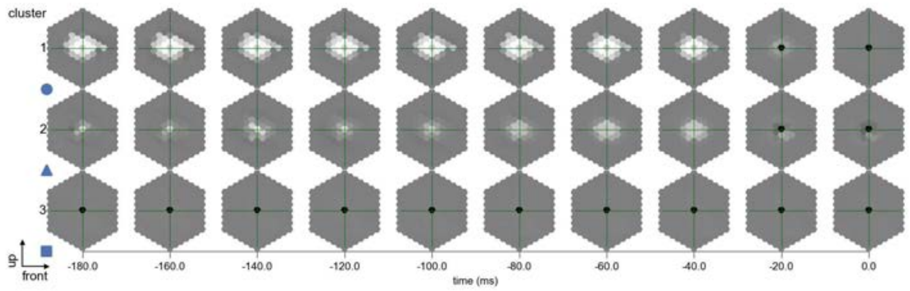

L1 - Figure 13: **Maximally excitatory stimuli.** Each row presents the regularized naturalistic-stimulus from the Sintel dataset that maximizes the cell type's central column response at second zero in the task-optimal model of the respective cluster (rows).

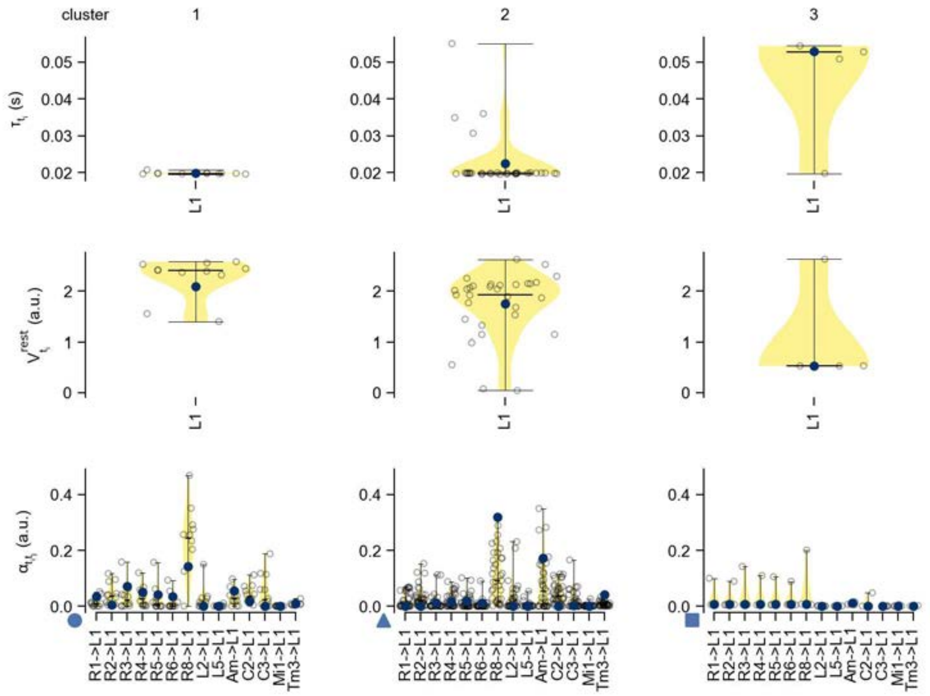

L1 - Figure 14: **Task-constrained parameters.** Each column shows the parameters inferred within the respective cluster. First row: learned time constants of the cell type. Second row: resting potentials of the cell type. Third row: scaling factors for the convolutional filters. The blue scatter represents the parameters from the task-optimal model within the cluster.

10 L2

← Cell types

Figures

1 Anatomical receptive fields. 64
2 Anatomical projective fields. 65
3 Clustering of the responses to naturalistic stimuli. 65
4 Responses to flashes. 66
5 Cluster-average responses to single-ommatidium flashes. 66
6 Peak responses to moving edges. 67
7 Peak responses to moving edges from task-optimal models. 67
8 Responses to moving edges from task-optimal models. 68
9 Peak responses to moving bars. 68
10 Peak responses to moving bars from task-optimal models. 69
11 Responses to moving bars from task-optimal models. 69
12 Spatio-temporal receptive field. 69
13 Maximally excitatory stimuli. 70
14 Task-constrained parameters. 70

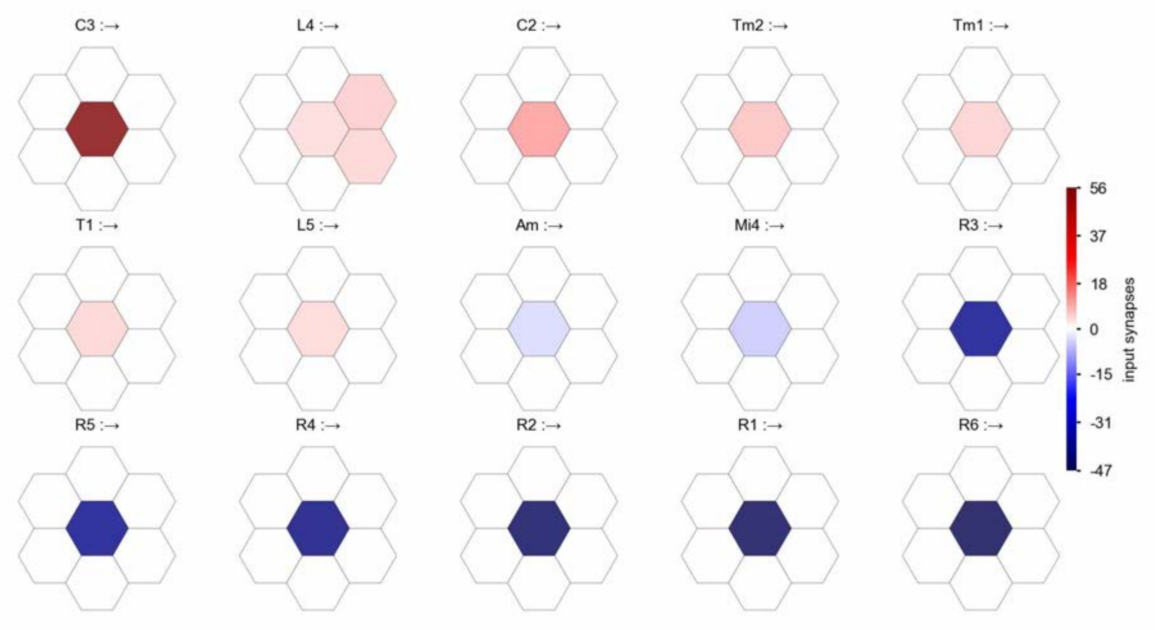

L2 - Figure 1: **Anatomical receptive fields.** Each colored hexagon is an input connection, with the connection strength characterized by the average number of synapses that we count from the EM reconstruction. Red indicates excitatory synapses, blue indicates inhibitory synapses from inferred signs. Filters in the order of their total number of synapses.

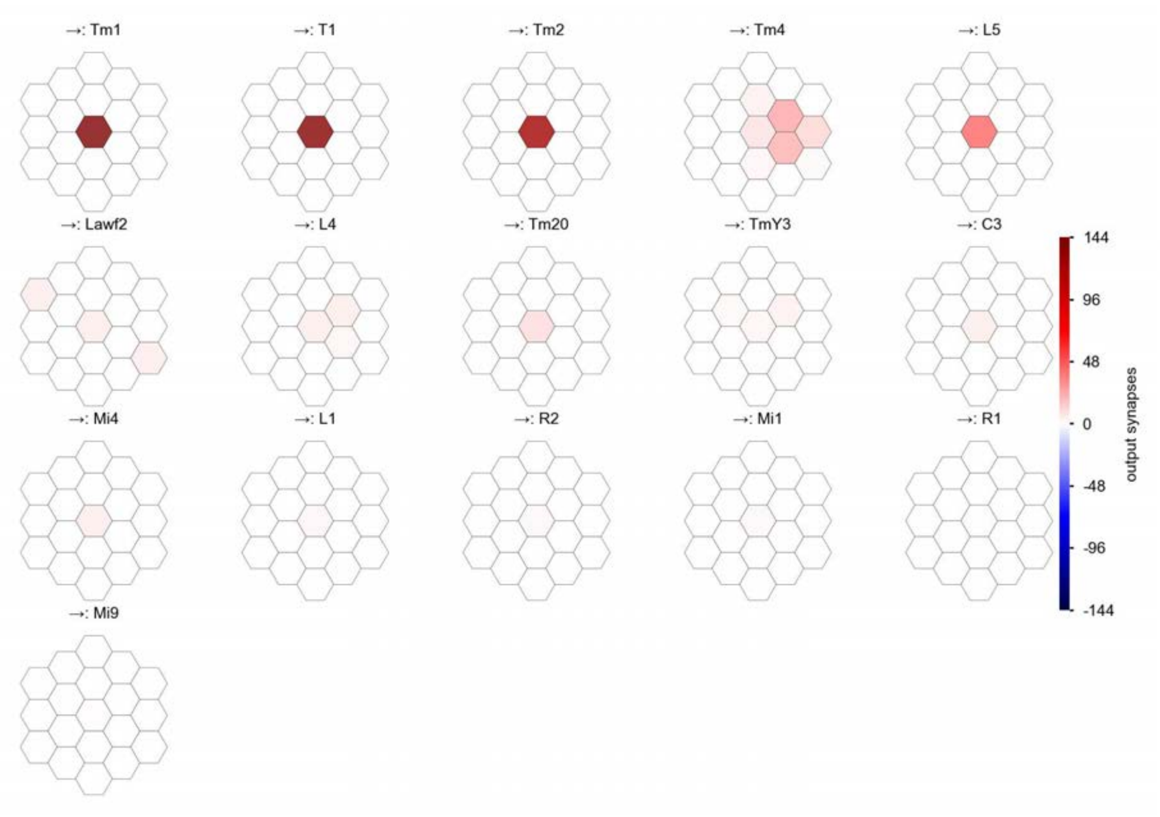

L2 - Figure 2: **Anatomical projective fields.** Each colored hexagon is an output connection, with the connection strength characterized by the average number of synapses that we count from the EM reconstruction. Red indicates excitatory synapses, blue indicates inhibitory synapses from inferred signs. Filters in the order of their total number of synapses.

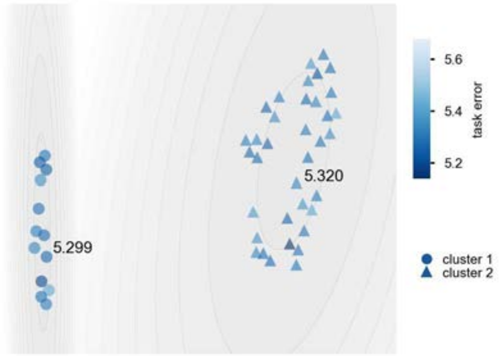

L2 - Figure 3: **Clustering of the responses to naturalistic stimuli.** Clustering of the 50 models based on the cell type responses to naturalistic scenes from the Sintel dataset. Scatterpoints represent individual models colored by their task error.

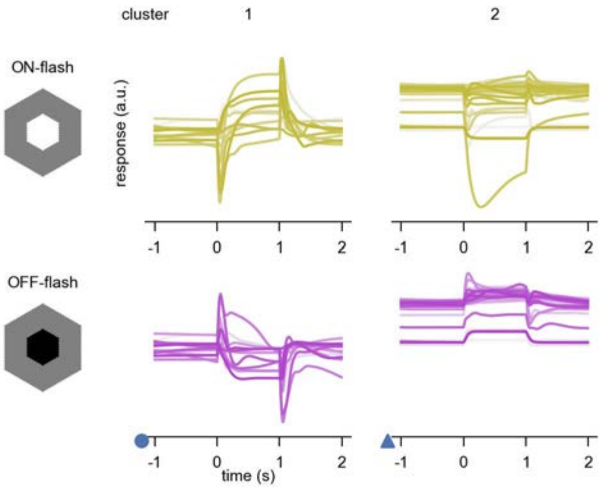

L2 - Figure 4: **Responses to flashes.** The top row shows responses to ON-flashes (yellow), the bottom row shows responses to OFF-flashes (magenta). The responses from the 50 different models that are separated into the different clusters (columns) overlay, with better task-performing models on top. Responses from better task-performing models are more saturated. The circular flashes (1s) cover 6 ommatidia in radius and are presented at time zero. Before and after, a grey-stimulus leads to a stationary state of the network.

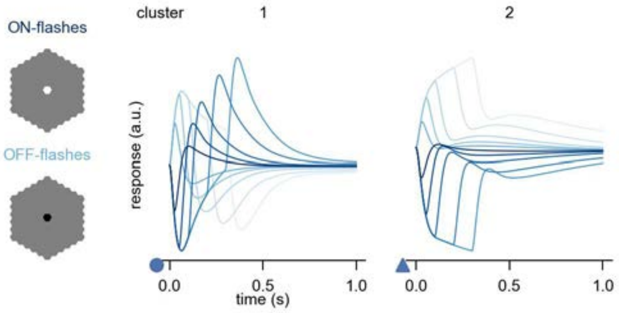

L2 - Figure 5: **Cluster-average responses to single-ommatidium flashes.** Responses to single-ommatidium ON-flashes (dark blue shades) and single-ommatidium OFF-flashes (light blue shades) of 20ms, 50ms, 100ms, 200ms, 300ms duration. The flashes occur at second zero.

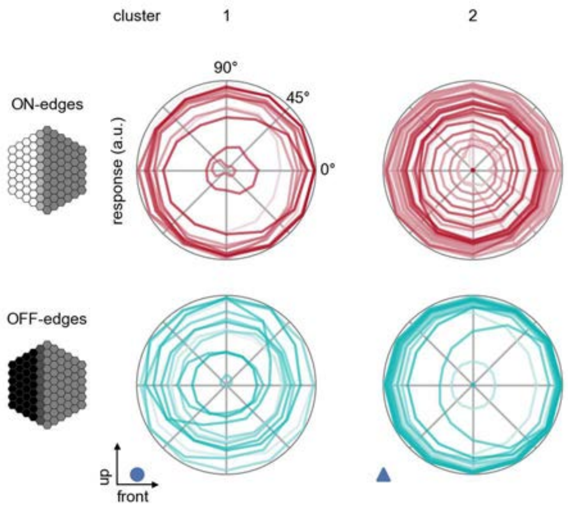

L2 - Figure 6: **Peak responses to moving edges.** The top row shows peak responses to moving ON-edges (red), the bottom row shows peak responses to moving OFF-edges (turqoise). The peak responses are averaged over edge-speeds. Edge-stimuli move in different directions from 0 to 360 degrees. The responses from the different models in the different clusters (columns) overlay. Responses from better task-performing models are more saturated.

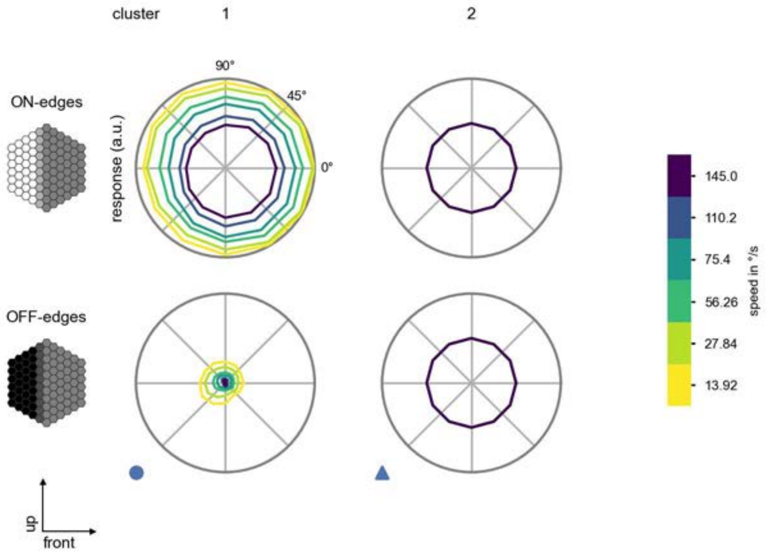

L2 - Figure 7: **Peak responses to moving edges from task-optimal models.** The top row shows peak responses to moving ON-edges, the bottom row shows peak responses to moving OFF-edges of varying speeds from 13.92°/s to 145°/s (yellow to dark blue). The edge-stimuli move in different directions from 0 to 360 degrees and at different speeds. Responses from the task-optimal model in the respective cluster.

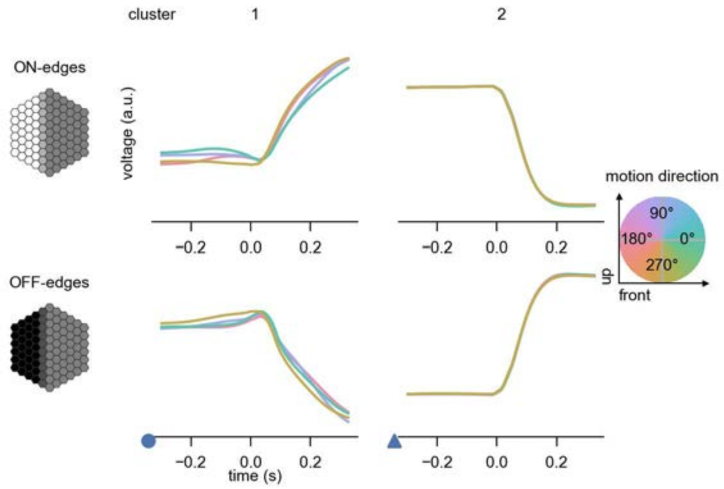

L2 - Figure 8: **Responses to moving edges from task-optimal models.** Responses to moving ON-edges (top row) and to moving OFF-edges (bottom row). Edges move in different directions from 0 to 360 degrees and at different speeds. Responses are from the task-optimal model in the respective cluster. Edges moving at 75.4°/s in all cardinal directions (green 0°, blue 90°, red 180°, yellow 270°) from -22.5 to 22.5° visual angle.

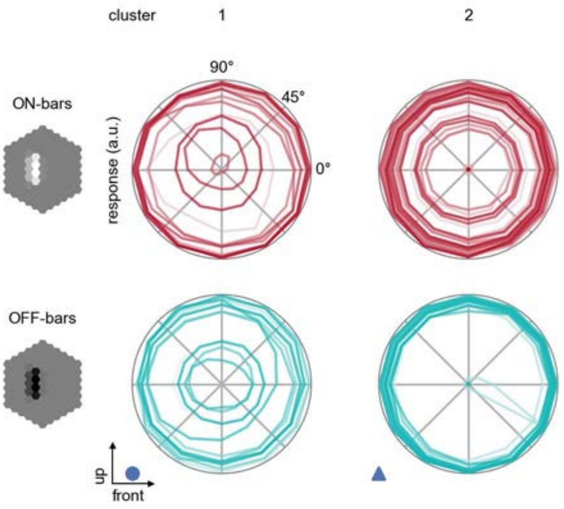

L2 - Figure 9: **Peak responses to moving bars.** The top row shows peak responses to moving ON-bars (red), the bottom row shows peak responses to moving OFF-bars (turqoise). The peak responses are averaged over bar-speeds. Bar-stimuli move in different directions from 0 to 360 degrees. The responses from the different models in the different clusters (columns) overlay. Responses from better task-performing models are more saturated.

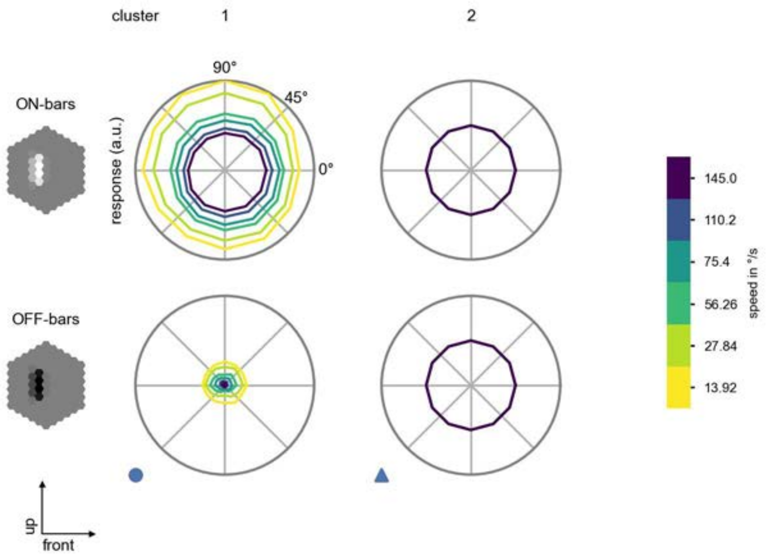

L2 - Figure 10: **Peak responses to moving bars from task-optimal models.** The top row shows peak responses to moving ON-bars, the bottom row shows peak responses to moving OFF-bars of varying speeds from 13.92°/s to 145°/s (yellow to dark blue). The bar-stimuli move in different directions from 0 to 360 degrees and at different speeds. Responses from the task-optimal model in the respective cluster.

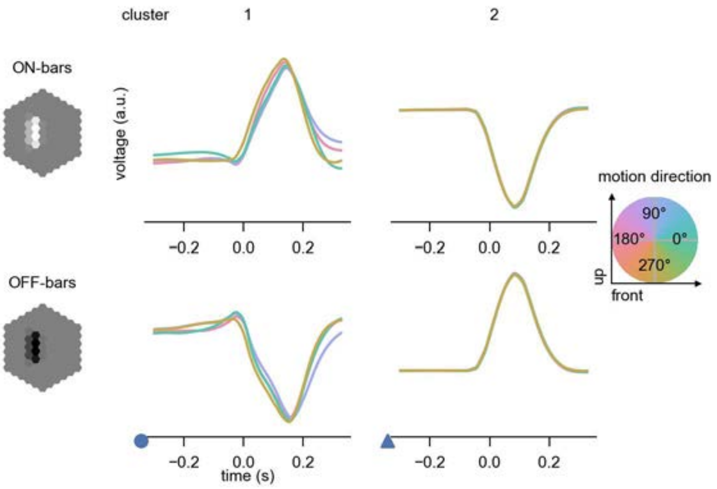

L2 - Figure 11: **Responses to moving bars from task-optimal models.** Responses to moving ON-bars (top row) and to moving OFF-bars (bottom row). Bars move in different directions from 0 to 360 degrees and at different speeds. Responses are from the task-optimal model in the respective cluster. Bars moving at 75.4°/s in all cardinal directions (green 0°, blue 90°, red 180°, yellow 270°) from -22.5 to 22.5° visual angle.

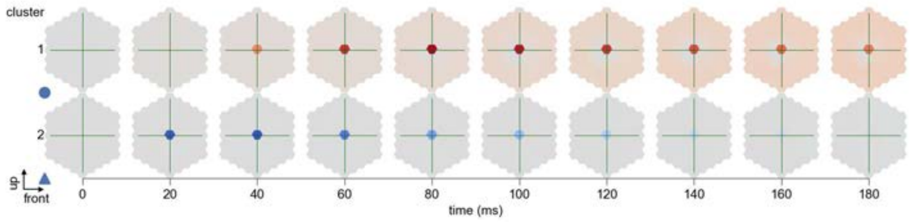

L2 - Figure 12: **Spatio-temporal receptive field.** Responses of the central cell to ON-impulses (5 ms) at single-ommatidium flash locations. The flash occurs at second zero. Responses from the task-optimal model of the respective cluster (rows). Red indicates depolarization, blue indicates hyperpolarization.

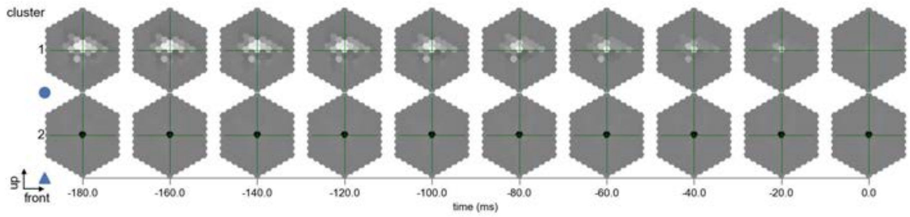

L2 - Figure 13: **Maximally excitatory stimuli.** Each row presents the regularized naturalistic-stimulus from the Sintel dataset that maximizes the cell type's central column response at second zero in the task-optimal model of the respective cluster (rows).

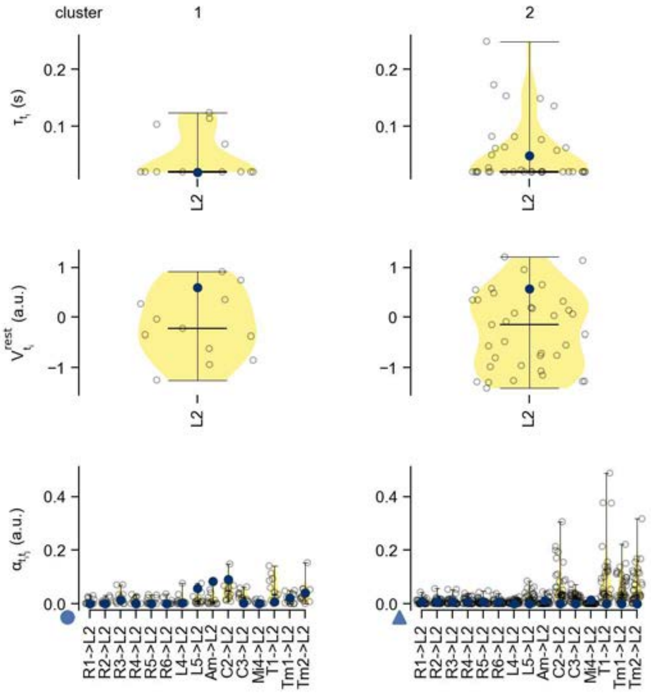

L2 - Figure 14: **Task-constrained parameters.** Each column shows the parameters inferred within the respective cluster. First row: learned time constants of the cell type. Second row: resting potentials of the cell type. Third row: scaling factors for the convolutional filters. The blue scatter represents the parameters from the task-optimal model within the cluster.

11 L3

← Cell types

Figures

1 Anatomical receptive fields. . . . 71
2 Anatomical projective fields. . . . 72
3 Clustering of the responses to naturalistic stimuli. . . . 72
4 Responses to flashes. . . . 73
5 Cluster-average responses to single-ommatidium flashes. . . . 73
6 Peak responses to moving edges. . . . 74
7 Peak responses to moving edges from task-optimal models. . . . 74
8 Responses to moving edges from task-optimal models. . . . 75
9 Peak responses to moving bars. . . . 75
10 Peak responses to moving bars from task-optimal models. . . . 76
11 Responses to moving bars from task-optimal models. . . . 76
12 Spatio-temporal receptive field. . . . 77
13 Maximally excitatory stimuli. . . . 77
14 Task-constrained parameters. . . . 78

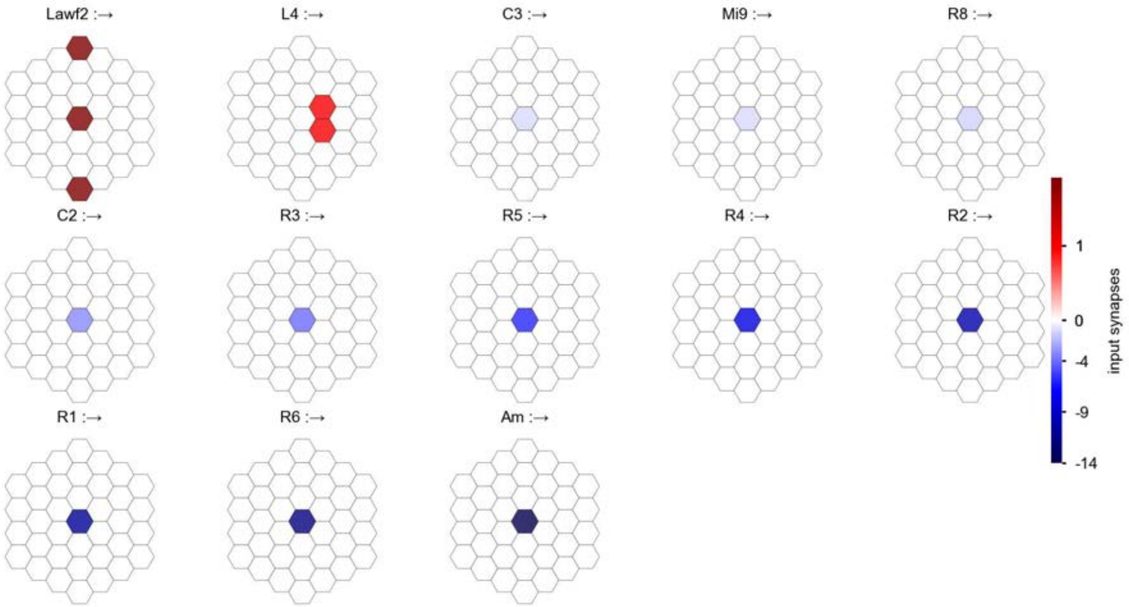

L3 - Figure 1: **Anatomical receptive fields.** Each colored hexagon is an input connection, with the connection strength characterized by the average number of synapses that we count from the EM reconstruction. Red indicates excitatory synapses, blue indicates inhibitory synapses from inferred signs. Filters in the order of their total number of synapses.

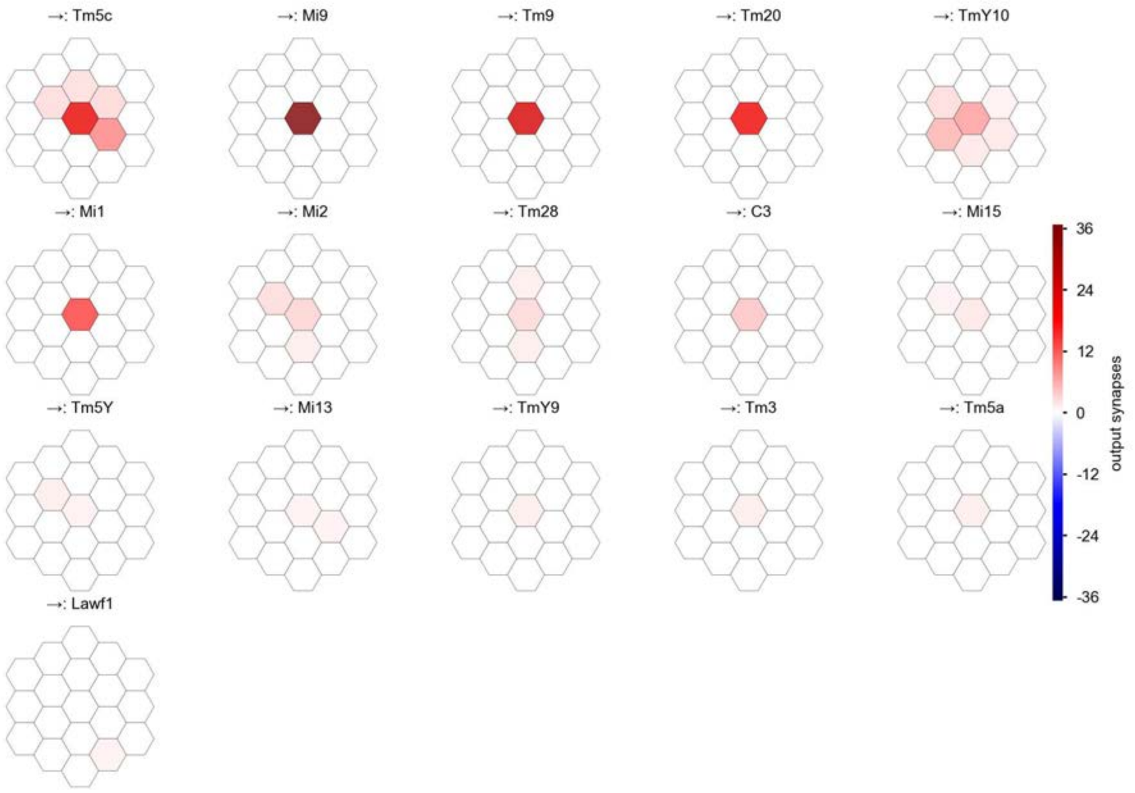

L3 - Figure 2: **Anatomical projective fields.** Each colored hexagon is an output connection, with the connection strength characterized by the average number of synapses that we count from the EM reconstruction. Red indicates excitatory synapses, blue indicates inhibitory synapses from inferred signs. Filters in the order of their total number of synapses.

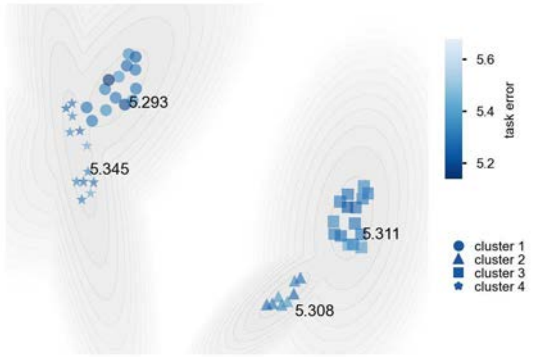

L3 - Figure 3: **Clustering of the responses to naturalistic stimuli.** Clustering of the 50 models based on the cell type responses to naturalistic scenes from the Sintel dataset. Scatterpoints represent individual models colored by their task error.

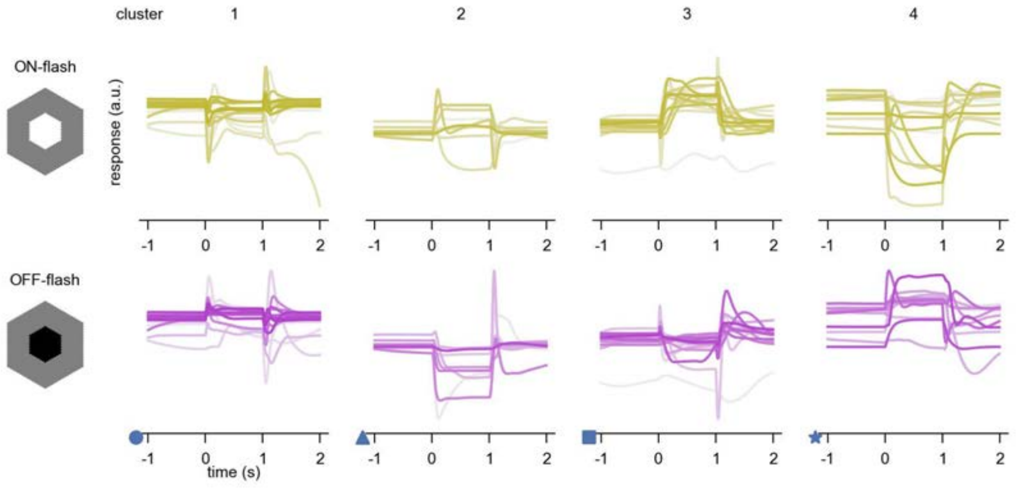

L3 - Figure 4: **Responses to flashes.** The top row shows responses to ON-flashes (yellow), the bottom row shows responses to OFF-flashes (magenta). The responses from the 50 different models that are separated into the different clusters (columns) overlay, with better task-performing models on top. Responses from better task-performing models are more saturated. The circular flashes (1s) cover 6 ommatidia in radius and are presented at time zero. Before and after, a grey-stimulus leads to a stationary state of the network.

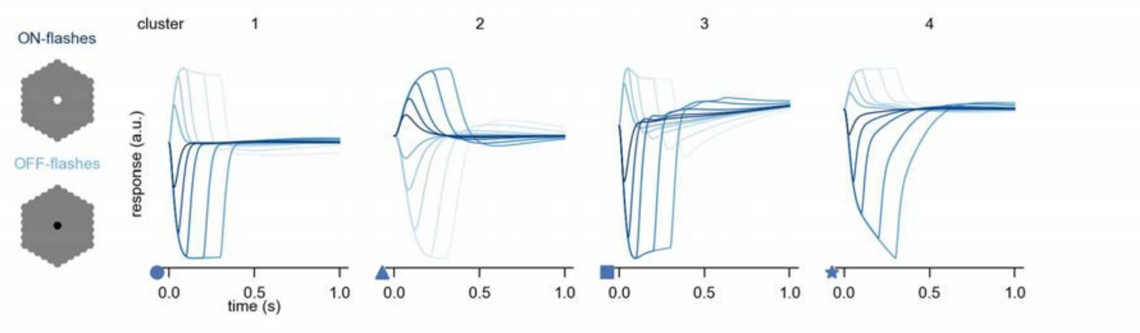

L3 - Figure 5: **Cluster-average responses to single-ommatidium flashes.** Responses to single-ommatidium ON-flashes (dark blue shades) and single-ommatidium OFF-flashes (light blue shades) of 20ms, 50ms, 100ms, 200ms, 300ms duration. The flashes occur at second zero.

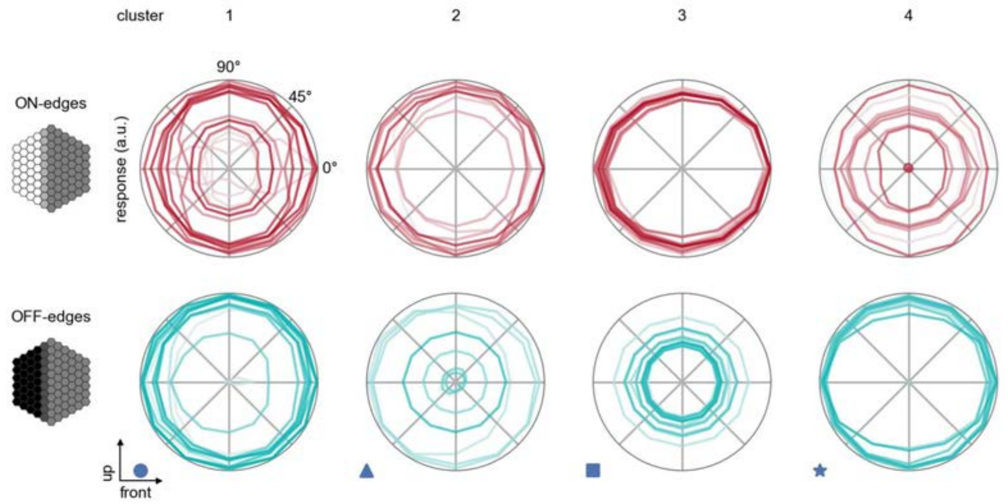

L3 - Figure 6: **Peak responses to moving edges.** The top row shows peak responses to moving ON-edges (red), the bottom row shows peak responses to moving OFF-edges (turqoise). The peak responses are averaged over edge-speeds. Edge-stimuli move in different directions from 0 to 360 degrees. The responses from the different models in the different clusters (columns) overlay. Responses from better task-performing models are more saturated.

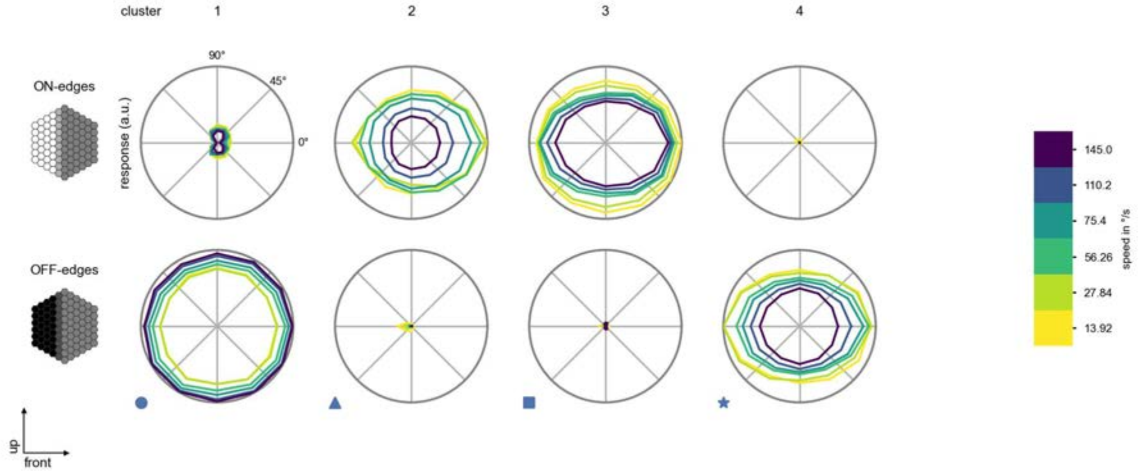

L3 - Figure 7: **Peak responses to moving edges from task-optimal models.** The top row shows peak responses to moving ON-edges, the bottom row shows peak responses to moving OFF-edges of varying speeds from 13.92°/s to 145°/s (yellow to dark blue). The edge-stimuli move in different directions from 0 to 360 degrees and at different speeds. Responses from the task-optimal model in the respective cluster.

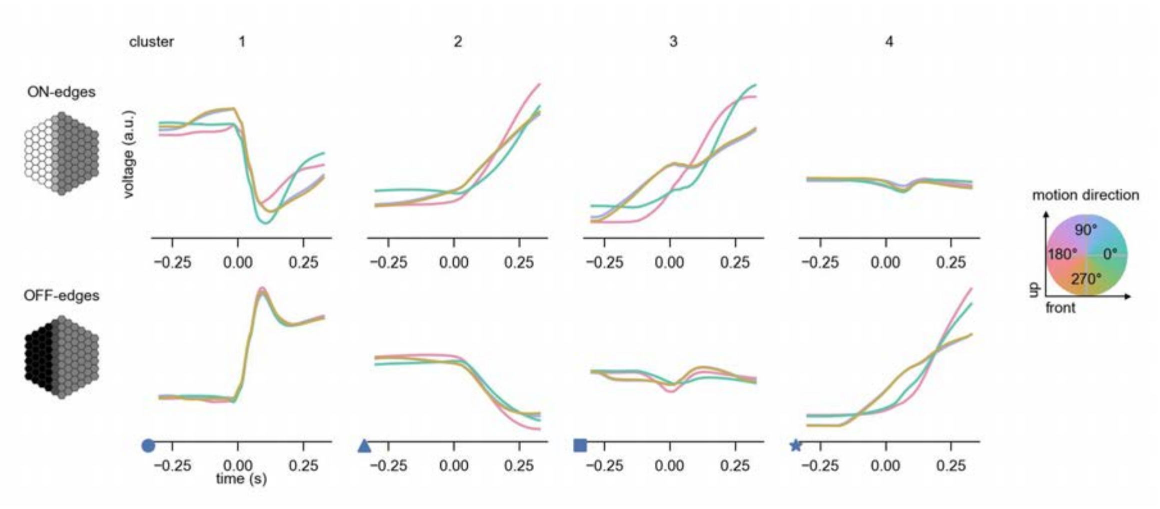

L3 - Figure 8: **Responses to moving edges from task-optimal models.** Responses to moving ON-edges (top row) and to moving OFF-edges (bottom row). Edges move in different directions from 0 to 360 degrees and at different speeds. Responses are from the task-optimal model in the respective cluster. Edges moving at 75.4°/s in all cardinal directions (green 0°, blue 90°, red 180°, yellow 270°) from -22.5 to 22.5° visual angle.

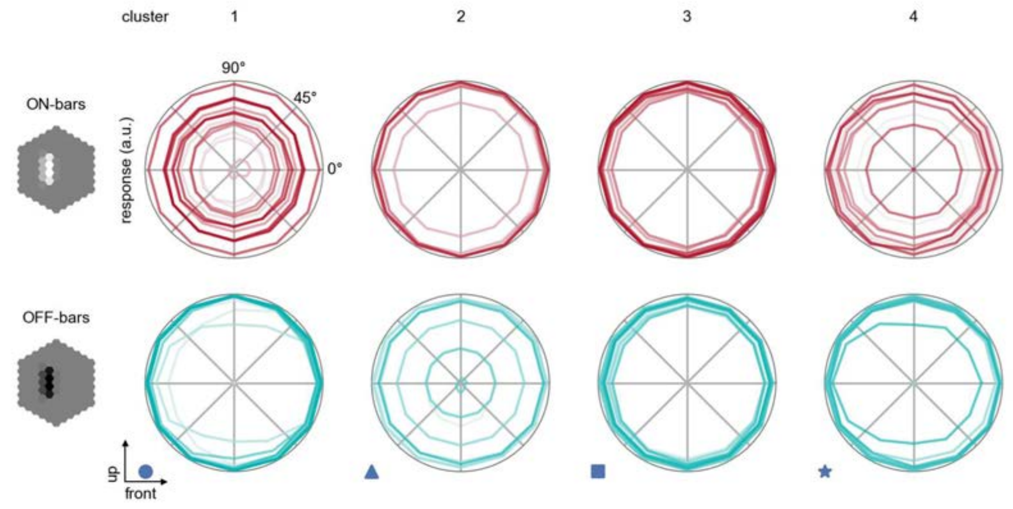

L3 - Figure 9: **Peak responses to moving bars.** The top row shows peak responses to moving ON-bars (red), the bottom row shows peak responses to moving OFF-bars (turqoise). The peak responses are averaged over bar-speeds. Bar-stimuli move in different directions from 0 to 360 degrees. The responses from the different models in the different clusters (columns) overlay. Responses from better task-performing models are more saturated.

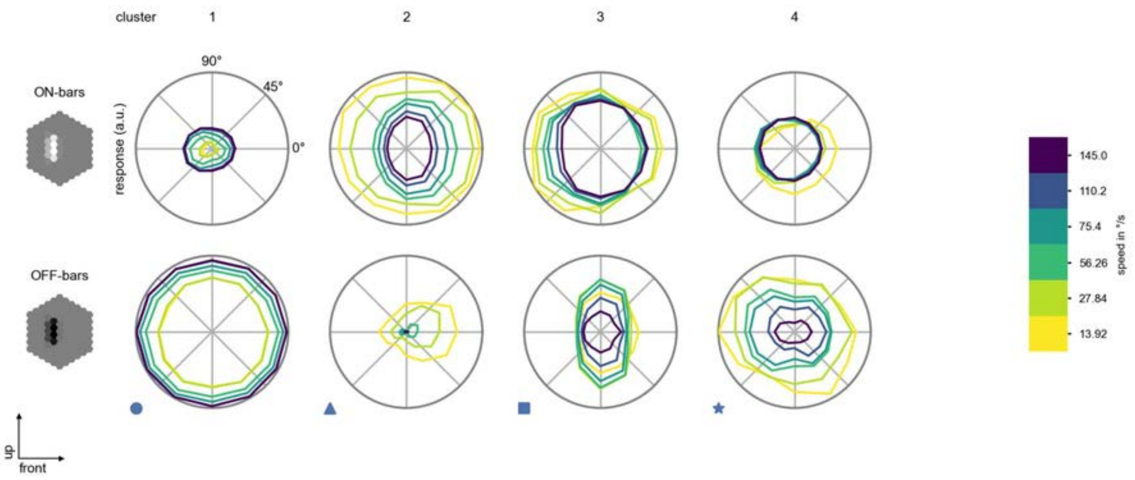

L3 - Figure 10: **Peak responses to moving bars from task-optimal models.** The top row shows peak responses to moving ON-bars, the bottom row shows peak responses to moving OFF-bars of varying speeds from 13.92°/s to 145°/s (yellow to dark blue). The bar-stimuli move in different directions from 0 to 360 degrees and at different speeds. Responses from the task-optimal model in the respective cluster.

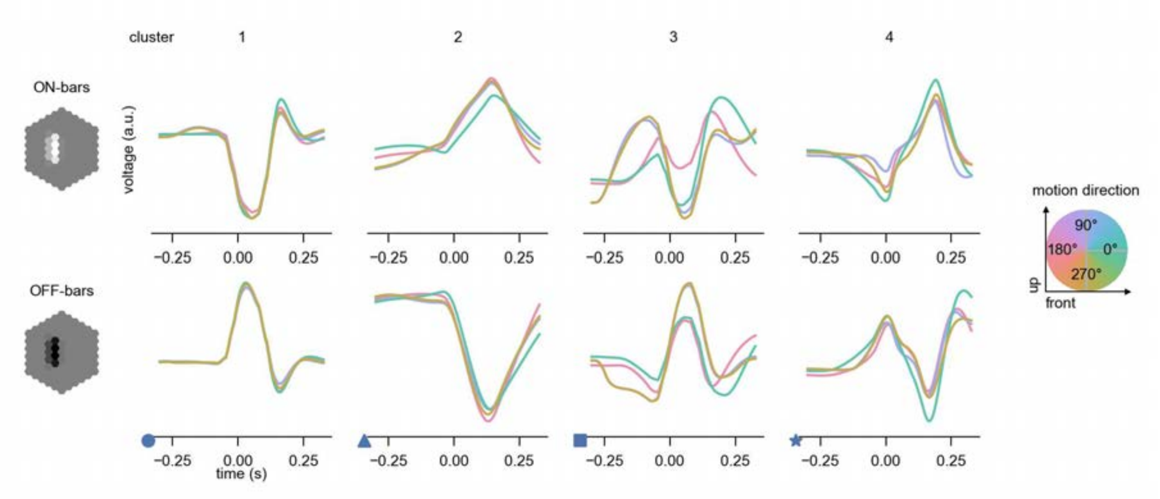

L3 - Figure 11: **Responses to moving bars from task-optimal models.** Responses to moving ON-bars (top row) and to moving OFF-bars (bottom row). Bars move in different directions from 0 to 360 degrees and at different speeds. Responses are from the task-optimal model in the respective cluster. Bars moving at 75.4°/s in all cardinal directions (green 0°, blue 90°, red 180°, yellow 270°) from -22.5 to 22.5° visual angle.

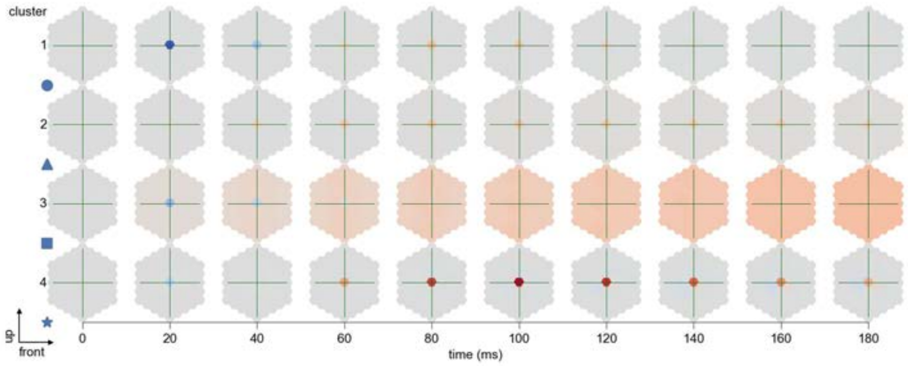

L3 - Figure 12: **Spatio-temporal receptive field.** Responses of the central cell to ON-impulses (5 ms) at single-ommatidium flash locations. The flash occurs at second zero. Responses from the task-optimal model of the respective cluster (rows). Red indicates depolarization, blue indicates hyperpolarization.

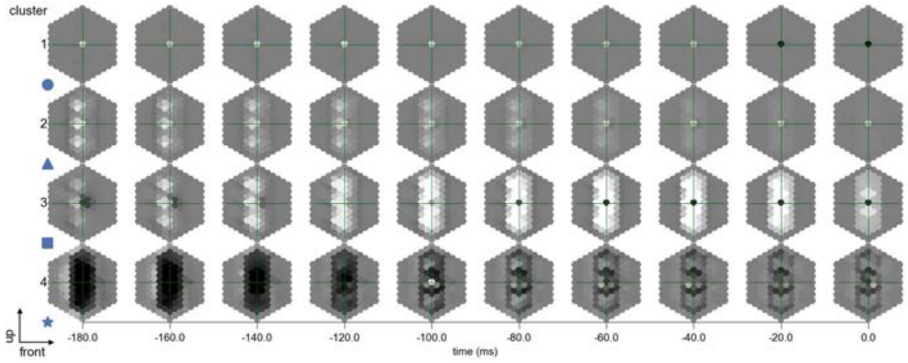

L3 - Figure 13: **Maximally excitatory stimuli.** Each row presents the regularized naturalistic-stimulus from the Sintel dataset that maximizes the cell type's central column response at second zero in the task-optimal model of the respective cluster (rows).

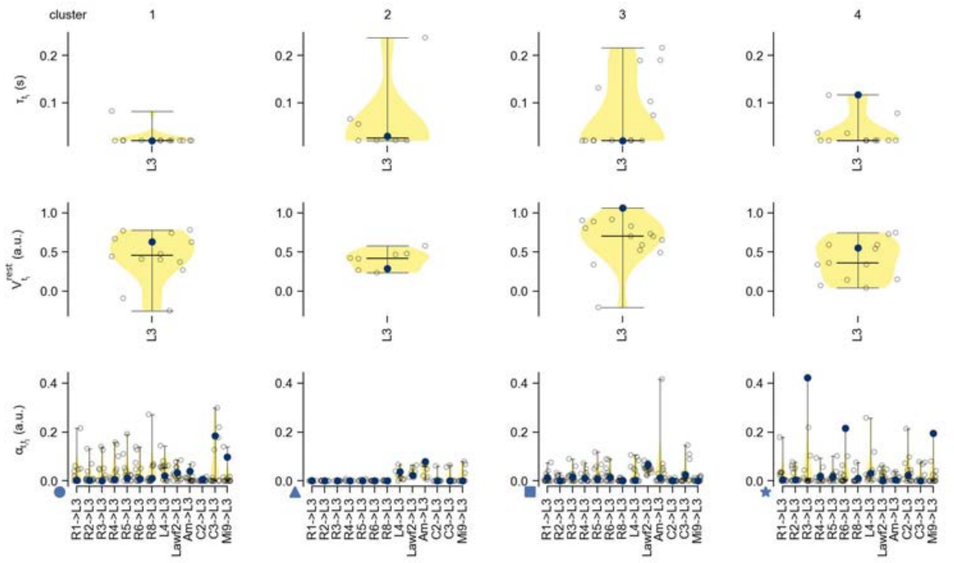

L3 - Figure 14: **Task-constrained parameters.** Each column shows the parameters inferred within the respective cluster. First row: learned time constants of the cell type. Second row: resting potentials of the cell type. Third row: scaling factors for the convolutional filters. The blue scatter represents the parameters from the task-optimal model within the cluster.

12 L4

← Cell types

Figures

1 Anatomical receptive fields. 79
2 Anatomical projective fields. 80
3 Clustering of the responses to naturalistic stimuli. 80
4 Responses to flashes. 81
5 Cluster-average responses to single-ommatidium flashes. 81
6 Peak responses to moving edges. 82
7 Peak responses to moving edges from task-optimal models. 82
8 Responses to moving edges from task-optimal models. 83
9 Peak responses to moving bars. 83
10 Peak responses to moving bars from task-optimal models. 84
11 Responses to moving bars from task-optimal models. 84
12 Spatio-temporal receptive field. 84
13 Maximally excitatory stimuli. 85
14 Task-constrained parameters. 85

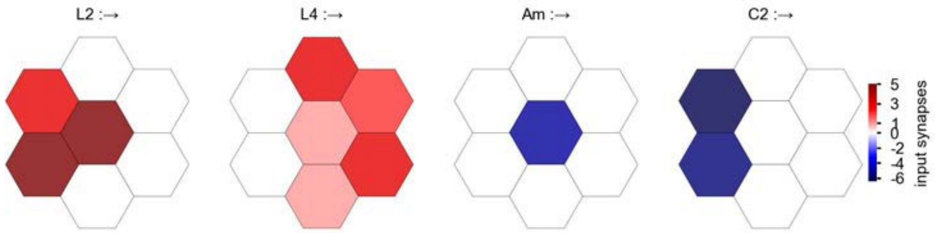

L4 - Figure 1: **Anatomical receptive fields.** Each colored hexagon is an input connection, with the connection strength characterized by the average number of synapses that we count from the EM reconstruction. Red indicates excitatory synapses, blue indicates inhibitory synapses from inferred signs. Filters in the order of their total number of synapses.

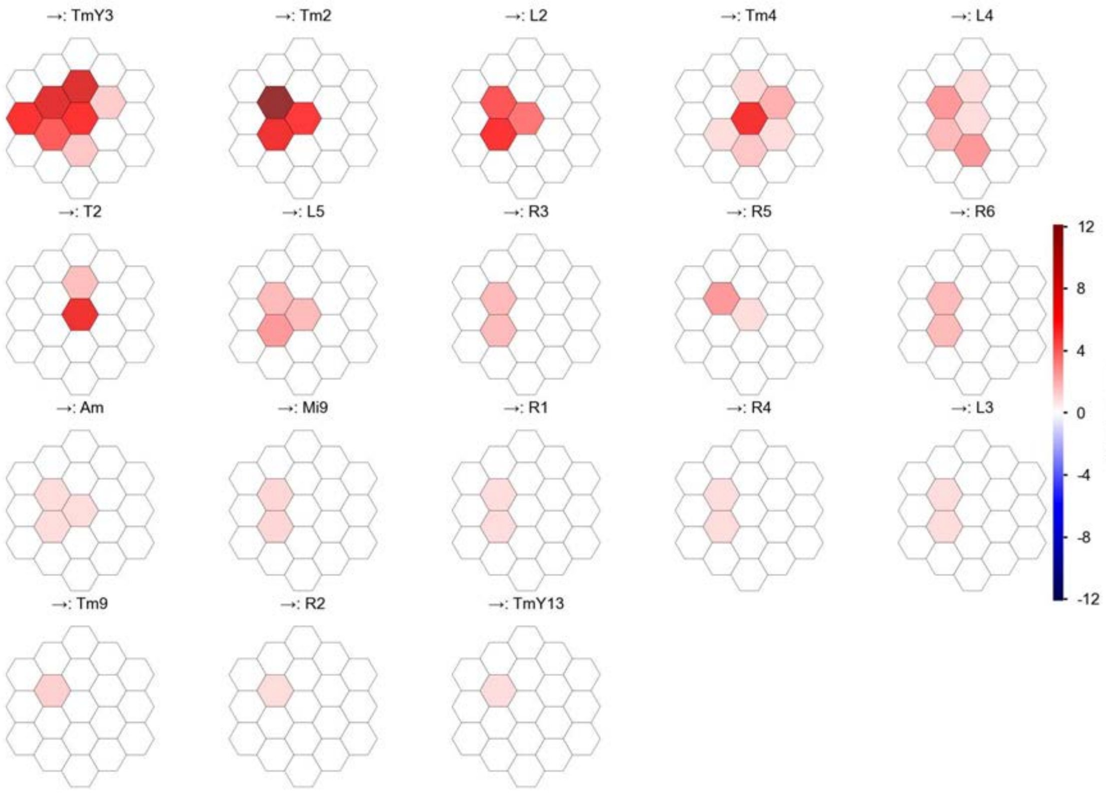

L4 - Figure 2: **Anatomical projective fields.** Each colored hexagon is an output connection, with the connection strength characterized by the average number of synapses that we count from the EM reconstruction. Red indicates excitatory synapses, blue indicates inhibitory synapses from inferred signs. Filters in the order of their total number of synapses.

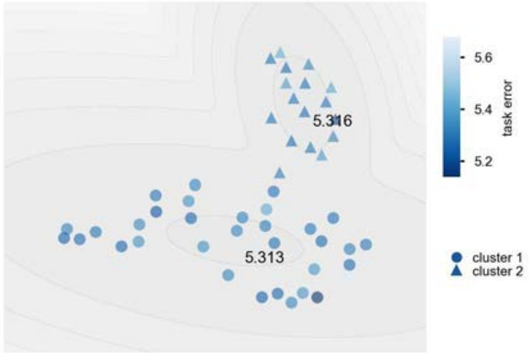

L4 - Figure 3: **Clustering of the responses to naturalistic stimuli.** Clustering of the 50 models based on the cell type responses to naturalistic scenes from the Sintel dataset. Scatterpoints represent individual models colored by their task error.

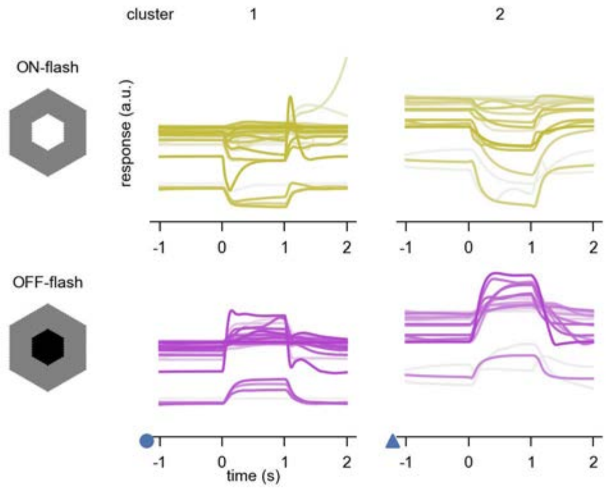

L4 - Figure 4: **Responses to flashes.** The top row shows responses to ON-flashes (yellow), the bottom row shows responses to OFF-flashes (magenta). The responses from the 50 different models that are separated into the different clusters (columns) overlay, with better task-performing models on top. Responses from better task-performing models are more saturated. The circular flashes (1s) cover 6 ommatidia in radius and are presented at time zero. Before and after, a grey-stimulus leads to a stationary state of the network.

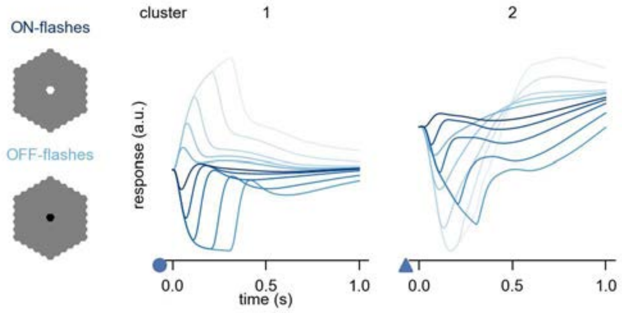

L4 - Figure 5: **Cluster-average responses to single-ommatidium flashes.** Responses to single-ommatidium ON-flashes (dark blue shades) and single-ommatidium OFF-flashes (light blue shades) of 20ms, 50ms, 100ms, 200ms, 300ms duration. The flashes occur at second zero.

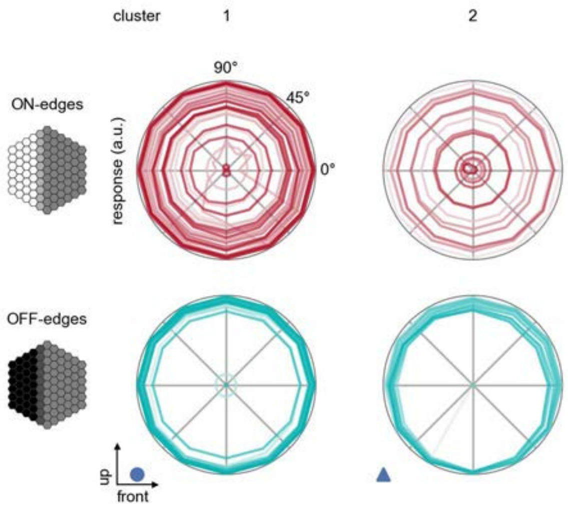

L4 - Figure 6: **Peak responses to moving edges.** The top row shows peak responses to moving ON-edges (red), the bottom row shows peak responses to moving OFF-edges (turqoise). The peak responses are averaged over edge-speeds. Edge-stimuli move in different directions from 0 to 360 degrees. The responses from the different models in the different clusters (columns) overlay. Responses from better task-performing models are more saturated.

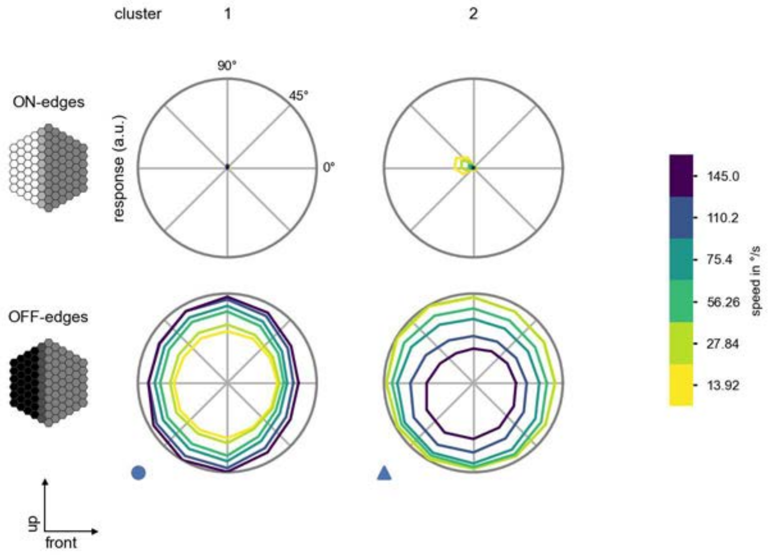

L4 - Figure 7: **Peak responses to moving edges from task-optimal models.** The top row shows peak responses to moving ON-edges, the bottom row shows peak responses to moving OFF-edges of varying speeds from 13.92°/s to 145°/s (yellow to dark blue). The edge-stimuli move in different directions from 0 to 360 degrees and at different speeds. Responses from the task-optimal model in the respective cluster.

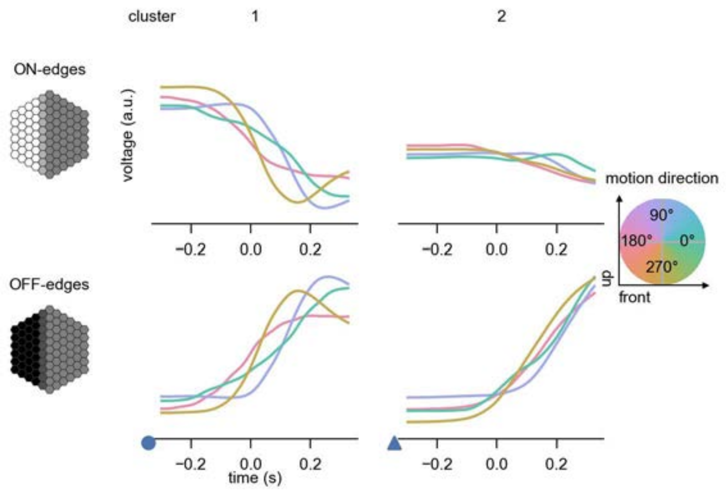

L4 - Figure 8: **Responses to moving edges from task-optimal models.** Responses to moving ON-edges (top row) and to moving OFF-edges (bottom row). Edges move in different directions from 0 to 360 degrees and at different speeds. Responses are from the task-optimal model in the respective cluster. Edges moving at 75.4°/s in all cardinal directions (green 0°, blue 90°, red 180°, yellow 270°) from -22.5 to 22.5° visual angle.

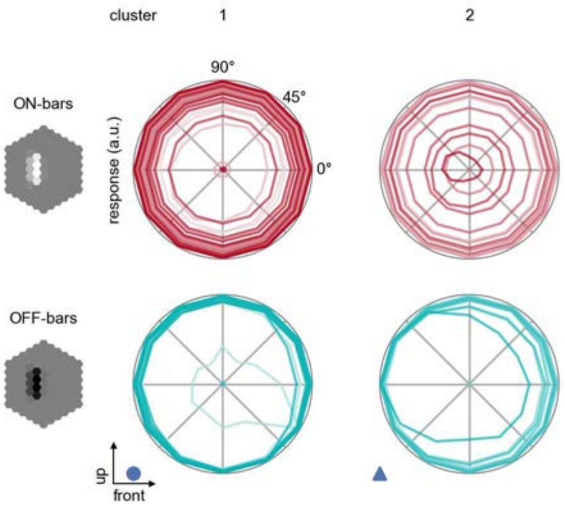

L4 - Figure 9: **Peak responses to moving bars.** The top row shows peak responses to moving ON-bars (red), the bottom row shows peak responses to moving OFF-bars (turqoise). The peak responses are averaged over bar-speeds. Bar-stimuli move in different directions from 0 to 360 degrees. The responses from the different models in the different clusters (columns) overlay. Responses from better task-performing models are more saturated.

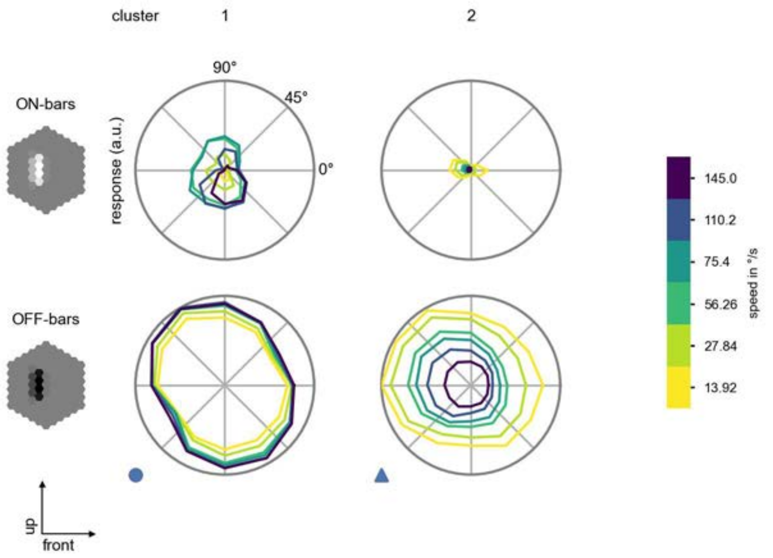

L4 - Figure 10: **Peak responses to moving bars from task-optimal models.** The top row shows peak responses to moving ON-bars, the bottom row shows peak responses to moving OFF-bars of varying speeds from 13.92°/s to 145°/s (yellow to dark blue). The bar-stimuli move in different directions from 0 to 360 degrees and at different speeds. Responses from the task-optimal model in the respective cluster.

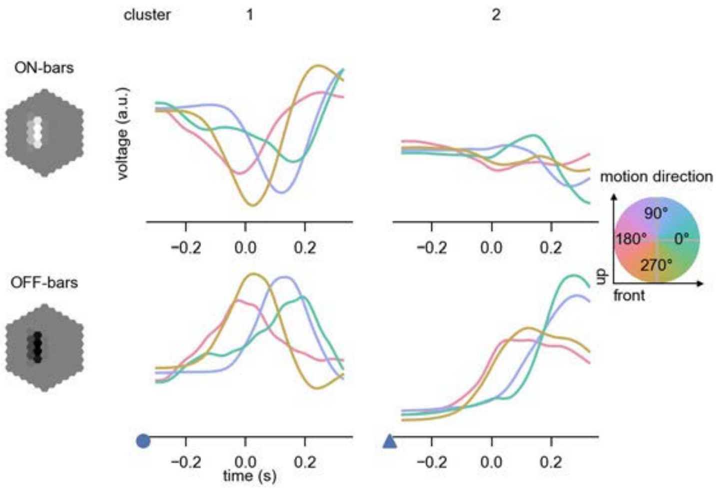

L4 - Figure 11: **Responses to moving bars from task-optimal models.** Responses to moving ON-bars (top row) and to moving OFF-bars (bottom row). Bars move in different directions from 0 to 360 degrees and at different speeds. Responses are from the task-optimal model in the respective cluster. Bars moving at 75.4°/s in all cardinal directions (green 0°, blue 90°, red 180°, yellow 270°) from -22.5 to 22.5° visual angle.

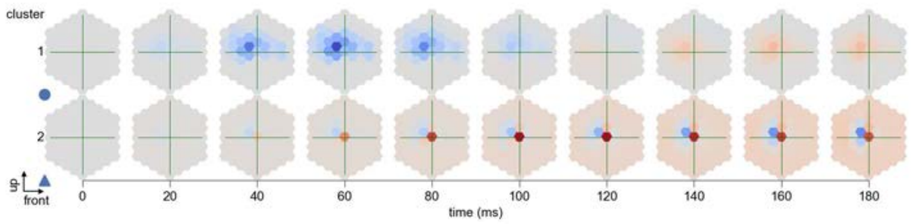

L4 - Figure 12: **Spatio-temporal receptive field.** Responses of the central cell to ON-impulses (5 ms) at single-ommatidium flash locations. The flash occurs at second zero. Responses from the task-optimal model of the respective cluster (rows). Red indicates depolarization, blue indicates hyperpolarization.

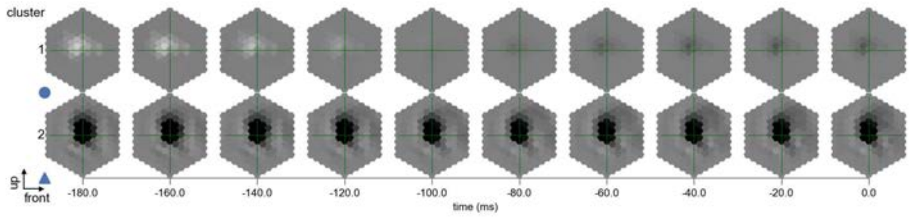

L4 - Figure 13: **Maximally excitatory stimuli.** Each row presents the regularized naturalistic-stimulus from the Sintel dataset that maximizes the cell type's central column response at second zero in the task-optimal model of the respective cluster (rows).

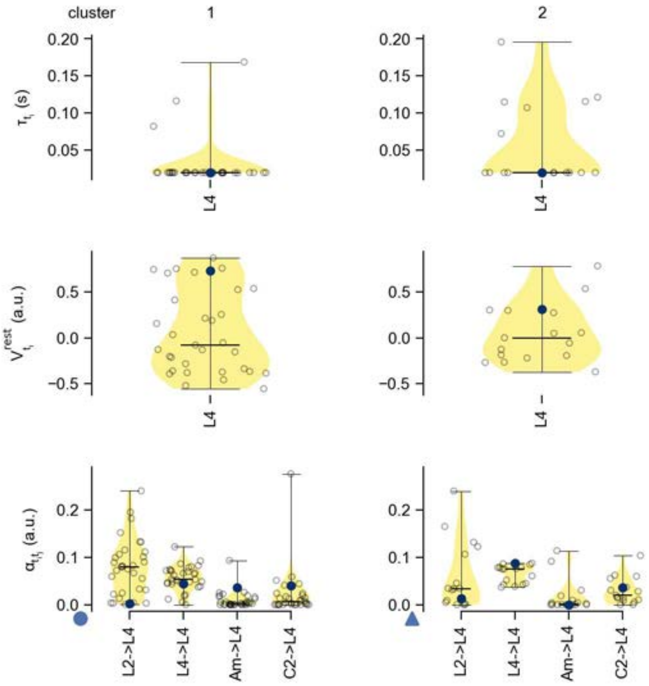

L4 - Figure 14: **Task-constrained parameters.** Each column shows the parameters inferred within the respective cluster. First row: learned time constants of the cell type. Second row: resting potentials of the cell type. Third row: scaling factors for the convolutional filters. The blue scatter represents the parameters from the task-optimal model within the cluster.

13 L5

← Cell types

Figures

1 Anatomical receptive fields. . . . 86
2 Anatomical projective fields. . . . 87
3 Clustering of the responses to naturalistic stimuli. . . . 87
4 Responses to flashes. . . . 88
5 Cluster-average responses to single-ommatidium flashes. . . . 88
6 Peak responses to moving edges. . . . 89
7 Peak responses to moving edges from task-optimal models. . . . 89
8 Responses to moving edges from task-optimal models. . . . 90
9 Peak responses to moving bars. . . . 90
10 Peak responses to moving bars from task-optimal models. . . . 91
11 Responses to moving bars from task-optimal models. . . . 91
12 Spatio-temporal receptive field. . . . 92
13 Maximally excitatory stimuli. . . . 92
14 Task-constrained parameters. . . . 93

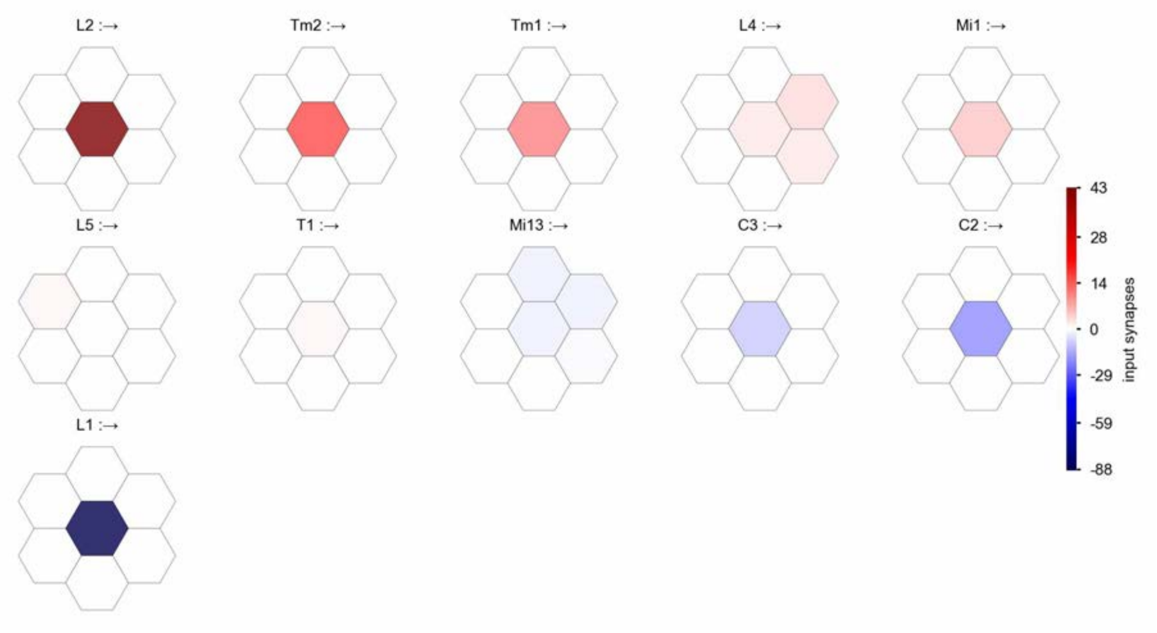

L5 - Figure 1: **Anatomical receptive fields.** Each colored hexagon is an input connection, with the connection strength characterized by the average number of synapses that we count from the EM reconstruction. Red indicates excitatory synapses, blue indicates inhibitory synapses from inferred signs. Filters in the order of their total number of synapses.

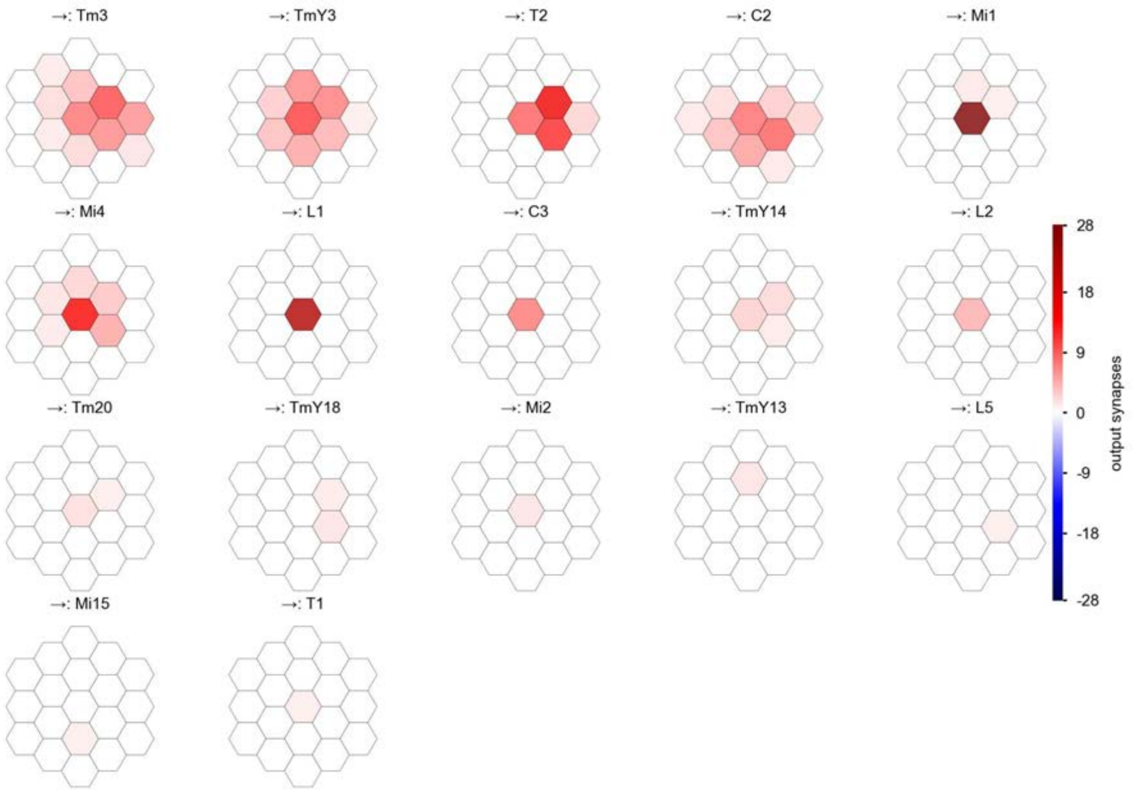

L5 - Figure 2: **Anatomical projective fields.** Each colored hexagon is an output connection, with the connection strength characterized by the average number of synapses that we count from the EM reconstruction. Red indicates excitatory synapses, blue indicates inhibitory synapses from inferred signs. Filters in the order of their total number of synapses.

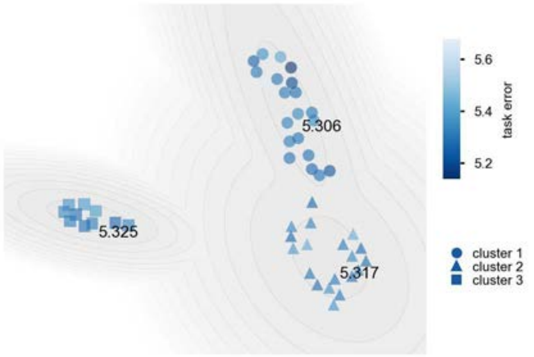

L5 - Figure 3: **Clustering of the responses to naturalistic stimuli.** Clustering of the 50 models based on the cell type responses to naturalistic scenes from the Sintel dataset. Scatterpoints represent individual models colored by their task error.

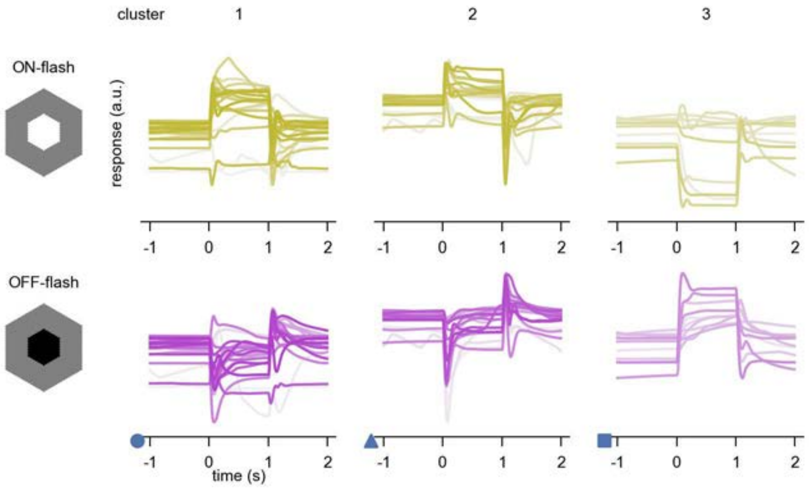

L5 - Figure 4: **Responses to flashes.** The top row shows responses to ON-flashes (yellow), the bottom row shows responses to OFF-flashes (magenta). The responses from the 50 different models that are separated into the different clusters (columns) overlay, with better task-performing models on top. Responses from better task-performing models are more saturated. The circular flashes (1s) cover 6 ommatidia in radius and are presented at time zero. Before and after, a grey-stimulus leads to a stationary state of the network.

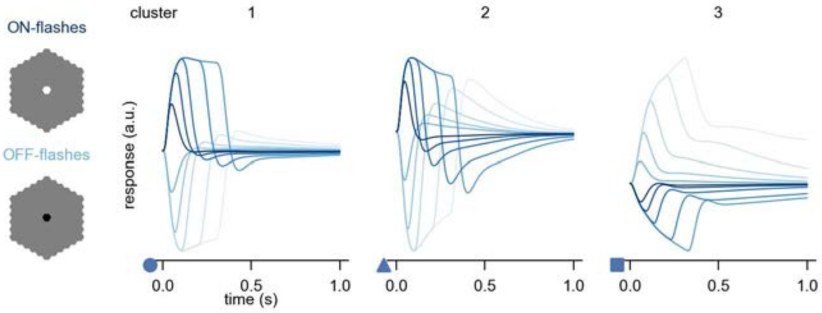

L5 - Figure 5: **Cluster-average responses to single-ommatidium flashes.** Responses to single-ommatidium ON-flashes (dark blue shades) and single-ommatidium OFF-flashes (light blue shades) of 20ms, 50ms, 100ms, 200ms, 300ms duration. The flashes occur at second zero.

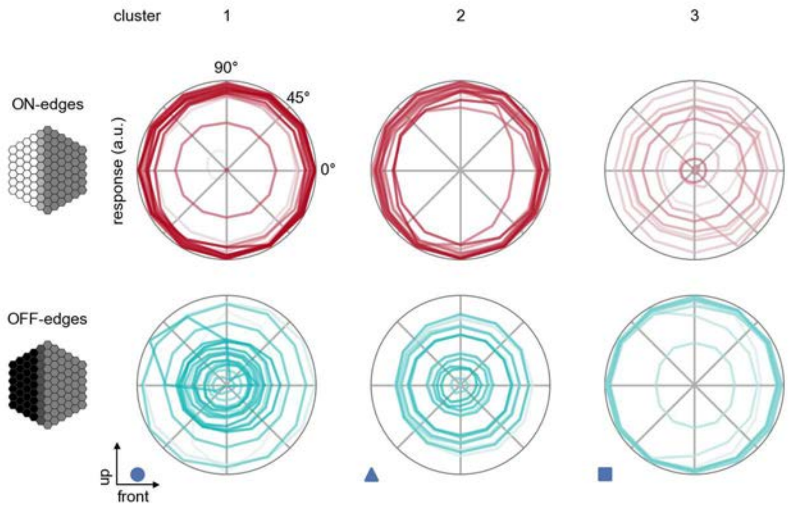

L5 - Figure 6: **Peak responses to moving edges.** The top row shows peak responses to moving ON-edges (red), the bottom row shows peak responses to moving OFF-edges (turqoise). The peak responses are averaged over edge-speeds. Edge-stimuli move in different directions from 0 to 360 degrees. The responses from the different models in the different clusters (columns) overlay. Responses from better task-performing models are more saturated.

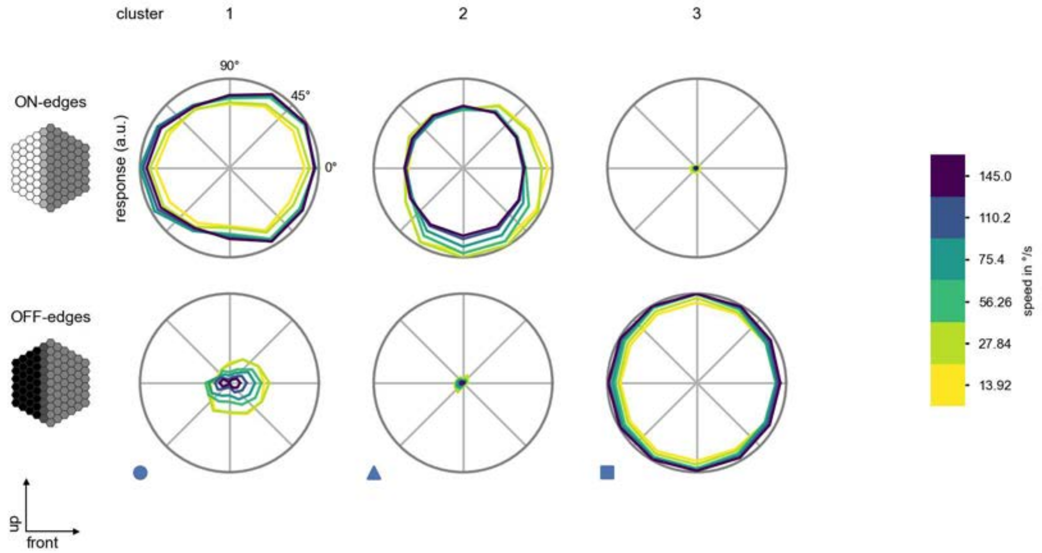

L5 - Figure 7: **Peak responses to moving edges from task-optimal models.** The top row shows peak responses to moving ON-edges, the bottom row shows peak responses to moving OFF-edges of varying speeds from 13.92°/s to 145°/s (yellow to dark blue). The edge-stimuli move in different directions from 0 to 360 degrees and at different speeds. Responses from the task-optimal model in the respective cluster.

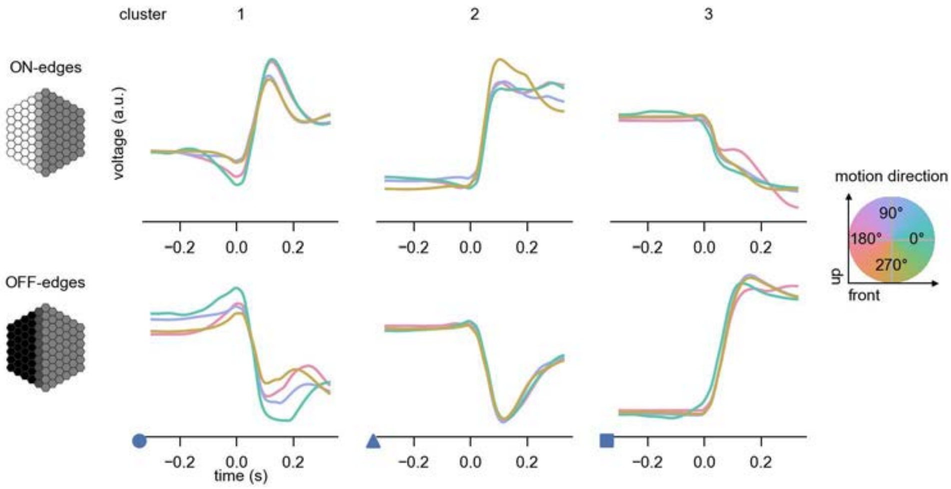

L5 - Figure 8: **Responses to moving edges from task-optimal models.** Responses to moving ON-edges (top row) and to moving OFF-edges (bottom row). Edges move in different directions from 0 to 360 degrees and at different speeds. Responses are from the task-optimal model in the respective cluster. Edges moving at 75.4°/s in all cardinal directions (green 0°, blue 90°, red 180°, yellow 270°) from -22.5 to 22.5° visual angle.

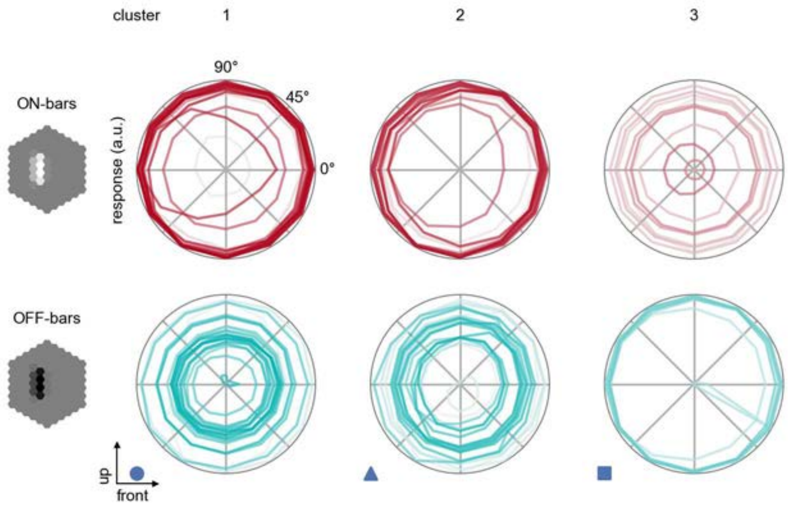

L5 - Figure 9: **Peak responses to moving bars.** The top row shows peak responses to moving ON-bars (red), the bottom row shows peak responses to moving OFF-bars (turqoise). The peak responses are averaged over bar-speeds. Bar-stimuli move in different directions from 0 to 360 degrees. The responses from the different models in the different clusters (columns) overlay. Responses from better task-performing models are more saturated.

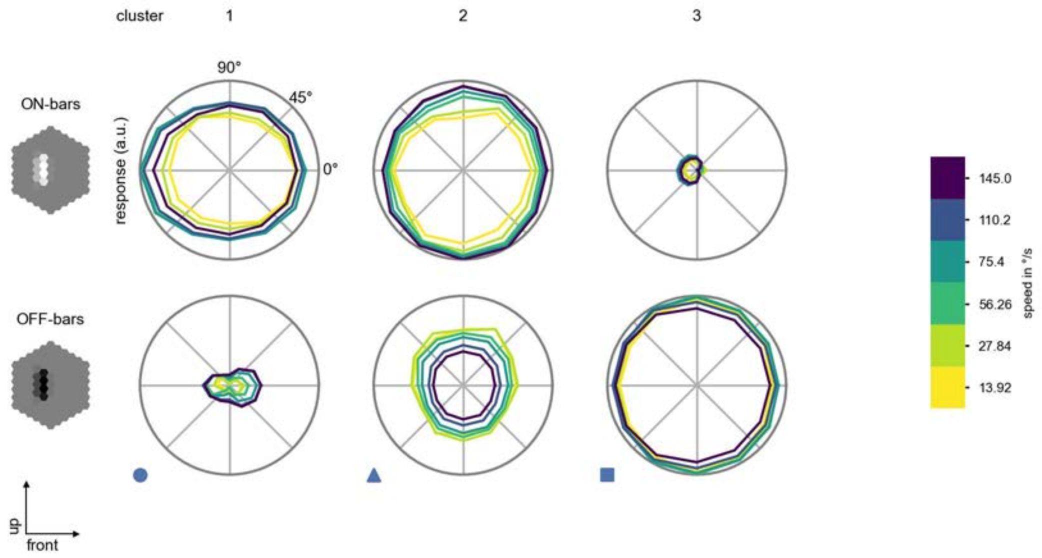

L5 - Figure 10: **Peak responses to moving bars from task-optimal models.** The top row shows peak responses to moving ON-bars, the bottom row shows peak responses to moving OFF-bars of varying speeds from 13.92°/s to 145°/s (yellow to dark blue). The bar-stimuli move in different directions from 0 to 360 degrees and at different speeds. Responses from the task-optimal model in the respective cluster.

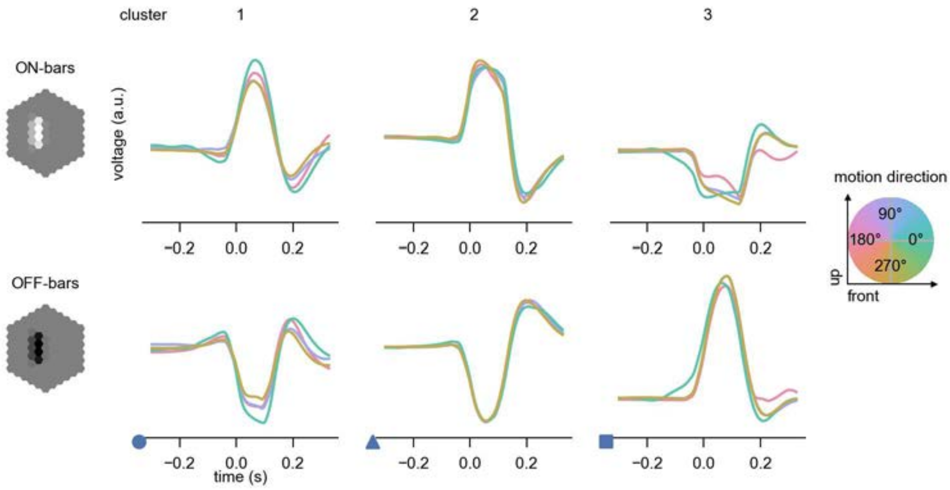

L5 - Figure 11: **Responses to moving bars from task-optimal models.** Responses to moving ON-bars (top row) and to moving OFF-bars (bottom row). Bars move in different directions from 0 to 360 degrees and at different speeds. Responses are from the task-optimal model in the respective cluster. Bars moving at 75.4°/s in all cardinal directions (green 0°, blue 90°, red 180°, yellow 270°) from -22.5 to 22.5° visual angle.

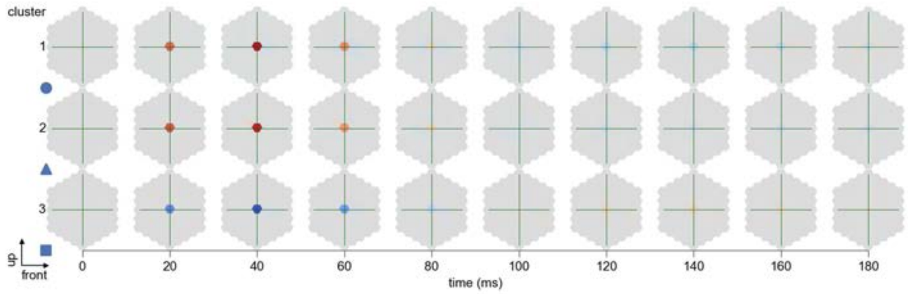

L5 - Figure 12: **Spatio-temporal receptive field.** Responses of the central cell to ON-impulses (5 ms) at single-ommatidium flash locations. The flash occurs at second zero. Responses from the task-optimal model of the respective cluster (rows). Red indicates depolarization, blue indicates hyperpolarization.

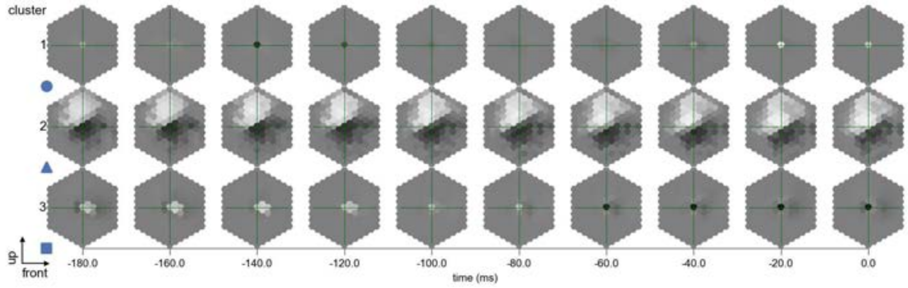

L5 - Figure 13: **Maximally excitatory stimuli.** Each row presents the regularized naturalistic-stimulus from the Sintel dataset that maximizes the cell type's central column response at second zero in the task-optimal model of the respective cluster (rows).

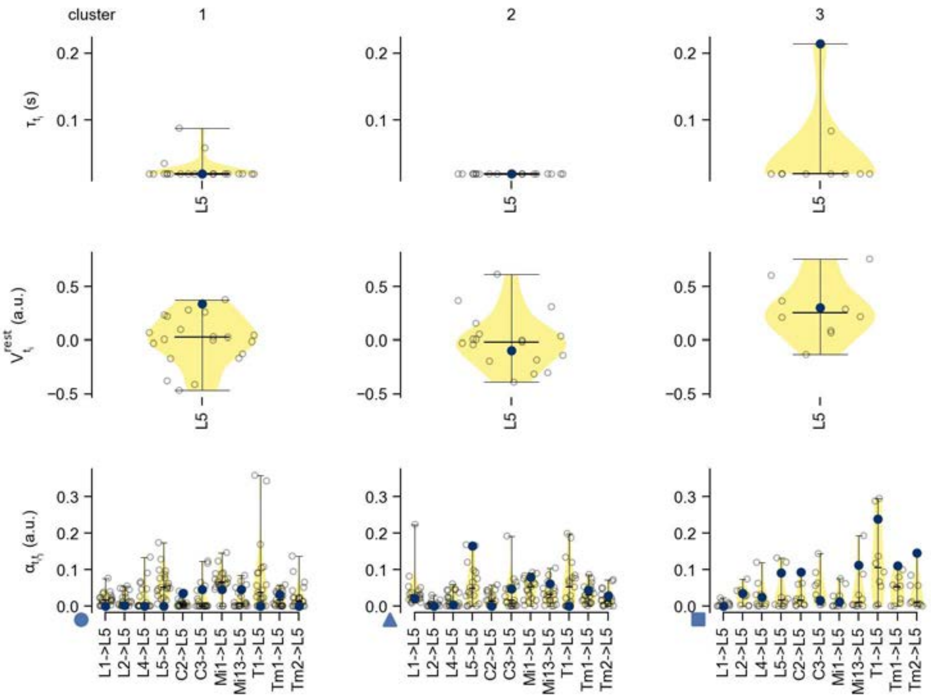

L5 - Figure 14: **Task-constrained parameters.** Each column shows the parameters inferred within the respective cluster. First row: learned time constants of the cell type. Second row: resting potentials of the cell type. Third row: scaling factors for the convolutional filters. The blue scatter represents the parameters from the task-optimal model within the cluster.

14 Lawf1

← Cell types

Figures

1 Anatomical receptive fields. . . . 94
2 Anatomical projective fields. . . . 94
3 Clustering of the responses to naturalistic stimuli. . . . 95
4 Responses to flashes. . . . 95
5 Cluster-average responses to single-ommatidium flashes. . . . 95
6 Peak responses to moving edges. . . . 96
7 Peak responses to moving edges from task-optimal models. . . . 96
8 Responses to moving edges from task-optimal models. . . . 97
9 Peak responses to moving bars. . . . 97
10 Peak responses to moving bars from task-optimal models. . . . 98
11 Responses to moving bars from task-optimal models. . . . 98
12 Spatio-temporal receptive field. . . . 99
13 Maximally excitatory stimuli. . . . 99
14 Task-constrained parameters. . . . 100

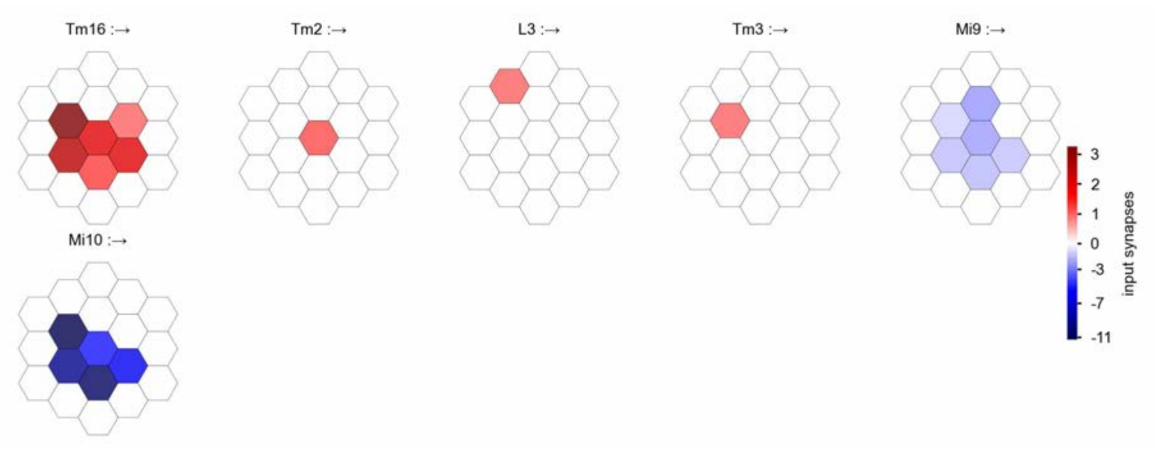

Lawf1 - Figure 1: **Anatomical receptive fields.** Each colored hexagon is an input connection, with the connection strength characterized by the average number of synapses that we count from the EM reconstruction. Red indicates excitatory synapses, blue indicates inhibitory synapses from inferred signs. Filters in the order of their total number of synapses.

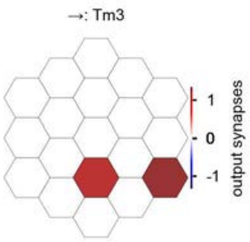

Lawf1 - Figure 2: **Anatomical projective fields.** Each colored hexagon is an output connection, with the connection strength characterized by the average number of synapses that we count from the EM reconstruction. Red indicates excitatory synapses, blue indicates inhibitory synapses from inferred signs. Filters in the order of their total number of synapses.

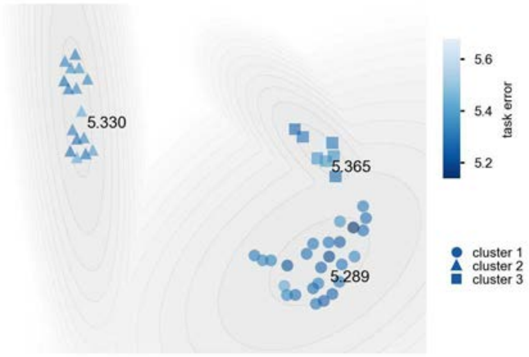

Lawf1 - Figure 3: **Clustering of the responses to naturalistic stimuli.** Clustering of the 50 models based on the cell type responses to naturalistic scenes from the Sintel dataset. Scatterpoints represent individual models colored by their task error.

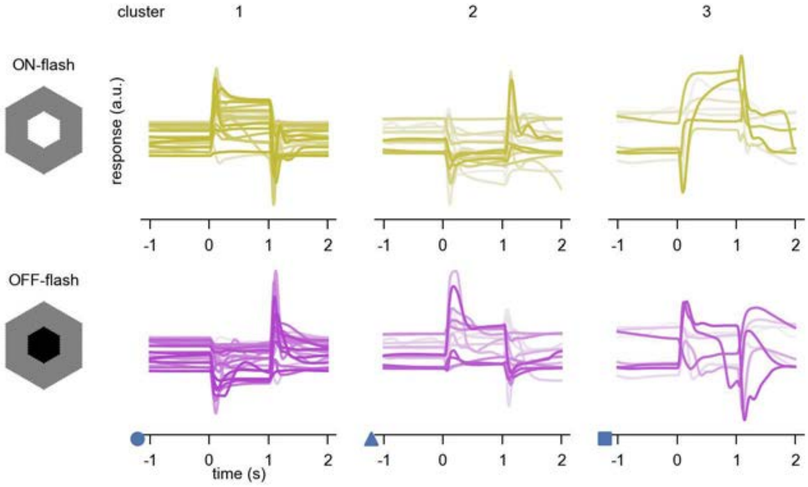

Lawf1 - Figure 4: **Responses to flashes.** The top row shows responses to ON-flashes (yellow), the bottom row shows responses to OFF-flashes (magenta). The responses from the 50 different models that are separated into the different clusters (columns) overlay, with better task-performing models on top. Responses from better task-performing models are more saturated. The circular flashes (1s) cover 6 ommatidia in radius and are presented at time zero. Before and after, a grey-stimulus leads to a stationary state of the network.

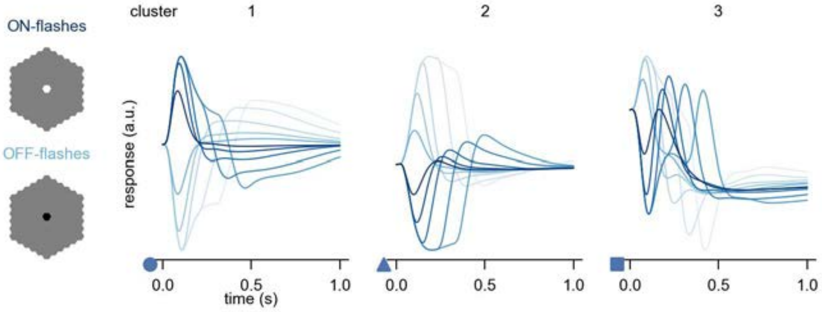

Lawf1 - Figure 5: **Cluster-average responses to single-ommatidium flashes.** Responses to single-ommatidium ON-flashes (dark blue shades) and single-ommatidium OFF-flashes (light blue shades) of 20ms, 50ms, 100ms, 200ms, 300ms duration. The flashes occur at second zero.

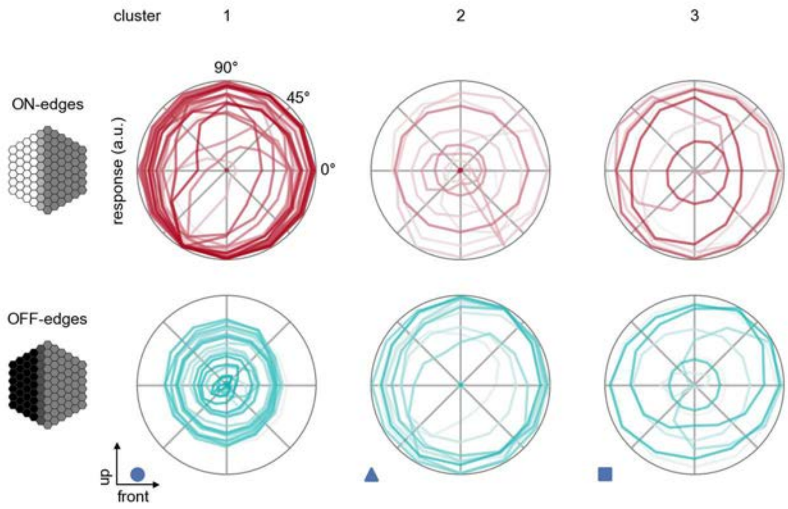

Lawf1 - Figure 6: **Peak responses to moving edges.** The top row shows peak responses to moving ON-edges (red), the bottom row shows peak responses to moving OFF-edges (turqoise). The peak responses are averaged over edge-speeds. Edge-stimuli move in different directions from 0 to 360 degrees. The responses from the different models in the different clusters (columns) overlay. Responses from better task-performing models are more saturated.

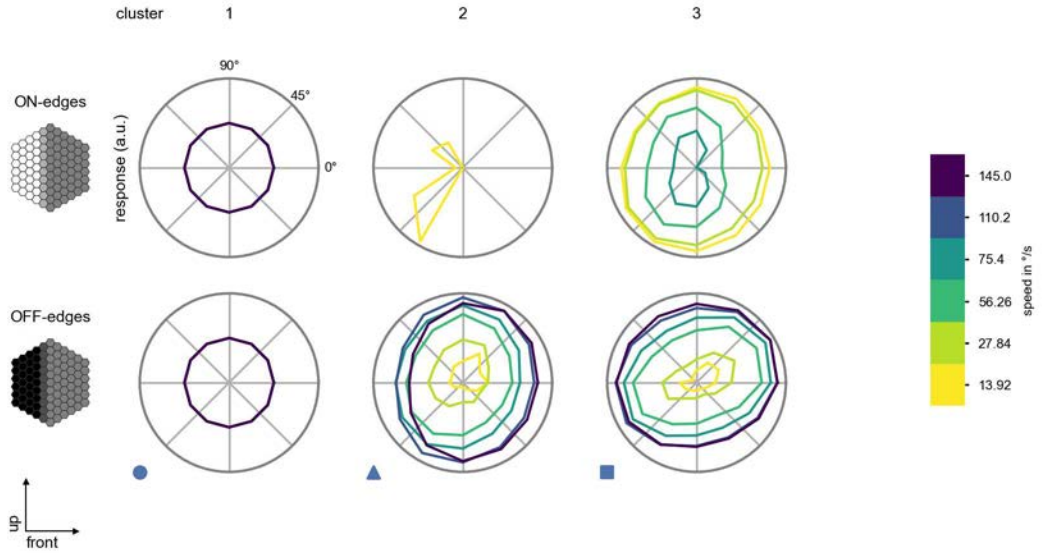

Lawf1 - Figure 7: **Peak responses to moving edges from task-optimal models.** The top row shows peak responses to moving ON-edges, the bottom row shows peak responses to moving OFF-edges of varying speeds from 13.92°/s to 145°/s (yellow to dark blue). The edge-stimuli move in different directions from 0 to 360 degrees and at different speeds. Responses from the task-optimal model in the respective cluster.

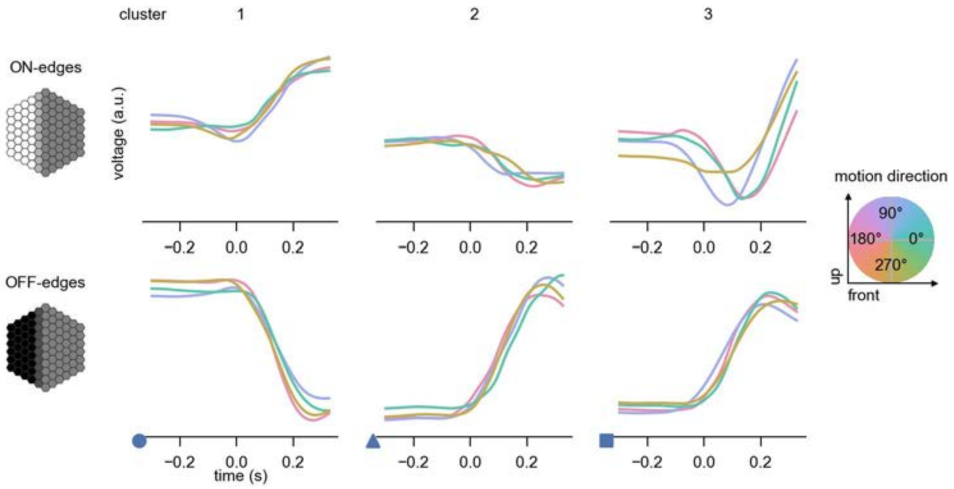

Lawf1 - Figure 8: **Responses to moving edges from task-optimal models.** Responses to moving ON-edges (top row) and to moving OFF-edges (bottom row). Edges move in different directions from 0 to 360 degrees and at different speeds. Responses are from the task-optimal model in the respective cluster. Edges moving at 75.4°/s in all cardinal directions (green 0°, blue 90°, red 180°, yellow 270°) from -22.5 to 22.5° visual angle.

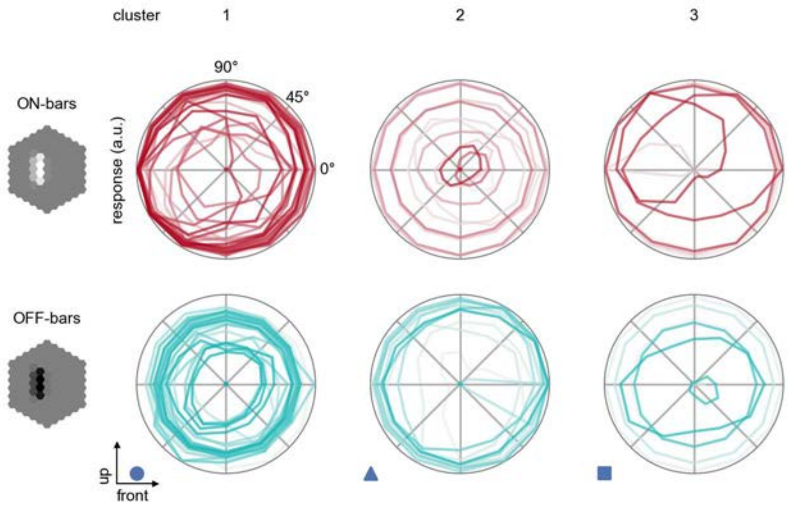

Lawf1 - Figure 9: **Peak responses to moving bars.** The top row shows peak responses to moving ON-bars (red), the bottom row shows peak responses to moving OFF-bars (turqoise). The peak responses are averaged over bar-speeds. Bar-stimuli move in different directions from 0 to 360 degrees. The responses from the different models in the different clusters (columns) overlay. Responses from better task-performing models are more saturated.

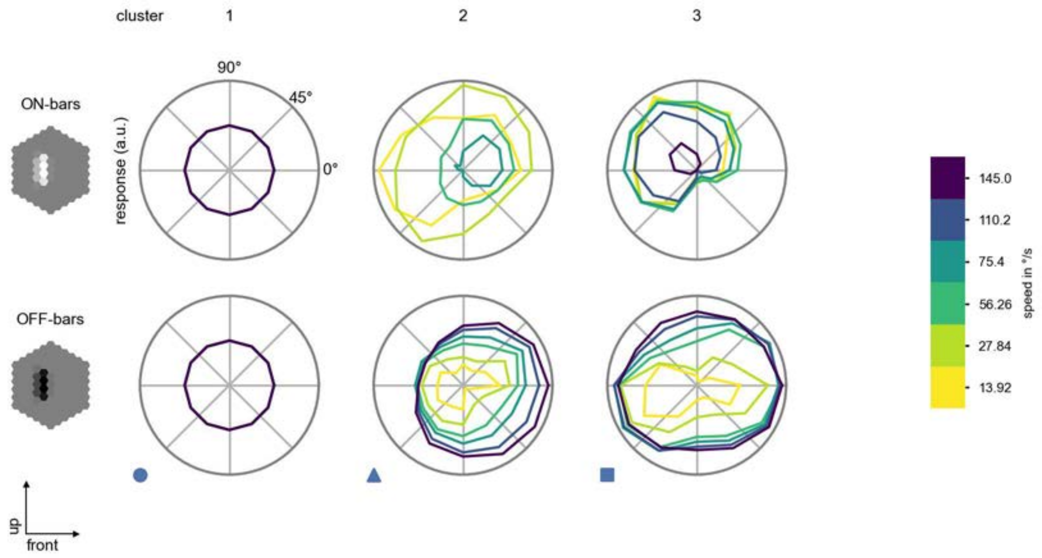

Lawf1 - Figure 10: **Peak responses to moving bars from task-optimal models.** The top row shows peak responses to moving ON-bars, the bottom row shows peak responses to moving OFF-bars of varying speeds from 13.92°/s to 145°/s (yellow to dark blue). The bar-stimuli move in different directions from 0 to 360 degrees and at different speeds. Responses from the task-optimal model in the respective cluster.

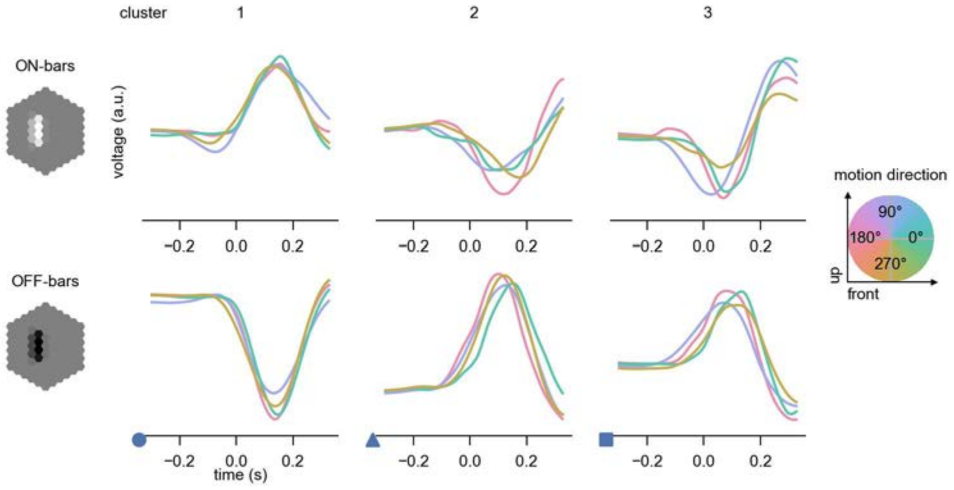

Lawf1 - Figure 11: **Responses to moving bars from task-optimal models.** Responses to moving ON-bars (top row) and to moving OFF-bars (bottom row). Bars move in different directions from 0 to 360 degrees and at different speeds. Responses are from the task-optimal model in the respective cluster. Bars moving at 75.4°/s in all cardinal directions (green 0°, blue 90°, red 180°, yellow 270°) from -22.5 to 22.5° visual angle.

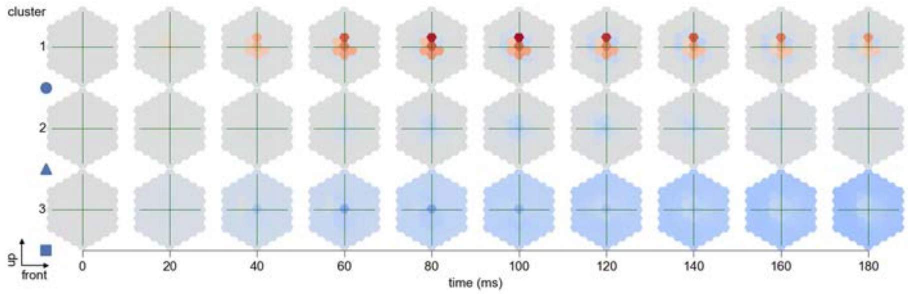

Lawf1 - Figure 12: **Spatio-temporal receptive field.** Responses of the central cell to ON-impulses (5 ms) at single-ommatidium flash locations. The flash occurs at second zero. Responses from the task-optimal model of the respective cluster (rows). Red indicates depolarization, blue indicates hyperpolarization.

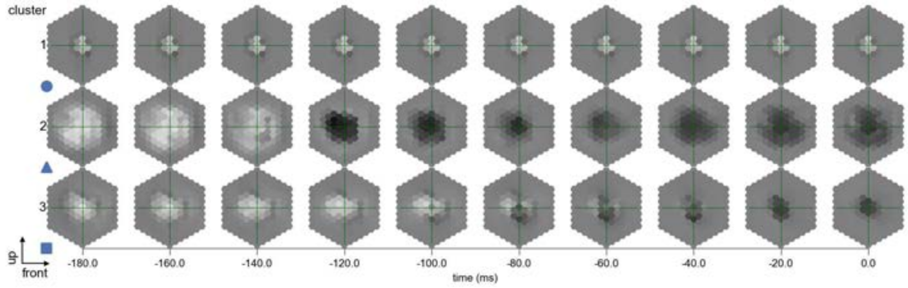

Lawf1 - Figure 13: **Maximally excitatory stimuli.** Each row presents the regularized naturalistic-stimulus from the Sintel dataset that maximizes the cell type's central column response at second zero in the task-optimal model of the respective cluster (rows).

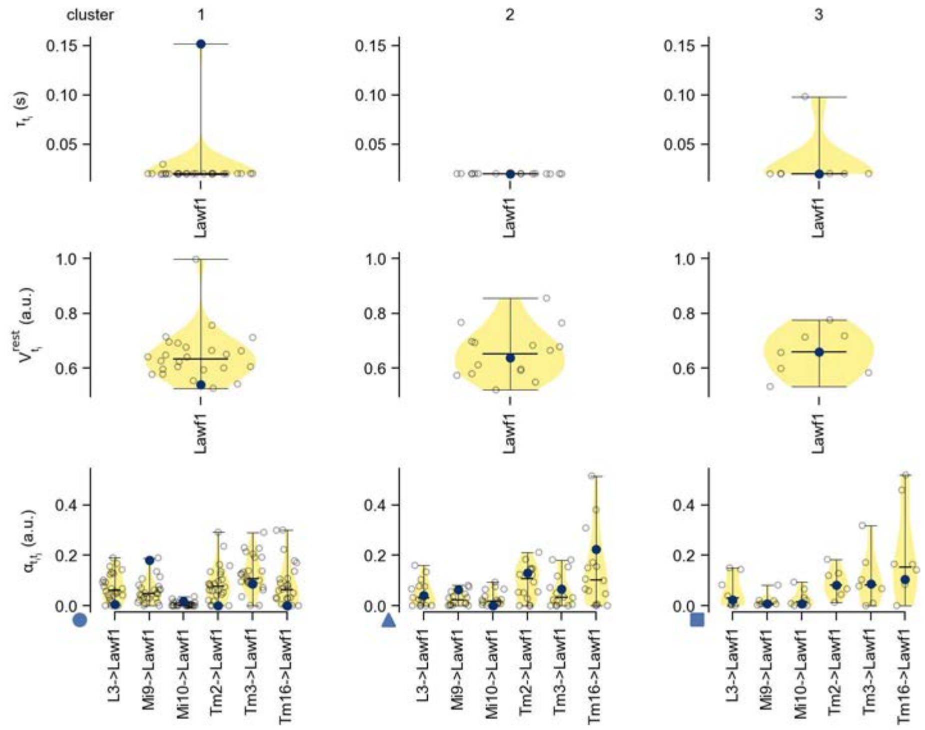

Lawf1 - Figure 14: **Task-constrained parameters.** Each column shows the parameters inferred within the respective cluster. First row: learned time constants of the cell type. Second row: resting potentials of the cell type. Third row: scaling factors for the convolutional filters. The blue scatter represents the parameters from the task-optimal model within the cluster.

15 Lawf2

← Cell types

Figures

1 Anatomical receptive fields. 101
2 Anatomical projective fields. 101
3 Clustering of the responses to naturalistic stimuli. 102
4 Responses to flashes. 102
5 Cluster-average responses to single-ommatidium flashes. 102
6 Peak responses to moving edges. 103
7 Peak responses to moving edges from task-optimal models. 103
8 Responses to moving edges from task-optimal models. 104
9 Peak responses to moving bars. 104
10 Peak responses to moving bars from task-optimal models. 105
11 Responses to moving bars from task-optimal models. 105
12 Spatio-temporal receptive field. 106
13 Maximally excitatory stimuli. 106
14 Task-constrained parameters. 107

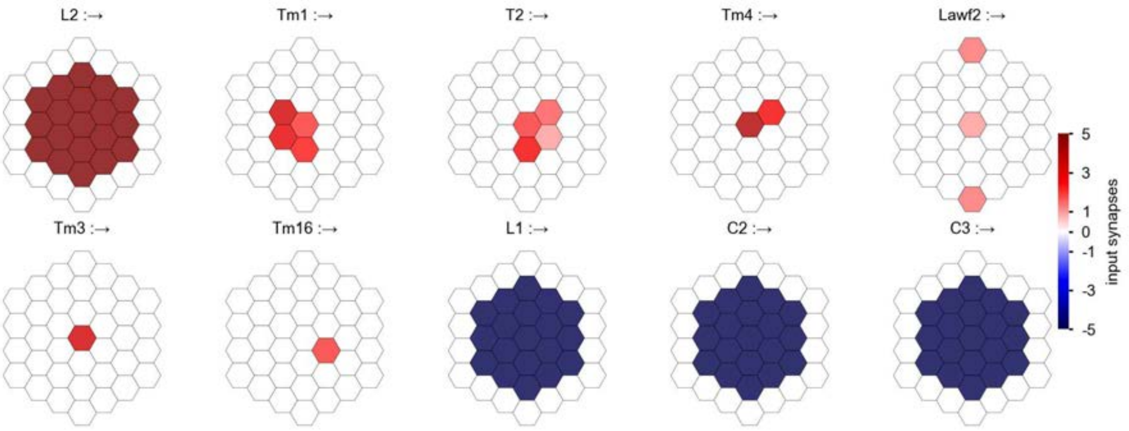

Lawf2 - Figure 1: **Anatomical receptive fields.** Each colored hexagon is an input connection, with the connection strength characterized by the average number of synapses that we count from the EM reconstruction. Red indicates excitatory synapses, blue indicates inhibitory synapses from inferred signs. Filters in the order of their total number of synapses.

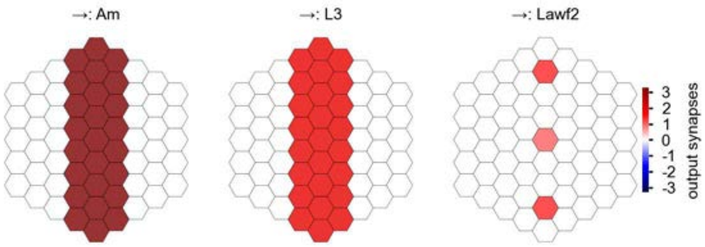

Lawf2 - Figure 2: **Anatomical projective fields.** Each colored hexagon is an output connection, with the connection strength characterized by the average number of synapses that we count from the EM reconstruction. Red indicates excitatory synapses, blue indicates inhibitory synapses from inferred signs. Filters in the order of their total number of synapses.

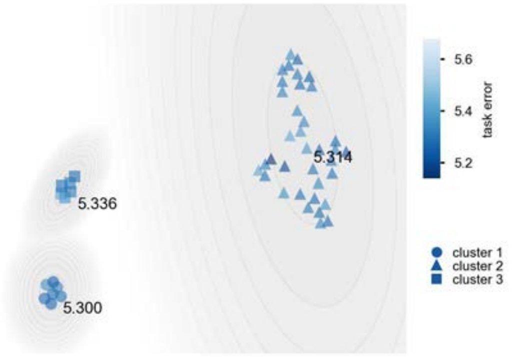

Lawf2 - Figure 3: **Clustering of the responses to naturalistic stimuli.** Clustering of the 50 models based on the cell type responses to naturalistic scenes from the Sintel dataset. Scatterpoints represent individual models colored by their task error.

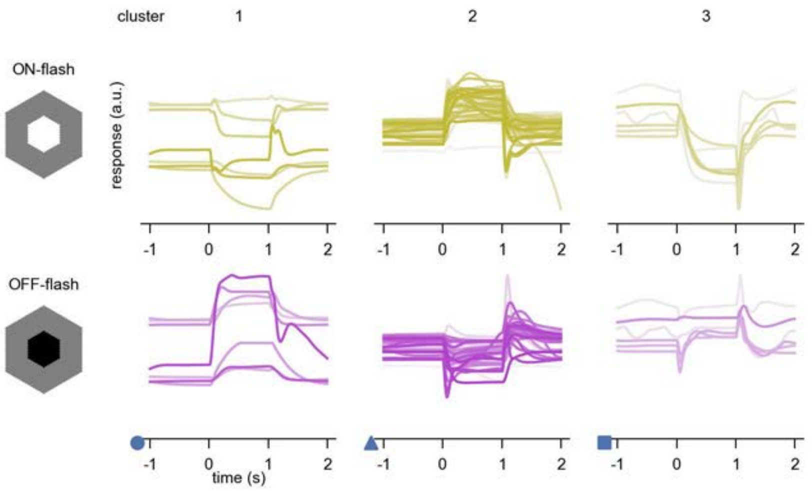

Lawf2 - Figure 4: **Responses to flashes.** The top row shows responses to ON-flashes (yellow), the bottom row shows responses to OFF-flashes (magenta). The responses from the 50 different models that are separated into the different clusters (columns) overlay, with better task-performing models on top. Responses from better task-performing models are more saturated. The circular flashes (1s) cover 6 ommatidia in radius and are presented at time zero. Before and after, a grey-stimulus leads to a stationary state of the network.

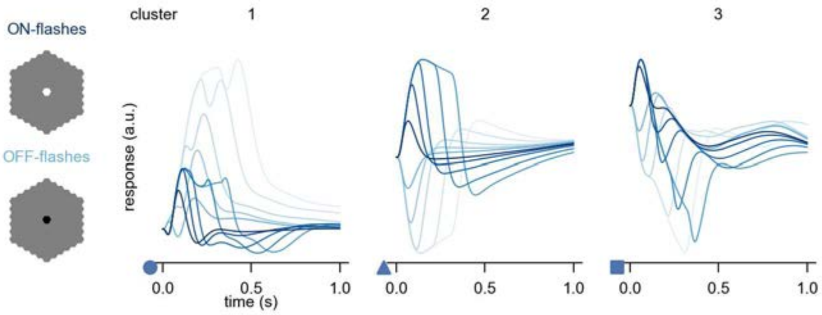

Lawf2 - Figure 5: **Cluster-average responses to single-ommatidium flashes.** Responses to single-ommatidium ON-flashes (dark blue shades) and single-ommatidium OFF-flashes (light blue shades) of 20ms, 50ms, 100ms, 200ms, 300ms duration. The flashes occur at second zero.

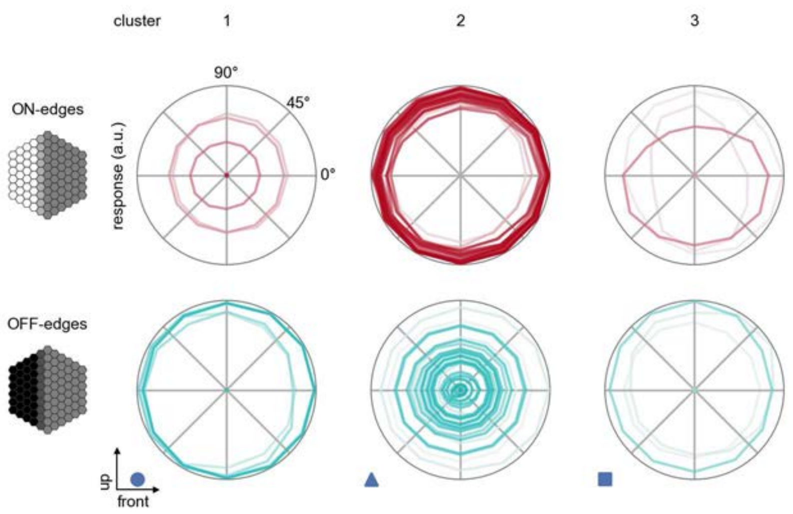

Lawf2 - Figure 6: **Peak responses to moving edges.** The top row shows peak responses to moving ON-edges (red), the bottom row shows peak responses to moving OFF-edges (turqoise). The peak responses are averaged over edge-speeds. Edge-stimuli move in different directions from 0 to 360 degrees. The responses from the different models in the different clusters (columns) overlay. Responses from better task-performing models are more saturated.

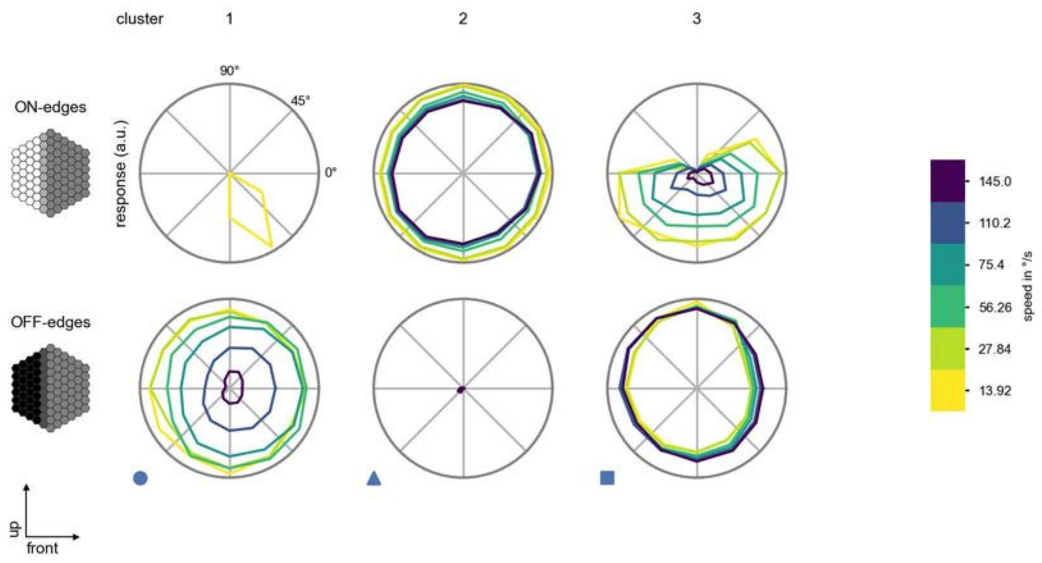

Lawf2 - Figure 7: **Peak responses to moving edges from task-optimal models.** The top row shows peak responses to moving ON-edges, the bottom row shows peak responses to moving OFF-edges of varying speeds from 13.92°/s to 145°/s (yellow to dark blue). The edge-stimuli move in different directions from 0 to 360 degrees and at different speeds. Responses from the task-optimal model in the respective cluster.

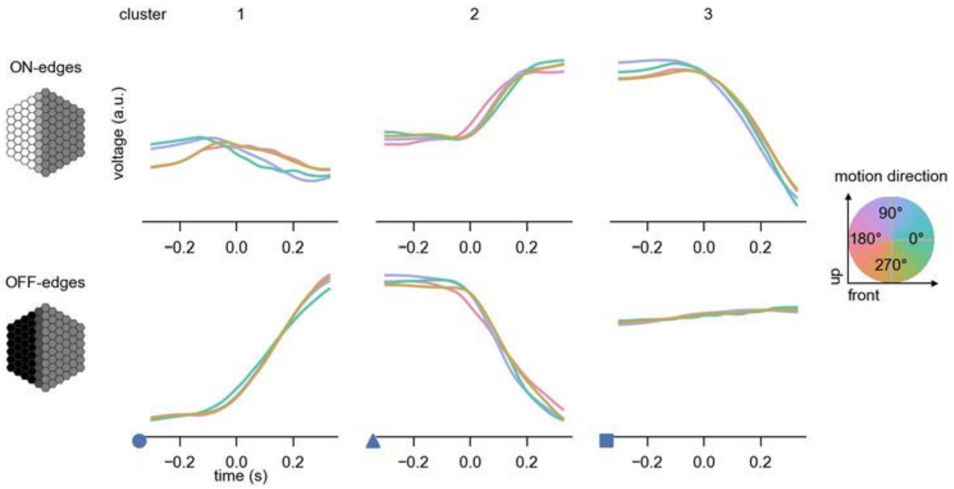

Lawf2 - Figure 8: **Responses to moving edges from task-optimal models.** Responses to moving ON-edges (top row) and to moving OFF-edges (bottom row). Edges move in different directions from 0 to 360 degrees and at different speeds. Responses are from the task-optimal model in the respective cluster. Edges moving at 75.4°/s in all cardinal directions (green 0°, blue 90°, red 180°, yellow 270°) from -22.5 to 22.5° visual angle.

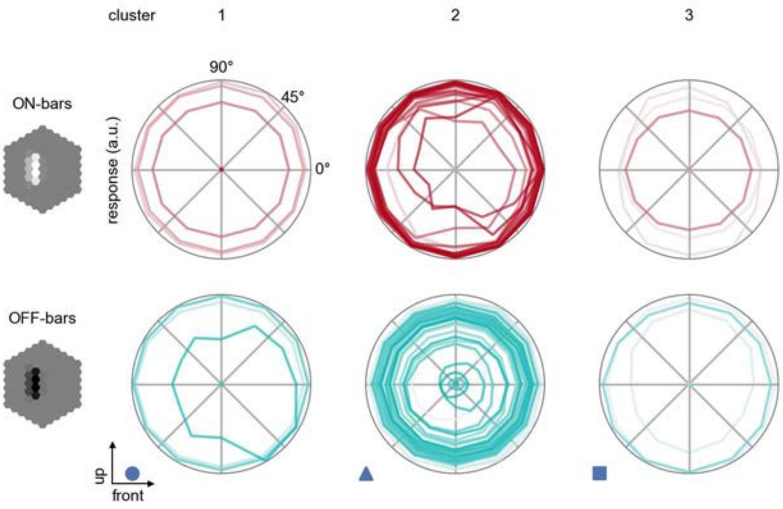

Lawf2 - Figure 9: **Peak responses to moving bars.** The top row shows peak responses to moving ON-bars (red), the bottom row shows peak responses to moving OFF-bars (turqoise). The peak responses are averaged over bar-speeds. Bar-stimuli move in different directions from 0 to 360 degrees. The responses from the different models in the different clusters (columns) overlay. Responses from better task-performing models are more saturated.

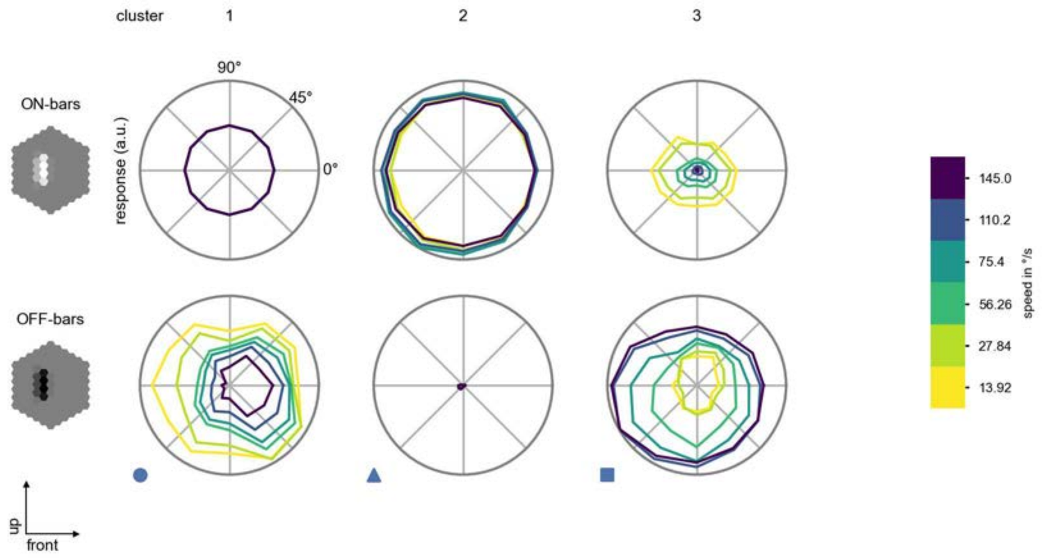

Lawf2 - Figure 10: **Peak responses to moving bars from task-optimal models.** The top row shows peak responses to moving ON-bars, the bottom row shows peak responses to moving OFF-bars of varying speeds from 13.92°/s to 145°/s (yellow to dark blue). The bar-stimuli move in different directions from 0 to 360 degrees and at different speeds. Responses from the task-optimal model in the respective cluster.

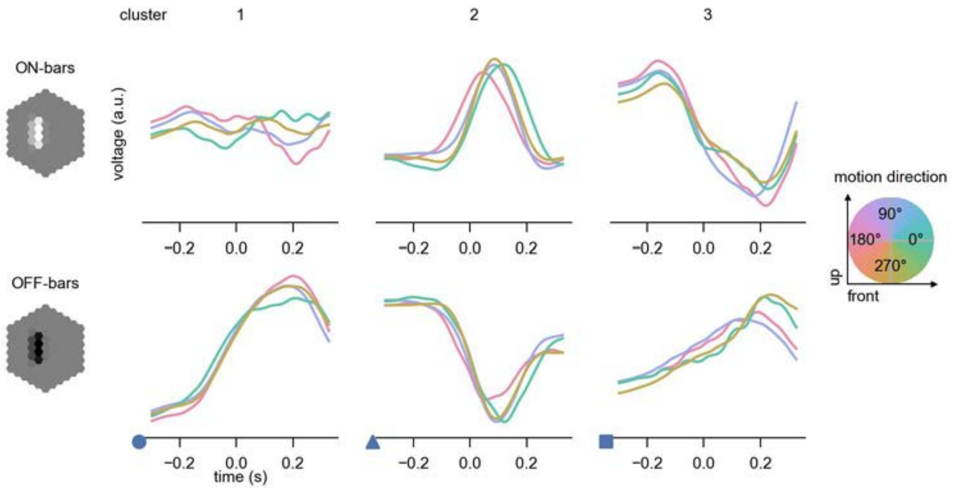

Lawf2 - Figure 11: **Responses to moving bars from task-optimal models.** Responses to moving ON-bars (top row) and to moving OFF-bars (bottom row). Bars move in different directions from 0 to 360 degrees and at different speeds. Responses are from the task-optimal model in the respective cluster. Bars moving at 75.4°/s in all cardinal directions (green 0°, blue 90°, red 180°, yellow 270°) from -22.5 to 22.5° visual angle.

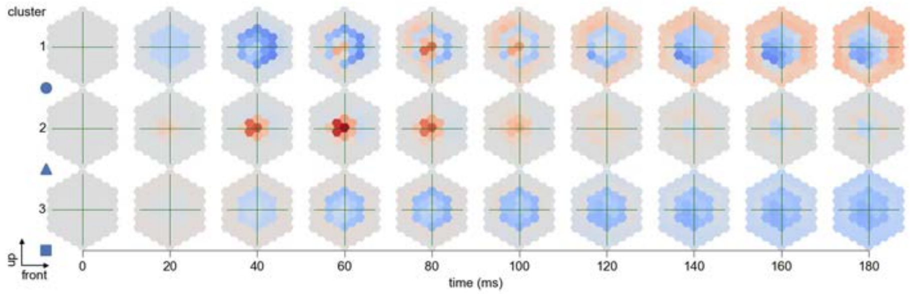

Lawf2 - Figure 12: **Spatio-temporal receptive field.** Responses of the central cell to ON-impulses (5 ms) at single-ommatidium flash locations. The flash occurs at second zero. Responses from the task-optimal model of the respective cluster (rows). Red indicates depolarization, blue indicates hyperpolarization.

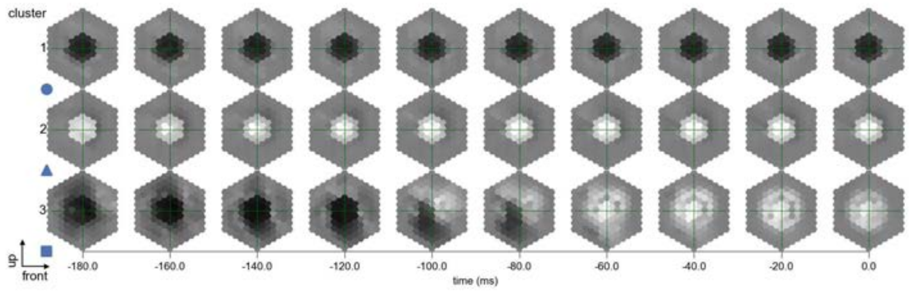

Lawf2 - Figure 13: **Maximally excitatory stimuli.** Each row presents the regularized naturalistic-stimulus from the Sintel dataset that maximizes the cell type's central column response at second zero in the task-optimal model of the respective cluster (rows).

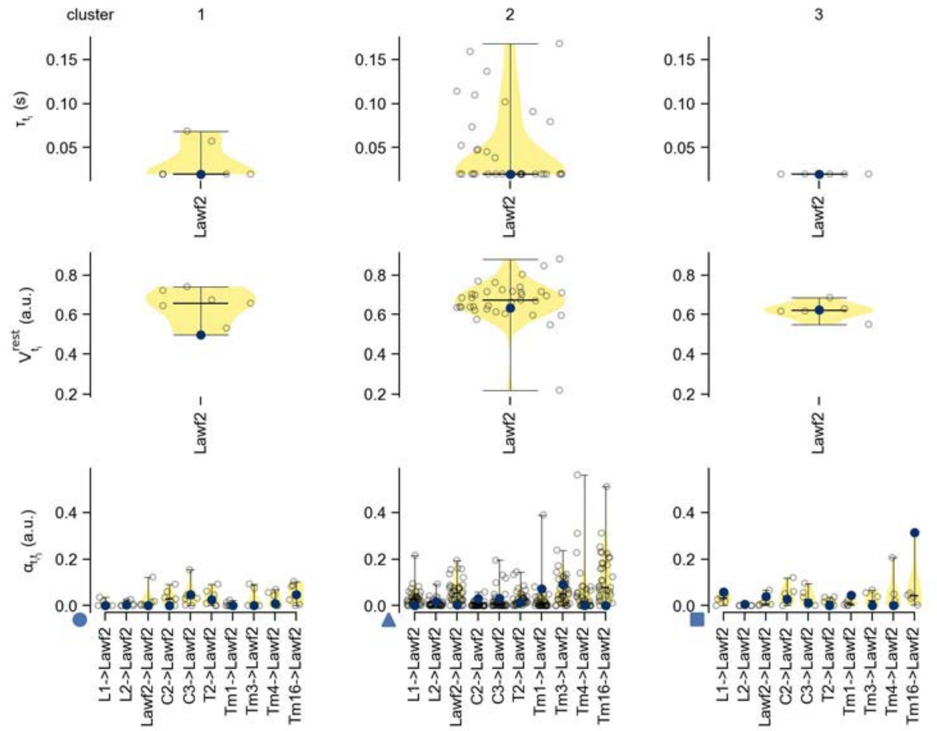

Lawf2 - Figure 14: **Task-constrained parameters.** Each column shows the parameters inferred within the respective cluster. First row: learned time constants of the cell type. Second row: resting potentials of the cell type. Third row: scaling factors for the convolutional filters. The blue scatter represents the parameters from the task-optimal model within the cluster.

16 Am

← Cell types

Figures

1 Anatomical receptive fields. . . . 108
2 Anatomical projective fields. . . . 109
3 Clustering of the responses to naturalistic stimuli. . . . 109
4 Responses to flashes. . . . 110
5 Cluster-average responses to single-ommatidium flashes. . . . 110
6 Peak responses to moving edges. . . . 111
7 Peak responses to moving edges from task-optimal models. . . . 111
8 Responses to moving edges from task-optimal models. . . . 112
9 Peak responses to moving bars. . . . 112
10 Peak responses to moving bars from task-optimal models. . . . 113
11 Responses to moving bars from task-optimal models. . . . 113
12 Spatio-temporal receptive field. . . . 114
13 Maximally excitatory stimuli. . . . 114
14 Task-constrained parameters. . . . 115

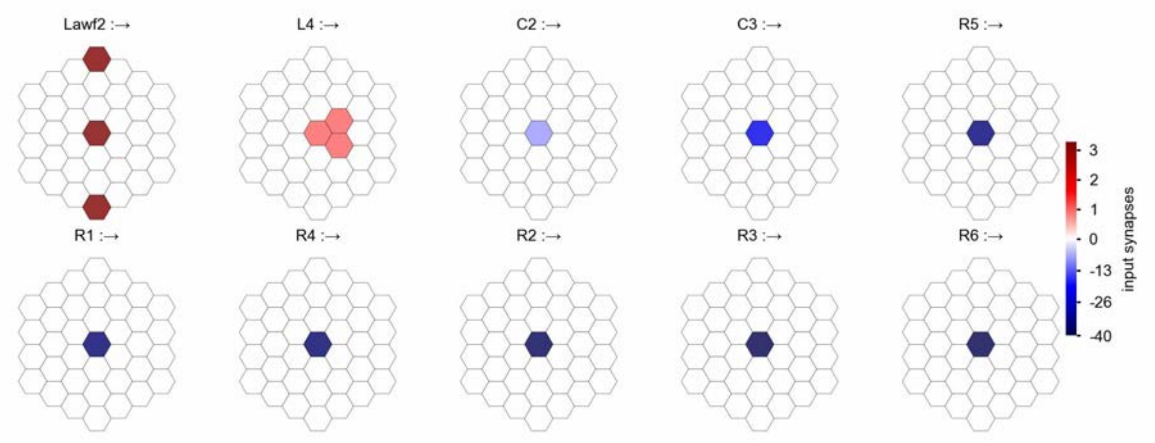

Am - Figure 1: **Anatomical receptive fields.** Each colored hexagon is an input connection, with the connection strength characterized by the average number of synapses that we count from the EM reconstruction. Red indicates excitatory synapses, blue indicates inhibitory synapses from inferred signs. Filters in the order of their total number of synapses.

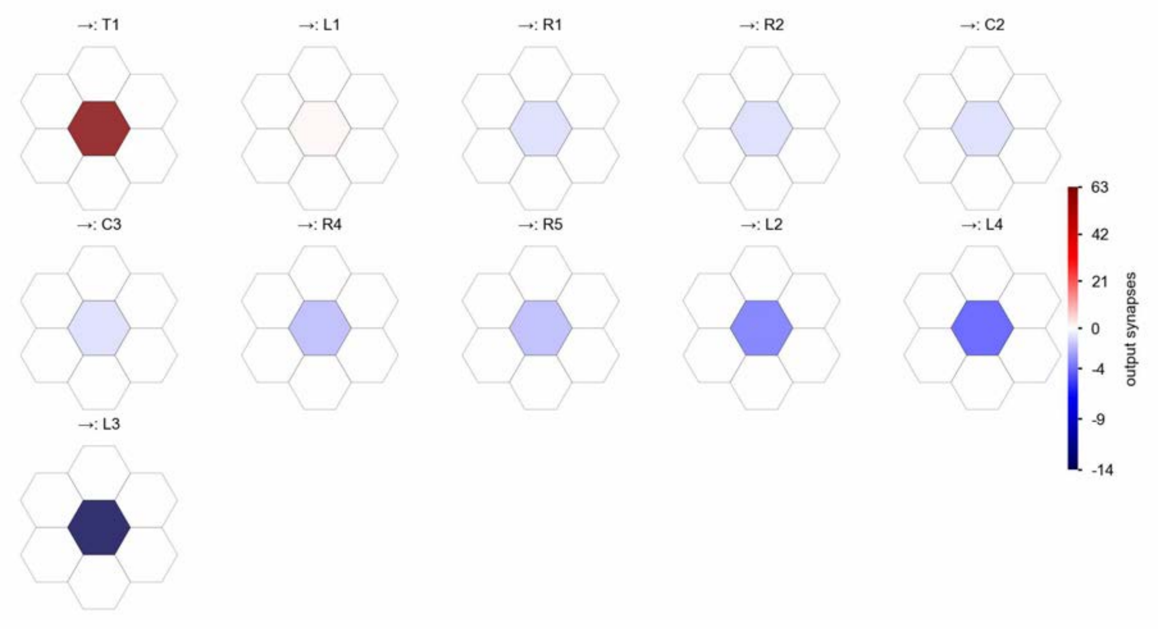

Am - Figure 2: **Anatomical projective fields.** Each colored hexagon is an output connection, with the connection strength characterized by the average number of synapses that we count from the EM reconstruction. Red indicates excitatory synapses, blue indicates inhibitory synapses from inferred signs. Filters in the order of their total number of synapses.

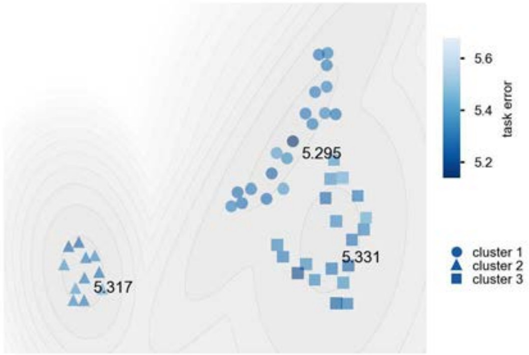

Am - Figure 3: **Clustering of the responses to naturalistic stimuli.** Clustering of the 50 models based on the cell type responses to naturalistic scenes from the Sintel dataset. Scatterpoints represent individual models colored by their task error.

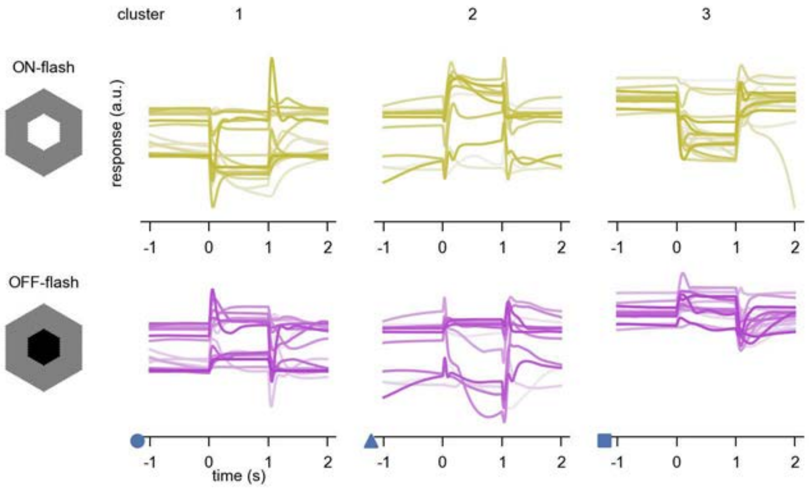

Am - Figure 4: **Responses to flashes.** The top row shows responses to ON-flashes (yellow), the bottom row shows responses to OFF-flashes (magenta). The responses from the 50 different models that are separated into the different clusters (columns) overlay, with better task-performing models on top. Responses from better task-performing models are more saturated. The circular flashes (1s) cover 6 ommatidia in radius and are presented at time zero. Before and after, a grey-stimulus leads to a stationary state of the network.

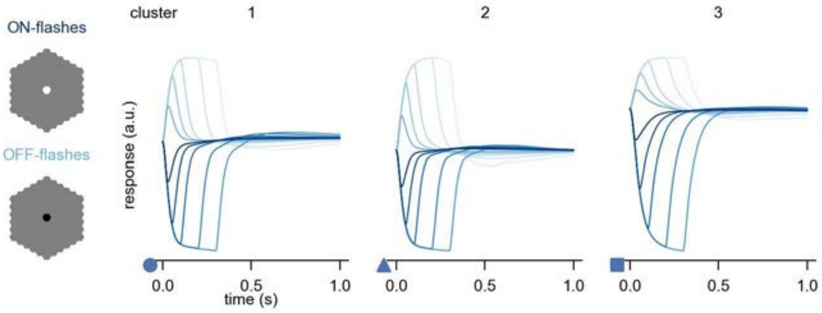

Am - Figure 5: **Cluster-average responses to single-ommatidium flashes.** Responses to single-ommatidium ON-flashes (dark blue shades) and single-ommatidium OFF-flashes (light blue shades) of 20ms, 50ms, 100ms, 200ms, 300ms duration. The flashes occur at second zero.

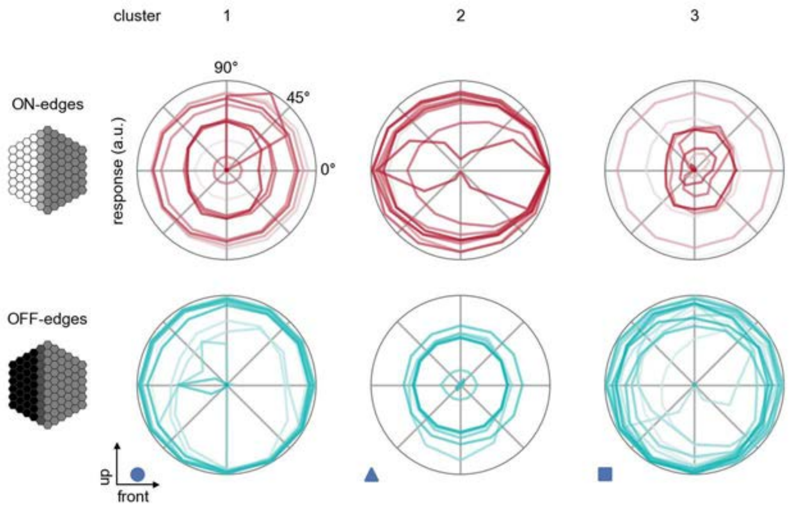

Am - Figure 6: **Peak responses to moving edges.** The top row shows peak responses to moving ON-edges (red), the bottom row shows peak responses to moving OFF-edges (turqoise). The peak responses are averaged over edge-speeds. Edge-stimuli move in different directions from 0 to 360 degrees. The responses from the different models in the different clusters (columns) overlay. Responses from better task-performing models are more saturated.

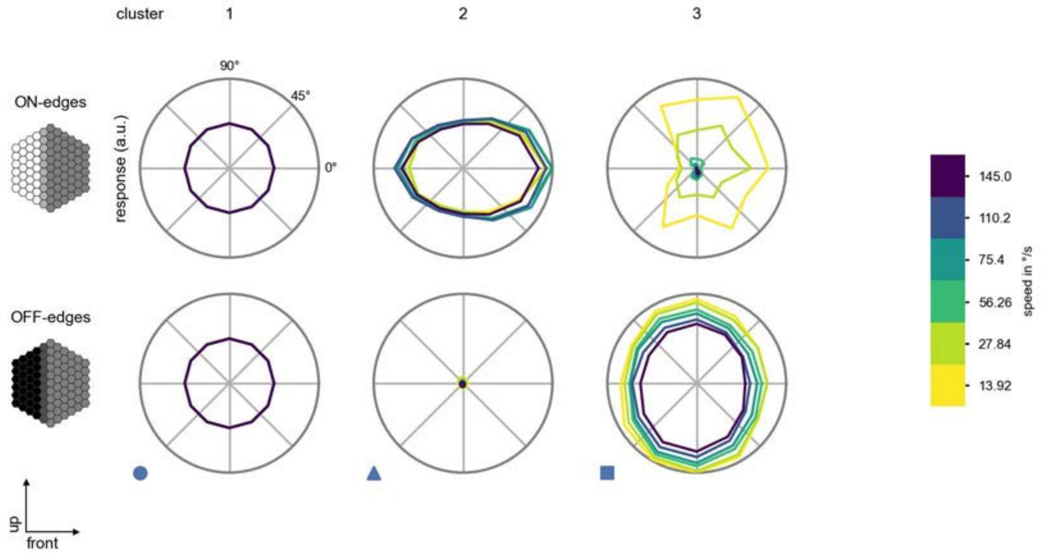

Am - Figure 7: **Peak responses to moving edges from task-optimal models.** The top row shows peak responses to moving ON-edges, the bottom row shows peak responses to moving OFF-edges of varying speeds from 13.92°/s to 145°/s (yellow to dark blue). The edge-stimuli move in different directions from 0 to 360 degrees and at different speeds. Responses from the task-optimal model in the respective cluster.

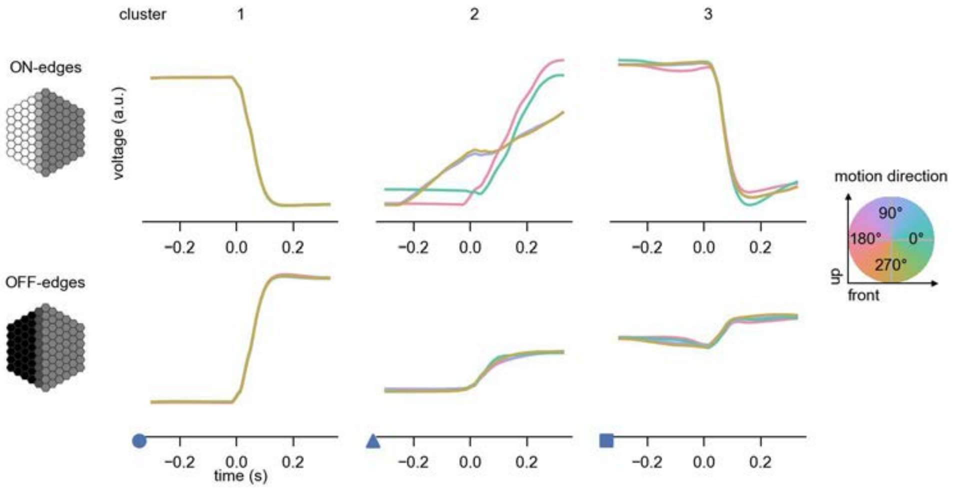

Am - Figure 8: **Responses to moving edges from task-optimal models.** Responses to moving ON-edges (top row) and to moving OFF-edges (bottom row). Edges move in different directions from 0 to 360 degrees and at different speeds. Responses are from the task-optimal model in the respective cluster. Edges moving at 75.4°/s in all cardinal directions (green 0°, blue 90°, red 180°, yellow 270°) from -22.5 to 22.5° visual angle.

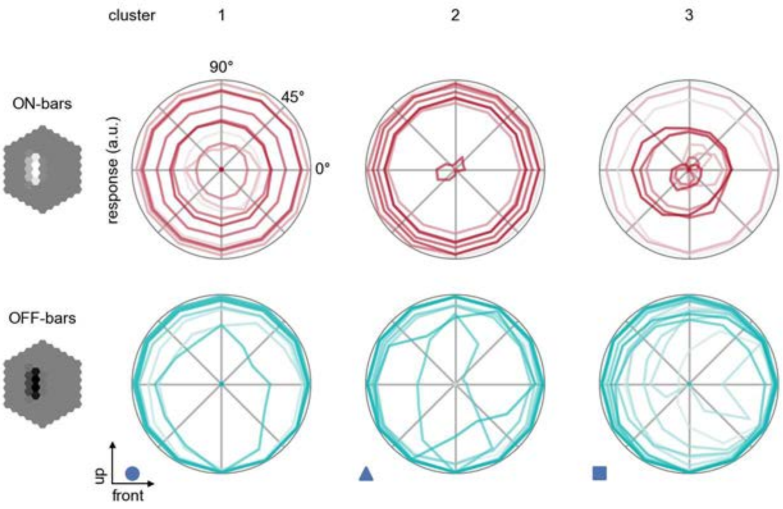

Am - Figure 9: **Peak responses to moving bars.** The top row shows peak responses to moving ON-bars (red), the bottom row shows peak responses to moving OFF-bars (turqoise). The peak responses are averaged over bar-speeds. Bar-stimuli move in different directions from 0 to 360 degrees. The responses from the different models in the different clusters (columns) overlay. Responses from better task-performing models are more saturated.

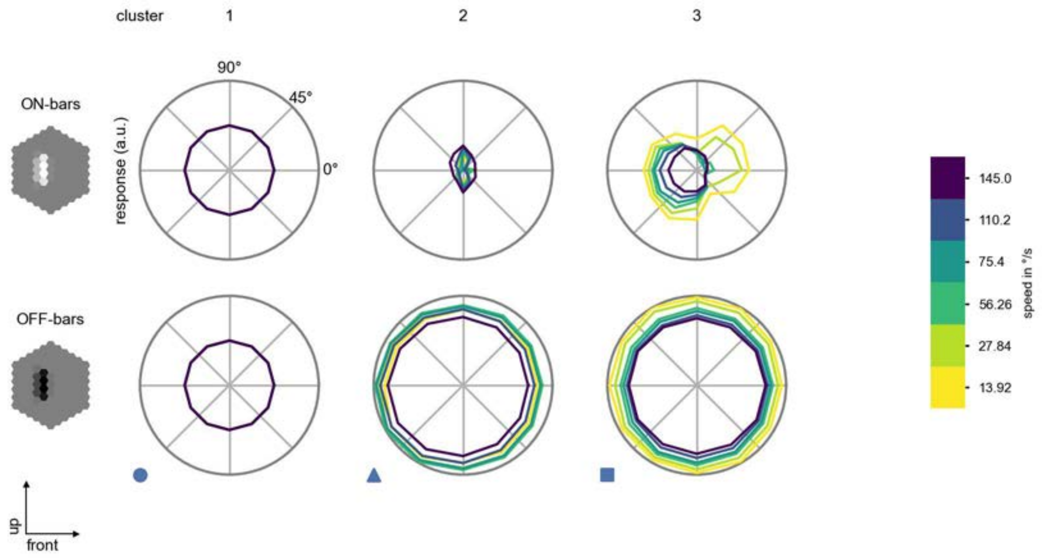

Am - Figure 10: **Peak responses to moving bars from task-optimal models.** The top row shows peak responses to moving ON-bars, the bottom row shows peak responses to moving OFF-bars of varying speeds from 13.92°/s to 145°/s (yellow to dark blue). The bar-stimuli move in different directions from 0 to 360 degrees and at different speeds. Responses from the task-optimal model in the respective cluster.

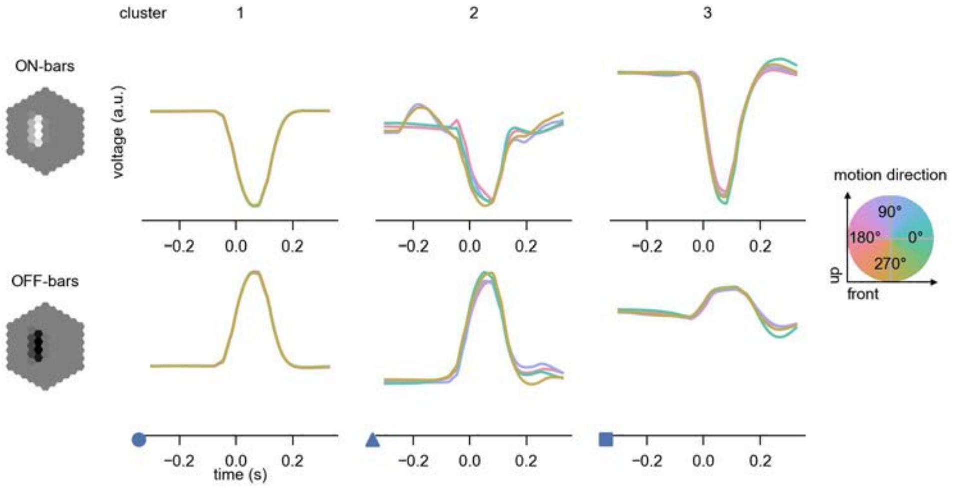

Am - Figure 11: **Responses to moving bars from task-optimal models.** Responses to moving ON-bars (top row) and to moving OFF-bars (bottom row). Bars move in different directions from 0 to 360 degrees and at different speeds. Responses are from the task-optimal model in the respective cluster. Bars moving at 75.4°/s in all cardinal directions (green 0°, blue 90°, red 180°, yellow 270°) from -22.5 to 22.5° visual angle.

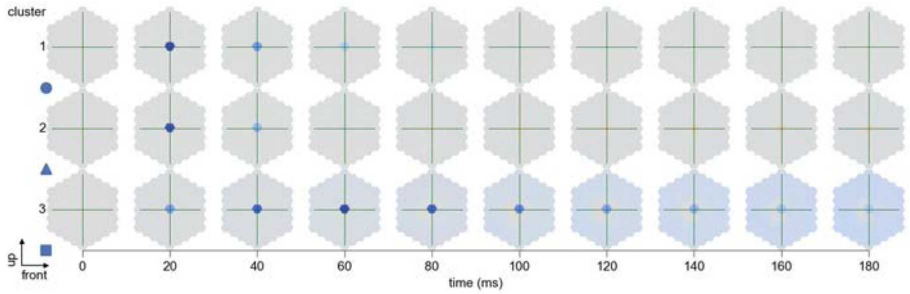

Am - Figure 12: **Spatio-temporal receptive field.** Responses of the central cell to ON-impulses (5 ms) at single-ommatidium flash locations. The flash occurs at second zero. Responses from the task-optimal model of the respective cluster (rows). Red indicates depolarization, blue indicates hyperpolarization.

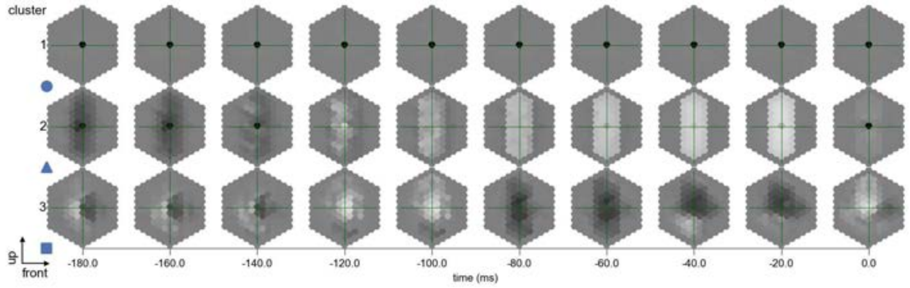

Am - Figure 13: **Maximally excitatory stimuli.** Each row presents the regularized naturalistic-stimulus from the Sintel dataset that maximizes the cell type's central column response at second zero in the task-optimal model of the respective cluster (rows).

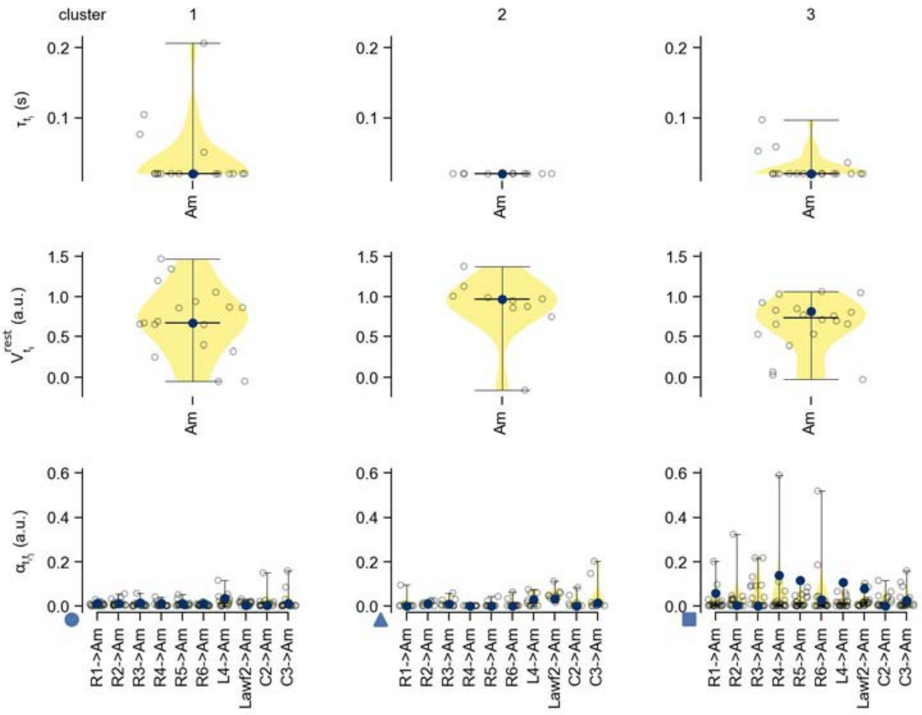

Am - Figure 14: **Task-constrained parameters.** Each column shows the parameters inferred within the respective cluster. First row: learned time constants of the cell type. Second row: resting potentials of the cell type. Third row: scaling factors for the convolutional filters. The blue scatter represents the parameters from the task-optimal model within the cluster.

17 C2

← Cell types

Figures

1 Anatomical receptive fields. 116
2 Anatomical projective fields. 117
3 Clustering of the responses to naturalistic stimuli. 117
4 Responses to flashes. 118
5 Cluster-average responses to single-ommatidium flashes. 118
6 Peak responses to moving edges. 119
7 Peak responses to moving edges from task-optimal models. 119
8 Responses to moving edges from task-optimal models. 120
9 Peak responses to moving bars. 120
10 Peak responses to moving bars from task-optimal models. 121
11 Responses to moving bars from task-optimal models. 121
12 Spatio-temporal receptive field. 121
13 Maximally excitatory stimuli. 122
14 Task-constrained parameters. 122

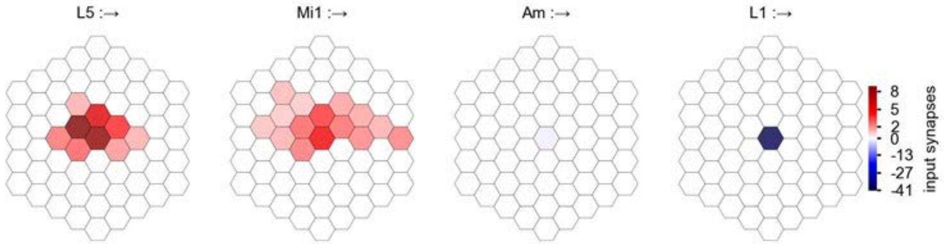

C2 - Figure 1: **Anatomical receptive fields.** Each colored hexagon is an input connection, with the connection strength characterized by the average number of synapses that we count from the EM reconstruction. Red indicates excitatory synapses, blue indicates inhibitory synapses from inferred signs. Filters in the order of their total number of synapses.

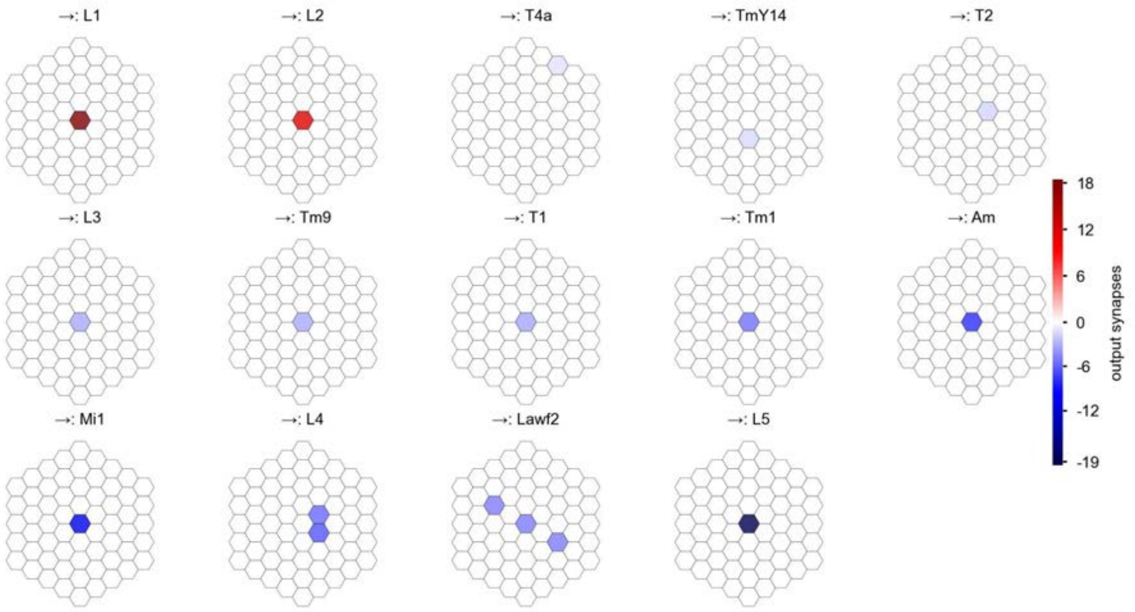

C2 - Figure 2: **Anatomical projective fields.** Each colored hexagon is an output connection, with the connection strength characterized by the average number of synapses that we count from the EM reconstruction. Red indicates excitatory synapses, blue indicates inhibitory synapses from inferred signs. Filters in the order of their total number of synapses.

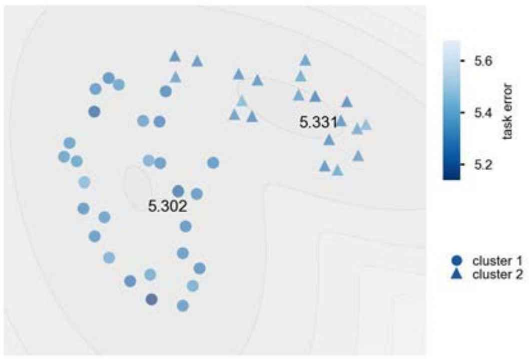

C2 - Figure 3: **Clustering of the responses to naturalistic stimuli.** Clustering of the 50 models based on the cell type responses to naturalistic scenes from the Sintel dataset. Scatterpoints represent individual models colored by their task error.

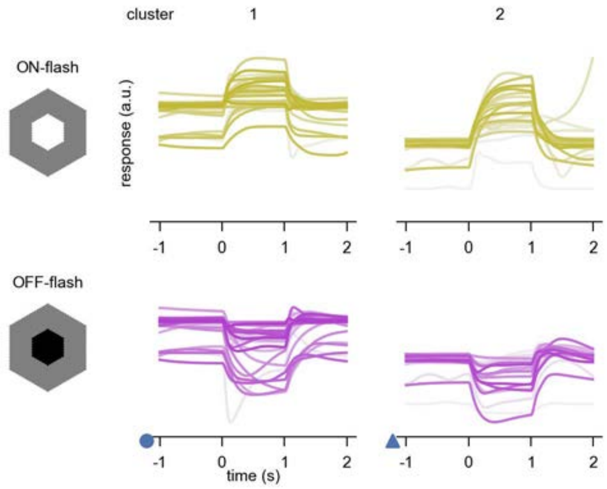

C2 - Figure 4: **Responses to flashes.** The top row shows responses to ON-flashes (yellow), the bottom row shows responses to OFF-flashes (magenta). The responses from the 50 different models that are separated into the different clusters (columns) overlay, with better task-performing models on top. Responses from better task-performing models are more saturated. The circular flashes (1s) cover 6 ommatidia in radius and are presented at time zero. Before and after, a grey-stimulus leads to a stationary state of the network.

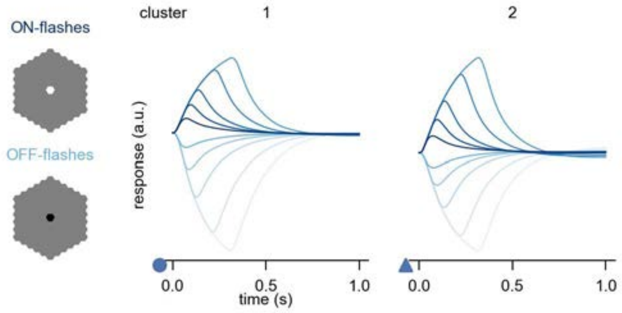

C2 - Figure 5: **Cluster-average responses to single-ommatidium flashes.** Responses to single-ommatidium ON-flashes (dark blue shades) and single-ommatidium OFF-flashes (light blue shades) of 20ms, 50ms, 100ms, 200ms, 300ms duration. The flashes occur at second zero.

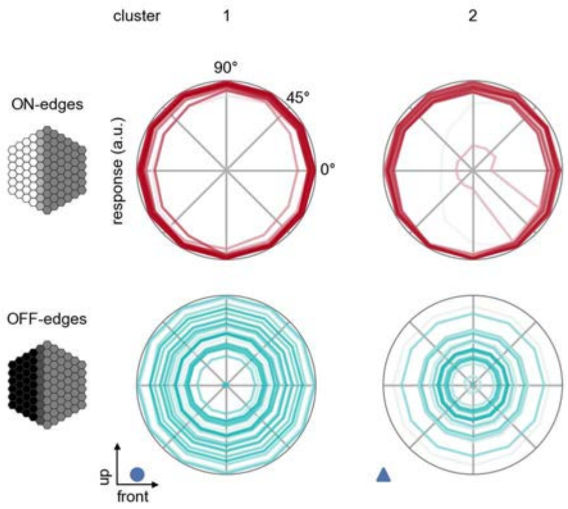

C2 - Figure 6: **Peak responses to moving edges.** The top row shows peak responses to moving ON-edges (red), the bottom row shows peak responses to moving OFF-edges (turqoise). The peak responses are averaged over edge-speeds. Edge-stimuli move in different directions from 0 to 360 degrees. The responses from the different models in the different clusters (columns) overlay. Responses from better task-performing models are more saturated.

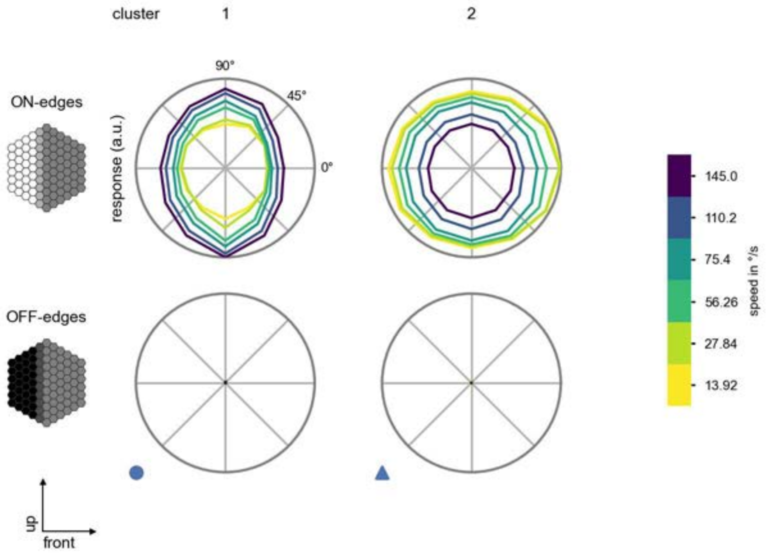

C2 - Figure 7: **Peak responses to moving edges from task-optimal models.** The top row shows peak responses to moving ON-edges, the bottom row shows peak responses to moving OFF-edges of varying speeds from 13.92°/s to 145°/s (yellow to dark blue). The edge-stimuli move in different directions from 0 to 360 degrees and at different speeds. Responses from the task-optimal model in the respective cluster.

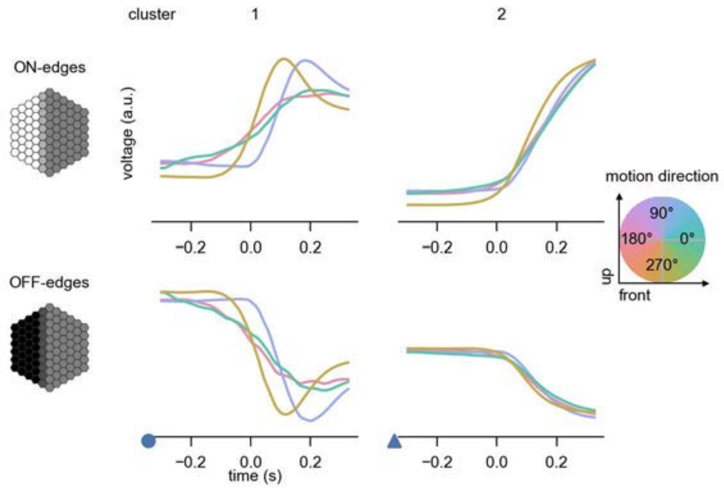

C2 - Figure 8: **Responses to moving edges from task-optimal models.** Responses to moving ON-edges (top row) and to moving OFF-edges (bottom row). Edges move in different directions from 0 to 360 degrees and at different speeds. Responses are from the task-optimal model in the respective cluster. Edges moving at 75.4°/s in all cardinal directions (green 0°, blue 90°, red 180°, yellow 270°) from -22.5 to 22.5° visual angle.

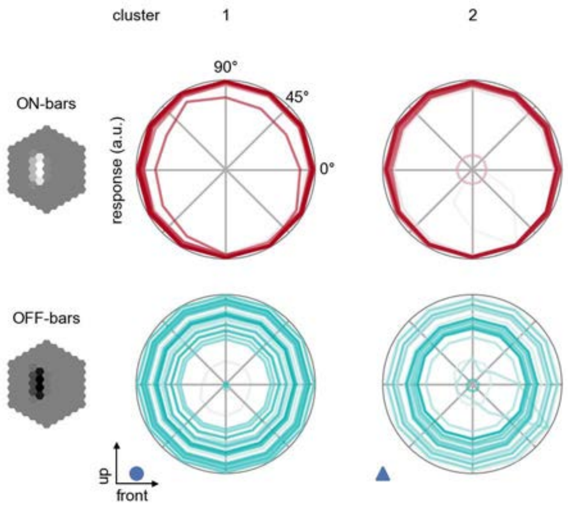

C2 - Figure 9: **Peak responses to moving bars.** The top row shows peak responses to moving ON-bars (red), the bottom row shows peak responses to moving OFF-bars (turqoise). The peak responses are averaged over bar-speeds. Bar-stimuli move in different directions from 0 to 360 degrees. The responses from the different models in the different clusters (columns) overlay. Responses from better task-performing models are more saturated.

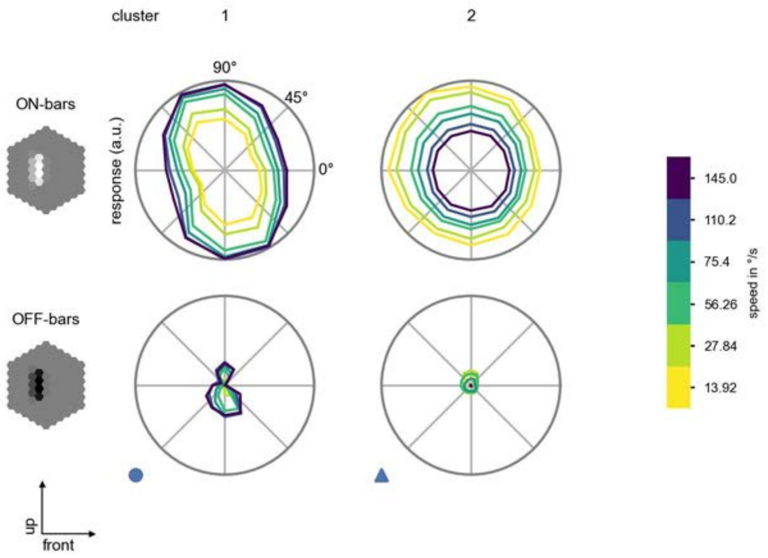

C2 - Figure 10: **Peak responses to moving bars from task-optimal models.** The top row shows peak responses to moving ON-bars, the bottom row shows peak responses to moving OFF-bars of varying speeds from 13.92°/s to 145°/s (yellow to dark blue). The bar-stimuli move in different directions from 0 to 360 degrees and at different speeds. Responses from the task-optimal model in the respective cluster.

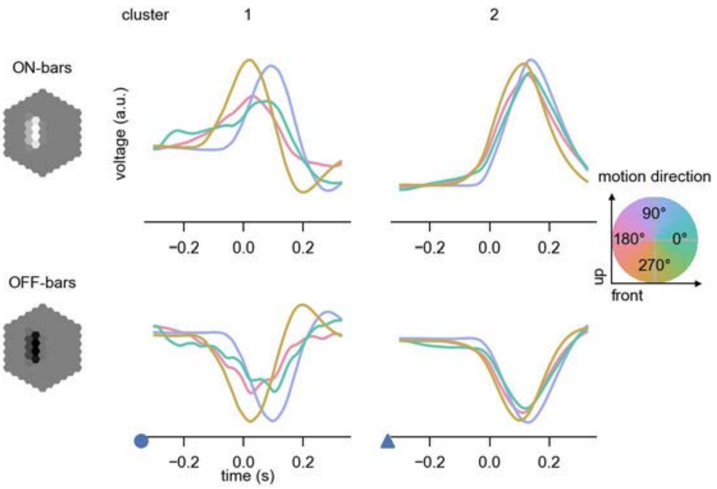

C2 - Figure 11: **Responses to moving bars from task-optimal models.** Responses to moving ON-bars (top row) and to moving OFF-bars (bottom row). Bars move in different directions from 0 to 360 degrees and at different speeds. Responses are from the task-optimal model in the respective cluster. Bars moving at 75.4°/s in all cardinal directions (green 0°, blue 90°, red 180°, yellow 270°) from -22.5 to 22.5° visual angle.

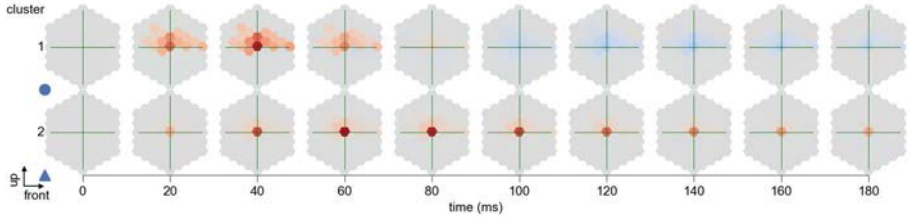

C2 - Figure 12: **Spatio-temporal receptive field.** Responses of the central cell to ON-impulses (5 ms) at single-ommatidium flash locations. The flash occurs at second zero. Responses from the task-optimal model of the respective cluster (rows). Red indicates depolarization, blue indicates hyperpolarization.

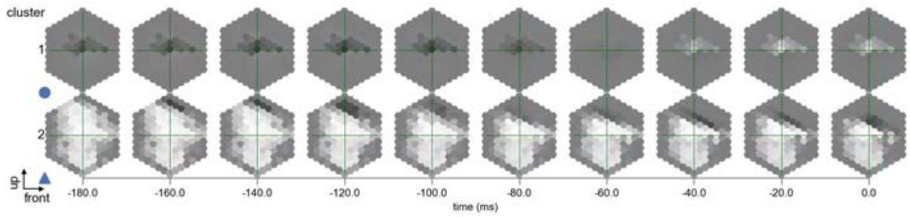

C2 - Figure 13: **Maximally excitatory stimuli.** Each row presents the regularized naturalistic-stimulus from the Sintel dataset that maximizes the cell type's central column response at second zero in the task-optimal model of the respective cluster (rows).

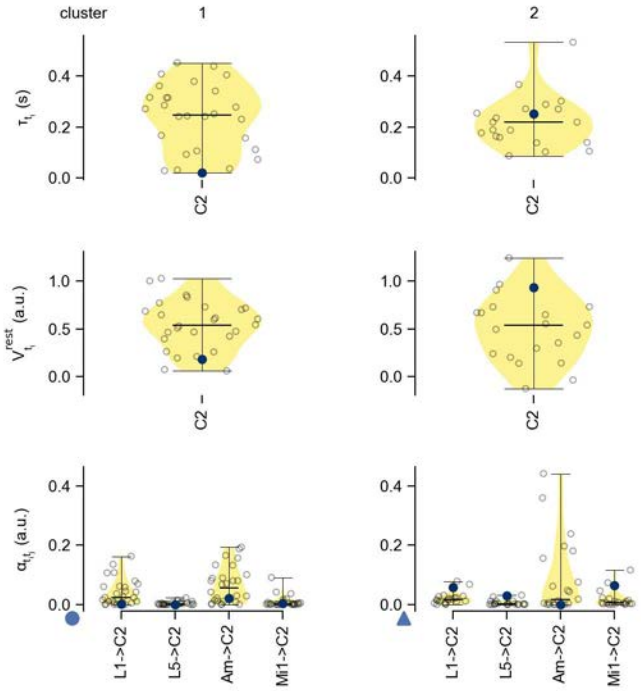

C2 - Figure 14: **Task-constrained parameters.** Each column shows the parameters inferred within the respective cluster. First row: learned time constants of the cell type. Second row: resting potentials of the cell type. Third row: scaling factors for the convolutional filters. The blue scatter represents the parameters from the task-optimal model within the cluster.

18 C3

← Cell types

Figures

1 Anatomical receptive fields. . . . 123
2 Anatomical projective fields. . . . 124
3 Clustering of the responses to naturalistic stimuli. . . . 124
4 Responses to flashes. . . . 125
5 Cluster-average responses to single-ommatidium flashes. . . . 125
6 Peak responses to moving edges. . . . 126
7 Peak responses to moving edges from task-optimal models. . . . 126
8 Responses to moving edges from task-optimal models. . . . 127
9 Peak responses to moving bars. . . . 127
10 Peak responses to moving bars from task-optimal models. . . . 128
11 Responses to moving bars from task-optimal models. . . . 128
12 Spatio-temporal receptive field. . . . 129
13 Maximally excitatory stimuli. . . . 129
14 Task-constrained parameters. . . . 130

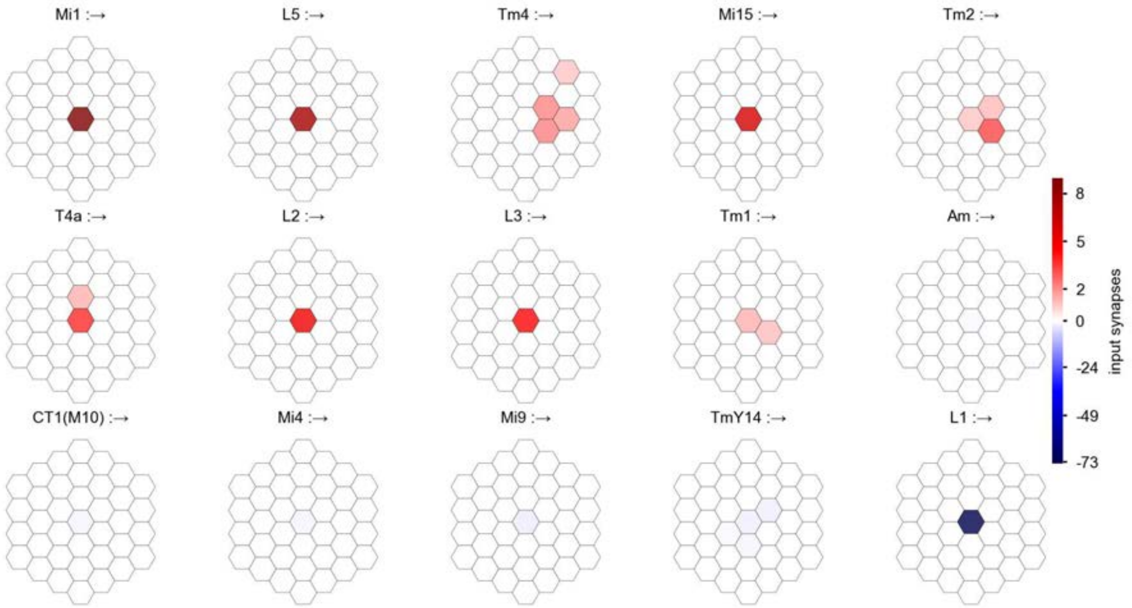

C3 - Figure 1: **Anatomical receptive fields.** Each colored hexagon is an input connection, with the connection strength characterized by the average number of synapses that we count from the EM reconstruction. Red indicates excitatory synapses, blue indicates inhibitory synapses from inferred signs. Filters in the order of their total number of synapses.

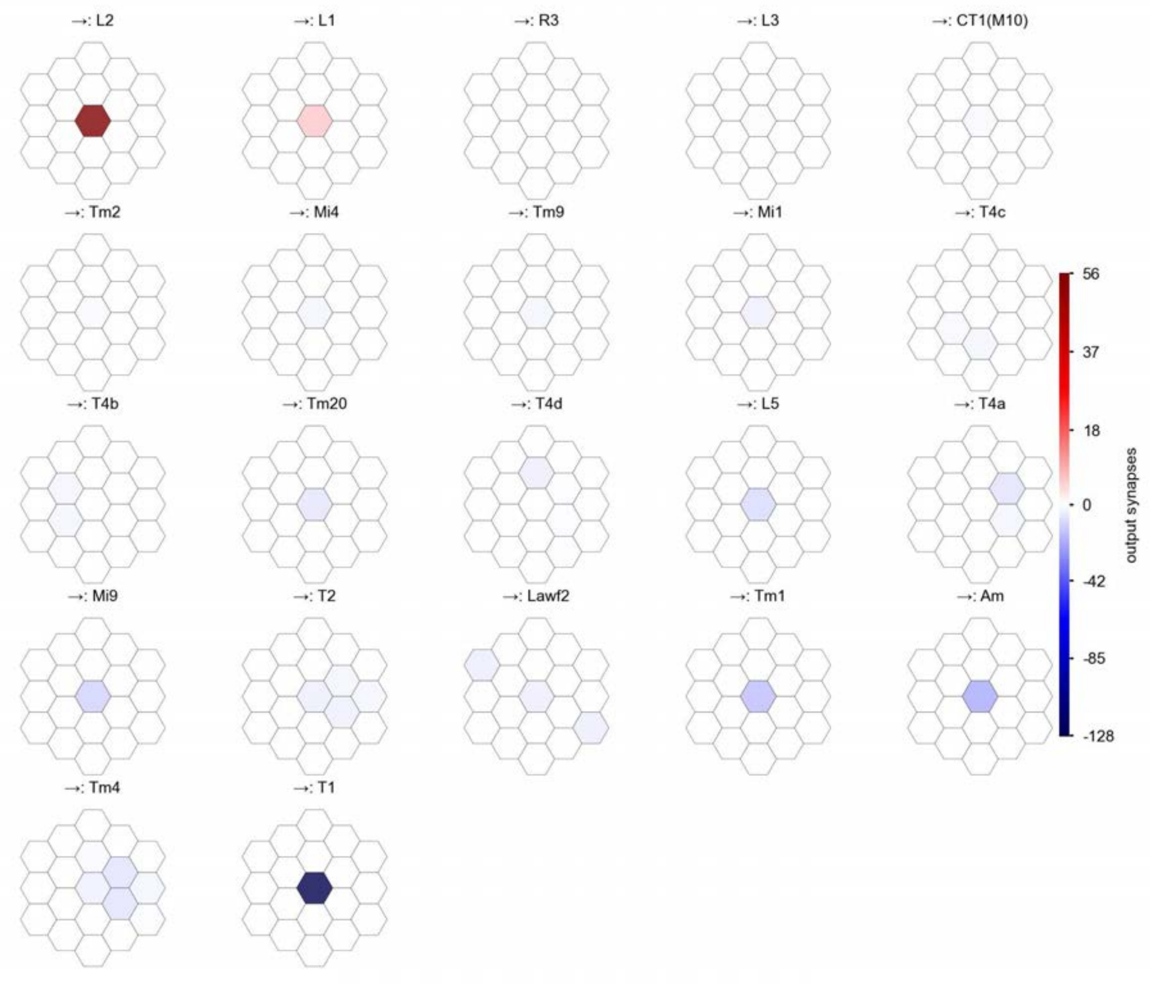

C3 - Figure 2: **Anatomical projective fields.** Each colored hexagon is an output connection, with the connection strength characterized by the average number of synapses that we count from the EM reconstruction. Red indicates excitatory synapses, blue indicates inhibitory synapses from inferred signs. Filters in the order of their total number of synapses.

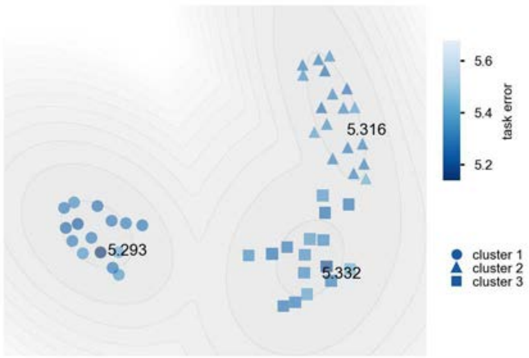

C3 - Figure 3: **Clustering of the responses to naturalistic stimuli.** Clustering of the 50 models based on the cell type responses to naturalistic scenes from the Sintel dataset. Scatterpoints represent individual models colored by their task error.

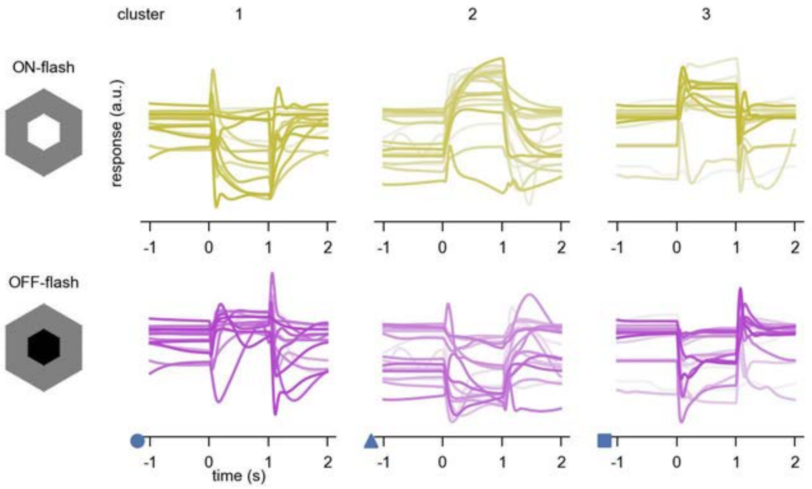

C3 - Figure 4: **Responses to flashes.** The top row shows responses to ON-flashes (yellow), the bottom row shows responses to OFF-flashes (magenta). The responses from the 50 different models that are separated into the different clusters (columns) overlay, with better task-performing models on top. Responses from better task-performing models are more saturated. The circular flashes (1s) cover 6 ommatidia in radius and are presented at time zero. Before and after, a grey-stimulus leads to a stationary state of the network.

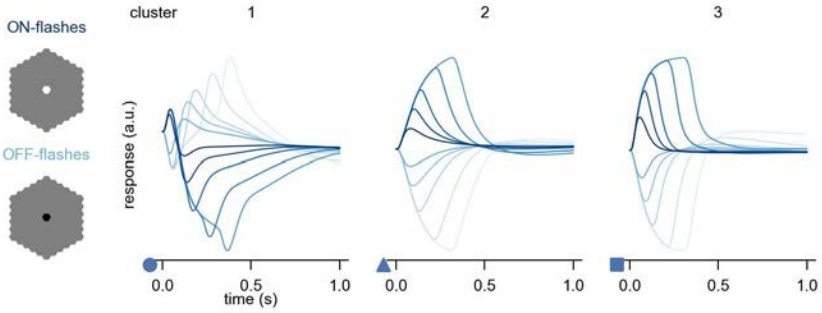

C3 - Figure 5: **Cluster-average responses to single-ommatidium flashes.** Responses to single-ommatidium ON-flashes (dark blue shades) and single-ommatidium OFF-flashes (light blue shades) of 20ms, 50ms, 100ms, 200ms, 300ms duration. The flashes occur at second zero.

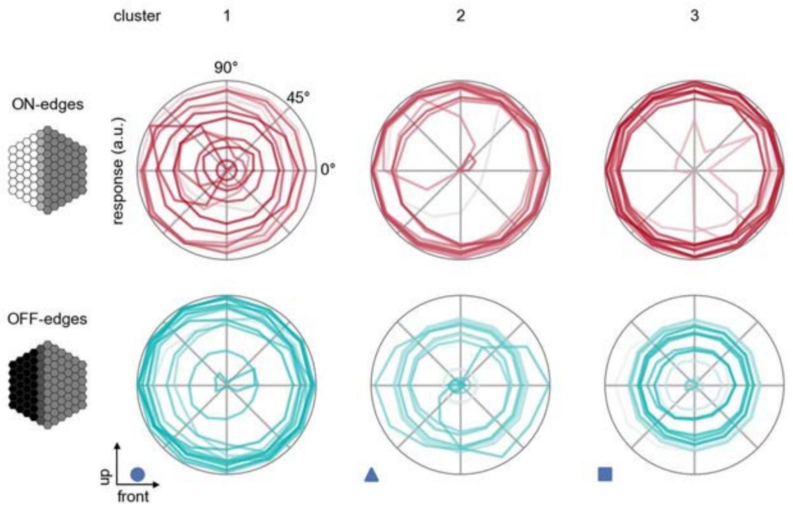

C3 - Figure 6: **Peak responses to moving edges.** The top row shows peak responses to moving ON-edges (red), the bottom row shows peak responses to moving OFF-edges (turqoise). The peak responses are averaged over edge-speeds. Edge-stimuli move in different directions from 0 to 360 degrees. The responses from the different models in the different clusters (columns) overlay. Responses from better task-performing models are more saturated.

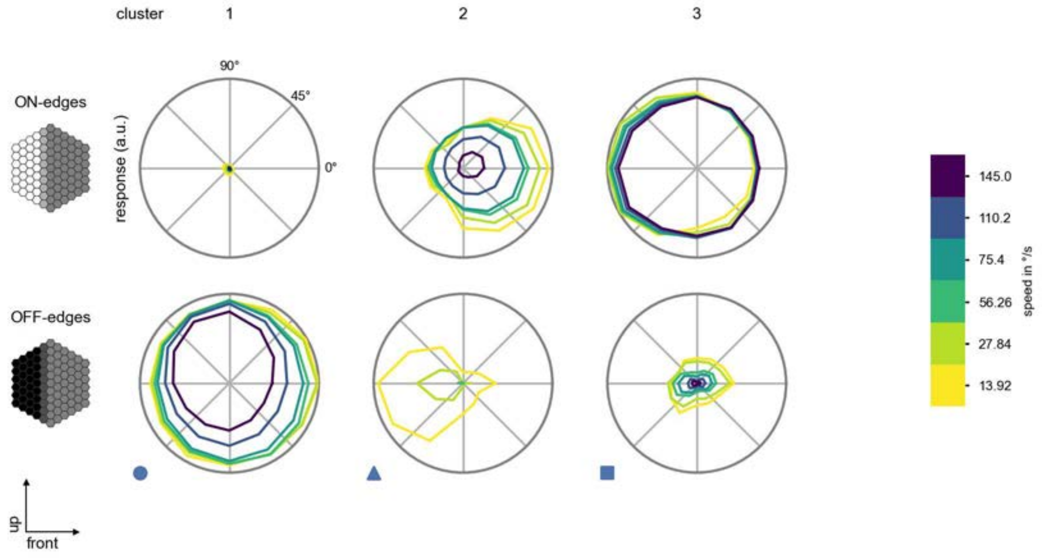

C3 - Figure 7: **Peak responses to moving edges from task-optimal models.** The top row shows peak responses to moving ON-edges, the bottom row shows peak responses to moving OFF-edges of varying speeds from 13.92°/s to 145°/s (yellow to dark blue). The edge-stimuli move in different directions from 0 to 360 degrees and at different speeds. Responses from the task-optimal model in the respective cluster.

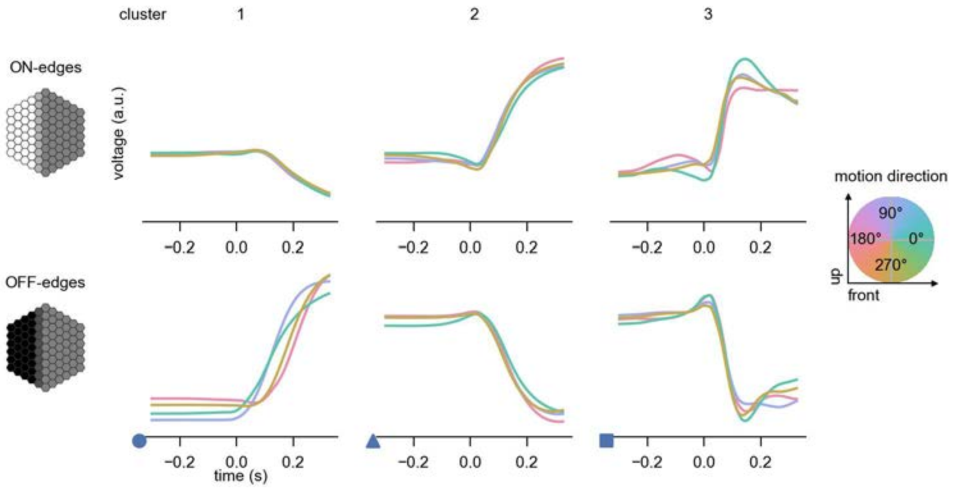

C3 - Figure 8: **Responses to moving edges from task-optimal models.** Responses to moving ON-edges (top row) and to moving OFF-edges (bottom row). Edges move in different directions from 0 to 360 degrees and at different speeds. Responses are from the task-optimal model in the respective cluster. Edges moving at 75.4°/s in all cardinal directions (green 0°, blue 90°, red 180°, yellow 270°) from -22.5 to 22.5° visual angle.

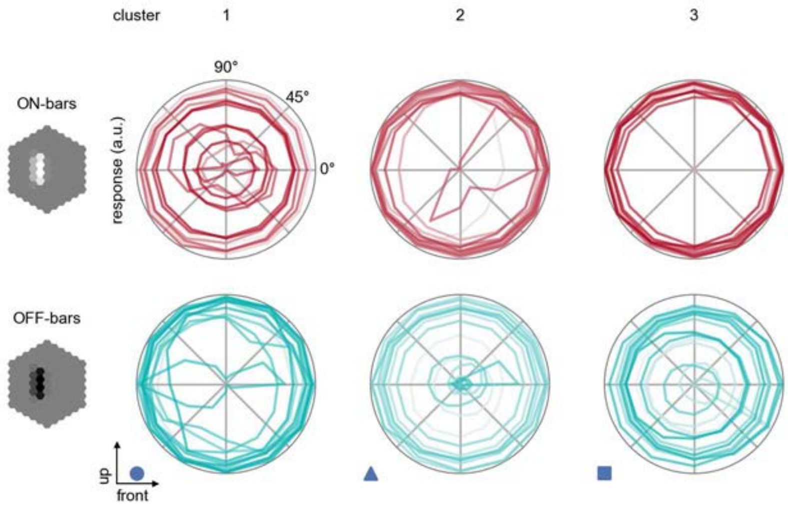

C3 - Figure 9: **Peak responses to moving bars.** The top row shows peak responses to moving ON-bars (red), the bottom row shows peak responses to moving OFF-bars (turqoise). The peak responses are averaged over bar-speeds. Bar-stimuli move in different directions from 0 to 360 degrees. The responses from the different models in the different clusters (columns) overlay. Responses from better task-performing models are more saturated.

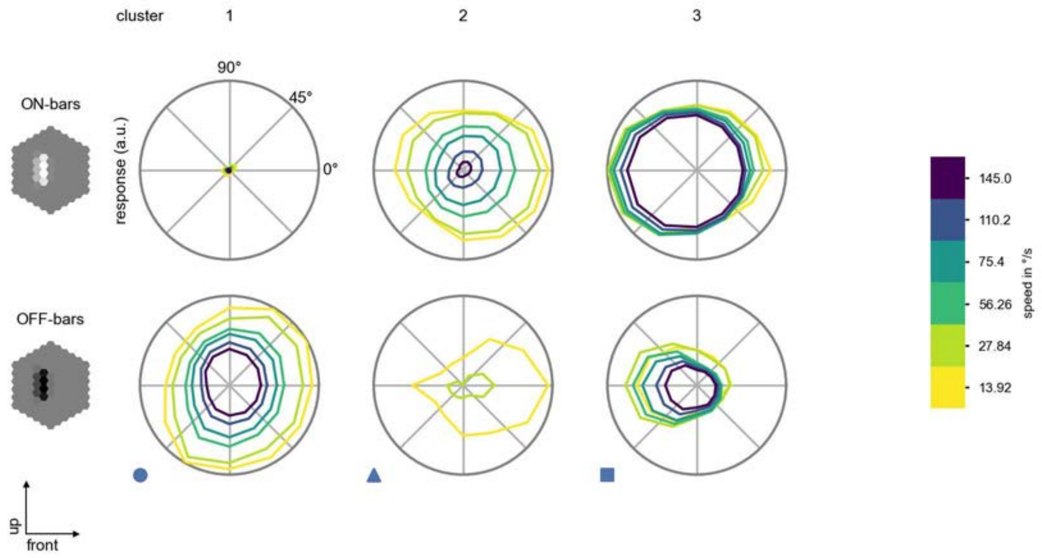

C3 - Figure 10: **Peak responses to moving bars from task-optimal models.** The top row shows peak responses to moving ON-bars, the bottom row shows peak responses to moving OFF-bars of varying speeds from 13.92°/s to 145°/s (yellow to dark blue). The bar-stimuli move in different directions from 0 to 360 degrees and at different speeds. Responses from the task-optimal model in the respective cluster.

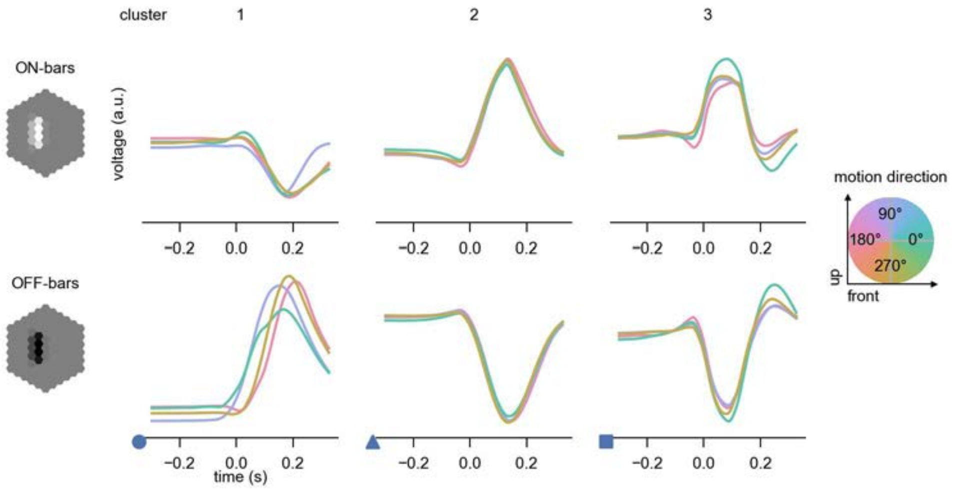

C3 - Figure 11: **Responses to moving bars from task-optimal models.** Responses to moving ON-bars (top row) and to moving OFF-bars (bottom row). Bars move in different directions from 0 to 360 degrees and at different speeds. Responses are from the task-optimal model in the respective cluster. Bars moving at 75.4°/s in all cardinal directions (green 0°, blue 90°, red 180°, yellow 270°) from -22.5 to 22.5° visual angle.

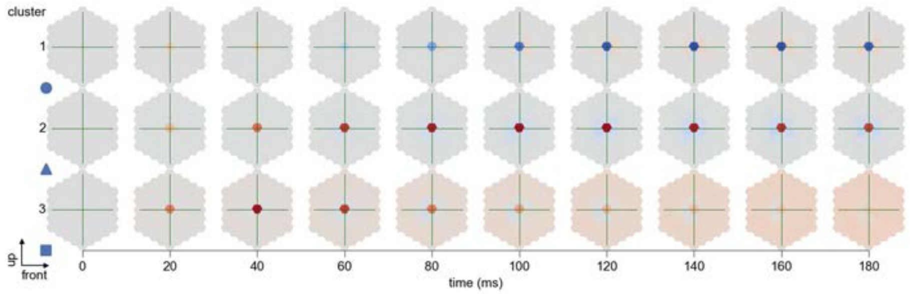

C3 - Figure 12: **Spatio-temporal receptive field.** Responses of the central cell to ON-impulses (5 ms) at single-ommatidium flash locations. The flash occurs at second zero. Responses from the task-optimal model of the respective cluster (rows). Red indicates depolarization, blue indicates hyperpolarization.

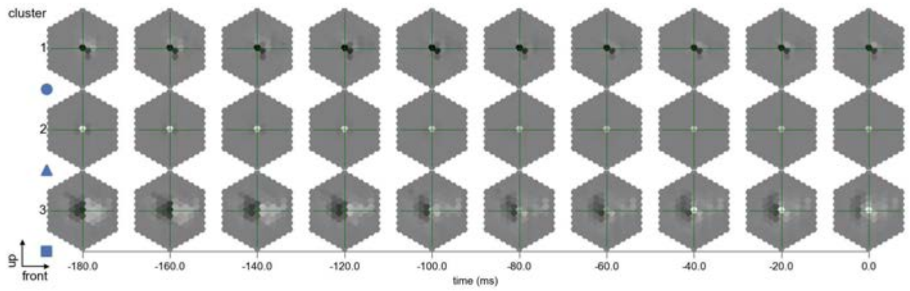

C3 - Figure 13: **Maximally excitatory stimuli.** Each row presents the regularized naturalistic-stimulus from the Sintel dataset that maximizes the cell type's central column response at second zero in the task-optimal model of the respective cluster (rows).

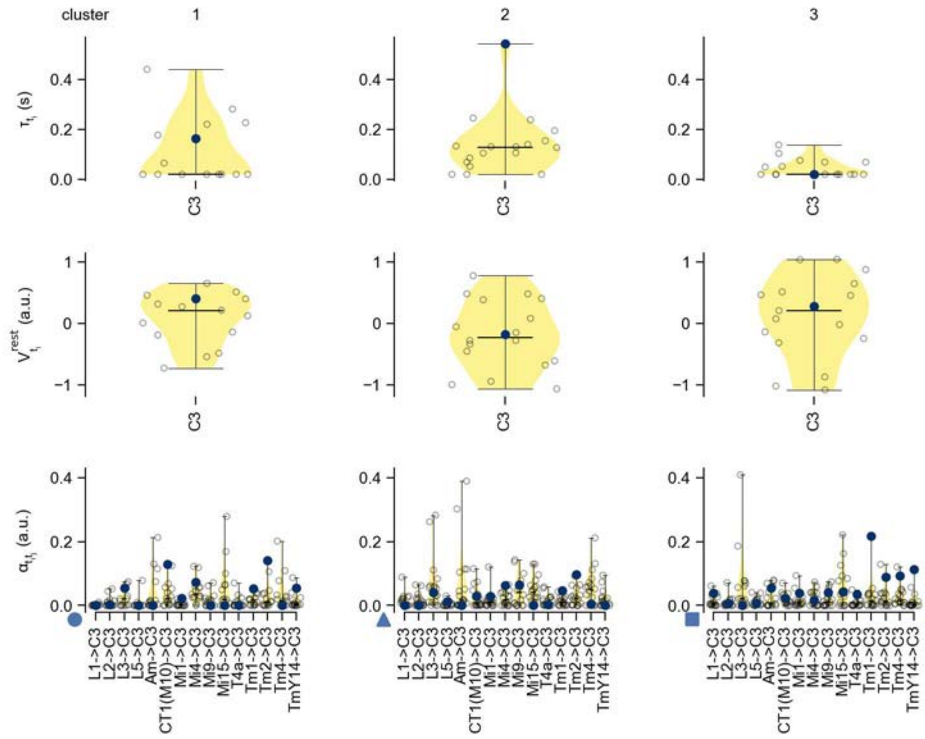

C3 - Figure 14: **Task-constrained parameters.** Each column shows the parameters inferred within the respective cluster. First row: learned time constants of the cell type. Second row: resting potentials of the cell type. Third row: scaling factors for the convolutional filters. The blue scatter represents the parameters from the task-optimal model within the cluster.

19 CT1(Lo1)

← Cell types

Figures

1 Anatomical receptive fields. 131
2 Anatomical projective fields. 132
3 Clustering of the responses to naturalistic stimuli. 132
4 Responses to flashes. 133
5 Cluster-average responses to single-ommatidium flashes. 133
6 Peak responses to moving edges. 134
7 Peak responses to moving edges from task-optimal models. 134
8 Responses to moving edges from task-optimal models. 135
9 Peak responses to moving bars. 135
10 Peak responses to moving bars from task-optimal models. 136
11 Responses to moving bars from task-optimal models. 136
12 Spatio-temporal receptive field. 137
13 Maximally excitatory stimuli. 137
14 Task-constrained parameters. 138

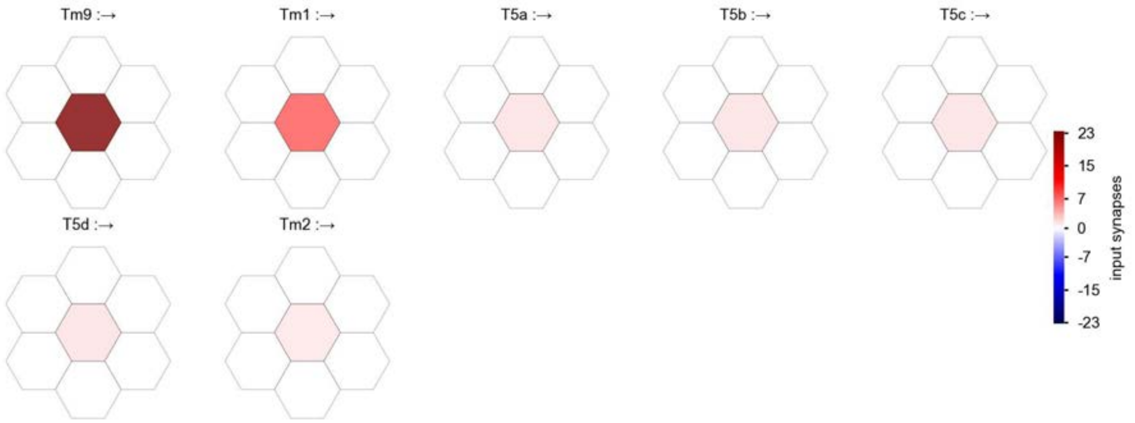

CT1(Lo1) - Figure 1: **Anatomical receptive fields.** Each colored hexagon is an input connection, with the connection strength characterized by the average number of synapses that we count from the EM reconstruction. Red indicates excitatory synapses, blue indicates inhibitory synapses from inferred signs. Filters in the order of their total number of synapses.

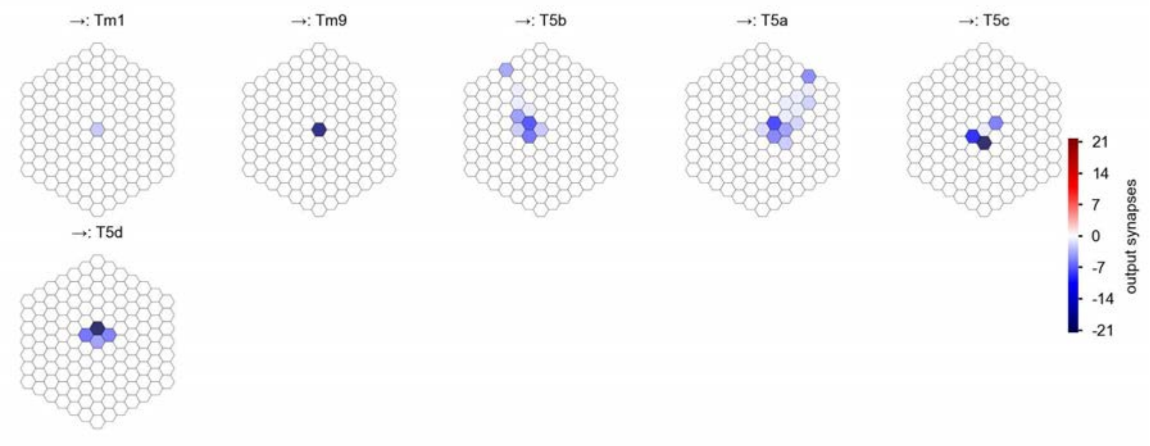

CT1(Lo1) - Figure 2: **Anatomical projective fields.** Each colored hexagon is an output connection, with the connection strength characterized by the average number of synapses that we count from the EM reconstruction. Red indicates excitatory synapses, blue indicates inhibitory synapses from inferred signs. Filters in the order of their total number of synapses.

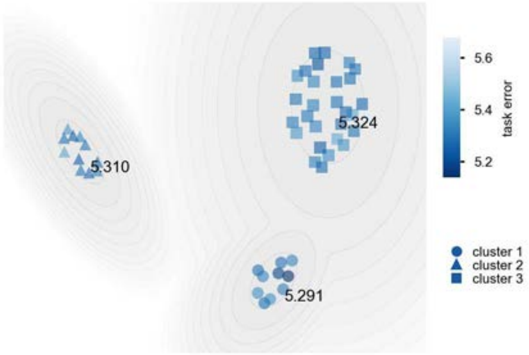

CT1(Lo1) - Figure 3: **Clustering of the responses to naturalistic stimuli.** Clustering of the 50 models based on the cell type responses to naturalistic scenes from the Sintel dataset. Scatterpoints represent individual models colored by their task error.

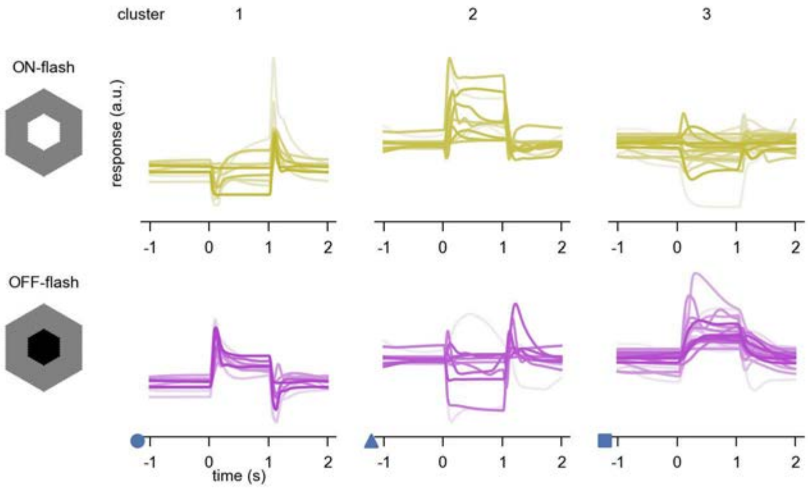

CT1(Lo1) - Figure 4: **Responses to flashes.** The top row shows responses to ON-flashes (yellow), the bottom row shows responses to OFF-flashes (magenta). The responses from the 50 different models that are separated into the different clusters (columns) overlay, with better task-performing models on top. Responses from better task-performing models are more saturated. The circular flashes (1s) cover 6 ommatidia in radius and are presented at time zero. Before and after, a grey-stimulus leads to a stationary state of the network.

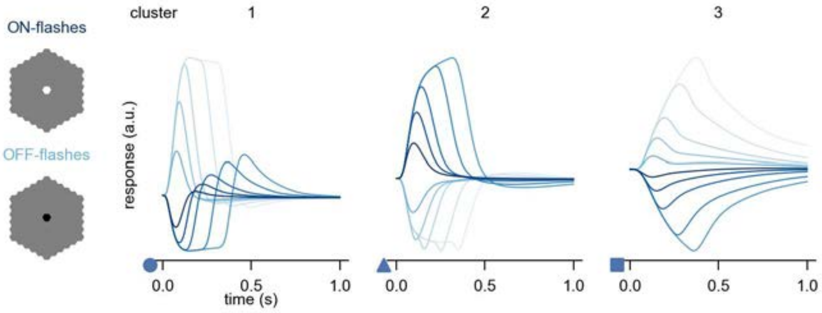

CT1(Lo1) - Figure 5: **Cluster-average responses to single-ommatidium flashes.** Responses to single-ommatidium ON-flashes (dark blue shades) and single-ommatidium OFF-flashes (light blue shades) of 20ms, 50ms, 100ms, 200ms, 300ms duration. The flashes occur at second zero.

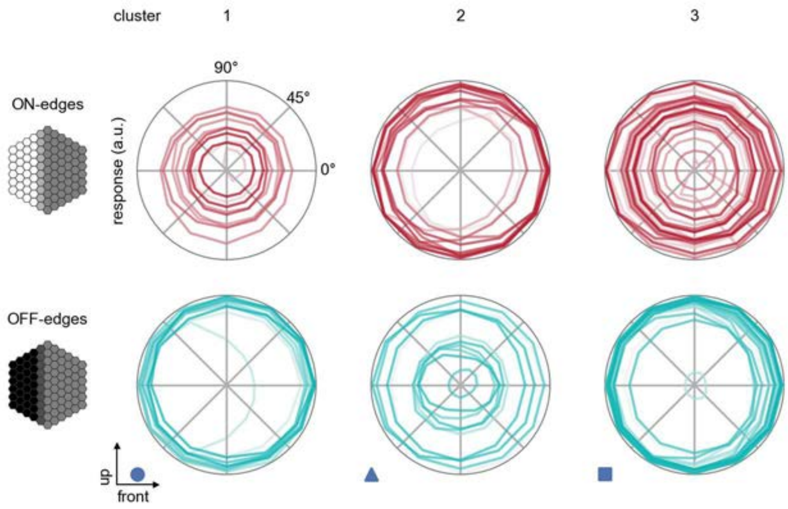

CT1(Lo1) - Figure 6: **Peak responses to moving edges.** The top row shows peak responses to moving ON-edges (red), the bottom row shows peak responses to moving OFF-edges (turqoise). The peak responses are averaged over edge-speeds. Edge-stimuli move in different directions from 0 to 360 degrees. The responses from the different models in the different clusters (columns) overlay. Responses from better task-performing models are more saturated.

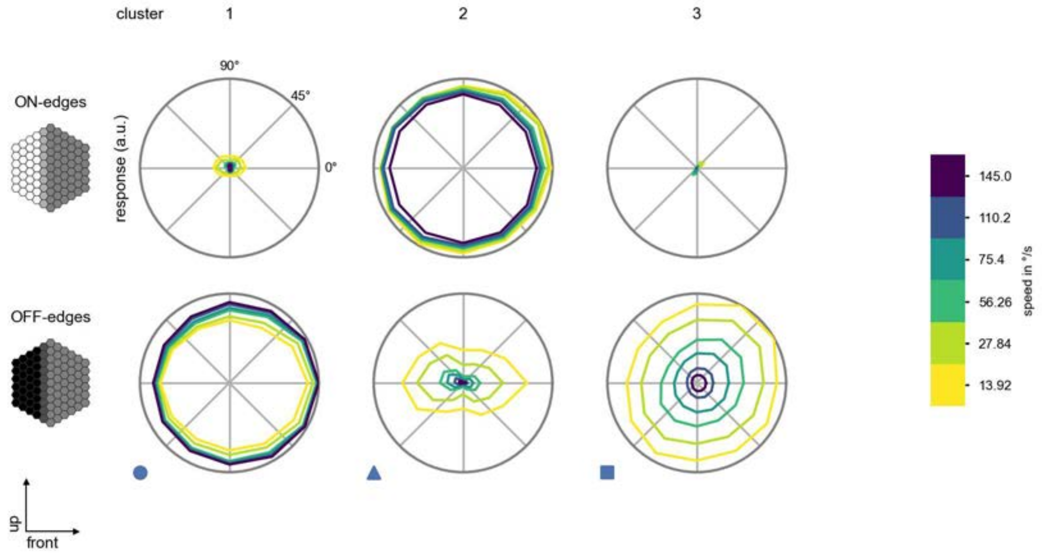

CT1(Lo1) - Figure 7: **Peak responses to moving edges from task-optimal models.** The top row shows peak responses to moving ON-edges, the bottom row shows peak responses to moving OFF-edges of varying speeds from 13.92°/s to 145°/s (yellow to dark blue). The edge-stimuli move in different directions from 0 to 360 degrees and at different speeds. Responses from the task-optimal model in the respective cluster.

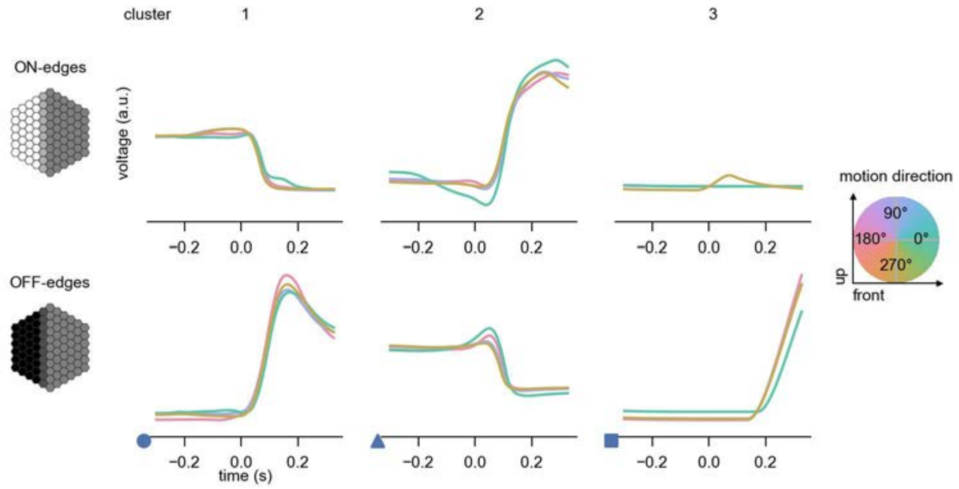

CT1(Lo1) - Figure 8: **Responses to moving edges from task-optimal models.** Responses to moving ON-edges (top row) and to moving OFF-edges (bottom row). Edges move in different directions from 0 to 360 degrees and at different speeds. Responses are from the task-optimal model in the respective cluster. Edges moving at 75.4°/s in all cardinal directions (green 0°, blue 90°, red 180°, yellow 270°) from -22.5 to 22.5° visual angle.

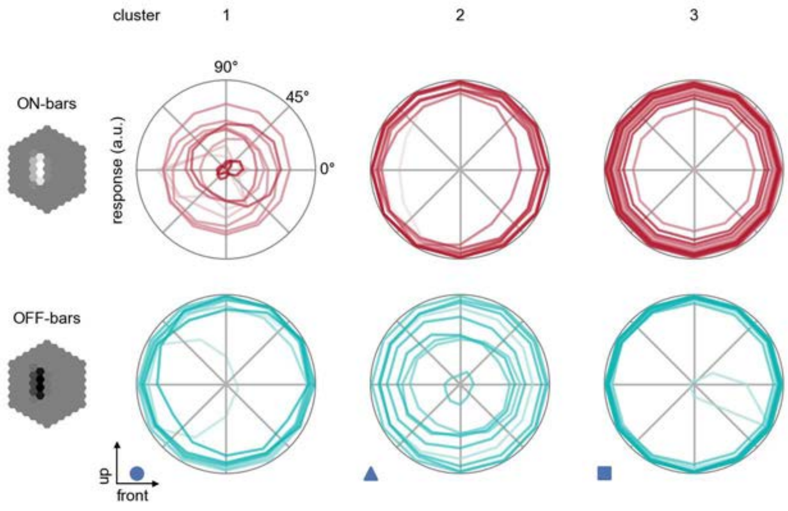

CT1(Lo1) - Figure 9: **Peak responses to moving bars.** The top row shows peak responses to moving ON-bars (red), the bottom row shows peak responses to moving OFF-bars (turqoise). The peak responses are averaged over bar-speeds. Bar-stimuli move in different directions from 0 to 360 degrees. The responses from the different models in the different clusters (columns) overlay. Responses from better task-performing models are more saturated.

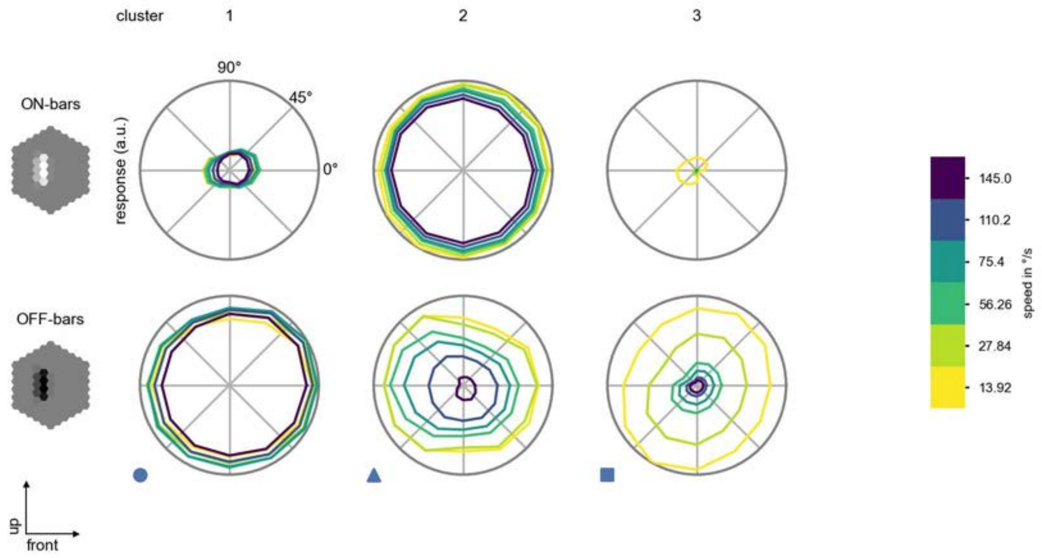

CT1(Lo1) - Figure 10: **Peak responses to moving bars from task-optimal models.** The top row shows peak responses to moving ON-bars, the bottom row shows peak responses to moving OFF-bars of varying speeds from 13.92°/s to 145°/s (yellow to dark blue). The bar-stimuli move in different directions from 0 to 360 degrees and at different speeds. Responses from the task-optimal model in the respective cluster.

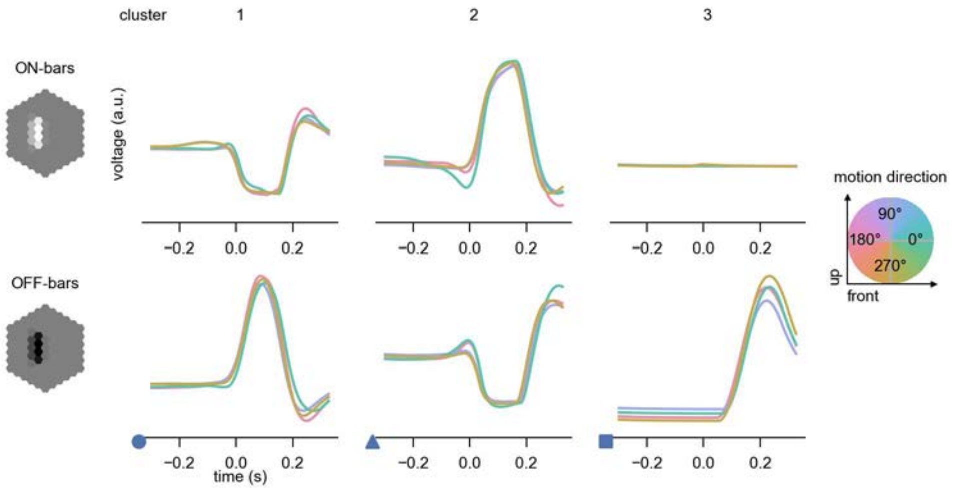

CT1(Lo1) - Figure 11: **Responses to moving bars from task-optimal models.** Responses to moving ON-bars (top row) and to moving OFF-bars (bottom row). Bars move in different directions from 0 to 360 degrees and at different speeds. Responses are from the task-optimal model in the respective cluster. Bars moving at 75.4°/s in all cardinal directions (green 0°, blue 90°, red 180°, yellow 270°) from -22.5 to 22.5° visual angle.

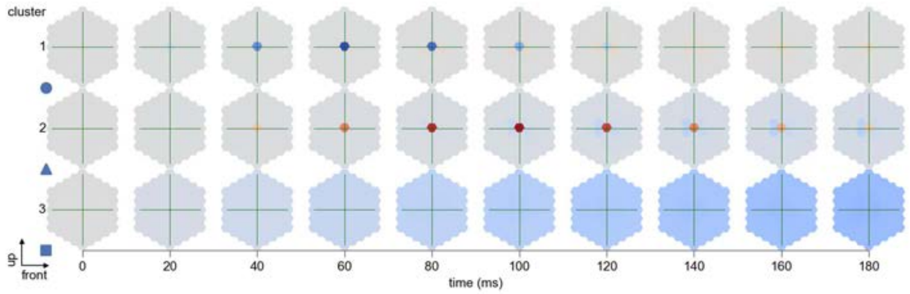

CT1(Lo1) - Figure 12: **Spatio-temporal receptive field.** Responses of the central cell to ON-impulses (5 ms) at single-ommatidium flash locations. The flash occurs at second zero. Responses from the task-optimal model of the respective cluster (rows). Red indicates depolarization, blue indicates hyperpolarization.

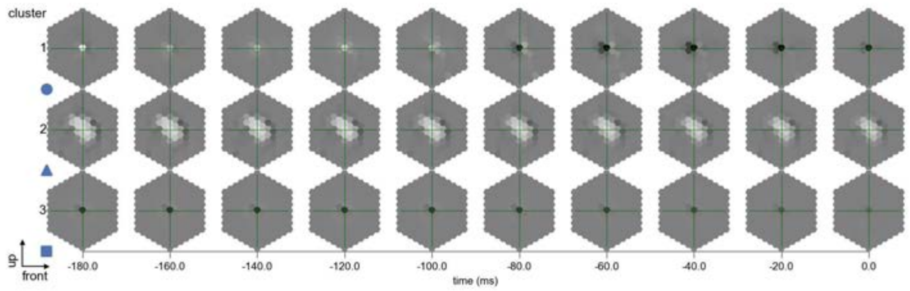

CT1(Lo1) - Figure 13: **Maximally excitatory stimuli.** Each row presents the regularized naturalistic-stimulus from the Sintel dataset that maximizes the cell type's central column response at second zero in the task-optimal model of the respective cluster (rows).

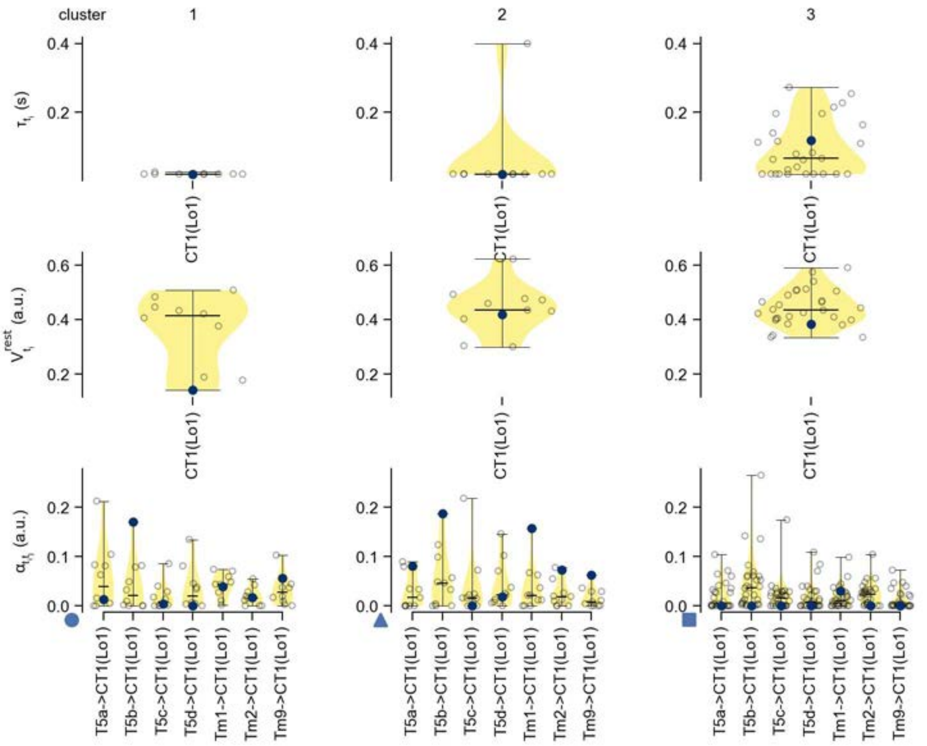

CT1(Lo1) - Figure 14: **Task-constrained parameters.** Each column shows the parameters inferred within the respective cluster. First row: learned time constants of the cell type. Second row: resting potentials of the cell type. Third row: scaling factors for the convolutional filters. The blue scatter represents the parameters from the task-optimal model within the cluster.

20 CT1(M10)

← Cell types

Figures

1 Anatomical receptive fields. 139
2 Anatomical projective fields. 140
3 Clustering of the responses to naturalistic stimuli. 140
4 Responses to flashes. 141
5 Cluster-average responses to single-ommatidium flashes. 141
6 Peak responses to moving edges. 142
7 Peak responses to moving edges from task-optimal models. 142
8 Responses to moving edges from task-optimal models. 143
9 Peak responses to moving bars. 143
10 Peak responses to moving bars from task-optimal models. 144
11 Responses to moving bars from task-optimal models. 144
12 Spatio-temporal receptive field. 145
13 Maximally excitatory stimuli. 145
14 Task-constrained parameters. 146

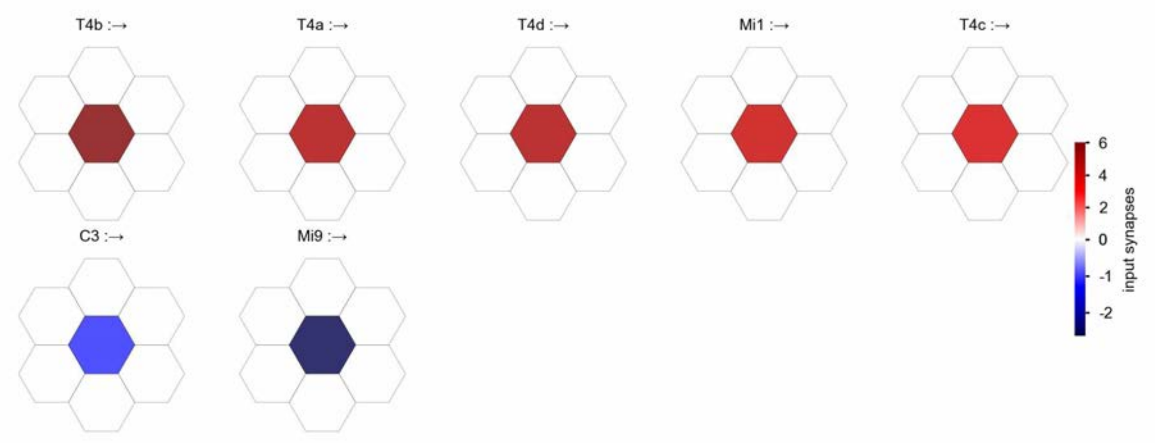

CT1(M10) - Figure 1: **Anatomical receptive fields.** Each colored hexagon is an input connection, with the connection strength characterized by the average number of synapses that we count from the EM reconstruction. Red indicates excitatory synapses, blue indicates inhibitory synapses from inferred signs. Filters in the order of their total number of synapses.

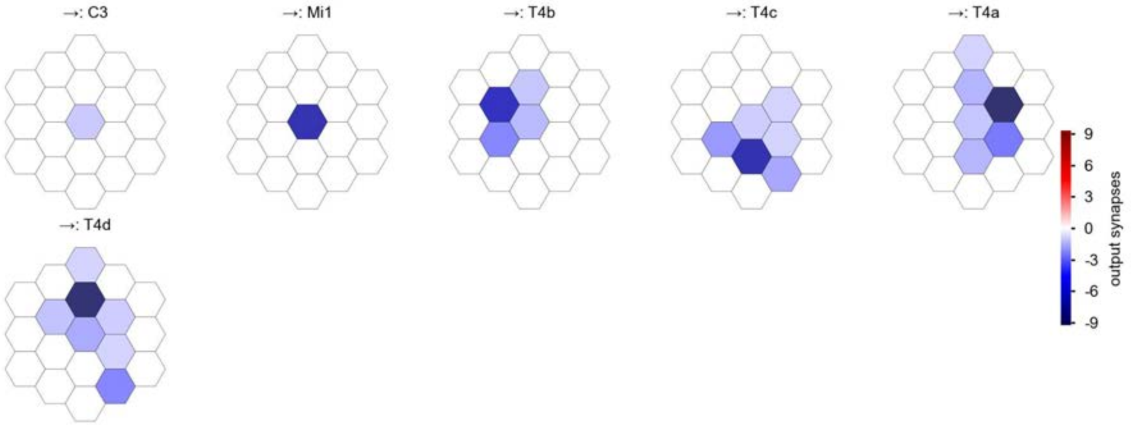

CT1(M10) - Figure 2: **Anatomical projective fields.** Each colored hexagon is an output connection, with the connection strength characterized by the average number of synapses that we count from the EM reconstruction. Red indicates excitatory synapses, blue indicates inhibitory synapses from inferred signs. Filters in the order of their total number of synapses.

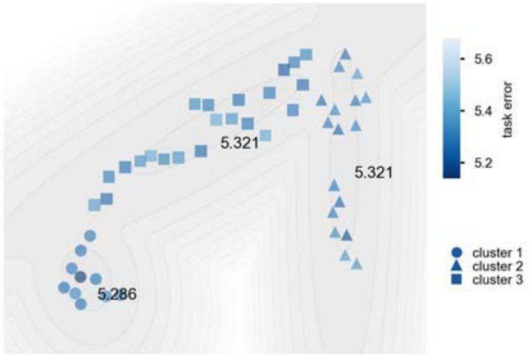

CT1(M10) - Figure 3: **Clustering of the responses to naturalistic stimuli.** Clustering of the 50 models based on the cell type responses to naturalistic scenes from the Sintel dataset. Scatterpoints represent individual models colored by their task error.

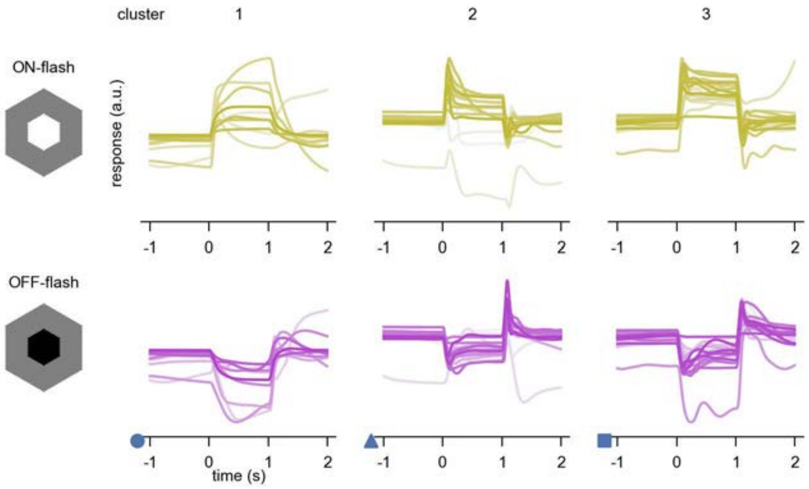

CT1(M10) - Figure 4: **Responses to flashes.** The top row shows responses to ON-flashes (yellow), the bottom row shows responses to OFF-flashes (magenta). The responses from the 50 different models that are separated into the different clusters (columns) overlay, with better task-performing models on top. Responses from better task-performing models are more saturated. The circular flashes (1s) cover 6 ommatidia in radius and are presented at time zero. Before and after, a grey-stimulus leads to a stationary state of the network.

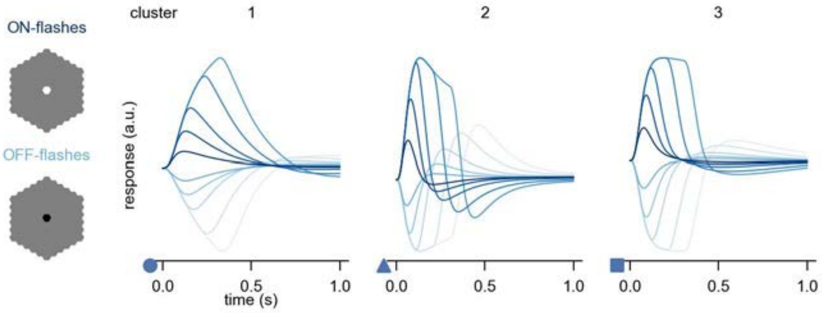

CT1(M10) - Figure 5: **Cluster-average responses to single-ommatidium flashes.** Responses to single-ommatidium ON-flashes (dark blue shades) and single-ommatidium OFF-flashes (light blue shades) of 20ms, 50ms, 100ms, 200ms, 300ms duration. The flashes occur at second zero.

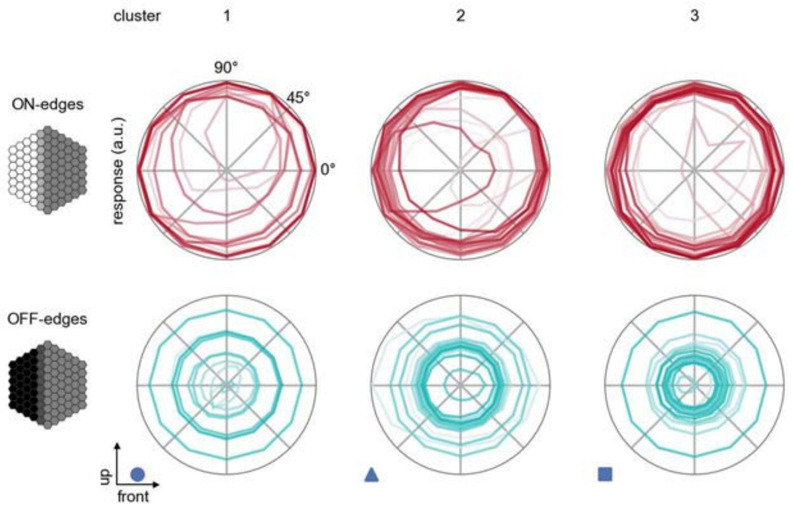

CT1(M10) - Figure 6: **Peak responses to moving edges.** The top row shows peak responses to moving ON-edges (red), the bottom row shows peak responses to moving OFF-edges (turqoise). The peak responses are averaged over edge-speeds. Edge-stimuli move in different directions from 0 to 360 degrees. The responses from the different models in the different clusters (columns) overlay. Responses from better task-performing models are more saturated.

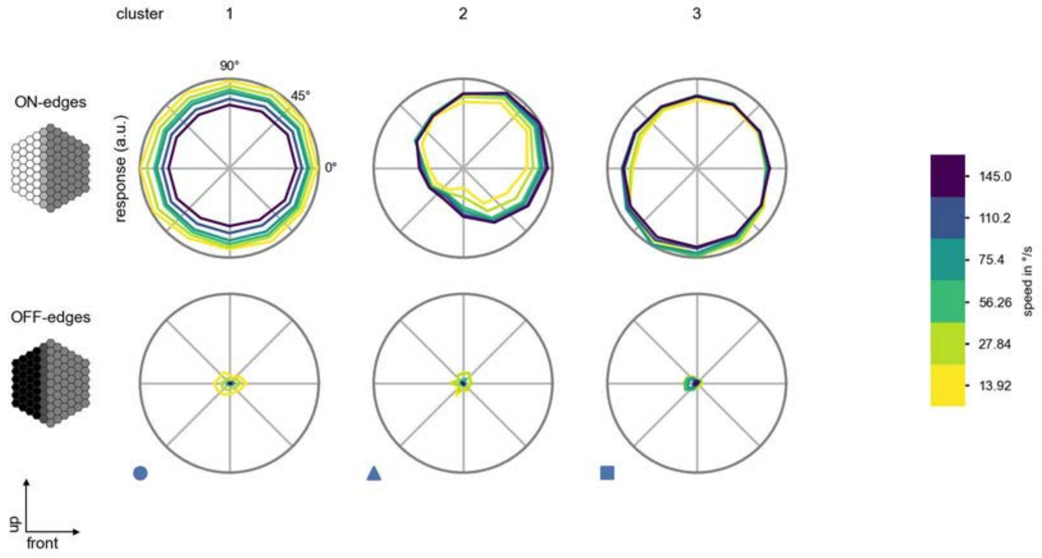

CT1(M10) - Figure 7: **Peak responses to moving edges from task-optimal models.** The top row shows peak responses to moving ON-edges, the bottom row shows peak responses to moving OFF-edges of varying speeds from 13.92°/s to 145°/s (yellow to dark blue). The edge-stimuli move in different directions from 0 to 360 degrees and at different speeds. Responses from the task-optimal model in the respective cluster.

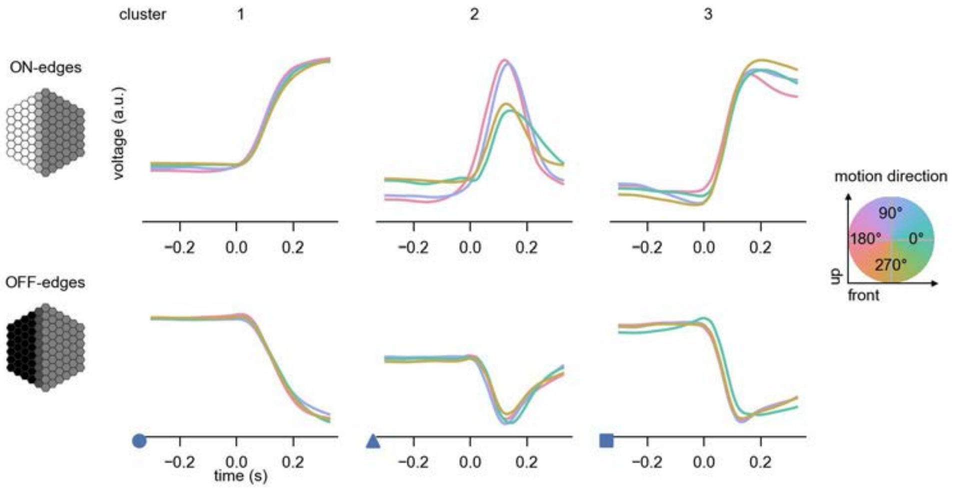

CT1(M10) - Figure 8: **Responses to moving edges from task-optimal models.** Responses to moving ON-edges (top row) and to moving OFF-edges (bottom row). Edges move in different directions from 0 to 360 degrees and at different speeds. Responses are from the task-optimal model in the respective cluster. Edges moving at 75.4°/s in all cardinal directions (green 0°, blue 90°, red 180°, yellow 270°) from -22.5 to 22.5° visual angle.

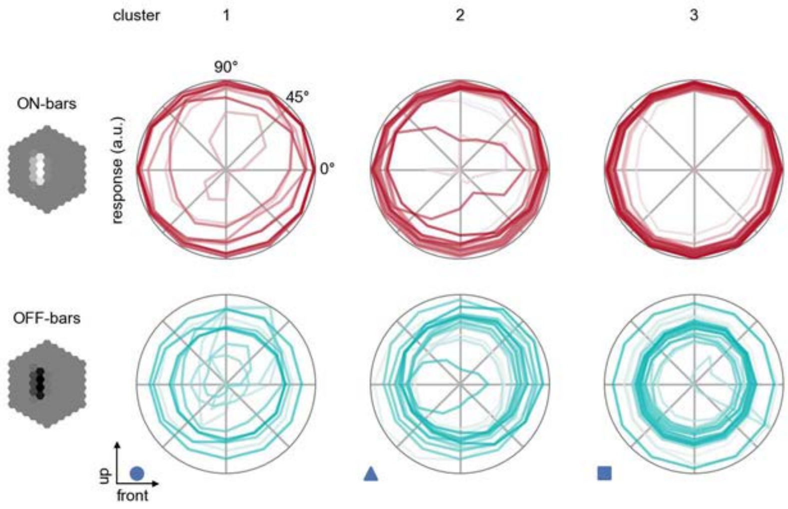

CT1(M10) - Figure 9: **Peak responses to moving bars.** The top row shows peak responses to moving ON-bars (red), the bottom row shows peak responses to moving OFF-bars (turqoise). The peak responses are averaged over bar-speeds. Bar-stimuli move in different directions from 0 to 360 degrees. The responses from the different models in the different clusters (columns) overlay. Responses from better task-performing models are more saturated.

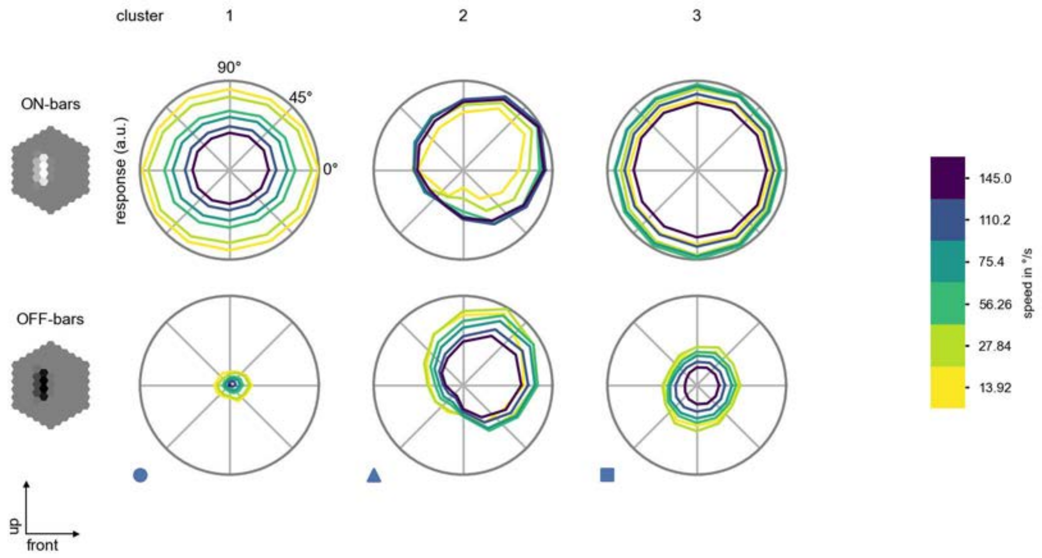

CT1(M10) - Figure 10: **Peak responses to moving bars from task-optimal models.** The top row shows peak responses to moving ON-bars, the bottom row shows peak responses to moving OFF-bars of varying speeds from 13.92°/s to 145°/s (yellow to dark blue). The bar-stimuli move in different directions from 0 to 360 degrees and at different speeds. Responses from the task-optimal model in the respective cluster.

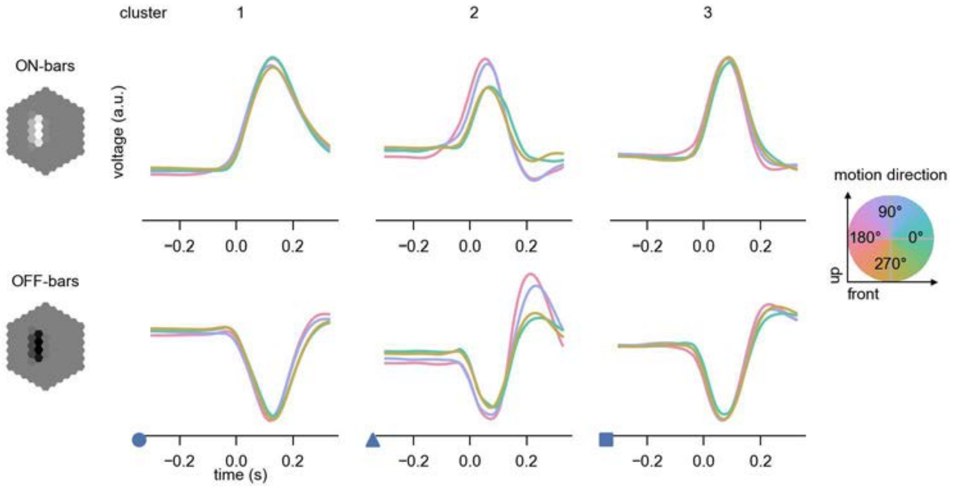

CT1(M10) - Figure 11: **Responses to moving bars from task-optimal models.** Responses to moving ON-bars (top row) and to moving OFF-bars (bottom row). Bars move in different directions from 0 to 360 degrees and at different speeds. Responses are from the task-optimal model in the respective cluster. Bars moving at 75.4°/s in all cardinal directions (green 0°, blue 90°, red 180°, yellow 270°) from -22.5 to 22.5° visual angle.

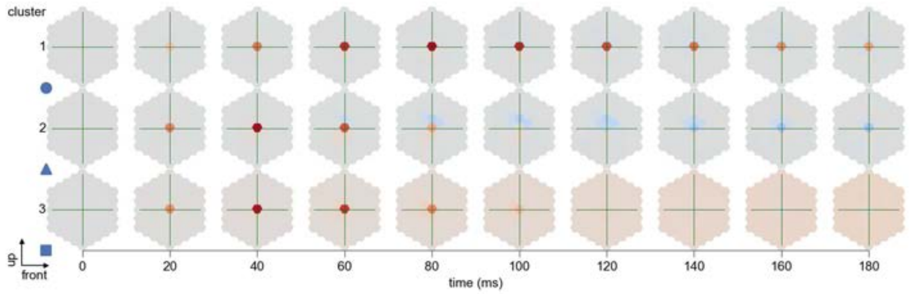

CT1(M10) - Figure 12: **Spatio-temporal receptive field.** Responses of the central cell to ON-impulses (5 ms) at single-ommatidium flash locations. The flash occurs at second zero. Responses from the task-optimal model of the respective cluster (rows). Red indicates depolarization, blue indicates hyperpolarization.

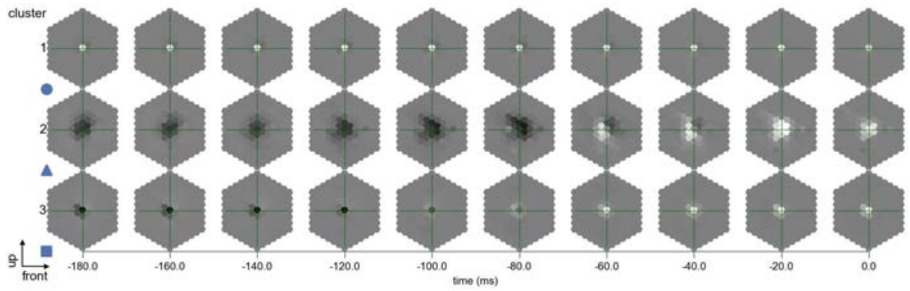

CT1(M10) - Figure 13: **Maximally excitatory stimuli.** Each row presents the regularized naturalistic-stimulus from the Sintel dataset that maximizes the cell type's central column response at second zero in the task-optimal model of the respective cluster (rows).

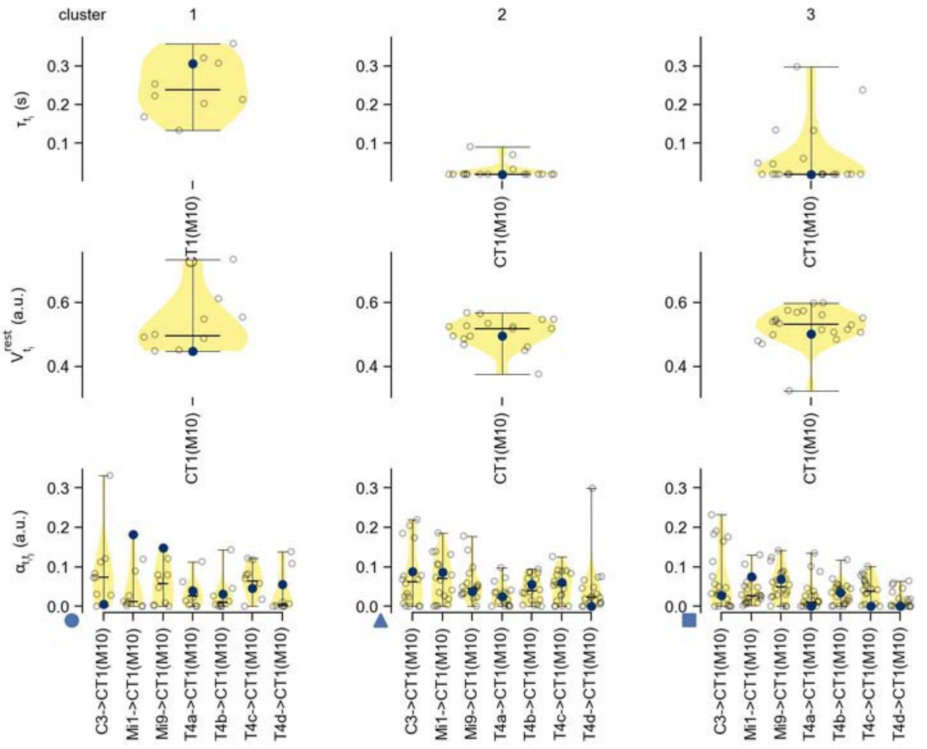

CT1(M10) - Figure 14: **Task-constrained parameters.** Each column shows the parameters inferred within the respective cluster. First row: learned time constants of the cell type. Second row: resting potentials of the cell type. Third row: scaling factors for the convolutional filters. The blue scatter represents the parameters from the task-optimal model within the cluster.

21 Mi1

← Cell types

Figures

1 Anatomical receptive fields. 147
2 Anatomical projective fields. 148
3 Clustering of the responses to naturalistic stimuli. 148
4 Responses to flashes. 149
5 Cluster-average responses to single-ommatidium flashes. 149
6 Peak responses to moving edges. 150
7 Peak responses to moving edges from task-optimal models. 150
8 Responses to moving edges from task-optimal models. 151
9 Peak responses to moving bars. 151
10 Peak responses to moving bars from task-optimal models. 152
11 Responses to moving bars from task-optimal models. 152
12 Spatio-temporal receptive field. 152
13 Maximally excitatory stimuli. 153
14 Task-constrained parameters. 153

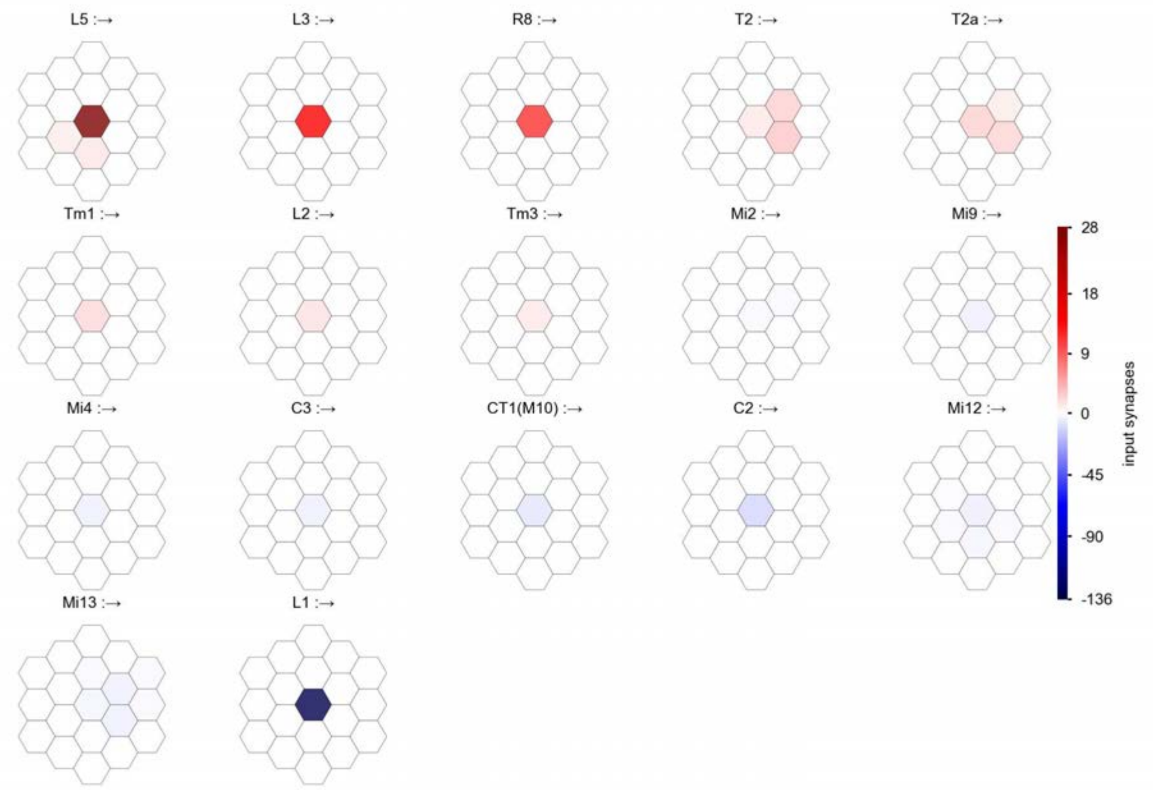

Mi1 - Figure 1: **Anatomical receptive fields.** Each colored hexagon is an input connection, with the connection strength characterized by the average number of synapses that we count from the EM reconstruction. Red indicates excitatory synapses, blue indicates inhibitory synapses from inferred signs. Filters in the order of their total number of synapses.

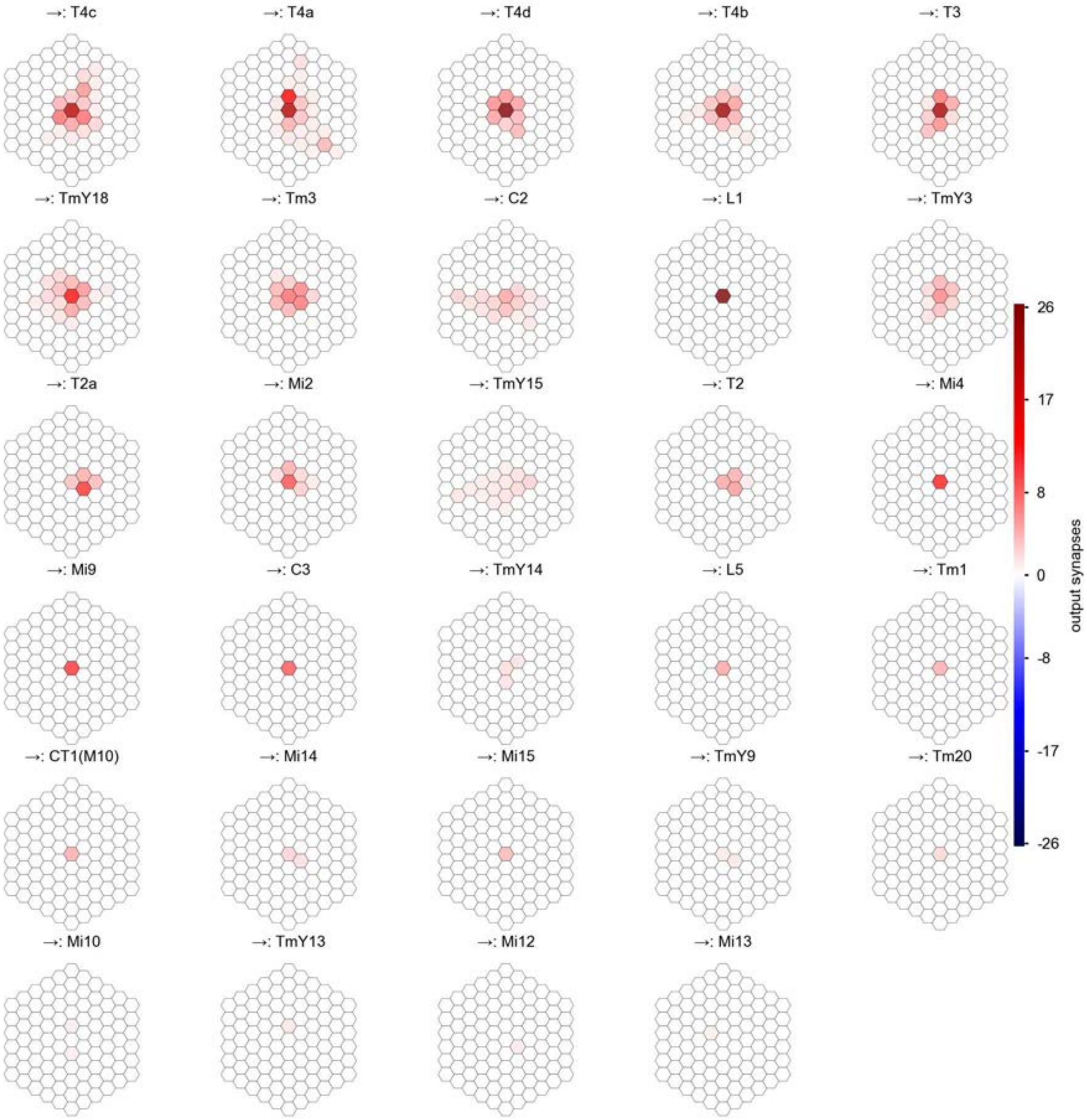

Mi1 - Figure 2: **Anatomical projective fields.** Each colored hexagon is an output connection, with the connection strength characterized by the average number of synapses that we count from the EM reconstruction. Red indicates excitatory synapses, blue indicates inhibitory synapses from inferred signs. Filters in the order of their total number of synapses.

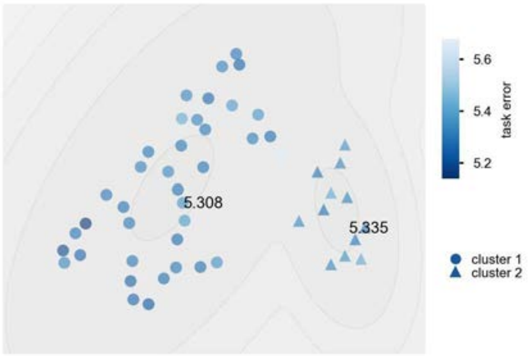

Mi1 - Figure 3: **Clustering of the responses to naturalistic stimuli.** Clustering of the 50 models based on the cell type responses to naturalistic scenes from the Sintel dataset. Scatterpoints represent individual models colored by their task error.

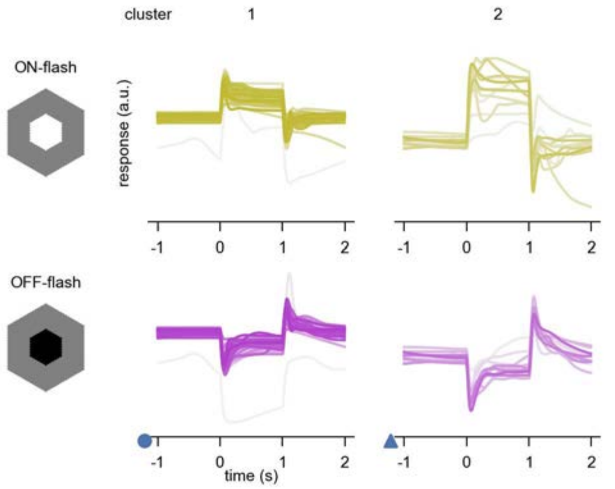

Mi1 - Figure 4: **Responses to flashes.** The top row shows responses to ON-flashes (yellow), the bottom row shows responses to OFF-flashes (magenta). The responses from the 50 different models that are separated into the different clusters (columns) overlay, with better task-performing models on top. Responses from better task-performing models are more saturated. The circular flashes (1s) cover 6 ommatidia in radius and are presented at time zero. Before and after, a grey-stimulus leads to a stationary state of the network.

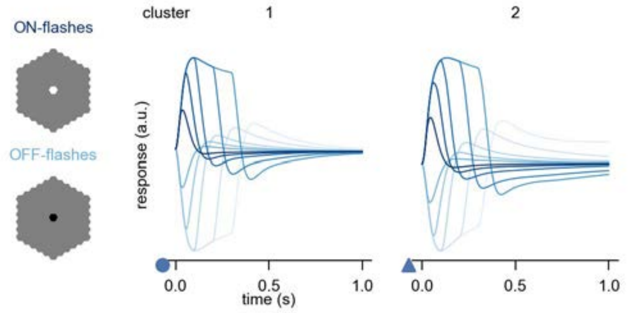

Mi1 - Figure 5: **Cluster-average responses to single-ommatidium flashes.** Responses to single-ommatidium ON-flashes (dark blue shades) and single-ommatidium OFF-flashes (light blue shades) of 20ms, 50ms, 100ms, 200ms, 300ms duration. The flashes occur at second zero.

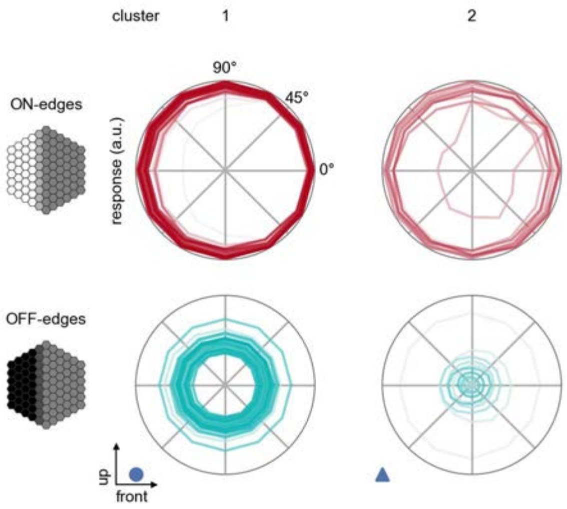

Mi1 - Figure 6: **Peak responses to moving edges.** The top row shows peak responses to moving ON-edges (red), the bottom row shows peak responses to moving OFF-edges (turqoise). The peak responses are averaged over edge-speeds. Edge-stimuli move in different directions from 0 to 360 degrees. The responses from the different models in the different clusters (columns) overlay. Responses from better task-performing models are more saturated.

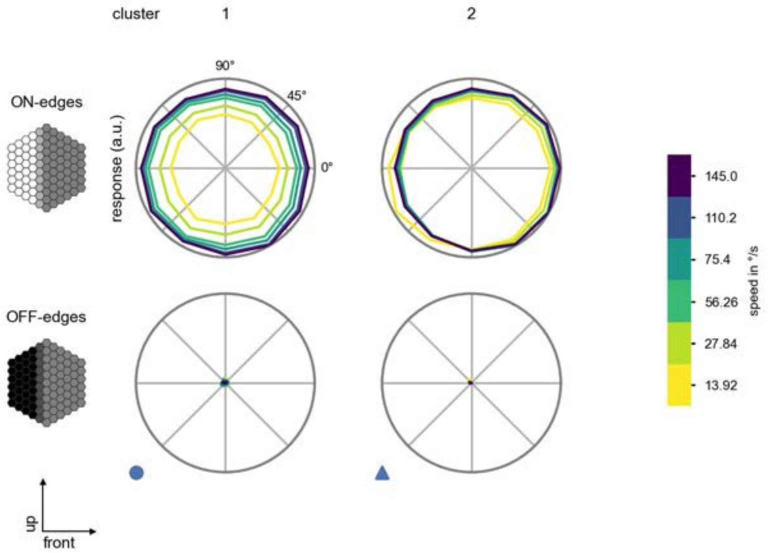

Mi1 - Figure 7: **Peak responses to moving edges from task-optimal models.** The top row shows peak responses to moving ON-edges, the bottom row shows peak responses to moving OFF-edges of varying speeds from 13.92°/s to 145°/s (yellow to dark blue). The edge-stimuli move in different directions from 0 to 360 degrees and at different speeds. Responses from the task-optimal model in the respective cluster.

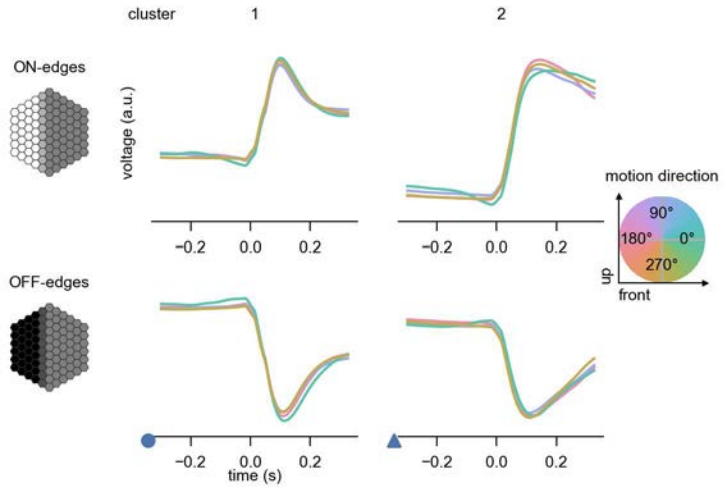

Mi1 - Figure 8: **Responses to moving edges from task-optimal models.** Responses to moving ON-edges (top row) and to moving OFF-edges (bottom row). Edges move in different directions from 0 to 360 degrees and at different speeds. Responses are from the task-optimal model in the respective cluster. Edges moving at 75.4°/s in all cardinal directions (green 0°, blue 90°, red 180°, yellow 270°) from -22.5 to 22.5° visual angle.

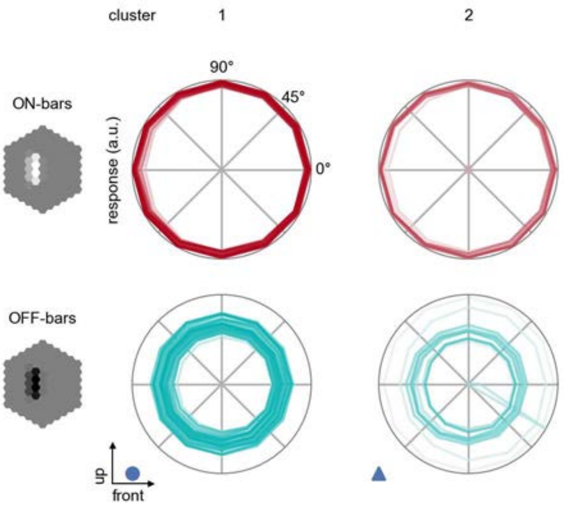

Mi1 - Figure 9: **Peak responses to moving bars.** The top row shows peak responses to moving ON-bars (red), the bottom row shows peak responses to moving OFF-bars (turqoise). The peak responses are averaged over bar-speeds. Bar-stimuli move in different directions from 0 to 360 degrees. The responses from the different models in the different clusters (columns) overlay. Responses from better task-performing models are more saturated.

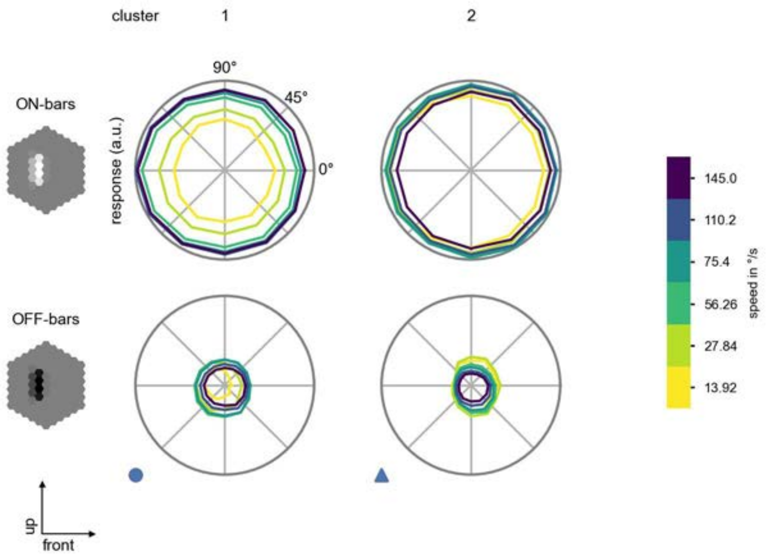

Mi1 - Figure 10: **Peak responses to moving bars from task-optimal models.** The top row shows peak responses to moving ON-bars, the bottom row shows peak responses to moving OFF-bars of varying speeds from 13.92°/s to 145°/s (yellow to dark blue). The bar-stimuli move in different directions from 0 to 360 degrees and at different speeds. Responses from the task-optimal model in the respective cluster.

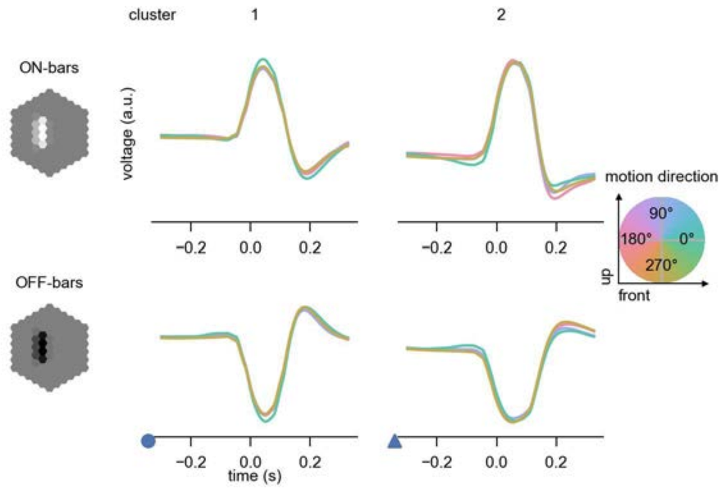

Mi1 - Figure 11: **Responses to moving bars from task-optimal models.** Responses to moving ON-bars (top row) and to moving OFF-bars (bottom row). Bars move in different directions from 0 to 360 degrees and at different speeds. Responses are from the task-optimal model in the respective cluster. Bars moving at 75.4°/s in all cardinal directions (green 0°, blue 90°, red 180°, yellow 270°) from -22.5 to 22.5° visual angle.

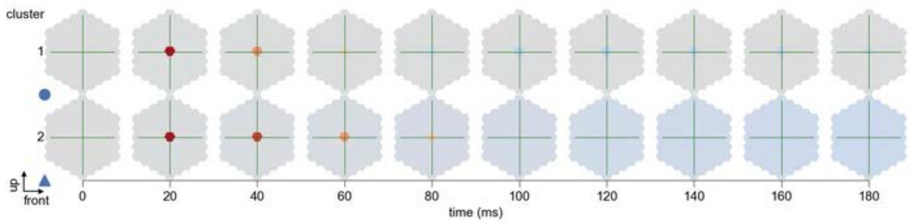

Mi1 - Figure 12: **Spatio-temporal receptive field.** Responses of the central cell to ON-impulses (5 ms) at single-ommatidium flash locations. The flash occurs at second zero. Responses from the task-optimal model of the respective cluster (rows). Red indicates depolarization, blue indicates hyperpolarization.

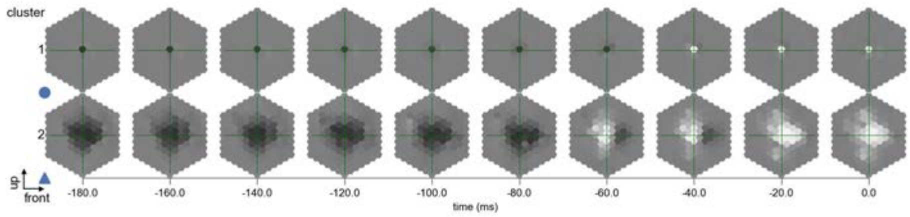

Mi1 - Figure 13: **Maximally excitatory stimuli.** Each row presents the regularized naturalistic-stimulus from the Sintel dataset that maximizes the cell type's central column response at second zero in the task-optimal model of the respective cluster (rows).

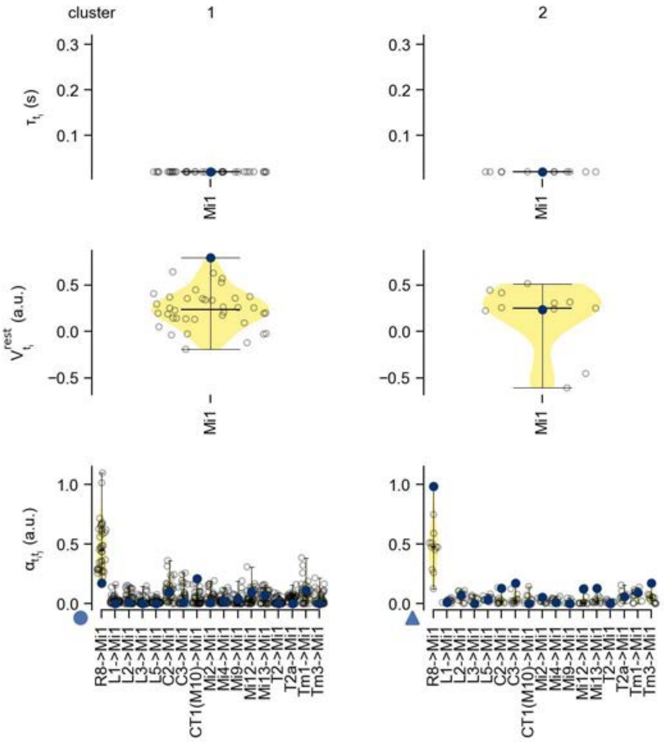

Mi1 - Figure 14: **Task-constrained parameters.** Each column shows the parameters inferred within the respective cluster. First row: learned time constants of the cell type. Second row: resting potentials of the cell type. Third row: scaling factors for the convolutional filters. The blue scatter represents the parameters from the task-optimal model within the cluster.

22 Mi2

← Cell types

Figures

1 Anatomical receptive fields. 154
2 Anatomical projective fields. 155
3 Clustering of the responses to naturalistic stimuli. 155
4 Responses to flashes. 155
5 Cluster-average responses to single-ommatidium flashes. 156
6 Peak responses to moving edges. 156
7 Peak responses to moving edges from task-optimal models. 156
8 Responses to moving edges from task-optimal models. 157
9 Peak responses to moving bars. 157
10 Peak responses to moving bars from task-optimal models. 157
11 Responses to moving bars from task-optimal models. 158
12 Spatio-temporal receptive field. 158
13 Maximally excitatory stimuli. 159
14 Task-constrained parameters. 159

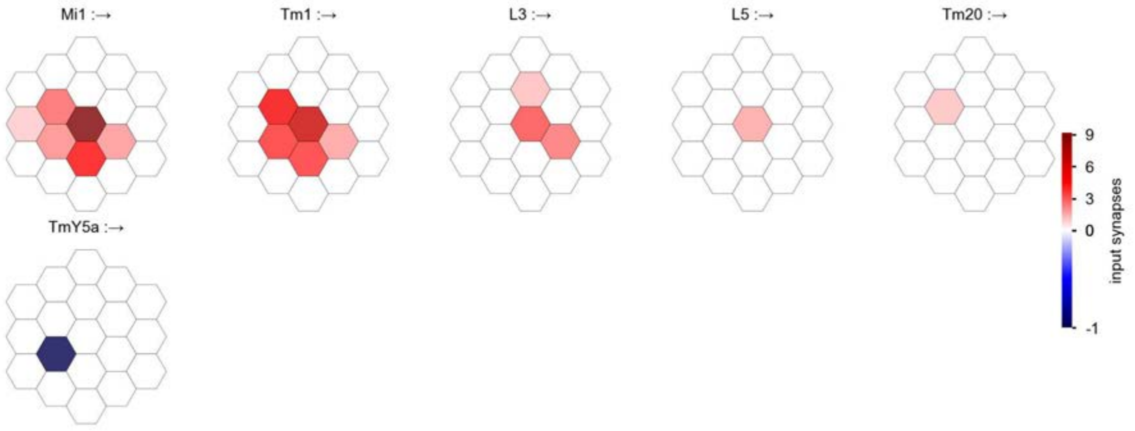

Mi2 - Figure 1: **Anatomical receptive fields.** Each colored hexagon is an input connection, with the connection strength characterized by the average number of synapses that we count from the EM reconstruction. Red indicates excitatory synapses, blue indicates inhibitory synapses from inferred signs. Filters in the order of their total number of synapses.

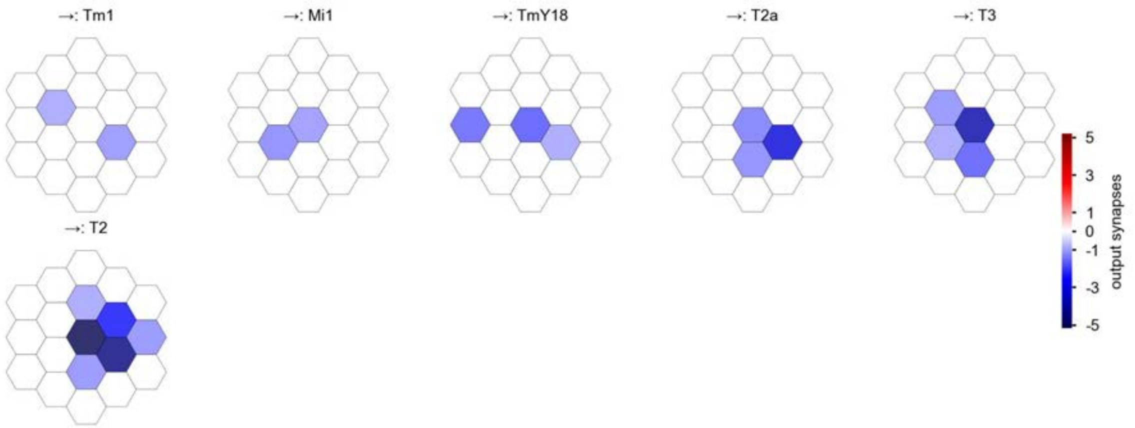

Mi2 - Figure 2: **Anatomical projective fields.** Each colored hexagon is an output connection, with the connection strength characterized by the average number of synapses that we count from the EM reconstruction. Red indicates excitatory synapses, blue indicates inhibitory synapses from inferred signs. Filters in the order of their total number of synapses.

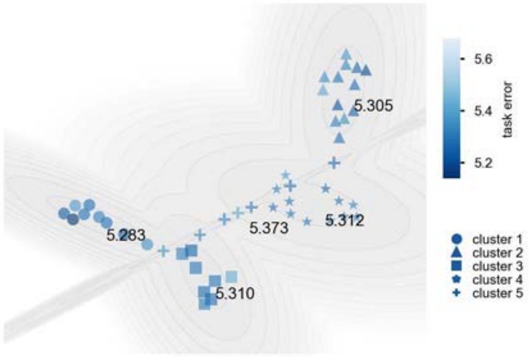

Mi2 - Figure 3: **Clustering of the responses to naturalistic stimuli.** Clustering of the 50 models based on the cell type responses to naturalistic scenes from the Sintel dataset. Scatterpoints represent individual models colored by their task error.

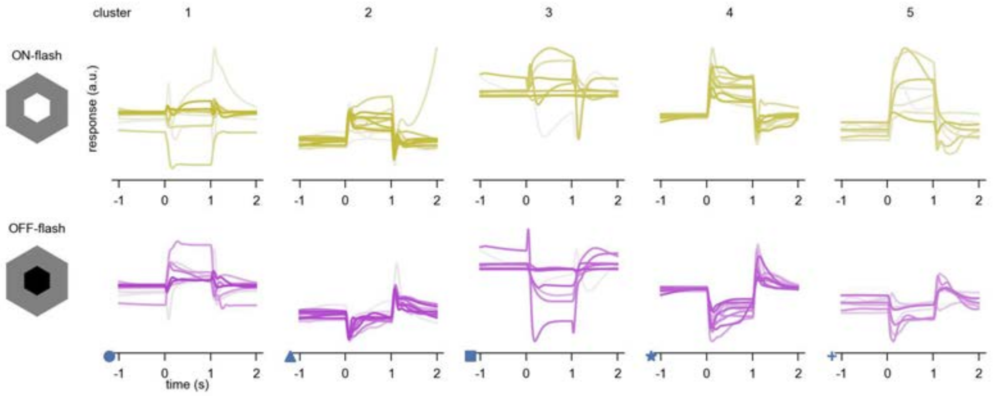

Mi2 - Figure 4: **Responses to flashes.** The top row shows responses to ON-flashes (yellow), the bottom row shows responses to OFF-flashes (magenta). The responses from the 50 different models that are separated into the different clusters (columns) overlay, with better task-performing models on top. Responses from better task-performing models are more saturated. The circular flashes (1s) cover 6 ommatidia in radius and are presented at time zero. Before and after, a grey-stimulus leads to a stationary state of the network.

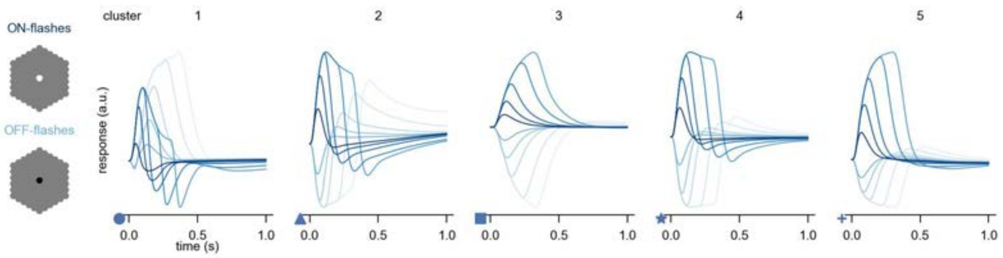

Mi2 - Figure 5: **Cluster-average responses to single-ommatidium flashes.** Responses to single-ommatidium ON-flashes (dark blue shades) and single-ommatidium OFF-flashes (light blue shades) of 20ms, 50ms, 100ms, 200ms, 300ms duration. The flashes occur at second zero.

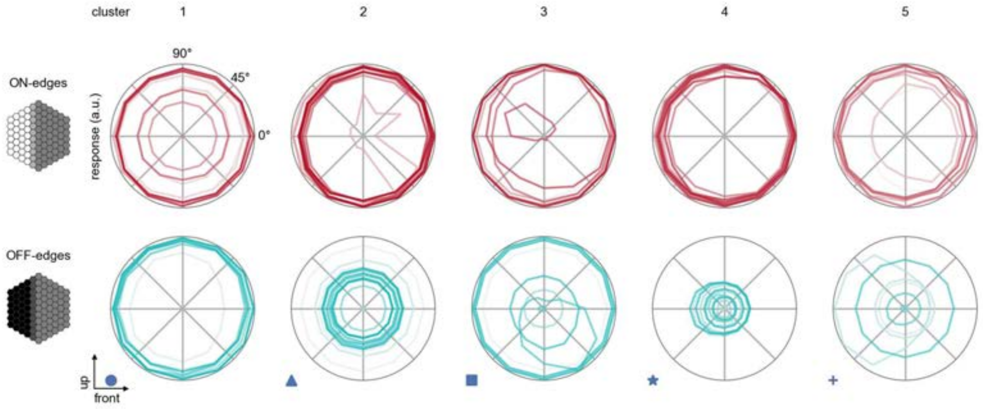

Mi2 - Figure 6: **Peak responses to moving edges.** The top row shows peak responses to moving ON-edges (red), the bottom row shows peak responses to moving OFF-edges (turqoise). The peak responses are averaged over edge-speeds. Edge-stimuli move in different directions from 0 to 360 degrees. The responses from the different models in the different clusters (columns) overlay. Responses from better task-performing models are more saturated.

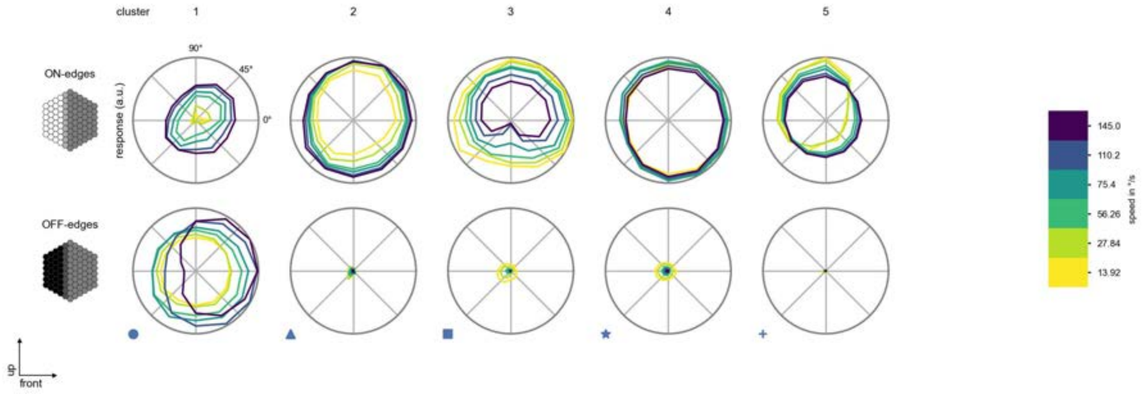

Mi2 - Figure 7: **Peak responses to moving edges from task-optimal models.** The top row shows peak responses to moving ON-edges, the bottom row shows peak responses to moving OFF-edges of varying speeds from 13.92°/s to 145°/s (yellow to dark blue). The edge-stimuli move in different directions from 0 to 360 degrees and at different speeds. Responses from the task-optimal model in the respective cluster.

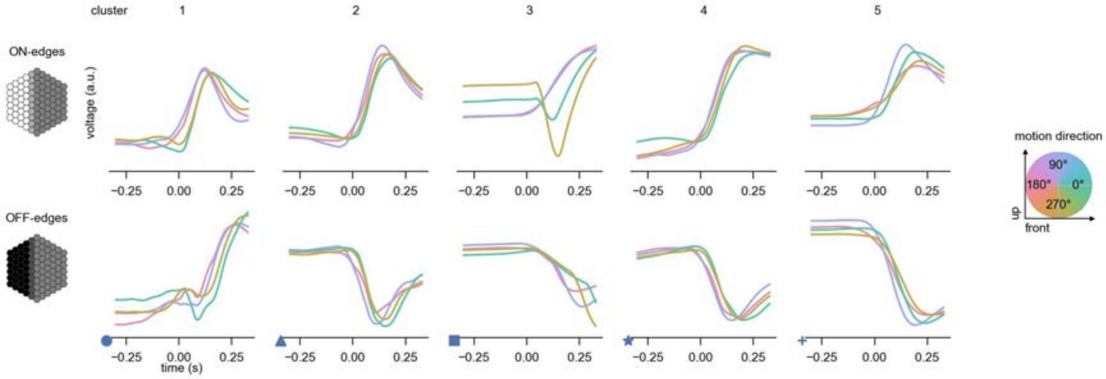

Mi2 - Figure 8: **Responses to moving edges from task-optimal models.** Responses to moving ON-edges (top row) and to moving OFF-edges (bottom row). Edges move in different directions from 0 to 360 degrees and at different speeds. Responses are from the task-optimal model in the respective cluster. Edges moving at 75.4°/s in all cardinal directions (green 0°, blue 90°, red 180°, yellow 270°) from -22.5 to 22.5° visual angle.

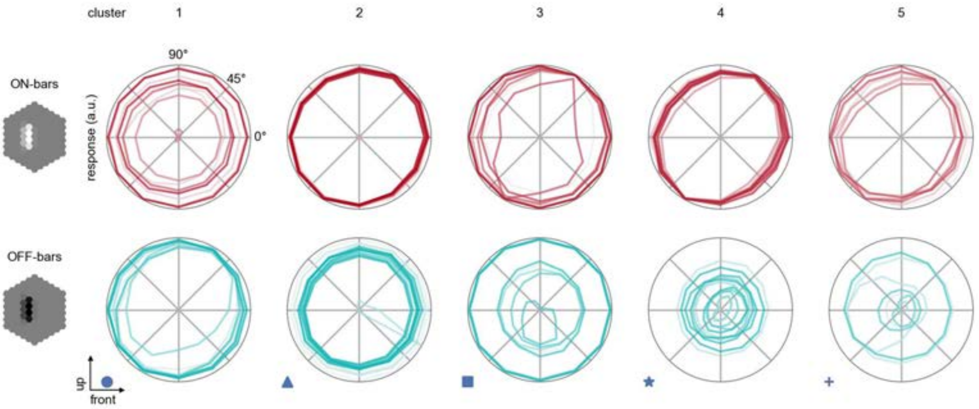

Mi2 - Figure 9: **Peak responses to moving bars.** The top row shows peak responses to moving ON-bars (red), the bottom row shows peak responses to moving OFF-bars (turqoise). The peak responses are averaged over bar-speeds. Bar-stimuli move in different directions from 0 to 360 degrees. The responses from the different models in the different clusters (columns) overlay. Responses from better task-performing models are more saturated.

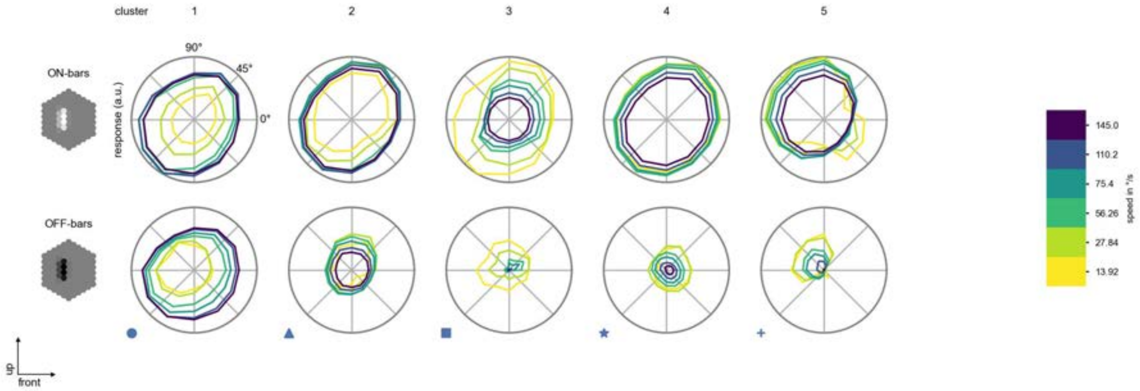

Mi2 - Figure 10: **Peak responses to moving bars from task-optimal models.** The top row shows peak responses to moving ON-bars, the bottom row shows peak responses to moving OFF-bars of varying speeds from 13.92°/s to 145°/s (yellow to dark blue). The bar-stimuli move in different directions from 0 to 360 degrees and at different speeds. Responses from the task-optimal model in the respective cluster.

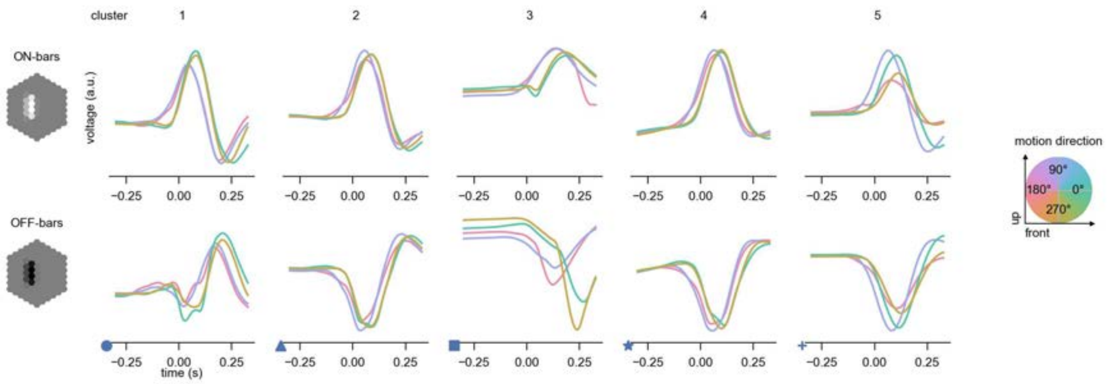

Mi2 - Figure 11: **Responses to moving bars from task-optimal models.** Responses to moving ON-bars (top row) and to moving OFF-bars (bottom row). Bars move in different directions from 0 to 360 degrees and at different speeds. Responses are from the task-optimal model in the respective cluster. Bars moving at 75.4°/s in all cardinal directions (green 0°, blue 90°, red 180°, yellow 270°) from -22.5 to 22.5° visual angle.

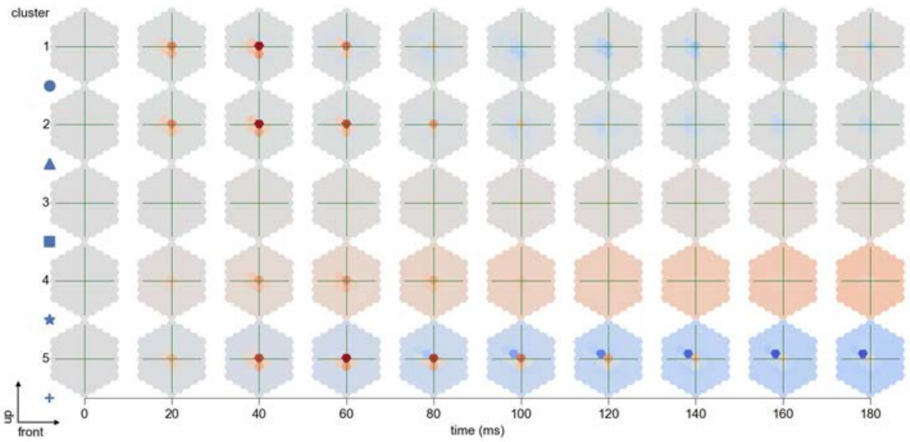

Mi2 - Figure 12: **Spatio-temporal receptive field.** Responses of the central cell to ON-impulses (5 ms) at single-ommatidium flash locations. The flash occurs at second zero. Responses from the task-optimal model of the respective cluster (rows). Red indicates depolarization, blue indicates hyperpolarization.

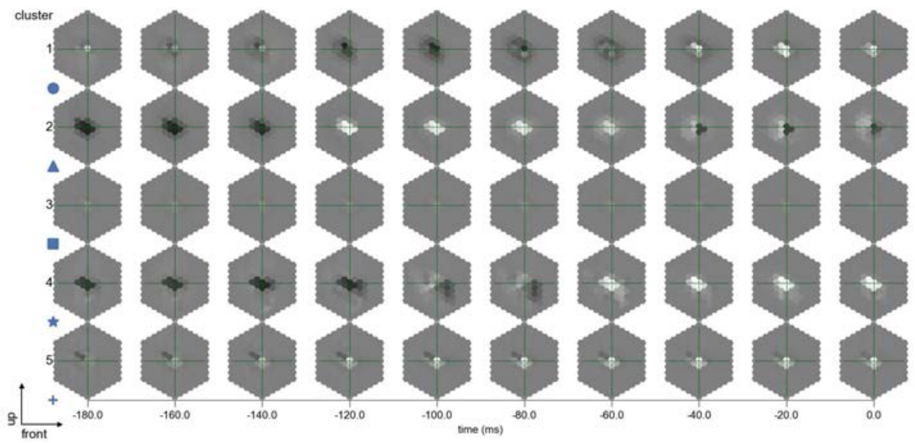

Mi2 - Figure 13: **Maximally excitatory stimuli.** Each row presents the regularized naturalistic-stimulus from the Sintel dataset that maximizes the cell type's central column response at second zero in the task-optimal model of the respective cluster (rows).

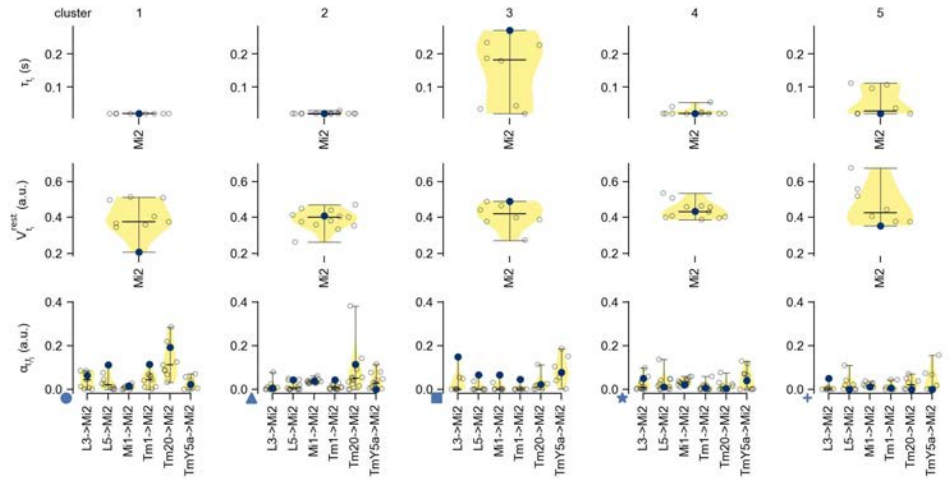

Mi2 - Figure 14: **Task-constrained parameters.** Each column shows the parameters inferred within the respective cluster. First row: learned time constants of the cell type. Second row: resting potentials of the cell type. Third row: scaling factors for the convolutional filters. The blue scatter represents the parameters from the task-optimal model within the cluster.

23 Mi3

← Cell types

Figures

1 Anatomical receptive fields. . . . 160
2 Anatomical projective fields. . . . 160
3 Clustering of the responses to naturalistic stimuli. . . . 161
4 Responses to flashes. . . . 161
5 Cluster-average responses to single-ommatidium flashes. . . . 161
6 Peak responses to moving edges. . . . 162
7 Peak responses to moving edges from task-optimal models. . . . 162
8 Responses to moving edges from task-optimal models. . . . 163
9 Peak responses to moving bars. . . . 163
10 Peak responses to moving bars from task-optimal models. . . . 164
11 Responses to moving bars from task-optimal models. . . . 164
12 Spatio-temporal receptive field. . . . 165
13 Maximally excitatory stimuli. . . . 165
14 Task-constrained parameters. . . . 166

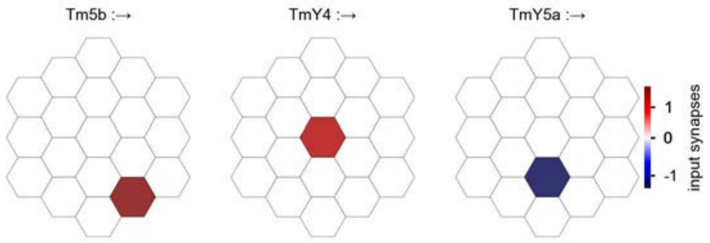

Mi3 - Figure 1: **Anatomical receptive fields.** Each colored hexagon is an input connection, with the connection strength characterized by the average number of synapses that we count from the EM reconstruction. Red indicates excitatory synapses, blue indicates inhibitory synapses from inferred signs. Filters in the order of their total number of synapses.

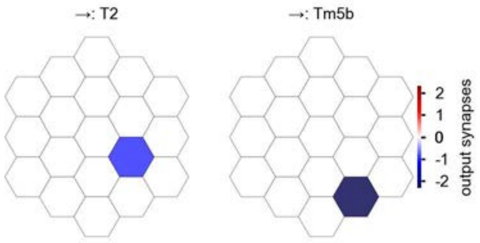

Mi3 - Figure 2: **Anatomical projective fields.** Each colored hexagon is an output connection, with the connection strength characterized by the average number of synapses that we count from the EM reconstruction. Red indicates excitatory synapses, blue indicates inhibitory synapses from inferred signs. Filters in the order of their total number of synapses.

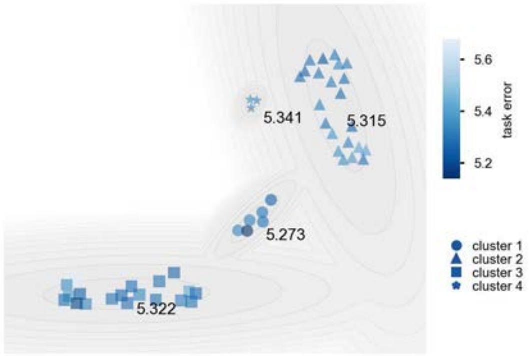

Mi3 - Figure 3: **Clustering of the responses to naturalistic stimuli.** Clustering of the 50 models based on the cell type responses to naturalistic scenes from the Sintel dataset. Scatterpoints represent individual models colored by their task error.

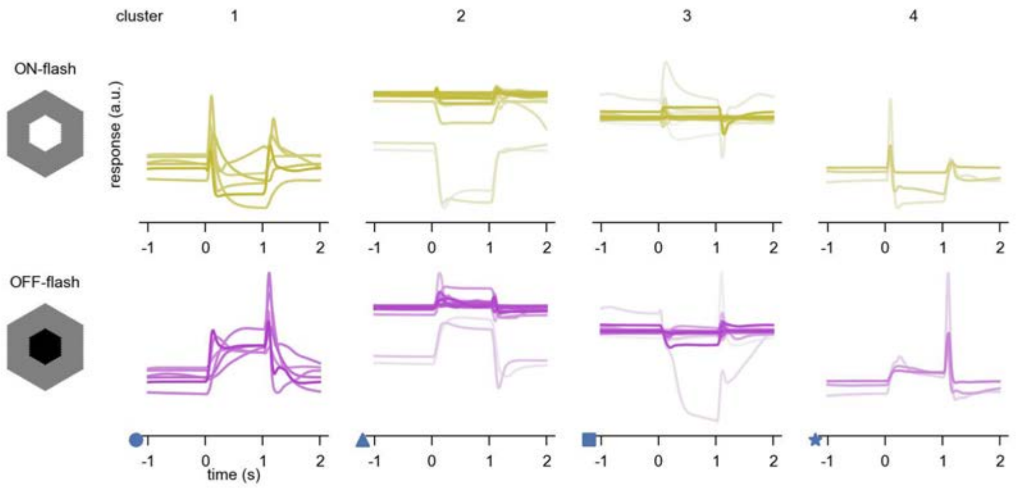

Mi3 - Figure 4: **Responses to flashes.** The top row shows responses to ON-flashes (yellow), the bottom row shows responses to OFF-flashes (magenta). The responses from the 50 different models that are separated into the different clusters (columns) overlay, with better task-performing models on top. Responses from better task-performing models are more saturated. The circular flashes (1s) cover 6 ommatidia in radius and are presented at time zero. Before and after, a grey-stimulus leads to a stationary state of the network.

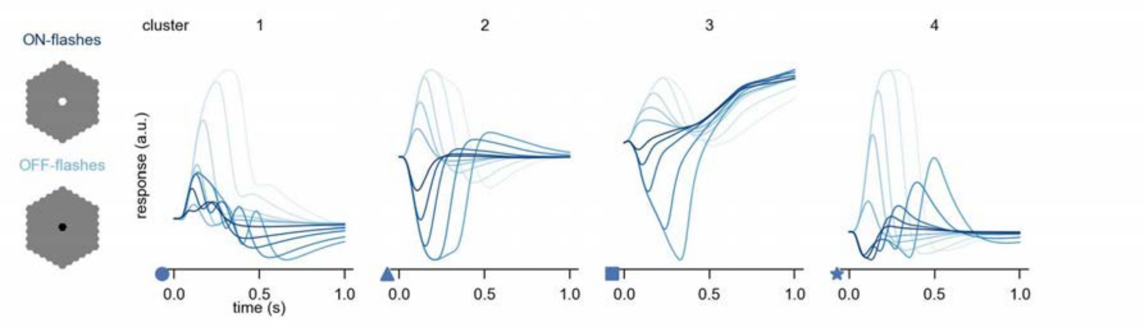

Mi3 - Figure 5: **Cluster-average responses to single-ommatidium flashes.** Responses to single-ommatidium ON-flashes (dark blue shades) and single-ommatidium OFF-flashes (light blue shades) of 20ms, 50ms, 100ms, 200ms, 300ms duration. The flashes occur at second zero.

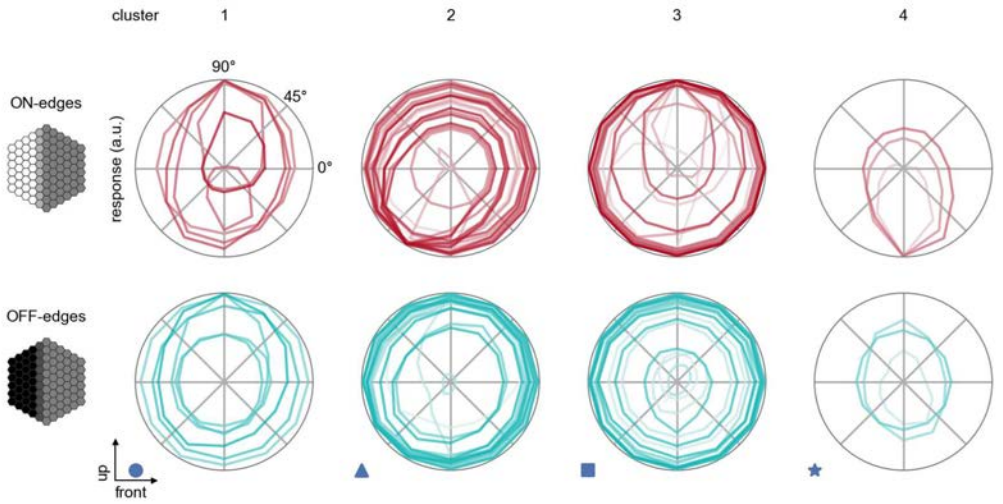

Mi3 - Figure 6: **Peak responses to moving edges.** The top row shows peak responses to moving ON-edges (red), the bottom row shows peak responses to moving OFF-edges (turqoise). The peak responses are averaged over edge-speeds. Edge-stimuli move in different directions from 0 to 360 degrees. The responses from the different models in the different clusters (columns) overlay. Responses from better task-performing models are more saturated.

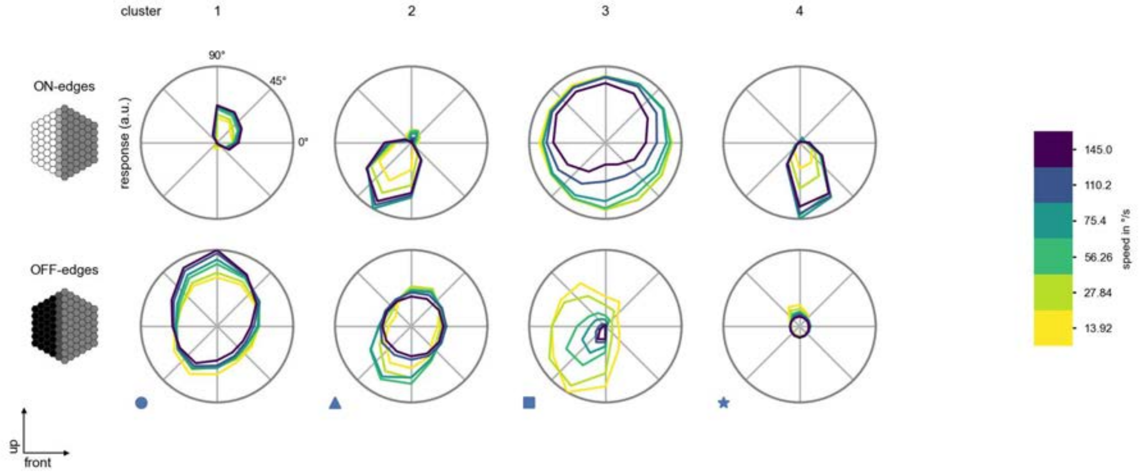

Mi3 - Figure 7: **Peak responses to moving edges from task-optimal models.** The top row shows peak responses to moving ON-edges, the bottom row shows peak responses to moving OFF-edges of varying speeds from 13.92°/s to 145°/s (yellow to dark blue). The edge-stimuli move in different directions from 0 to 360 degrees and at different speeds. Responses from the task-optimal model in the respective cluster.

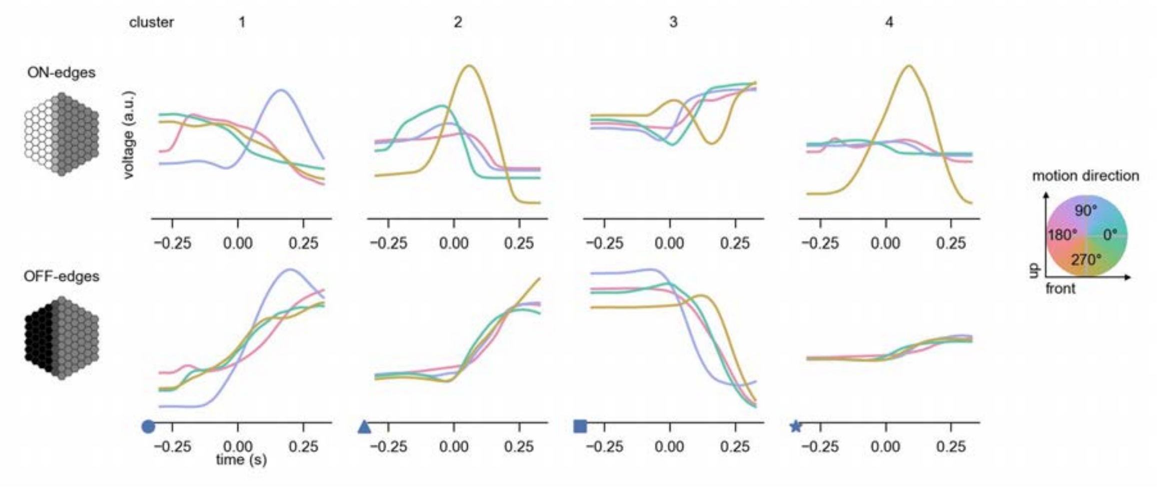

Mi3 - Figure 8: **Responses to moving edges from task-optimal models.** Responses to moving ON-edges (top row) and to moving OFF-edges (bottom row). Edges move in different directions from 0 to 360 degrees and at different speeds. Responses are from the task-optimal model in the respective cluster. Edges moving at 75.4°/s in all cardinal directions (green 0°, blue 90°, red 180°, yellow 270°) from -22.5 to 22.5° visual angle.

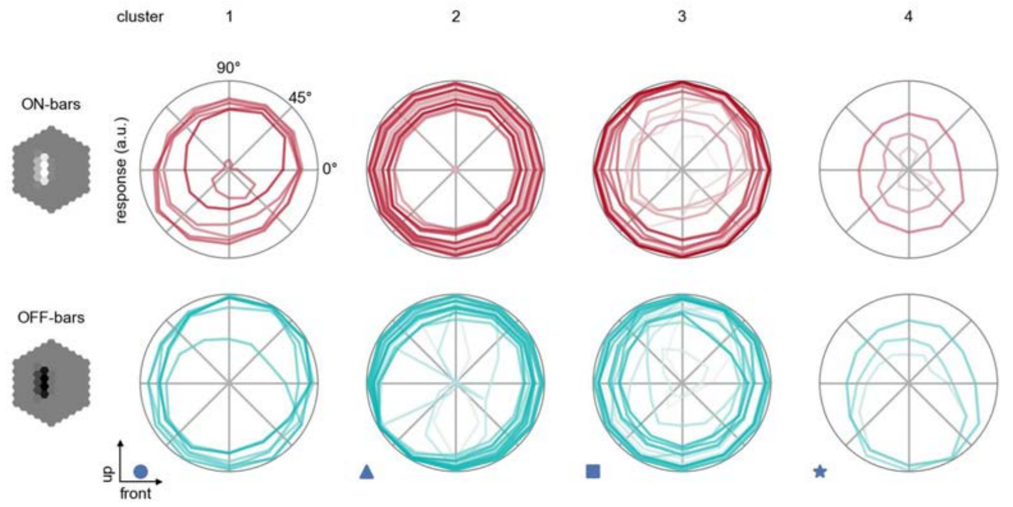

Mi3 - Figure 9: **Peak responses to moving bars.** The top row shows peak responses to moving ON-bars (red), the bottom row shows peak responses to moving OFF-bars (turqoise). The peak responses are averaged over bar-speeds. Bar-stimuli move in different directions from 0 to 360 degrees. The responses from the different models in the different clusters (columns) overlay. Responses from better task-performing models are more saturated.

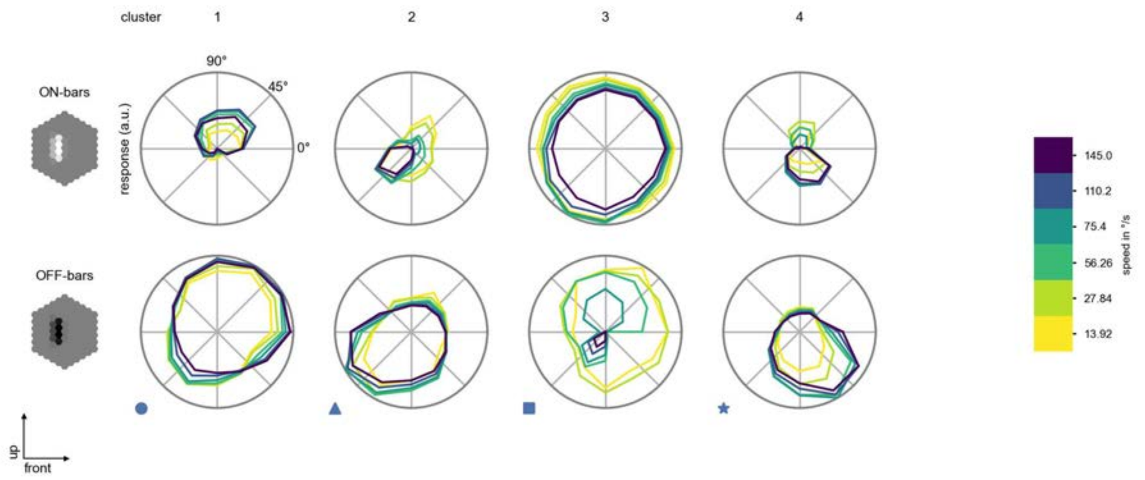

Mi3 - Figure 10: **Peak responses to moving bars from task-optimal models.** The top row shows peak responses to moving ON-bars, the bottom row shows peak responses to moving OFF-bars of varying speeds from 13.92°/s to 145°/s (yellow to dark blue). The bar-stimuli move in different directions from 0 to 360 degrees and at different speeds. Responses from the task-optimal model in the respective cluster.

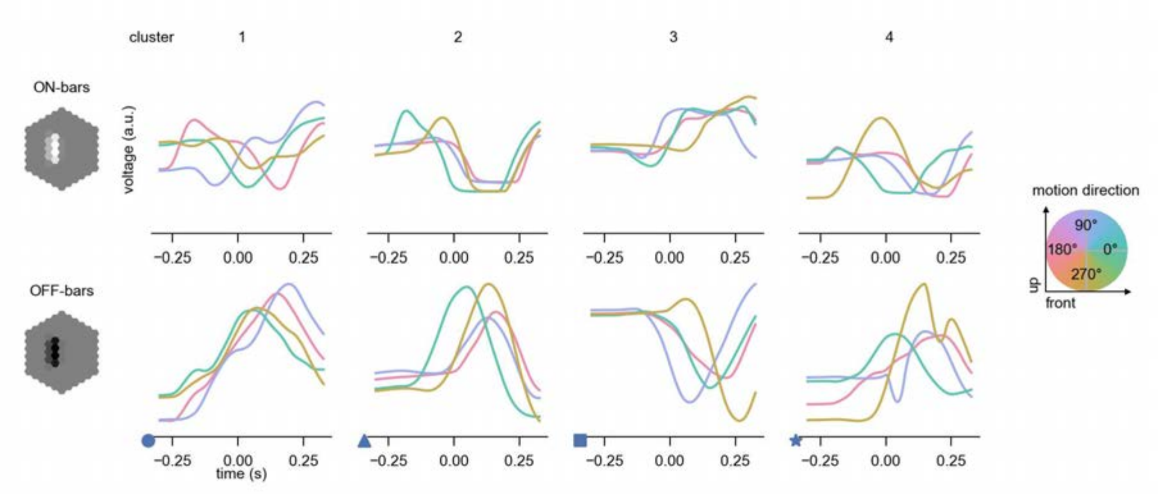

Mi3 - Figure 11: **Responses to moving bars from task-optimal models.** Responses to moving ON-bars (top row) and to moving OFF-bars (bottom row). Bars move in different directions from 0 to 360 degrees and at different speeds. Responses are from the task-optimal model in the respective cluster. Bars moving at 75.4°/s in all cardinal directions (green 0°, blue 90°, red 180°, yellow 270°) from -22.5 to 22.5° visual angle.

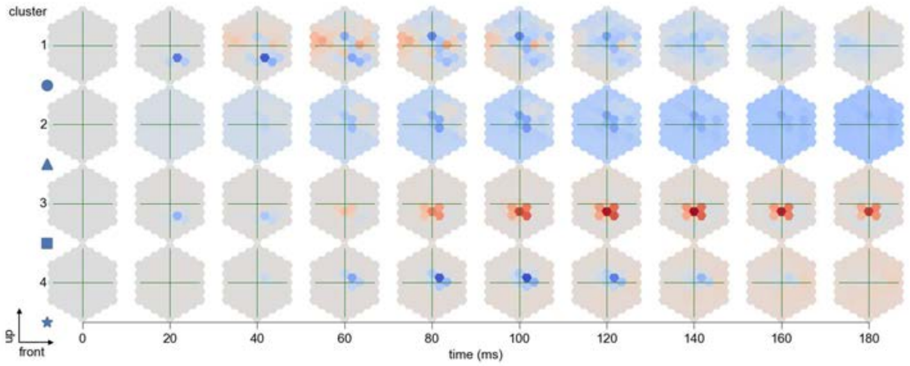

Mi3 - Figure 12: **Spatio-temporal receptive field.** Responses of the central cell to ON-impulses (5 ms) at single-ommatidium flash locations. The flash occurs at second zero. Responses from the task-optimal model of the respective cluster (rows). Red indicates depolarization, blue indicates hyperpolarization.

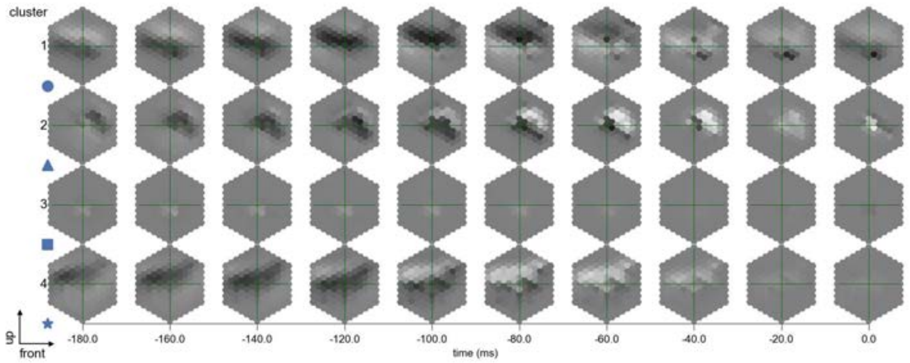

Mi3 - Figure 13: **Maximally excitatory stimuli.** Each row presents the regularized naturalistic-stimulus from the Sintel dataset that maximizes the cell type's central column response at second zero in the task-optimal model of the respective cluster (rows).

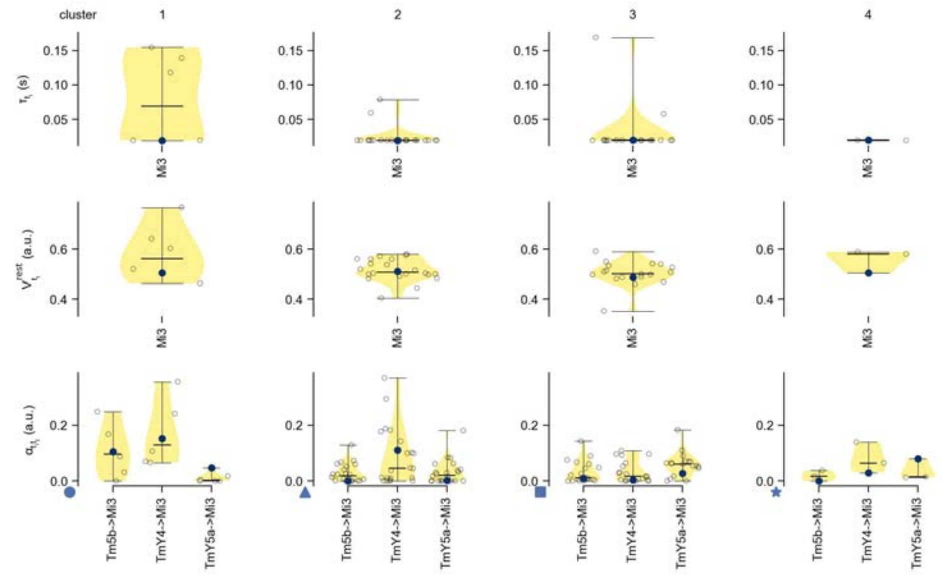

Mi3 - Figure 14: **Task-constrained parameters.** Each column shows the parameters inferred within the respective cluster. First row: learned time constants of the cell type. Second row: resting potentials of the cell type. Third row: scaling factors for the convolutional filters. The blue scatter represents the parameters from the task-optimal model within the cluster.

24 Mi4

← Cell types

Figures

1 Anatomical receptive fields. . . . 167
2 Anatomical projective fields. . . . 168
3 Clustering of the responses to naturalistic stimuli. . . . 168
4 Responses to flashes. . . . 169
5 Cluster-average responses to single-ommatidium flashes. . . . 169
6 Peak responses to moving edges. . . . 170
7 Peak responses to moving edges from task-optimal models. . . . 170
8 Responses to moving edges from task-optimal models. . . . 171
9 Peak responses to moving bars. . . . 171
10 Peak responses to moving bars from task-optimal models. . . . 172
11 Responses to moving bars from task-optimal models. . . . 172
12 Spatio-temporal receptive field. . . . 172
13 Maximally excitatory stimuli. . . . 173
14 Task-constrained parameters. . . . 173

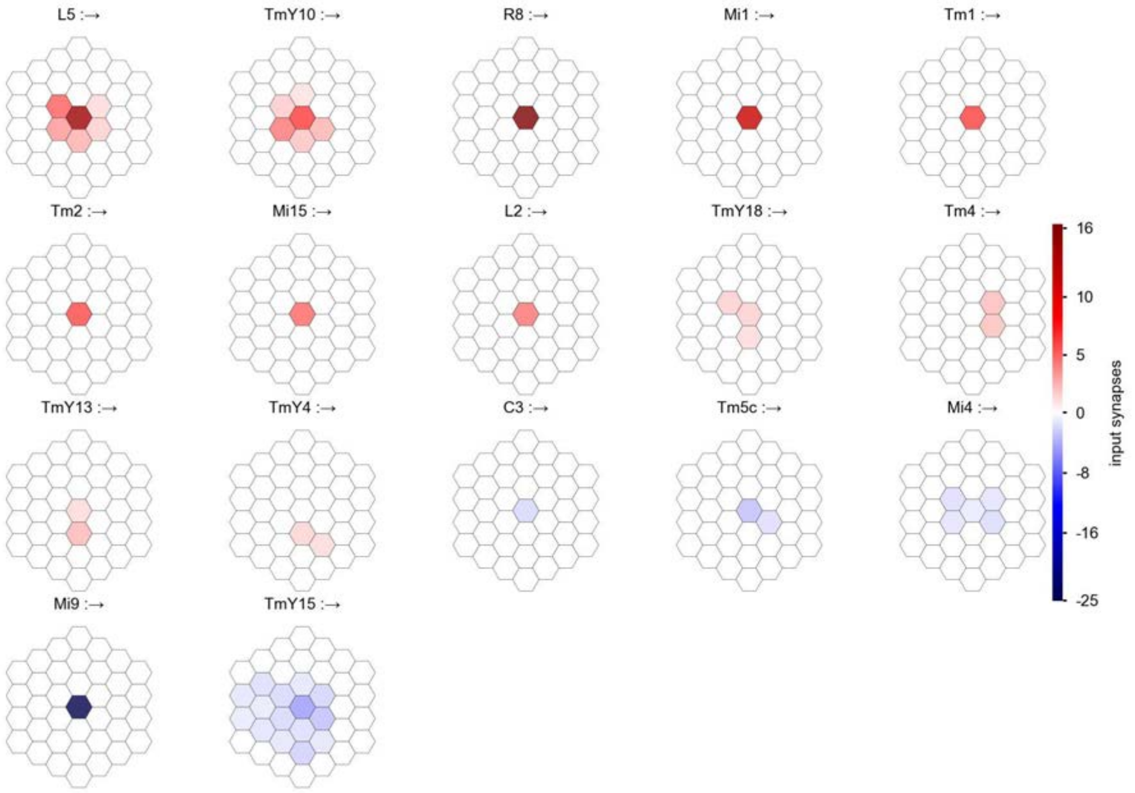

Mi4 - Figure 1: **Anatomical receptive fields.** Each colored hexagon is an input connection, with the connection strength characterized by the average number of synapses that we count from the EM reconstruction. Red indicates excitatory synapses, blue indicates inhibitory synapses from inferred signs. Filters in the order of their total number of synapses.

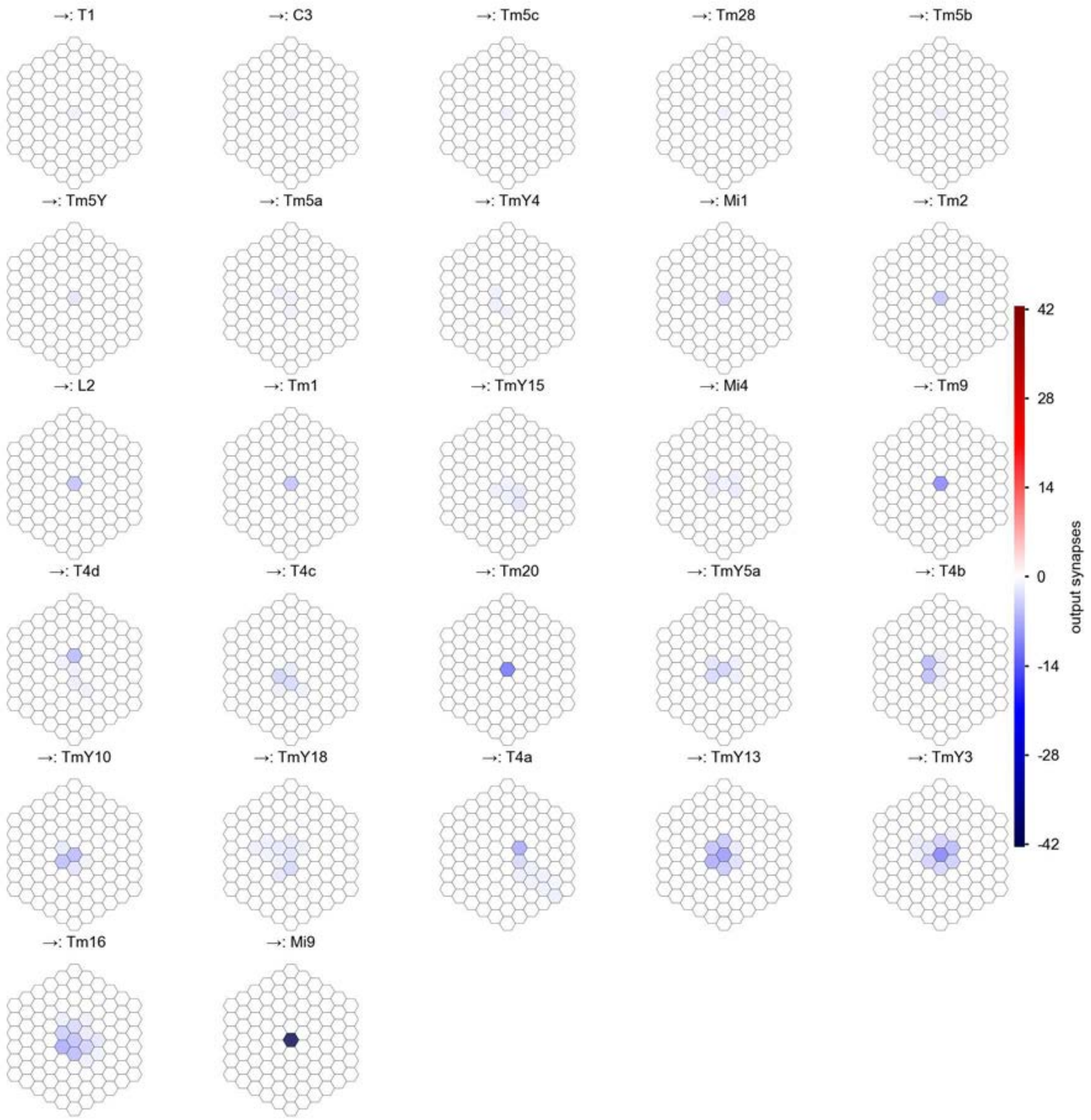

Mi4 - Figure 2: **Anatomical projective fields.** Each colored hexagon is an output connection, with the connection strength characterized by the average number of synapses that we count from the EM reconstruction. Red indicates excitatory synapses, blue indicates inhibitory synapses from inferred signs. Filters in the order of their total number of synapses.

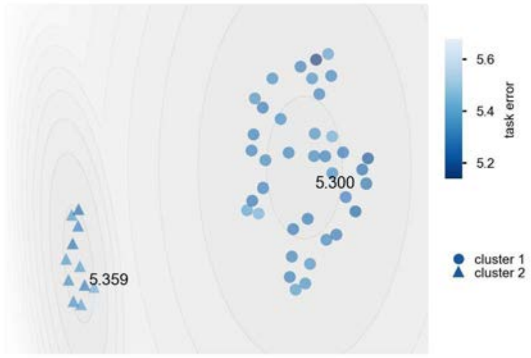

Mi4 - Figure 3: **Clustering of the responses to naturalistic stimuli.** Clustering of the 50 models based on the cell type responses to naturalistic scenes from the Sintel dataset. Scatterpoints represent individual models colored by their task error.

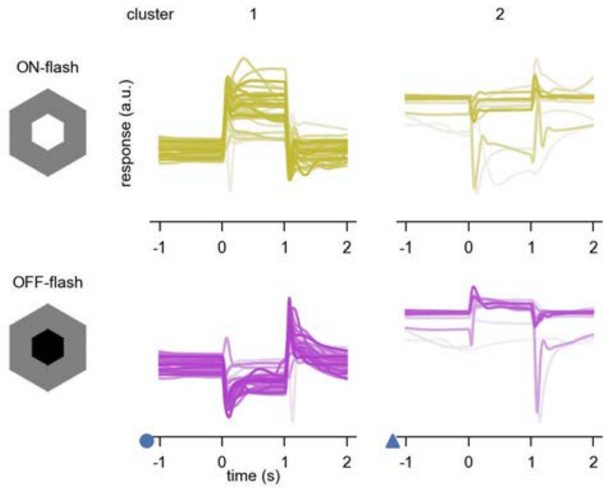

Mi4 - Figure 4: **Responses to flashes.** The top row shows responses to ON-flashes (yellow), the bottom row shows responses to OFF-flashes (magenta). The responses from the 50 different models that are separated into the different clusters (columns) overlay, with better task-performing models on top. Responses from better task-performing models are more saturated. The circular flashes (1s) cover 6 ommatidia in radius and are presented at time zero. Before and after, a grey-stimulus leads to a stationary state of the network.

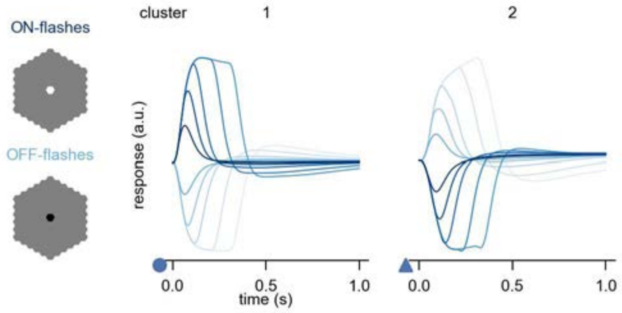

Mi4 - Figure 5: **Cluster-average responses to single-ommatidium flashes.** Responses to single-ommatidium ON-flashes (dark blue shades) and single-ommatidium OFF-flashes (light blue shades) of 20ms, 50ms, 100ms, 200ms, 300ms duration. The flashes occur at second zero.

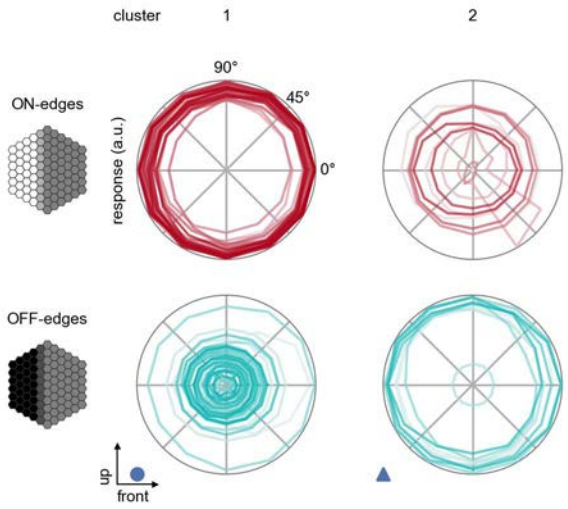

Mi4 - Figure 6: **Peak responses to moving edges.** The top row shows peak responses to moving ON-edges (red), the bottom row shows peak responses to moving OFF-edges (turqoise). The peak responses are averaged over edge-speeds. Edge-stimuli move in different directions from 0 to 360 degrees. The responses from the different models in the different clusters (columns) overlay. Responses from better task-performing models are more saturated.

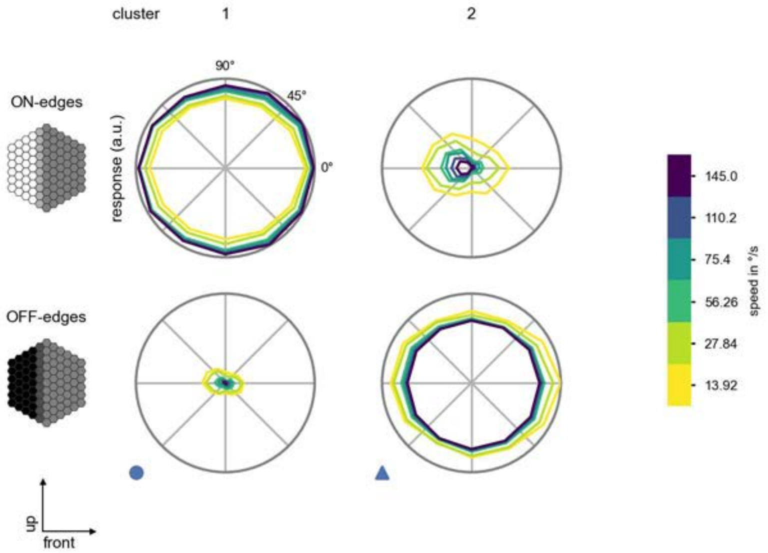

Mi4 - Figure 7: **Peak responses to moving edges from task-optimal models.** The top row shows peak responses to moving ON-edges, the bottom row shows peak responses to moving OFF-edges of varying speeds from 13.92°/s to 145°/s (yellow to dark blue). The edge-stimuli move in different directions from 0 to 360 degrees and at different speeds. Responses from the task-optimal model in the respective cluster.

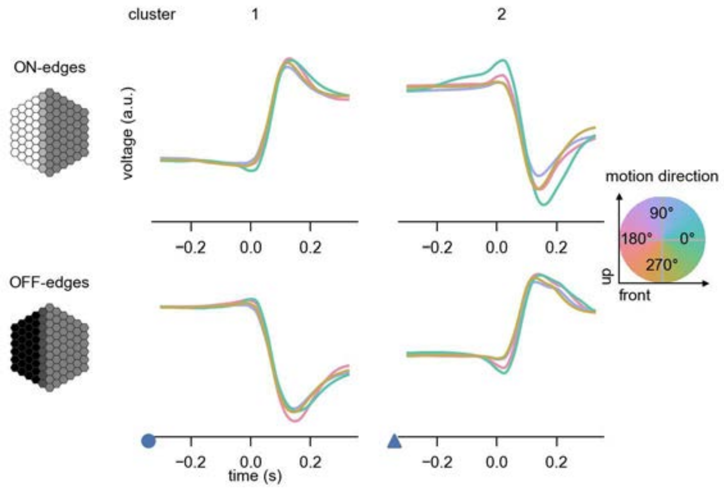

Mi4 - Figure 8: **Responses to moving edges from task-optimal models.** Responses to moving ON-edges (top row) and to moving OFF-edges (bottom row). Edges move in different directions from 0 to 360 degrees and at different speeds. Responses are from the task-optimal model in the respective cluster. Edges moving at 75.4°/s in all cardinal directions (green 0°, blue 90°, red 180°, yellow 270°) from -22.5 to 22.5° visual angle.

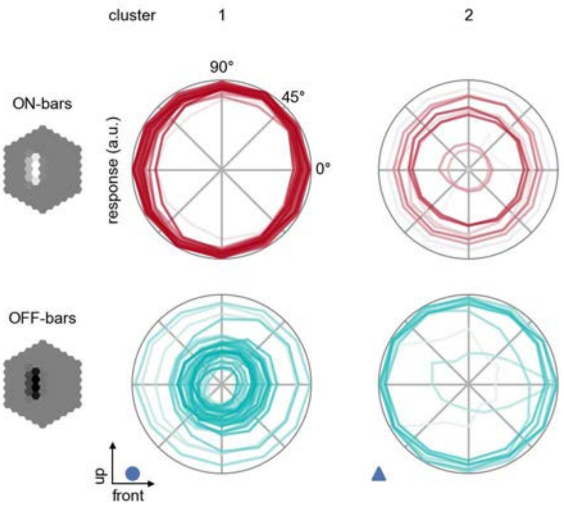

Mi4 - Figure 9: **Peak responses to moving bars.** The top row shows peak responses to moving ON-bars (red), the bottom row shows peak responses to moving OFF-bars (turqoise). The peak responses are averaged over bar-speeds. Bar-stimuli move in different directions from 0 to 360 degrees. The responses from the different models in the different clusters (columns) overlay. Responses from better task-performing models are more saturated.

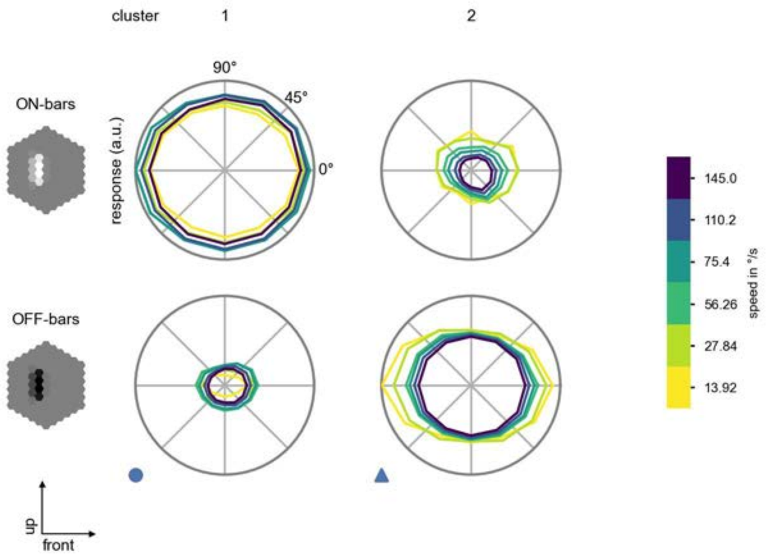

Mi4 - Figure 10: **Peak responses to moving bars from task-optimal models.** The top row shows peak responses to moving ON-bars, the bottom row shows peak responses to moving OFF-bars of varying speeds from 13.92°/s to 145°/s (yellow to dark blue). The bar-stimuli move in different directions from 0 to 360 degrees and at different speeds. Responses from the task-optimal model in the respective cluster.

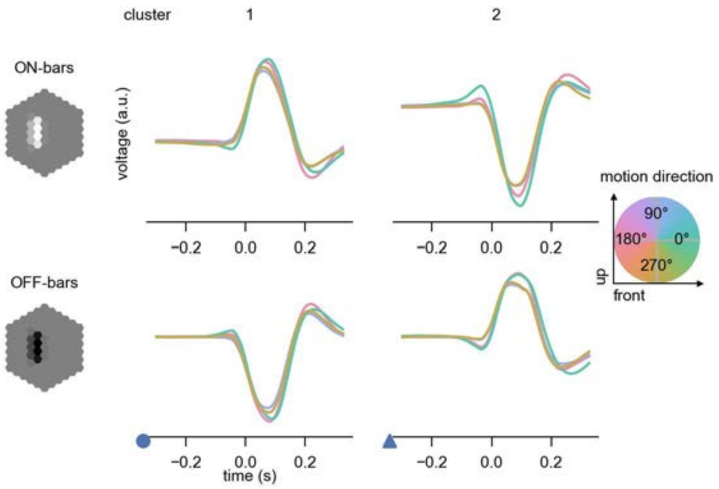

Mi4 - Figure 11: **Responses to moving bars from task-optimal models.** Responses to moving ON-bars (top row) and to moving OFF-bars (bottom row). Bars move in different directions from 0 to 360 degrees and at different speeds. Responses are from the task-optimal model in the respective cluster. Bars moving at 75.4°/s in all cardinal directions (green 0°, blue 90°, red 180°, yellow 270°) from -22.5 to 22.5° visual angle.

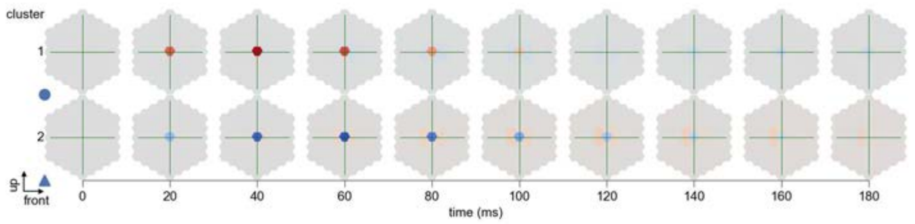

Mi4 - Figure 12: **Spatio-temporal receptive field.** Responses of the central cell to ON-impulses (5 ms) at single-ommatidium flash locations. The flash occurs at second zero. Responses from the task-optimal model of the respective cluster (rows). Red indicates depolarization, blue indicates hyperpolarization.

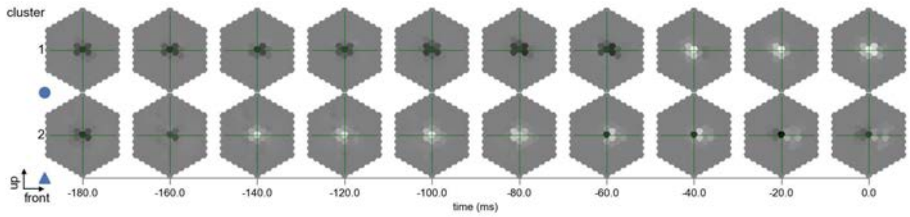

Mi4 - Figure 13: **Maximally excitatory stimuli.** Each row presents the regularized naturalistic-stimulus from the Sintel dataset that maximizes the cell type's central column response at second zero in the task-optimal model of the respective cluster (rows).

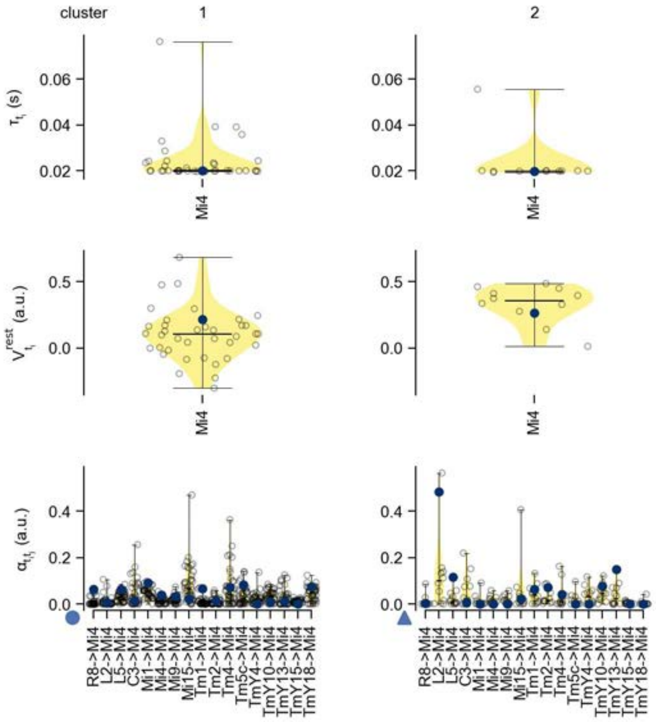

Mi4 - Figure 14: **Task-constrained parameters.** Each column shows the parameters inferred within the respective cluster. First row: learned time constants of the cell type. Second row: resting potentials of the cell type. Third row: scaling factors for the convolutional filters. The blue scatter represents the parameters from the task-optimal model within the cluster.

25 Mi9

← Cell types

Figures

1 Anatomical receptive fields. 174
2 Anatomical projective fields. 175
3 Clustering of the responses to naturalistic stimuli. 175
4 Responses to flashes. 176
5 Cluster-average responses to single-ommatidium flashes. 176
6 Peak responses to moving edges. 177
7 Peak responses to moving edges from task-optimal models. 177
8 Responses to moving edges from task-optimal models. 178
9 Peak responses to moving bars. 178
10 Peak responses to moving bars from task-optimal models. 179
11 Responses to moving bars from task-optimal models. 179
12 Spatio-temporal receptive field. 179
13 Maximally excitatory stimuli. 180
14 Task-constrained parameters. 180

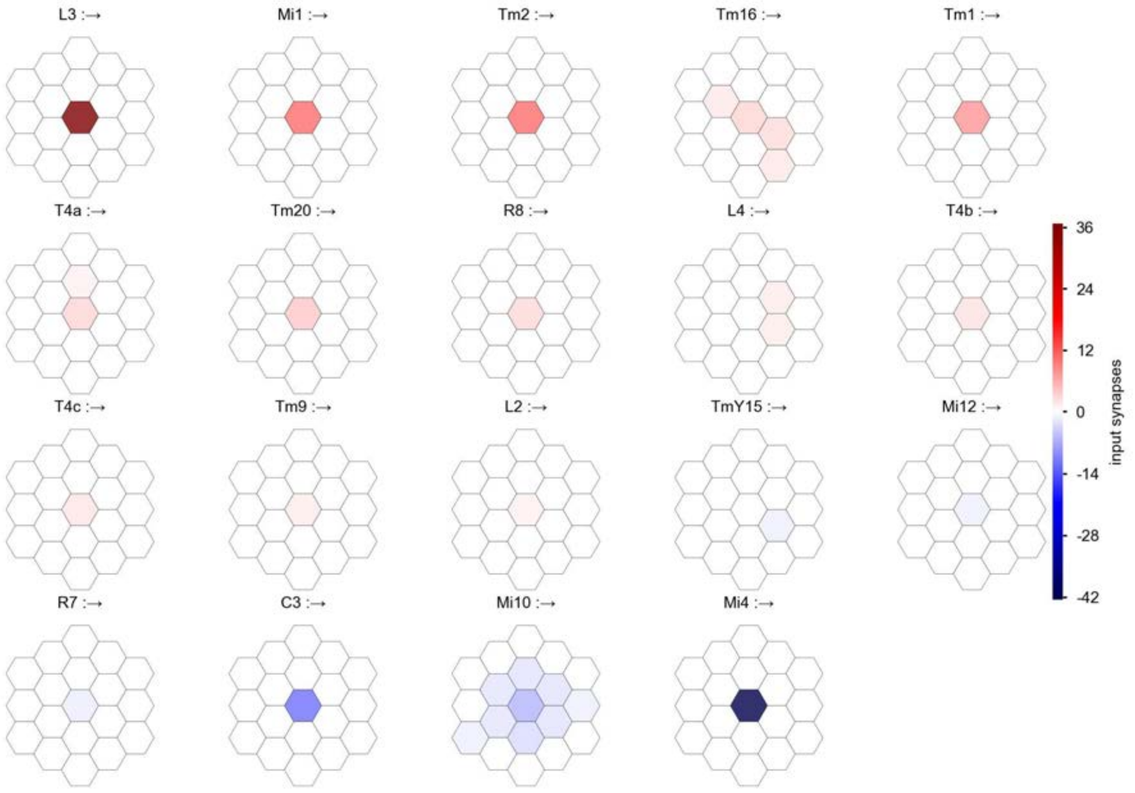

Mi9 - Figure 1: **Anatomical receptive fields.** Each colored hexagon is an input connection, with the connection strength characterized by the average number of synapses that we count from the EM reconstruction. Red indicates excitatory synapses, blue indicates inhibitory synapses from inferred signs. Filters in the order of their total number of synapses.

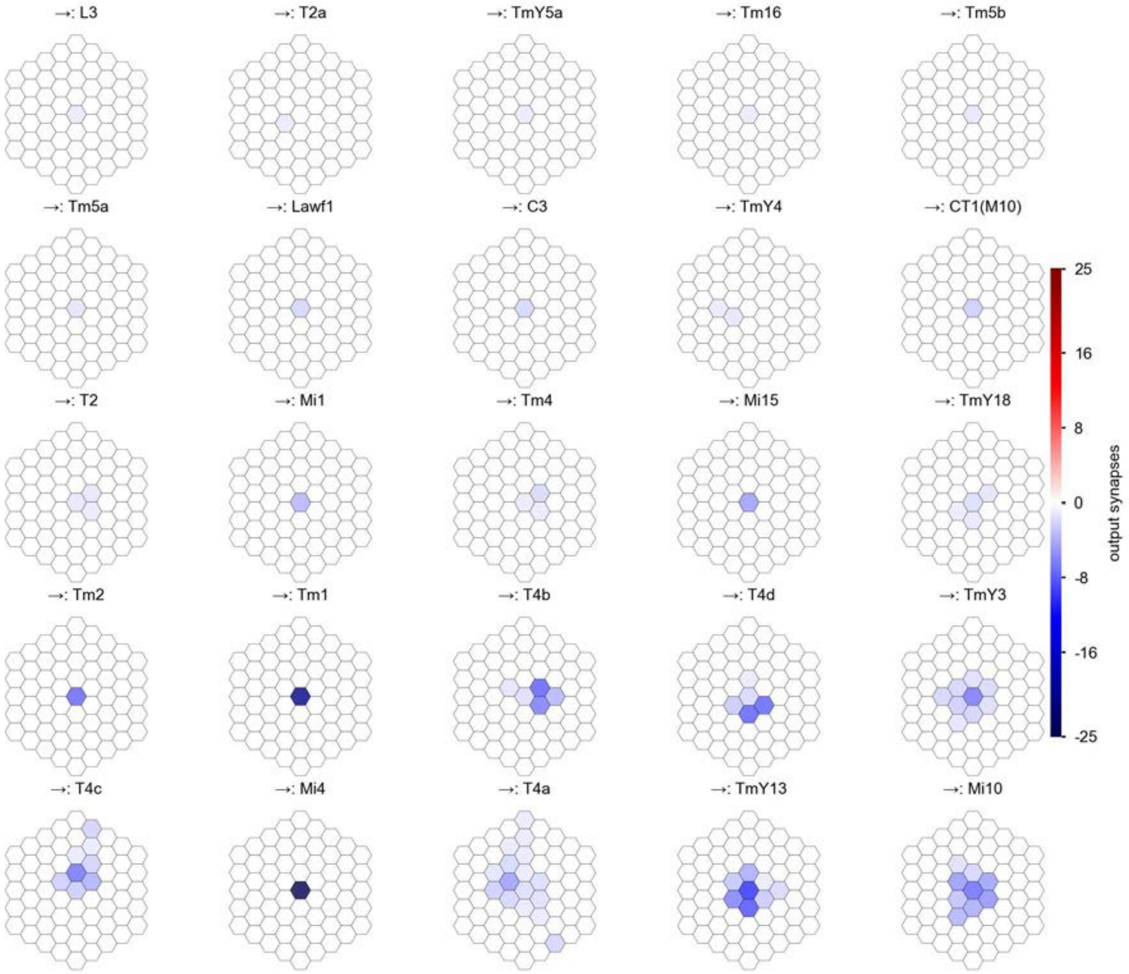

Mi9 - Figure 2: **Anatomical projective fields.** Each colored hexagon is an output connection, with the connection strength characterized by the average number of synapses that we count from the EM reconstruction. Red indicates excitatory synapses, blue indicates inhibitory synapses from inferred signs. Filters in the order of their total number of synapses.

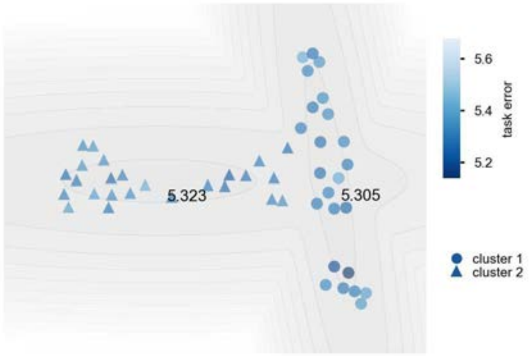

Mi9 - Figure 3: **Clustering of the responses to naturalistic stimuli.** Clustering of the 50 models based on the cell type responses to naturalistic scenes from the Sintel dataset. Scatterpoints represent individual models colored by their task error.

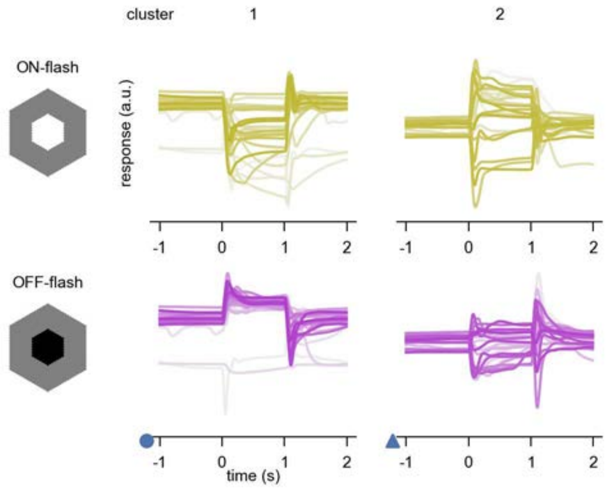

Mi9 - Figure 4: **Responses to flashes.** The top row shows responses to ON-flashes (yellow), the bottom row shows responses to OFF-flashes (magenta). The responses from the 50 different models that are separated into the different clusters (columns) overlay, with better task-performing models on top. Responses from better task-performing models are more saturated. The circular flashes (1s) cover 6 ommatidia in radius and are presented at time zero. Before and after, a grey-stimulus leads to a stationary state of the network.

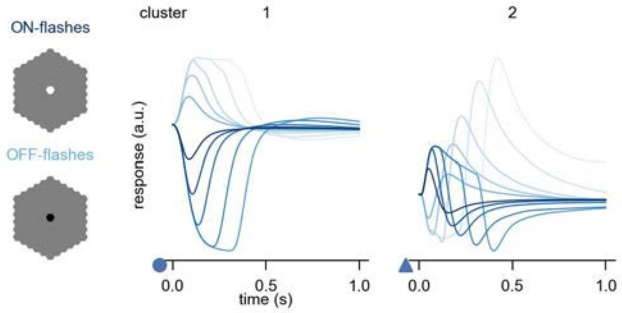

Mi9 - Figure 5: **Cluster-average responses to single-ommatidium flashes.** Responses to single-ommatidium ON-flashes (dark blue shades) and single-ommatidium OFF-flashes (light blue shades) of 20ms, 50ms, 100ms, 200ms, 300ms duration. The flashes occur at second zero.

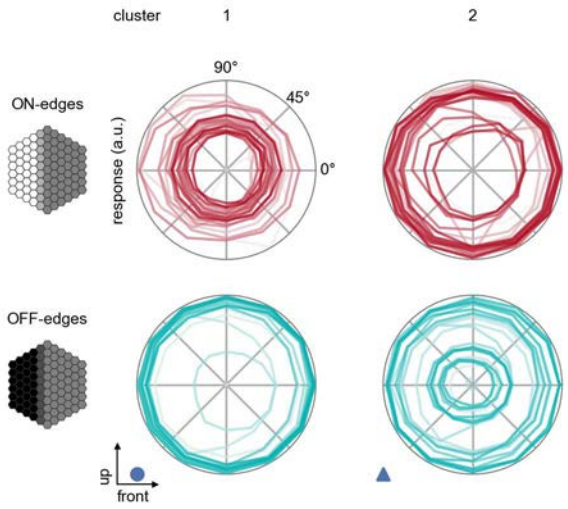

Mi9 - Figure 6: **Peak responses to moving edges.** The top row shows peak responses to moving ON-edges (red), the bottom row shows peak responses to moving OFF-edges (turqoise). The peak responses are averaged over edge-speeds. Edge-stimuli move in different directions from 0 to 360 degrees. The responses from the different models in the different clusters (columns) overlay. Responses from better task-performing models are more saturated.

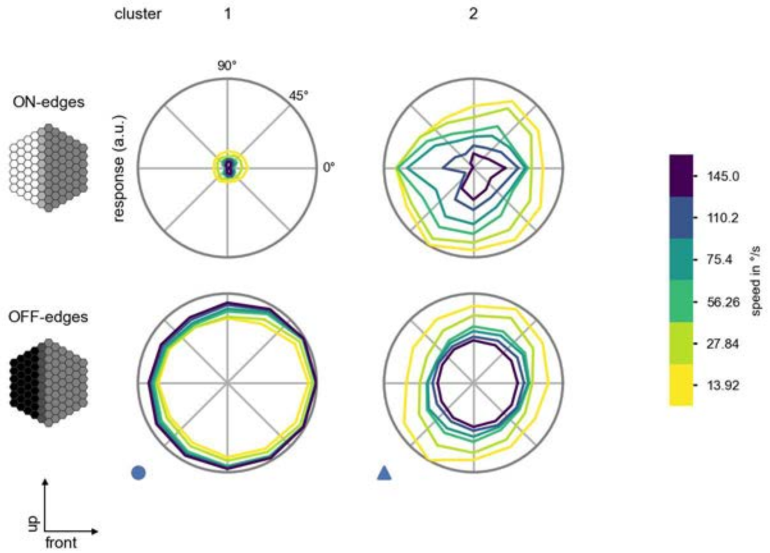

Mi9 - Figure 7: **Peak responses to moving edges from task-optimal models.** The top row shows peak responses to moving ON-edges, the bottom row shows peak responses to moving OFF-edges of varying speeds from 13.92°/s to 145°/s (yellow to dark blue). The edge-stimuli move in different directions from 0 to 360 degrees and at different speeds. Responses from the task-optimal model in the respective cluster.

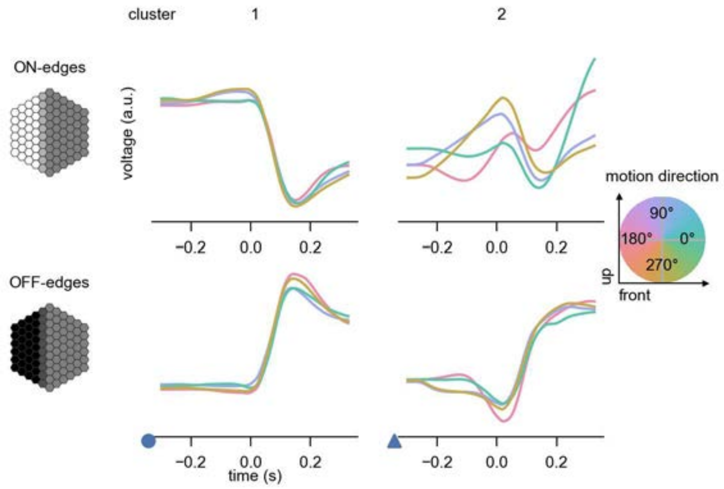

Mi9 - Figure 8: **Responses to moving edges from task-optimal models.** Responses to moving ON-edges (top row) and to moving OFF-edges (bottom row). Edges move in different directions from 0 to 360 degrees and at different speeds. Responses are from the task-optimal model in the respective cluster. Edges moving at 75.4°/s in all cardinal directions (green 0°, blue 90°, red 180°, yellow 270°) from -22.5 to 22.5° visual angle.

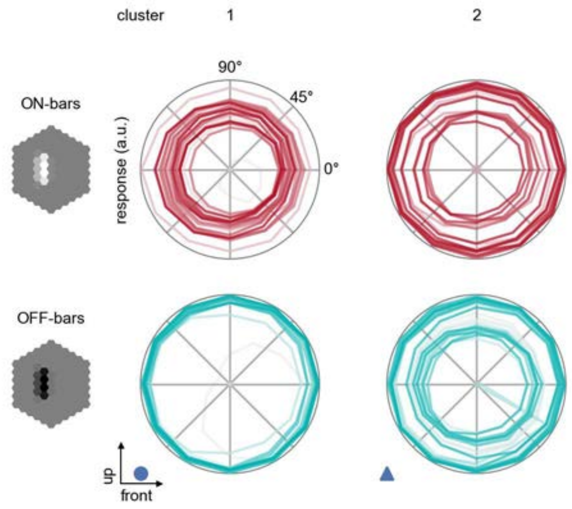

Mi9 - Figure 9: **Peak responses to moving bars.** The top row shows peak responses to moving ON-bars (red), the bottom row shows peak responses to moving OFF-bars (turqoise). The peak responses are averaged over bar-speeds. Bar-stimuli move in different directions from 0 to 360 degrees. The responses from the different models in the different clusters (columns) overlay. Responses from better task-performing models are more saturated.

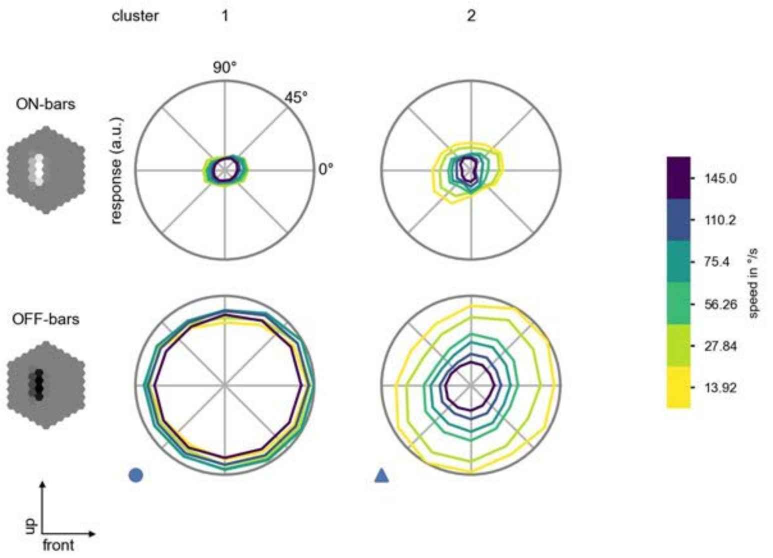

Mi9 - Figure 10: **Peak responses to moving bars from task-optimal models.** The top row shows peak responses to moving ON-bars, the bottom row shows peak responses to moving OFF-bars of varying speeds from 13.92°/s to 145°/s (yellow to dark blue). The bar-stimuli move in different directions from 0 to 360 degrees and at different speeds. Responses from the task-optimal model in the respective cluster.

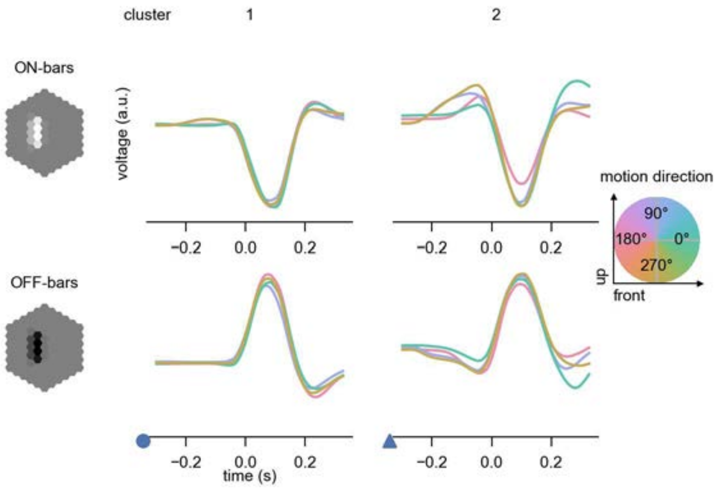

Mi9 - Figure 11: **Responses to moving bars from task-optimal models.** Responses to moving ON-bars (top row) and to moving OFF-bars (bottom row). Bars move in different directions from 0 to 360 degrees and at different speeds. Responses are from the task-optimal model in the respective cluster. Bars moving at 75.4°/s in all cardinal directions (green 0°, blue 90°, red 180°, yellow 270°) from -22.5 to 22.5° visual angle.

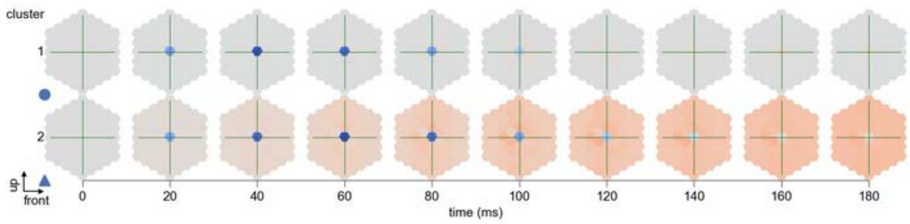

Mi9 - Figure 12: **Spatio-temporal receptive field.** Responses of the central cell to ON-impulses (5 ms) at single-ommatidium flash locations. The flash occurs at second zero. Responses from the task-optimal model of the respective cluster (rows). Red indicates depolarization, blue indicates hyperpolarization.

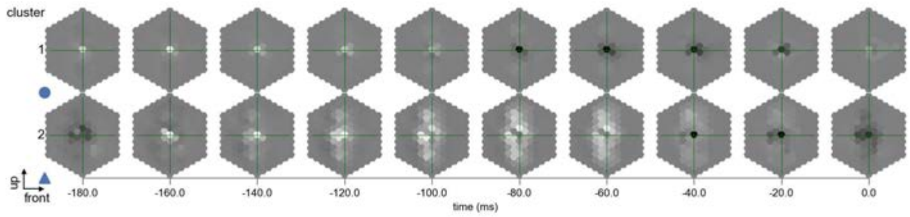

Mi9 - Figure 13: **Maximally excitatory stimuli.** Each row presents the regularized naturalistic-stimulus from the Sintel dataset that maximizes the cell type's central column response at second zero in the task-optimal model of the respective cluster (rows).

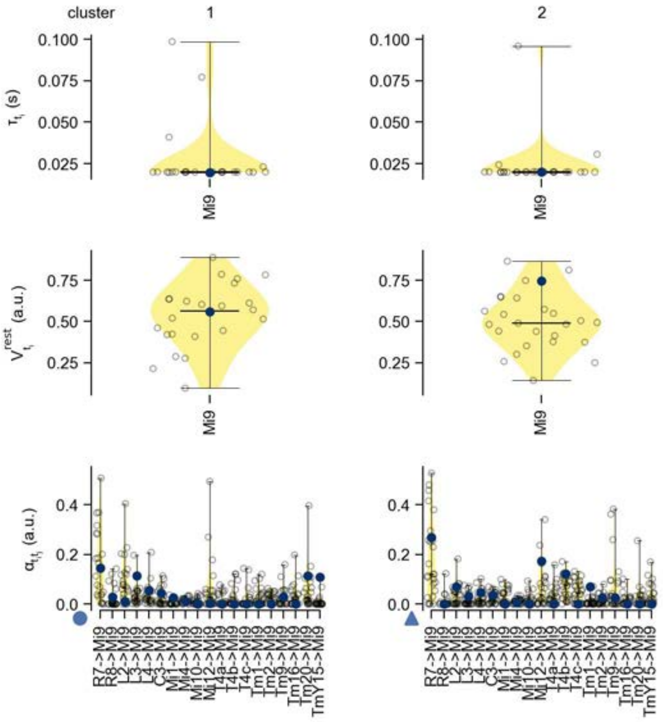

Mi9 - Figure 14: **Task-constrained parameters.** Each column shows the parameters inferred within the respective cluster. First row: learned time constants of the cell type. Second row: resting potentials of the cell type. Third row: scaling factors for the convolutional filters. The blue scatter represents the parameters from the task-optimal model within the cluster.

26 Mi10

← Cell types

Figures

1 Anatomical receptive fields. 181
2 Anatomical projective fields. 182
3 Clustering of the responses to naturalistic stimuli. 182
4 Responses to flashes. 183
5 Cluster-average responses to single-ommatidium flashes. 183
6 Peak responses to moving edges. 184
7 Peak responses to moving edges from task-optimal models. 184
8 Responses to moving edges from task-optimal models. 185
9 Peak responses to moving bars. 185
10 Peak responses to moving bars from task-optimal models. 186
11 Responses to moving bars from task-optimal models. 186
12 Spatio-temporal receptive field. 187
13 Maximally excitatory stimuli. 187
14 Task-constrained parameters. 188

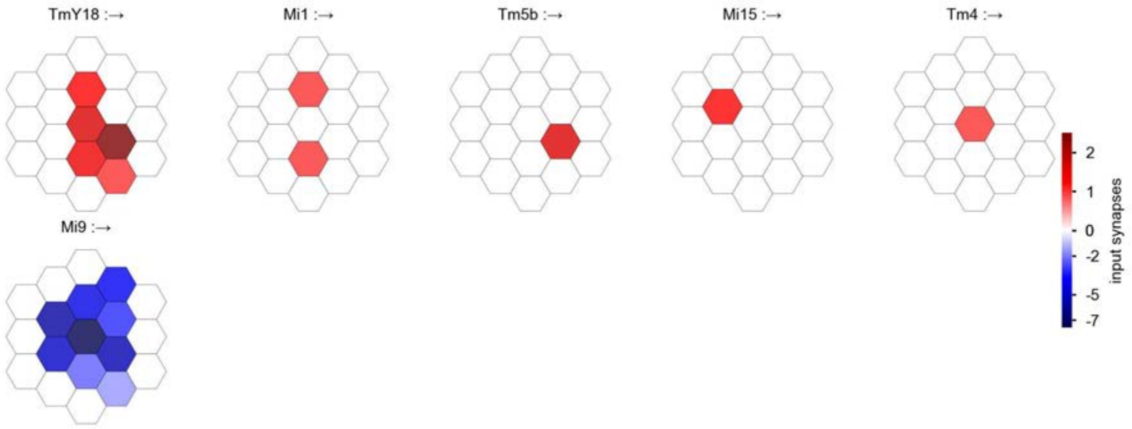

Mi10 - Figure 1: **Anatomical receptive fields.** Each colored hexagon is an input connection, with the connection strength characterized by the average number of synapses that we count from the EM reconstruction. Red indicates excitatory synapses, blue indicates inhibitory synapses from inferred signs. Filters in the order of their total number of synapses.

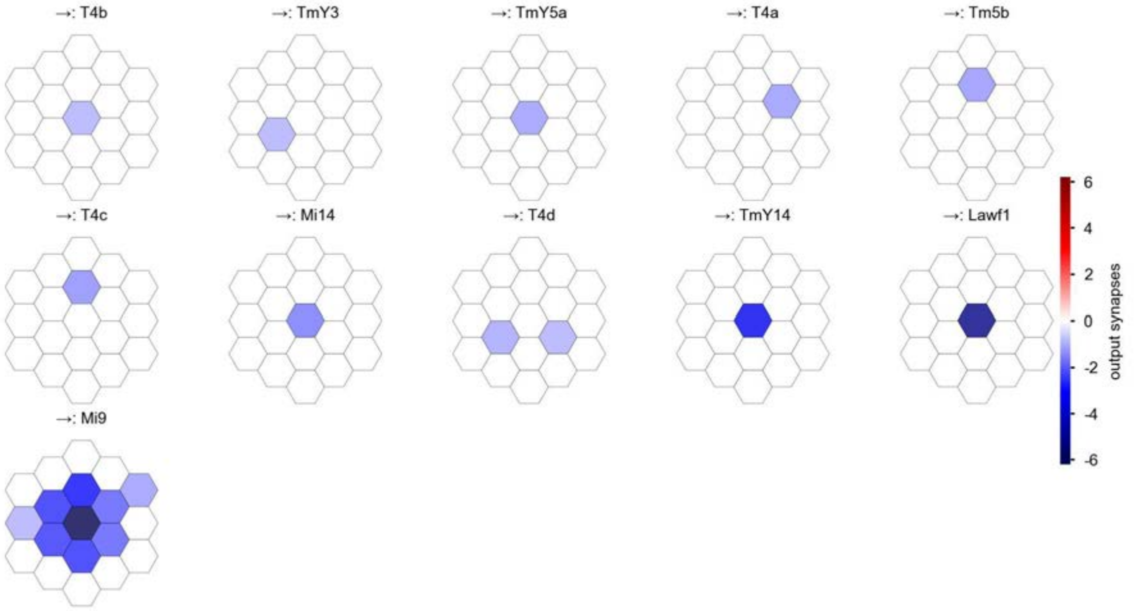

Mi10 - Figure 2: **Anatomical projective fields.** Each colored hexagon is an output connection, with the connection strength characterized by the average number of synapses that we count from the EM reconstruction. Red indicates excitatory synapses, blue indicates inhibitory synapses from inferred signs. Filters in the order of their total number of synapses.

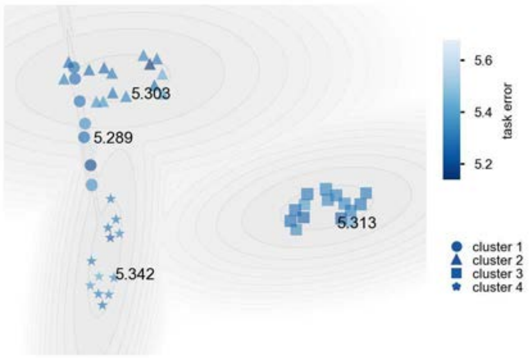

Mi10 - Figure 3: **Clustering of the responses to naturalistic stimuli.** Clustering of the 50 models based on the cell type responses to naturalistic scenes from the Sintel dataset. Scatterpoints represent individual models colored by their task error.

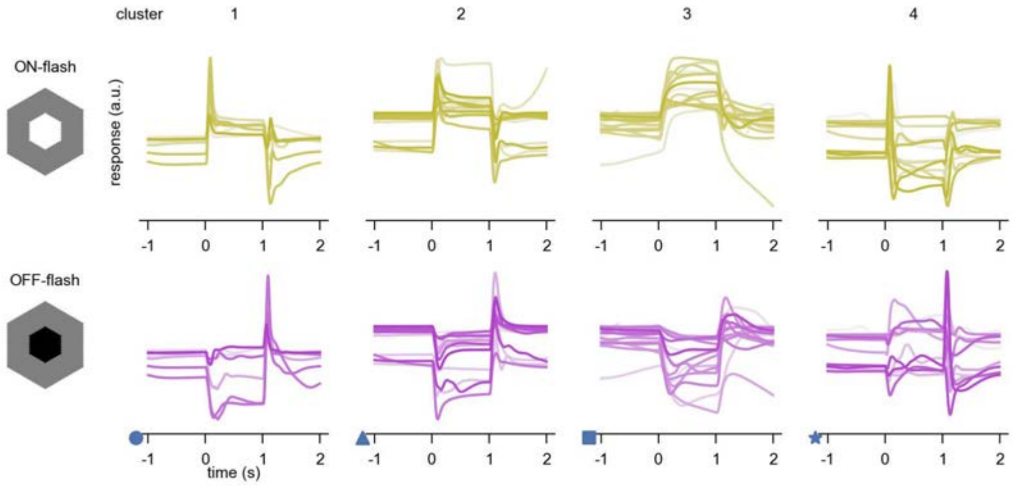

Mi10 - Figure 4: **Responses to flashes.** The top row shows responses to ON-flashes (yellow), the bottom row shows responses to OFF-flashes (magenta). The responses from the 50 different models that are separated into the different clusters (columns) overlay, with better task-performing models on top. Responses from better task-performing models are more saturated. The circular flashes (1s) cover 6 ommatidia in radius and are presented at time zero. Before and after, a grey-stimulus leads to a stationary state of the network.

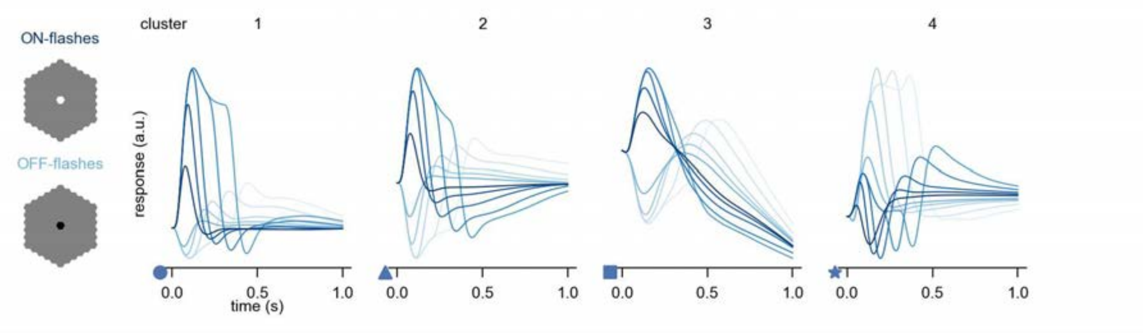

Mi10 - Figure 5: **Cluster-average responses to single-ommatidium flashes.** Responses to single-ommatidium ON-flashes (dark blue shades) and single-ommatidium OFF-flashes (light blue shades) of 20ms, 50ms, 100ms, 200ms, 300ms duration. The flashes occur at second zero.

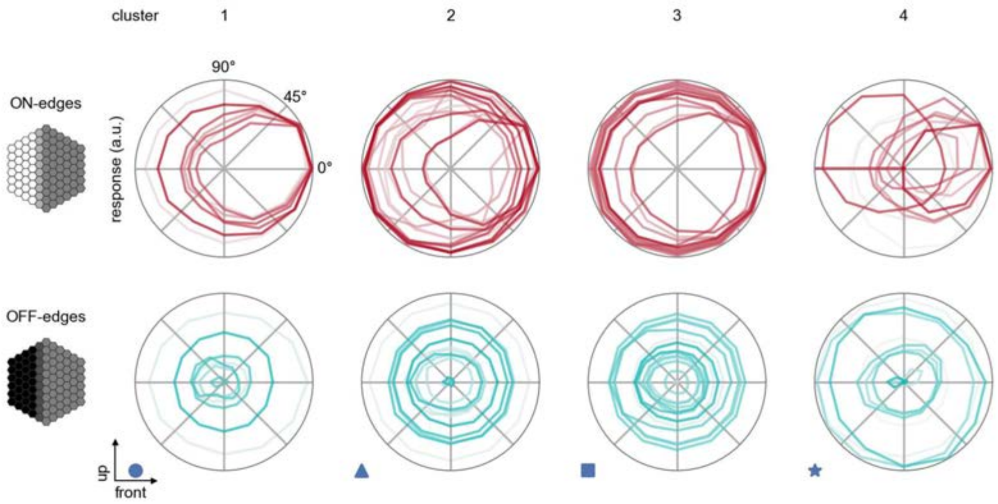

Mi10 - Figure 6: **Peak responses to moving edges.** The top row shows peak responses to moving ON-edges (red), the bottom row shows peak responses to moving OFF-edges (turqoise). The peak responses are averaged over edge-speeds. Edge-stimuli move in different directions from 0 to 360 degrees. The responses from the different models in the different clusters (columns) overlay. Responses from better task-performing models are more saturated.

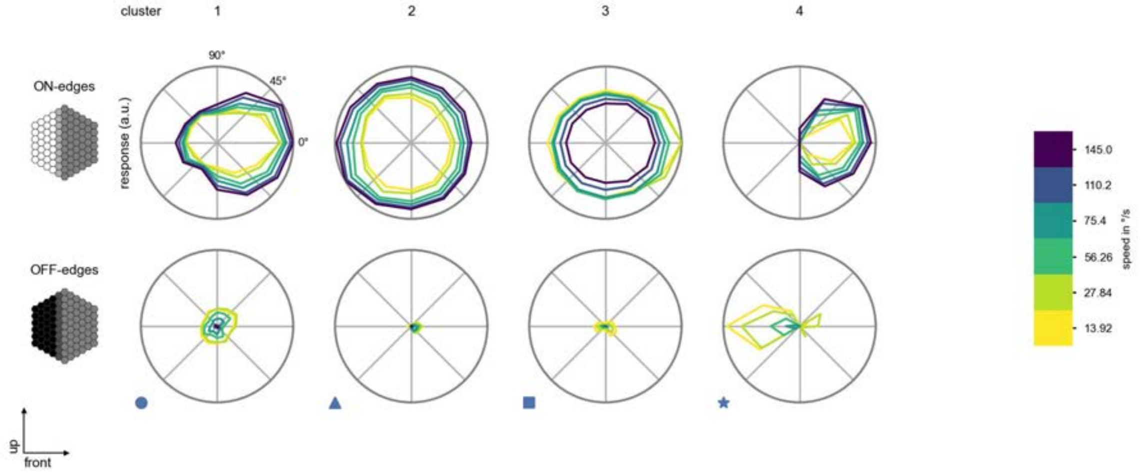

Mi10 - Figure 7: **Peak responses to moving edges from task-optimal models.** The top row shows peak responses to moving ON-edges, the bottom row shows peak responses to moving OFF-edges of varying speeds from 13.92°/s to 145°/s (yellow to dark blue). The edge-stimuli move in different directions from 0 to 360 degrees and at different speeds. Responses from the task-optimal model in the respective cluster.

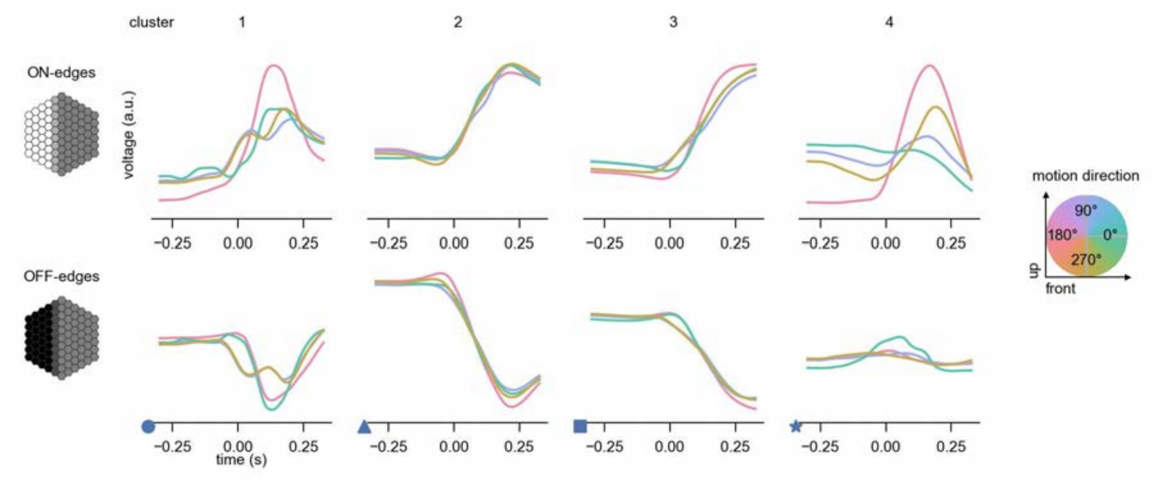

Mi10 - Figure 8: **Responses to moving edges from task-optimal models.** Responses to moving ON-edges (top row) and to moving OFF-edges (bottom row). Edges move in different directions from 0 to 360 degrees and at different speeds. Responses are from the task-optimal model in the respective cluster. Edges moving at 75.4°/s in all cardinal directions (green 0°, blue 90°, red 180°, yellow 270°) from -22.5 to 22.5° visual angle.

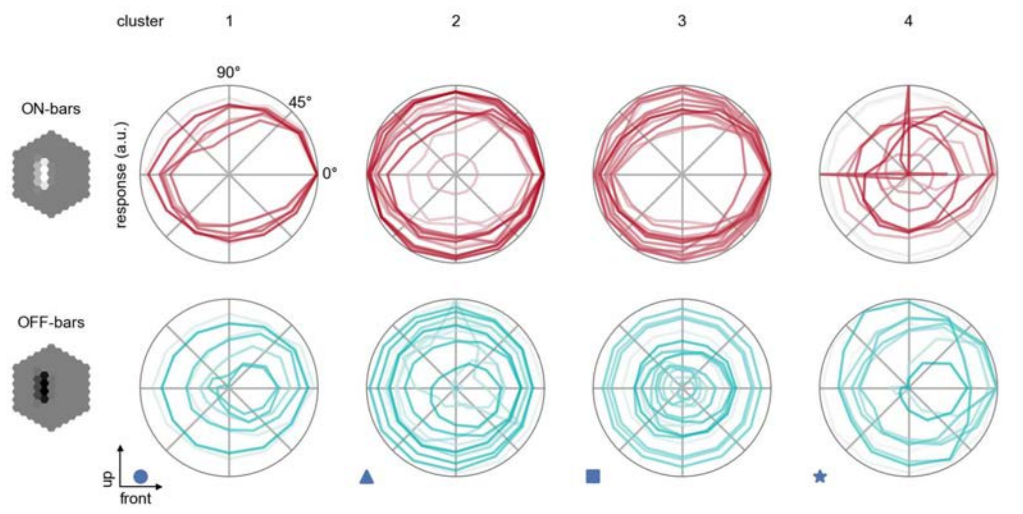

Mi10 - Figure 9: **Peak responses to moving bars.** The top row shows peak responses to moving ON-bars (red), the bottom row shows peak responses to moving OFF-bars (turqoise). The peak responses are averaged over bar-speeds. Bar-stimuli move in different directions from 0 to 360 degrees. The responses from the different models in the different clusters (columns) overlay. Responses from better task-performing models are more saturated.

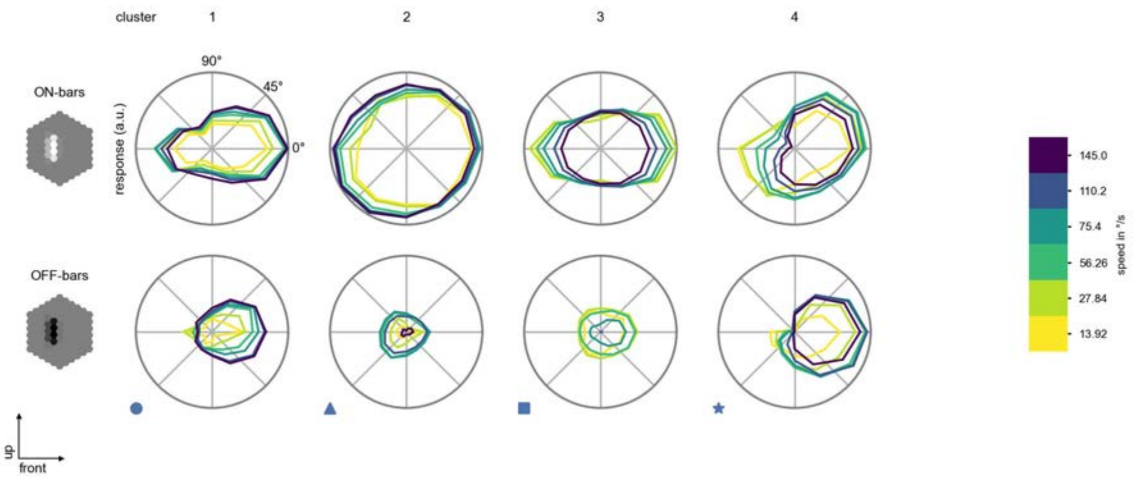

Mi10 - Figure 10: **Peak responses to moving bars from task-optimal models.** The top row shows peak responses to moving ON-bars, the bottom row shows peak responses to moving OFF-bars of varying speeds from 13.92°/s to 145°/s (yellow to dark blue). The bar-stimuli move in different directions from 0 to 360 degrees and at different speeds. Responses from the task-optimal model in the respective cluster.

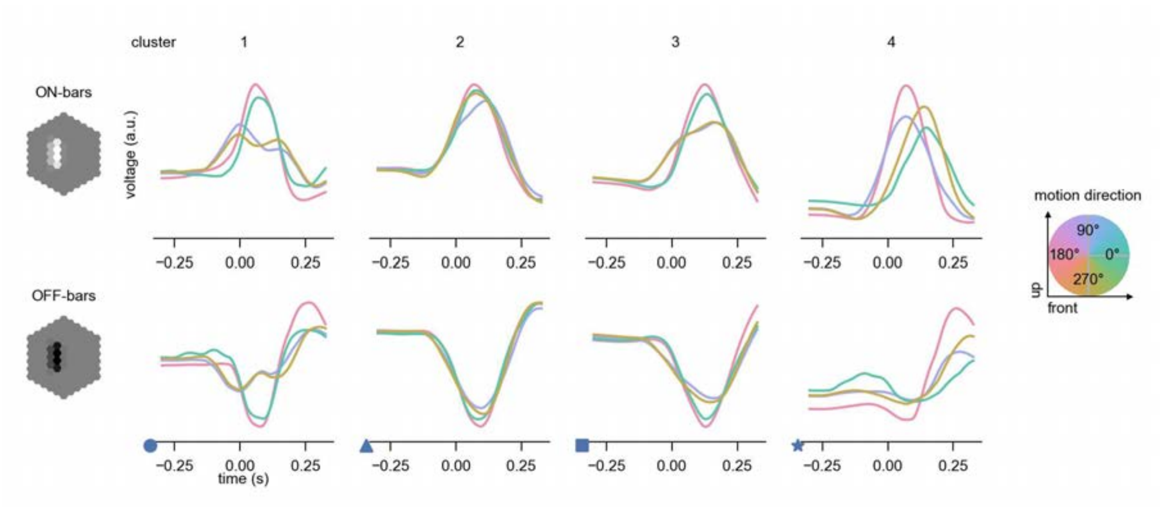

Mi10 - Figure 11: **Responses to moving bars from task-optimal models.** Responses to moving ON-bars (top row) and to moving OFF-bars (bottom row). Bars move in different directions from 0 to 360 degrees and at different speeds. Responses are from the task-optimal model in the respective cluster. Bars moving at 75.4°/s in all cardinal directions (green 0°, blue 90°, red 180°, yellow 270°) from -22.5 to 22.5° visual angle.

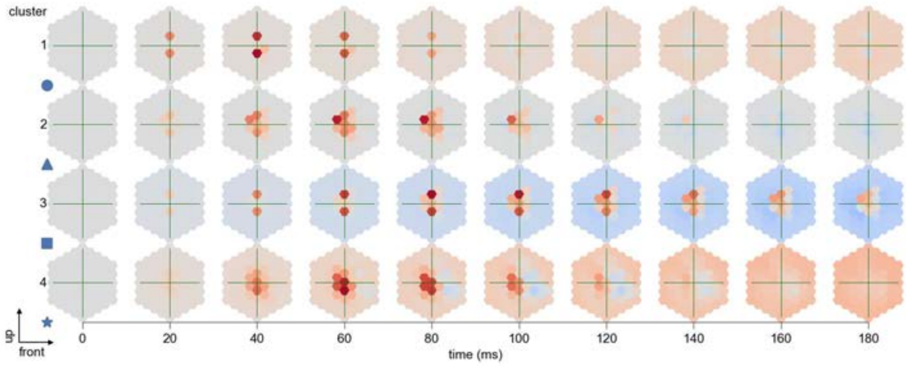

Mi10 - Figure 12: **Spatio-temporal receptive field.** Responses of the central cell to ON-impulses (5 ms) at single-ommatidium flash locations. The flash occurs at second zero. Responses from the task-optimal model of the respective cluster (rows). Red indicates depolarization, blue indicates hyperpolarization.

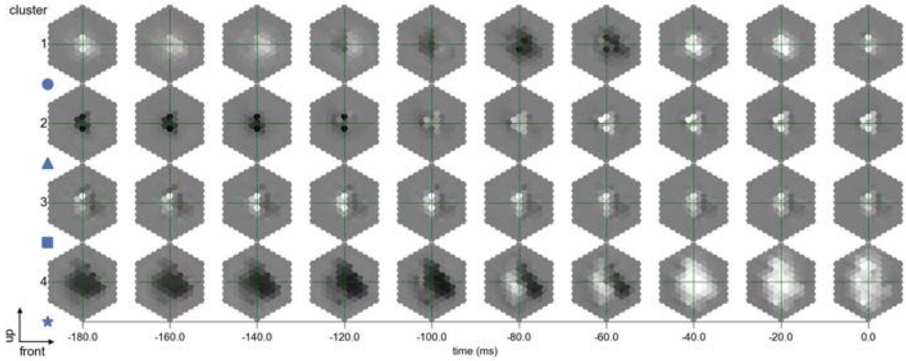

Mi10 - Figure 13: **Maximally excitatory stimuli.** Each row presents the regularized naturalistic-stimulus from the Sintel dataset that maximizes the cell type's central column response at second zero in the task-optimal model of the respective cluster (rows).

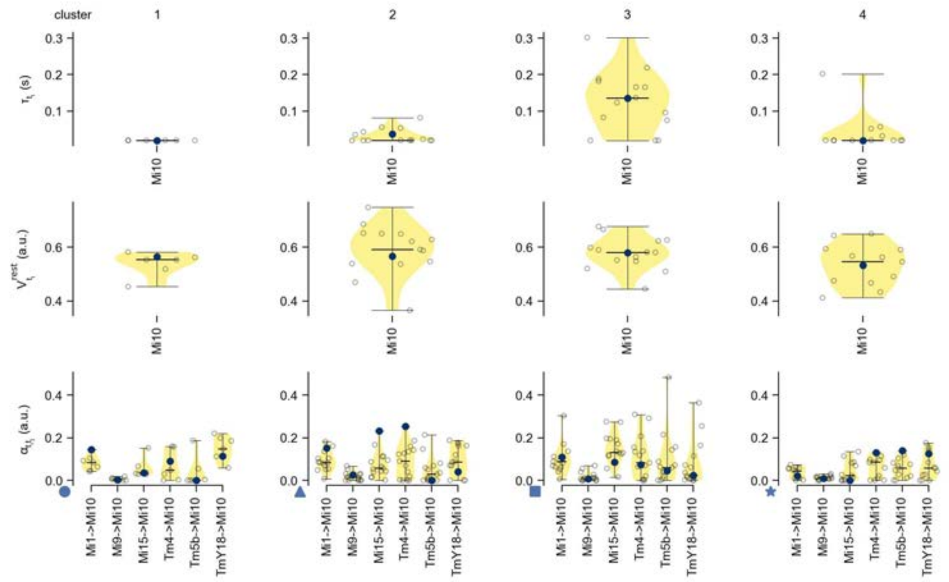

Mi10 - Figure 14: **Task-constrained parameters.** Each column shows the parameters inferred within the respective cluster. First row: learned time constants of the cell type. Second row: resting potentials of the cell type. Third row: scaling factors for the convolutional filters. The blue scatter represents the parameters from the task-optimal model within the cluster.

27 Mi11

← Cell types

Figures

1 Anatomical receptive fields. 189
2 Clustering of the responses to naturalistic stimuli. 189
3 Responses to flashes. 190
4 Cluster-average responses to single-ommatidium flashes. 190
5 Peak responses to moving edges. 191
6 Peak responses to moving edges from task-optimal models. 191
7 Responses to moving edges from task-optimal models. 192
8 Peak responses to moving bars. 192
9 Peak responses to moving bars from task-optimal models. 193
10 Responses to moving bars from task-optimal models. 193
11 Spatio-temporal receptive field. 193
12 Maximally excitatory stimuli. 194
13 Task-constrained parameters. 194

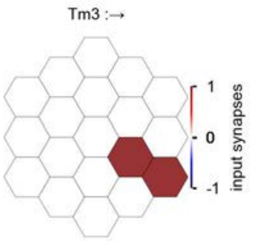

Mi11 - Figure 1: **Anatomical receptive fields.** Each colored hexagon is an input connection, with the connection strength characterized by the average number of synapses that we count from the EM reconstruction. Red indicates excitatory synapses, blue indicates inhibitory synapses from inferred signs. Filters in the order of their total number of synapses.

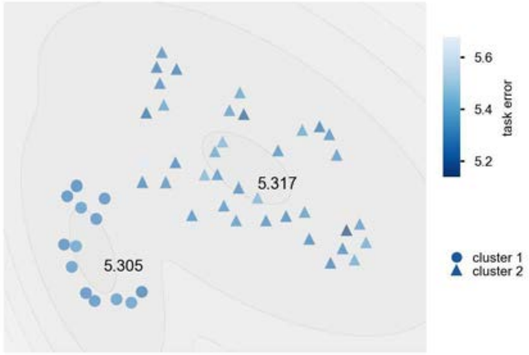

Mi11 - Figure 2: **Clustering of the responses to naturalistic stimuli.** Clustering of the 50 models based on the cell type responses to naturalistic scenes from the Sintel dataset. Scatterpoints represent individual models colored by their task error.

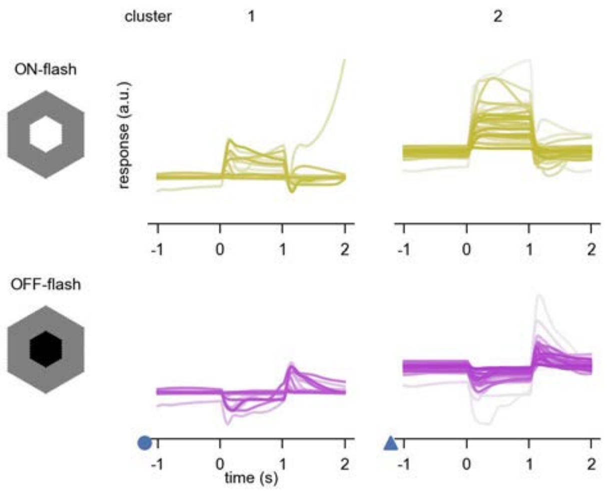

Mi11 - Figure 3: **Responses to flashes.** The top row shows responses to ON-flashes (yellow), the bottom row shows responses to OFF-flashes (magenta). The responses from the 50 different models that are separated into the different clusters (columns) overlay, with better task-performing models on top. Responses from better task-performing models are more saturated. The circular flashes (1s) cover 6 ommatidia in radius and are presented at time zero. Before and after, a grey-stimulus leads to a stationary state of the network.

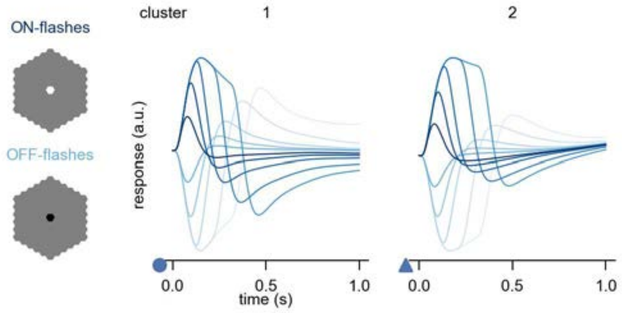

Mi11 - Figure 4: **Cluster-average responses to single-ommatidium flashes.** Responses to single-ommatidium ON-flashes (dark blue shades) and single-ommatidium OFF-flashes (light blue shades) of 20ms, 50ms, 100ms, 200ms, 300ms duration. The flashes occur at second zero.

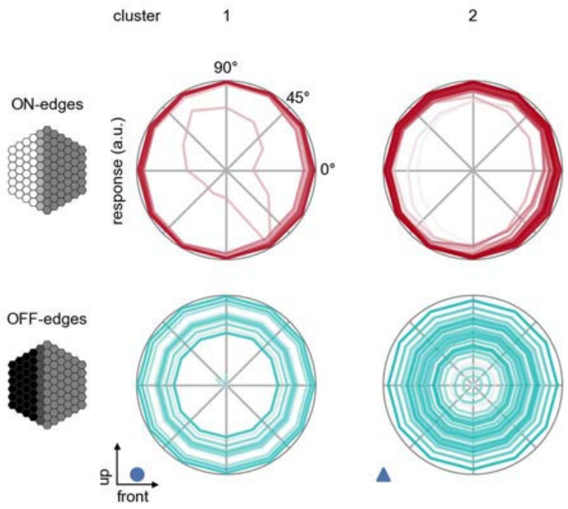

Mi11 - Figure 5: **Peak responses to moving edges.** The top row shows peak responses to moving ON-edges (red), the bottom row shows peak responses to moving OFF-edges (turqoise). The peak responses are averaged over edge-speeds. Edge-stimuli move in different directions from 0 to 360 degrees. The responses from the different models in the different clusters (columns) overlay. Responses from better task-performing models are more saturated.

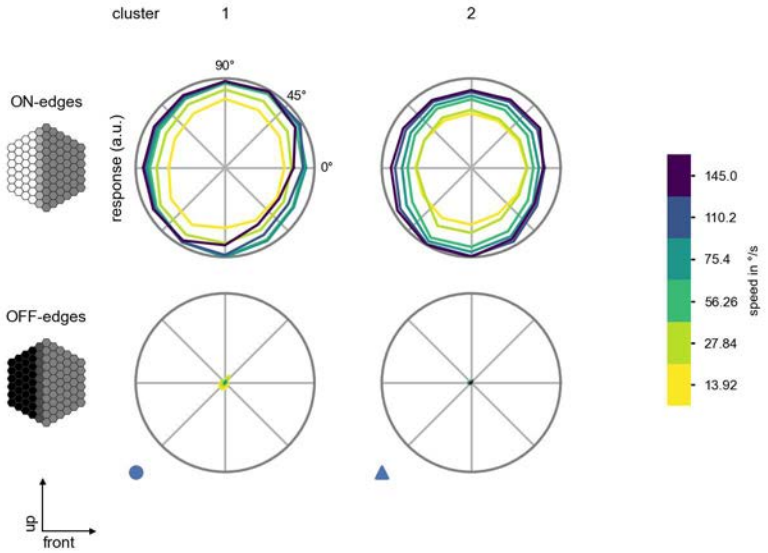

Mi11 - Figure 6: **Peak responses to moving edges from task-optimal models.** The top row shows peak responses to moving ON-edges, the bottom row shows peak responses to moving OFF-edges of varying speeds from 13.92°/s to 145°/s (yellow to dark blue). The edge-stimuli move in different directions from 0 to 360 degrees and at different speeds. Responses from the task-optimal model in the respective cluster.

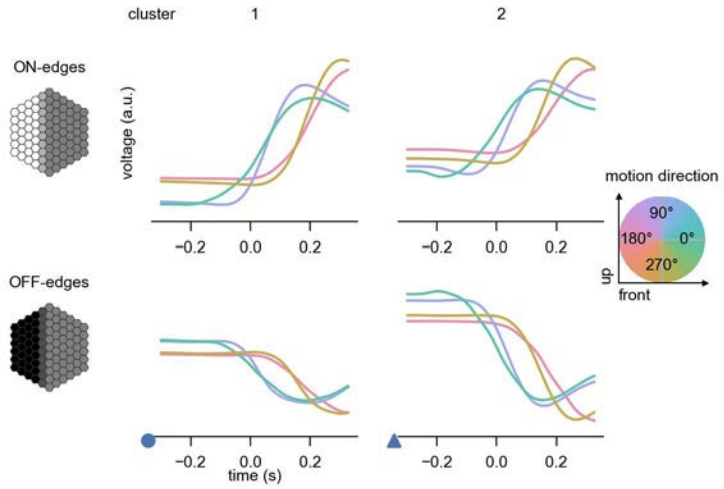

Mi11 - Figure 7: **Responses to moving edges from task-optimal models.** Responses to moving ON-edges (top row) and to moving OFF-edges (bottom row). Edges move in different directions from 0 to 360 degrees and at different speeds. Responses are from the task-optimal model in the respective cluster. Edges moving at 75.4°/s in all cardinal directions (green 0°, blue 90°, red 180°, yellow 270°) from -22.5 to 22.5° visual angle.

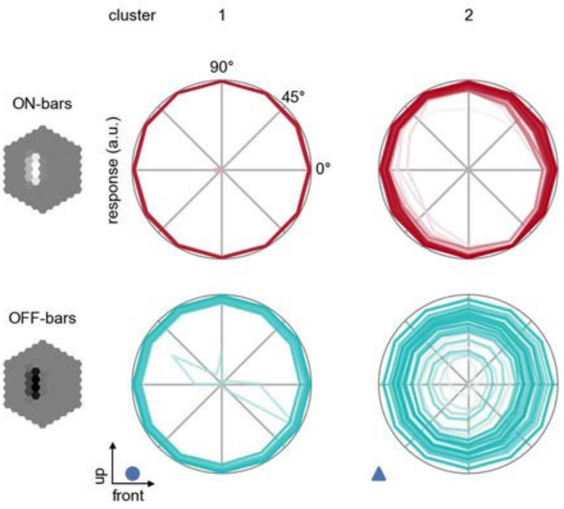

Mi11 - Figure 8: **Peak responses to moving bars.** The top row shows peak responses to moving ON-bars (red), the bottom row shows peak responses to moving OFF-bars (turqoise). The peak responses are averaged over bar-speeds. Bar-stimuli move in different directions from 0 to 360 degrees. The responses from the different models in the different clusters (columns) overlay. Responses from better task-performing models are more saturated.

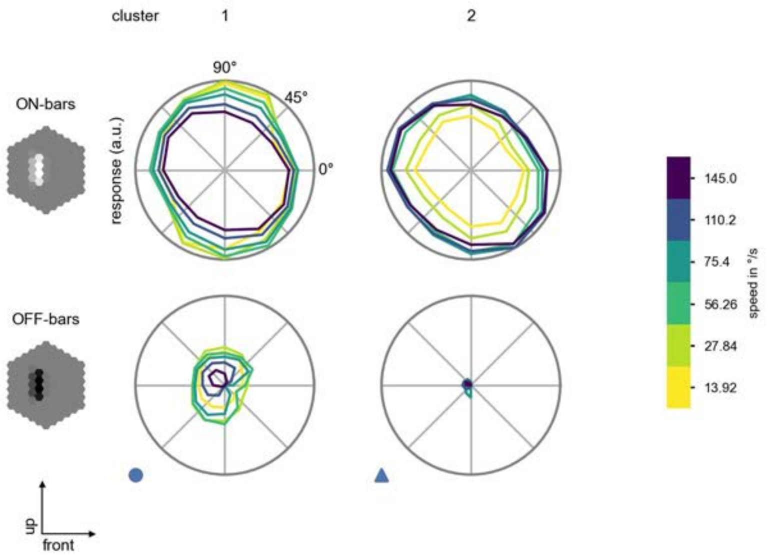

Mi11 - Figure 9: **Peak responses to moving bars from task-optimal models.** The top row shows peak responses to moving ON-bars, the bottom row shows peak responses to moving OFF-bars of varying speeds from 13.92°/s to 145°/s (yellow to dark blue). The bar-stimuli move in different directions from 0 to 360 degrees and at different speeds. Responses from the task-optimal model in the respective cluster.

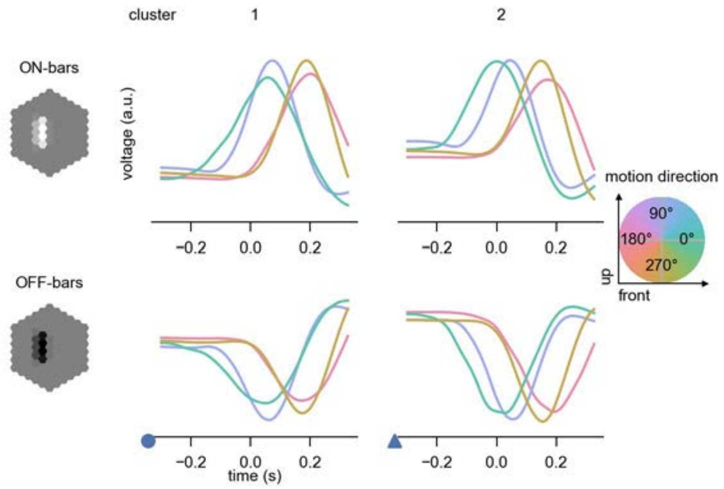

Mi11 - Figure 10: **Responses to moving bars from task-optimal models.** Responses to moving ON-bars (top row) and to moving OFF-bars (bottom row). Bars move in different directions from 0 to 360 degrees and at different speeds. Responses are from the task-optimal model in the respective cluster. Bars moving at 75.4°/s in all cardinal directions (green 0°, blue 90°, red 180°, yellow 270°) from -22.5 to 22.5° visual angle.

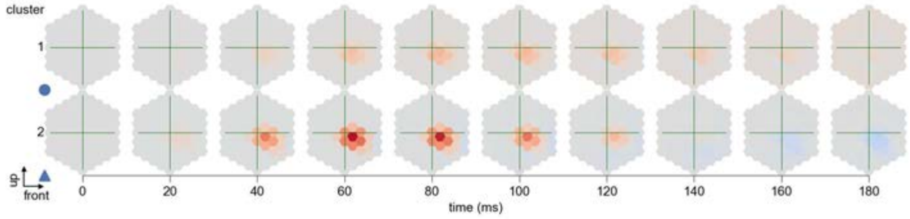

Mi11 - Figure 11: **Spatio-temporal receptive field.** Responses of the central cell to ON-impulses (5 ms) at single-ommatidium flash locations. The flash occurs at second zero. Responses from the task-optimal model of the respective cluster (rows). Red indicates depolarization, blue indicates hyperpolarization.

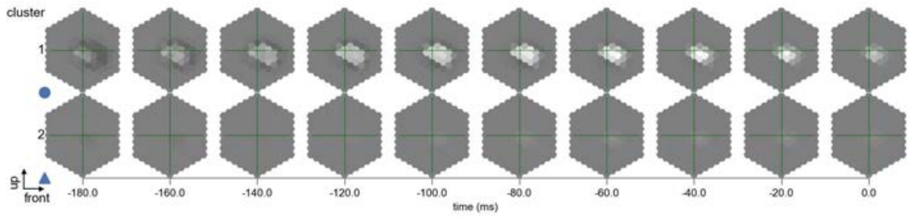

Mi11 - Figure 12: **Maximally excitatory stimuli.** Each row presents the regularized naturalistic-stimulus from the Sintel dataset that maximizes the cell type's central column response at second zero in the task-optimal model of the respective cluster (rows).

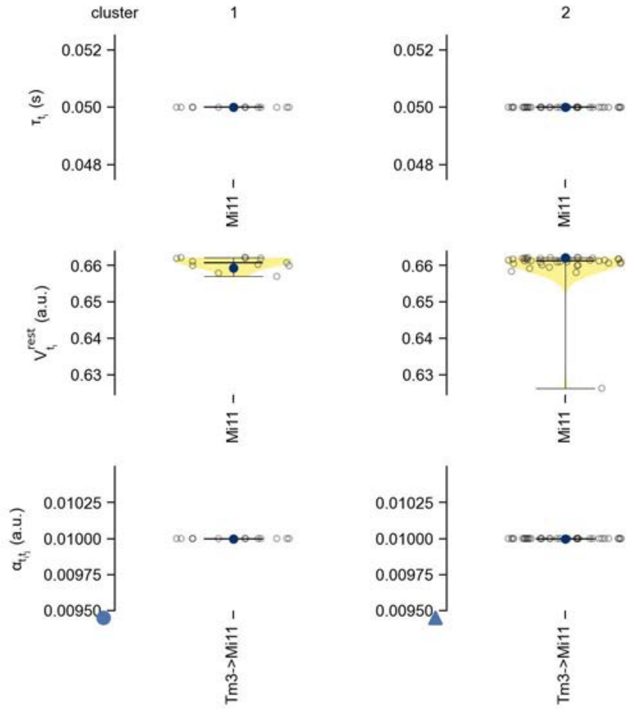

Mi11 - Figure 13: **Task-constrained parameters.** Each column shows the parameters inferred within the respective cluster. First row: learned time constants of the cell type. Second row: resting potentials of the cell type. Third row: scaling factors for the convolutional filters. The blue scatter represents the parameters from the task-optimal model within the cluster.

28 Mi12

← Cell types

Figures

1 Anatomical receptive fields. 195
2 Anatomical projective fields. 195
3 Clustering of the responses to naturalistic stimuli. 196
4 Responses to flashes. 196
5 Cluster-average responses to single-ommatidium flashes. 196
6 Peak responses to moving edges. 197
7 Peak responses to moving edges from task-optimal models. 197
8 Responses to moving edges from task-optimal models. 198
9 Peak responses to moving bars. 198
10 Peak responses to moving bars from task-optimal models. 199
11 Responses to moving bars from task-optimal models. 199
12 Spatio-temporal receptive field. 200
13 Maximally excitatory stimuli. 200
14 Task-constrained parameters. 201

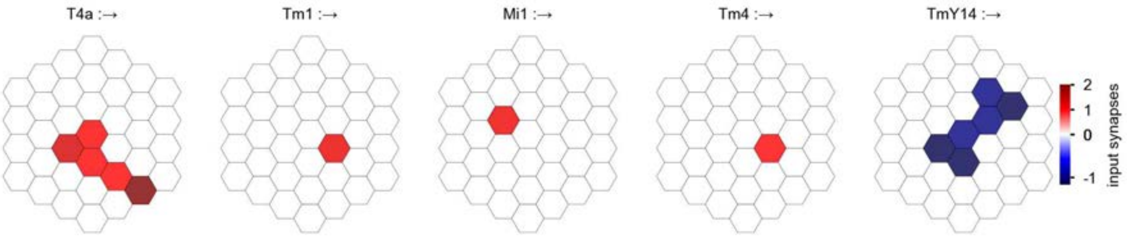

Mi12 - Figure 1: **Anatomical receptive fields.** Each colored hexagon is an input connection, with the connection strength characterized by the average number of synapses that we count from the EM reconstruction. Red indicates excitatory synapses, blue indicates inhibitory synapses from inferred signs. Filters in the order of their total number of synapses.

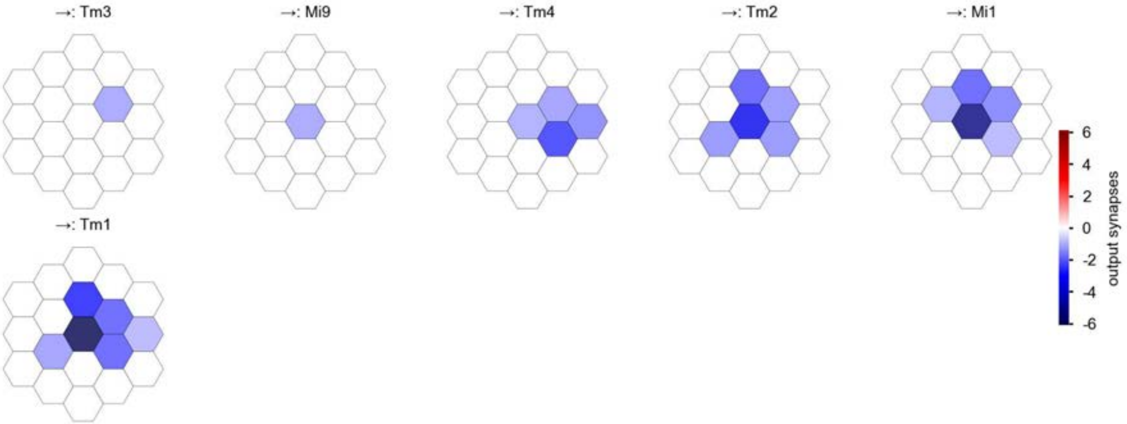

Mi12 - Figure 2: **Anatomical projective fields.** Each colored hexagon is an output connection, with the connection strength characterized by the average number of synapses that we count from the EM reconstruction. Red indicates excitatory synapses, blue indicates inhibitory synapses from inferred signs. Filters in the order of their total number of synapses.

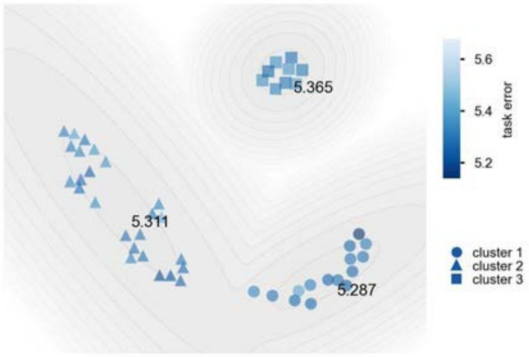

Mi12 - Figure 3: **Clustering of the responses to naturalistic stimuli.** Clustering of the 50 models based on the cell type responses to naturalistic scenes from the Sintel dataset. Scatterpoints represent individual models colored by their task error.

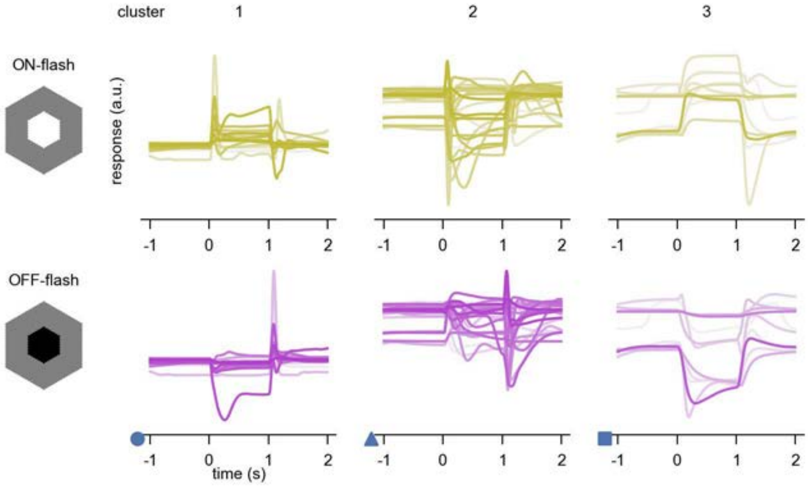

Mi12 - Figure 4: **Responses to flashes.** The top row shows responses to ON-flashes (yellow), the bottom row shows responses to OFF-flashes (magenta). The responses from the 50 different models that are separated into the different clusters (columns) overlay, with better task-performing models on top. Responses from better task-performing models are more saturated. The circular flashes (1s) cover 6 ommatidia in radius and are presented at time zero. Before and after, a grey-stimulus leads to a stationary state of the network.

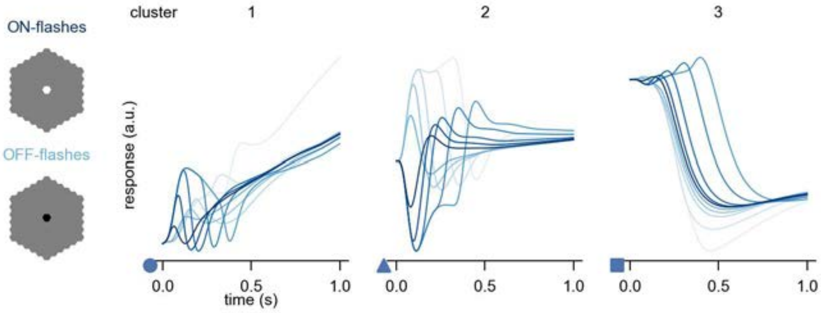

Mi12 - Figure 5: **Cluster-average responses to single-ommatidium flashes.** Responses to single-ommatidium ON-flashes (dark blue shades) and single-ommatidium OFF-flashes (light blue shades) of 20ms, 50ms, 100ms, 200ms, 300ms duration. The flashes occur at second zero.

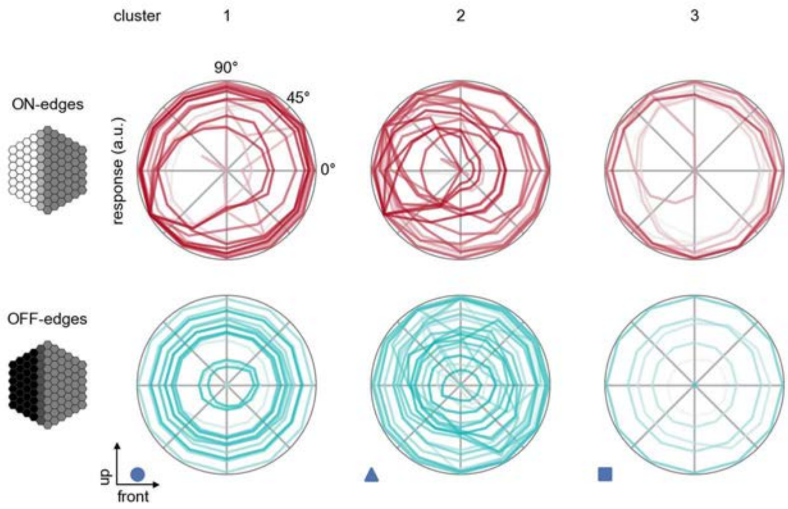

Mi12 - Figure 6: **Peak responses to moving edges.** The top row shows peak responses to moving ON-edges (red), the bottom row shows peak responses to moving OFF-edges (turqoise). The peak responses are averaged over edge-speeds. Edge-stimuli move in different directions from 0 to 360 degrees. The responses from the different models in the different clusters (columns) overlay. Responses from better task-performing models are more saturated.

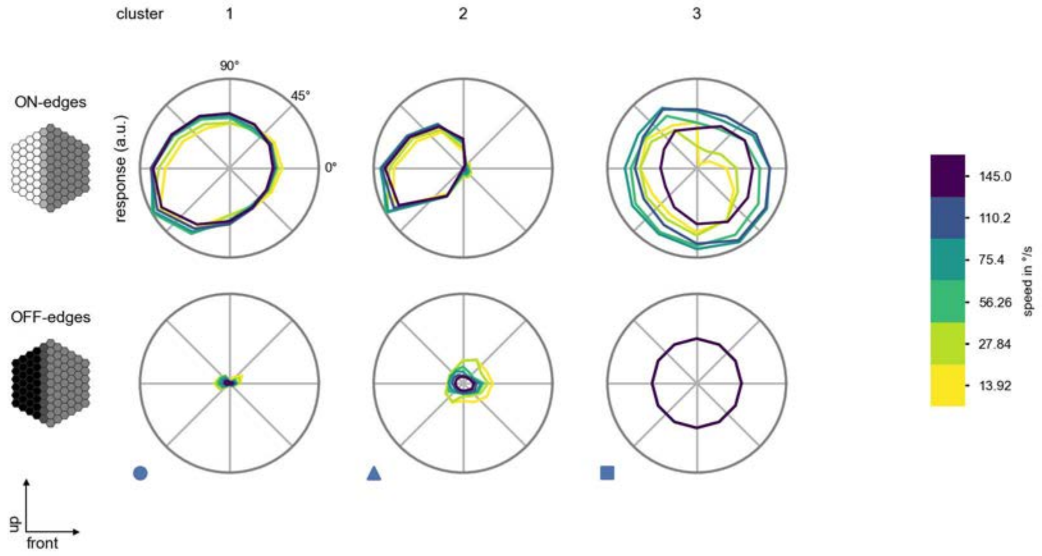

Mi12 - Figure 7: **Peak responses to moving edges from task-optimal models.** The top row shows peak responses to moving ON-edges, the bottom row shows peak responses to moving OFF-edges of varying speeds from 13.92°/s to 145°/s (yellow to dark blue). The edge-stimuli move in different directions from 0 to 360 degrees and at different speeds. Responses from the task-optimal model in the respective cluster.

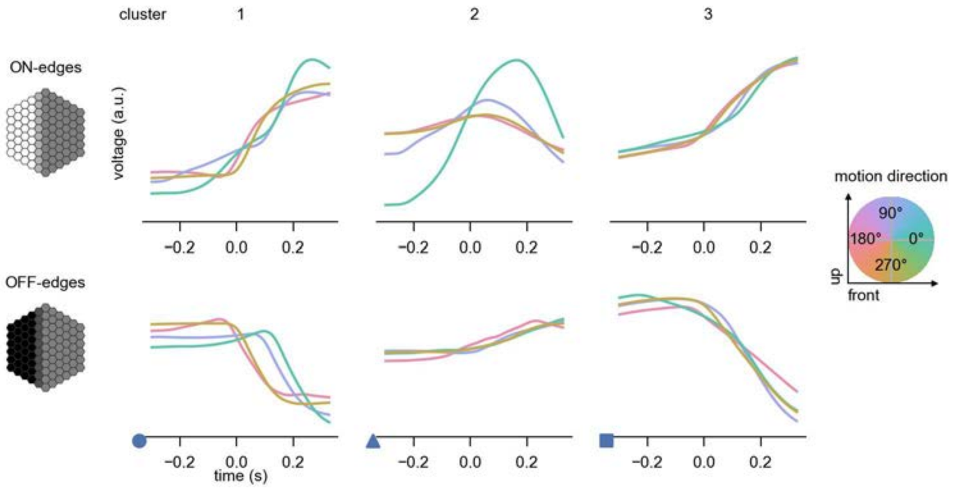

Mi12 - Figure 8: **Responses to moving edges from task-optimal models.** Responses to moving ON-edges (top row) and to moving OFF-edges (bottom row). Edges move in different directions from 0 to 360 degrees and at different speeds. Responses are from the task-optimal model in the respective cluster. Edges moving at 75.4°/s in all cardinal directions (green 0°, blue 90°, red 180°, yellow 270°) from -22.5 to 22.5° visual angle.

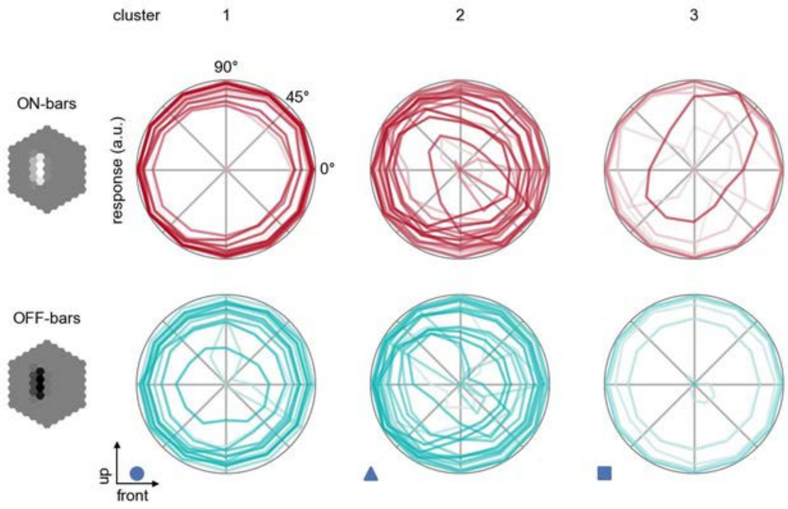

Mi12 - Figure 9: **Peak responses to moving bars.** The top row shows peak responses to moving ON-bars (red), the bottom row shows peak responses to moving OFF-bars (turqoise). The peak responses are averaged over bar-speeds. Bar-stimuli move in different directions from 0 to 360 degrees. The responses from the different models in the different clusters (columns) overlay. Responses from better task-performing models are more saturated.

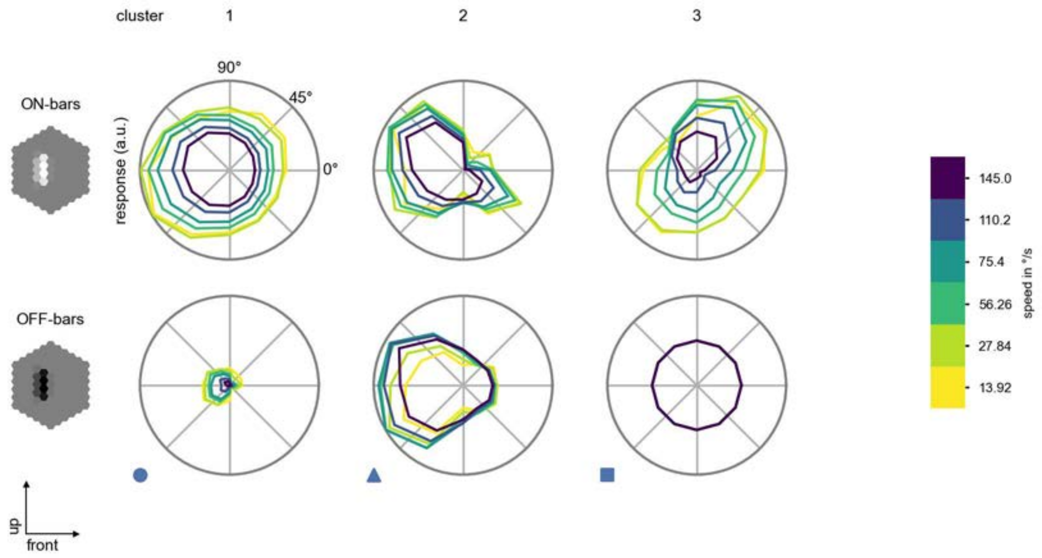

Mi12 - Figure 10: **Peak responses to moving bars from task-optimal models.** The top row shows peak responses to moving ON-bars, the bottom row shows peak responses to moving OFF-bars of varying speeds from 13.92°/s to 145°/s (yellow to dark blue). The bar-stimuli move in different directions from 0 to 360 degrees and at different speeds. Responses from the task-optimal model in the respective cluster.

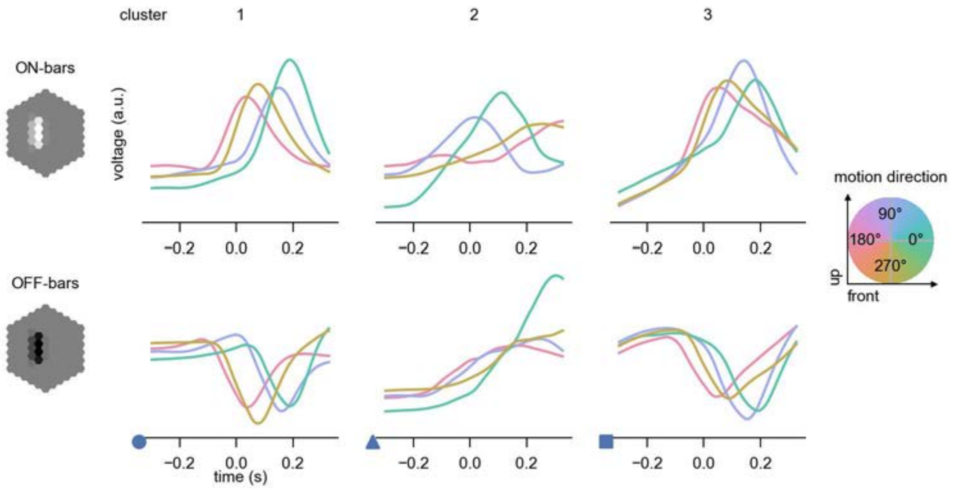

Mi12 - Figure 11: **Responses to moving bars from task-optimal models.** Responses to moving ON-bars (top row) and to moving OFF-bars (bottom row). Bars move in different directions from 0 to 360 degrees and at different speeds. Responses are from the task-optimal model in the respective cluster. Bars moving at 75.4°/s in all cardinal directions (green 0°, blue 90°, red 180°, yellow 270°) from -22.5 to 22.5° visual angle.

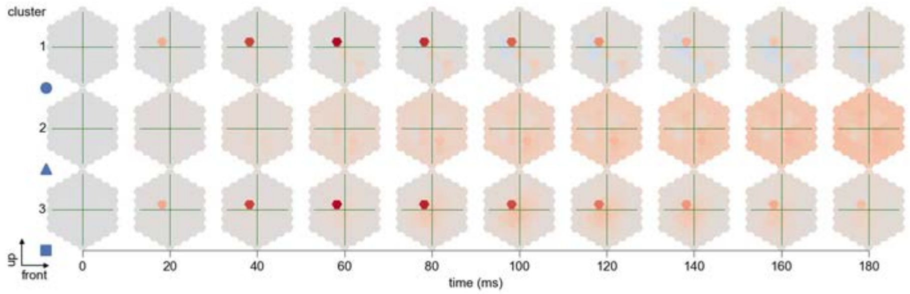

Mi12 - Figure 12: **Spatio-temporal receptive field.** Responses of the central cell to ON-impulses (5 ms) at single-ommatidium flash locations. The flash occurs at second zero. Responses from the task-optimal model of the respective cluster (rows). Red indicates depolarization, blue indicates hyperpolarization.

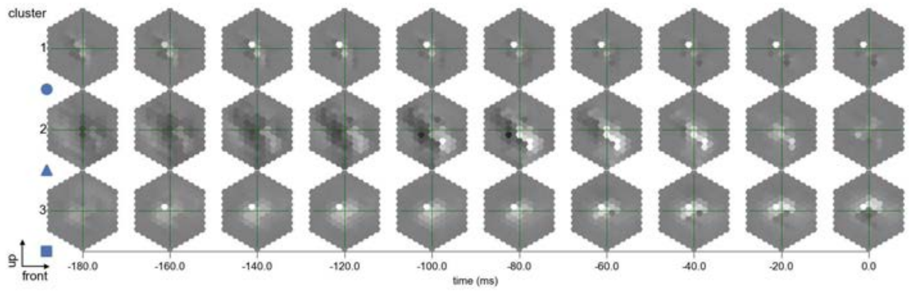

Mi12 - Figure 13: **Maximally excitatory stimuli.** Each row presents the regularized naturalistic-stimulus from the Sintel dataset that maximizes the cell type's central column response at second zero in the task-optimal model of the respective cluster (rows).

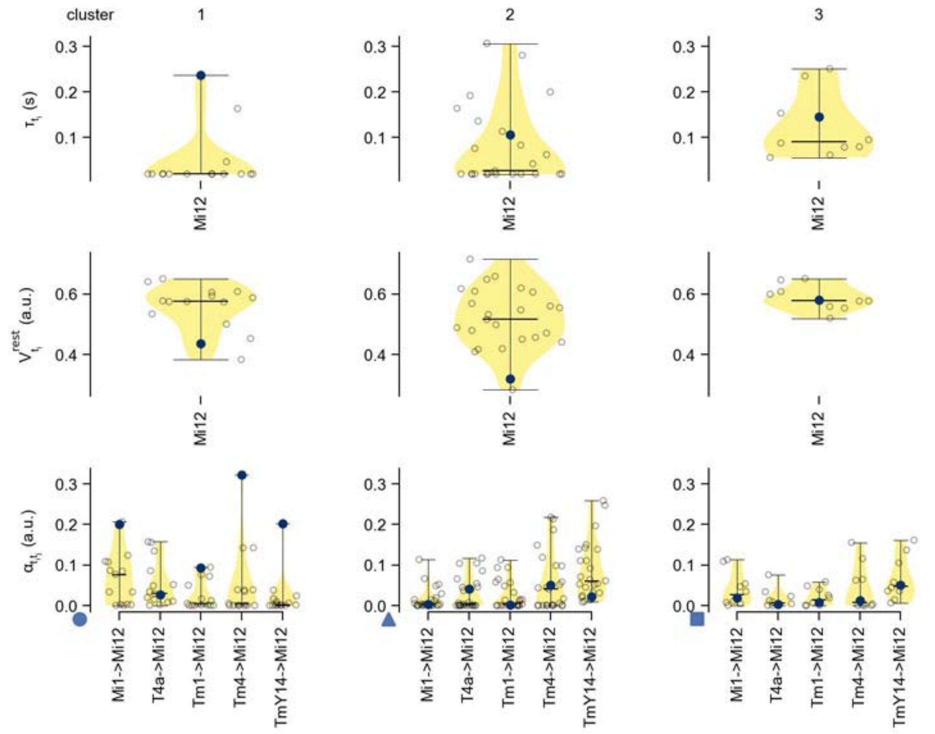

Mi12 - Figure 14: **Task-constrained parameters.** Each column shows the parameters inferred within the respective cluster. First row: learned time constants of the cell type. Second row: resting potentials of the cell type. Third row: scaling factors for the convolutional filters. The blue scatter represents the parameters from the task-optimal model within the cluster.

29 Mi13

← Cell types

Figures

1 Anatomical receptive fields. . . . 202
2 Anatomical projective fields. . . . 203
3 Clustering of the responses to naturalistic stimuli. . . . 203
4 Responses to flashes. . . . 203
5 Cluster-average responses to single-ommatidium flashes. . . . 204
6 Peak responses to moving edges. . . . 204
7 Peak responses to moving edges from task-optimal models. . . . 204
8 Responses to moving edges from task-optimal models. . . . 205
9 Peak responses to moving bars. . . . 205
10 Peak responses to moving bars from task-optimal models. . . . 205
11 Responses to moving bars from task-optimal models. . . . 206
12 Spatio-temporal receptive field. . . . 206
13 Maximally excitatory stimuli. . . . 207
14 Task-constrained parameters. . . . 207

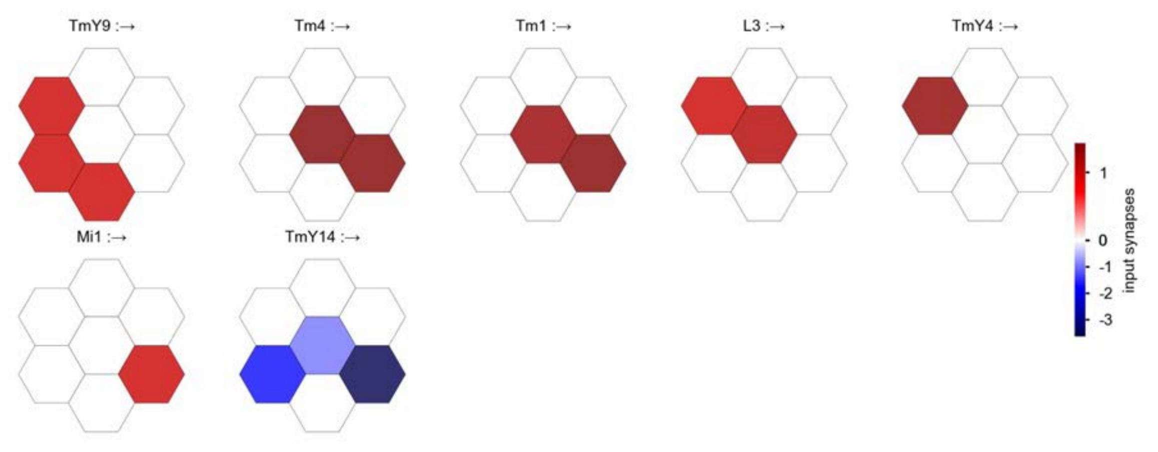

Mi13 - Figure 1: **Anatomical receptive fields.** Each colored hexagon is an input connection, with the connection strength characterized by the average number of synapses that we count from the EM reconstruction. Red indicates excitatory synapses, blue indicates inhibitory synapses from inferred signs. Filters in the order of their total number of synapses.

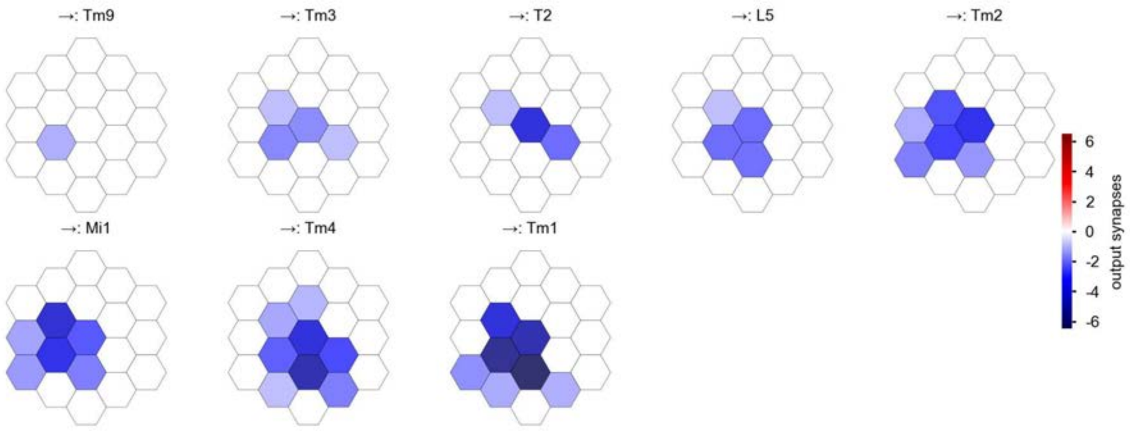

Mi13 - Figure 2: **Anatomical projective fields.** Each colored hexagon is an output connection, with the connection strength characterized by the average number of synapses that we count from the EM reconstruction. Red indicates excitatory synapses, blue indicates inhibitory synapses from inferred signs. Filters in the order of their total number of synapses.

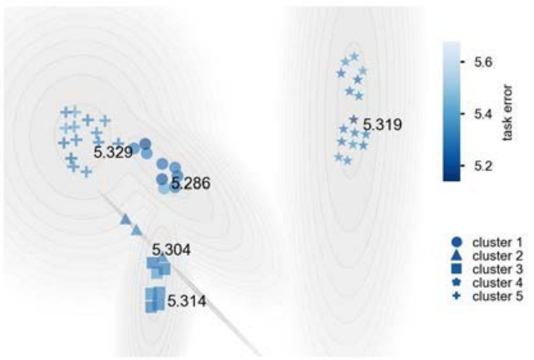

Mi13 - Figure 3: **Clustering of the responses to naturalistic stimuli.** Clustering of the 50 models based on the cell type responses to naturalistic scenes from the Sintel dataset. Scatterpoints represent individual models colored by their task error.

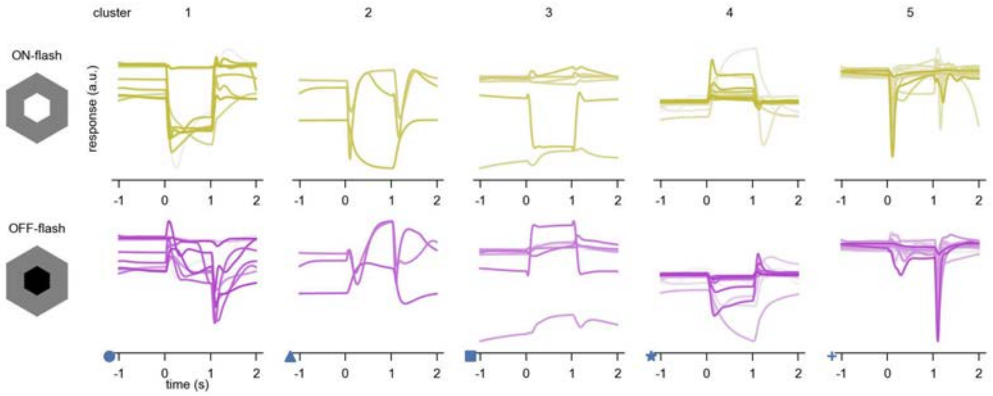

Mi13 - Figure 4: **Responses to flashes.** The top row shows responses to ON-flashes (yellow), the bottom row shows responses to OFF-flashes (magenta). The responses from the 50 different models that are separated into the different clusters (columns) overlay, with better task-performing models on top. Responses from better task-performing models are more saturated. The circular flashes (1s) cover 6 ommatidia in radius and are presented at time zero. Before and after, a grey-stimulus leads to a stationary state of the network.

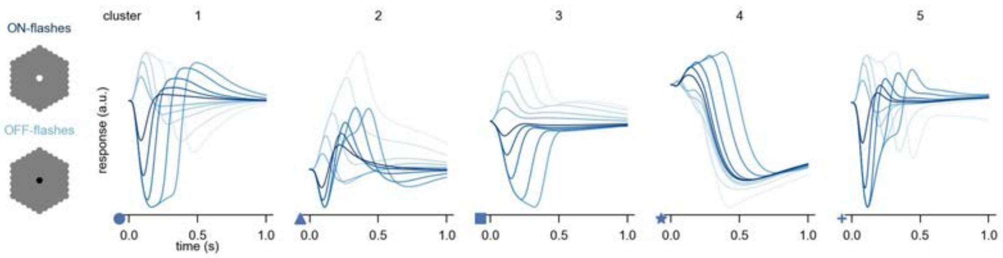

Mi13 - Figure 5: **Cluster-average responses to single-ommatidium flashes.** Responses to single-ommatidium ON-flashes (dark blue shades) and single-ommatidium OFF-flashes (light blue shades) of 20ms, 50ms, 100ms, 200ms, 300ms duration. The flashes occur at second zero.

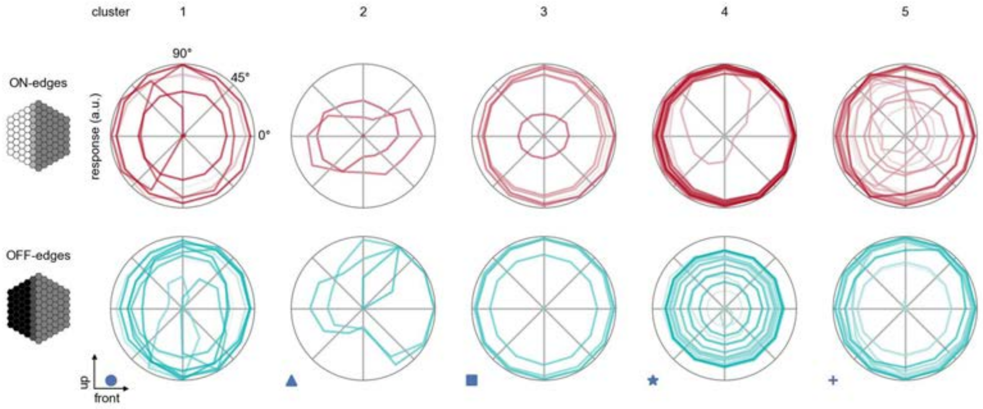

Mi13 - Figure 6: **Peak responses to moving edges.** The top row shows peak responses to moving ON-edges (red), the bottom row shows peak responses to moving OFF-edges (turqoise). The peak responses are averaged over edge-speeds. Edge-stimuli move in different directions from 0 to 360 degrees. The responses from the different models in the different clusters (columns) overlay. Responses from better task-performing models are more saturated.

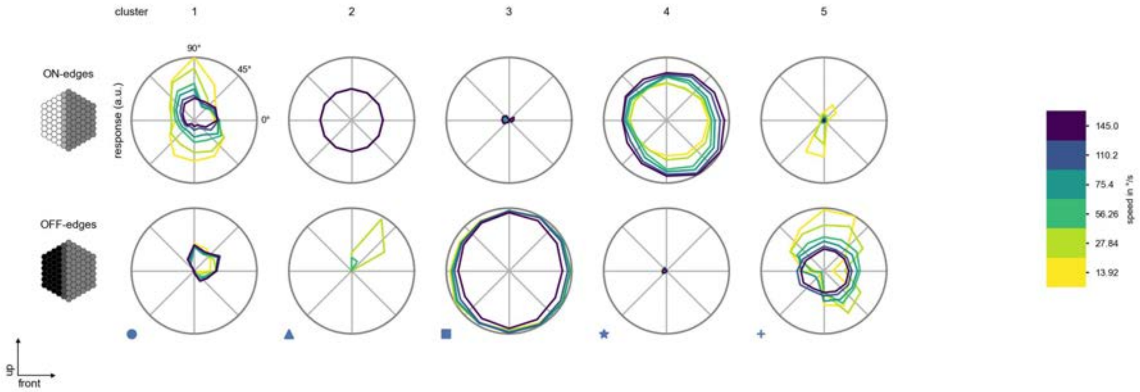

Mi13 - Figure 7: **Peak responses to moving edges from task-optimal models.** The top row shows peak responses to moving ON-edges, the bottom row shows peak responses to moving OFF-edges of varying speeds from 13.92°/s to 145°/s (yellow to dark blue). The edge-stimuli move in different directions from 0 to 360 degrees and at different speeds. Responses from the task-optimal model in the respective cluster.

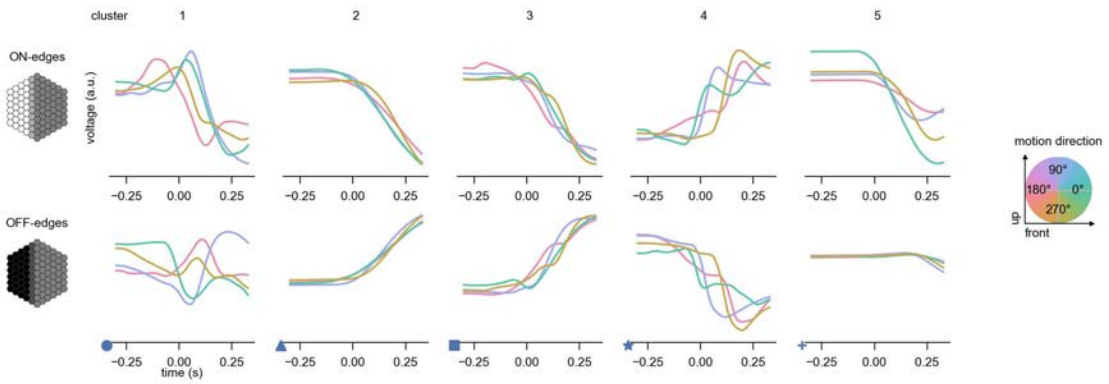

Mi13 - Figure 8: **Responses to moving edges from task-optimal models.** Responses to moving ON-edges (top row) and to moving OFF-edges (bottom row). Edges move in different directions from 0 to 360 degrees and at different speeds. Responses are from the task-optimal model in the respective cluster. Edges moving at 75.4°/s in all cardinal directions (green 0°, blue 90°, red 180°, yellow 270°) from -22.5 to 22.5° visual angle.

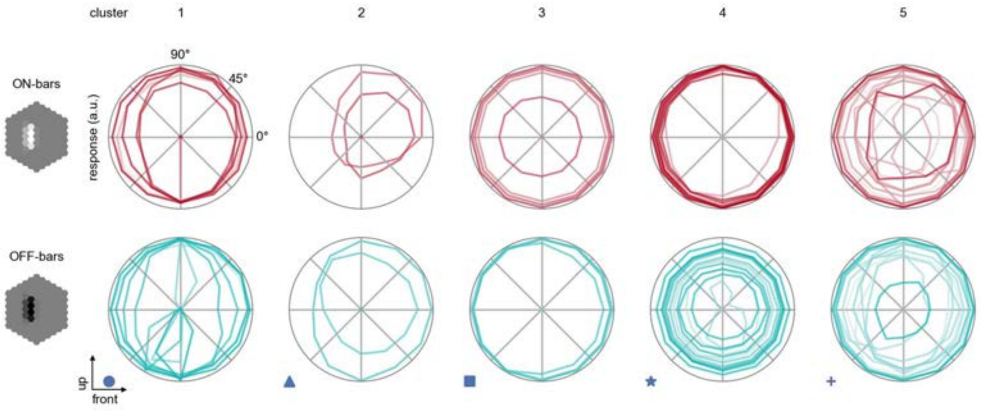

Mi13 - Figure 9: **Peak responses to moving bars.** The top row shows peak responses to moving ON-bars (red), the bottom row shows peak responses to moving OFF-bars (turqoise). The peak responses are averaged over bar-speeds. Bar-stimuli move in different directions from 0 to 360 degrees. The responses from the different models in the different clusters (columns) overlay. Responses from better task-performing models are more saturated.

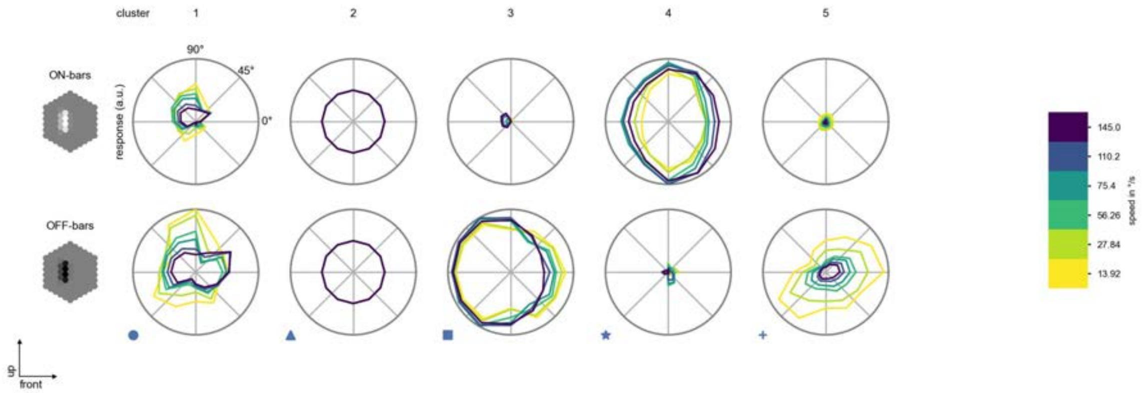

Mi13 - Figure 10: **Peak responses to moving bars from task-optimal models.** The top row shows peak responses to moving ON-bars, the bottom row shows peak responses to moving OFF-bars of varying speeds from 13.92°/s to 145°/s (yellow to dark blue). The bar-stimuli move in different directions from 0 to 360 degrees and at different speeds. Responses from the task-optimal model in the respective cluster.

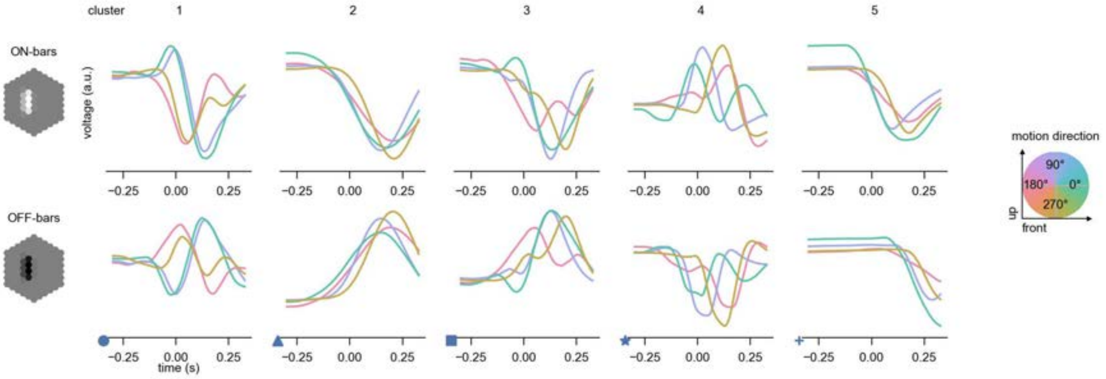

Mi13 - Figure 11: **Responses to moving bars from task-optimal models.** Responses to moving ON-bars (top row) and to moving OFF-bars (bottom row). Bars move in different directions from 0 to 360 degrees and at different speeds. Responses are from the task-optimal model in the respective cluster. Bars moving at 75.4°/s in all cardinal directions (green 0°, blue 90°, red 180°, yellow 270°) from -22.5 to 22.5° visual angle.

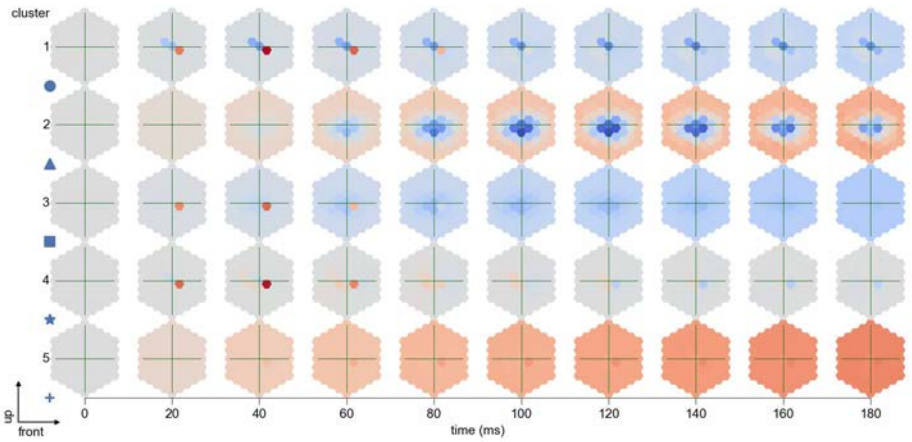

Mi13 - Figure 12: **Spatio-temporal receptive field.** Responses of the central cell to ON-impulses (5 ms) at single-ommatidium flash locations. The flash occurs at second zero. Responses from the task-optimal model of the respective cluster (rows). Red indicates depolarization, blue indicates hyperpolarization.

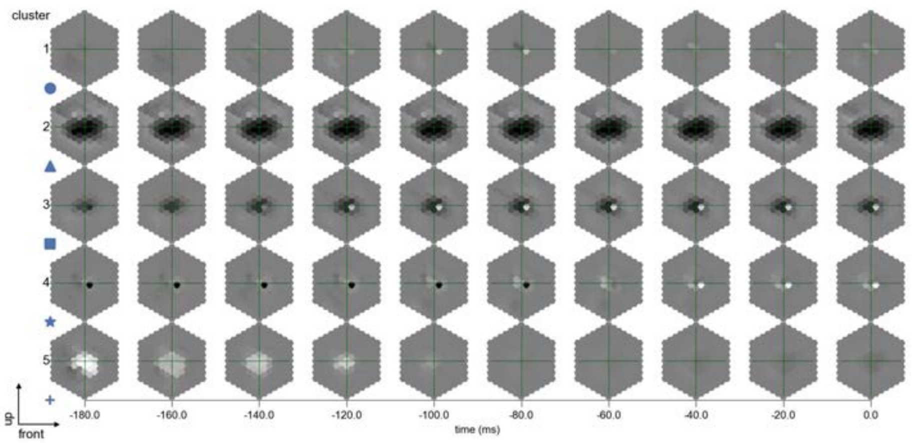

Mi13 - Figure 13: **Maximally excitatory stimuli.** Each row presents the regularized naturalistic-stimulus from the Sintel dataset that maximizes the cell type's central column response at second zero in the task-optimal model of the respective cluster (rows).

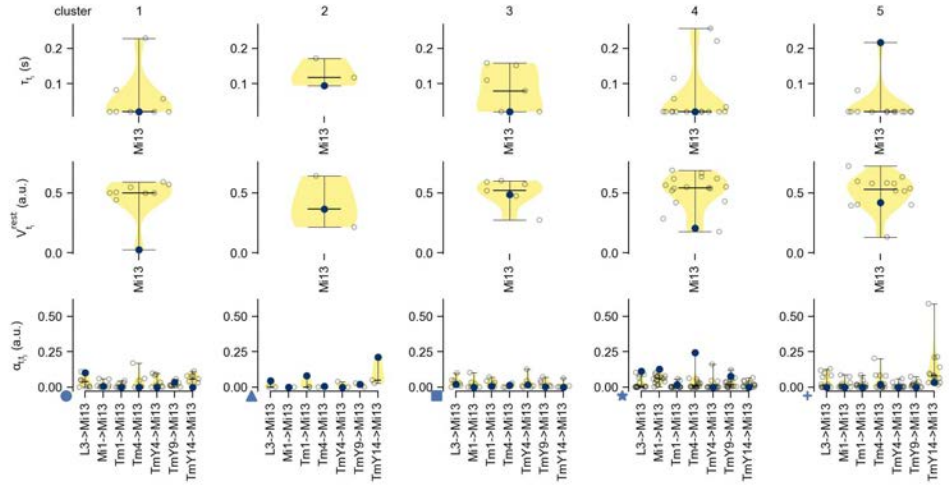

Mi13 - Figure 14: **Task-constrained parameters.** Each column shows the parameters inferred within the respective cluster. First row: learned time constants of the cell type. Second row: resting potentials of the cell type. Third row: scaling factors for the convolutional filters. The blue scatter represents the parameters from the task-optimal model within the cluster.

30 Mi14

← Cell types

Figures

1 Anatomical receptive fields. 208
2 Anatomical projective fields. 208
3 Clustering of the responses to naturalistic stimuli. 209
4 Responses to flashes. 209
5 Cluster-average responses to single-ommatidium flashes. 209
6 Peak responses to moving edges. 210
7 Peak responses to moving edges from task-optimal models. 210
8 Responses to moving edges from task-optimal models. 211
9 Peak responses to moving bars. 211
10 Peak responses to moving bars from task-optimal models. 212
11 Responses to moving bars from task-optimal models. 212
12 Spatio-temporal receptive field. 213
13 Maximally excitatory stimuli. 213
14 Task-constrained parameters. 214

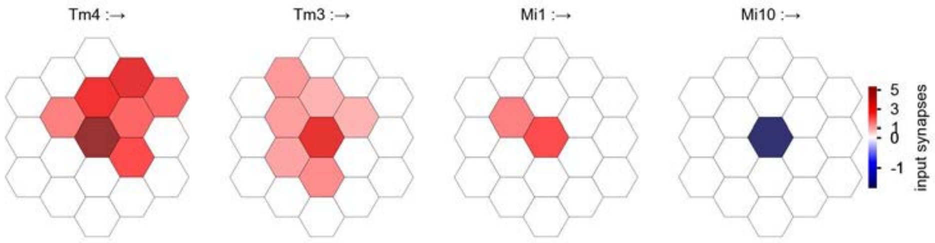

Mi14 - Figure 1: **Anatomical receptive fields.** Each colored hexagon is an input connection, with the connection strength characterized by the average number of synapses that we count from the EM reconstruction. Red indicates excitatory synapses, blue indicates inhibitory synapses from inferred signs. Filters in the order of their total number of synapses.

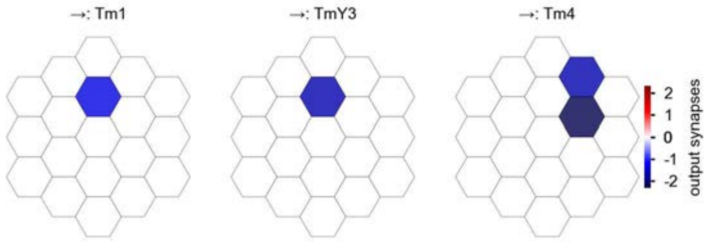

Mi14 - Figure 2: **Anatomical projective fields.** Each colored hexagon is an output connection, with the connection strength characterized by the average number of synapses that we count from the EM reconstruction. Red indicates excitatory synapses, blue indicates inhibitory synapses from inferred signs. Filters in the order of their total number of synapses.

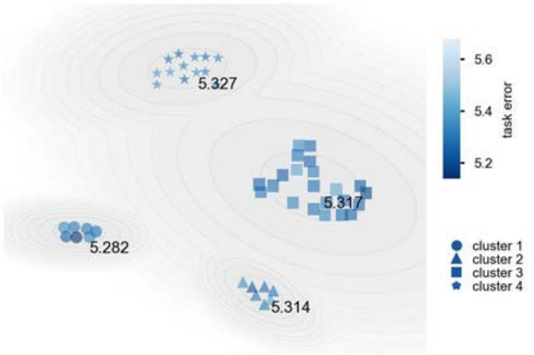

Mi14 - Figure 3: **Clustering of the responses to naturalistic stimuli.** Clustering of the 50 models based on the cell type responses to naturalistic scenes from the Sintel dataset. Scatterpoints represent individual models colored by their task error.

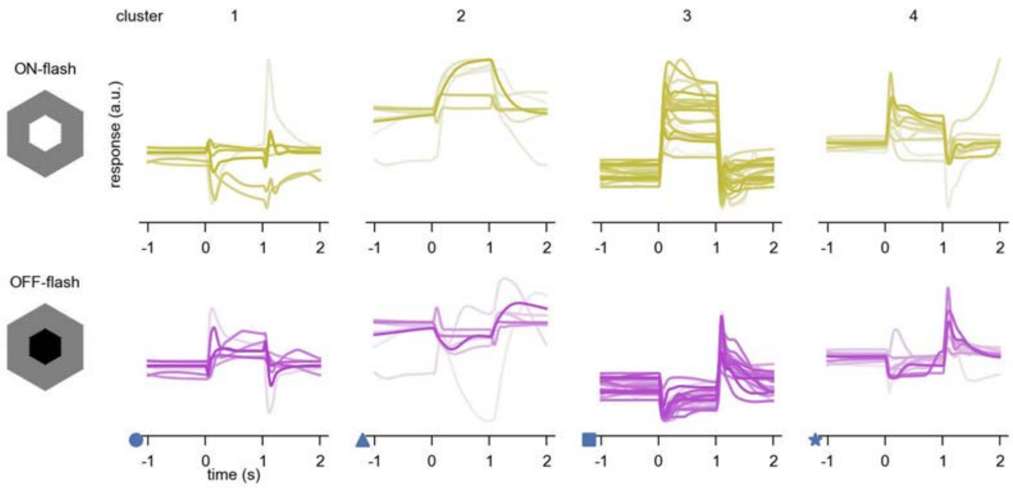

Mi14 - Figure 4: **Responses to flashes.** The top row shows responses to ON-flashes (yellow), the bottom row shows responses to OFF-flashes (magenta). The responses from the 50 different models that are separated into the different clusters (columns) overlay, with better task-performing models on top. Responses from better task-performing models are more saturated. The circular flashes (1s) cover 6 ommatidia in radius and are presented at time zero. Before and after, a grey-stimulus leads to a stationary state of the network.

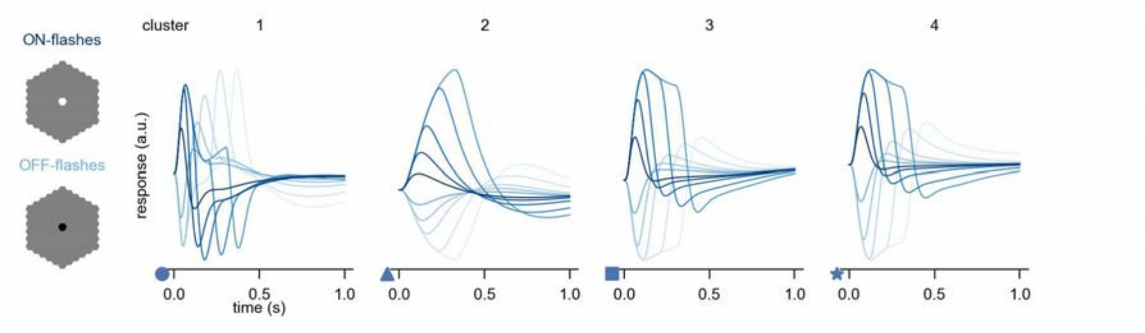

Mi14 - Figure 5: **Cluster-average responses to single-ommatidium flashes.** Responses to single-ommatidium ON-flashes (dark blue shades) and single-ommatidium OFF-flashes (light blue shades) of 20ms, 50ms, 100ms, 200ms, 300ms duration. The flashes occur at second zero.

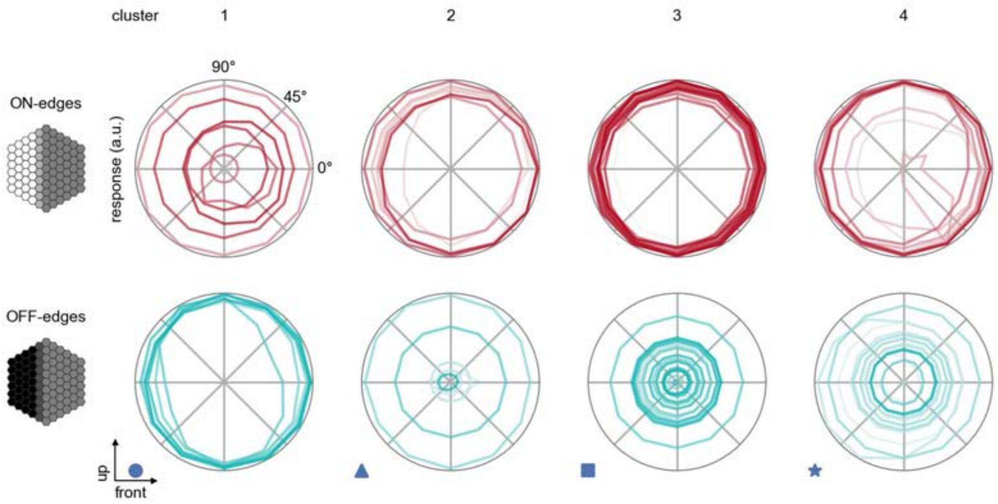

Mi14 - Figure 6: **Peak responses to moving edges.** The top row shows peak responses to moving ON-edges (red), the bottom row shows peak responses to moving OFF-edges (turqoise). The peak responses are averaged over edge-speeds. Edge-stimuli move in different directions from 0 to 360 degrees. The responses from the different models in the different clusters (columns) overlay. Responses from better task-performing models are more saturated.

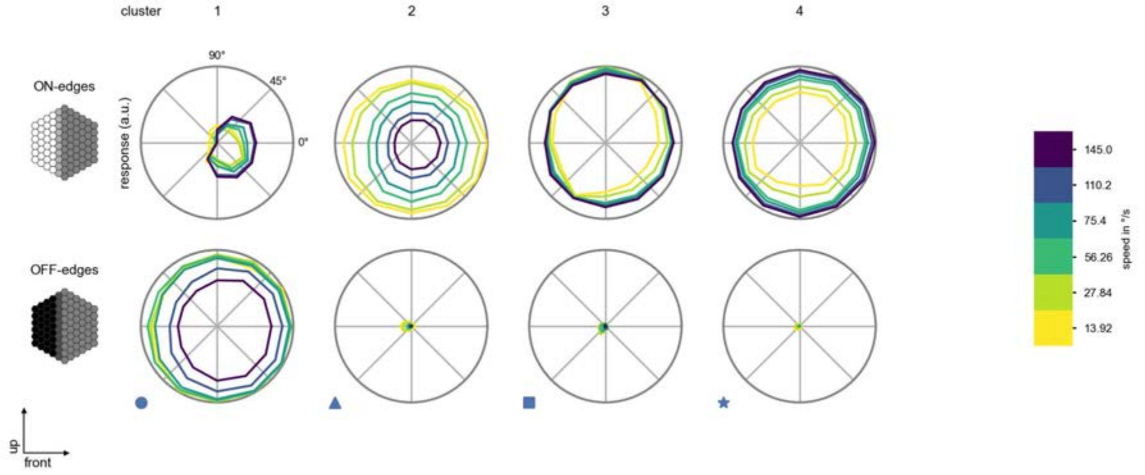

Mi14 - Figure 7: **Peak responses to moving edges from task-optimal models.** The top row shows peak responses to moving ON-edges, the bottom row shows peak responses to moving OFF-edges of varying speeds from 13.92°/s to 145°/s (yellow to dark blue). The edge-stimuli move in different directions from 0 to 360 degrees and at different speeds. Responses from the task-optimal model in the respective cluster.

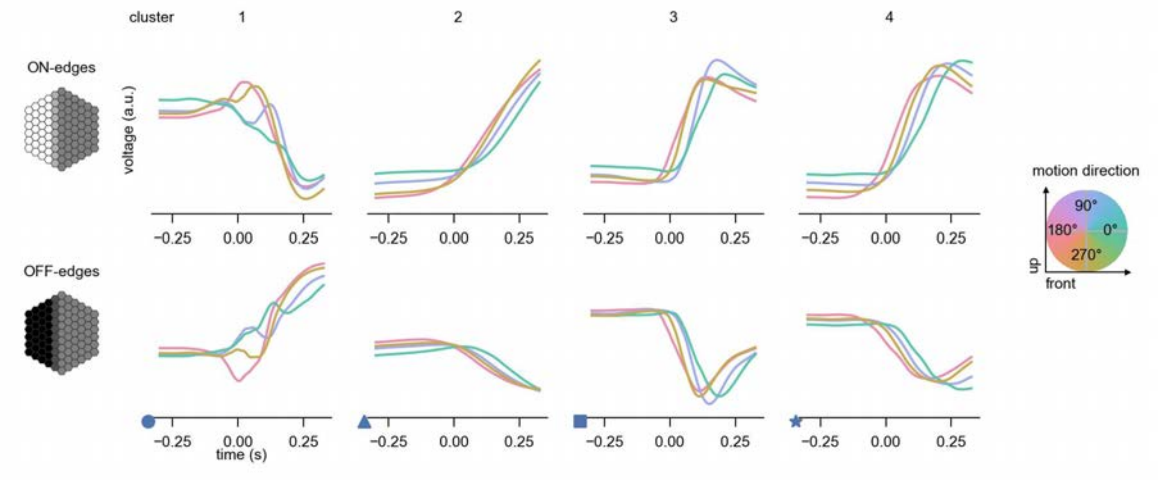

Mi14 - Figure 8: **Responses to moving edges from task-optimal models.** Responses to moving ON-edges (top row) and to moving OFF-edges (bottom row). Edges move in different directions from 0 to 360 degrees and at different speeds. Responses are from the task-optimal model in the respective cluster. Edges moving at 75.4°/s in all cardinal directions (green 0°, blue 90°, red 180°, yellow 270°) from -22.5 to 22.5° visual angle.

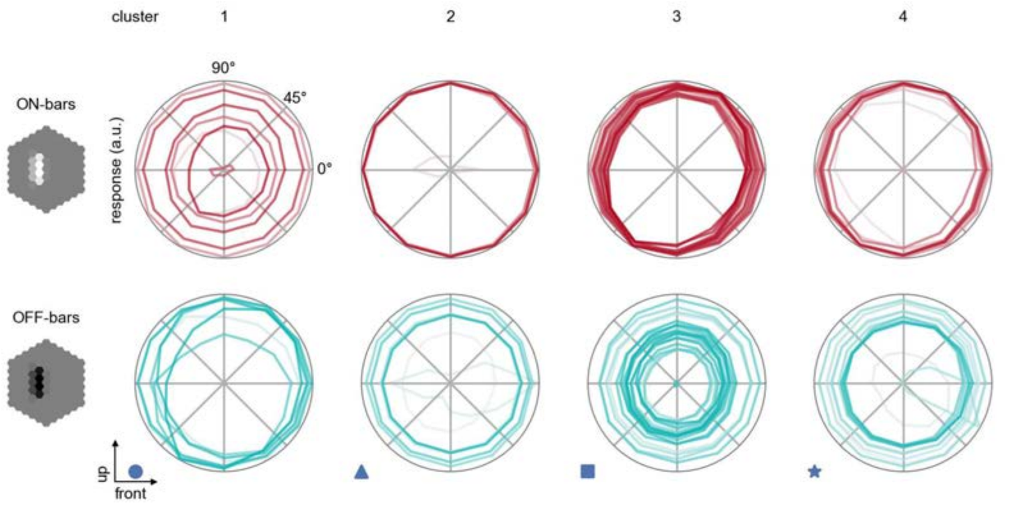

Mi14 - Figure 9: **Peak responses to moving bars.** The top row shows peak responses to moving ON-bars (red), the bottom row shows peak responses to moving OFF-bars (turqoise). The peak responses are averaged over bar-speeds. Bar-stimuli move in different directions from 0 to 360 degrees. The responses from the different models in the different clusters (columns) overlay. Responses from better task-performing models are more saturated.

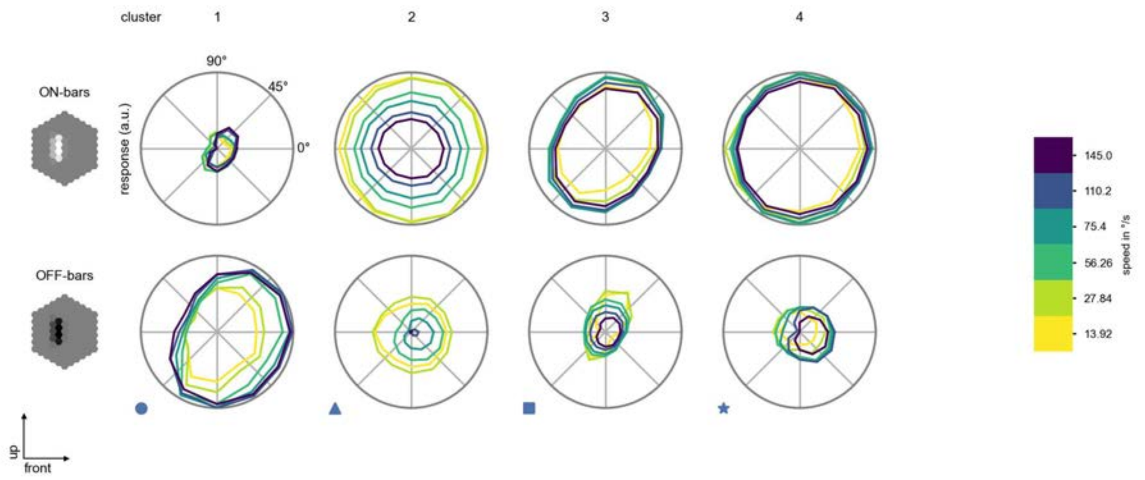

Mi14 - Figure 10: **Peak responses to moving bars from task-optimal models.** The top row shows peak responses to moving ON-bars, the bottom row shows peak responses to moving OFF-bars of varying speeds from 13.92°/s to 145°/s (yellow to dark blue). The bar-stimuli move in different directions from 0 to 360 degrees and at different speeds. Responses from the task-optimal model in the respective cluster.

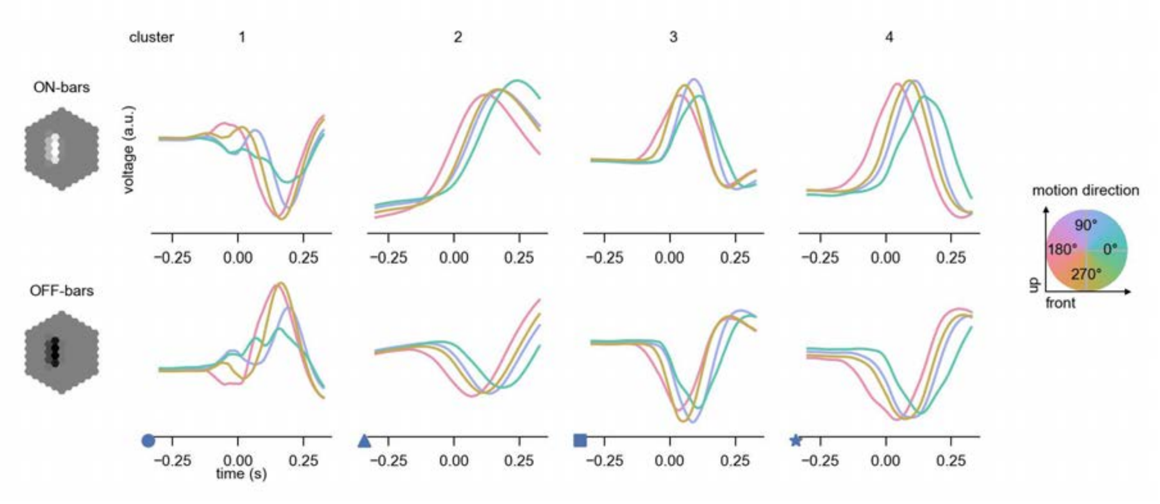

Mi14 - Figure 11: **Responses to moving bars from task-optimal models.** Responses to moving ON-bars (top row) and to moving OFF-bars (bottom row). Bars move in different directions from 0 to 360 degrees and at different speeds. Responses are from the task-optimal model in the respective cluster. Bars moving at 75.4°/s in all cardinal directions (green 0°, blue 90°, red 180°, yellow 270°) from -22.5 to 22.5° visual angle.

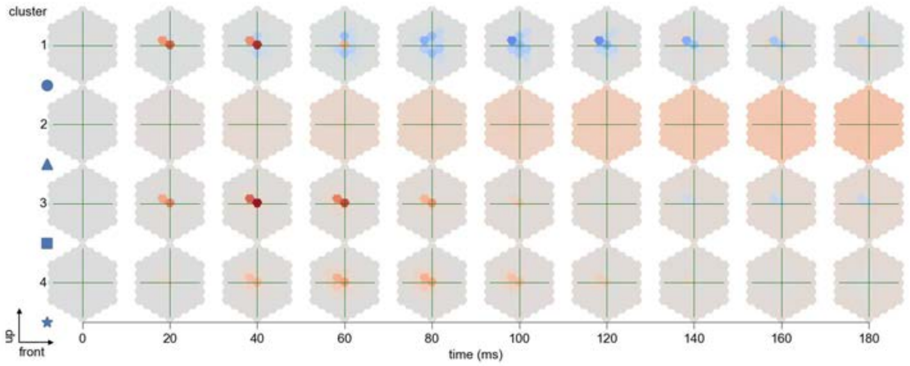

Mi14 - Figure 12: **Spatio-temporal receptive field.** Responses of the central cell to ON-impulses (5 ms) at single-ommatidium flash locations. The flash occurs at second zero. Responses from the task-optimal model of the respective cluster (rows). Red indicates depolarization, blue indicates hyperpolarization.

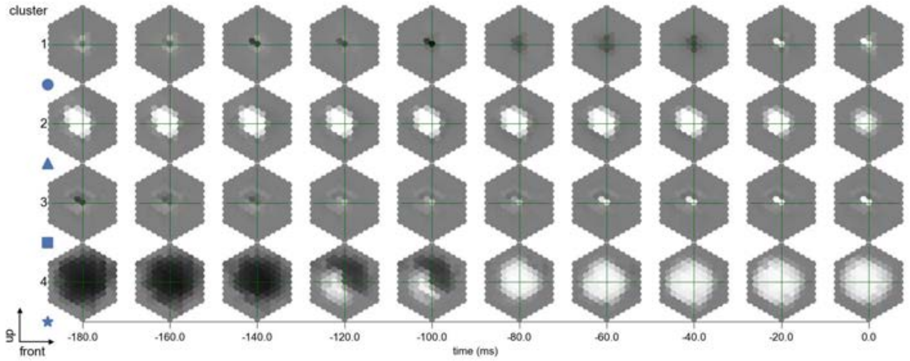

Mi14 - Figure 13: **Maximally excitatory stimuli.** Each row presents the regularized naturalistic-stimulus from the Sintel dataset that maximizes the cell type's central column response at second zero in the task-optimal model of the respective cluster (rows).

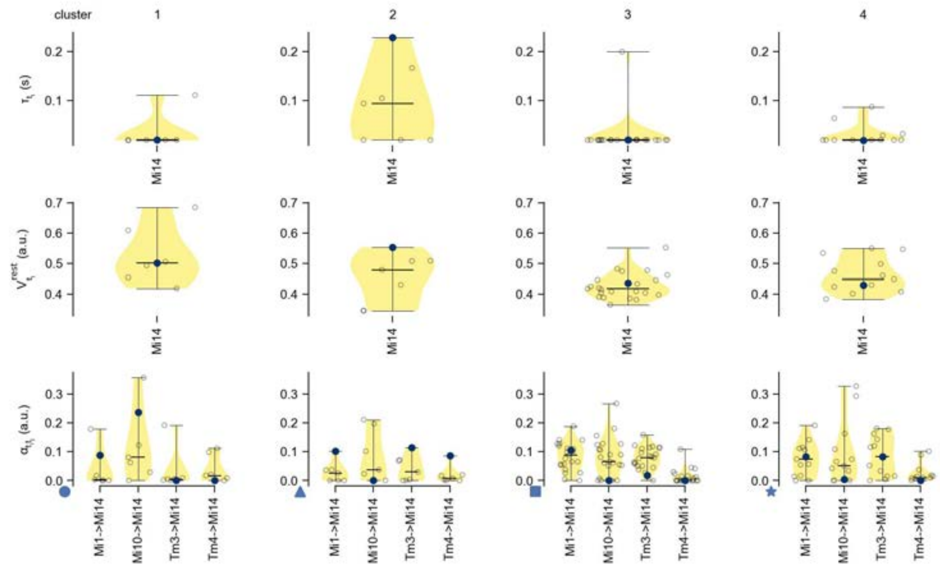

Mi14 - Figure 14: **Task-constrained parameters.** Each column shows the parameters inferred within the respective cluster. First row: learned time constants of the cell type. Second row: resting potentials of the cell type. Third row: scaling factors for the convolutional filters. The blue scatter represents the parameters from the task-optimal model within the cluster.

31 Mi15

← Cell types

Figures

1 Anatomical receptive fields. . . . 215
2 Anatomical projective fields. . . . 215
3 Clustering of the responses to naturalistic stimuli. . . . 216
4 Responses to flashes. . . . 216
5 Cluster-average responses to single-ommatidium flashes. . . . 216
6 Peak responses to moving edges. . . . 217
7 Peak responses to moving edges from task-optimal models. . . . 217
8 Responses to moving edges from task-optimal models. . . . 218
9 Peak responses to moving bars. . . . 218
10 Peak responses to moving bars from task-optimal models. . . . 219
11 Responses to moving bars from task-optimal models. . . . 219
12 Spatio-temporal receptive field. . . . 219
13 Maximally excitatory stimuli. . . . 220
14 Task-constrained parameters. . . . 220

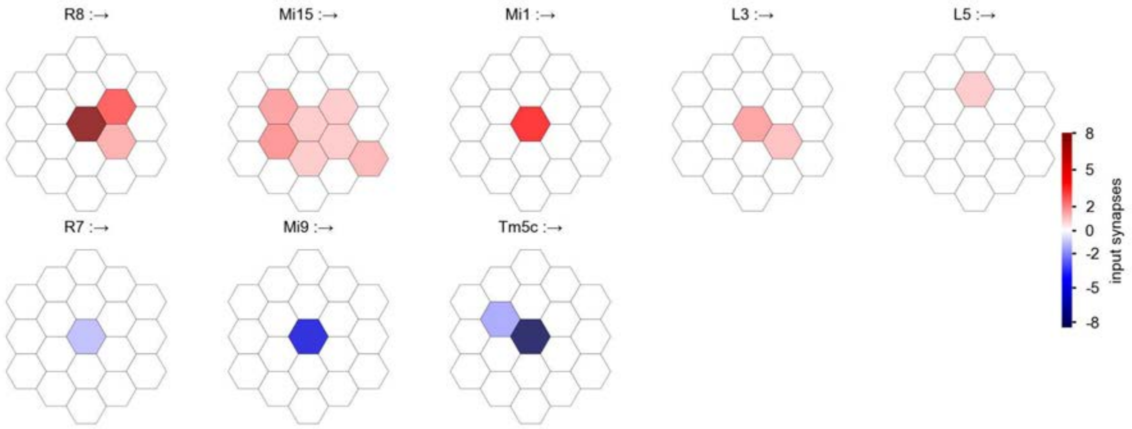

Mi15 - Figure 1: **Anatomical receptive fields.** Each colored hexagon is an input connection, with the connection strength characterized by the average number of synapses that we count from the EM reconstruction. Red indicates excitatory synapses, blue indicates inhibitory synapses from inferred signs. Filters in the order of their total number of synapses.

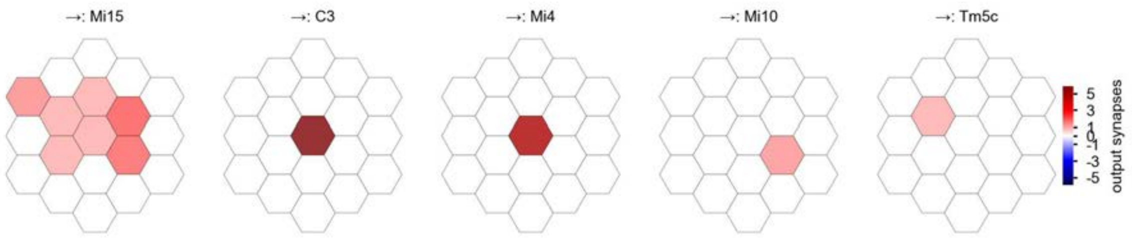

Mi15 - Figure 2: **Anatomical projective fields.** Each colored hexagon is an output connection, with the connection strength characterized by the average number of synapses that we count from the EM reconstruction. Red indicates excitatory synapses, blue indicates inhibitory synapses from inferred signs. Filters in the order of their total number of synapses.

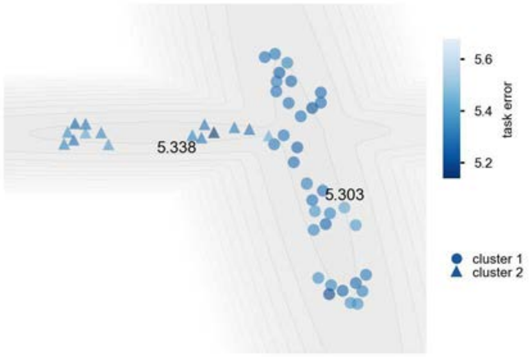

Mi15 - Figure 3: **Clustering of the responses to naturalistic stimuli.** Clustering of the 50 models based on the cell type responses to naturalistic scenes from the Sintel dataset. Scatterpoints represent individual models colored by their task error.

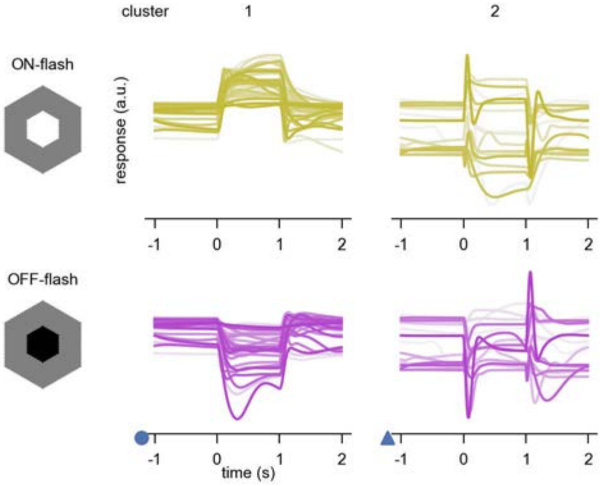

Mi15 - Figure 4: **Responses to flashes.** The top row shows responses to ON-flashes (yellow), the bottom row shows responses to OFF-flashes (magenta). The responses from the 50 different models that are separated into the different clusters (columns) overlay, with better task-performing models on top. Responses from better task-performing models are more saturated. The circular flashes (1s) cover 6 ommatidia in radius and are presented at time zero. Before and after, a grey-stimulus leads to a stationary state of the network.

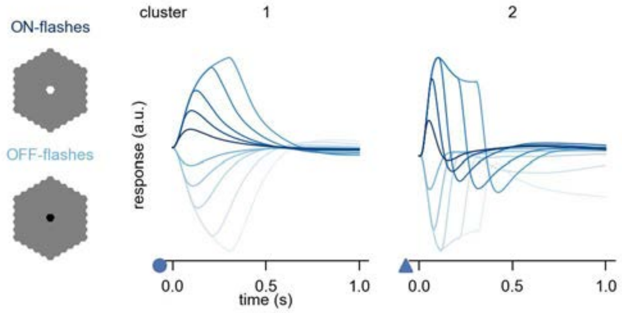

Mi15 - Figure 5: **Cluster-average responses to single-ommatidium flashes.** Responses to single-ommatidium ON-flashes (dark blue shades) and single-ommatidium OFF-flashes (light blue shades) of 20ms, 50ms, 100ms, 200ms, 300ms duration. The flashes occur at second zero.

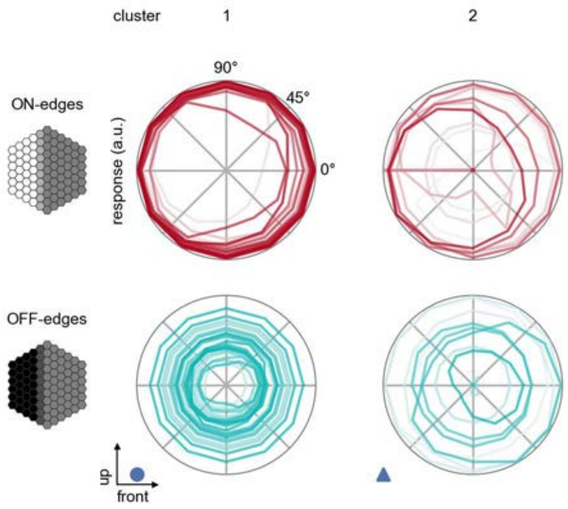

Mi15 - Figure 6: **Peak responses to moving edges.** The top row shows peak responses to moving ON-edges (red), the bottom row shows peak responses to moving OFF-edges (turqoise). The peak responses are averaged over edge-speeds. Edge-stimuli move in different directions from 0 to 360 degrees. The responses from the different models in the different clusters (columns) overlay. Responses from better task-performing models are more saturated.

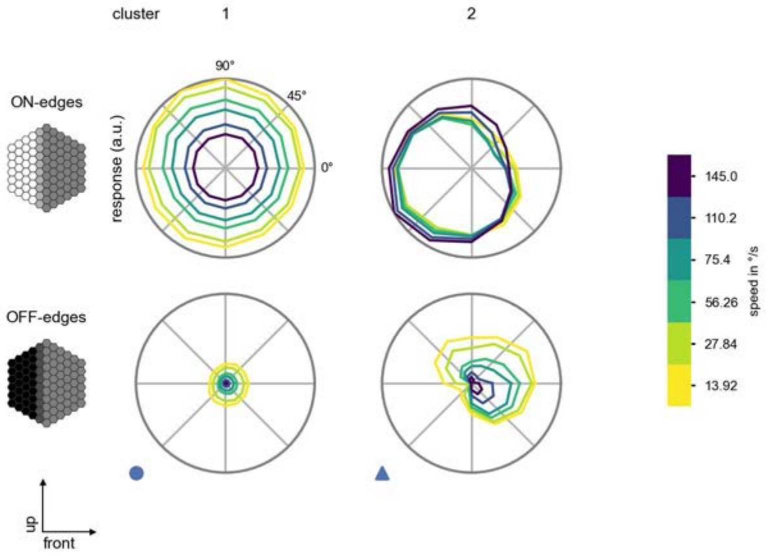

Mi15 - Figure 7: **Peak responses to moving edges from task-optimal models.** The top row shows peak responses to moving ON-edges, the bottom row shows peak responses to moving OFF-edges of varying speeds from 13.92°/s to 145°/s (yellow to dark blue). The edge-stimuli move in different directions from 0 to 360 degrees and at different speeds. Responses from the task-optimal model in the respective cluster.

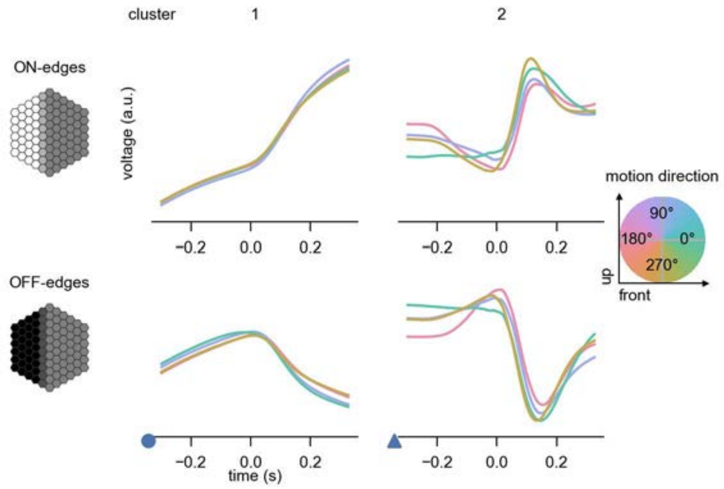

Mi15 - Figure 8: **Responses to moving edges from task-optimal models.** Responses to moving ON-edges (top row) and to moving OFF-edges (bottom row). Edges move in different directions from 0 to 360 degrees and at different speeds. Responses are from the task-optimal model in the respective cluster. Edges moving at 75.4°/s in all cardinal directions (green 0°, blue 90°, red 180°, yellow 270°) from -22.5 to 22.5° visual angle.

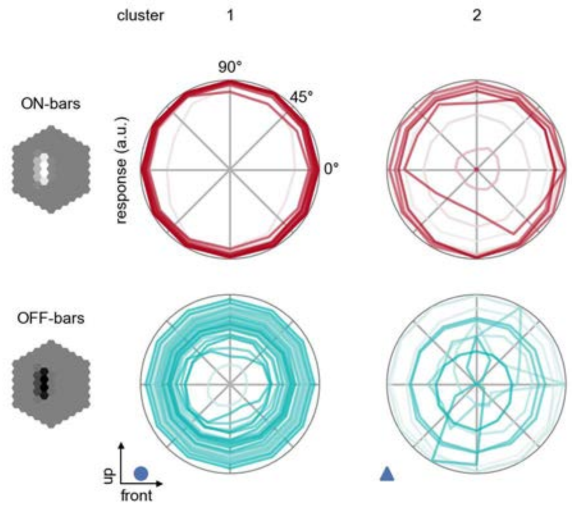

Mi15 - Figure 9: **Peak responses to moving bars.** The top row shows peak responses to moving ON-bars (red), the bottom row shows peak responses to moving OFF-bars (turqoise). The peak responses are averaged over bar-speeds. Bar-stimuli move in different directions from 0 to 360 degrees. The responses from the different models in the different clusters (columns) overlay. Responses from better task-performing models are more saturated.

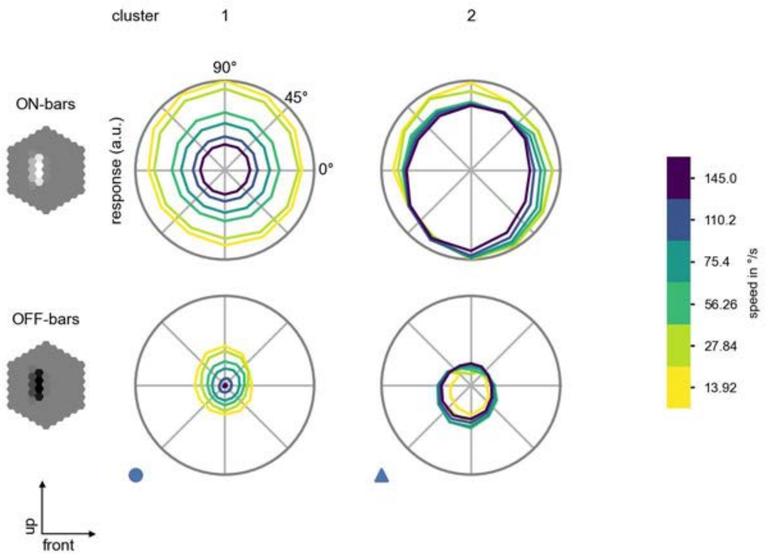

Mi15 - Figure 10: **Peak responses to moving bars from task-optimal models.** The top row shows peak responses to moving ON-bars, the bottom row shows peak responses to moving OFF-bars of varying speeds from 13.92°/s to 145°/s (yellow to dark blue). The bar-stimuli move in different directions from 0 to 360 degrees and at different speeds. Responses from the task-optimal model in the respective cluster.

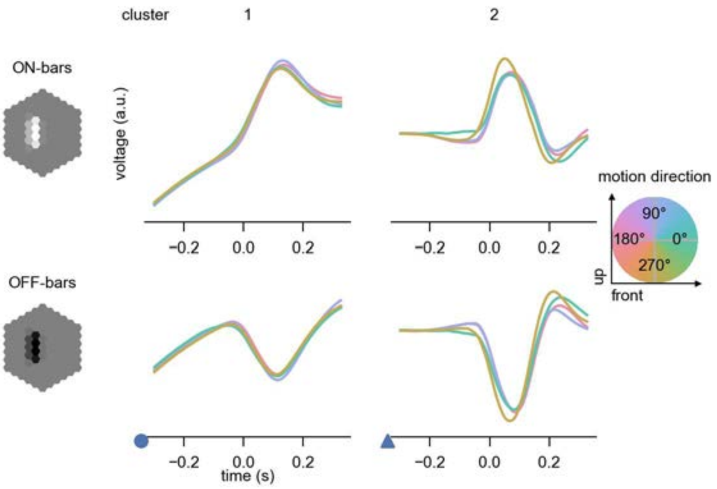

Mi15 - Figure 11: **Responses to moving bars from task-optimal models.** Responses to moving ON-bars (top row) and to moving OFF-bars (bottom row). Bars move in different directions from 0 to 360 degrees and at different speeds. Responses are from the task-optimal model in the respective cluster. Bars moving at 75.4°/s in all cardinal directions (green 0°, blue 90°, red 180°, yellow 270°) from -22.5 to 22.5° visual angle.

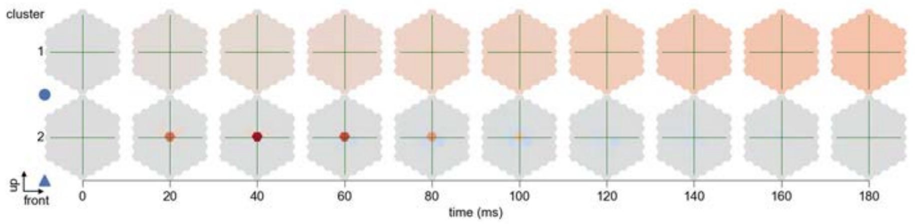

Mi15 - Figure 12: **Spatio-temporal receptive field.** Responses of the central cell to ON-impulses (5 ms) at single-ommatidium flash locations. The flash occurs at second zero. Responses from the task-optimal model of the respective cluster (rows). Red indicates depolarization, blue indicates hyperpolarization.

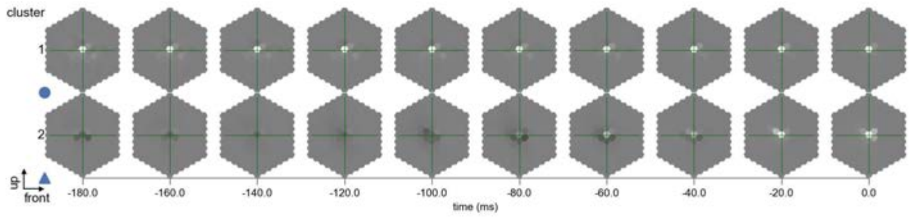

Mi15 - Figure 13: **Maximally excitatory stimuli.** Each row presents the regularized naturalistic-stimulus from the Sintel dataset that maximizes the cell type's central column response at second zero in the task-optimal model of the respective cluster (rows).

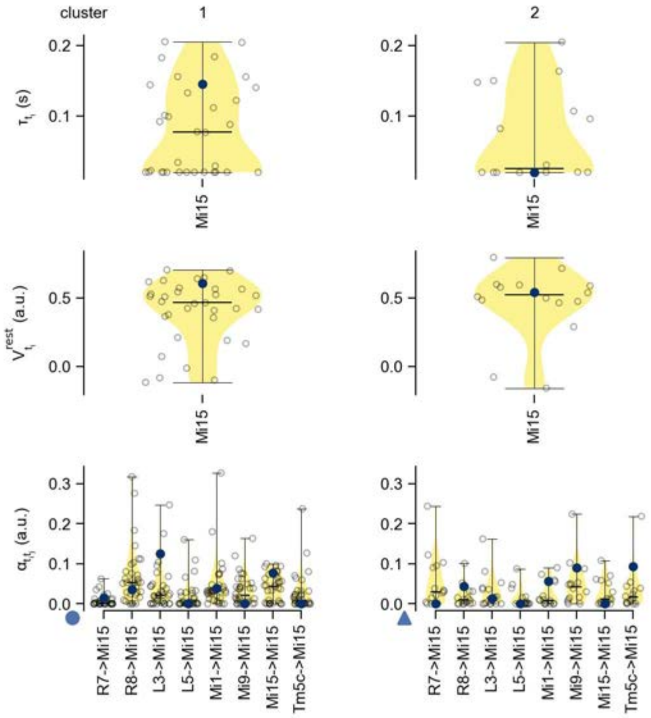

Mi15 - Figure 14: **Task-constrained parameters.** Each column shows the parameters inferred within the respective cluster. First row: learned time constants of the cell type. Second row: resting potentials of the cell type. Third row: scaling factors for the convolutional filters. The blue scatter represents the parameters from the task-optimal model within the cluster.

32 T1

← Cell types

Figures

1 Anatomical receptive fields. 221
2 Anatomical projective fields. 221
3 Clustering of the responses to naturalistic stimuli. 222
4 Responses to flashes. 222
5 Cluster-average responses to single-ommatidium flashes. 222
6 Peak responses to moving edges. 223
7 Peak responses to moving edges from task-optimal models. 223
8 Responses to moving edges from task-optimal models. 224
9 Peak responses to moving bars. 224
10 Peak responses to moving bars from task-optimal models. 225
11 Responses to moving bars from task-optimal models. 225
12 Spatio-temporal receptive field. 226
13 Maximally excitatory stimuli. 226
14 Task-constrained parameters. 227

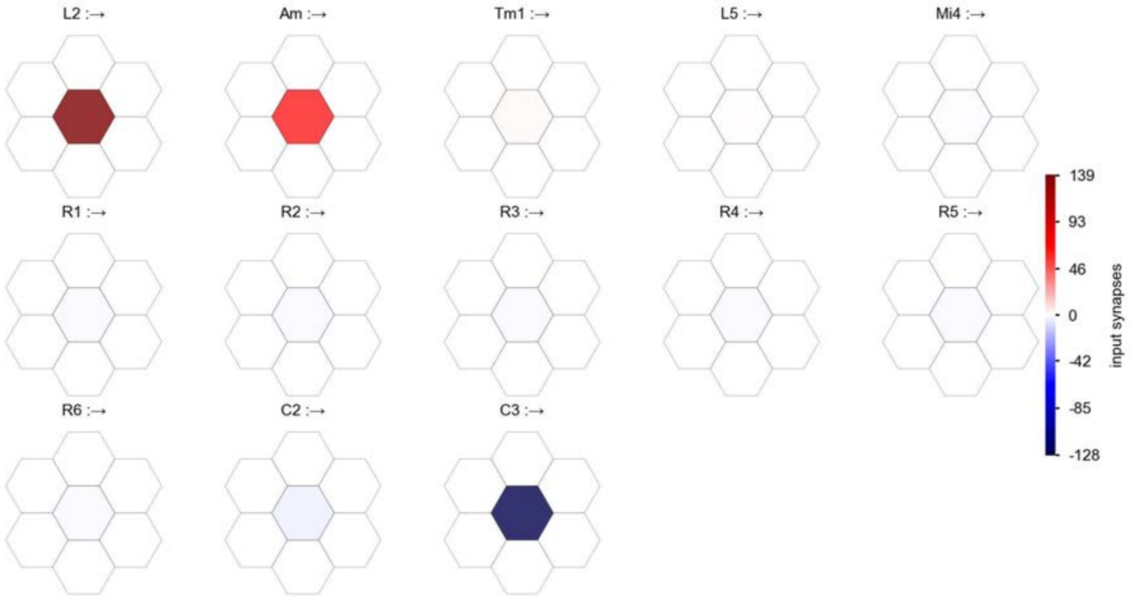

T1 - Figure 1: **Anatomical receptive fields.** Each colored hexagon is an input connection, with the connection strength characterized by the average number of synapses that we count from the EM reconstruction. Red indicates excitatory synapses, blue indicates inhibitory synapses from inferred signs. Filters in the order of their total number of synapses.

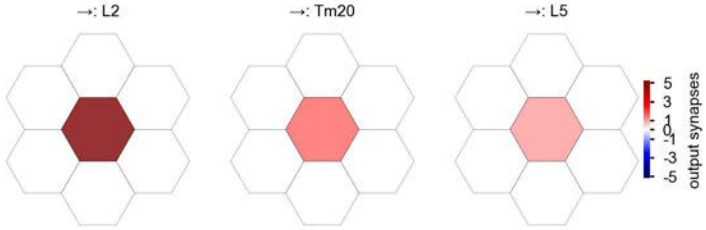

T1 - Figure 2: **Anatomical projective fields.** Each colored hexagon is an output connection, with the connection strength characterized by the average number of synapses that we count from the EM reconstruction. Red indicates excitatory synapses, blue indicates inhibitory synapses from inferred signs. Filters in the order of their total number of synapses.

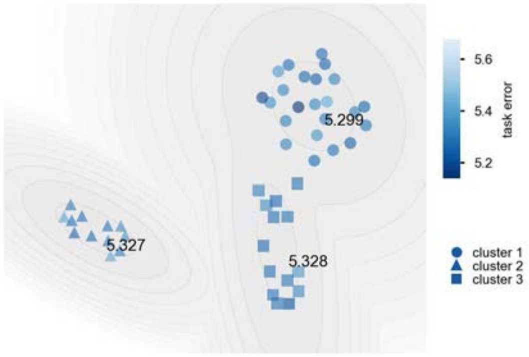

T1 - Figure 3: **Clustering of the responses to naturalistic stimuli.** Clustering of the 50 models based on the cell type responses to naturalistic scenes from the Sintel dataset. Scatterpoints represent individual models colored by their task error.

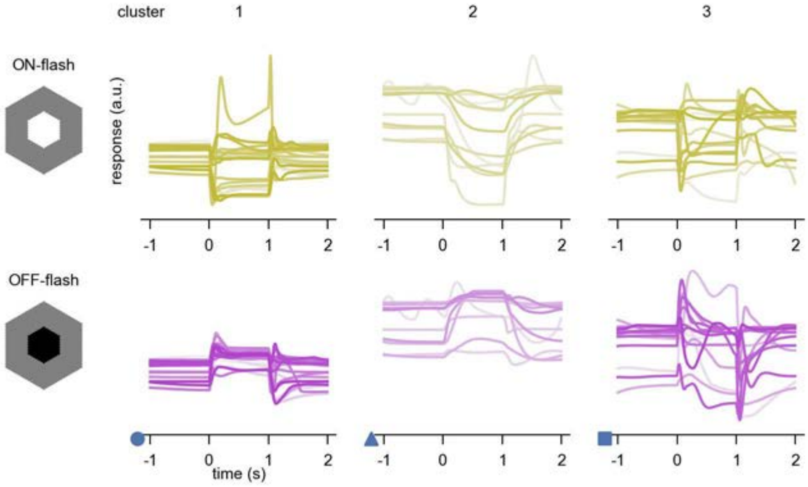

T1 - Figure 4: **Responses to flashes.** The top row shows responses to ON-flashes (yellow), the bottom row shows responses to OFF-flashes (magenta). The responses from the 50 different models that are separated into the different clusters (columns) overlay, with better task-performing models on top. Responses from better task-performing models are more saturated. The circular flashes (1s) cover 6 ommatidia in radius and are presented at time zero. Before and after, a grey-stimulus leads to a stationary state of the network.

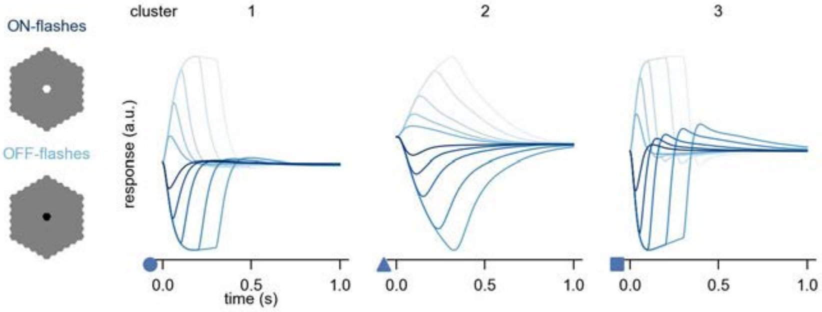

T1 - Figure 5: **Cluster-average responses to single-ommatidium flashes.** Responses to single-ommatidium ON-flashes (dark blue shades) and single-ommatidium OFF-flashes (light blue shades) of 20ms, 50ms, 100ms, 200ms, 300ms duration. The flashes occur at second zero.

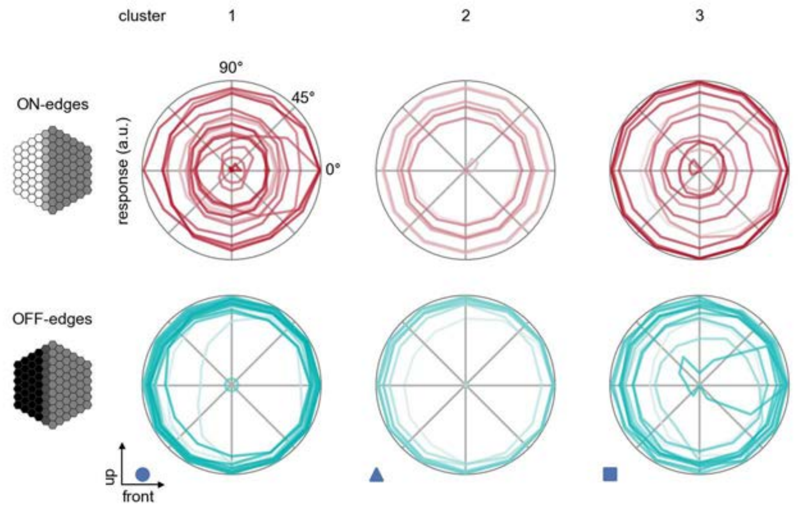

T1 - Figure 6: **Peak responses to moving edges.** The top row shows peak responses to moving ON-edges (red), the bottom row shows peak responses to moving OFF-edges (turqoise). The peak responses are averaged over edge-speeds. Edge-stimuli move in different directions from 0 to 360 degrees. The responses from the different models in the different clusters (columns) overlay. Responses from better task-performing models are more saturated.

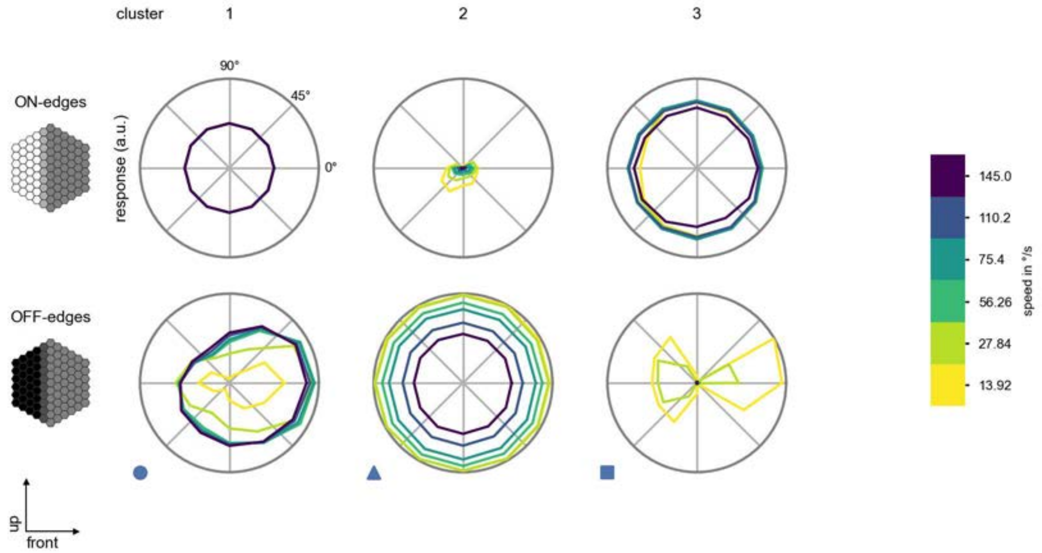

T1 - Figure 7: **Peak responses to moving edges from task-optimal models.** The top row shows peak responses to moving ON-edges, the bottom row shows peak responses to moving OFF-edges of varying speeds from 13.92°/s to 145°/s (yellow to dark blue). The edge-stimuli move in different directions from 0 to 360 degrees and at different speeds. Responses from the task-optimal model in the respective cluster.

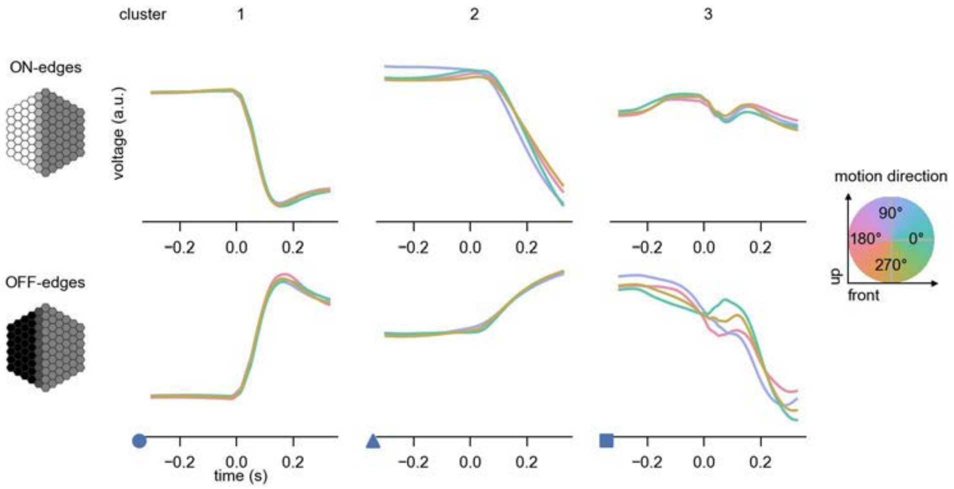

T1 - Figure 8: **Responses to moving edges from task-optimal models.** Responses to moving ON-edges (top row) and to moving OFF-edges (bottom row). Edges move in different directions from 0 to 360 degrees and at different speeds. Responses are from the task-optimal model in the respective cluster. Edges moving at 75.4°/s in all cardinal directions (green 0°, blue 90°, red 180°, yellow 270°) from -22.5 to 22.5° visual angle.

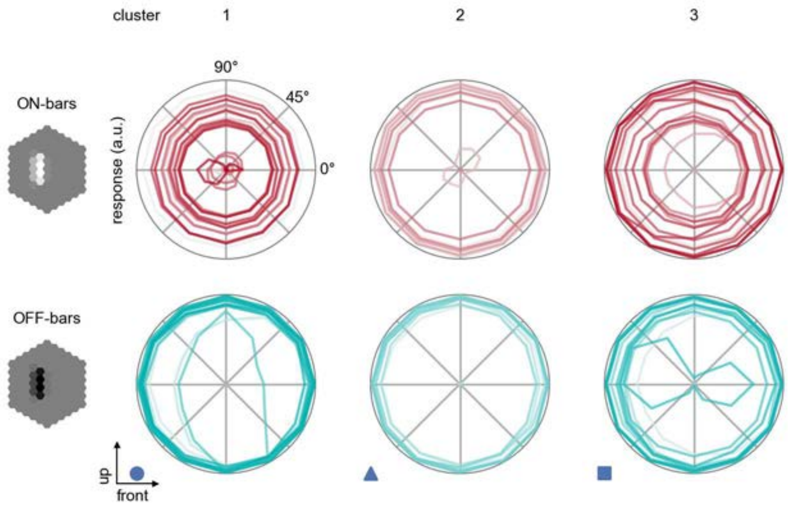

T1 - Figure 9: **Peak responses to moving bars.** The top row shows peak responses to moving ON-bars (red), the bottom row shows peak responses to moving OFF-bars (turqoise). The peak responses are averaged over bar-speeds. Bar-stimuli move in different directions from 0 to 360 degrees. The responses from the different models in the different clusters (columns) overlay. Responses from better task-performing models are more saturated.

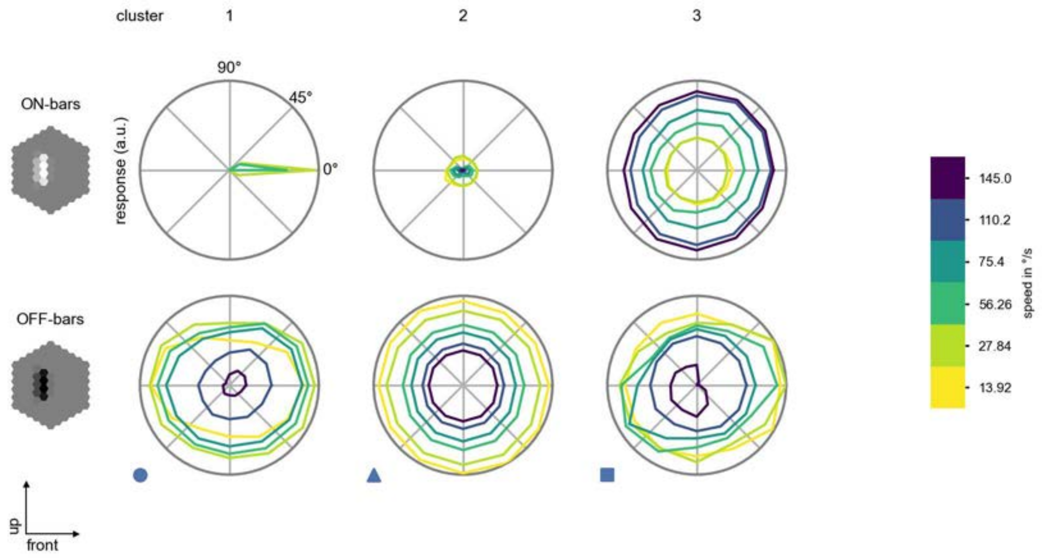

T1 - Figure 10: **Peak responses to moving bars from task-optimal models.** The top row shows peak responses to moving ON-bars, the bottom row shows peak responses to moving OFF-bars of varying speeds from 13.92°/s to 145°/s (yellow to dark blue). The bar-stimuli move in different directions from 0 to 360 degrees and at different speeds. Responses from the task-optimal model in the respective cluster.

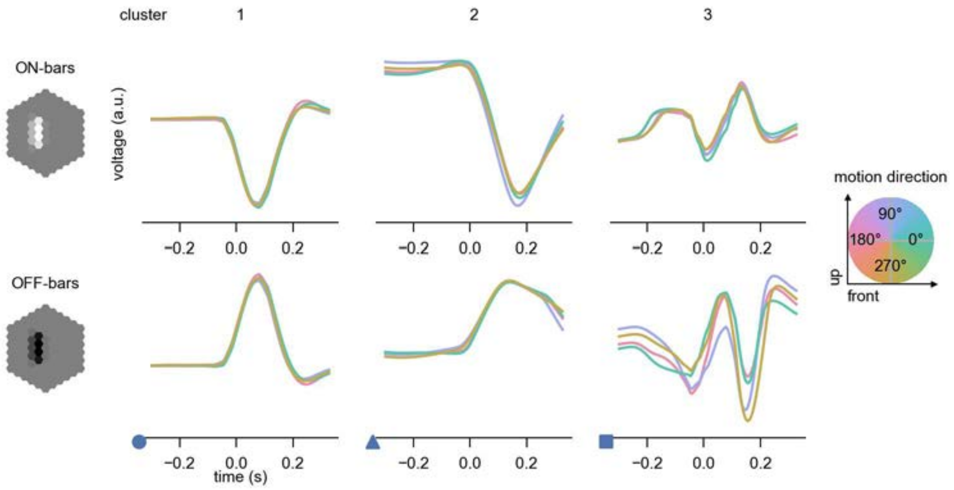

T1 - Figure 11: **Responses to moving bars from task-optimal models.** Responses to moving ON-bars (top row) and to moving OFF-bars (bottom row). Bars move in different directions from 0 to 360 degrees and at different speeds. Responses are from the task-optimal model in the respective cluster. Bars moving at 75.4°/s in all cardinal directions (green 0°, blue 90°, red 180°, yellow 270°) from -22.5 to 22.5° visual angle.

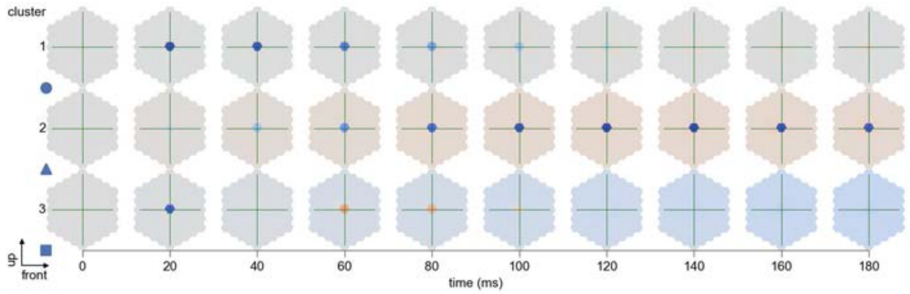

T1 - Figure 12: **Spatio-temporal receptive field.** Responses of the central cell to ON-impulses (5 ms) at single-ommatidium flash locations. The flash occurs at second zero. Responses from the task-optimal model of the respective cluster (rows). Red indicates depolarization, blue indicates hyperpolarization.

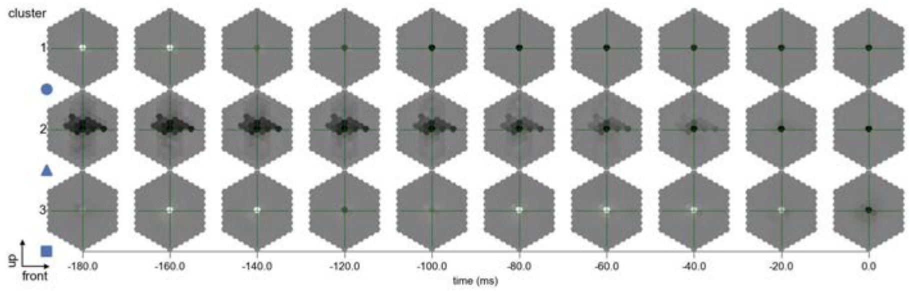

T1 - Figure 13: **Maximally excitatory stimuli.** Each row presents the regularized naturalistic-stimulus from the Sintel dataset that maximizes the cell type's central column response at second zero in the task-optimal model of the respective cluster (rows).

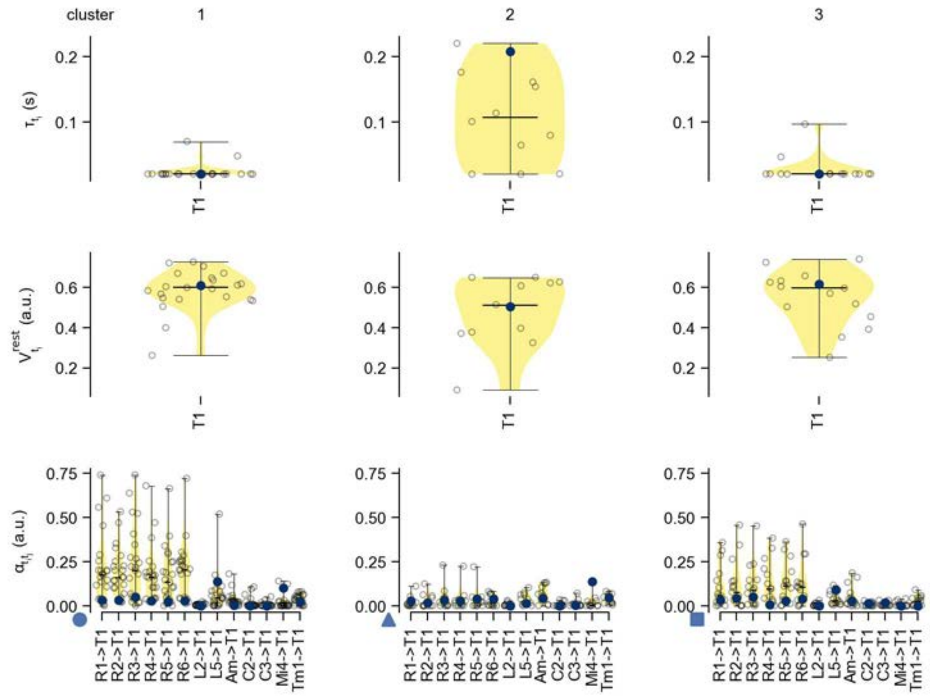

T1 - Figure 14: **Task-constrained parameters.** Each column shows the parameters inferred within the respective cluster. First row: learned time constants of the cell type. Second row: resting potentials of the cell type. Third row: scaling factors for the convolutional filters. The blue scatter represents the parameters from the task-optimal model within the cluster.

33 T2

← Cell types

Figures

1 Anatomical receptive fields. . . . 228
2 Anatomical projective fields. . . . 229
3 Clustering of the responses to naturalistic stimuli. . . . 229
4 Responses to flashes. . . . 230
5 Cluster-average responses to single-ommatidium flashes. . . . 230
6 Peak responses to moving edges. . . . 231
7 Peak responses to moving edges from task-optimal models. . . . 231
8 Responses to moving edges from task-optimal models. . . . 232
9 Peak responses to moving bars. . . . 232
10 Peak responses to moving bars from task-optimal models. . . . 233
11 Responses to moving bars from task-optimal models. . . . 233
12 Spatio-temporal receptive field. . . . 234
13 Maximally excitatory stimuli. . . . 234
14 Task-constrained parameters. . . . 235

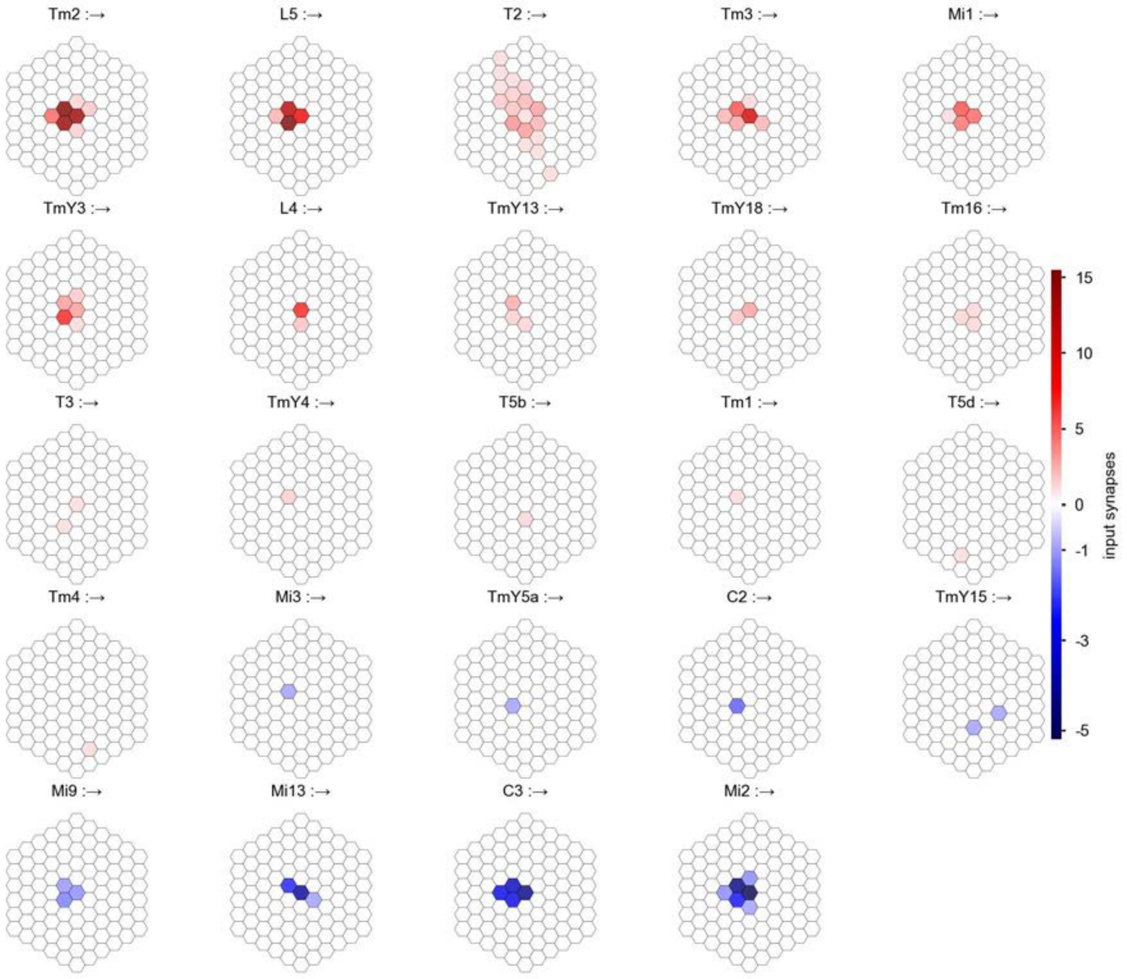

T2 - Figure 1: **Anatomical receptive fields.** Each colored hexagon is an input connection, with the connection strength characterized by the average number of synapses that we count from the EM reconstruction. Red indicates excitatory synapses, blue indicates inhibitory synapses from inferred signs. Filters in the order of their total number of synapses.

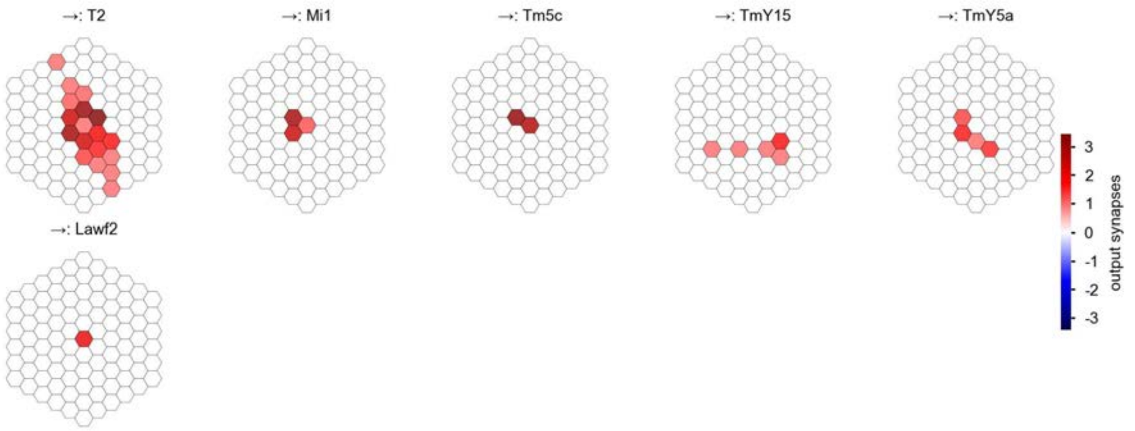

T2 - Figure 2: **Anatomical projective fields.** Each colored hexagon is an output connection, with the connection strength characterized by the average number of synapses that we count from the EM reconstruction. Red indicates excitatory synapses, blue indicates inhibitory synapses from inferred signs. Filters in the order of their total number of synapses.

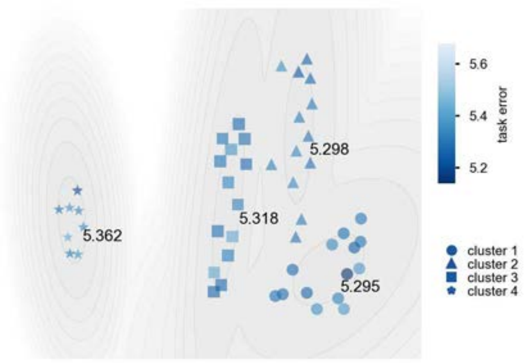

T2 - Figure 3: **Clustering of the responses to naturalistic stimuli.** Clustering of the 50 models based on the cell type responses to naturalistic scenes from the Sintel dataset. Scatterpoints represent individual models colored by their task error.

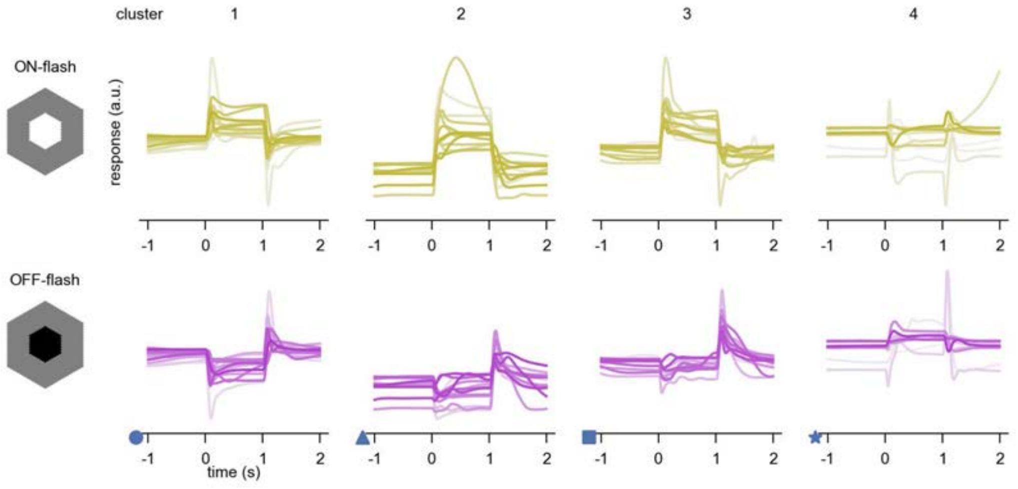

T2 - Figure 4: **Responses to flashes.** The top row shows responses to ON-flashes (yellow), the bottom row shows responses to OFF-flashes (magenta). The responses from the 50 different models that are separated into the different clusters (columns) overlay, with better task-performing models on top. Responses from better task-performing models are more saturated. The circular flashes (1s) cover 6 ommatidia in radius and are presented at time zero. Before and after, a grey-stimulus leads to a stationary state of the network.

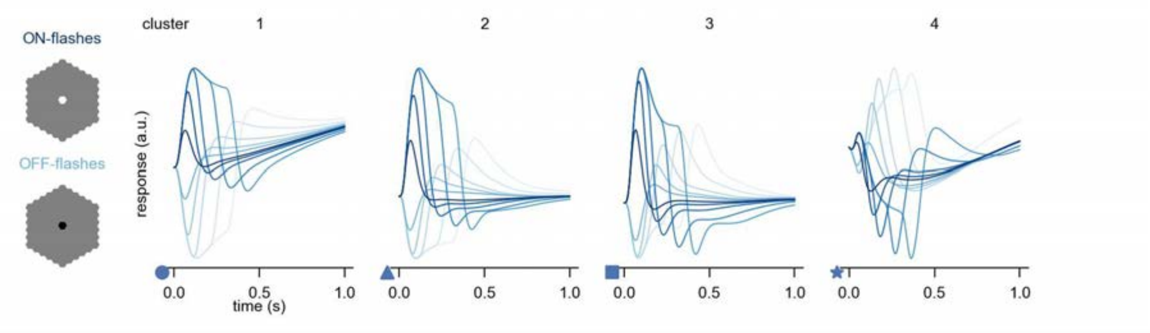

T2 - Figure 5: **Cluster-average responses to single-ommatidium flashes.** Responses to single-ommatidium ON-flashes (dark blue shades) and single-ommatidium OFF-flashes (light blue shades) of 20ms, 50ms, 100ms, 200ms, 300ms duration. The flashes occur at second zero.

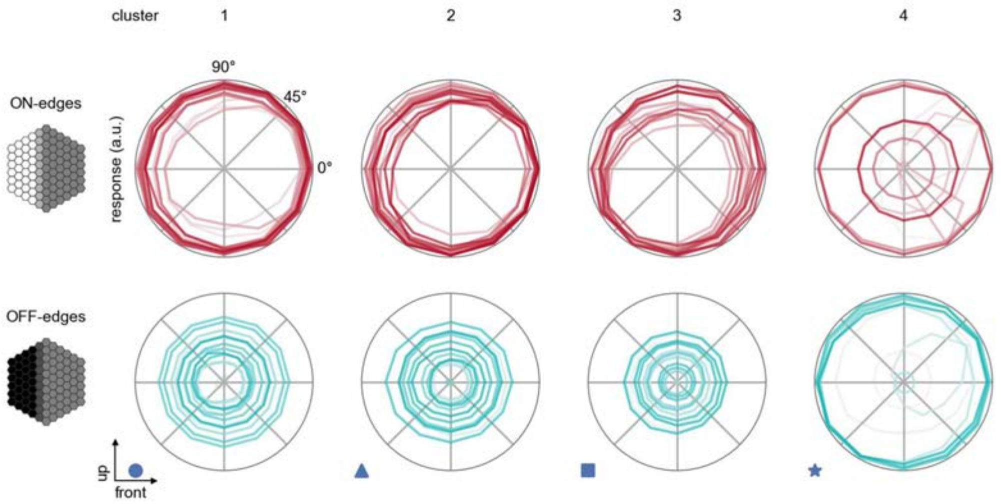

T2 - Figure 6: **Peak responses to moving edges.** The top row shows peak responses to moving ON-edges (red), the bottom row shows peak responses to moving OFF-edges (turqoise). The peak responses are averaged over edge-speeds. Edge-stimuli move in different directions from 0 to 360 degrees. The responses from the different models in the different clusters (columns) overlay. Responses from better task-performing models are more saturated.

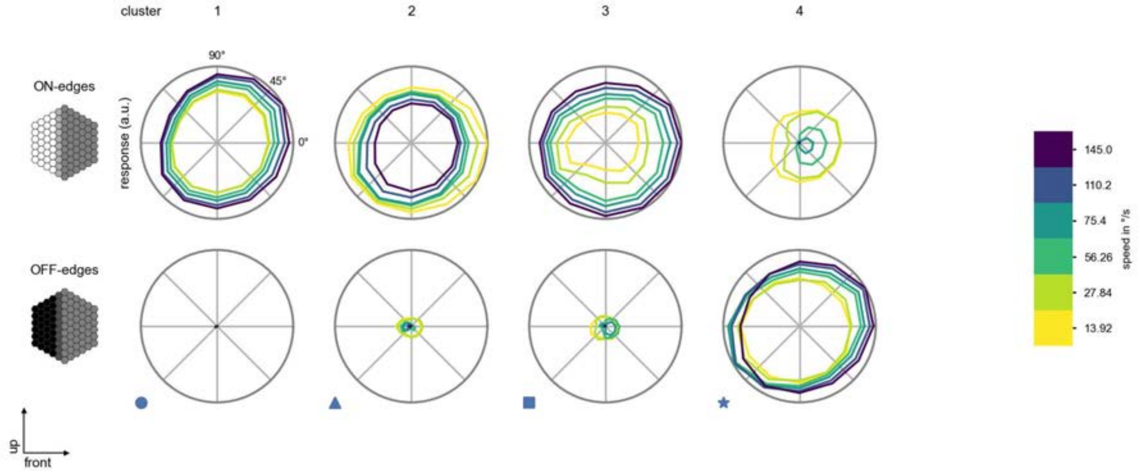

T2 - Figure 7: **Peak responses to moving edges from task-optimal models.** The top row shows peak responses to moving ON-edges, the bottom row shows peak responses to moving OFF-edges of varying speeds from 13.92°/s to 145°/s (yellow to dark blue). The edge-stimuli move in different directions from 0 to 360 degrees and at different speeds. Responses from the task-optimal model in the respective cluster.

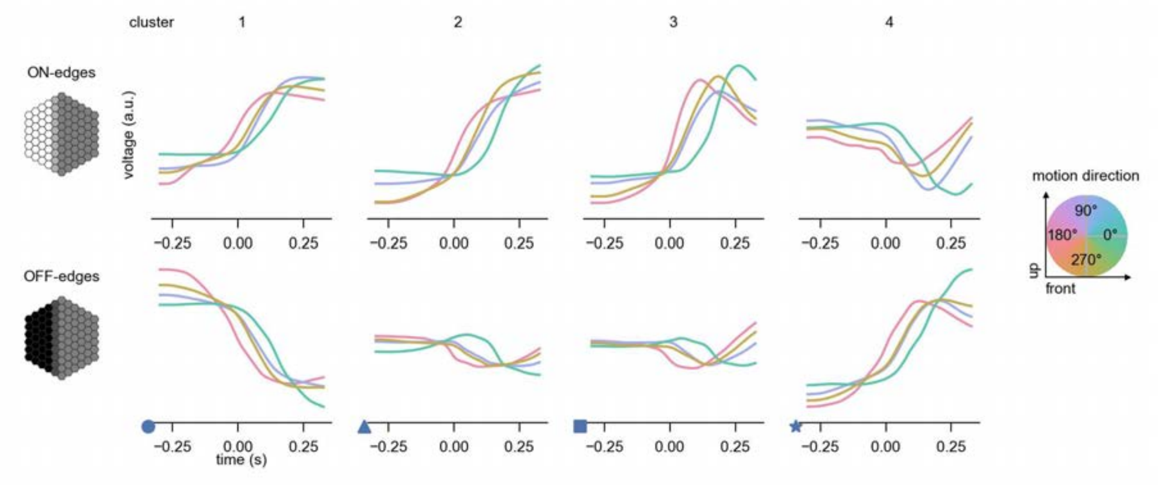

T2 - Figure 8: **Responses to moving edges from task-optimal models.** Responses to moving ON-edges (top row) and to moving OFF-edges (bottom row). Edges move in different directions from 0 to 360 degrees and at different speeds. Responses are from the task-optimal model in the respective cluster. Edges moving at 75.4°/s in all cardinal directions (green 0°, blue 90°, red 180°, yellow 270°) from -22.5 to 22.5° visual angle.

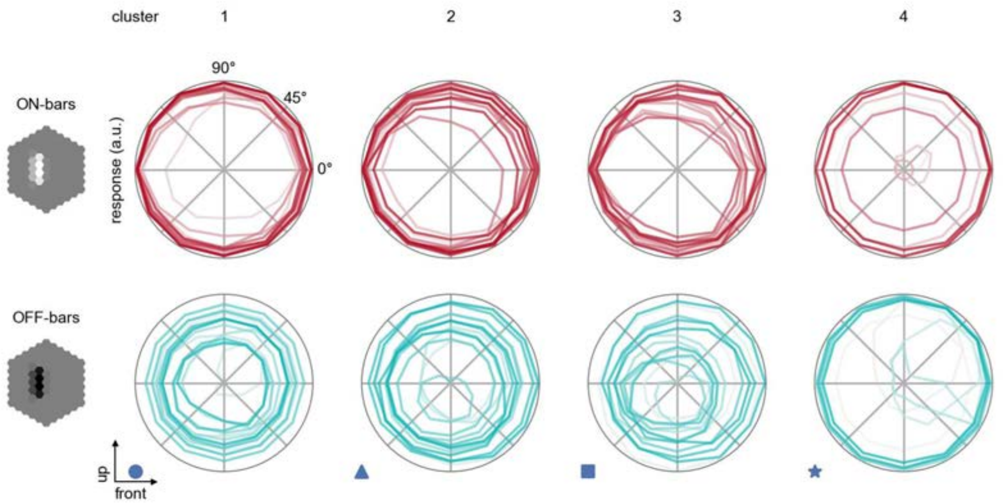

T2 - Figure 9: **Peak responses to moving bars.** The top row shows peak responses to moving ON-bars (red), the bottom row shows peak responses to moving OFF-bars (turqoise). The peak responses are averaged over bar-speeds. Bar-stimuli move in different directions from 0 to 360 degrees. The responses from the different models in the different clusters (columns) overlay. Responses from better task-performing models are more saturated.

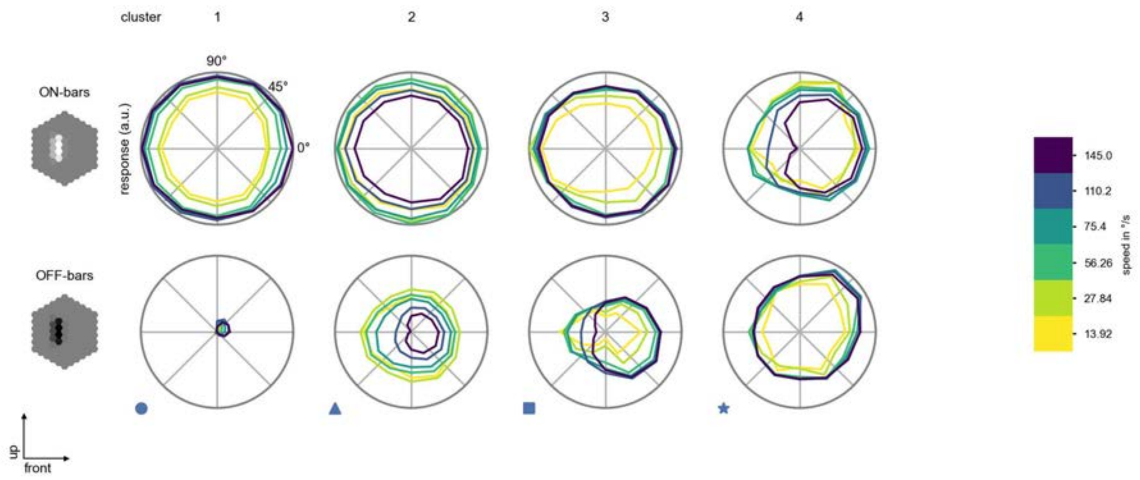

T2 - Figure 10: **Peak responses to moving bars from task-optimal models.** The top row shows peak responses to moving ON-bars, the bottom row shows peak responses to moving OFF-bars of varying speeds from 13.92°/s to 145°/s (yellow to dark blue). The bar-stimuli move in different directions from 0 to 360 degrees and at different speeds. Responses from the task-optimal model in the respective cluster.

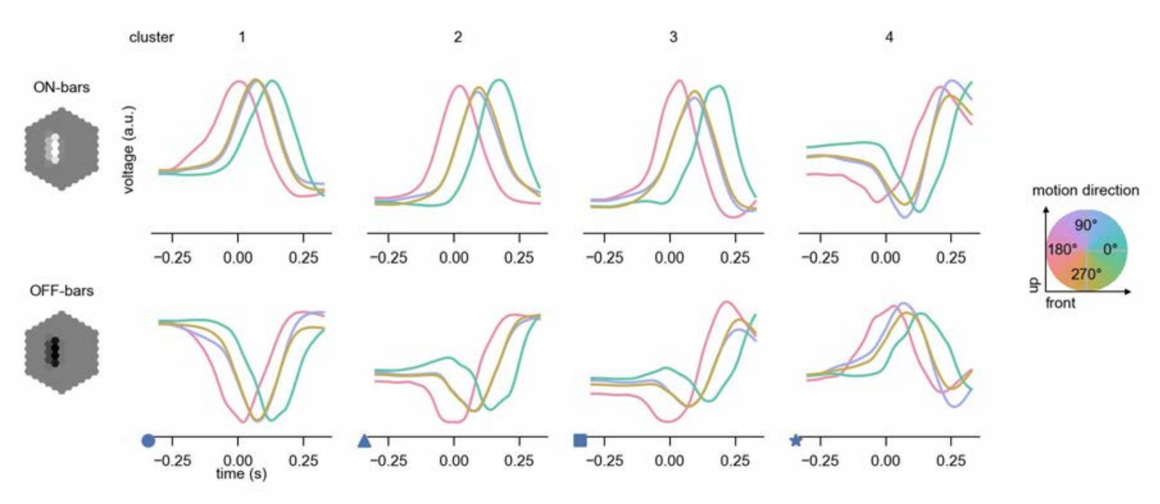

T2 - Figure 11: **Responses to moving bars from task-optimal models.** Responses to moving ON-bars (top row) and to moving OFF-bars (bottom row). Bars move in different directions from 0 to 360 degrees and at different speeds. Responses are from the task-optimal model in the respective cluster. Bars moving at 75.4°/s in all cardinal directions (green 0°, blue 90°, red 180°, yellow 270°) from -22.5 to 22.5° visual angle.

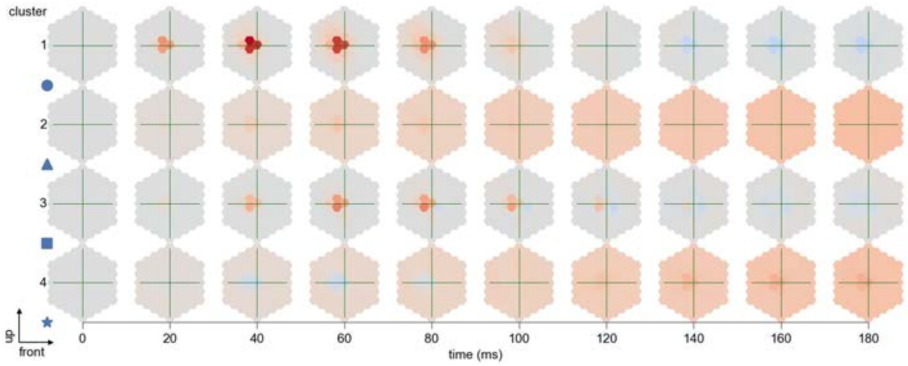

T2 - Figure 12: **Spatio-temporal receptive field.** Responses of the central cell to ON-impulses (5 ms) at single-ommatidium flash locations. The flash occurs at second zero. Responses from the task-optimal model of the respective cluster (rows). Red indicates depolarization, blue indicates hyperpolarization.

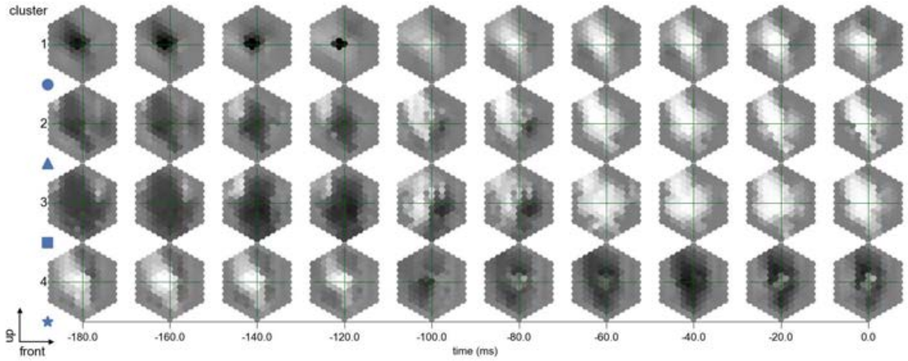

T2 - Figure 13: **Maximally excitatory stimuli.** Each row presents the regularized naturalistic-stimulus from the Sintel dataset that maximizes the cell type's central column response at second zero in the task-optimal model of the respective cluster (rows).

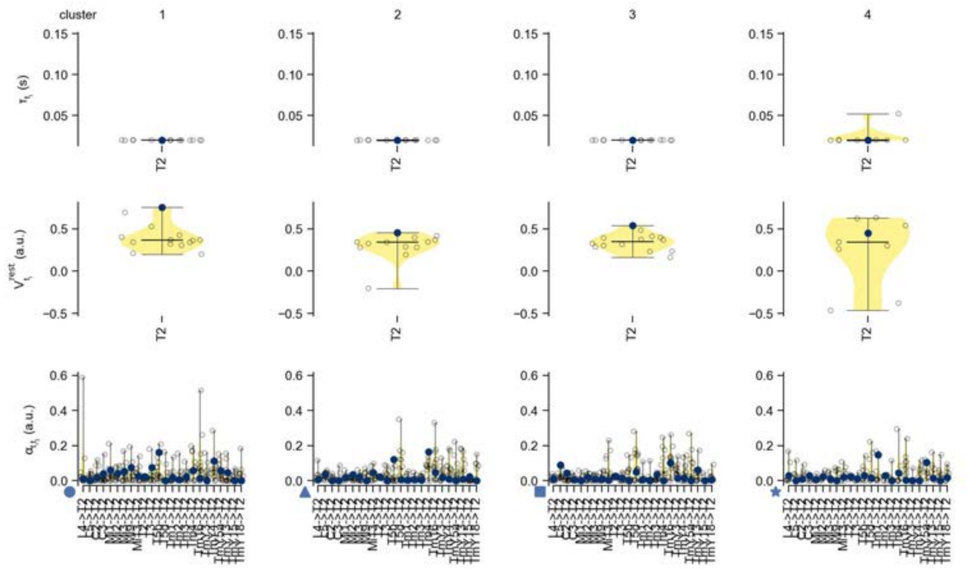

T2 - Figure 14: **Task-constrained parameters.** Each column shows the parameters inferred within the respective cluster. First row: learned time constants of the cell type. Second row: resting potentials of the cell type. Third row: scaling factors for the convolutional filters. The blue scatter represents the parameters from the task-optimal model within the cluster.

34 T2a

← Cell types

Figures

1 Anatomical receptive fields. . . . 236
2 Anatomical projective fields. . . . 237
3 Clustering of the responses to naturalistic stimuli. . . . 237
4 Responses to flashes. . . . 238
5 Cluster-average responses to single-ommatidium flashes. . . . 238
6 Peak responses to moving edges. . . . 239
7 Peak responses to moving edges from task-optimal models. . . . 239
8 Responses to moving edges from task-optimal models. . . . 240
9 Peak responses to moving bars. . . . 240
10 Peak responses to moving bars from task-optimal models. . . . 241
11 Responses to moving bars from task-optimal models. . . . 241
12 Spatio-temporal receptive field. . . . 242
13 Maximally excitatory stimuli. . . . 242
14 Task-constrained parameters. . . . 243

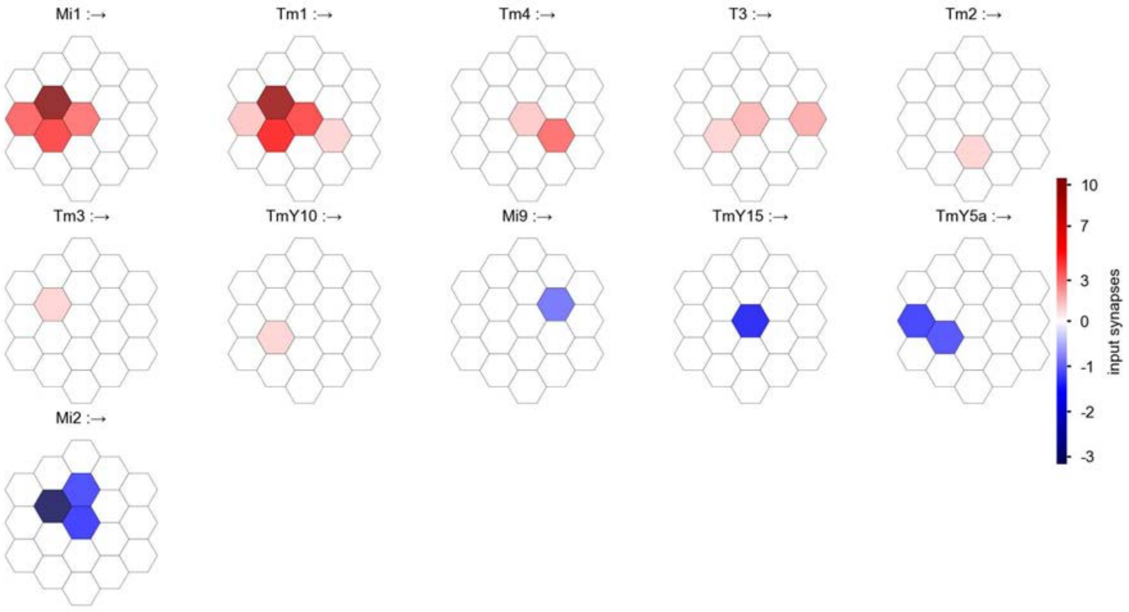

T2a - Figure 1: **Anatomical receptive fields.** Each colored hexagon is an input connection, with the connection strength characterized by the average number of synapses that we count from the EM reconstruction. Red indicates excitatory synapses, blue indicates inhibitory synapses from inferred signs. Filters in the order of their total number of synapses.

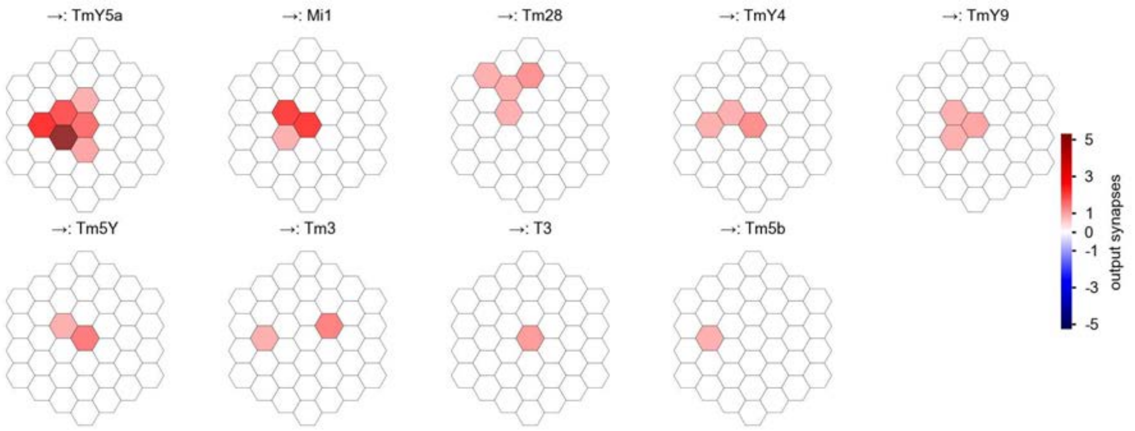

T2a - Figure 2: **Anatomical projective fields.** Each colored hexagon is an output connection, with the connection strength characterized by the average number of synapses that we count from the EM reconstruction. Red indicates excitatory synapses, blue indicates inhibitory synapses from inferred signs. Filters in the order of their total number of synapses.

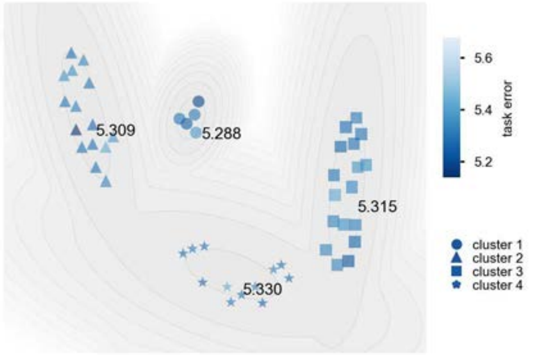

T2a - Figure 3: **Clustering of the responses to naturalistic stimuli.** Clustering of the 50 models based on the cell type responses to naturalistic scenes from the Sintel dataset. Scatterpoints represent individual models colored by their task error.

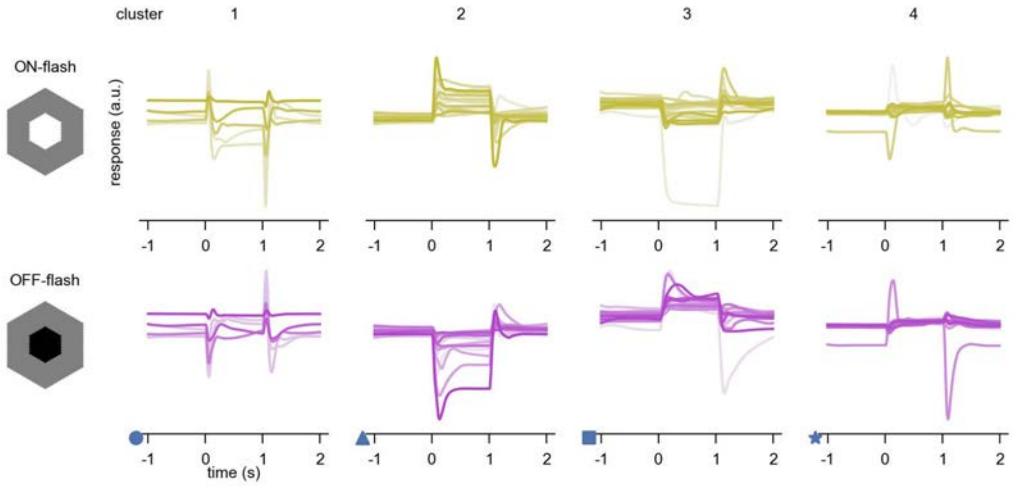

T2a - Figure 4: **Responses to flashes.** The top row shows responses to ON-flashes (yellow), the bottom row shows responses to OFF-flashes (magenta). The responses from the 50 different models that are separated into the different clusters (columns) overlay, with better task-performing models on top. Responses from better task-performing models are more saturated. The circular flashes (1s) cover 6 ommatidia in radius and are presented at time zero. Before and after, a grey-stimulus leads to a stationary state of the network.

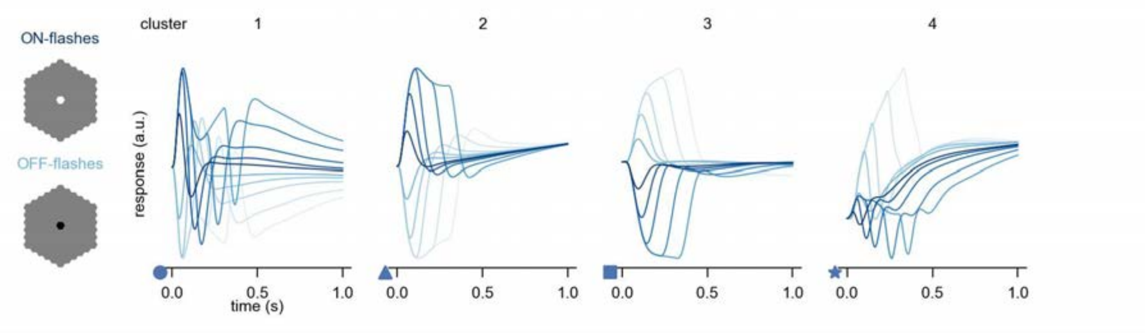

T2a - Figure 5: **Cluster-average responses to single-ommatidium flashes.** Responses to single-ommatidium ON-flashes (dark blue shades) and single-ommatidium OFF-flashes (light blue shades) of 20ms, 50ms, 100ms, 200ms, 300ms duration. The flashes occur at second zero.

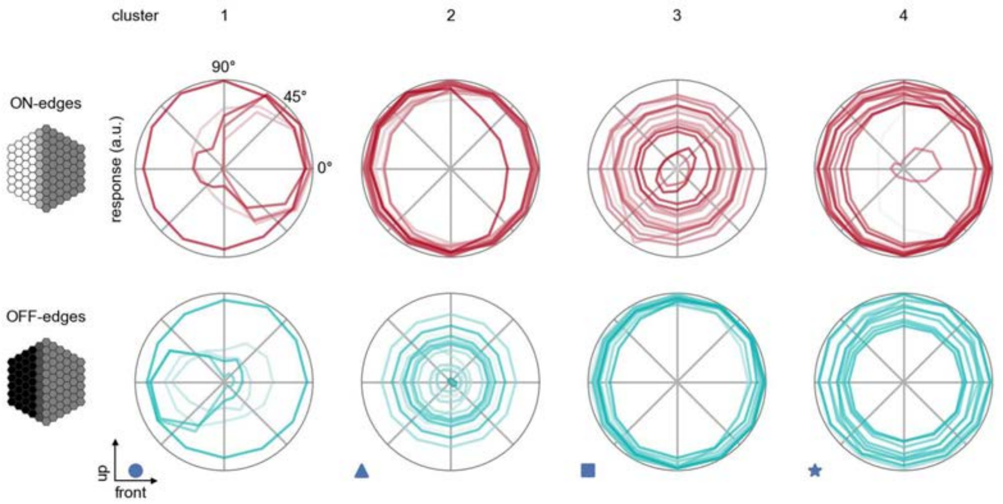

T2a - Figure 6: **Peak responses to moving edges.** The top row shows peak responses to moving ON-edges (red), the bottom row shows peak responses to moving OFF-edges (turqoise). The peak responses are averaged over edge-speeds. Edge-stimuli move in different directions from 0 to 360 degrees. The responses from the different models in the different clusters (columns) overlay. Responses from better task-performing models are more saturated.

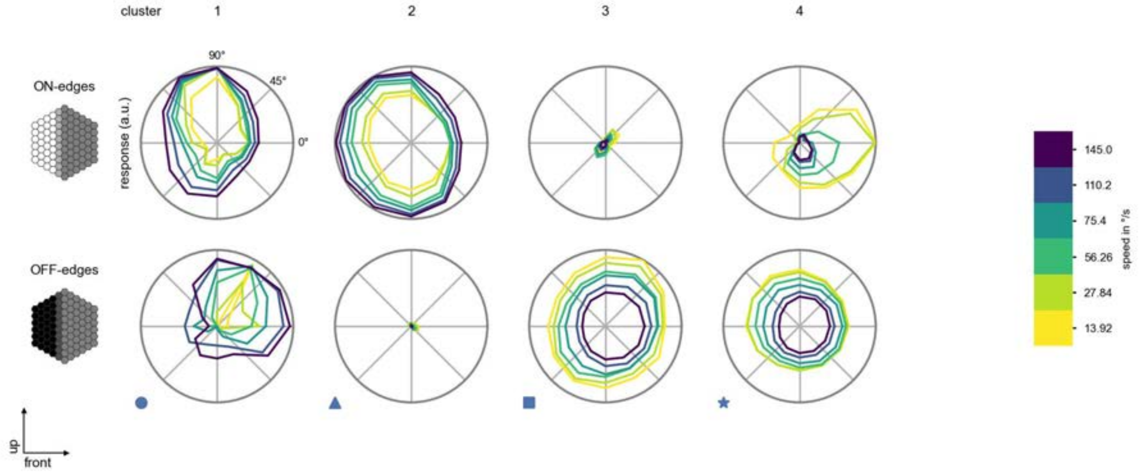

T2a - Figure 7: **Peak responses to moving edges from task-optimal models.** The top row shows peak responses to moving ON-edges, the bottom row shows peak responses to moving OFF-edges of varying speeds from 13.92°/s to 145°/s (yellow to dark blue). The edge-stimuli move in different directions from 0 to 360 degrees and at different speeds. Responses from the task-optimal model in the respective cluster.

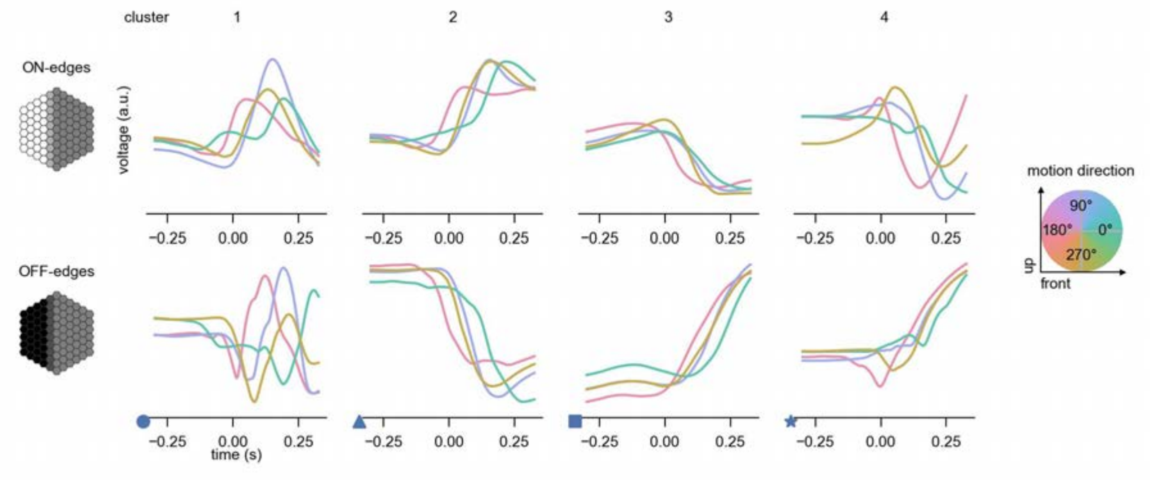

T2a - Figure 8: **Responses to moving edges from task-optimal models.** Responses to moving ON-edges (top row) and to moving OFF-edges (bottom row). Edges move in different directions from 0 to 360 degrees and at different speeds. Responses are from the task-optimal model in the respective cluster. Edges moving at 75.4°/s in all cardinal directions (green 0°, blue 90°, red 180°, yellow 270°) from -22.5 to 22.5° visual angle.

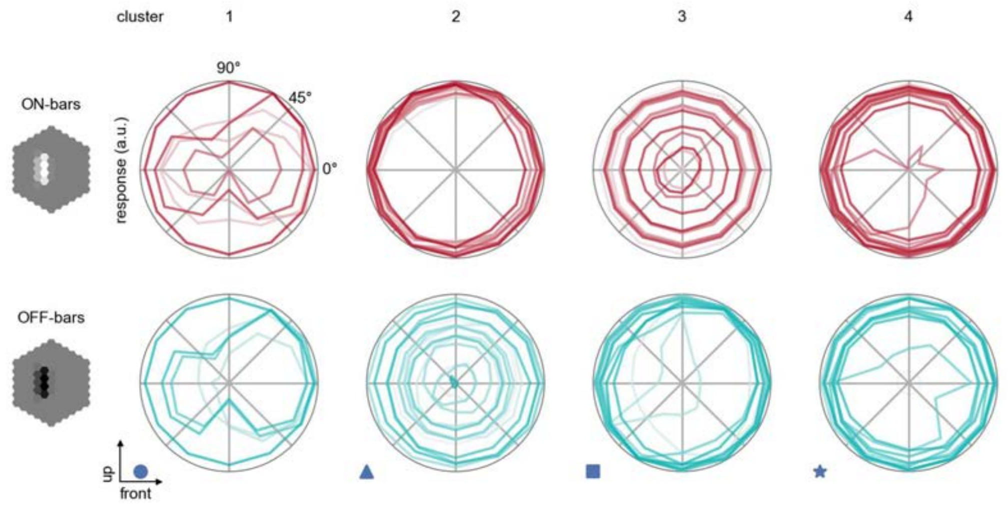

T2a - Figure 9: **Peak responses to moving bars.** The top row shows peak responses to moving ON-bars (red), the bottom row shows peak responses to moving OFF-bars (turqoise). The peak responses are averaged over bar-speeds. Bar-stimuli move in different directions from 0 to 360 degrees. The responses from the different models in the different clusters (columns) overlay. Responses from better task-performing models are more saturated.

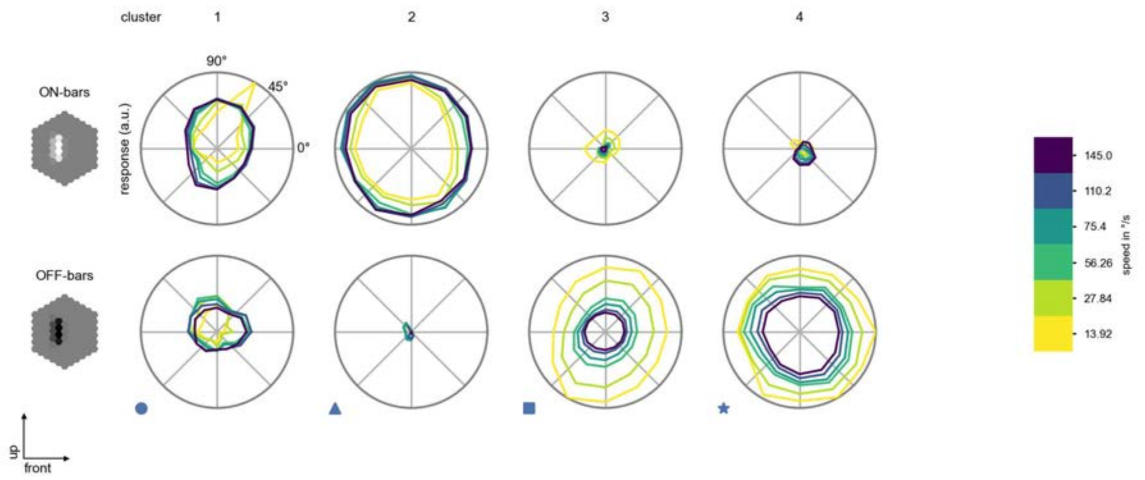

T2a - Figure 10: **Peak responses to moving bars from task-optimal models.** The top row shows peak responses to moving ON-bars, the bottom row shows peak responses to moving OFF-bars of varying speeds from 13.92°/s to 145°/s (yellow to dark blue). The bar-stimuli move in different directions from 0 to 360 degrees and at different speeds. Responses from the task-optimal model in the respective cluster.

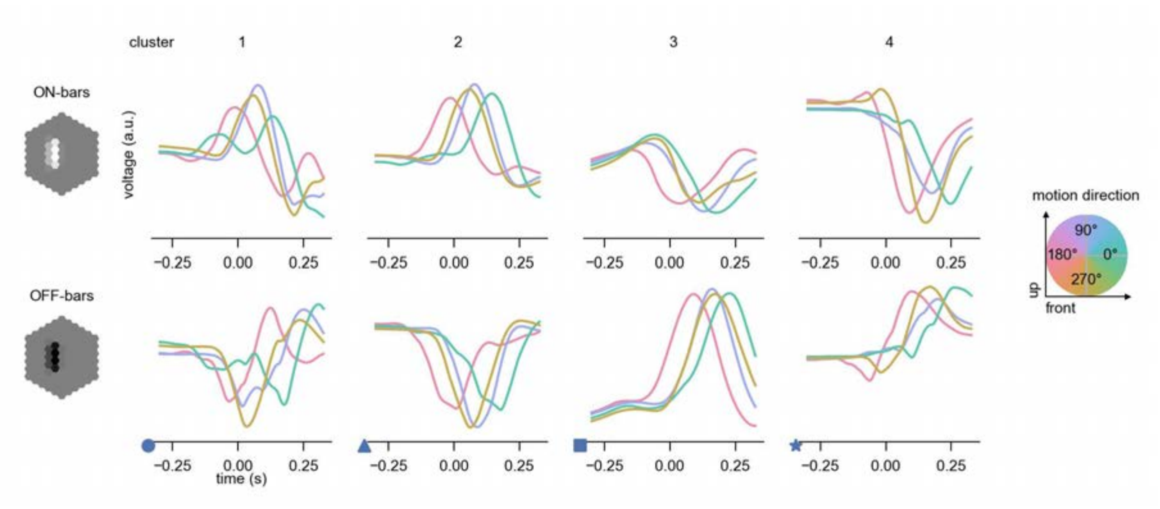

T2a - Figure 11: **Responses to moving bars from task-optimal models.** Responses to moving ON-bars (top row) and to moving OFF-bars (bottom row). Bars move in different directions from 0 to 360 degrees and at different speeds. Responses are from the task-optimal model in the respective cluster. Bars moving at 75.4°/s in all cardinal directions (green 0°, blue 90°, red 180°, yellow 270°) from -22.5 to 22.5° visual angle.

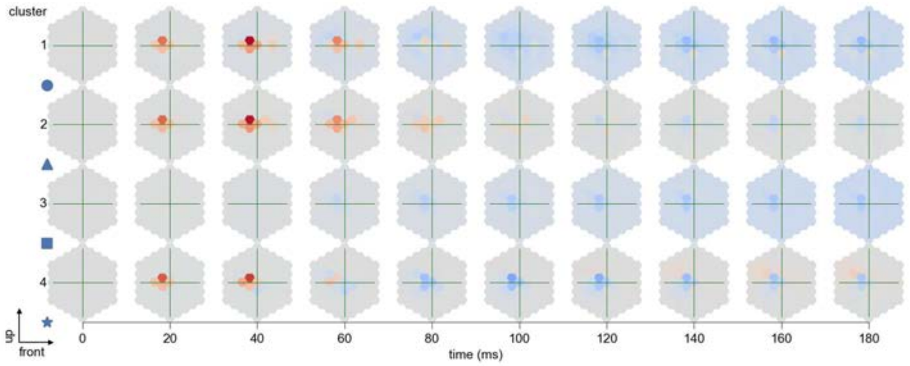

T2a - Figure 12: **Spatio-temporal receptive field.** Responses of the central cell to ON-impulses (5 ms) at single-ommatidium flash locations. The flash occurs at second zero. Responses from the task-optimal model of the respective cluster (rows). Red indicates depolarization, blue indicates hyperpolarization.

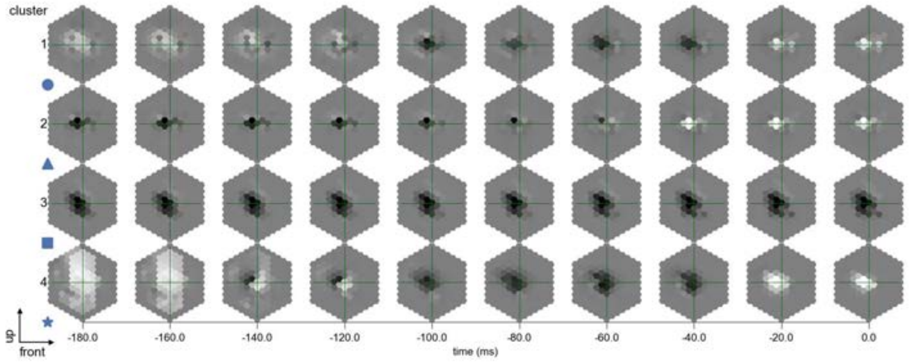

T2a - Figure 13: **Maximally excitatory stimuli.** Each row presents the regularized naturalistic-stimulus from the Sintel dataset that maximizes the cell type's central column response at second zero in the task-optimal model of the respective cluster (rows).

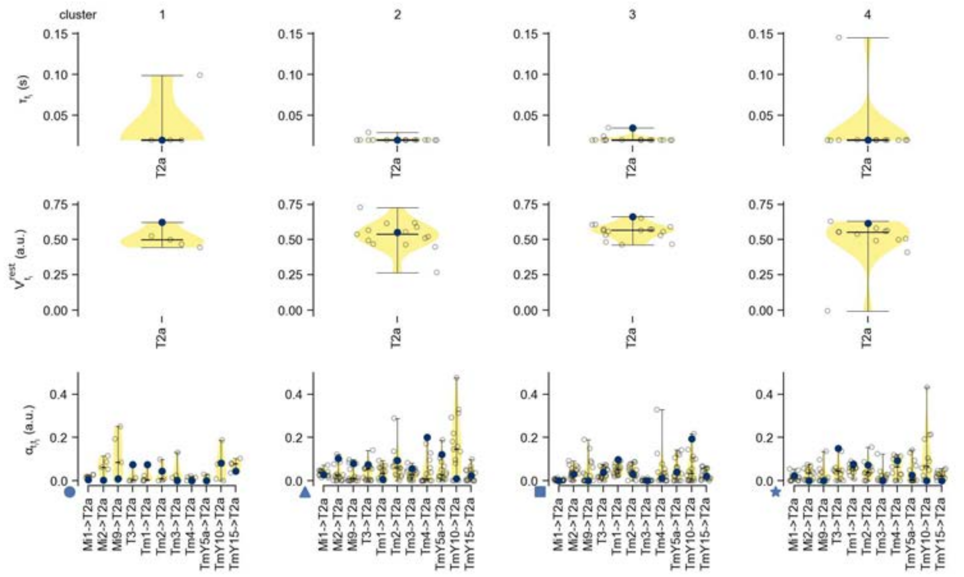

T2a - Figure 14: **Task-constrained parameters.** Each column shows the parameters inferred within the respective cluster. First row: learned time constants of the cell type. Second row: resting potentials of the cell type. Third row: scaling factors for the convolutional filters. The blue scatter represents the parameters from the task-optimal model within the cluster.

35 T3

← Cell types

Figures

1 Anatomical receptive fields. . . . 244
2 Anatomical projective fields. . . . 244
3 Clustering of the responses to naturalistic stimuli. . . . 245
4 Responses to flashes. . . . 245
5 Cluster-average responses to single-ommatidium flashes. . . . 245
6 Peak responses to moving edges. . . . 246
7 Peak responses to moving edges from task-optimal models. . . . 246
8 Responses to moving edges from task-optimal models. . . . 247
9 Peak responses to moving bars. . . . 247
10 Peak responses to moving bars from task-optimal models. . . . 248
11 Responses to moving bars from task-optimal models. . . . 248
12 Spatio-temporal receptive field. . . . 248
13 Maximally excitatory stimuli. . . . 249
14 Task-constrained parameters. . . . 249

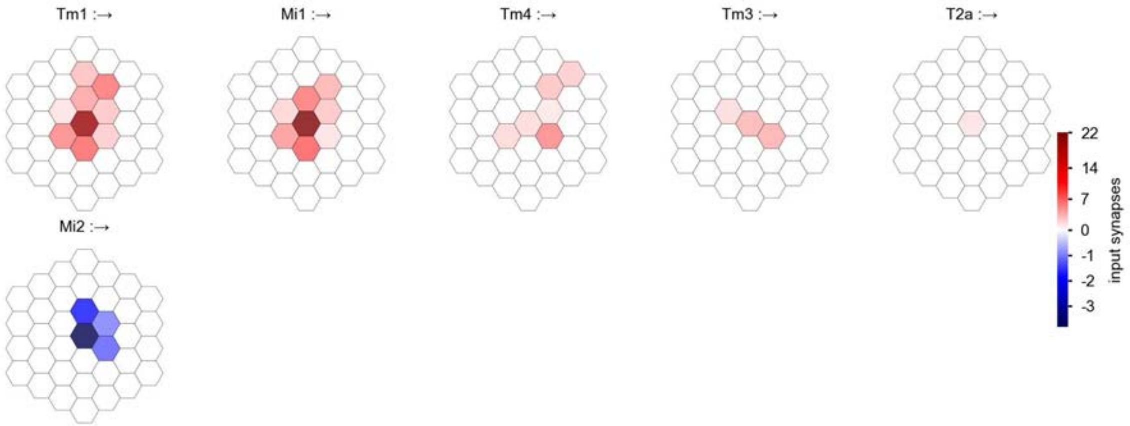

T3 - Figure 1: **Anatomical receptive fields.** Each colored hexagon is an input connection, with the connection strength characterized by the average number of synapses that we count from the EM reconstruction. Red indicates excitatory synapses, blue indicates inhibitory synapses from inferred signs. Filters in the order of their total number of synapses.

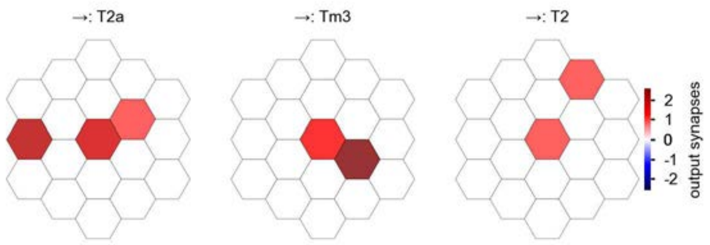

T3 - Figure 2: **Anatomical projective fields.** Each colored hexagon is an output connection, with the connection strength characterized by the average number of synapses that we count from the EM reconstruction. Red indicates excitatory synapses, blue indicates inhibitory synapses from inferred signs. Filters in the order of their total number of synapses.

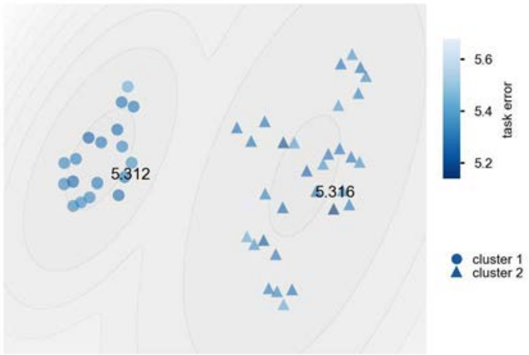

T3 - Figure 3: **Clustering of the responses to naturalistic stimuli.** Clustering of the 50 models based on the cell type responses to naturalistic scenes from the Sintel dataset. Scatterpoints represent individual models colored by their task error.

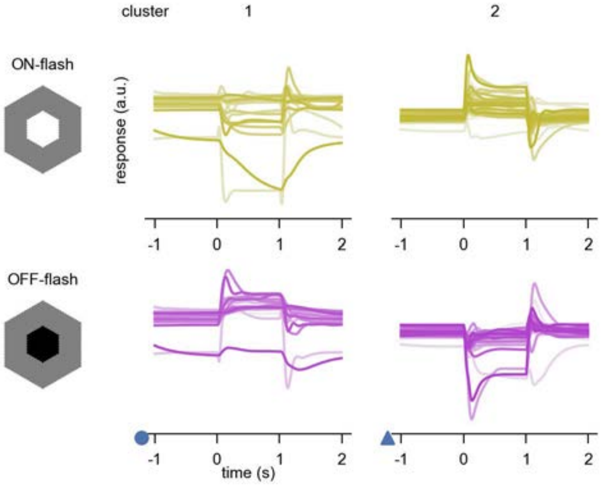

T3 - Figure 4: **Responses to flashes.** The top row shows responses to ON-flashes (yellow), the bottom row shows responses to OFF-flashes (magenta). The responses from the 50 different models that are separated into the different clusters (columns) overlay, with better task-performing models on top. Responses from better task-performing models are more saturated. The circular flashes (1s) cover 6 ommatidia in radius and are presented at time zero. Before and after, a grey-stimulus leads to a stationary state of the network.

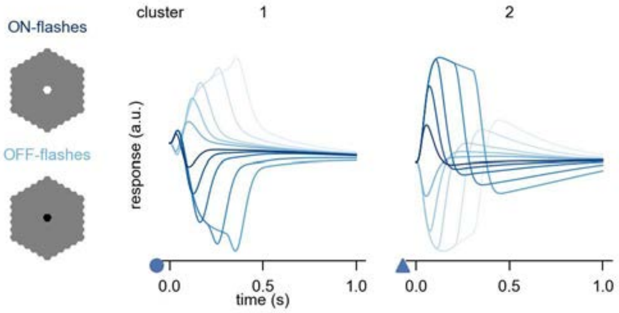

T3 - Figure 5: **Cluster-average responses to single-ommatidium flashes.** Responses to single-ommatidium ON-flashes (dark blue shades) and single-ommatidium OFF-flashes (light blue shades) of 20ms, 50ms, 100ms, 200ms, 300ms duration. The flashes occur at second zero.

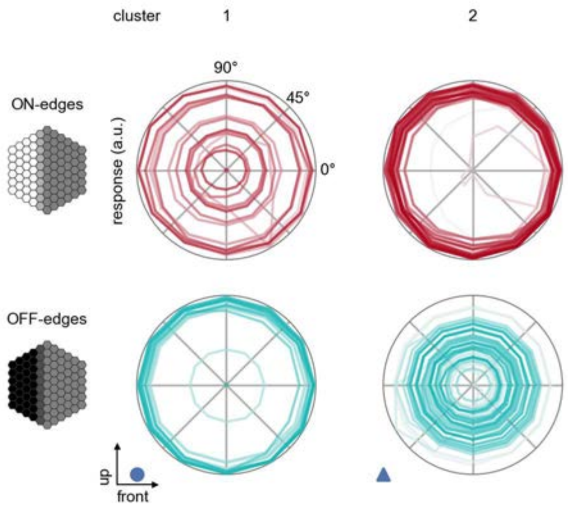

T3 - Figure 6: **Peak responses to moving edges.** The top row shows peak responses to moving ON-edges (red), the bottom row shows peak responses to moving OFF-edges (turqoise). The peak responses are averaged over edge-speeds. Edge-stimuli move in different directions from 0 to 360 degrees. The responses from the different models in the different clusters (columns) overlay. Responses from better task-performing models are more saturated.

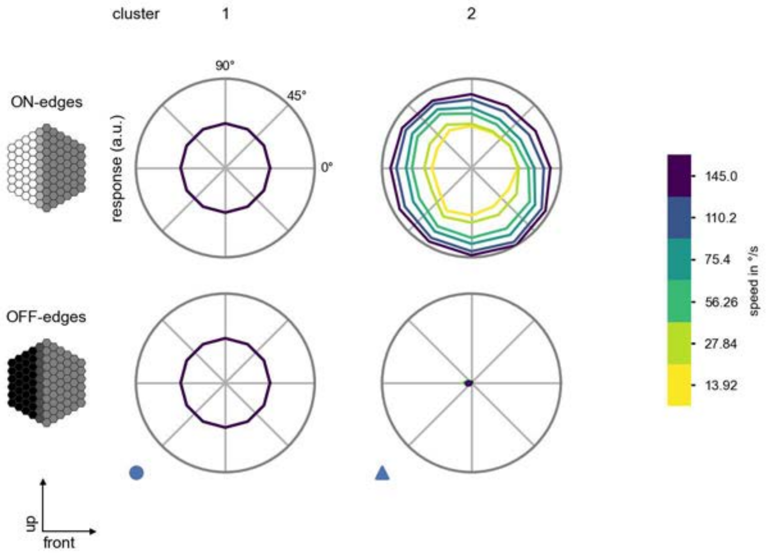

T3 - Figure 7: **Peak responses to moving edges from task-optimal models.** The top row shows peak responses to moving ON-edges, the bottom row shows peak responses to moving OFF-edges of varying speeds from 13.92°/s to 145°/s (yellow to dark blue). The edge-stimuli move in different directions from 0 to 360 degrees and at different speeds. Responses from the task-optimal model in the respective cluster.

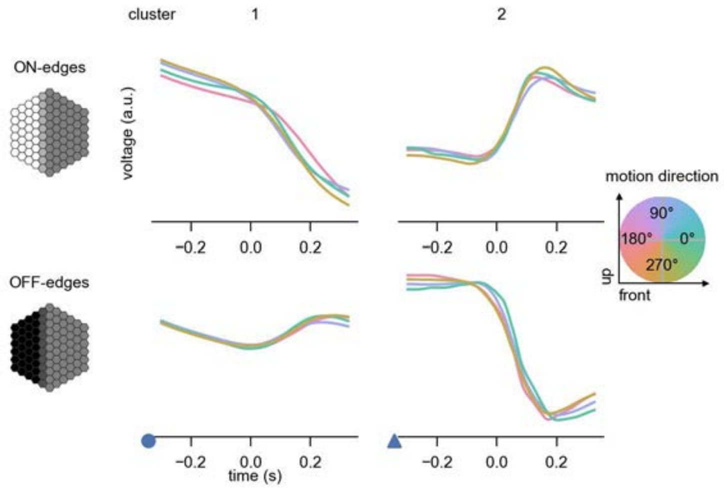

T3 - Figure 8: **Responses to moving edges from task-optimal models.** Responses to moving ON-edges (top row) and to moving OFF-edges (bottom row). Edges move in different directions from 0 to 360 degrees and at different speeds. Responses are from the task-optimal model in the respective cluster. Edges moving at 75.4°/s in all cardinal directions (green 0°, blue 90°, red 180°, yellow 270°) from -22.5 to 22.5° visual angle.

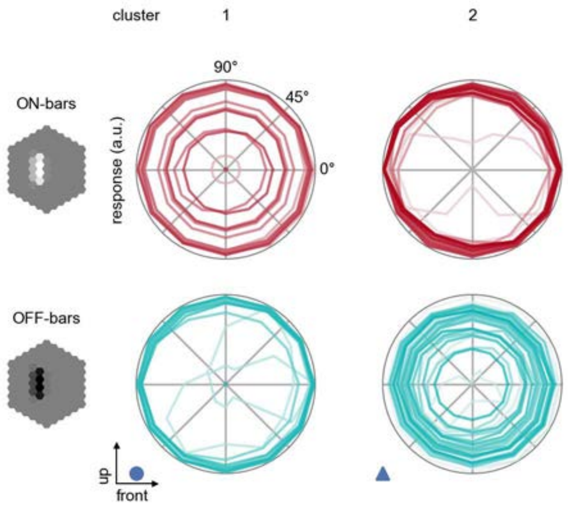

T3 - Figure 9: **Peak responses to moving bars.** The top row shows peak responses to moving ON-bars (red), the bottom row shows peak responses to moving OFF-bars (turqoise). The peak responses are averaged over bar-speeds. Bar-stimuli move in different directions from 0 to 360 degrees. The responses from the different models in the different clusters (columns) overlay. Responses from better task-performing models are more saturated.

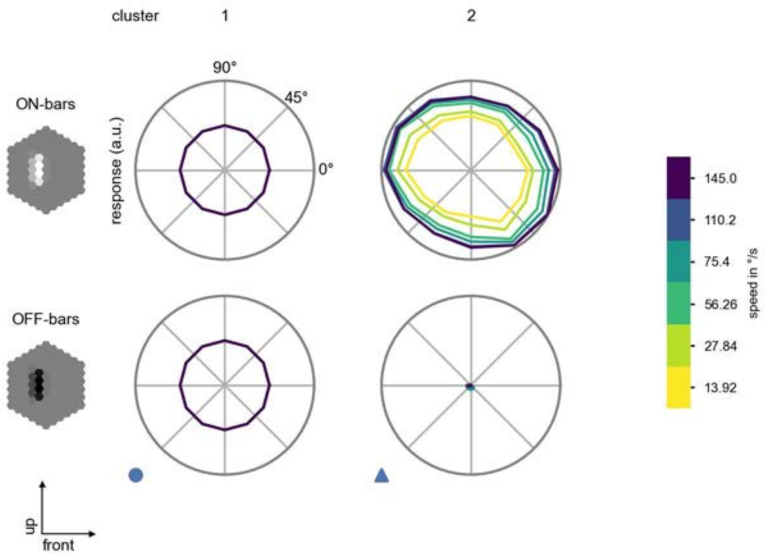

T3 - Figure 10: **Peak responses to moving bars from task-optimal models.** The top row shows peak responses to moving ON-bars, the bottom row shows peak responses to moving OFF-bars of varying speeds from 13.92°/s to 145°/s (yellow to dark blue). The bar-stimuli move in different directions from 0 to 360 degrees and at different speeds. Responses from the task-optimal model in the respective cluster.

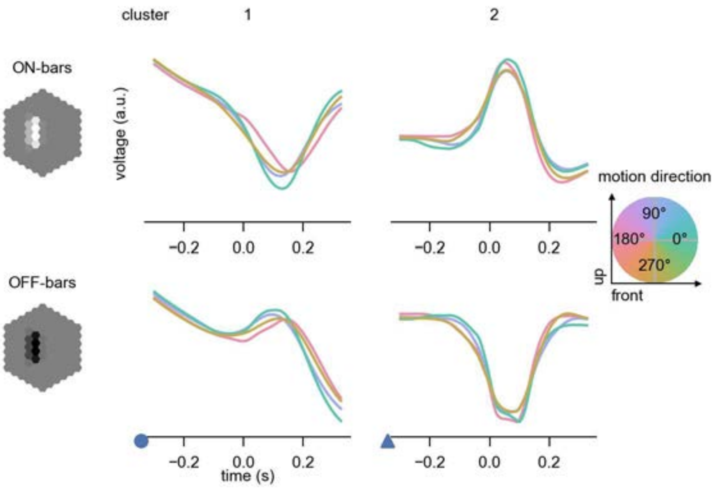

T3 - Figure 11: **Responses to moving bars from task-optimal models.** Responses to moving ON-bars (top row) and to moving OFF-bars (bottom row). Bars move in different directions from 0 to 360 degrees and at different speeds. Responses are from the task-optimal model in the respective cluster. Bars moving at 75.4°/s in all cardinal directions (green 0°, blue 90°, red 180°, yellow 270°) from -22.5 to 22.5° visual angle.

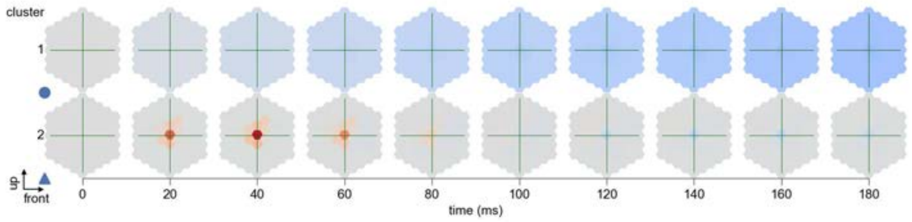

T3 - Figure 12: **Spatio-temporal receptive field.** Responses of the central cell to ON-impulses (5 ms) at single-ommatidium flash locations. The flash occurs at second zero. Responses from the task-optimal model of the respective cluster (rows). Red indicates depolarization, blue indicates hyperpolarization.

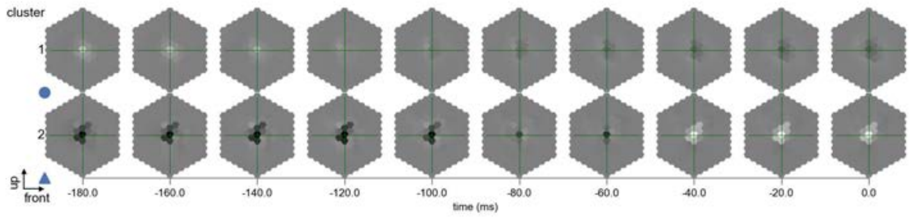

T3 - Figure 13: **Maximally excitatory stimuli.** Each row presents the regularized naturalistic-stimulus from the Sintel dataset that maximizes the cell type's central column response at second zero in the task-optimal model of the respective cluster (rows).

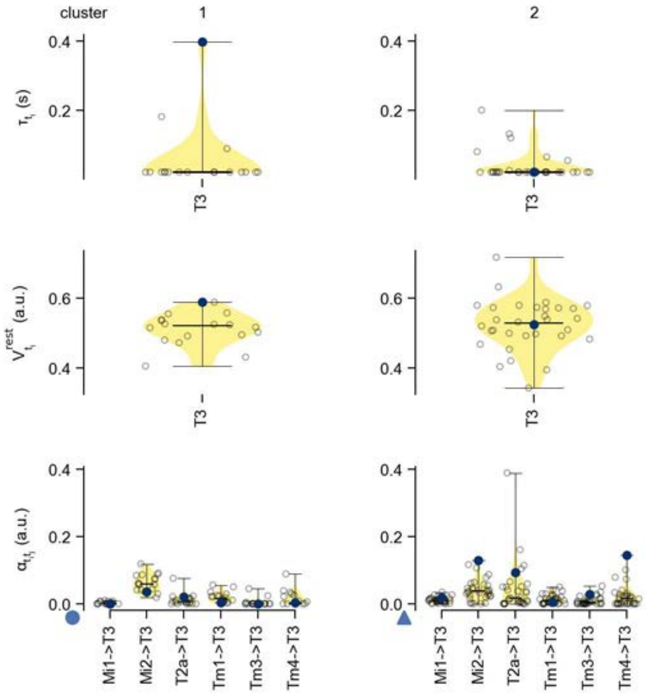

T3 - Figure 14: **Task-constrained parameters.** Each column shows the parameters inferred within the respective cluster. First row: learned time constants of the cell type. Second row: resting potentials of the cell type. Third row: scaling factors for the convolutional filters. The blue scatter represents the parameters from the task-optimal model within the cluster.

36 T4a

← Cell types

Figures

1 Anatomical receptive fields. . . . 250
2 Anatomical projective fields. . . . 251
3 Clustering of the responses to naturalistic stimuli. . . . 251
4 Responses to flashes. . . . 252
5 Cluster-average responses to single-ommatidium flashes. . . . 252
6 Peak responses to moving edges. . . . 253
7 Peak responses to moving edges from task-optimal models. . . . 253
8 Responses to moving edges from task-optimal models. . . . 254
9 Peak responses to moving bars. . . . 254
10 Peak responses to moving bars from task-optimal models. . . . 255
11 Responses to moving bars from task-optimal models. . . . 255
12 Spatio-temporal receptive field. . . . 256
13 Maximally excitatory stimuli. . . . 256
14 Task-constrained parameters. . . . 257

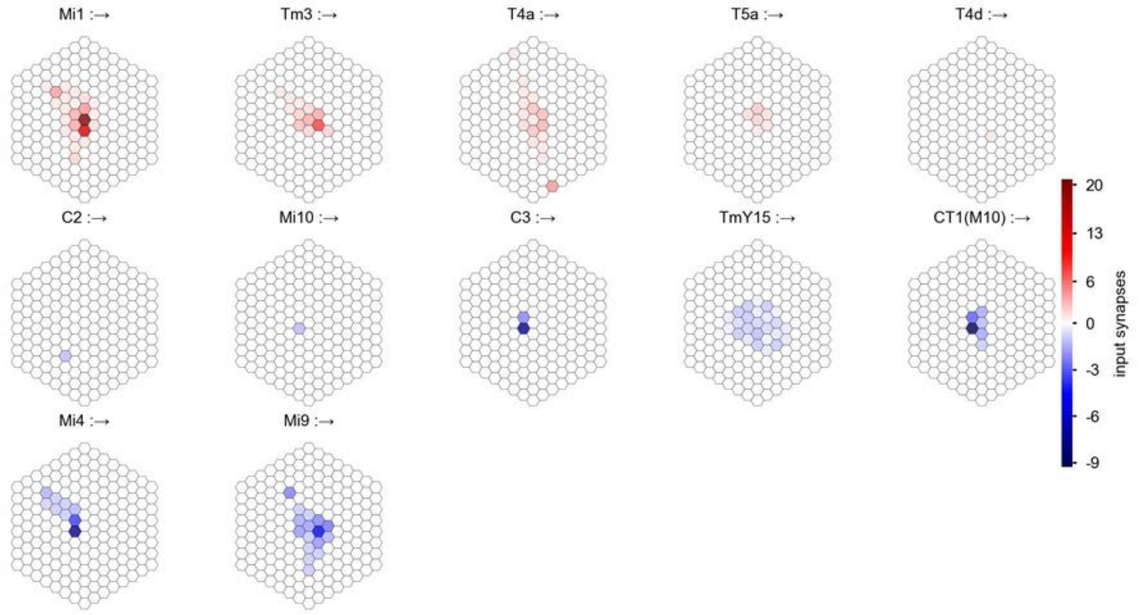

T4a - Figure 1: **Anatomical receptive fields.** Each colored hexagon is an input connection, with the connection strength characterized by the average number of synapses that we count from the EM reconstruction. Red indicates excitatory synapses, blue indicates inhibitory synapses from inferred signs. Filters in the order of their total number of synapses.

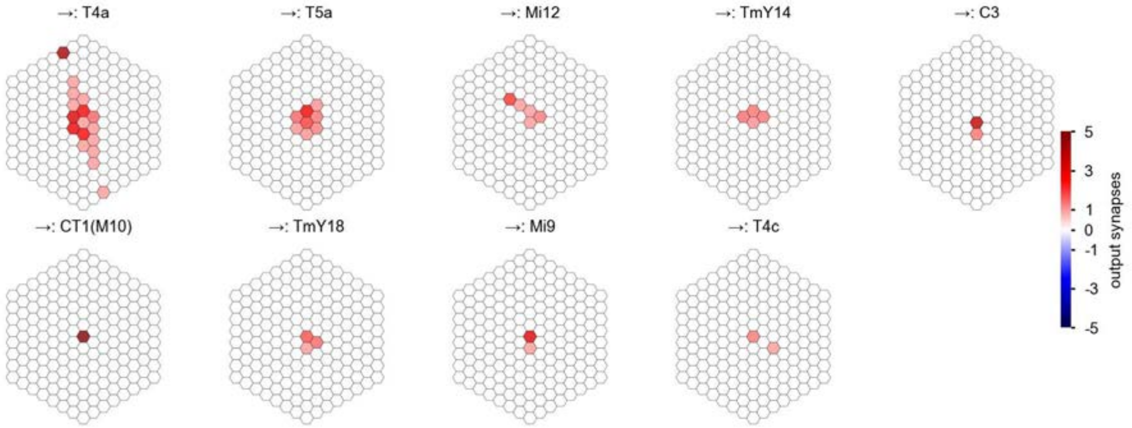

T4a - Figure 2: **Anatomical projective fields.** Each colored hexagon is an output connection, with the connection strength characterized by the average number of synapses that we count from the EM reconstruction. Red indicates excitatory synapses, blue indicates inhibitory synapses from inferred signs. Filters in the order of their total number of synapses.

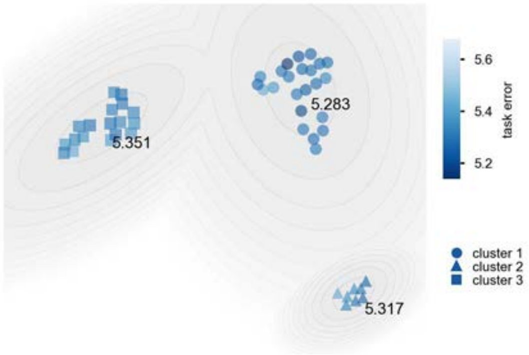

T4a - Figure 3: **Clustering of the responses to naturalistic stimuli.** Clustering of the 50 models based on the cell type responses to naturalistic scenes from the Sintel dataset. Scatterpoints represent individual models colored by their task error.

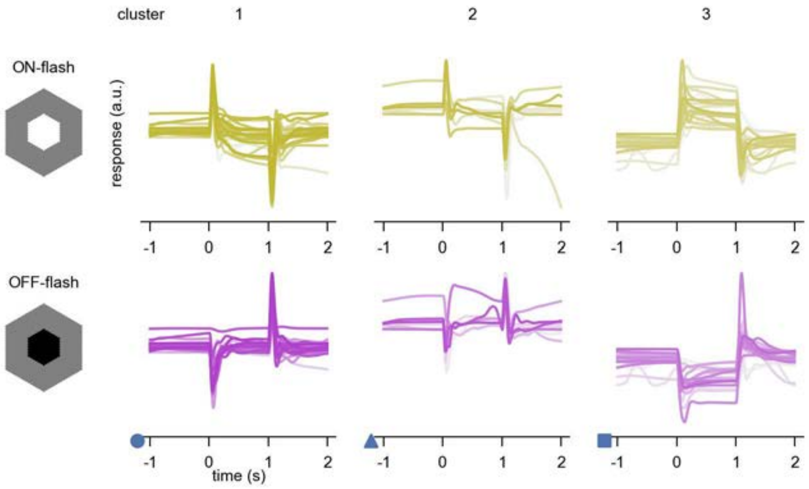

T4a - Figure 4: **Responses to flashes.** The top row shows responses to ON-flashes (yellow), the bottom row shows responses to OFF-flashes (magenta). The responses from the 50 different models that are separated into the different clusters (columns) overlay, with better task-performing models on top. Responses from better task-performing models are more saturated. The circular flashes (1s) cover 6 ommatidia in radius and are presented at time zero. Before and after, a grey-stimulus leads to a stationary state of the network.

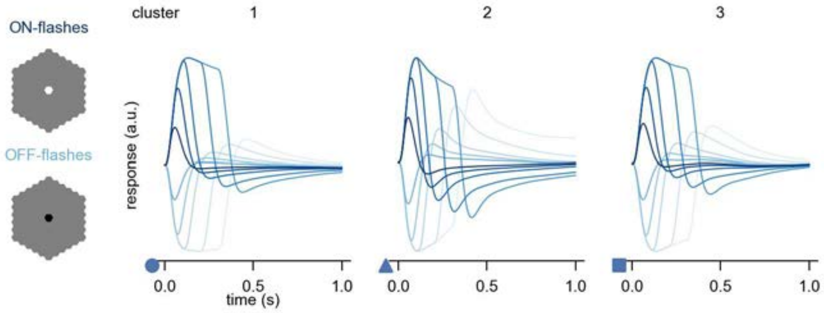

T4a - Figure 5: **Cluster-average responses to single-ommatidium flashes.** Responses to single-ommatidium ON-flashes (dark blue shades) and single-ommatidium OFF-flashes (light blue shades) of 20ms, 50ms, 100ms, 200ms, 300ms duration. The flashes occur at second zero.

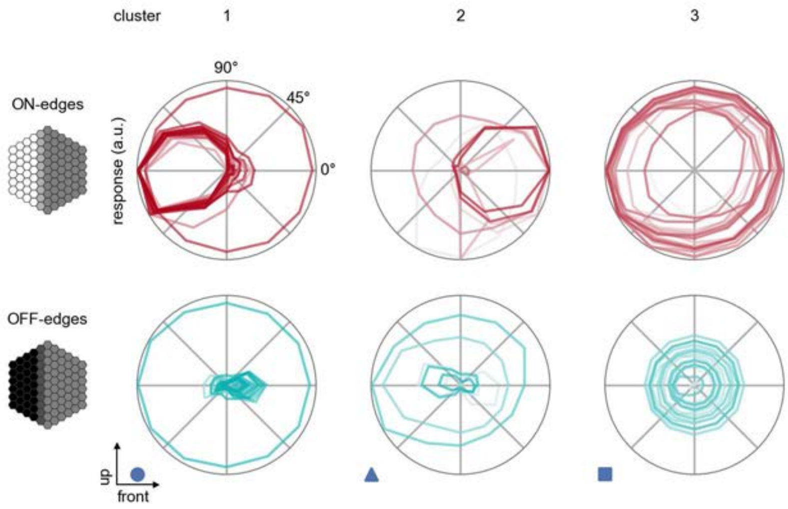

T4a - Figure 6: **Peak responses to moving edges.** The top row shows peak responses to moving ON-edges (red), the bottom row shows peak responses to moving OFF-edges (turqoise). The peak responses are averaged over edge-speeds. Edge-stimuli move in different directions from 0 to 360 degrees. The responses from the different models in the different clusters (columns) overlay. Responses from better task-performing models are more saturated.

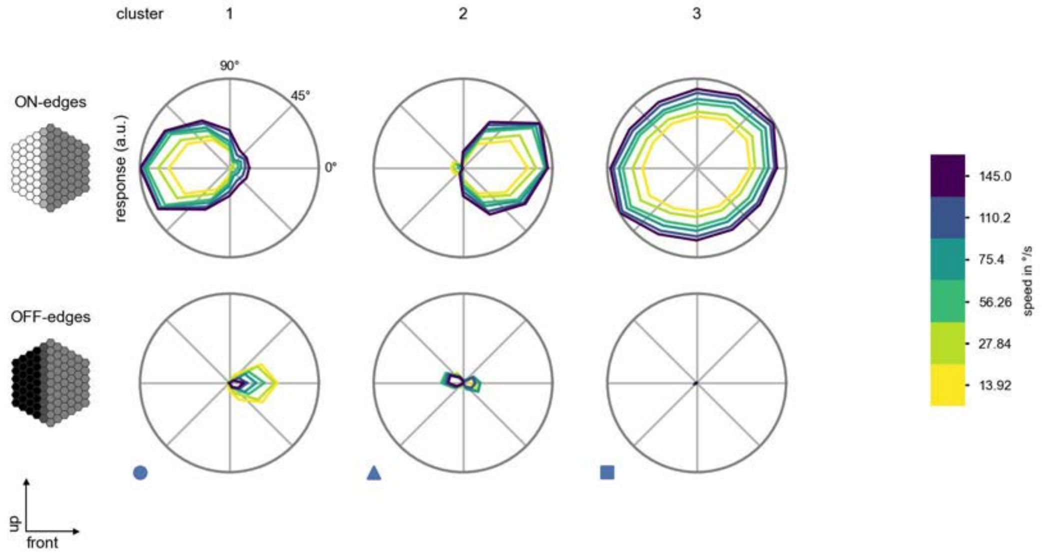

T4a - Figure 7: **Peak responses to moving edges from task-optimal models.** The top row shows peak responses to moving ON-edges, the bottom row shows peak responses to moving OFF-edges of varying speeds from 13.92°/s to 145°/s (yellow to dark blue). The edge-stimuli move in different directions from 0 to 360 degrees and at different speeds. Responses from the task-optimal model in the respective cluster.

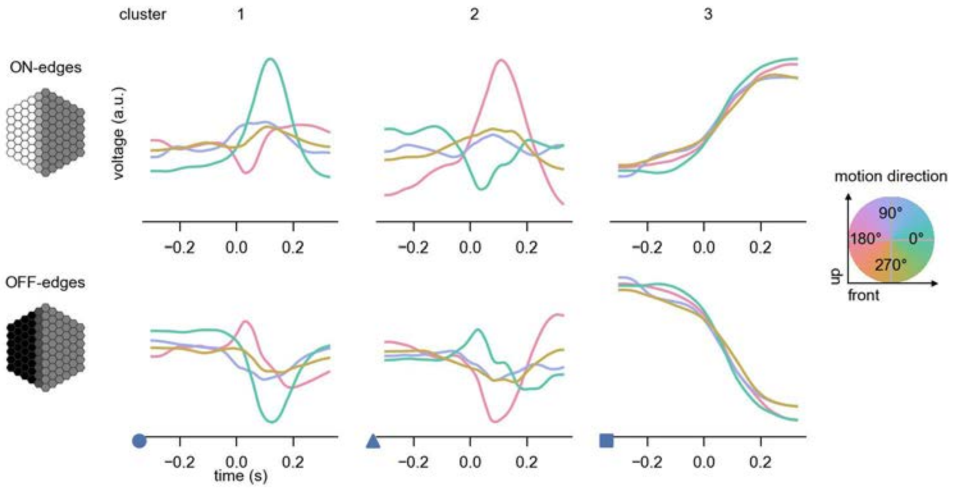

T4a - Figure 8: **Responses to moving edges from task-optimal models.** Responses to moving ON-edges (top row) and to moving OFF-edges (bottom row). Edges move in different directions from 0 to 360 degrees and at different speeds. Responses are from the task-optimal model in the respective cluster. Edges moving at 75.4°/s in all cardinal directions (green 0°, blue 90°, red 180°, yellow 270°) from -22.5 to 22.5° visual angle.

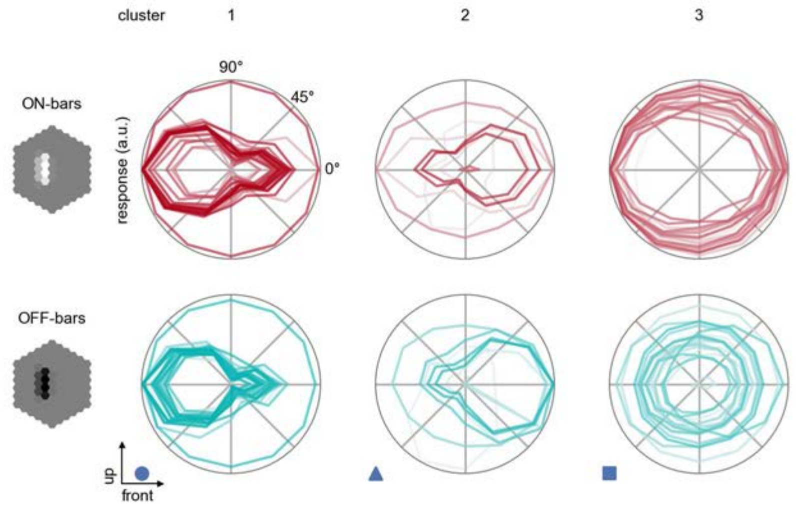

T4a - Figure 9: **Peak responses to moving bars.** The top row shows peak responses to moving ON-bars (red), the bottom row shows peak responses to moving OFF-bars (turqoise). The peak responses are averaged over bar-speeds. Bar-stimuli move in different directions from 0 to 360 degrees. The responses from the different models in the different clusters (columns) overlay. Responses from better task-performing models are more saturated.

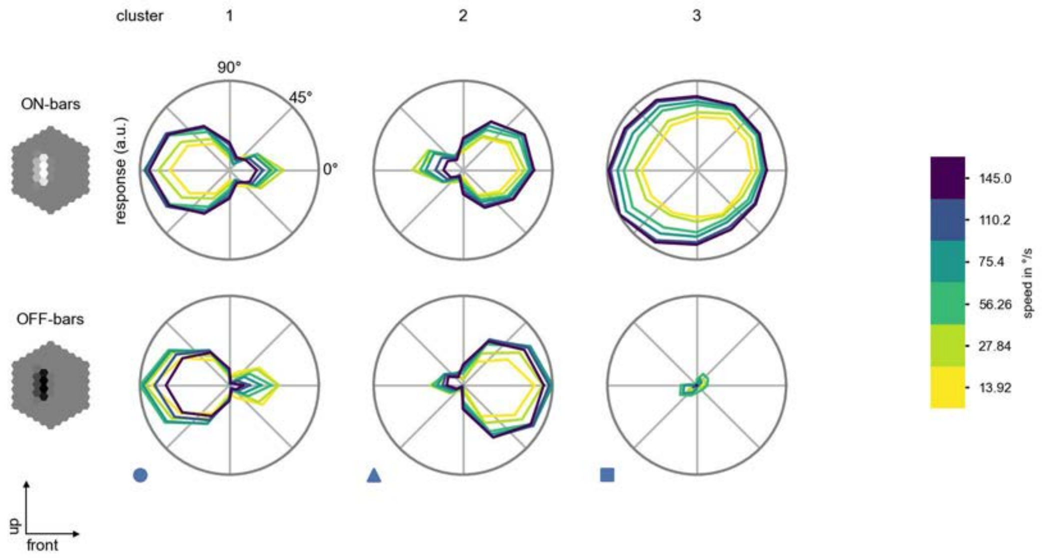

T4a - Figure 10: **Peak responses to moving bars from task-optimal models.** The top row shows peak responses to moving ON-bars, the bottom row shows peak responses to moving OFF-bars of varying speeds from 13.92°/s to 145°/s (yellow to dark blue). The bar-stimuli move in different directions from 0 to 360 degrees and at different speeds. Responses from the task-optimal model in the respective cluster.

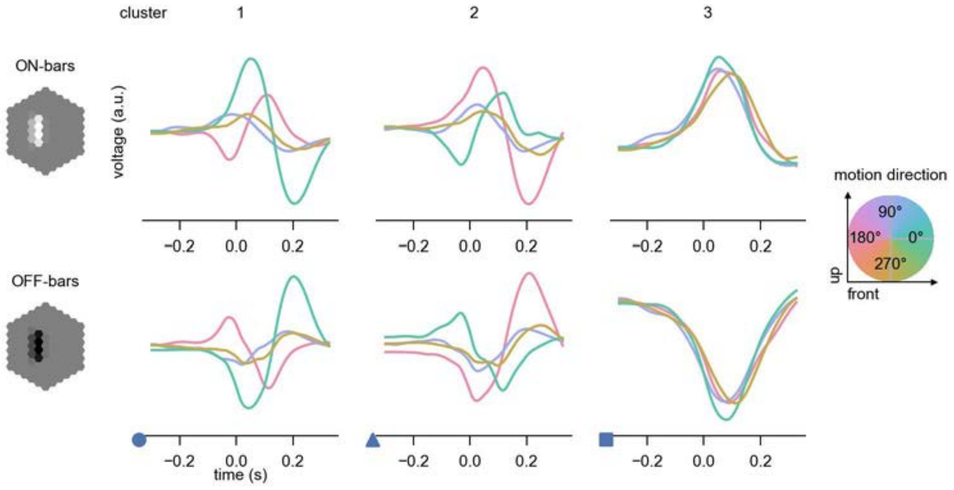

T4a - Figure 11: **Responses to moving bars from task-optimal models.** Responses to moving ON-bars (top row) and to moving OFF-bars (bottom row). Bars move in different directions from 0 to 360 degrees and at different speeds. Responses are from the task-optimal model in the respective cluster. Bars moving at 75.4°/s in all cardinal directions (green 0°, blue 90°, red 180°, yellow 270°) from -22.5 to 22.5° visual angle.

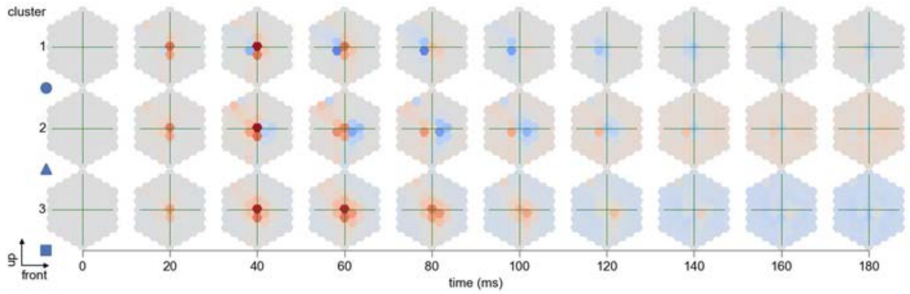

T4a - Figure 12: **Spatio-temporal receptive field.** Responses of the central cell to ON-impulses (5 ms) at single-ommatidium flash locations. The flash occurs at second zero. Responses from the task-optimal model of the respective cluster (rows). Red indicates depolarization, blue indicates hyperpolarization.

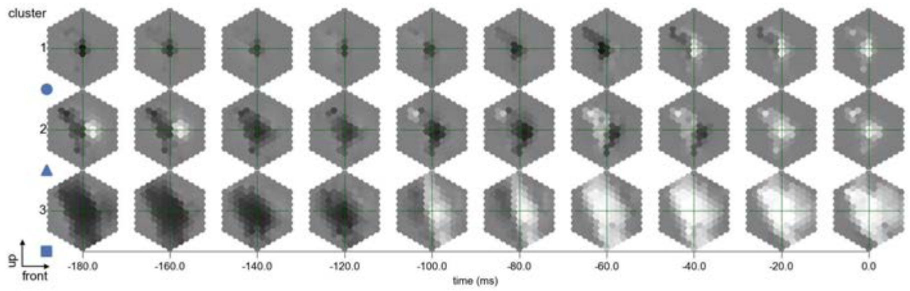

T4a - Figure 13: **Maximally excitatory stimuli.** Each row presents the regularized naturalistic-stimulus from the Sintel dataset that maximizes the cell type's central column response at second zero in the task-optimal model of the respective cluster (rows).

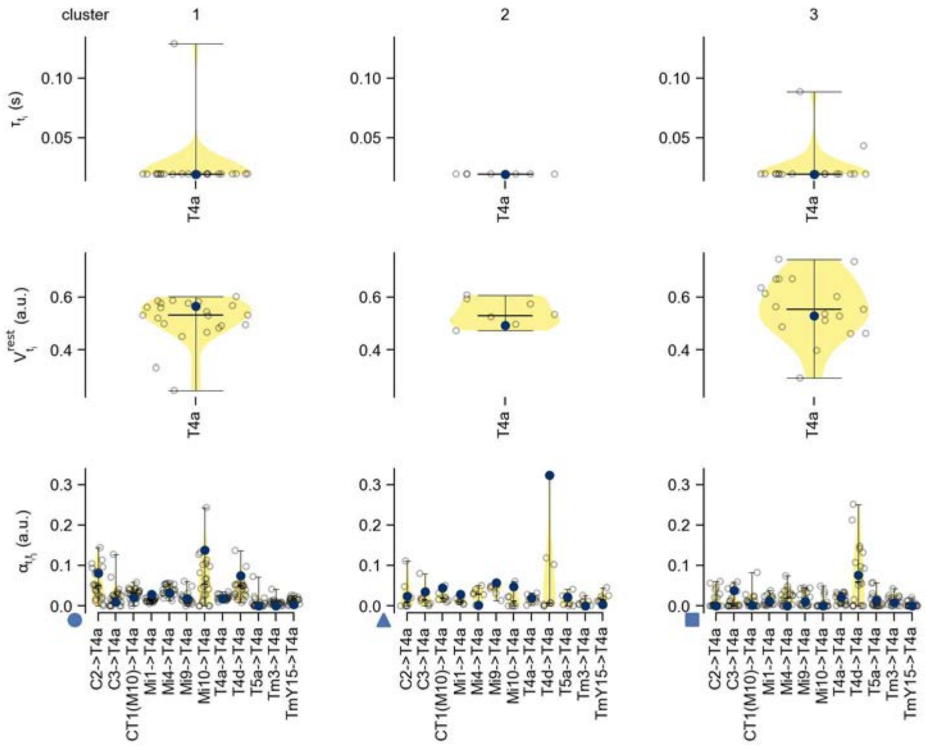

T4a - Figure 14: **Task-constrained parameters.** Each column shows the parameters inferred within the respective cluster. First row: learned time constants of the cell type. Second row: resting potentials of the cell type. Third row: scaling factors for the convolutional filters. The blue scatter represents the parameters from the task-optimal model within the cluster.

37 T4b

← Cell types

Figures

1 Anatomical receptive fields. 258
2 Anatomical projective fields. 259
3 Clustering of the responses to naturalistic stimuli. 259
4 Responses to flashes. 260
5 Cluster-average responses to single-ommatidium flashes. 260
6 Peak responses to moving edges. 261
7 Peak responses to moving edges from task-optimal models. 261
8 Responses to moving edges from task-optimal models. 262
9 Peak responses to moving bars. 262
10 Peak responses to moving bars from task-optimal models. 263
11 Responses to moving bars from task-optimal models. 263
12 Spatio-temporal receptive field. 264
13 Maximally excitatory stimuli. 264
14 Task-constrained parameters. 265

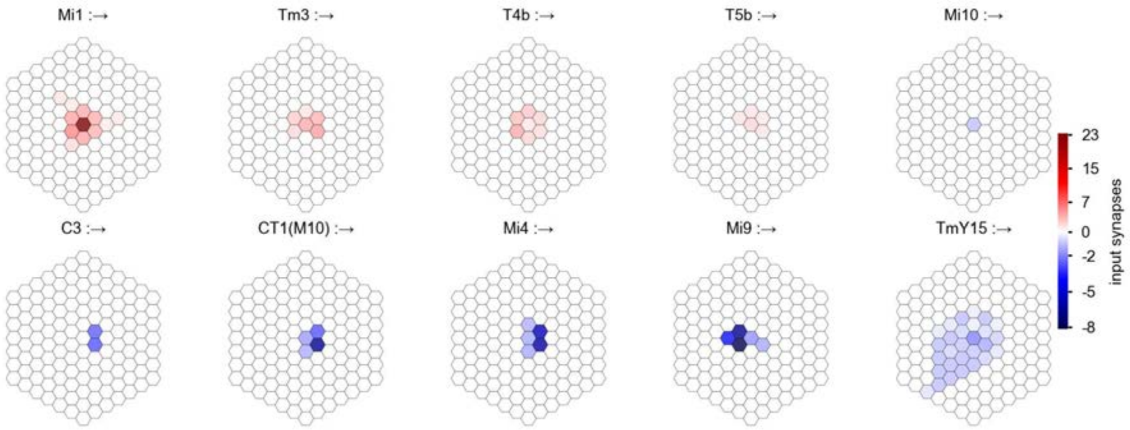

T4b - Figure 1: **Anatomical receptive fields.** Each colored hexagon is an input connection, with the connection strength characterized by the average number of synapses that we count from the EM reconstruction. Red indicates excitatory synapses, blue indicates inhibitory synapses from inferred signs. Filters in the order of their total number of synapses.

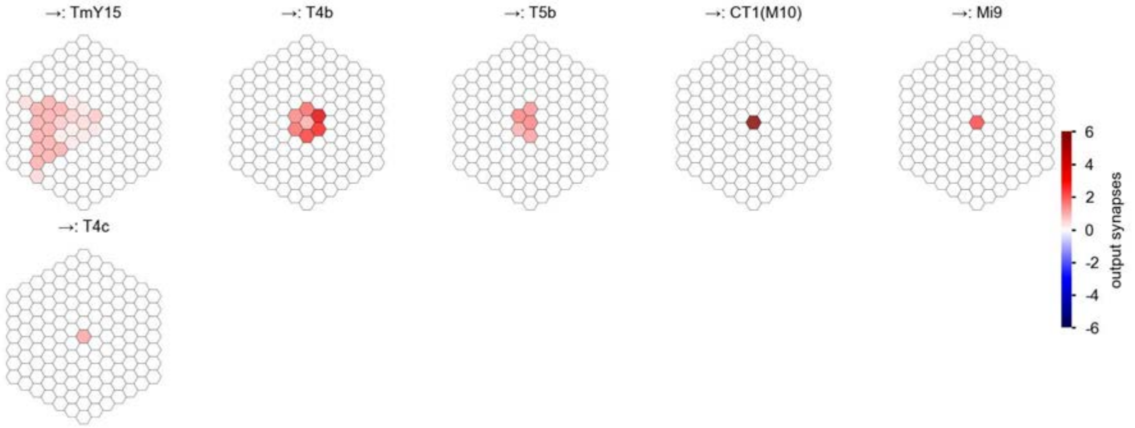

T4b - Figure 2: **Anatomical projective fields.** Each colored hexagon is an output connection, with the connection strength characterized by the average number of synapses that we count from the EM reconstruction. Red indicates excitatory synapses, blue indicates inhibitory synapses from inferred signs. Filters in the order of their total number of synapses.

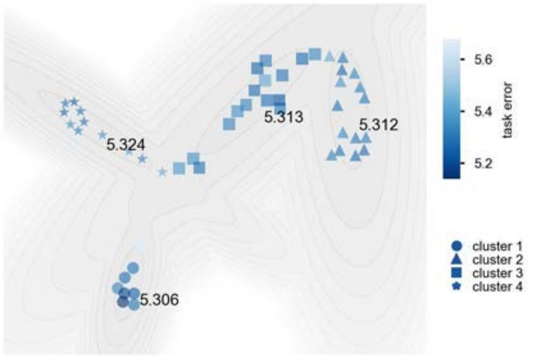

T4b - Figure 3: **Clustering of the responses to naturalistic stimuli.** Clustering of the 50 models based on the cell type responses to naturalistic scenes from the Sintel dataset. Scatterpoints represent individual models colored by their task error.

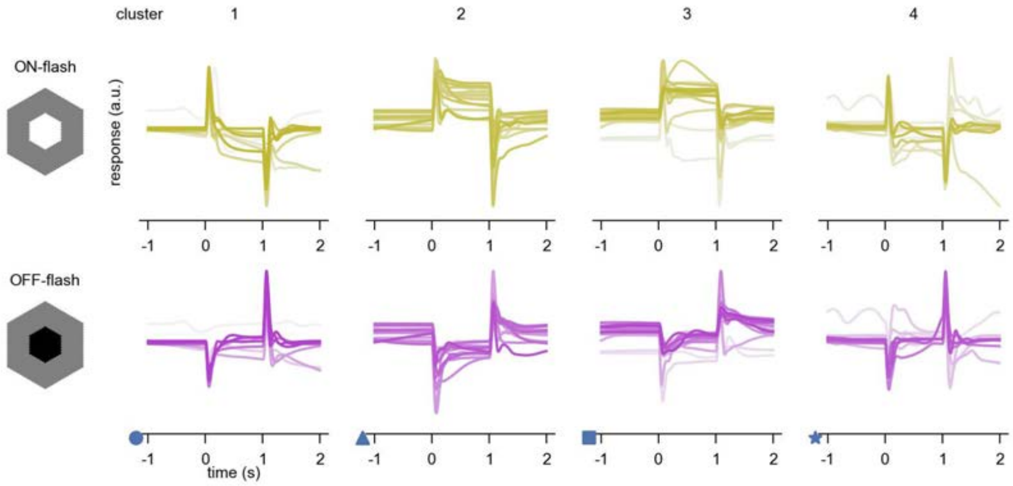

T4b - Figure 4: **Responses to flashes.** The top row shows responses to ON-flashes (yellow), the bottom row shows responses to OFF-flashes (magenta). The responses from the 50 different models that are separated into the different clusters (columns) overlay, with better task-performing models on top. Responses from better task-performing models are more saturated. The circular flashes (1s) cover 6 ommatidia in radius and are presented at time zero. Before and after, a grey-stimulus leads to a stationary state of the network.

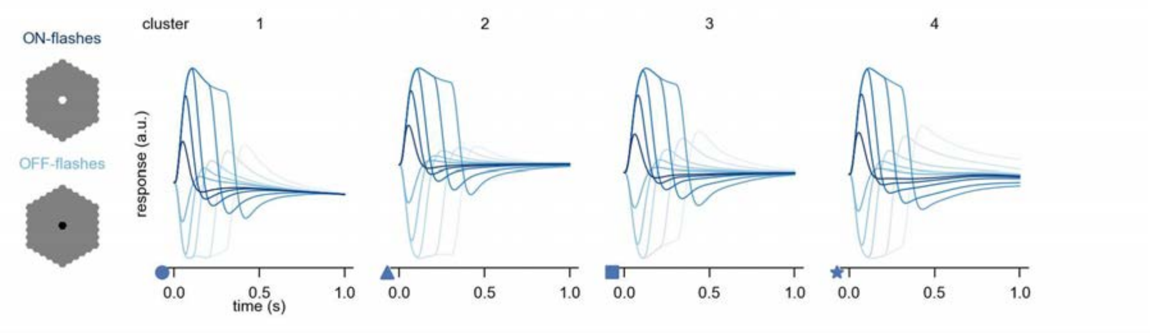

T4b - Figure 5: **Cluster-average responses to single-ommatidium flashes.** Responses to single-ommatidium ON-flashes (dark blue shades) and single-ommatidium OFF-flashes (light blue shades) of 20ms, 50ms, 100ms, 200ms, 300ms duration. The flashes occur at second zero.

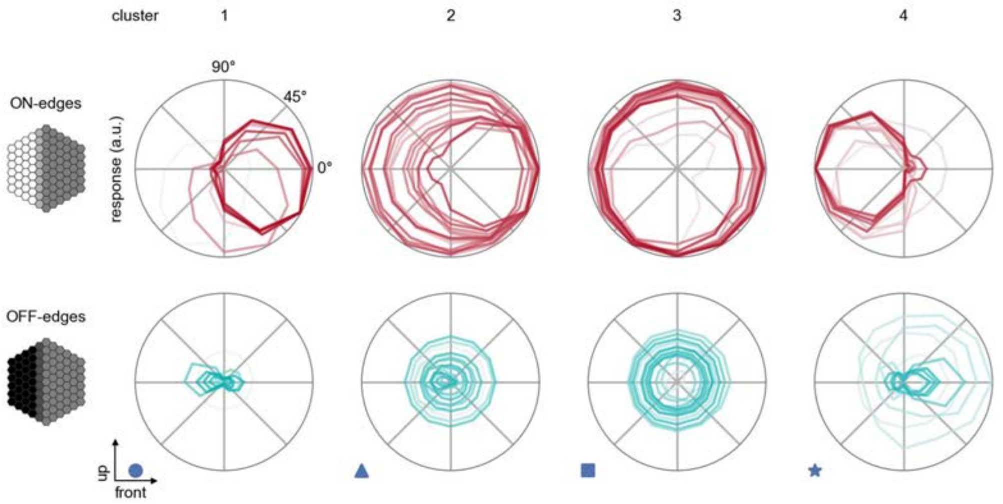

T4b - Figure 6: **Peak responses to moving edges.** The top row shows peak responses to moving ON-edges (red), the bottom row shows peak responses to moving OFF-edges (turqoise). The peak responses are averaged over edge-speeds. Edge-stimuli move in different directions from 0 to 360 degrees. The responses from the different models in the different clusters (columns) overlay. Responses from better task-performing models are more saturated.

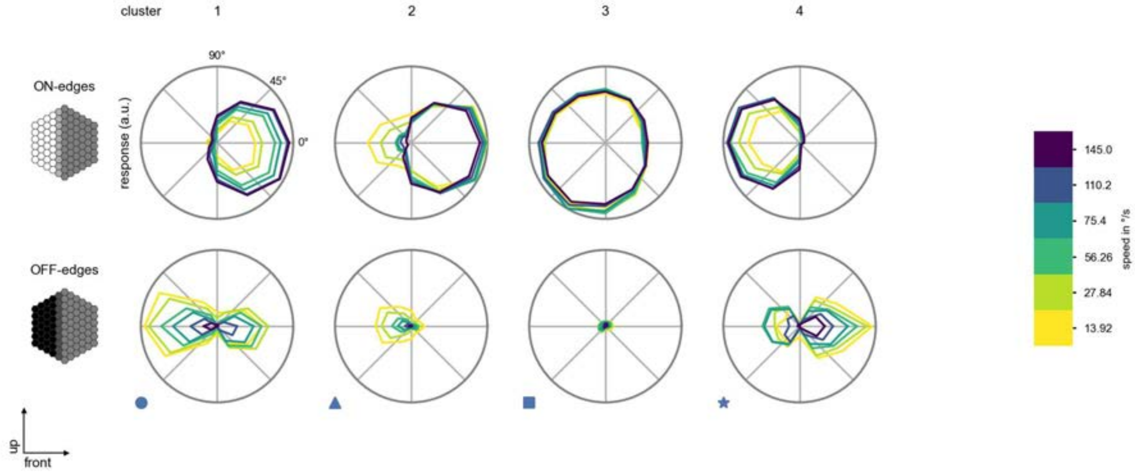

T4b - Figure 7: **Peak responses to moving edges from task-optimal models.** The top row shows peak responses to moving ON-edges, the bottom row shows peak responses to moving OFF-edges of varying speeds from 13.92°/s to 145°/s (yellow to dark blue). The edge-stimuli move in different directions from 0 to 360 degrees and at different speeds. Responses from the task-optimal model in the respective cluster.

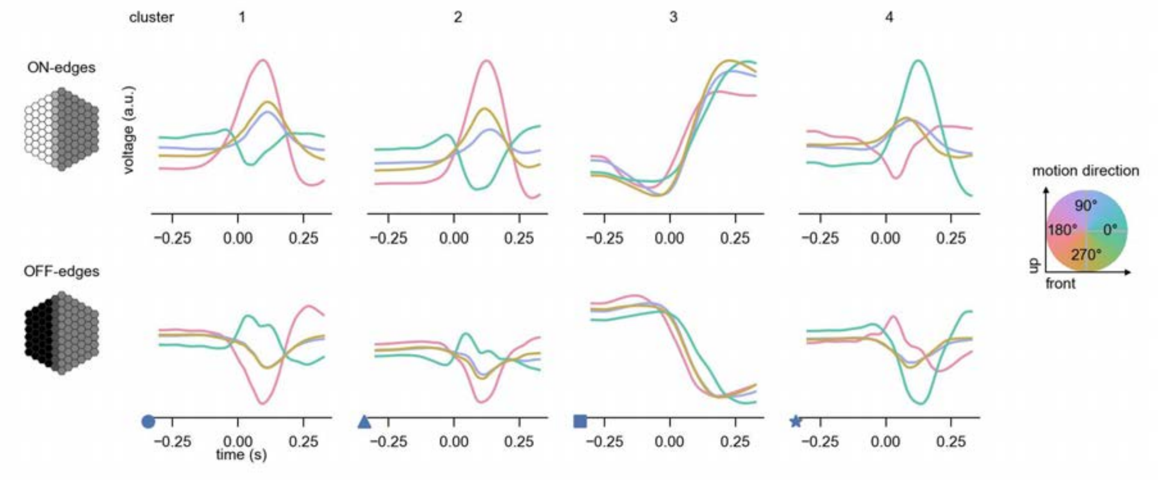

T4b - Figure 8: **Responses to moving edges from task-optimal models.** Responses to moving ON-edges (top row) and to moving OFF-edges (bottom row). Edges move in different directions from 0 to 360 degrees and at different speeds. Responses are from the task-optimal model in the respective cluster. Edges moving at 75.4°/s in all cardinal directions (green 0°, blue 90°, red 180°, yellow 270°) from -22.5 to 22.5° visual angle.

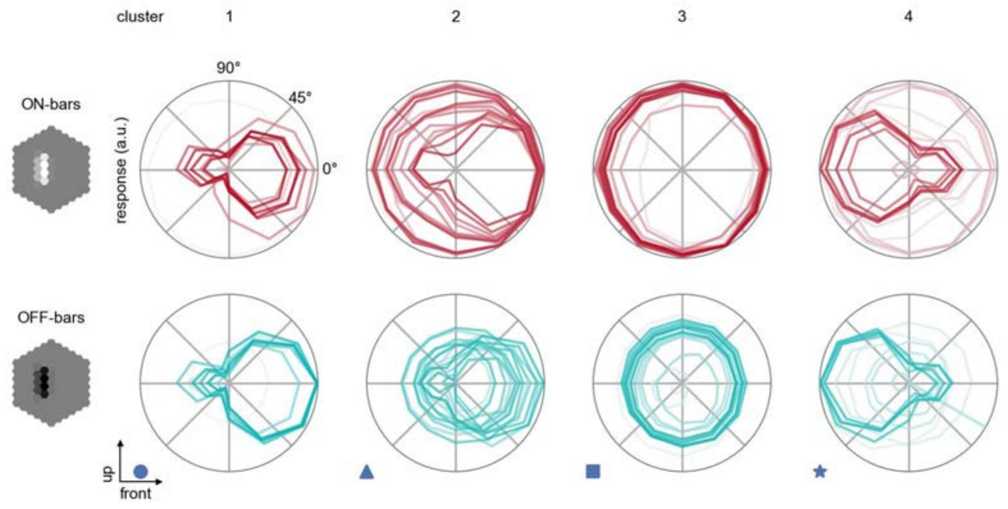

T4b - Figure 9: **Peak responses to moving bars.** The top row shows peak responses to moving ON-bars (red), the bottom row shows peak responses to moving OFF-bars (turqoise). The peak responses are averaged over bar-speeds. Bar-stimuli move in different directions from 0 to 360 degrees. The responses from the different models in the different clusters (columns) overlay. Responses from better task-performing models are more saturated.

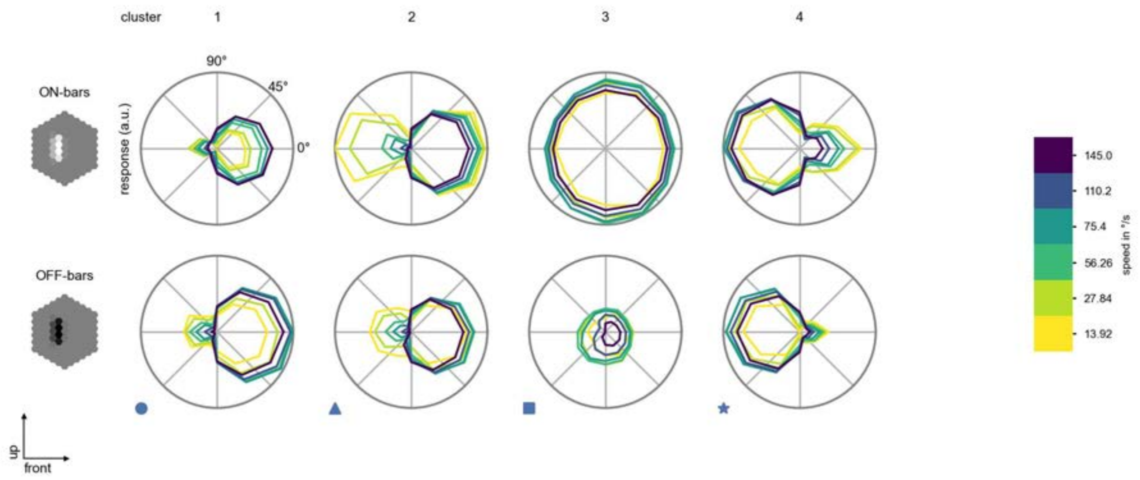

T4b - Figure 10: **Peak responses to moving bars from task-optimal models.** The top row shows peak responses to moving ON-bars, the bottom row shows peak responses to moving OFF-bars of varying speeds from 13.92°/s to 145°/s (yellow to dark blue). The bar-stimuli move in different directions from 0 to 360 degrees and at different speeds. Responses from the task-optimal model in the respective cluster.

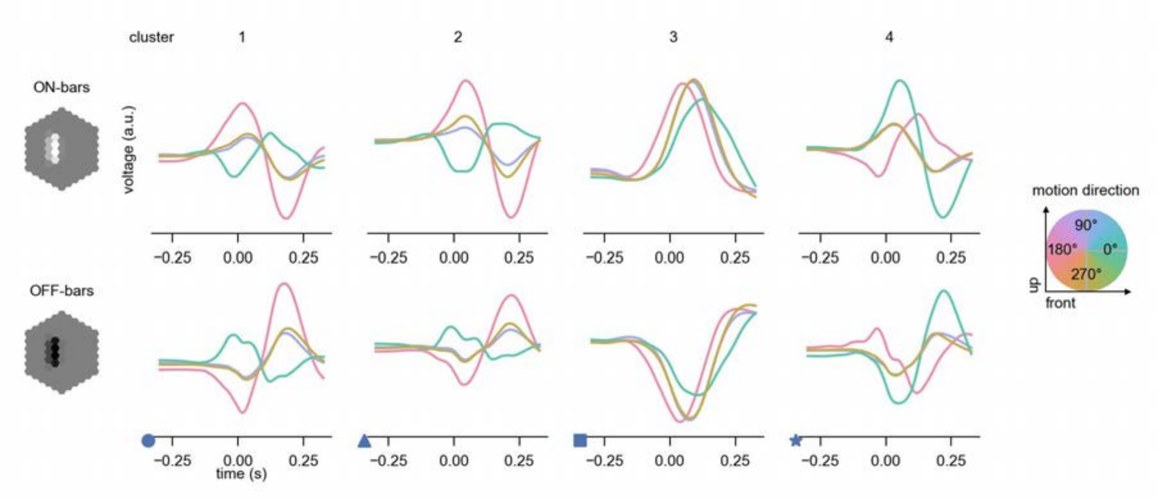

T4b - Figure 11: **Responses to moving bars from task-optimal models.** Responses to moving ON-bars (top row) and to moving OFF-bars (bottom row). Bars move in different directions from 0 to 360 degrees and at different speeds. Responses are from the task-optimal model in the respective cluster. Bars moving at 75.4°/s in all cardinal directions (green 0°, blue 90°, red 180°, yellow 270°) from -22.5 to 22.5° visual angle.

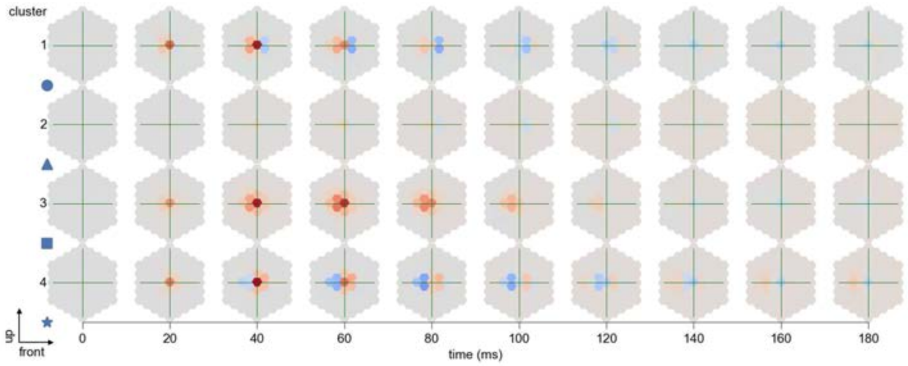

T4b - Figure 12: **Spatio-temporal receptive field.** Responses of the central cell to ON-impulses (5 ms) at single-ommatidium flash locations. The flash occurs at second zero. Responses from the task-optimal model of the respective cluster (rows). Red indicates depolarization, blue indicates hyperpolarization.

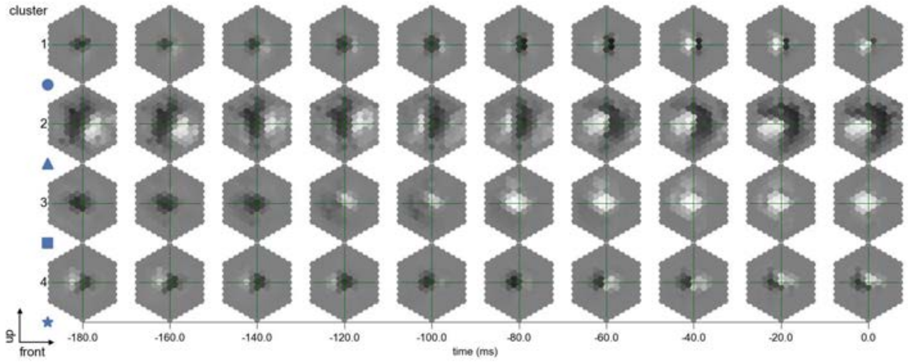

T4b - Figure 13: **Maximally excitatory stimuli.** Each row presents the regularized naturalistic-stimulus from the Sintel dataset that maximizes the cell type's central column response at second zero in the task-optimal model of the respective cluster (rows).

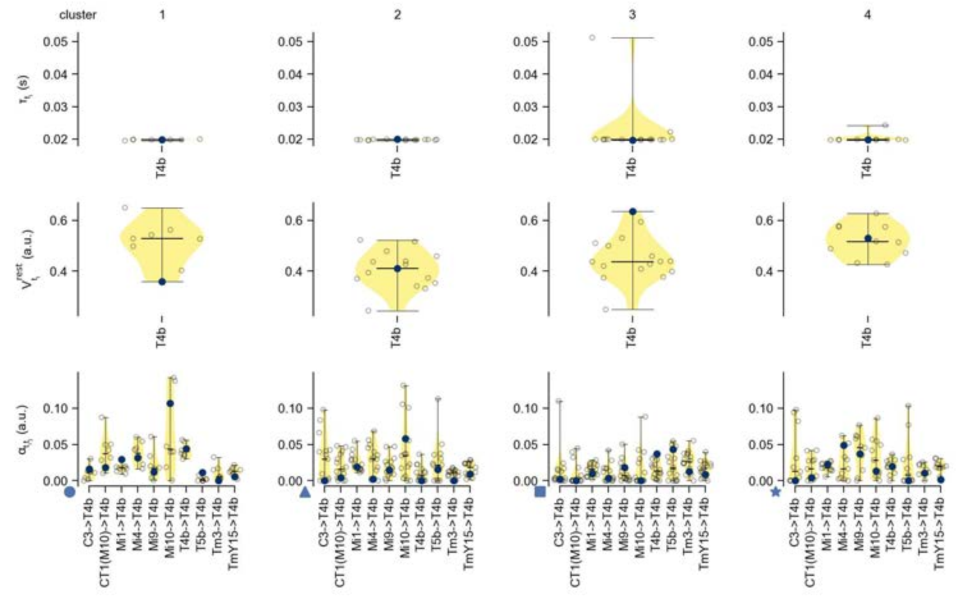

T4b - Figure 14: **Task-constrained parameters.** Each column shows the parameters inferred within the respective cluster. First row: learned time constants of the cell type. Second row: resting potentials of the cell type. Third row: scaling factors for the convolutional filters. The blue scatter represents the parameters from the task-optimal model within the cluster.

38 T4c

← Cell types

Figures

1 Anatomical receptive fields. . . . 266
2 Anatomical projective fields. . . . 267
3 Clustering of the responses to naturalistic stimuli. . . . 267
4 Responses to flashes. . . . 268
5 Cluster-average responses to single-ommatidium flashes. . . . 268
6 Peak responses to moving edges. . . . 269
7 Peak responses to moving edges from task-optimal models. . . . 269
8 Responses to moving edges from task-optimal models. . . . 270
9 Peak responses to moving bars. . . . 270
10 Peak responses to moving bars from task-optimal models. . . . 271
11 Responses to moving bars from task-optimal models. . . . 271
12 Spatio-temporal receptive field. . . . 272
13 Maximally excitatory stimuli. . . . 272
14 Task-constrained parameters. . . . 273

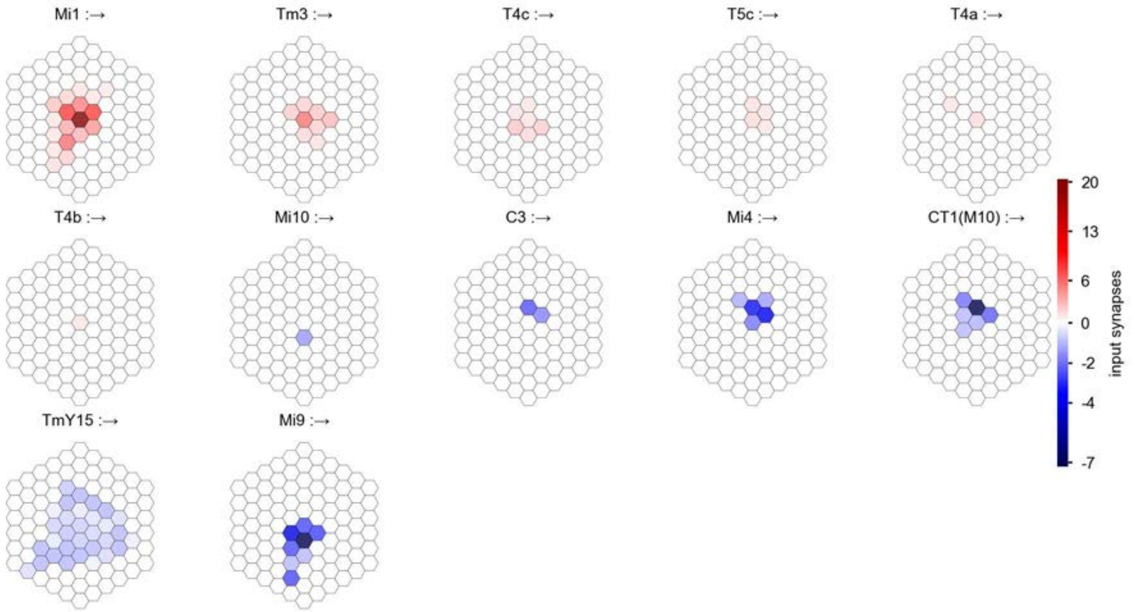

T4c - Figure 1: **Anatomical receptive fields.** Each colored hexagon is an input connection, with the connection strength characterized by the average number of synapses that we count from the EM reconstruction. Red indicates excitatory synapses, blue indicates inhibitory synapses from inferred signs. Filters in the order of their total number of synapses.

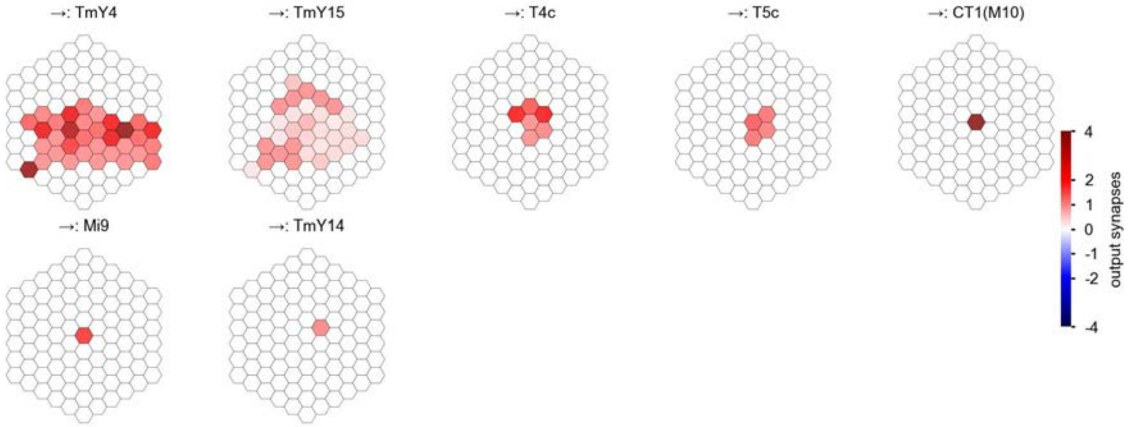

T4c - Figure 2: **Anatomical projective fields.** Each colored hexagon is an output connection, with the connection strength characterized by the average number of synapses that we count from the EM reconstruction. Red indicates excitatory synapses, blue indicates inhibitory synapses from inferred signs. Filters in the order of their total number of synapses.

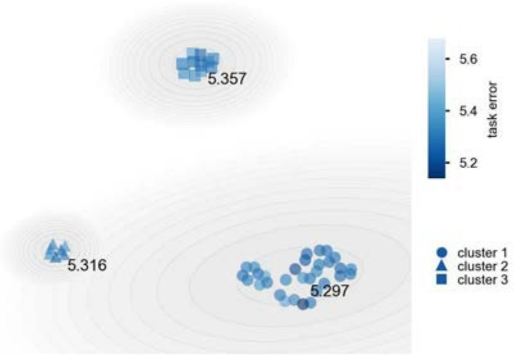

T4c - Figure 3: **Clustering of the responses to naturalistic stimuli.** Clustering of the 50 models based on the cell type responses to naturalistic scenes from the Sintel dataset. Scatterpoints represent individual models colored by their task error.

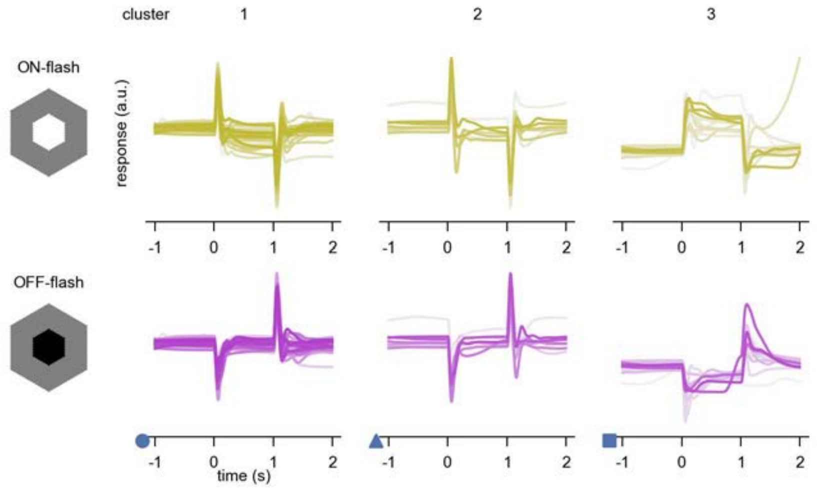

T4c - Figure 4: **Responses to flashes.** The top row shows responses to ON-flashes (yellow), the bottom row shows responses to OFF-flashes (magenta). The responses from the 50 different models that are separated into the different clusters (columns) overlay, with better task-performing models on top. Responses from better task-performing models are more saturated. The circular flashes (1s) cover 6 ommatidia in radius and are presented at time zero. Before and after, a grey-stimulus leads to a stationary state of the network.

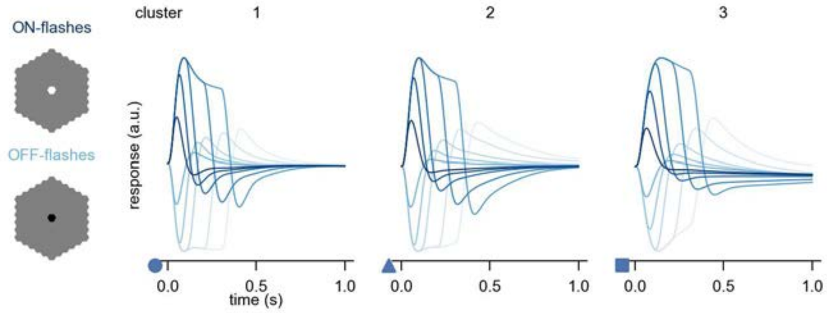

T4c - Figure 5: **Cluster-average responses to single-ommatidium flashes.** Responses to single-ommatidium ON-flashes (dark blue shades) and single-ommatidium OFF-flashes (light blue shades) of 20ms, 50ms, 100ms, 200ms, 300ms duration. The flashes occur at second zero.

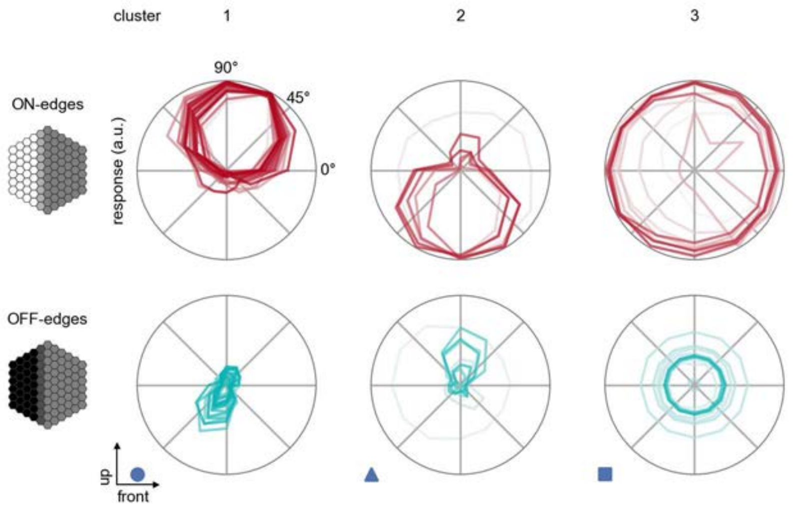

T4c - Figure 6: **Peak responses to moving edges.** The top row shows peak responses to moving ON-edges (red), the bottom row shows peak responses to moving OFF-edges (turqoise). The peak responses are averaged over edge-speeds. Edge-stimuli move in different directions from 0 to 360 degrees. The responses from the different models in the different clusters (columns) overlay. Responses from better task-performing models are more saturated.

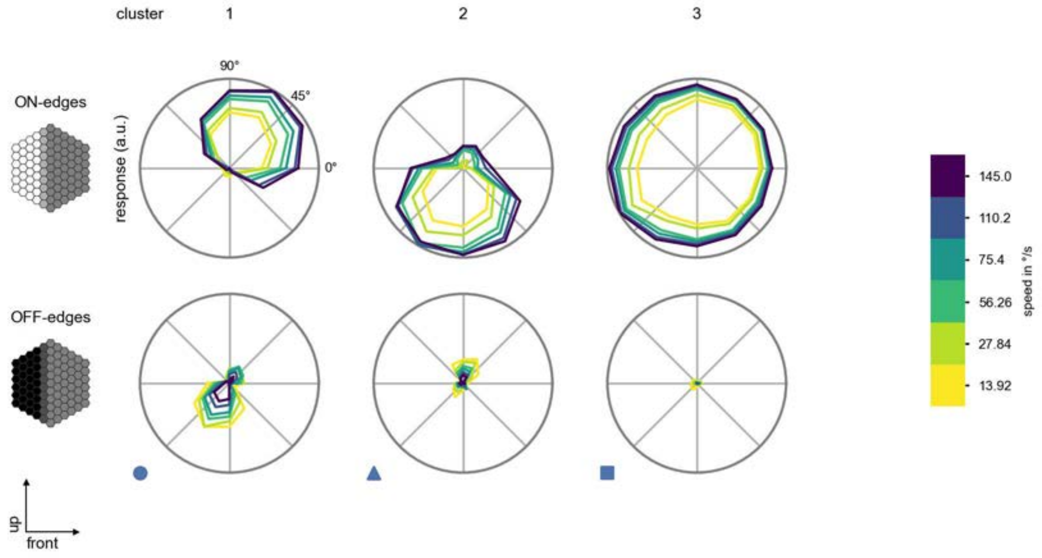

T4c - Figure 7: **Peak responses to moving edges from task-optimal models.** The top row shows peak responses to moving ON-edges, the bottom row shows peak responses to moving OFF-edges of varying speeds from 13.92°/s to 145°/s (yellow to dark blue). The edge-stimuli move in different directions from 0 to 360 degrees and at different speeds. Responses from the task-optimal model in the respective cluster.

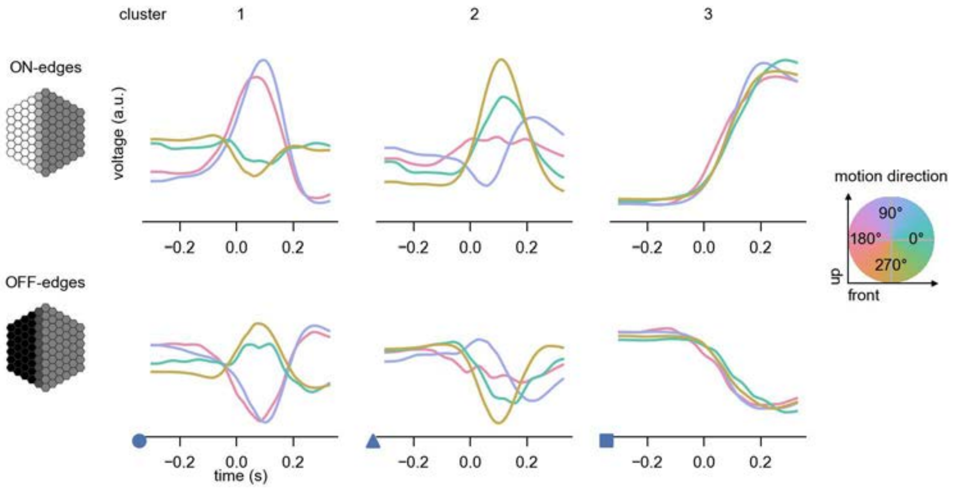

T4c - Figure 8: **Responses to moving edges from task-optimal models.** Responses to moving ON-edges (top row) and to moving OFF-edges (bottom row). Edges move in different directions from 0 to 360 degrees and at different speeds. Responses are from the task-optimal model in the respective cluster. Edges moving at 75.4°/s in all cardinal directions (green 0°, blue 90°, red 180°, yellow 270°) from -22.5 to 22.5° visual angle.

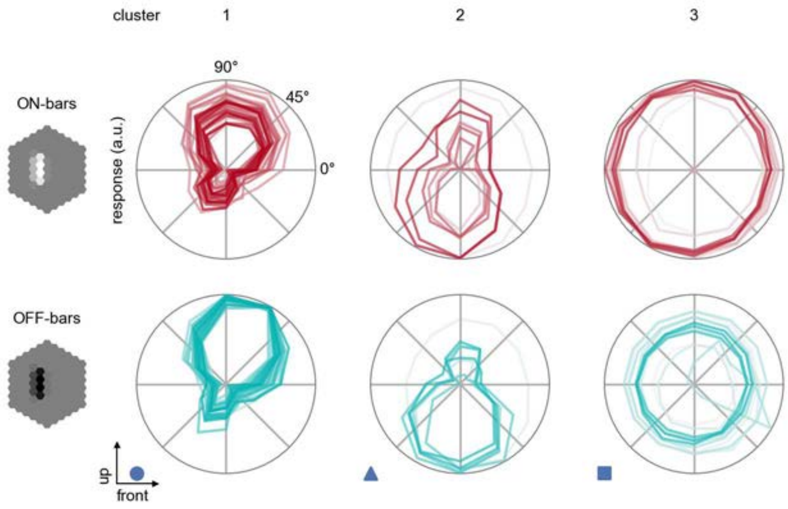

T4c - Figure 9: **Peak responses to moving bars.** The top row shows peak responses to moving ON-bars (red), the bottom row shows peak responses to moving OFF-bars (turqoise). The peak responses are averaged over bar-speeds. Bar-stimuli move in different directions from 0 to 360 degrees. The responses from the different models in the different clusters (columns) overlay. Responses from better task-performing models are more saturated.

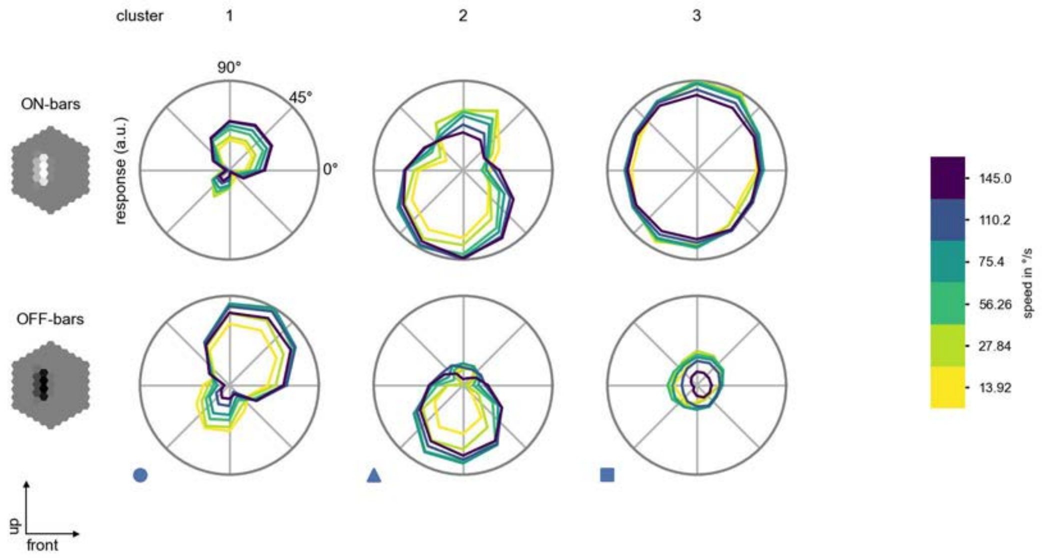

T4c - Figure 10: **Peak responses to moving bars from task-optimal models.** The top row shows peak responses to moving ON-bars, the bottom row shows peak responses to moving OFF-bars of varying speeds from 13.92°/s to 145°/s (yellow to dark blue). The bar-stimuli move in different directions from 0 to 360 degrees and at different speeds. Responses from the task-optimal model in the respective cluster.

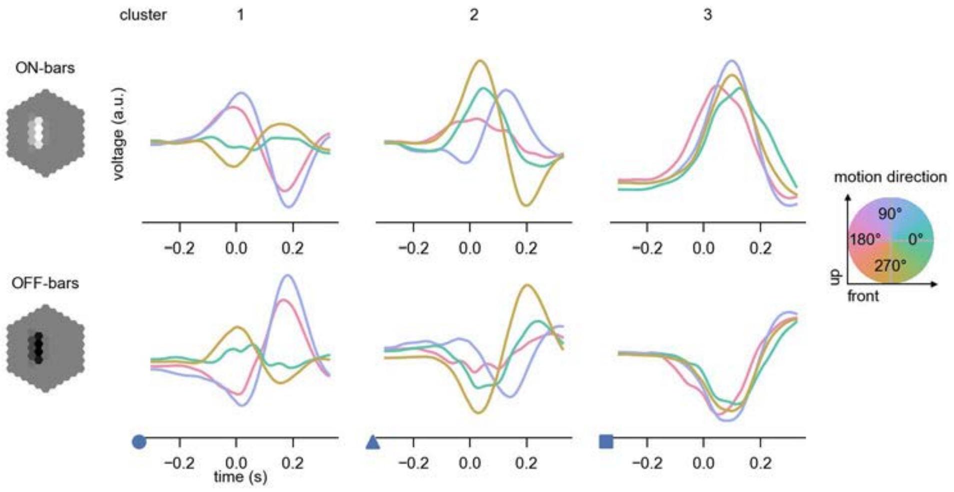

T4c - Figure 11: **Responses to moving bars from task-optimal models.** Responses to moving ON-bars (top row) and to moving OFF-bars (bottom row). Bars move in different directions from 0 to 360 degrees and at different speeds. Responses are from the task-optimal model in the respective cluster. Bars moving at 75.4°/s in all cardinal directions (green 0°, blue 90°, red 180°, yellow 270°) from -22.5 to 22.5° visual angle.

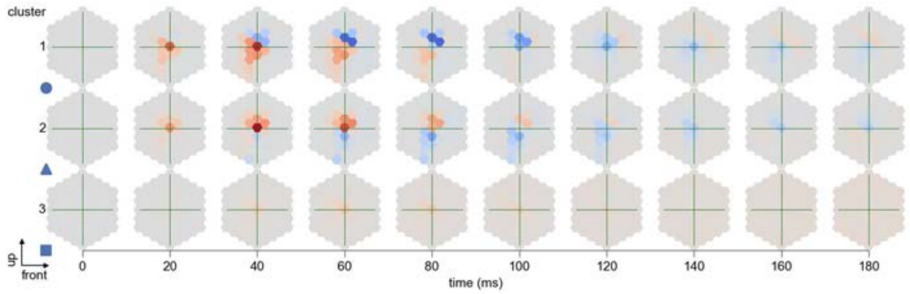

T4c - Figure 12: **Spatio-temporal receptive field.** Responses of the central cell to ON-impulses (5 ms) at single-ommatidium flash locations. The flash occurs at second zero. Responses from the task-optimal model of the respective cluster (rows). Red indicates depolarization, blue indicates hyperpolarization.

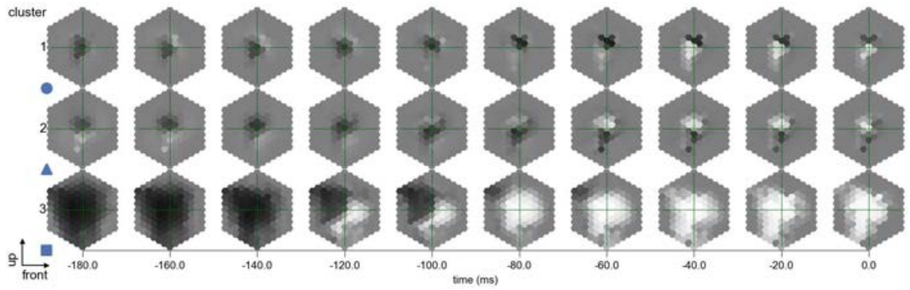

T4c - Figure 13: **Maximally excitatory stimuli.** Each row presents the regularized naturalistic-stimulus from the Sintel dataset that maximizes the cell type's central column response at second zero in the task-optimal model of the respective cluster (rows).

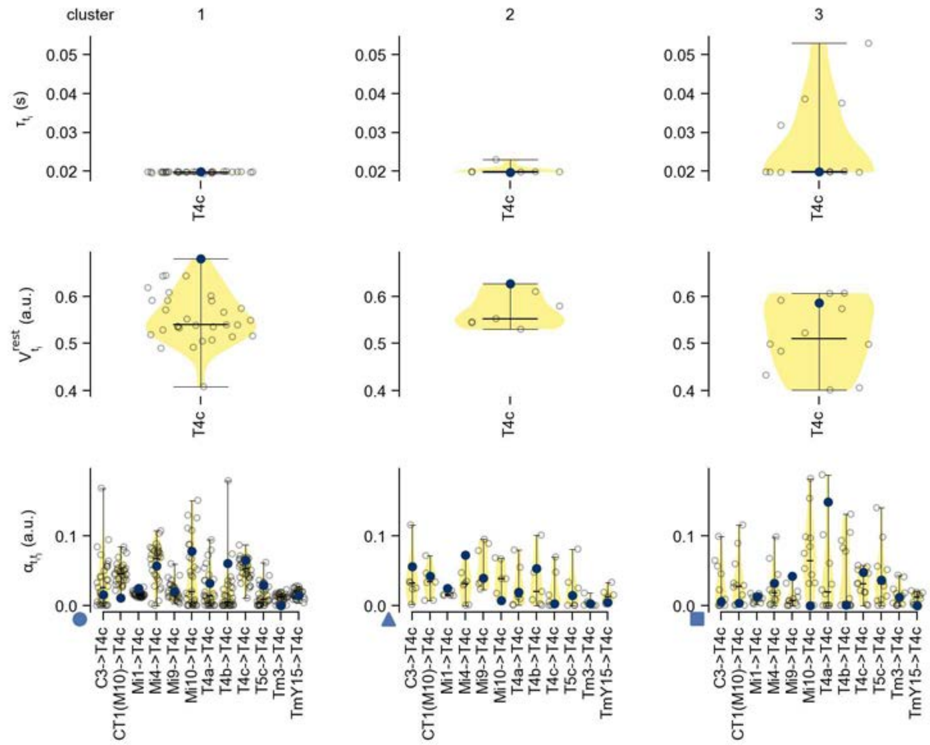

T4c - Figure 14: **Task-constrained parameters.** Each column shows the parameters inferred within the respective cluster. First row: learned time constants of the cell type. Second row: resting potentials of the cell type. Third row: scaling factors for the convolutional filters. The blue scatter represents the parameters from the task-optimal model within the cluster.

39 T4d

← Cell types

Figures

1 Anatomical receptive fields. 274
2 Anatomical projective fields. 275
3 Clustering of the responses to naturalistic stimuli. 275
4 Responses to flashes. 276
5 Cluster-average responses to single-ommatidium flashes. 276
6 Peak responses to moving edges. 277
7 Peak responses to moving edges from task-optimal models. 277
8 Responses to moving edges from task-optimal models. 278
9 Peak responses to moving bars. 278
10 Peak responses to moving bars from task-optimal models. 279
11 Responses to moving bars from task-optimal models. 279
12 Spatio-temporal receptive field. 280
13 Maximally excitatory stimuli. 280
14 Task-constrained parameters. 281

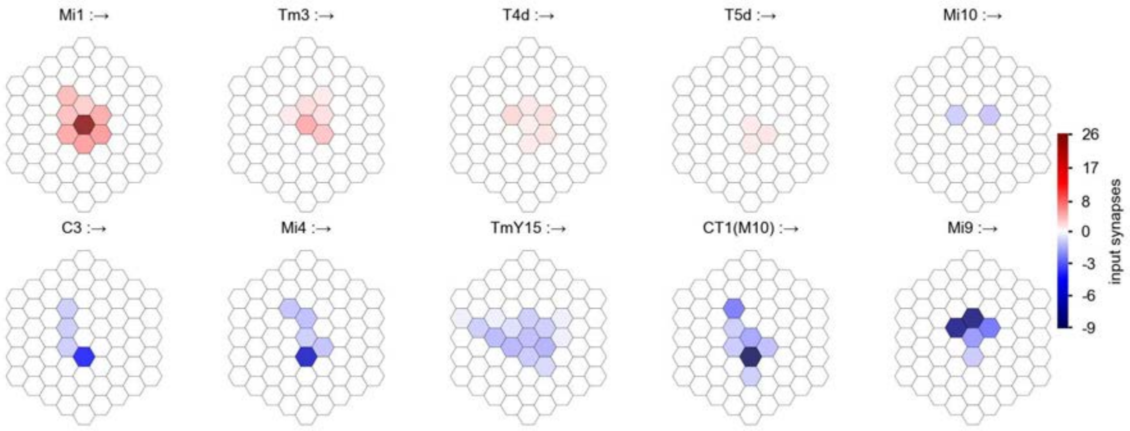

T4d - Figure 1: **Anatomical receptive fields.** Each colored hexagon is an input connection, with the connection strength characterized by the average number of synapses that we count from the EM reconstruction. Red indicates excitatory synapses, blue indicates inhibitory synapses from inferred signs. Filters in the order of their total number of synapses.

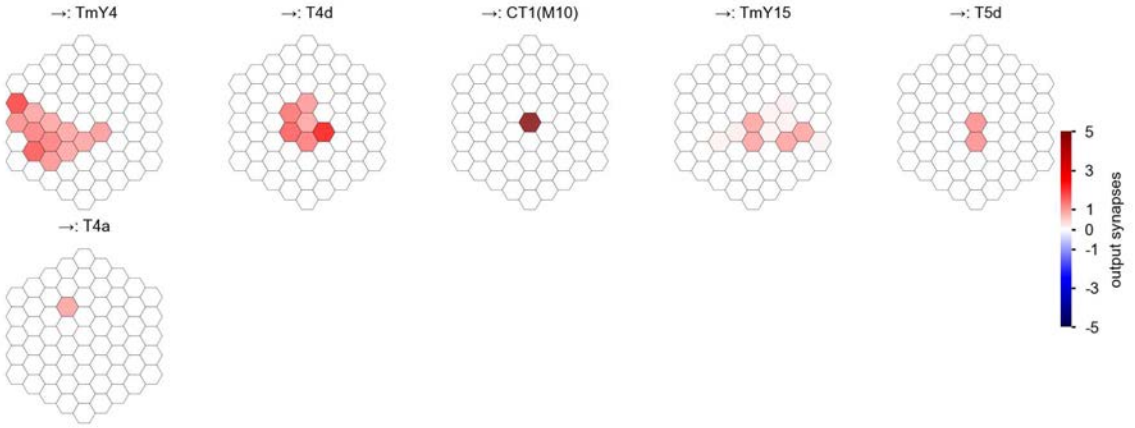

T4d - Figure 2: **Anatomical projective fields.** Each colored hexagon is an output connection, with the connection strength characterized by the average number of synapses that we count from the EM reconstruction. Red indicates excitatory synapses, blue indicates inhibitory synapses from inferred signs. Filters in the order of their total number of synapses.

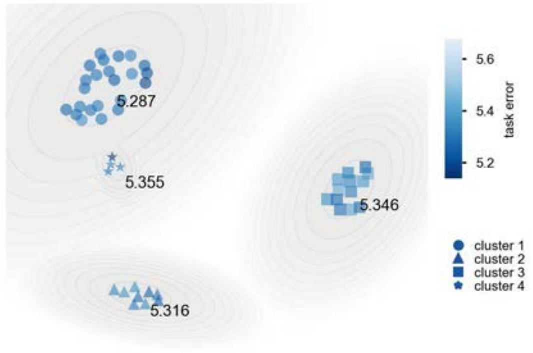

T4d - Figure 3: **Clustering of the responses to naturalistic stimuli.** Clustering of the 50 models based on the cell type responses to naturalistic scenes from the Sintel dataset. Scatterpoints represent individual models colored by their task error.

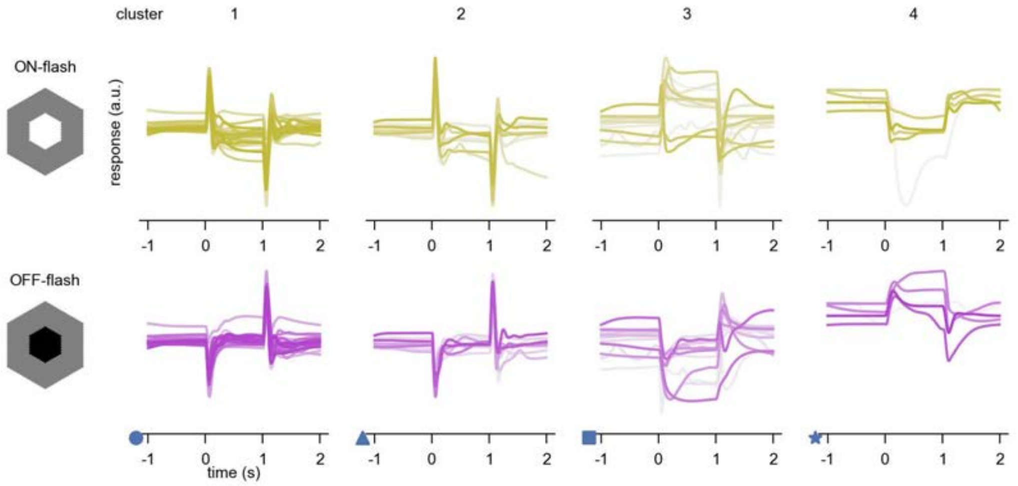

T4d - Figure 4: **Responses to flashes.** The top row shows responses to ON-flashes (yellow), the bottom row shows responses to OFF-flashes (magenta). The responses from the 50 different models that are separated into the different clusters (columns) overlay, with better task-performing models on top. Responses from better task-performing models are more saturated. The circular flashes (1s) cover 6 ommatidia in radius and are presented at time zero. Before and after, a grey-stimulus leads to a stationary state of the network.

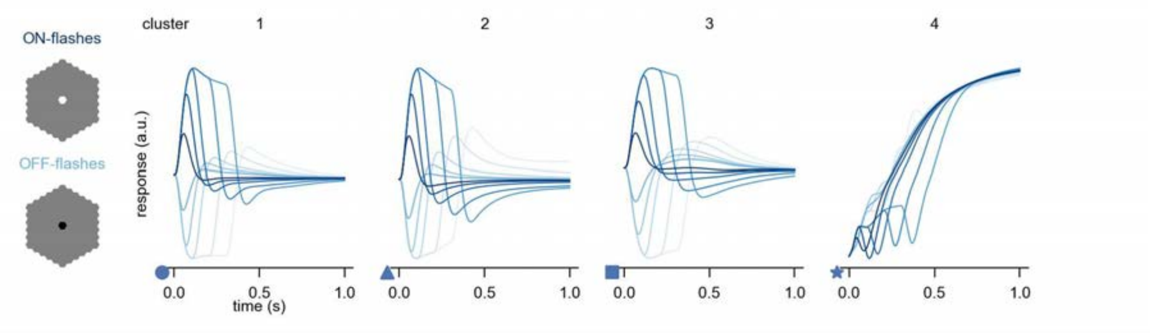

T4d - Figure 5: **Cluster-average responses to single-ommatidium flashes.** Responses to single-ommatidium ON-flashes (dark blue shades) and single-ommatidium OFF-flashes (light blue shades) of 20ms, 50ms, 100ms, 200ms, 300ms duration. The flashes occur at second zero.

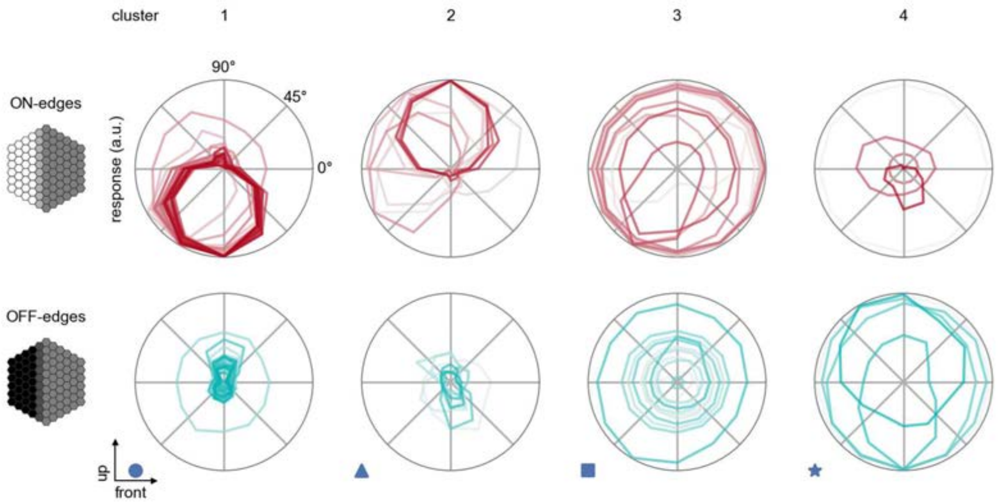

T4d - Figure 6: **Peak responses to moving edges.** The top row shows peak responses to moving ON-edges (red), the bottom row shows peak responses to moving OFF-edges (turqoise). The peak responses are averaged over edge-speeds. Edge-stimuli move in different directions from 0 to 360 degrees. The responses from the different models in the different clusters (columns) overlay. Responses from better task-performing models are more saturated.

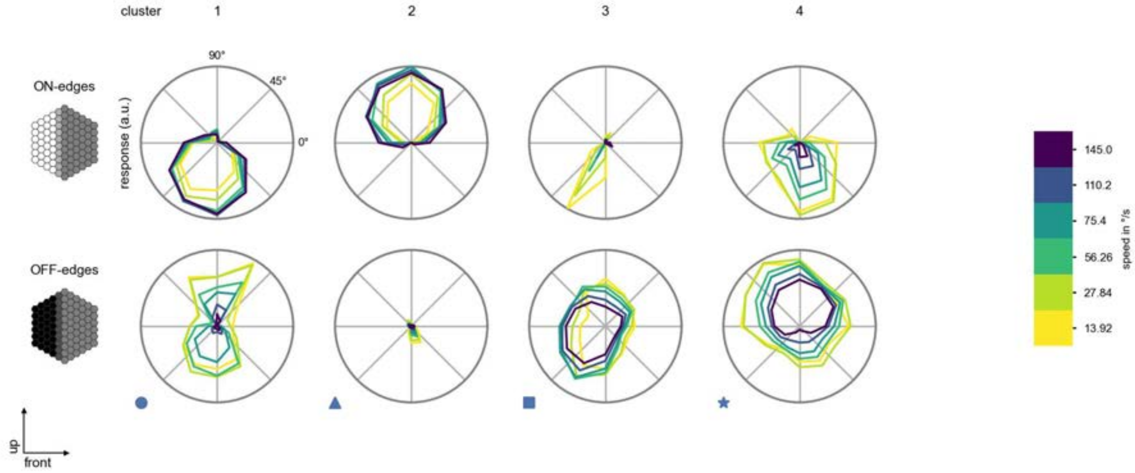

T4d - Figure 7: **Peak responses to moving edges from task-optimal models.** The top row shows peak responses to moving ON-edges, the bottom row shows peak responses to moving OFF-edges of varying speeds from 13.92°/s to 145°/s (yellow to dark blue). The edge-stimuli move in different directions from 0 to 360 degrees and at different speeds. Responses from the task-optimal model in the respective cluster.

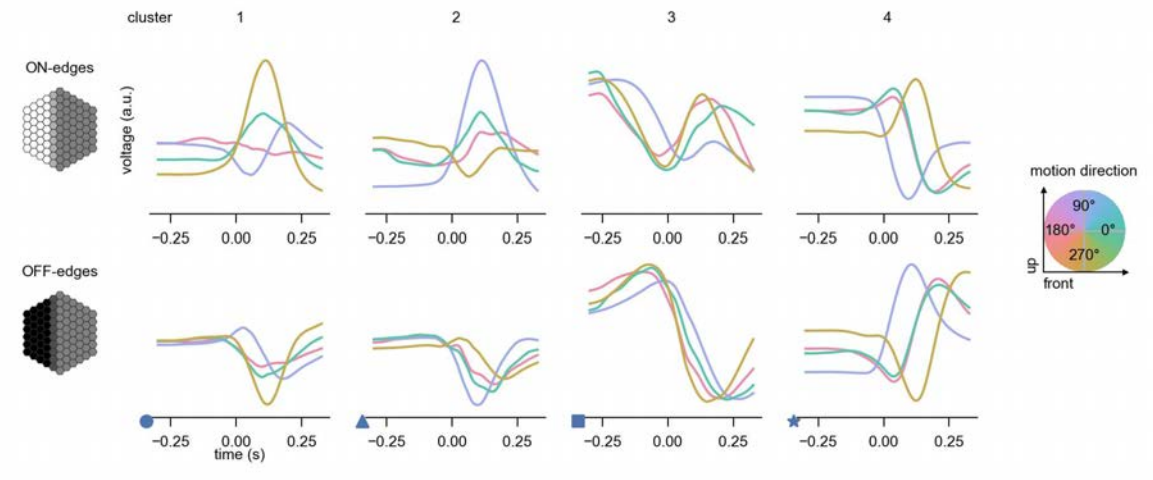

T4d - Figure 8: **Responses to moving edges from task-optimal models.** Responses to moving ON-edges (top row) and to moving OFF-edges (bottom row). Edges move in different directions from 0 to 360 degrees and at different speeds. Responses are from the task-optimal model in the respective cluster. Edges moving at 75.4°/s in all cardinal directions (green 0°, blue 90°, red 180°, yellow 270°) from -22.5 to 22.5° visual angle.

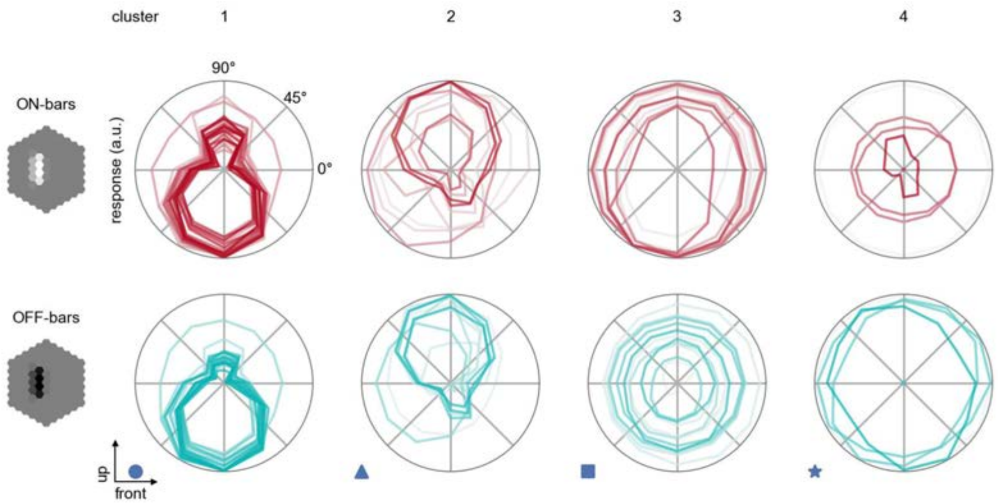

T4d - Figure 9: **Peak responses to moving bars.** The top row shows peak responses to moving ON-bars (red), the bottom row shows peak responses to moving OFF-bars (turqoise). The peak responses are averaged over bar-speeds. Bar-stimuli move in different directions from 0 to 360 degrees. The responses from the different models in the different clusters (columns) overlay. Responses from better task-performing models are more saturated.

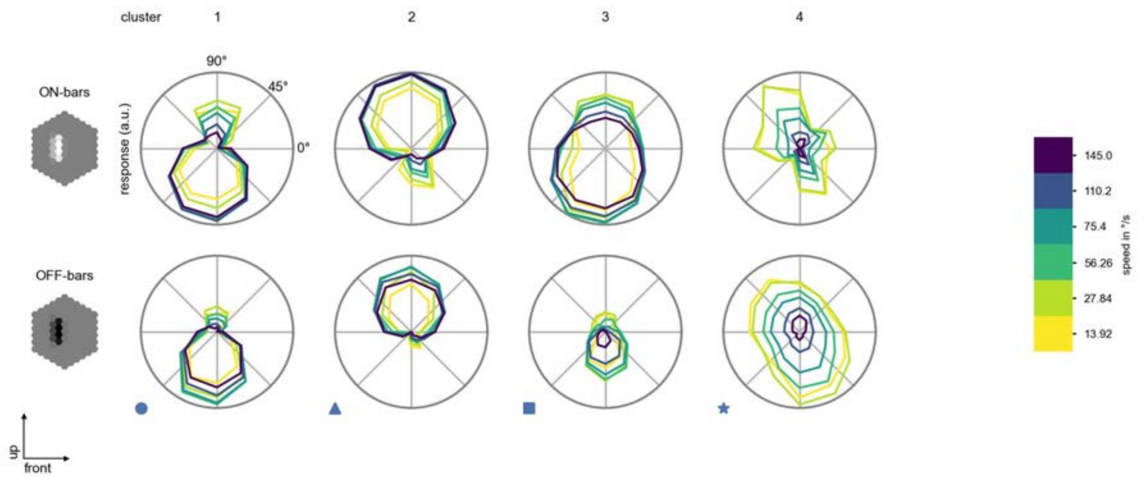

T4d - Figure 10: **Peak responses to moving bars from task-optimal models.** The top row shows peak responses to moving ON-bars, the bottom row shows peak responses to moving OFF-bars of varying speeds from 13.92°/s to 145°/s (yellow to dark blue). The bar-stimuli move in different directions from 0 to 360 degrees and at different speeds. Responses from the task-optimal model in the respective cluster.

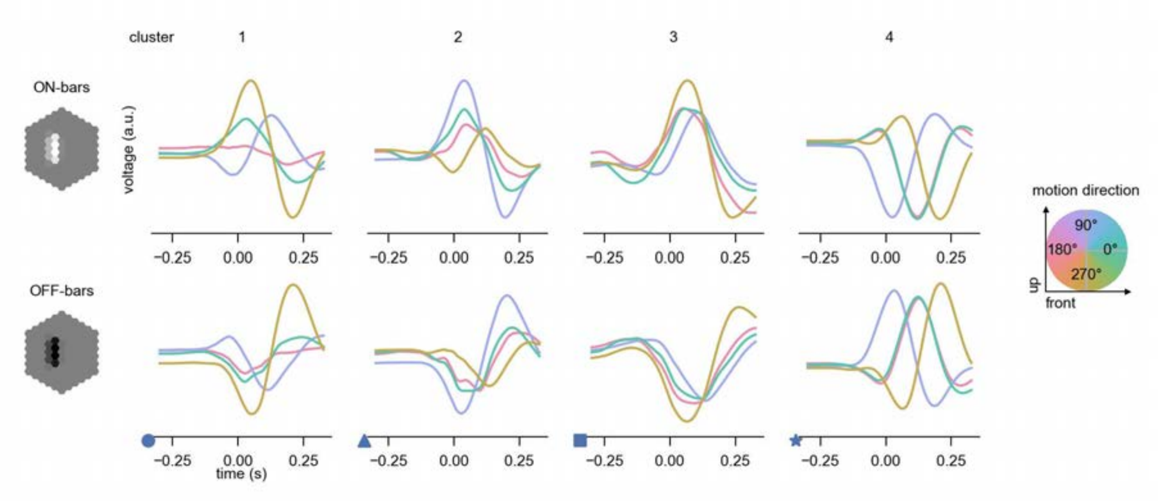

T4d - Figure 11: **Responses to moving bars from task-optimal models.** Responses to moving ON-bars (top row) and to moving OFF-bars (bottom row). Bars move in different directions from 0 to 360 degrees and at different speeds. Responses are from the task-optimal model in the respective cluster. Bars moving at 75.4°/s in all cardinal directions (green 0°, blue 90°, red 180°, yellow 270°) from -22.5 to 22.5° visual angle.

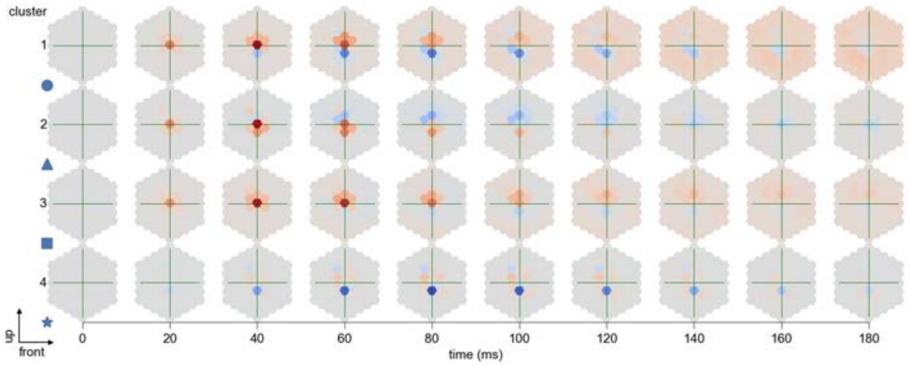

T4d - Figure 12: **Spatio-temporal receptive field.** Responses of the central cell to ON-impulses (5 ms) at single-ommatidium flash locations. The flash occurs at second zero. Responses from the task-optimal model of the respective cluster (rows). Red indicates depolarization, blue indicates hyperpolarization.

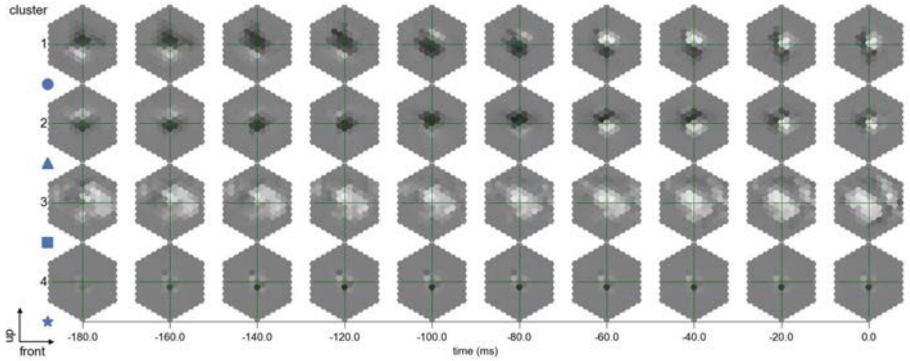

T4d - Figure 13: **Maximally excitatory stimuli.** Each row presents the regularized naturalistic-stimulus from the Sintel dataset that maximizes the cell type's central column response at second zero in the task-optimal model of the respective cluster (rows).

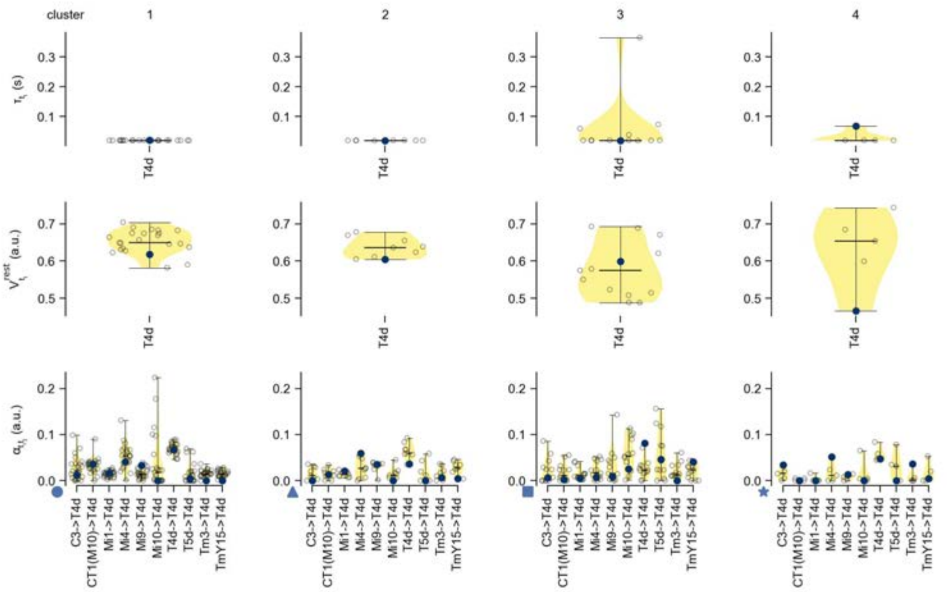

T4d - Figure 14: **Task-constrained parameters.** Each column shows the parameters inferred within the respective cluster. First row: learned time constants of the cell type. Second row: resting potentials of the cell type. Third row: scaling factors for the convolutional filters. The blue scatter represents the parameters from the task-optimal model within the cluster.

40 T5a

← Cell types

Figures

1 Anatomical receptive fields. . . . 282
2 Anatomical projective fields. . . . 283
3 Clustering of the responses to naturalistic stimuli. . . . 283
4 Responses to flashes. . . . 284
5 Cluster-average responses to single-ommatidium flashes. . . . 284
6 Peak responses to moving edges. . . . 285
7 Peak responses to moving edges from task-optimal models. . . . 285
8 Responses to moving edges from task-optimal models. . . . 286
9 Peak responses to moving bars. . . . 286
10 Peak responses to moving bars from task-optimal models. . . . 287
11 Responses to moving bars from task-optimal models. . . . 287
12 Spatio-temporal receptive field. . . . 288
13 Maximally excitatory stimuli. . . . 288
14 Task-constrained parameters. . . . 289

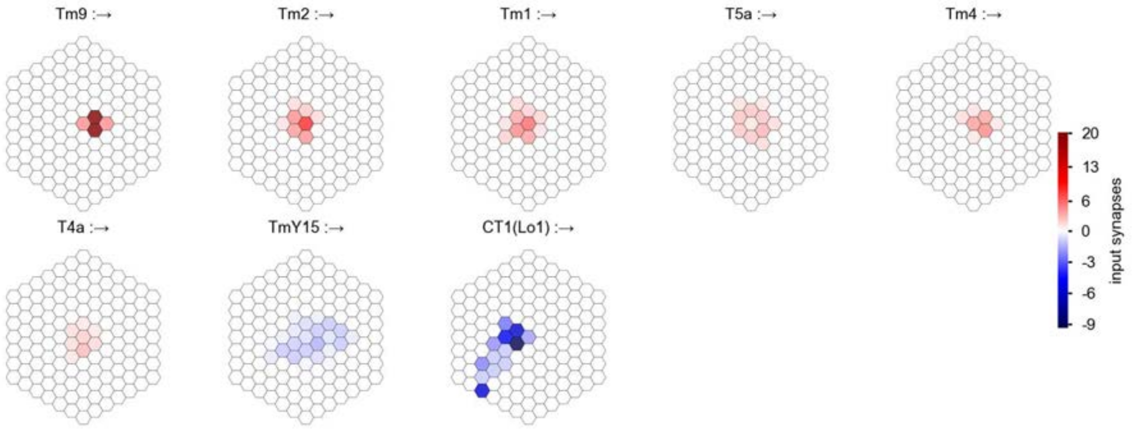

T5a - Figure 1: **Anatomical receptive fields.** Each colored hexagon is an input connection, with the connection strength characterized by the average number of synapses that we count from the EM reconstruction. Red indicates excitatory synapses, blue indicates inhibitory synapses from inferred signs. Filters in the order of their total number of synapses.

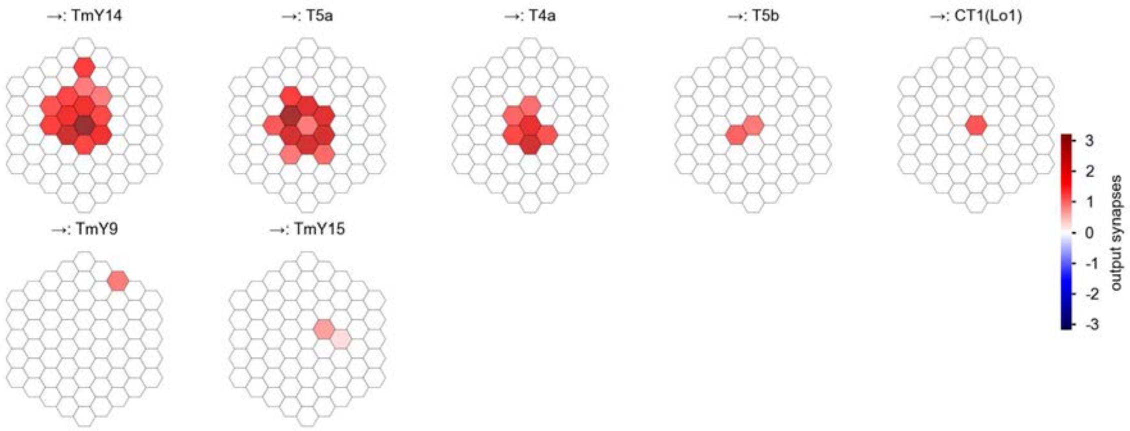

T5a - Figure 2: **Anatomical projective fields.** Each colored hexagon is an output connection, with the connection strength characterized by the average number of synapses that we count from the EM reconstruction. Red indicates excitatory synapses, blue indicates inhibitory synapses from inferred signs. Filters in the order of their total number of synapses.

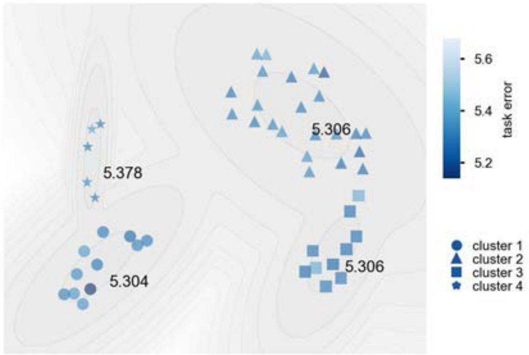

T5a - Figure 3: **Clustering of the responses to naturalistic stimuli.** Clustering of the 50 models based on the cell type responses to naturalistic scenes from the Sintel dataset. Scatterpoints represent individual models colored by their task error.

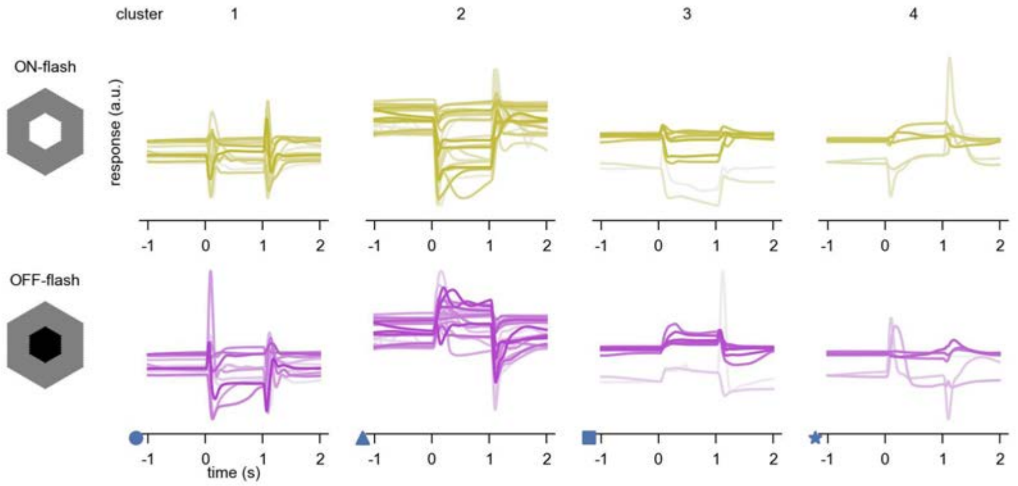

T5a - Figure 4: **Responses to flashes.** The top row shows responses to ON-flashes (yellow), the bottom row shows responses to OFF-flashes (magenta). The responses from the 50 different models that are separated into the different clusters (columns) overlay, with better task-performing models on top. Responses from better task-performing models are more saturated. The circular flashes (1s) cover 6 ommatidia in radius and are presented at time zero. Before and after, a grey-stimulus leads to a stationary state of the network.

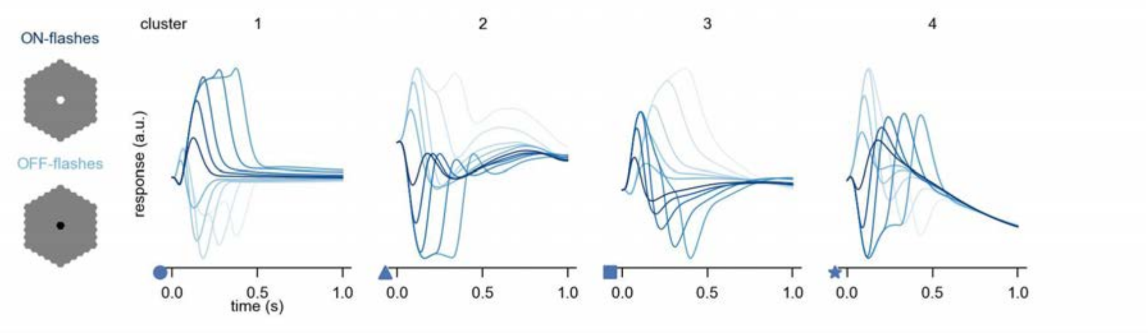

T5a - Figure 5: **Cluster-average responses to single-ommatidium flashes.** Responses to single-ommatidium ON-flashes (dark blue shades) and single-ommatidium OFF-flashes (light blue shades) of 20ms, 50ms, 100ms, 200ms, 300ms duration. The flashes occur at second zero.

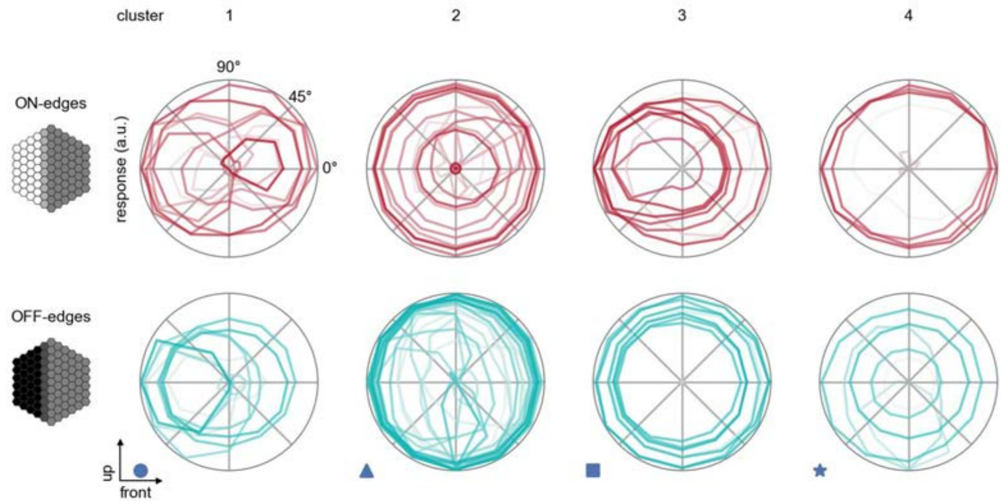

T5a - Figure 6: **Peak responses to moving edges.** The top row shows peak responses to moving ON-edges (red), the bottom row shows peak responses to moving OFF-edges (turqoise). The peak responses are averaged over edge-speeds. Edge-stimuli move in different directions from 0 to 360 degrees. The responses from the different models in the different clusters (columns) overlay. Responses from better task-performing models are more saturated.

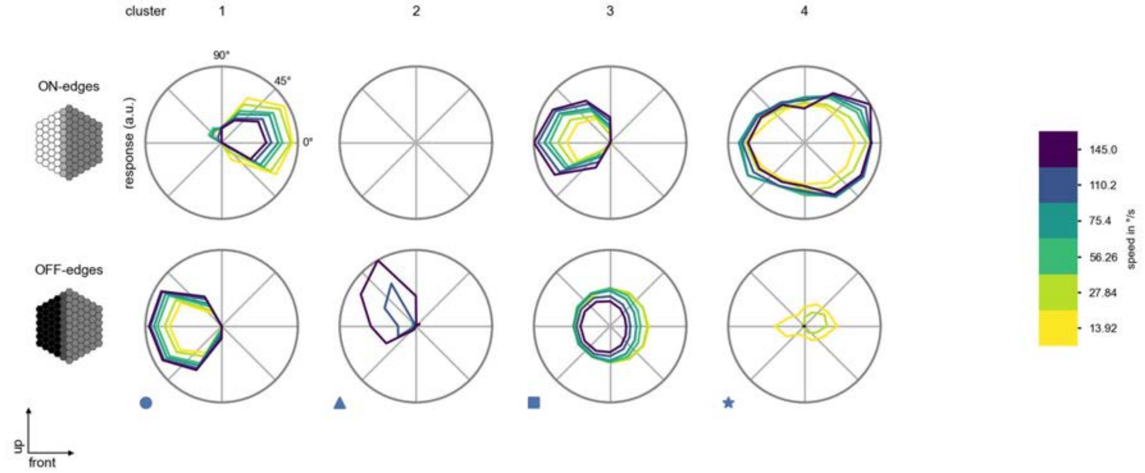

T5a - Figure 7: **Peak responses to moving edges from task-optimal models.** The top row shows peak responses to moving ON-edges, the bottom row shows peak responses to moving OFF-edges of varying speeds from 13.92°/s to 145°/s (yellow to dark blue). The edge-stimuli move in different directions from 0 to 360 degrees and at different speeds. Responses from the task-optimal model in the respective cluster.

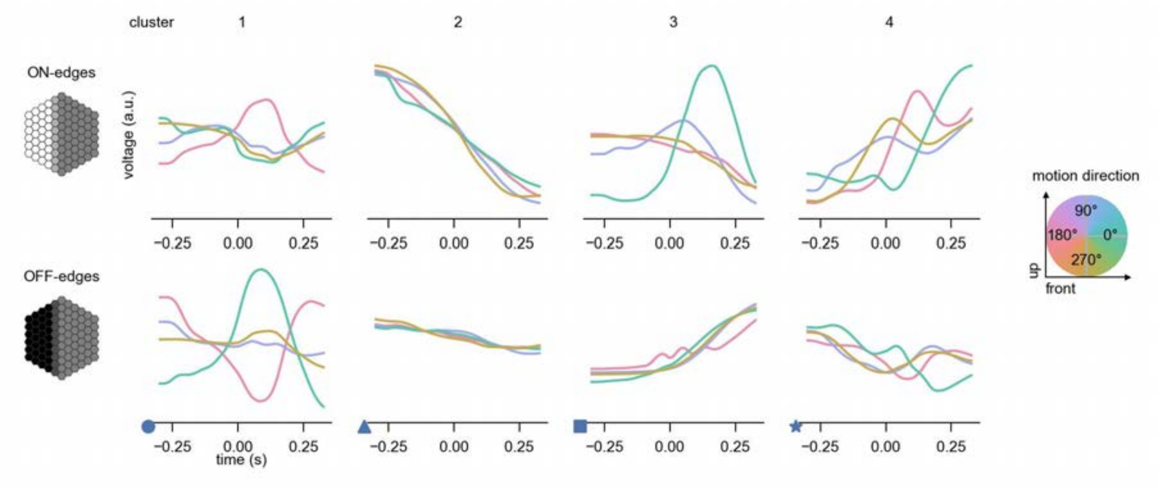

T5a - Figure 8: **Responses to moving edges from task-optimal models.** Responses to moving ON-edges (top row) and to moving OFF-edges (bottom row). Edges move in different directions from 0 to 360 degrees and at different speeds. Responses are from the task-optimal model in the respective cluster. Edges moving at 75.4°/s in all cardinal directions (green 0°, blue 90°, red 180°, yellow 270°) from -22.5 to 22.5° visual angle.

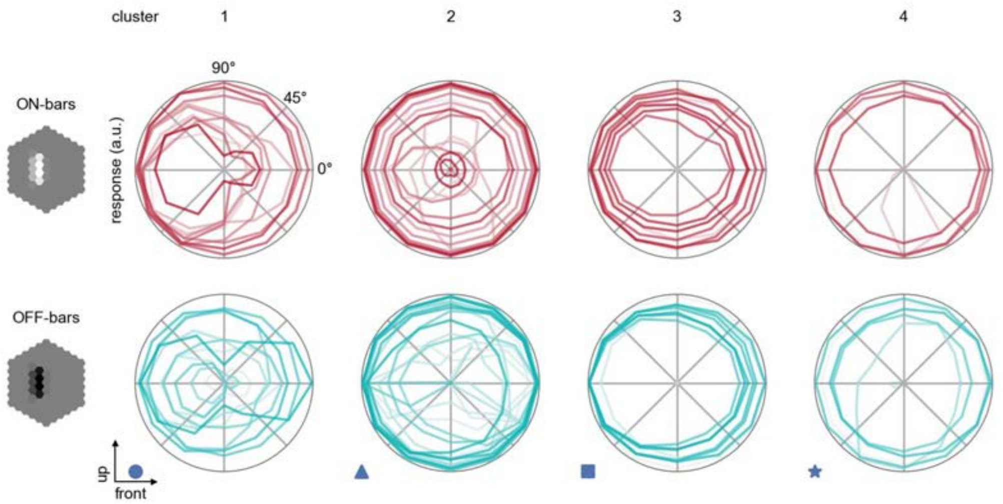

T5a - Figure 9: **Peak responses to moving bars.** The top row shows peak responses to moving ON-bars (red), the bottom row shows peak responses to moving OFF-bars (turqoise). The peak responses are averaged over bar-speeds. Bar-stimuli move in different directions from 0 to 360 degrees. The responses from the different models in the different clusters (columns) overlay. Responses from better task-performing models are more saturated.

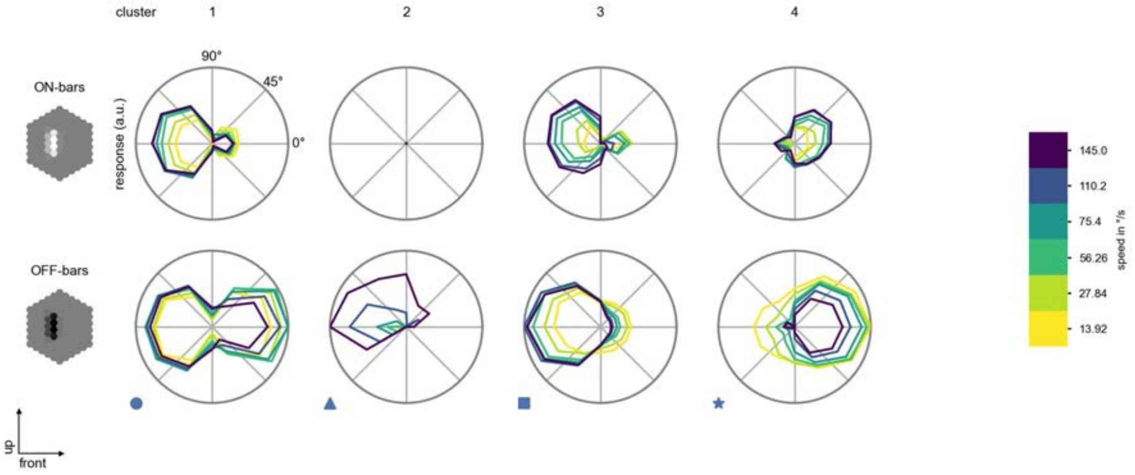

T5a - Figure 10: **Peak responses to moving bars from task-optimal models.** The top row shows peak responses to moving ON-bars, the bottom row shows peak responses to moving OFF-bars of varying speeds from 13.92°/s to 145°/s (yellow to dark blue). The bar-stimuli move in different directions from 0 to 360 degrees and at different speeds. Responses from the task-optimal model in the respective cluster.

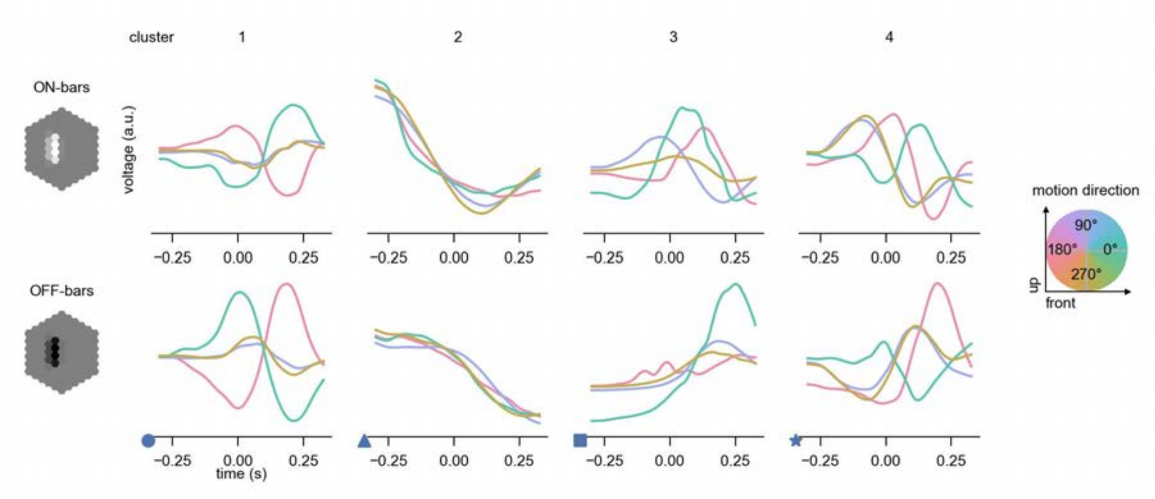

T5a - Figure 11: **Responses to moving bars from task-optimal models.** Responses to moving ON-bars (top row) and to moving OFF-bars (bottom row). Bars move in different directions from 0 to 360 degrees and at different speeds. Responses are from the task-optimal model in the respective cluster. Bars moving at 75.4°/s in all cardinal directions (green 0°, blue 90°, red 180°, yellow 270°) from -22.5 to 22.5° visual angle.

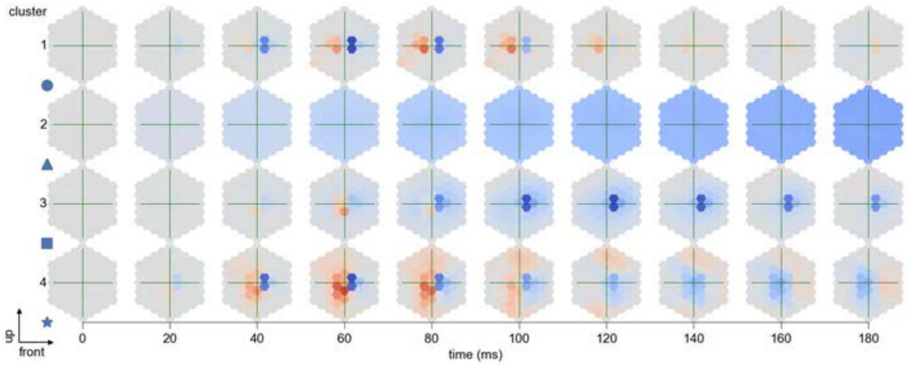

T5a - Figure 12: **Spatio-temporal receptive field.** Responses of the central cell to ON-impulses (5 ms) at single-ommatidium flash locations. The flash occurs at second zero. Responses from the task-optimal model of the respective cluster (rows). Red indicates depolarization, blue indicates hyperpolarization.

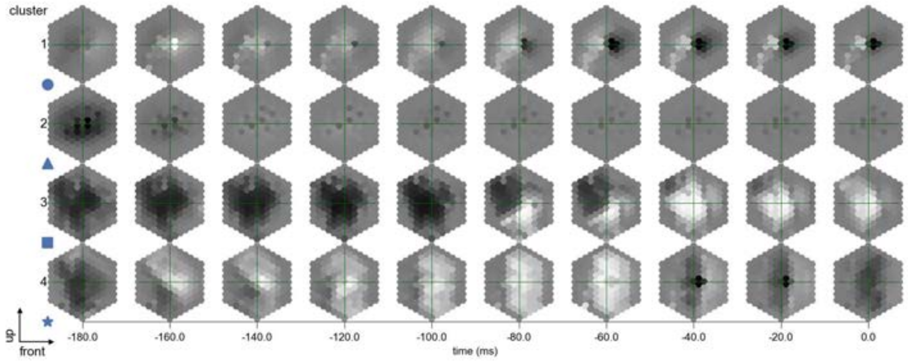

T5a - Figure 13: **Maximally excitatory stimuli.** Each row presents the regularized naturalistic-stimulus from the Sintel dataset that maximizes the cell type's central column response at second zero in the task-optimal model of the respective cluster (rows).

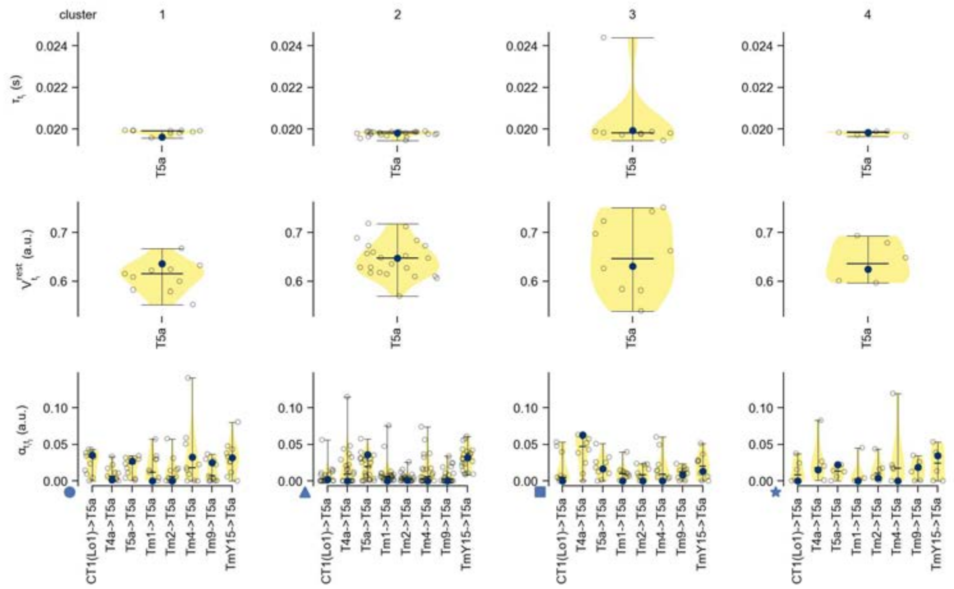

T5a - Figure 14: **Task-constrained parameters.** Each column shows the parameters inferred within the respective cluster. First row: learned time constants of the cell type. Second row: resting potentials of the cell type. Third row: scaling factors for the convolutional filters. The blue scatter represents the parameters from the task-optimal model within the cluster.

41 T5b

← Cell types

Figures

1 Anatomical receptive fields. . . . 290
2 Anatomical projective fields. . . . 291
3 Clustering of the responses to naturalistic stimuli. . . . 291
4 Responses to flashes. . . . 292
5 Cluster-average responses to single-ommatidium flashes. . . . 292
6 Peak responses to moving edges. . . . 293
7 Peak responses to moving edges from task-optimal models. . . . 293
8 Responses to moving edges from task-optimal models. . . . 294
9 Peak responses to moving bars. . . . 294
10 Peak responses to moving bars from task-optimal models. . . . 295
11 Responses to moving bars from task-optimal models. . . . 295
12 Spatio-temporal receptive field. . . . 296
13 Maximally excitatory stimuli. . . . 296
14 Task-constrained parameters. . . . 297

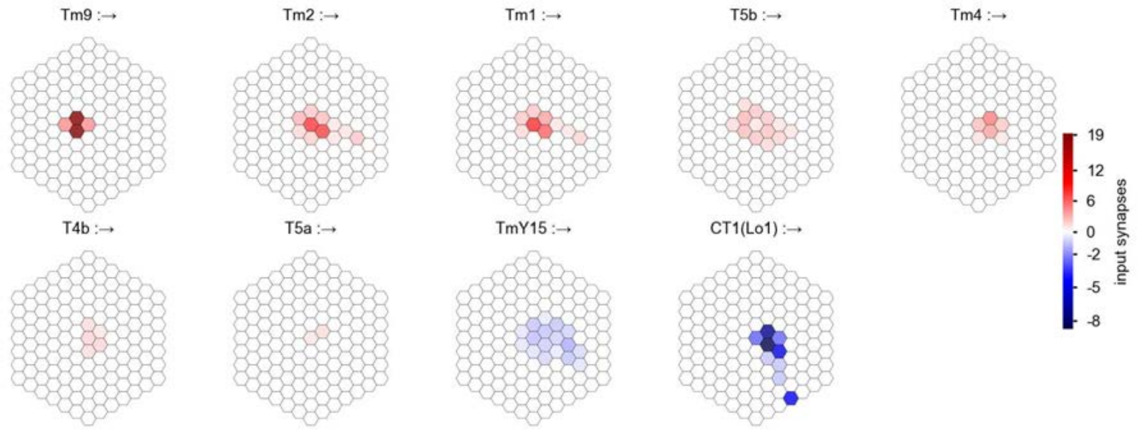

T5b - Figure 1: **Anatomical receptive fields.** Each colored hexagon is an input connection, with the connection strength characterized by the average number of synapses that we count from the EM reconstruction. Red indicates excitatory synapses, blue indicates inhibitory synapses from inferred signs. Filters in the order of their total number of synapses.

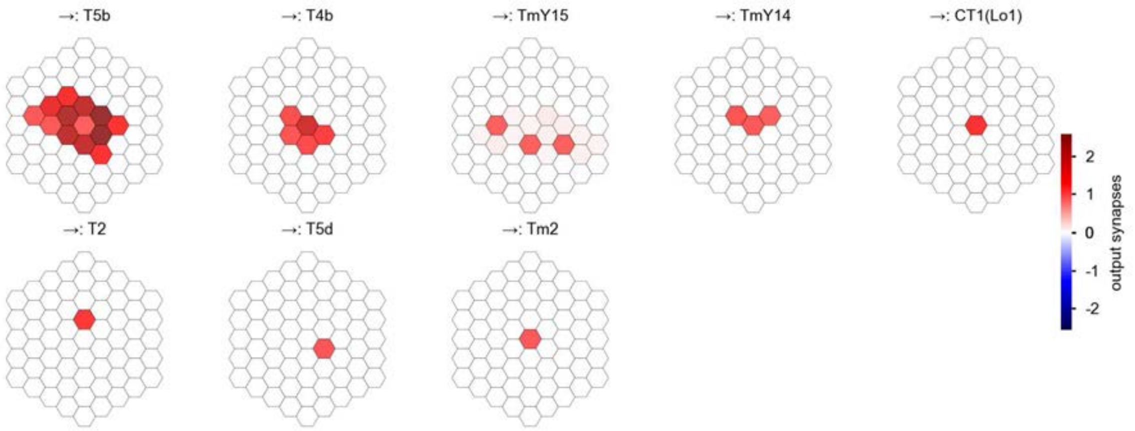

T5b - Figure 2: **Anatomical projective fields.** Each colored hexagon is an output connection, with the connection strength characterized by the average number of synapses that we count from the EM reconstruction. Red indicates excitatory synapses, blue indicates inhibitory synapses from inferred signs. Filters in the order of their total number of synapses.

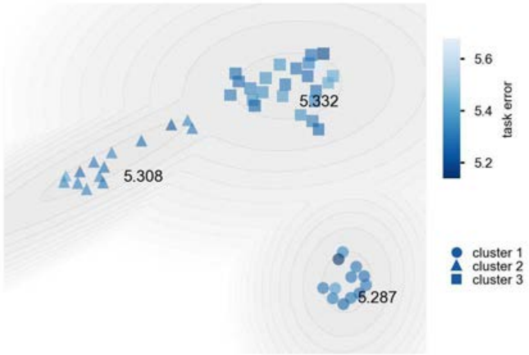

T5b - Figure 3: **Clustering of the responses to naturalistic stimuli.** Clustering of the 50 models based on the cell type responses to naturalistic scenes from the Sintel dataset. Scatterpoints represent individual models colored by their task error.

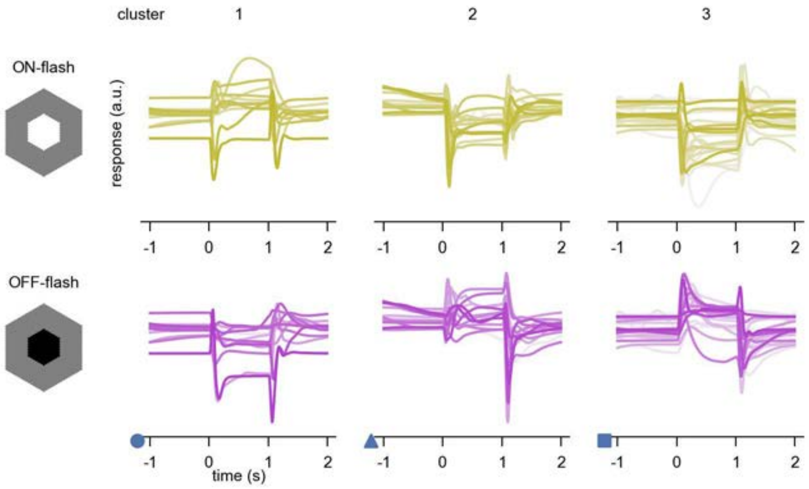

T5b - Figure 4: **Responses to flashes.** The top row shows responses to ON-flashes (yellow), the bottom row shows responses to OFF-flashes (magenta). The responses from the 50 different models that are separated into the different clusters (columns) overlay, with better task-performing models on top. Responses from better task-performing models are more saturated. The circular flashes (1s) cover 6 ommatidia in radius and are presented at time zero. Before and after, a grey-stimulus leads to a stationary state of the network.

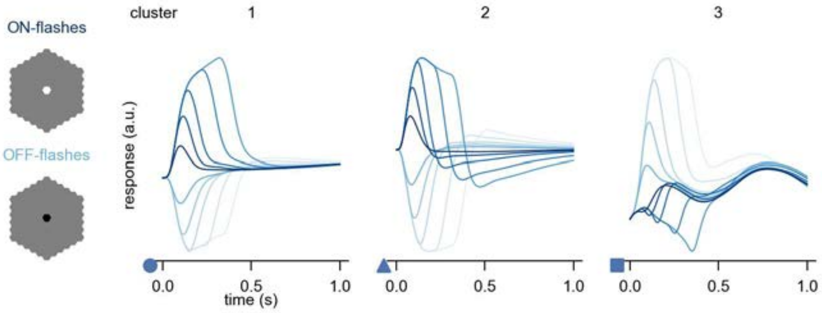

T5b - Figure 5: **Cluster-average responses to single-ommatidium flashes.** Responses to single-ommatidium ON-flashes (dark blue shades) and single-ommatidium OFF-flashes (light blue shades) of 20ms, 50ms, 100ms, 200ms, 300ms duration. The flashes occur at second zero.

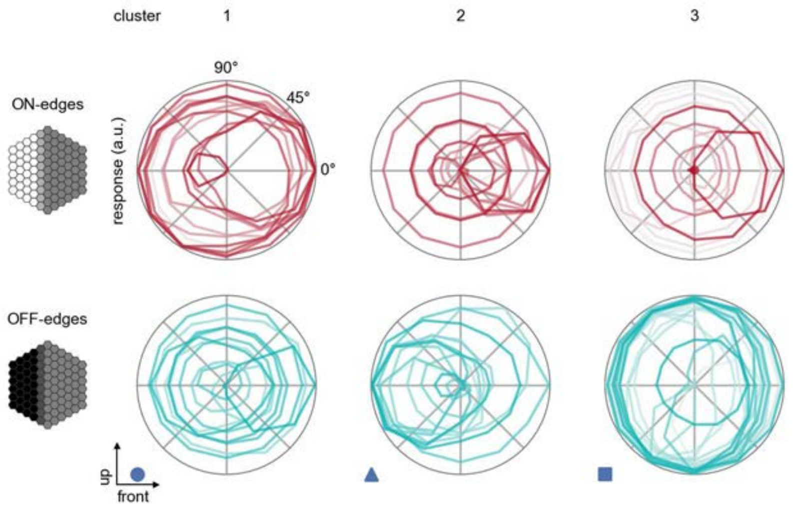

T5b - Figure 6: **Peak responses to moving edges.** The top row shows peak responses to moving ON-edges (red), the bottom row shows peak responses to moving OFF-edges (turqoise). The peak responses are averaged over edge-speeds. Edge-stimuli move in different directions from 0 to 360 degrees. The responses from the different models in the different clusters (columns) overlay. Responses from better task-performing models are more saturated.

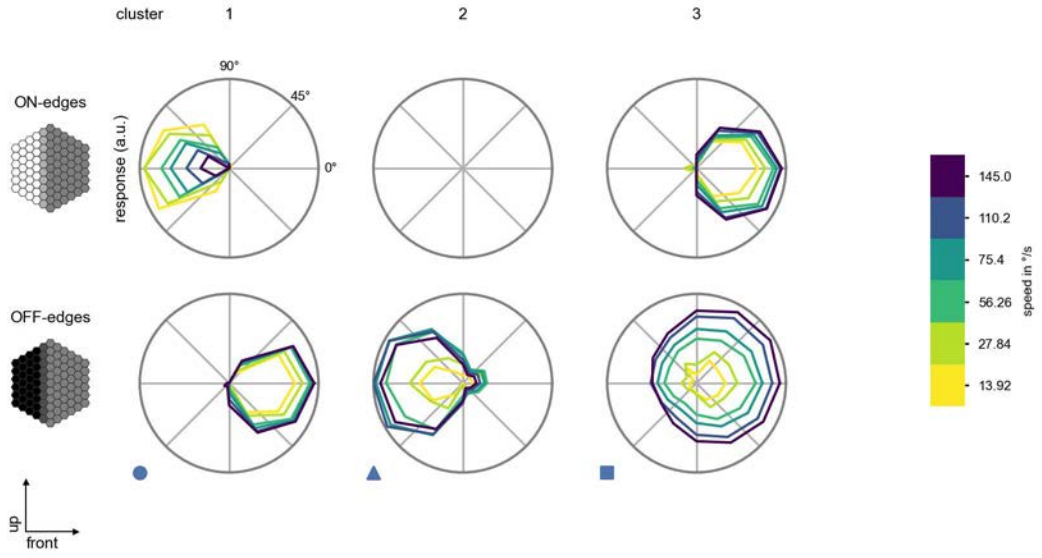

T5b - Figure 7: **Peak responses to moving edges from task-optimal models.** The top row shows peak responses to moving ON-edges, the bottom row shows peak responses to moving OFF-edges of varying speeds from 13.92°/s to 145°/s (yellow to dark blue). The edge-stimuli move in different directions from 0 to 360 degrees and at different speeds. Responses from the task-optimal model in the respective cluster.

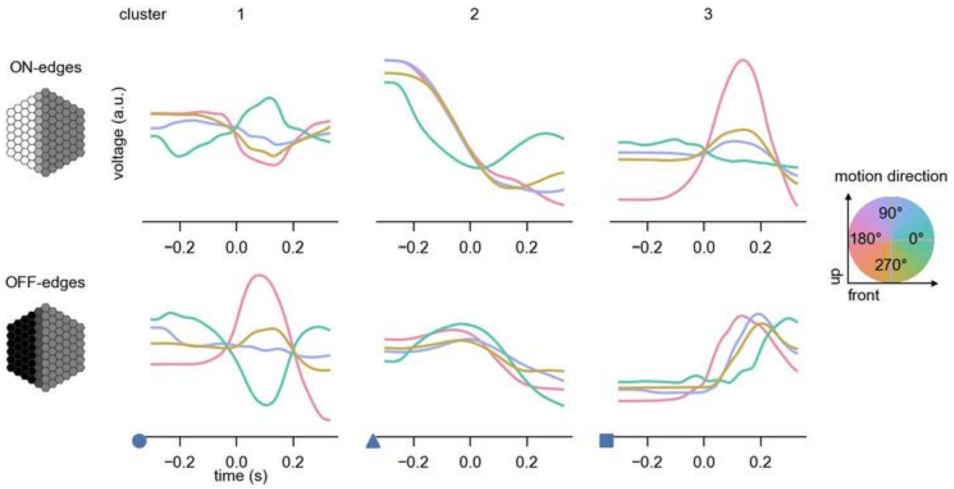

T5b - Figure 8: **Responses to moving edges from task-optimal models.** Responses to moving ON-edges (top row) and to moving OFF-edges (bottom row). Edges move in different directions from 0 to 360 degrees and at different speeds. Responses are from the task-optimal model in the respective cluster. Edges moving at 75.4°/s in all cardinal directions (green 0°, blue 90°, red 180°, yellow 270°) from -22.5 to 22.5° visual angle.

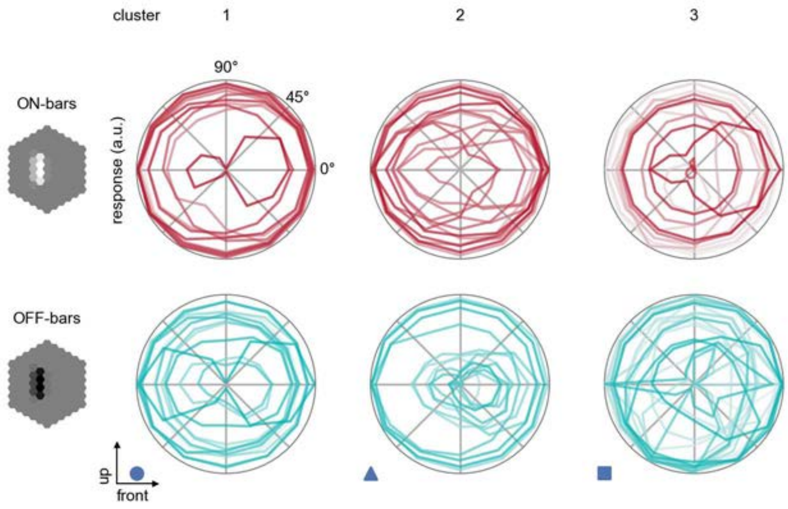

T5b - Figure 9: **Peak responses to moving bars.** The top row shows peak responses to moving ON-bars (red), the bottom row shows peak responses to moving OFF-bars (turqoise). The peak responses are averaged over bar-speeds. Bar-stimuli move in different directions from 0 to 360 degrees. The responses from the different models in the different clusters (columns) overlay. Responses from better task-performing models are more saturated.

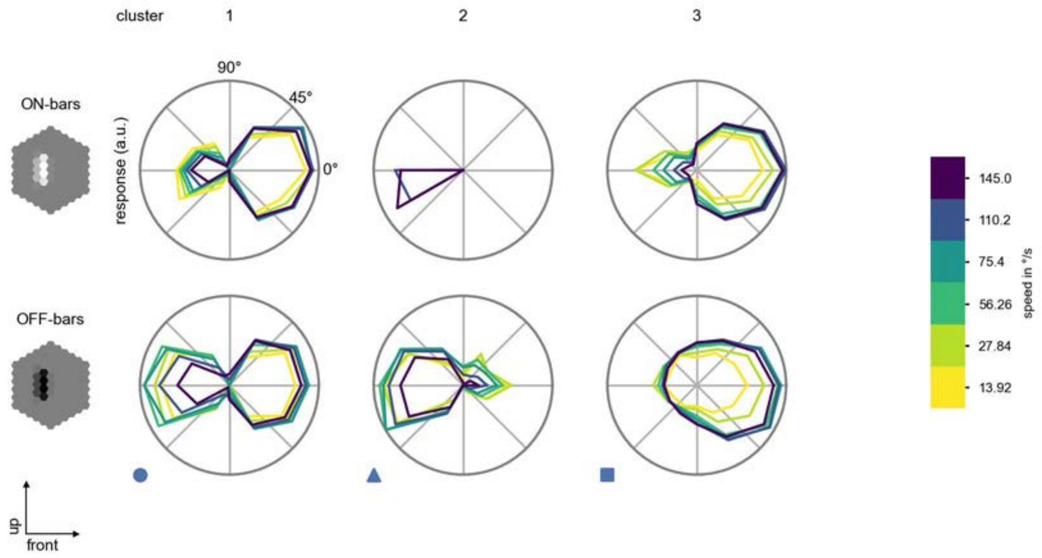

T5b - Figure 10: **Peak responses to moving bars from task-optimal models.** The top row shows peak responses to moving ON-bars, the bottom row shows peak responses to moving OFF-bars of varying speeds from 13.92°/s to 145°/s (yellow to dark blue). The bar-stimuli move in different directions from 0 to 360 degrees and at different speeds. Responses from the task-optimal model in the respective cluster.

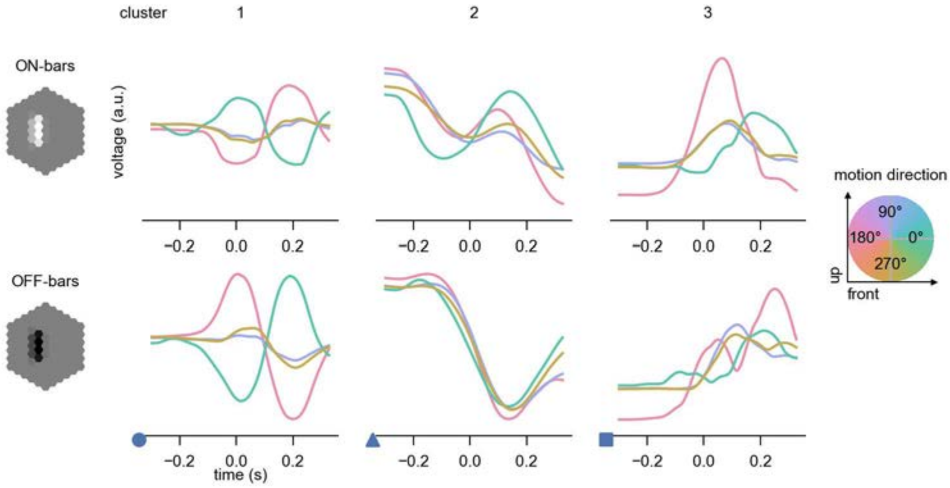

T5b - Figure 11: **Responses to moving bars from task-optimal models.** Responses to moving ON-bars (top row) and to moving OFF-bars (bottom row). Bars move in different directions from 0 to 360 degrees and at different speeds. Responses are from the task-optimal model in the respective cluster. Bars moving at 75.4°/s in all cardinal directions (green 0°, blue 90°, red 180°, yellow 270°) from -22.5 to 22.5° visual angle.

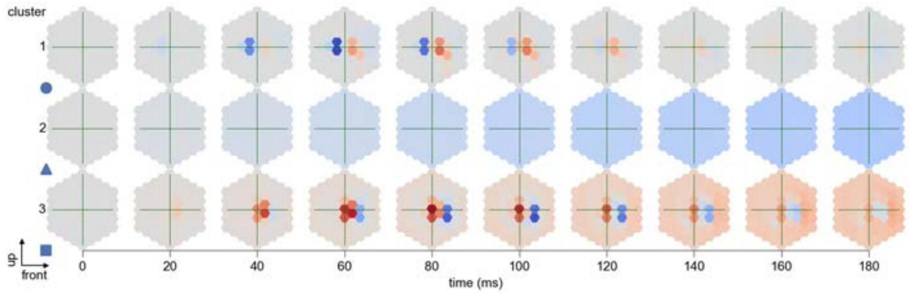

T5b - Figure 12: **Spatio-temporal receptive field.** Responses of the central cell to ON-impulses (5 ms) at single-ommatidium flash locations. The flash occurs at second zero. Responses from the task-optimal model of the respective cluster (rows). Red indicates depolarization, blue indicates hyperpolarization.

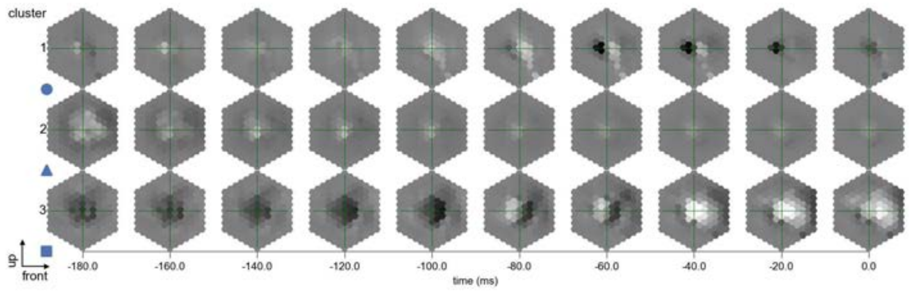

T5b - Figure 13: **Maximally excitatory stimuli.** Each row presents the regularized naturalistic-stimulus from the Sintel dataset that maximizes the cell type's central column response at second zero in the task-optimal model of the respective cluster (rows).

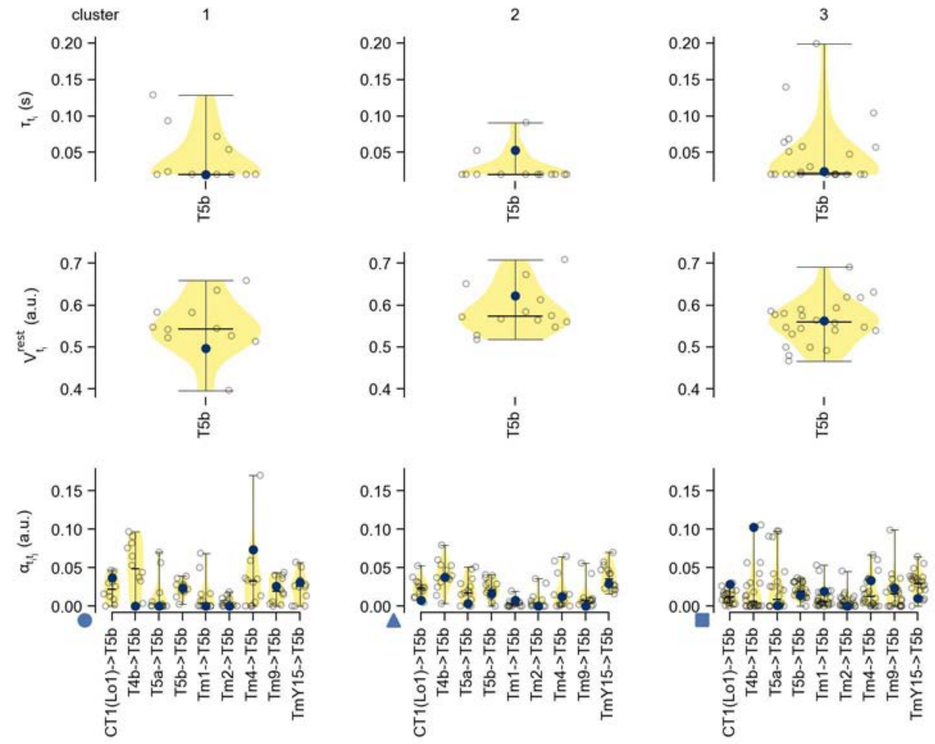

T5b - Figure 14: **Task-constrained parameters.** Each column shows the parameters inferred within the respective cluster. First row: learned time constants of the cell type. Second row: resting potentials of the cell type. Third row: scaling factors for the convolutional filters. The blue scatter represents the parameters from the task-optimal model within the cluster.

42 T5c

← Cell types

Figures

1 Anatomical receptive fields. . . . 298
2 Anatomical projective fields. . . . 299
3 Clustering of the responses to naturalistic stimuli. . . . 299
4 Responses to flashes. . . . 300
5 Cluster-average responses to single-ommatidium flashes. . . . 300
6 Peak responses to moving edges. . . . 301
7 Peak responses to moving edges from task-optimal models. . . . 301
8 Responses to moving edges from task-optimal models. . . . 302
9 Peak responses to moving bars. . . . 302
10 Peak responses to moving bars from task-optimal models. . . . 303
11 Responses to moving bars from task-optimal models. . . . 303
12 Spatio-temporal receptive field. . . . 304
13 Maximally excitatory stimuli. . . . 304
14 Task-constrained parameters. . . . 305

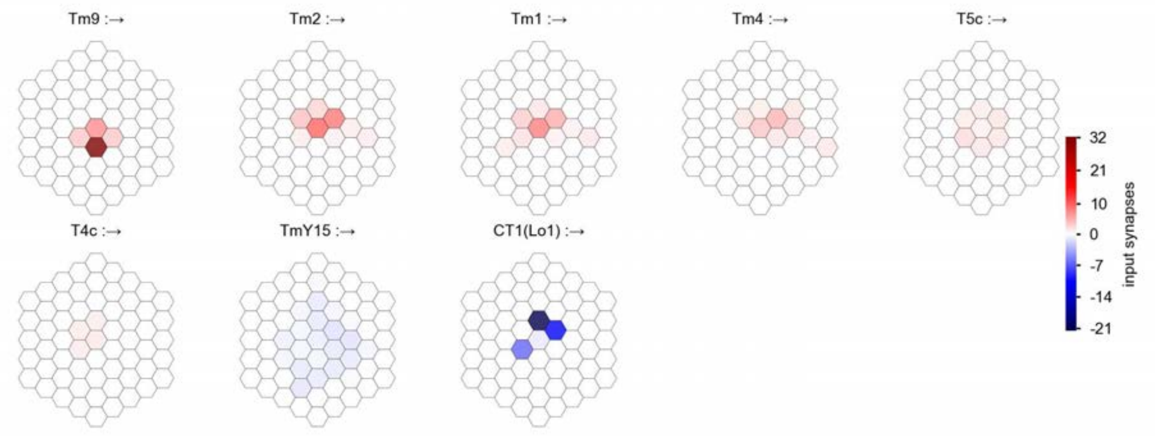

T5c - Figure 1: **Anatomical receptive fields.** Each colored hexagon is an input connection, with the connection strength characterized by the average number of synapses that we count from the EM reconstruction. Red indicates excitatory synapses, blue indicates inhibitory synapses from inferred signs. Filters in the order of their total number of synapses.

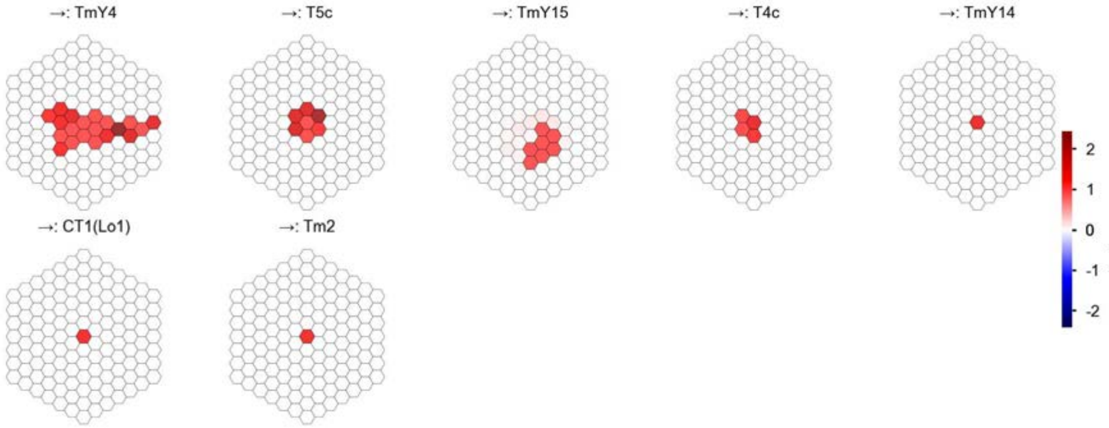

T5c - Figure 2: **Anatomical projective fields.** Each colored hexagon is an output connection, with the connection strength characterized by the average number of synapses that we count from the EM reconstruction. Red indicates excitatory synapses, blue indicates inhibitory synapses from inferred signs. Filters in the order of their total number of synapses.

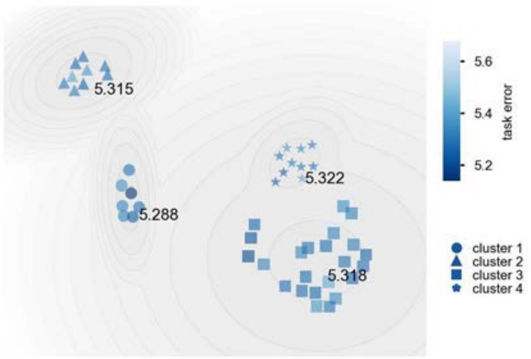

T5c - Figure 3: **Clustering of the responses to naturalistic stimuli.** Clustering of the 50 models based on the cell type responses to naturalistic scenes from the Sintel dataset. Scatterpoints represent individual models colored by their task error.

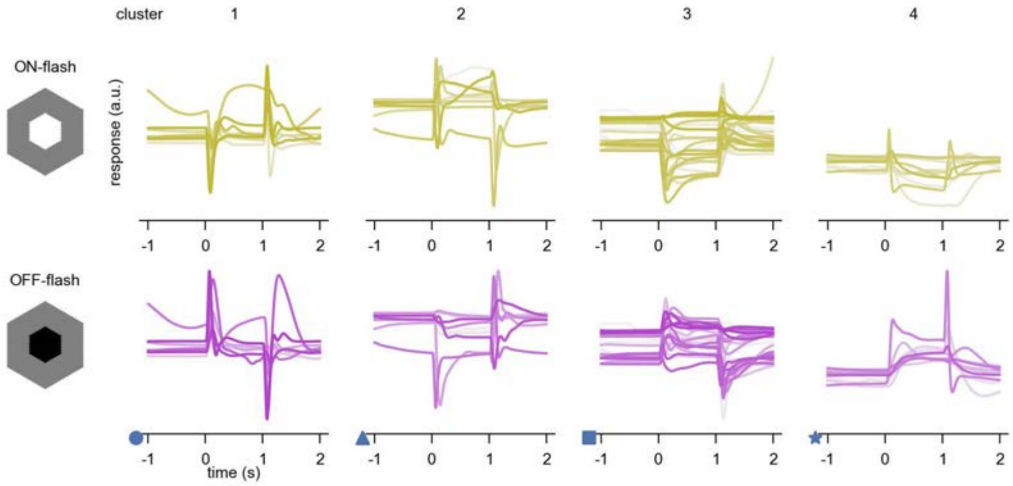

T5c - Figure 4: **Responses to flashes.** The top row shows responses to ON-flashes (yellow), the bottom row shows responses to OFF-flashes (magenta). The responses from the 50 different models that are separated into the different clusters (columns) overlay, with better task-performing models on top. Responses from better task-performing models are more saturated. The circular flashes (1s) cover 6 ommatidia in radius and are presented at time zero. Before and after, a grey-stimulus leads to a stationary state of the network.

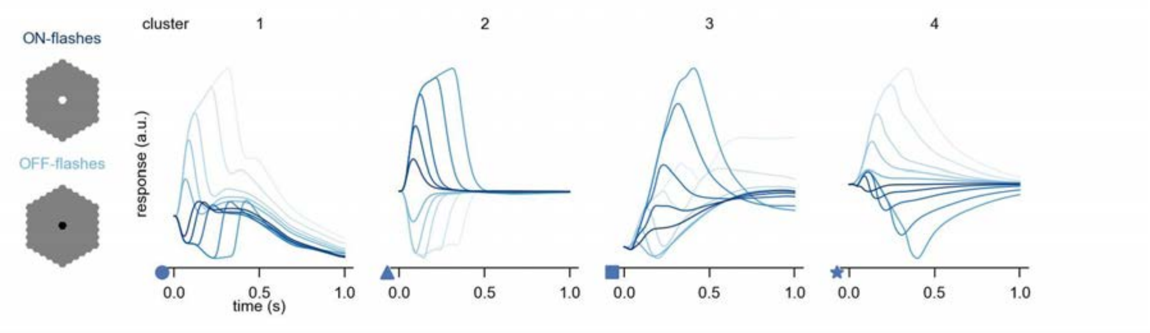

T5c - Figure 5: **Cluster-average responses to single-ommatidium flashes.** Responses to single-ommatidium ON-flashes (dark blue shades) and single-ommatidium OFF-flashes (light blue shades) of 20ms, 50ms, 100ms, 200ms, 300ms duration. The flashes occur at second zero.

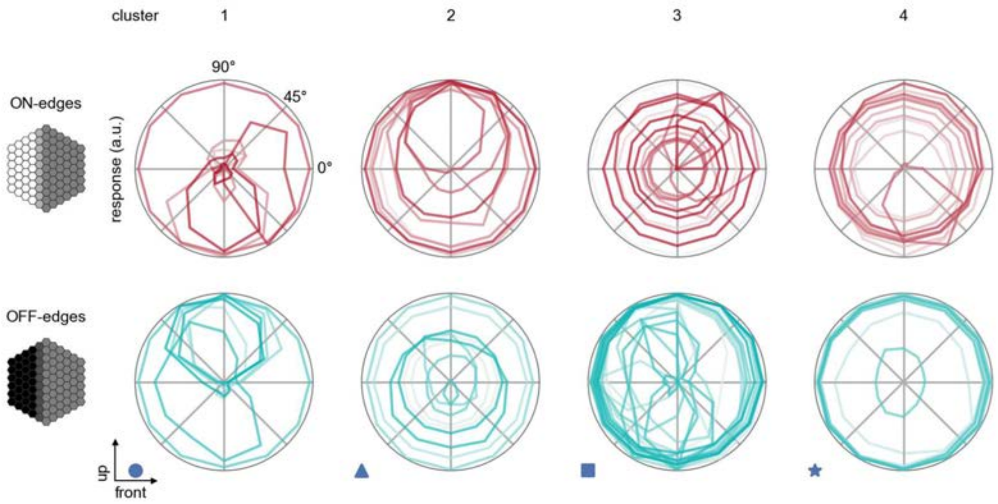

T5c - Figure 6: **Peak responses to moving edges.** The top row shows peak responses to moving ON-edges (red), the bottom row shows peak responses to moving OFF-edges (turqoise). The peak responses are averaged over edge-speeds. Edge-stimuli move in different directions from 0 to 360 degrees. The responses from the different models in the different clusters (columns) overlay. Responses from better task-performing models are more saturated.

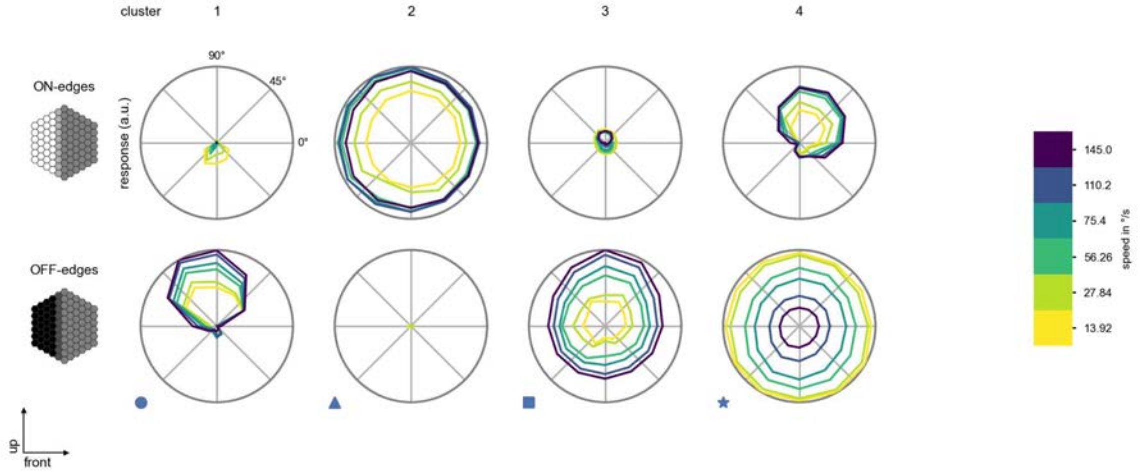

T5c - Figure 7: **Peak responses to moving edges from task-optimal models.** The top row shows peak responses to moving ON-edges, the bottom row shows peak responses to moving OFF-edges of varying speeds from 13.92°/s to 145°/s (yellow to dark blue). The edge-stimuli move in different directions from 0 to 360 degrees and at different speeds. Responses from the task-optimal model in the respective cluster.

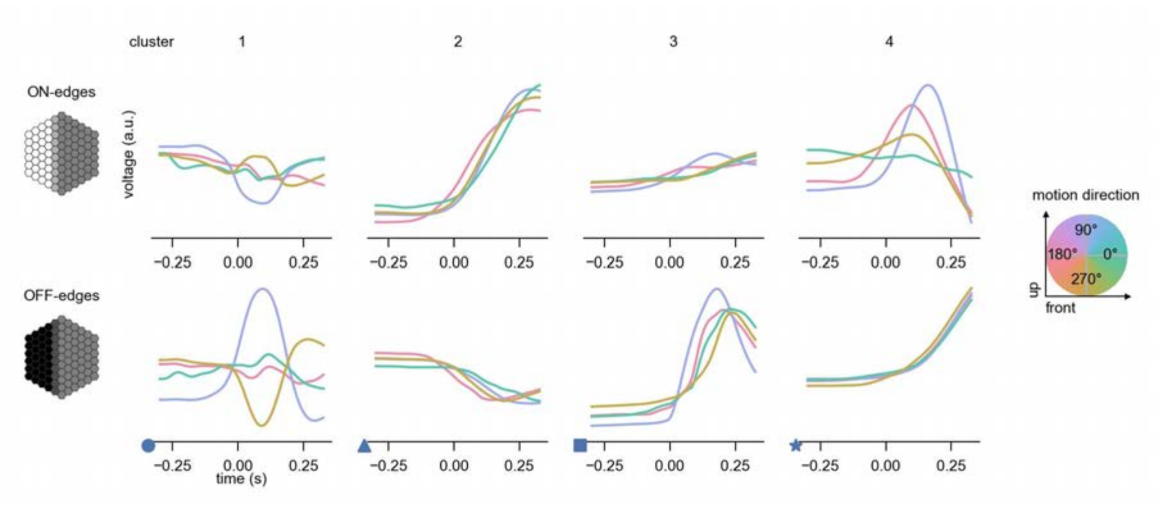

T5c - Figure 8: **Responses to moving edges from task-optimal models.** Responses to moving ON-edges (top row) and to moving OFF-edges (bottom row). Edges move in different directions from 0 to 360 degrees and at different speeds. Responses are from the task-optimal model in the respective cluster. Edges moving at 75.4°/s in all cardinal directions (green 0°, blue 90°, red 180°, yellow 270°) from -22.5 to 22.5° visual angle.

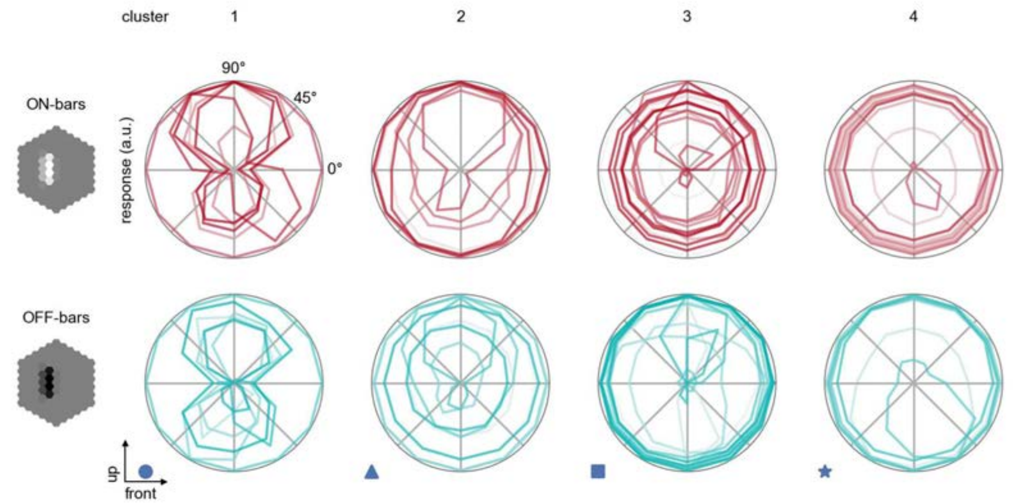

T5c - Figure 9: **Peak responses to moving bars.** The top row shows peak responses to moving ON-bars (red), the bottom row shows peak responses to moving OFF-bars (turqoise). The peak responses are averaged over bar-speeds. Bar-stimuli move in different directions from 0 to 360 degrees. The responses from the different models in the different clusters (columns) overlay. Responses from better task-performing models are more saturated.

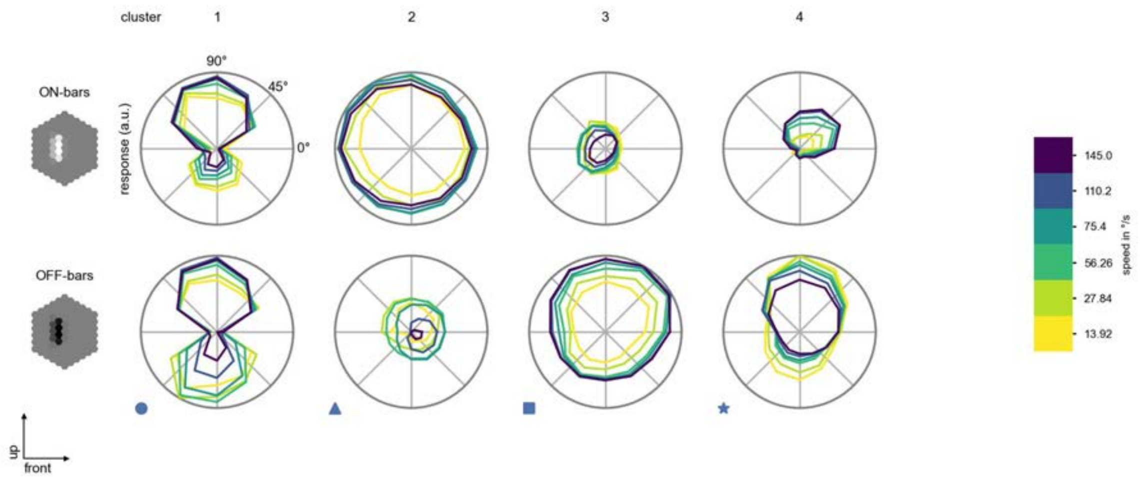

T5c - Figure 10: **Peak responses to moving bars from task-optimal models.** The top row shows peak responses to moving ON-bars, the bottom row shows peak responses to moving OFF-bars of varying speeds from 13.92°/s to 145°/s (yellow to dark blue). The bar-stimuli move in different directions from 0 to 360 degrees and at different speeds. Responses from the task-optimal model in the respective cluster.

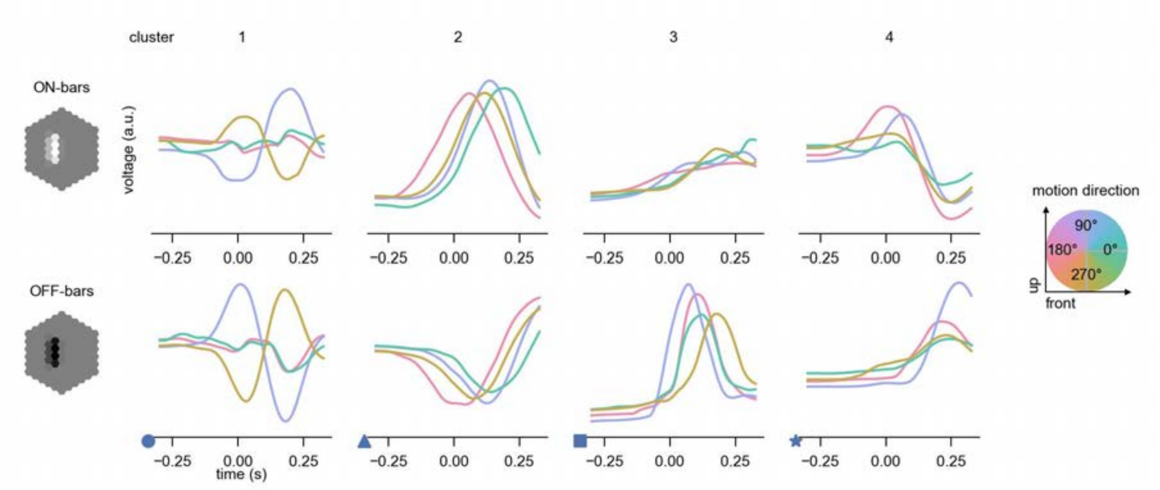

T5c - Figure 11: **Responses to moving bars from task-optimal models.** Responses to moving ON-bars (top row) and to moving OFF-bars (bottom row). Bars move in different directions from 0 to 360 degrees and at different speeds. Responses are from the task-optimal model in the respective cluster. Bars moving at 75.4°/s in all cardinal directions (green 0°, blue 90°, red 180°, yellow 270°) from -22.5 to 22.5° visual angle.

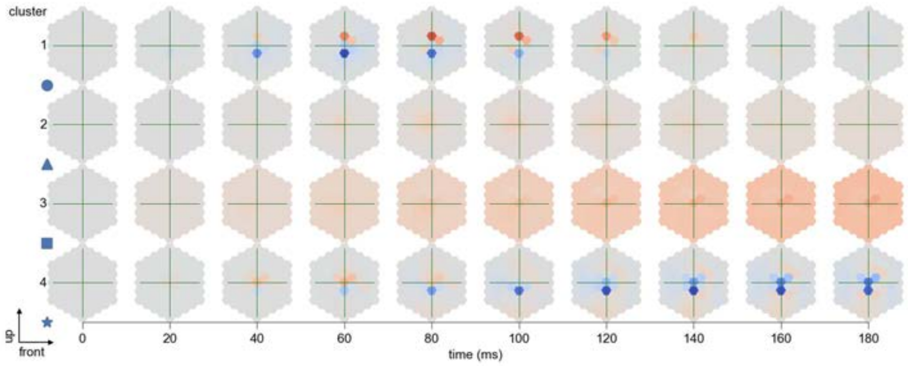

T5c - Figure 12: **Spatio-temporal receptive field.** Responses of the central cell to ON-impulses (5 ms) at single-ommatidium flash locations. The flash occurs at second zero. Responses from the task-optimal model of the respective cluster (rows). Red indicates depolarization, blue indicates hyperpolarization.

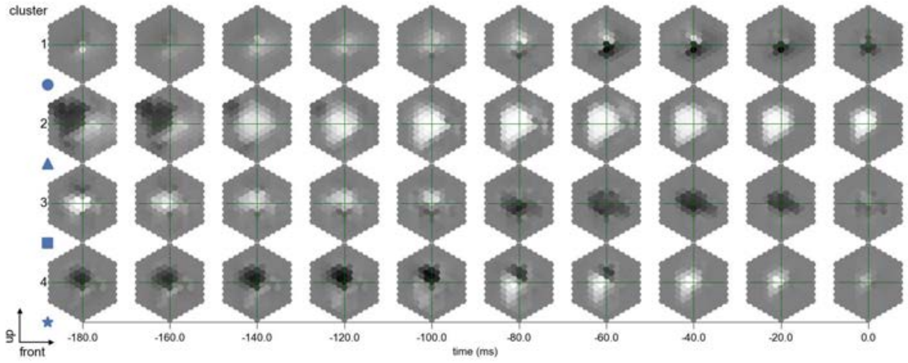

T5c - Figure 13: **Maximally excitatory stimuli.** Each row presents the regularized naturalistic-stimulus from the Sintel dataset that maximizes the cell type's central column response at second zero in the task-optimal model of the respective cluster (rows).

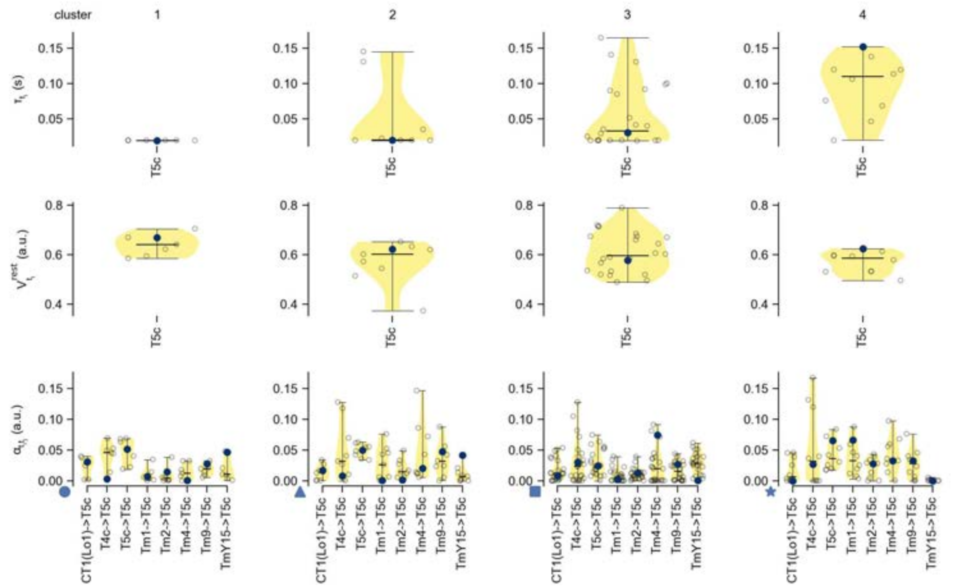

T5c - Figure 14: **Task-constrained parameters.** Each column shows the parameters inferred within the respective cluster. First row: learned time constants of the cell type. Second row: resting potentials of the cell type. Third row: scaling factors for the convolutional filters. The blue scatter represents the parameters from the task-optimal model within the cluster.

43 T5d

← Cell types

Figures

1 Anatomical receptive fields. . . . 306
2 Anatomical projective fields. . . . 307
3 Clustering of the responses to naturalistic stimuli. . . . 307
4 Responses to flashes. . . . 308
5 Cluster-average responses to single-ommatidium flashes. . . . 308
6 Peak responses to moving edges. . . . 309
7 Peak responses to moving edges from task-optimal models. . . . 309
8 Responses to moving edges from task-optimal models. . . . 310
9 Peak responses to moving bars. . . . 310
10 Peak responses to moving bars from task-optimal models. . . . 311
11 Responses to moving bars from task-optimal models. . . . 311
12 Spatio-temporal receptive field. . . . 312
13 Maximally excitatory stimuli. . . . 312
14 Task-constrained parameters. . . . 313

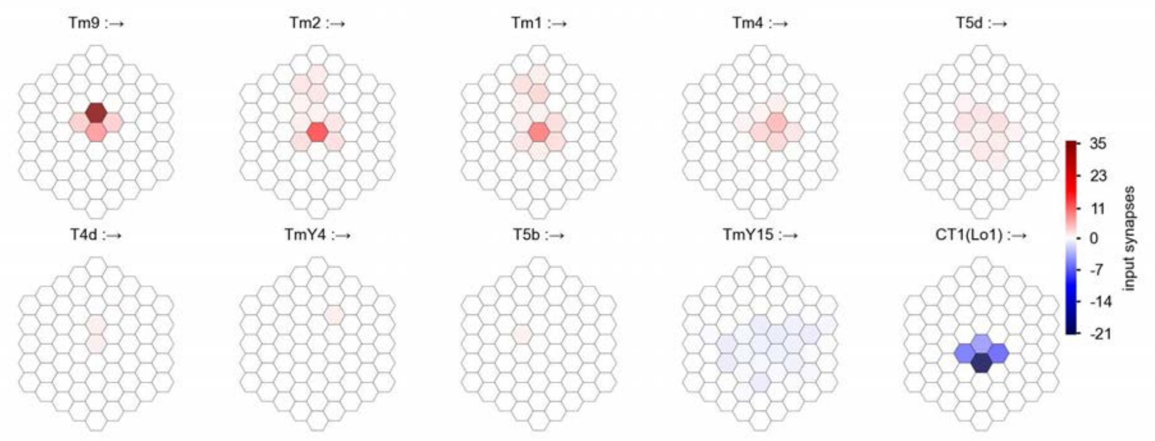

T5d - Figure 1: **Anatomical receptive fields.** Each colored hexagon is an input connection, with the connection strength characterized by the average number of synapses that we count from the EM reconstruction. Red indicates excitatory synapses, blue indicates inhibitory synapses from inferred signs. Filters in the order of their total number of synapses.

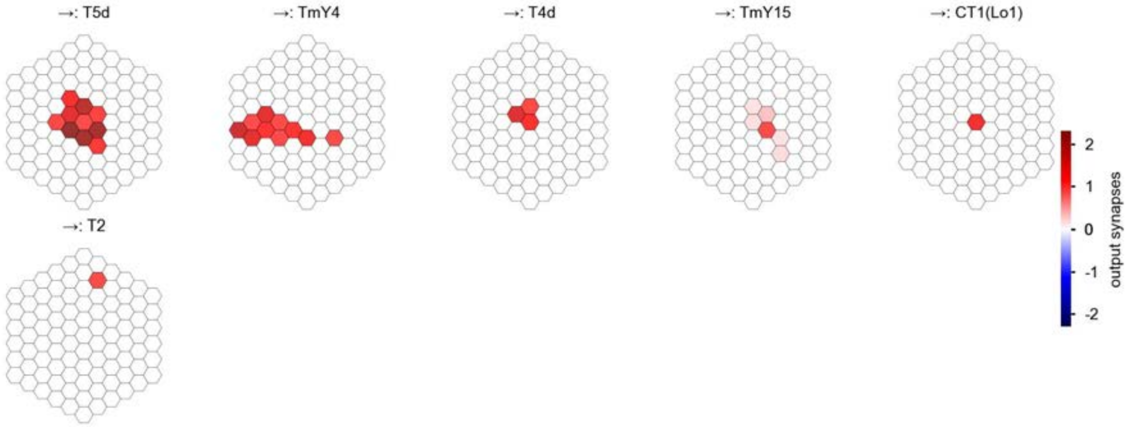

T5d - Figure 2: **Anatomical projective fields.** Each colored hexagon is an output connection, with the connection strength characterized by the average number of synapses that we count from the EM reconstruction. Red indicates excitatory synapses, blue indicates inhibitory synapses from inferred signs. Filters in the order of their total number of synapses.

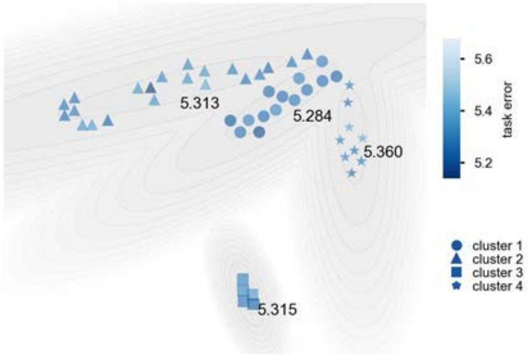

T5d - Figure 3: **Clustering of the responses to naturalistic stimuli.** Clustering of the 50 models based on the cell type responses to naturalistic scenes from the Sintel dataset. Scatterpoints represent individual models colored by their task error.

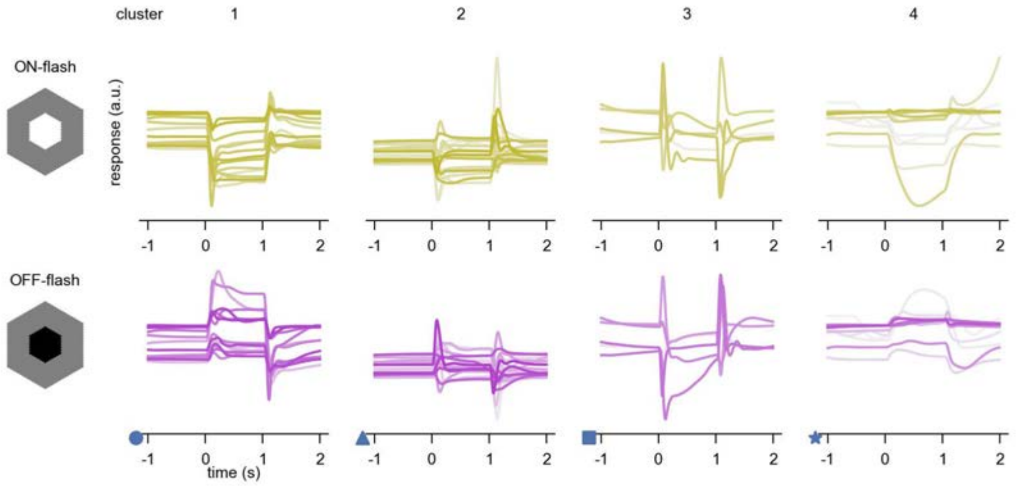

T5d - Figure 4: **Responses to flashes.** The top row shows responses to ON-flashes (yellow), the bottom row shows responses to OFF-flashes (magenta). The responses from the 50 different models that are separated into the different clusters (columns) overlay, with better task-performing models on top. Responses from better task-performing models are more saturated. The circular flashes (1s) cover 6 ommatidia in radius and are presented at time zero. Before and after, a grey-stimulus leads to a stationary state of the network.

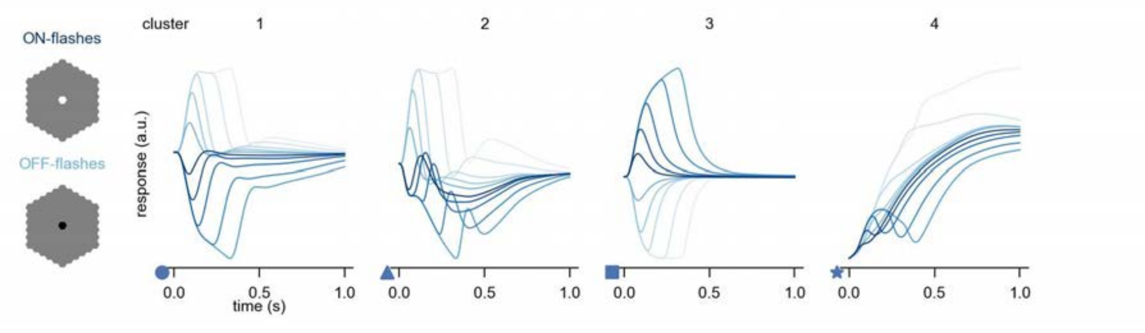

T5d - Figure 5: **Cluster-average responses to single-ommatidium flashes.** Responses to single-ommatidium ON-flashes (dark blue shades) and single-ommatidium OFF-flashes (light blue shades) of 20ms, 50ms, 100ms, 200ms, 300ms duration. The flashes occur at second zero.

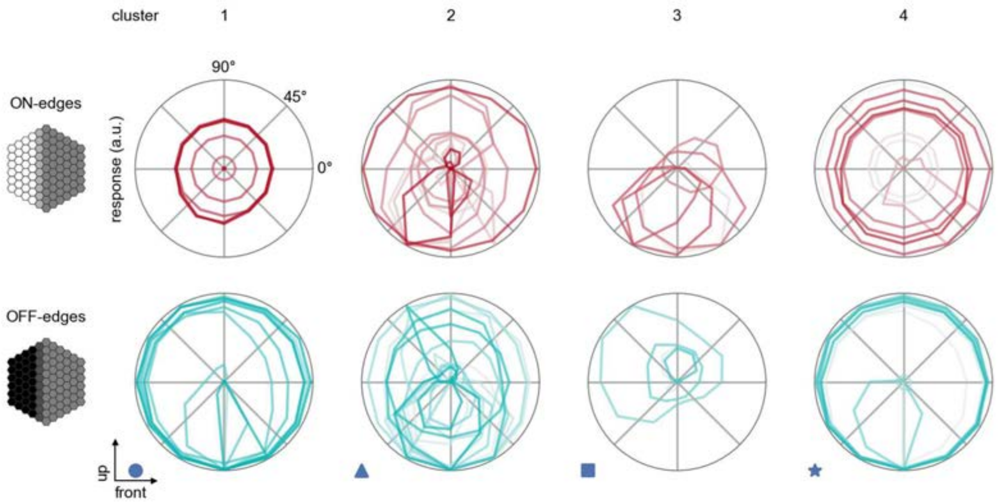

T5d - Figure 6: **Peak responses to moving edges.** The top row shows peak responses to moving ON-edges (red), the bottom row shows peak responses to moving OFF-edges (turqoise). The peak responses are averaged over edge-speeds. Edge-stimuli move in different directions from 0 to 360 degrees. The responses from the different models in the different clusters (columns) overlay. Responses from better task-performing models are more saturated.

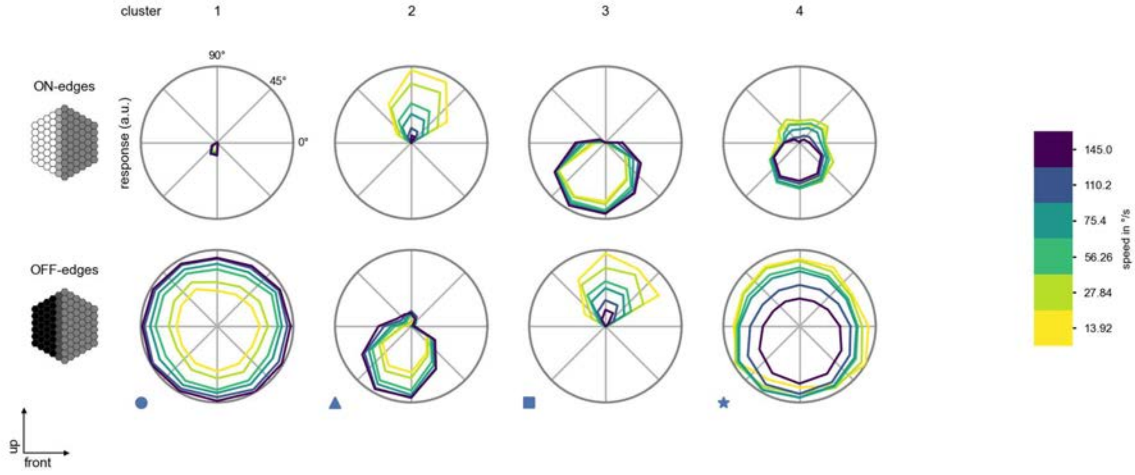

T5d - Figure 7: **Peak responses to moving edges from task-optimal models.** The top row shows peak responses to moving ON-edges, the bottom row shows peak responses to moving OFF-edges of varying speeds from 13.92°/s to 145°/s (yellow to dark blue). The edge-stimuli move in different directions from 0 to 360 degrees and at different speeds. Responses from the task-optimal model in the respective cluster.

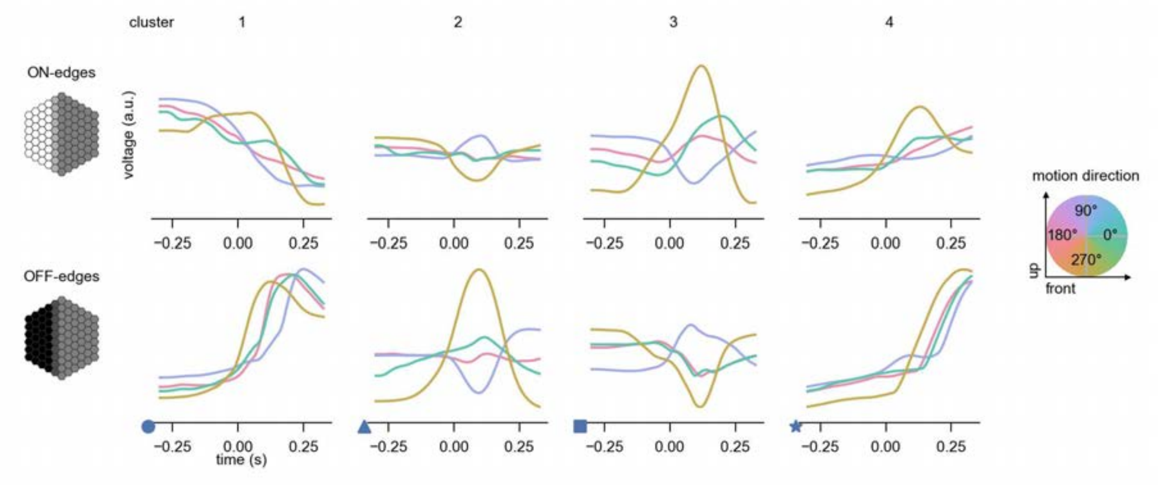

T5d - Figure 8: **Responses to moving edges from task-optimal models.** Responses to moving ON-edges (top row) and to moving OFF-edges (bottom row). Edges move in different directions from 0 to 360 degrees and at different speeds. Responses are from the task-optimal model in the respective cluster. Edges moving at 75.4°/s in all cardinal directions (green 0°, blue 90°, red 180°, yellow 270°) from -22.5 to 22.5° visual angle.

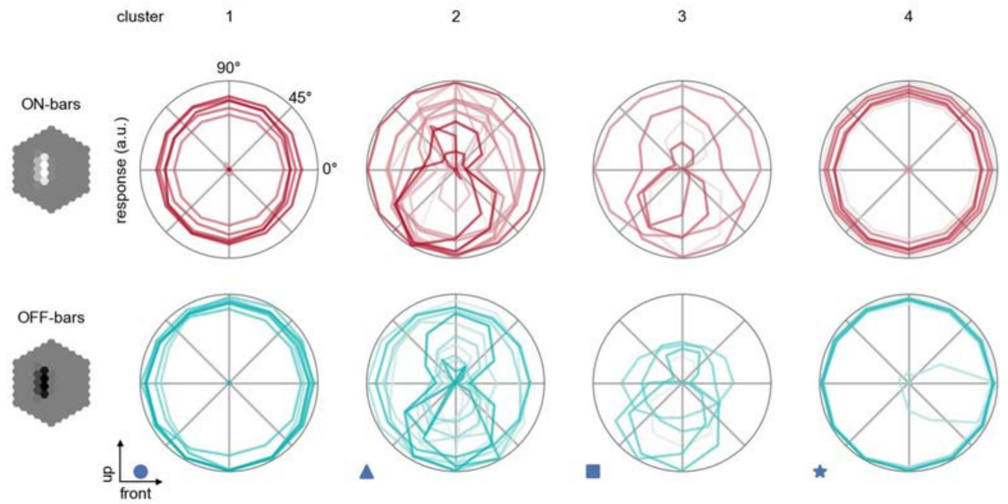

T5d - Figure 9: **Peak responses to moving bars.** The top row shows peak responses to moving ON-bars (red), the bottom row shows peak responses to moving OFF-bars (turqoise). The peak responses are averaged over bar-speeds. Bar-stimuli move in different directions from 0 to 360 degrees. The responses from the different models in the different clusters (columns) overlay. Responses from better task-performing models are more saturated.

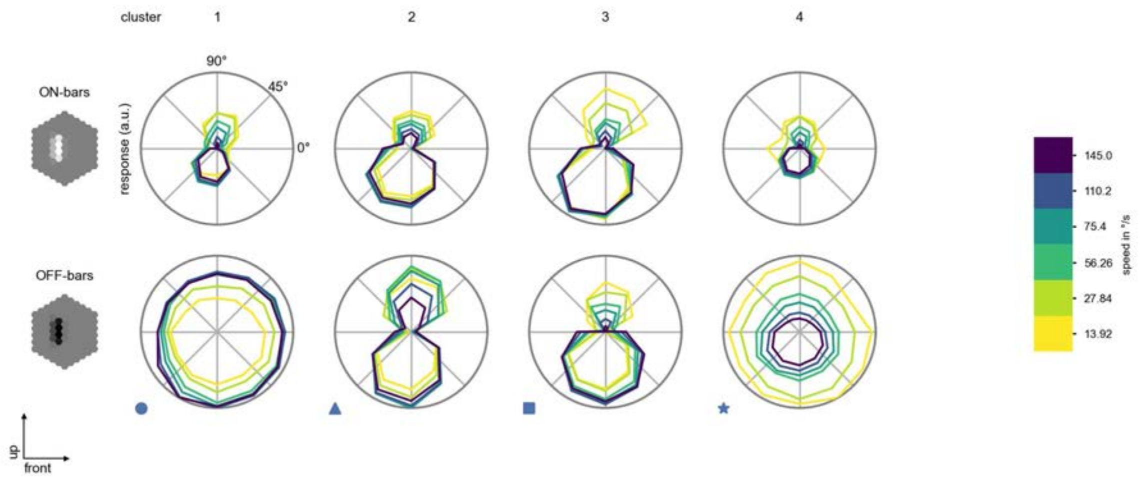

T5d - Figure 10: **Peak responses to moving bars from task-optimal models.** The top row shows peak responses to moving ON-bars, the bottom row shows peak responses to moving OFF-bars of varying speeds from 13.92°/s to 145°/s (yellow to dark blue). The bar-stimuli move in different directions from 0 to 360 degrees and at different speeds. Responses from the task-optimal model in the respective cluster.

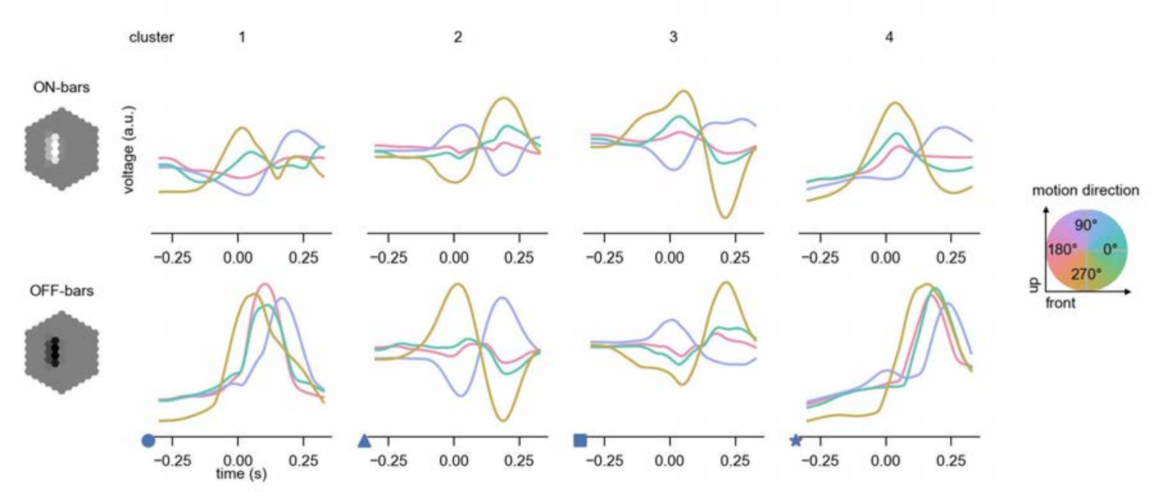

T5d - Figure 11: **Responses to moving bars from task-optimal models.** Responses to moving ON-bars (top row) and to moving OFF-bars (bottom row). Bars move in different directions from 0 to 360 degrees and at different speeds. Responses are from the task-optimal model in the respective cluster. Bars moving at 75.4°/s in all cardinal directions (green 0°, blue 90°, red 180°, yellow 270°) from -22.5 to 22.5° visual angle.

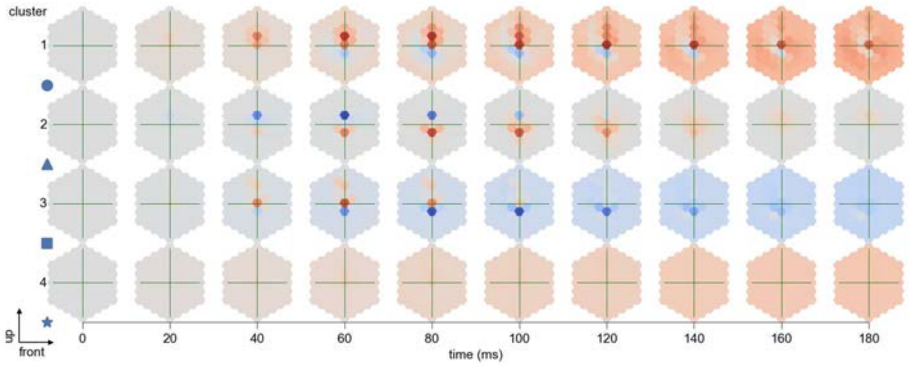

T5d - Figure 12: **Spatio-temporal receptive field.** Responses of the central cell to ON-impulses (5 ms) at single-ommatidium flash locations. The flash occurs at second zero. Responses from the task-optimal model of the respective cluster (rows). Red indicates depolarization, blue indicates hyperpolarization.

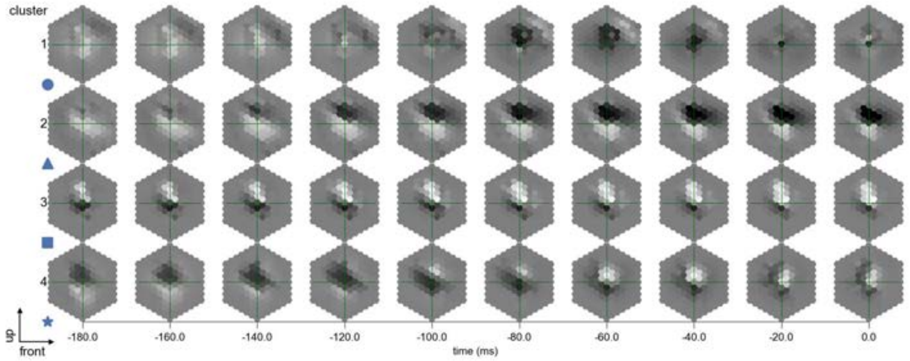

T5d - Figure 13: **Maximally excitatory stimuli.** Each row presents the regularized naturalistic-stimulus from the Sintel dataset that maximizes the cell type's central column response at second zero in the task-optimal model of the respective cluster (rows).

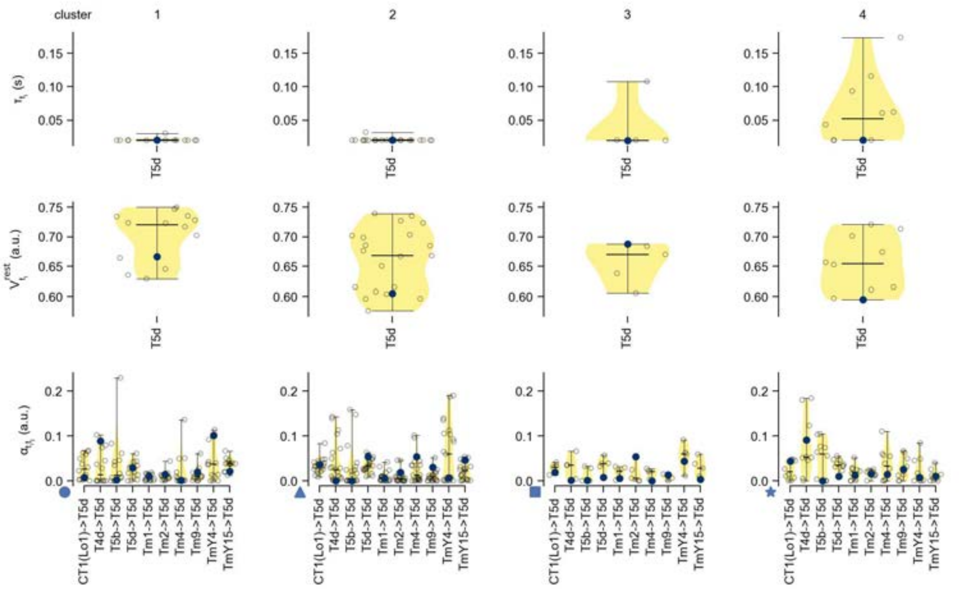

T5d - Figure 14: **Task-constrained parameters.** Each column shows the parameters inferred within the respective cluster. First row: learned time constants of the cell type. Second row: resting potentials of the cell type. Third row: scaling factors for the convolutional filters. The blue scatter represents the parameters from the task-optimal model within the cluster.

44 Tm1

← Cell types

Figures

1 Anatomical receptive fields. 314
2 Anatomical projective fields. 315
3 Clustering of the responses to naturalistic stimuli. 315
4 Responses to flashes. 316
5 Cluster-average responses to single-ommatidium flashes. 316
6 Peak responses to moving edges. 317
7 Peak responses to moving edges from task-optimal models. 317
8 Responses to moving edges from task-optimal models. 318
9 Peak responses to moving bars. 318
10 Peak responses to moving bars from task-optimal models. 319
11 Responses to moving bars from task-optimal models. 319
12 Spatio-temporal receptive field. 320
13 Maximally excitatory stimuli. 320
14 Task-constrained parameters. 321

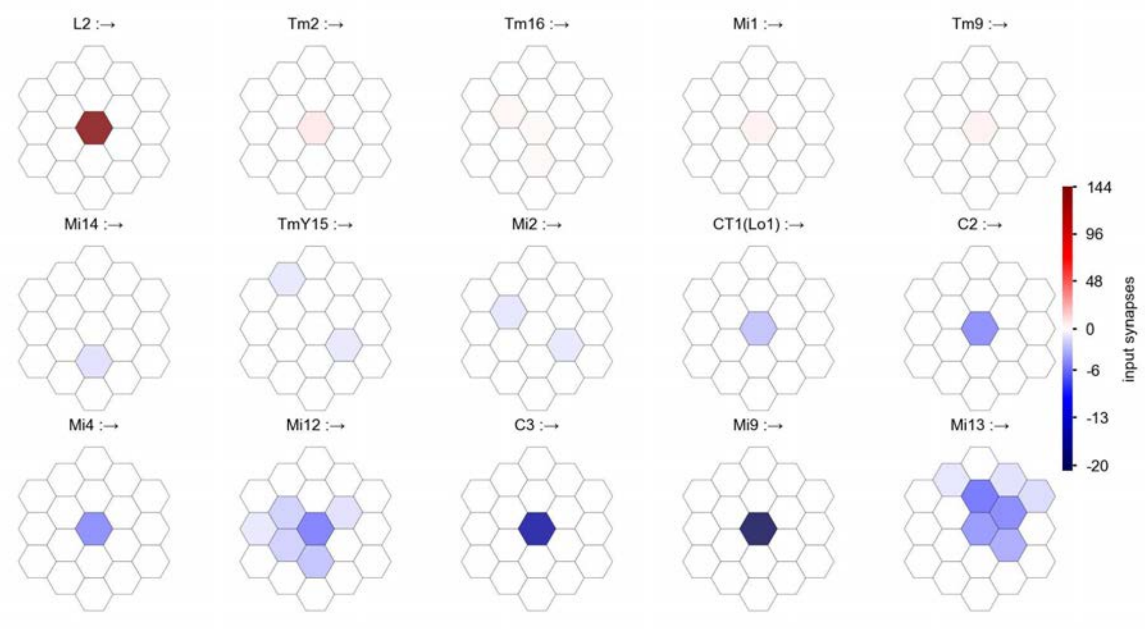

Tm1 - Figure 1: **Anatomical receptive fields.** Each colored hexagon is an input connection, with the connection strength characterized by the average number of synapses that we count from the EM reconstruction. Red indicates excitatory synapses, blue indicates inhibitory synapses from inferred signs. Filters in the order of their total number of synapses.

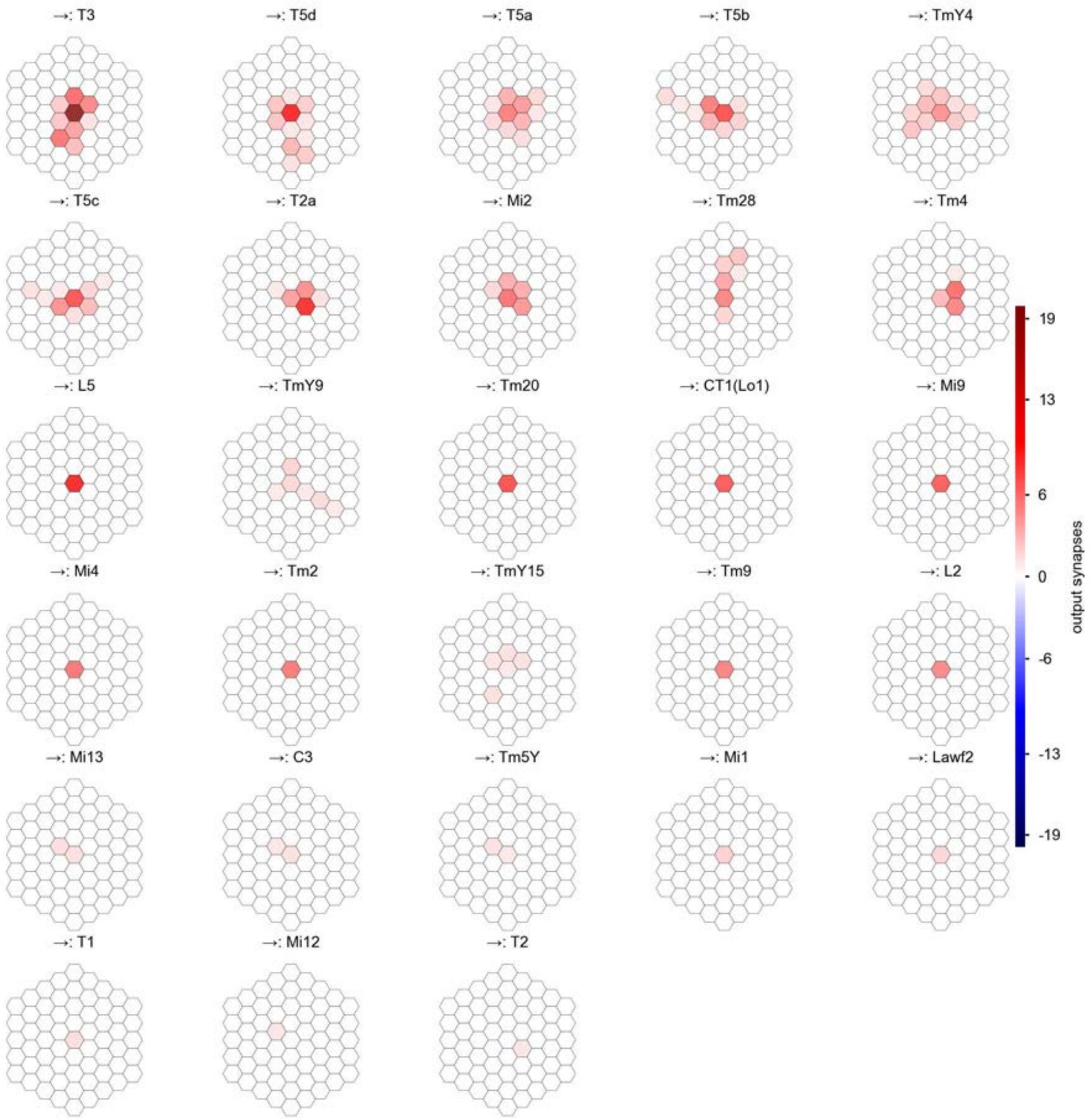

Tm1 - Figure 2: **Anatomical projective fields.** Each colored hexagon is an output connection, with the connection strength characterized by the average number of synapses that we count from the EM reconstruction. Red indicates excitatory synapses, blue indicates inhibitory synapses from inferred signs. Filters in the order of their total number of synapses.

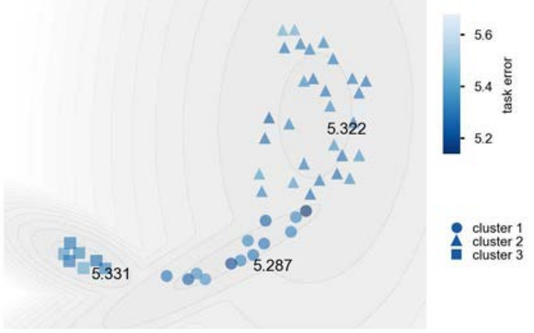

Tm1 - Figure 3: **Clustering of the responses to naturalistic stimuli.** Clustering of the 50 models based on the cell type responses to naturalistic scenes from the Sintel dataset. Scatterpoints represent individual models colored by their task error.

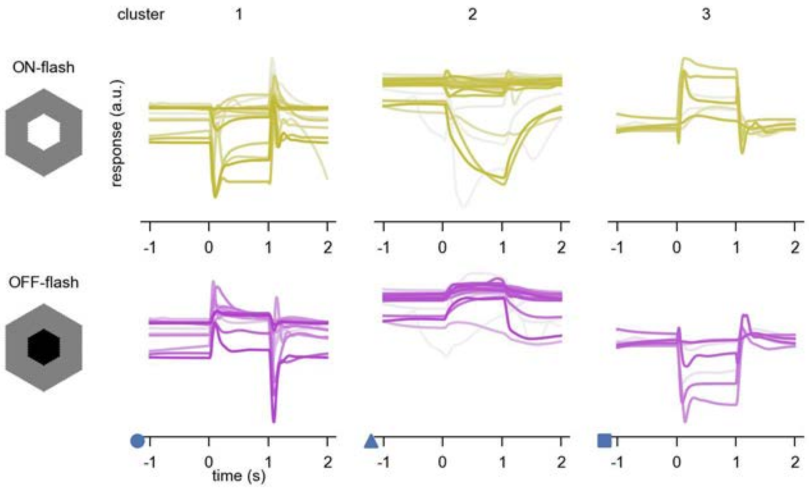

Tm1 - Figure 4: **Responses to flashes.** The top row shows responses to ON-flashes (yellow), the bottom row shows responses to OFF-flashes (magenta). The responses from the 50 different models that are separated into the different clusters (columns) overlay, with better task-performing models on top. Responses from better task-performing models are more saturated. The circular flashes (1s) cover 6 ommatidia in radius and are presented at time zero. Before and after, a grey-stimulus leads to a stationary state of the network.

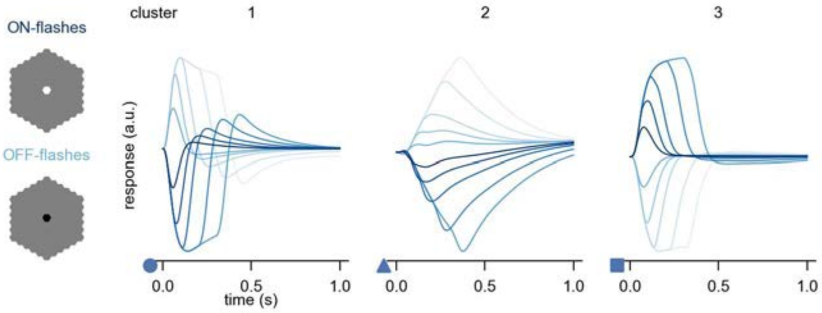

Tm1 - Figure 5: **Cluster-average responses to single-ommatidium flashes.** Responses to single-ommatidium ON-flashes (dark blue shades) and single-ommatidium OFF-flashes (light blue shades) of 20ms, 50ms, 100ms, 200ms, 300ms duration. The flashes occur at second zero.

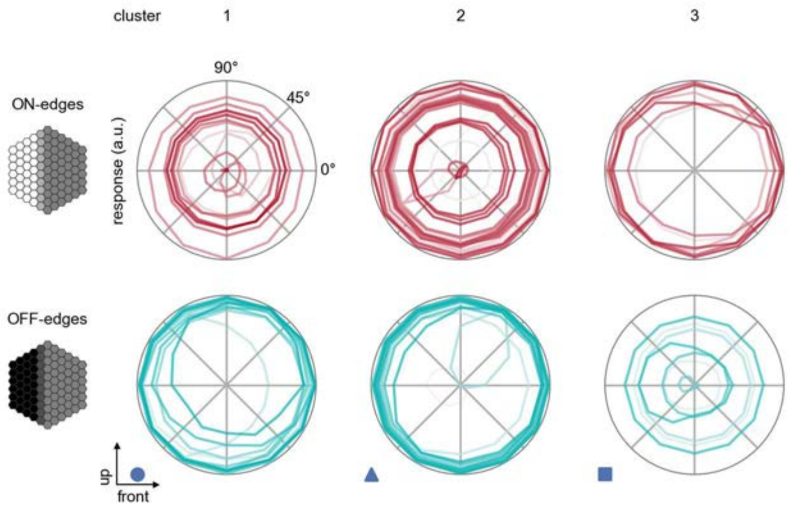

Tm1 - Figure 6: **Peak responses to moving edges.** The top row shows peak responses to moving ON-edges (red), the bottom row shows peak responses to moving OFF-edges (turqoise). The peak responses are averaged over edge-speeds. Edge-stimuli move in different directions from 0 to 360 degrees. The responses from the different models in the different clusters (columns) overlay. Responses from better task-performing models are more saturated.

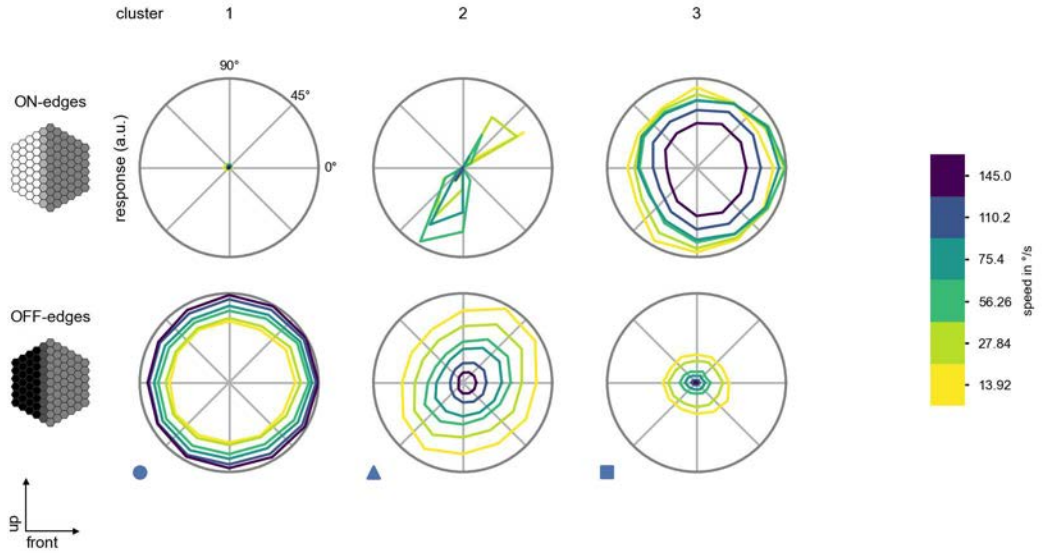

Tm1 - Figure 7: **Peak responses to moving edges from task-optimal models.** The top row shows peak responses to moving ON-edges, the bottom row shows peak responses to moving OFF-edges of varying speeds from 13.92°/s to 145°/s (yellow to dark blue). The edge-stimuli move in different directions from 0 to 360 degrees and at different speeds. Responses from the task-optimal model in the respective cluster.

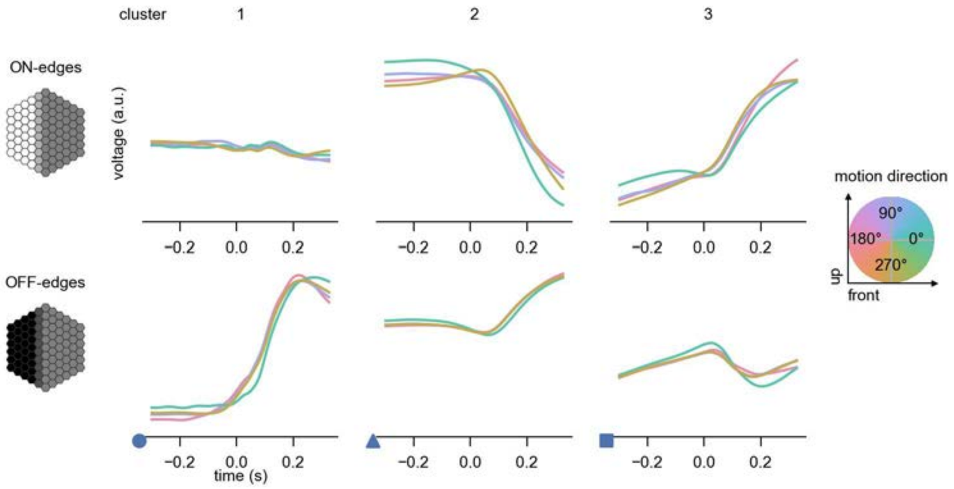

Tm1 - Figure 8: **Responses to moving edges from task-optimal models.** Responses to moving ON-edges (top row) and to moving OFF-edges (bottom row). Edges move in different directions from 0 to 360 degrees and at different speeds. Responses are from the task-optimal model in the respective cluster. Edges moving at 75.4°/s in all cardinal directions (green 0°, blue 90°, red 180°, yellow 270°) from -22.5 to 22.5° visual angle.

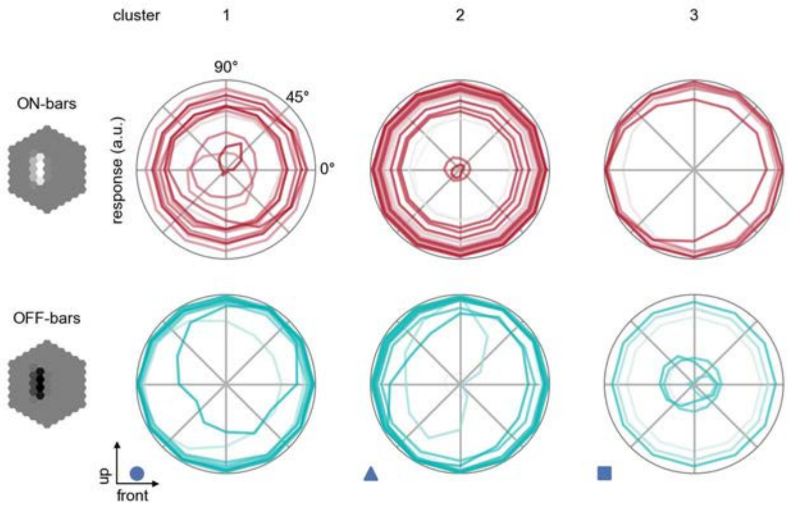

Tm1 - Figure 9: **Peak responses to moving bars.** The top row shows peak responses to moving ON-bars (red), the bottom row shows peak responses to moving OFF-bars (turqoise). The peak responses are averaged over bar-speeds. Bar-stimuli move in different directions from 0 to 360 degrees. The responses from the different models in the different clusters (columns) overlay. Responses from better task-performing models are more saturated.

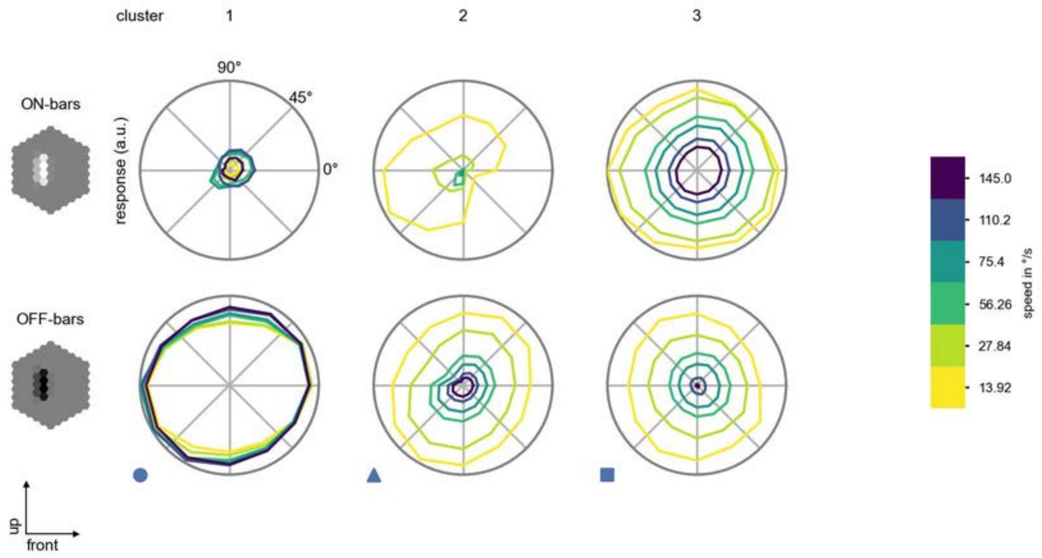

Tm1 - Figure 10: **Peak responses to moving bars from task-optimal models.** The top row shows peak responses to moving ON-bars, the bottom row shows peak responses to moving OFF-bars of varying speeds from 13.92°/s to 145°/s (yellow to dark blue). The bar-stimuli move in different directions from 0 to 360 degrees and at different speeds. Responses from the task-optimal model in the respective cluster.

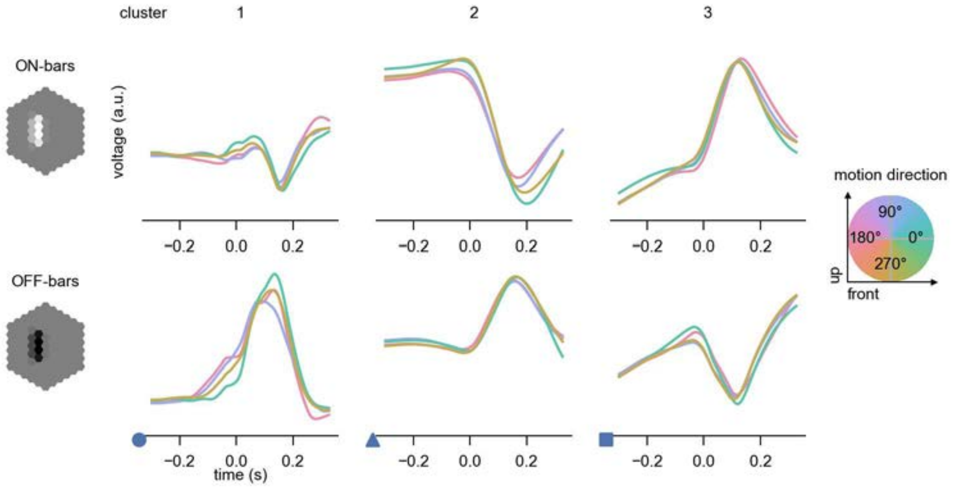

Tm1 - Figure 11: **Responses to moving bars from task-optimal models.** Responses to moving ON-bars (top row) and to moving OFF-bars (bottom row). Bars move in different directions from 0 to 360 degrees and at different speeds. Responses are from the task-optimal model in the respective cluster. Bars moving at 75.4°/s in all cardinal directions (green 0°, blue 90°, red 180°, yellow 270°) from -22.5 to 22.5° visual angle.

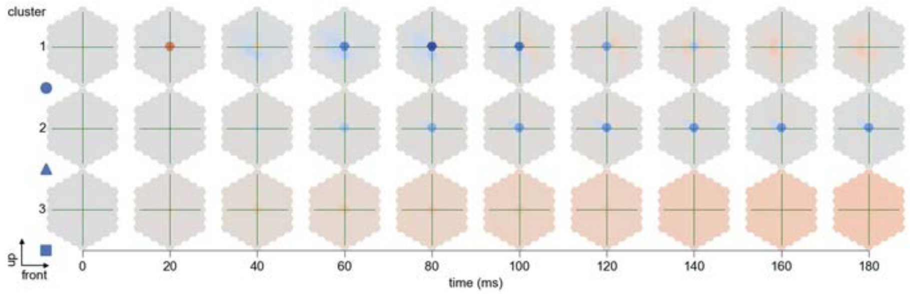

Tm1 - Figure 12: **Spatio-temporal receptive field.** Responses of the central cell to ON-impulses (5 ms) at single-ommatidium flash locations. The flash occurs at second zero. Responses from the task-optimal model of the respective cluster (rows). Red indicates depolarization, blue indicates hyperpolarization.

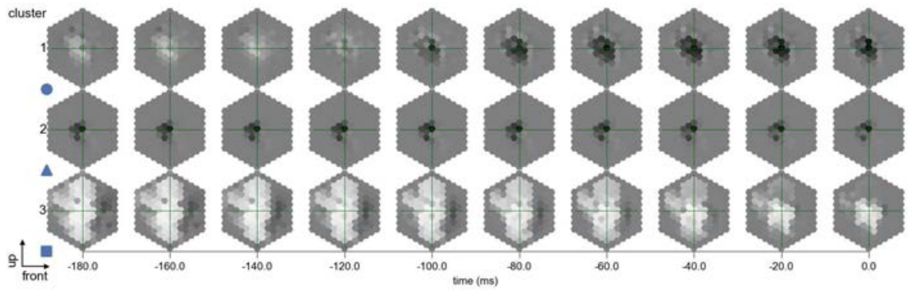

Tm1 - Figure 13: **Maximally excitatory stimuli.** Each row presents the regularized naturalistic-stimulus from the Sintel dataset that maximizes the cell type's central column response at second zero in the task-optimal model of the respective cluster (rows).

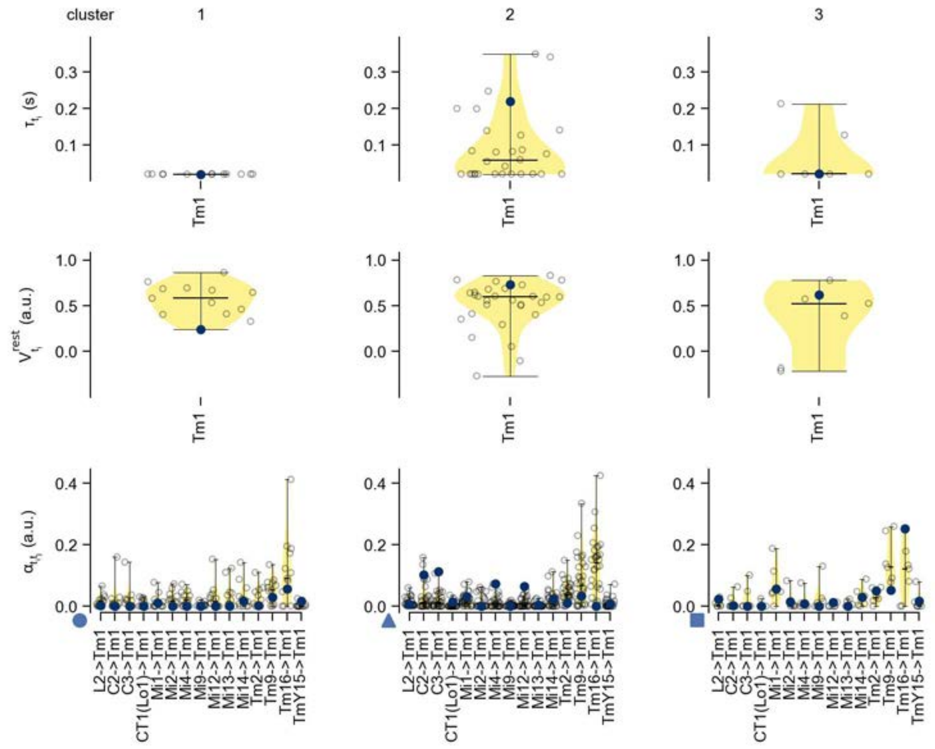

Tm1 - Figure 14: **Task-constrained parameters.** Each column shows the parameters inferred within the respective cluster. First row: learned time constants of the cell type. Second row: resting potentials of the cell type. Third row: scaling factors for the convolutional filters. The blue scatter represents the parameters from the task-optimal model within the cluster.

45 Tm2

← Cell types

Figures

1 Anatomical receptive fields. 322
2 Anatomical projective fields. 323
3 Clustering of the responses to naturalistic stimuli. 323
4 Responses to flashes. 324
5 Cluster-average responses to single-ommatidium flashes. 324
6 Peak responses to moving edges. 325
7 Peak responses to moving edges from task-optimal models. 325
8 Responses to moving edges from task-optimal models. 326
9 Peak responses to moving bars. 326
10 Peak responses to moving bars from task-optimal models. 327
11 Responses to moving bars from task-optimal models. 327
12 Spatio-temporal receptive field. 328
13 Maximally excitatory stimuli. 328
14 Task-constrained parameters. 329

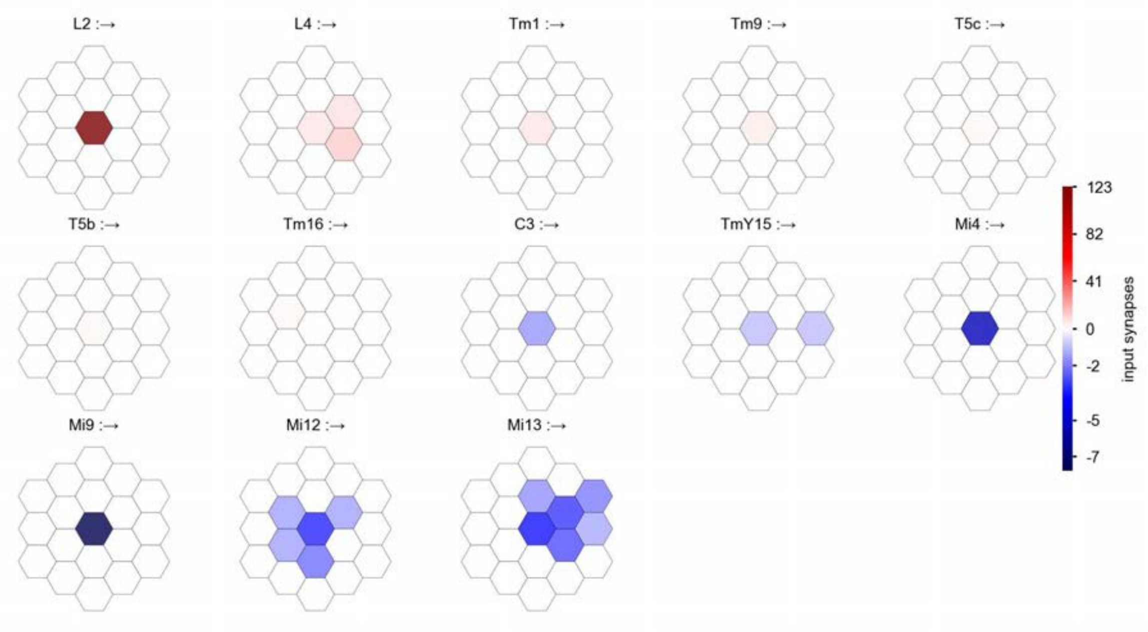

Tm2 - Figure 1: **Anatomical receptive fields.** Each colored hexagon is an input connection, with the connection strength characterized by the average number of synapses that we count from the EM reconstruction. Red indicates excitatory synapses, blue indicates inhibitory synapses from inferred signs. Filters in the order of their total number of synapses.

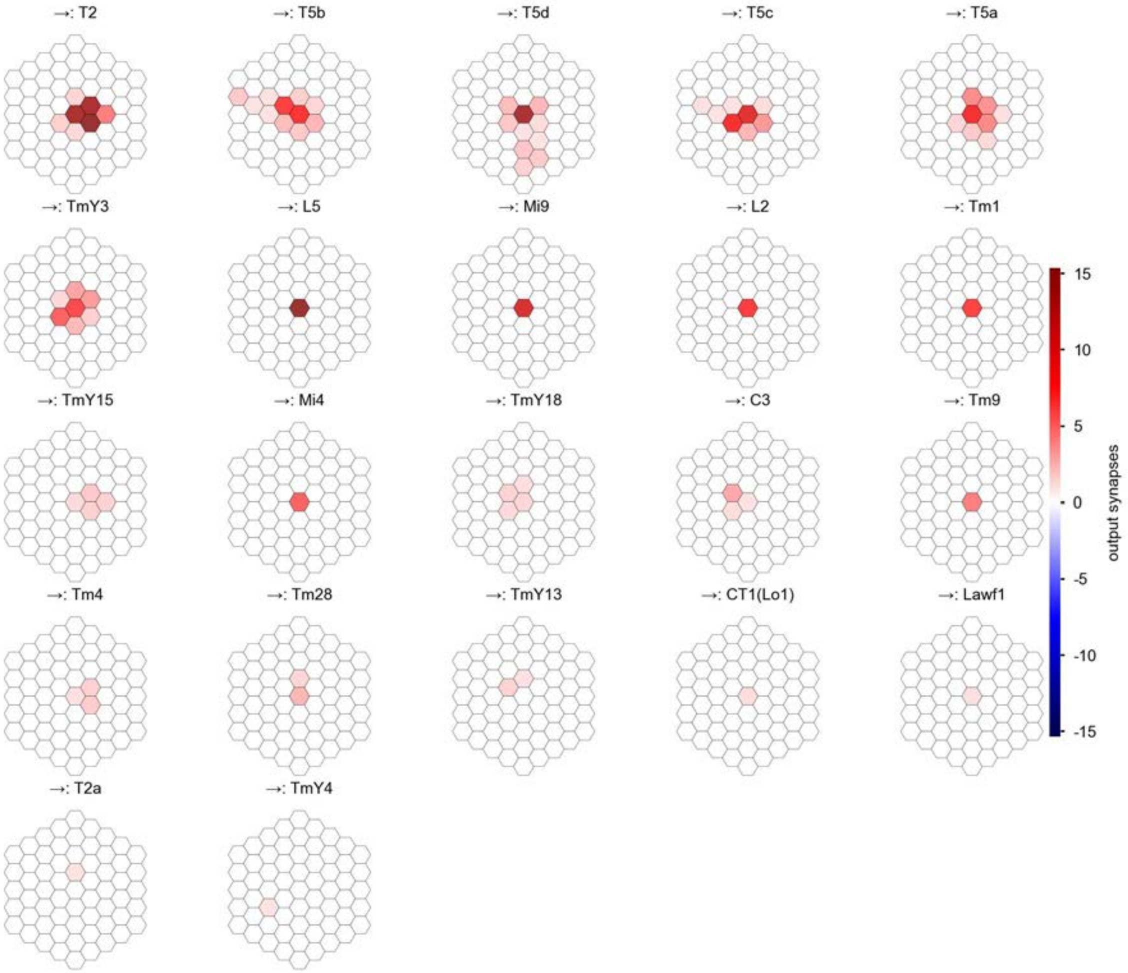

Tm2 - Figure 2: **Anatomical projective fields.** Each colored hexagon is an output connection, with the connection strength characterized by the average number of synapses that we count from the EM reconstruction. Red indicates excitatory synapses, blue indicates inhibitory synapses from inferred signs. Filters in the order of their total number of synapses.

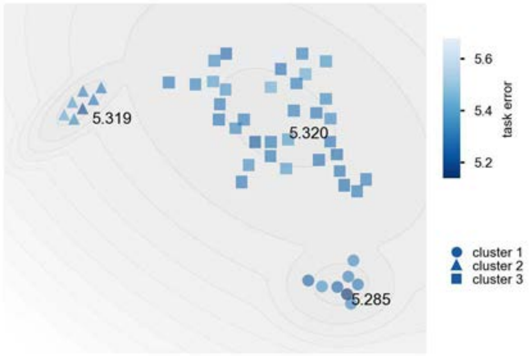

Tm2 - Figure 3: **Clustering of the responses to naturalistic stimuli.** Clustering of the 50 models based on the cell type responses to naturalistic scenes from the Sintel dataset. Scatterpoints represent individual models colored by their task error.

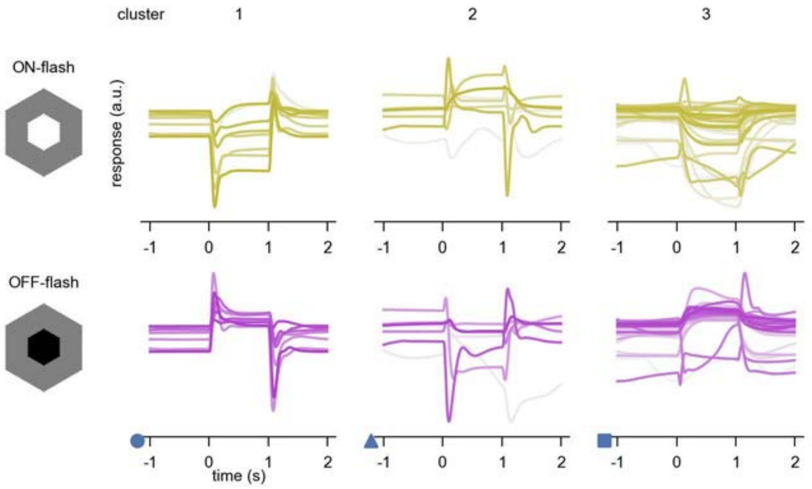

Tm2 - Figure 4: **Responses to flashes.** The top row shows responses to ON-flashes (yellow), the bottom row shows responses to OFF-flashes (magenta). The responses from the 50 different models that are separated into the different clusters (columns) overlay, with better task-performing models on top. Responses from better task-performing models are more saturated. The circular flashes (1s) cover 6 ommatidia in radius and are presented at time zero. Before and after, a grey-stimulus leads to a stationary state of the network.

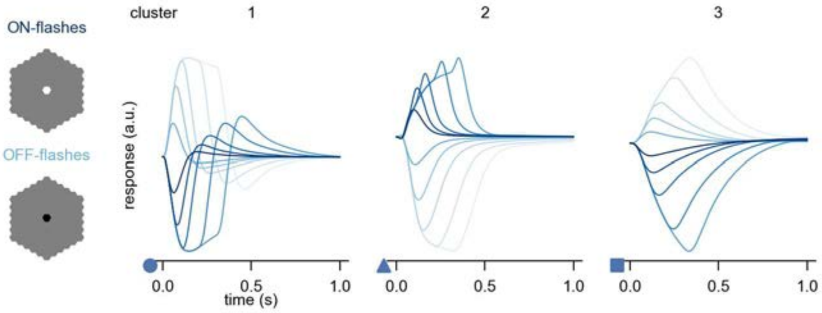

Tm2 - Figure 5: **Cluster-average responses to single-ommatidium flashes.** Responses to single-ommatidium ON-flashes (dark blue shades) and single-ommatidium OFF-flashes (light blue shades) of 20ms, 50ms, 100ms, 200ms, 300ms duration. The flashes occur at second zero.

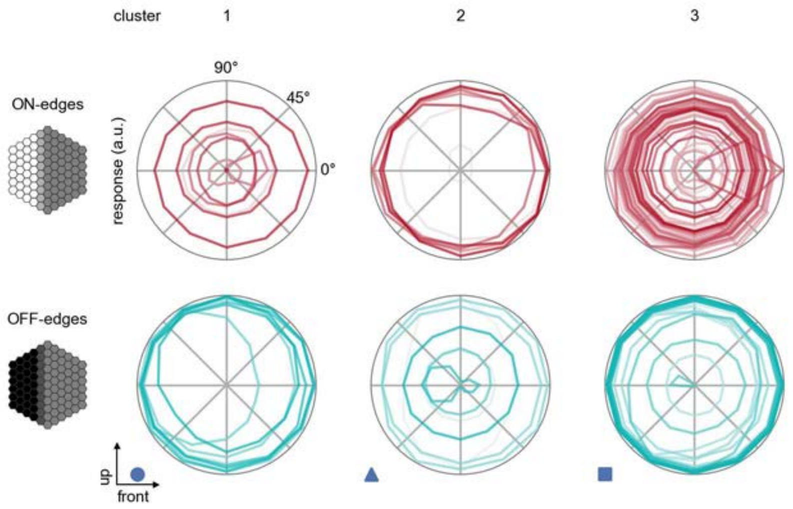

Tm2 - Figure 6: **Peak responses to moving edges.** The top row shows peak responses to moving ON-edges (red), the bottom row shows peak responses to moving OFF-edges (turqoise). The peak responses are averaged over edge-speeds. Edge-stimuli move in different directions from 0 to 360 degrees. The responses from the different models in the different clusters (columns) overlay. Responses from better task-performing models are more saturated.

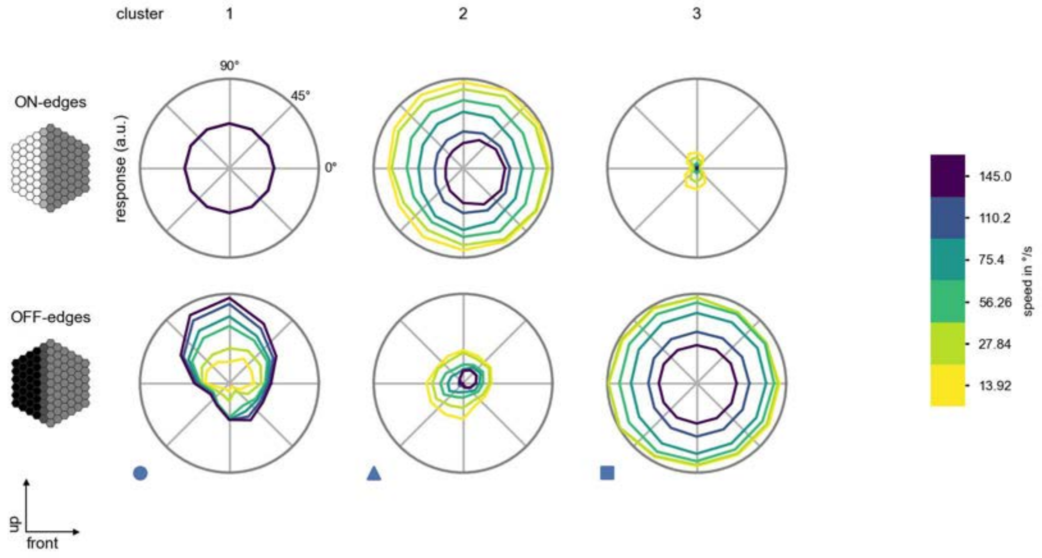

Tm2 - Figure 7: **Peak responses to moving edges from task-optimal models.** The top row shows peak responses to moving ON-edges, the bottom row shows peak responses to moving OFF-edges of varying speeds from 13.92°/s to 145°/s (yellow to dark blue). The edge-stimuli move in different directions from 0 to 360 degrees and at different speeds. Responses from the task-optimal model in the respective cluster.

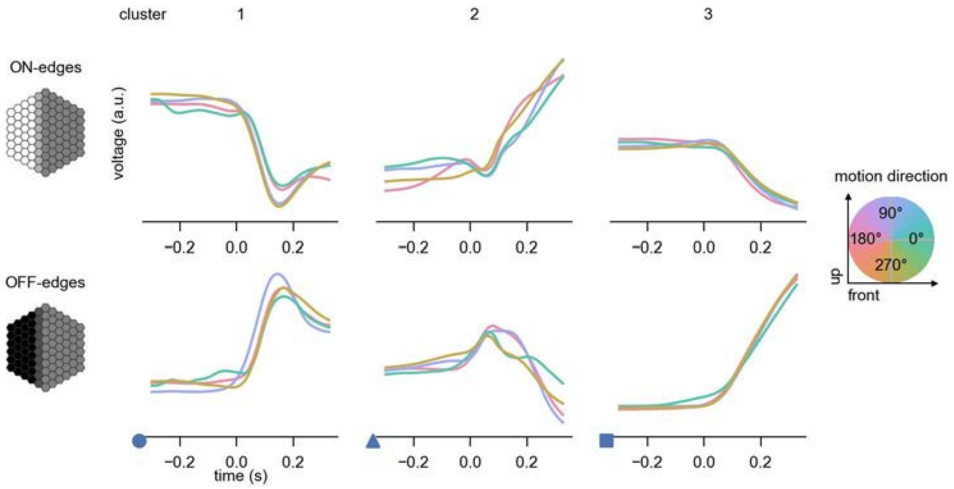

Tm2 - Figure 8: **Responses to moving edges from task-optimal models.** Responses to moving ON-edges (top row) and to moving OFF-edges (bottom row). Edges move in different directions from 0 to 360 degrees and at different speeds. Responses are from the task-optimal model in the respective cluster. Edges moving at 75.4°/s in all cardinal directions (green 0°, blue 90°, red 180°, yellow 270°) from -22.5 to 22.5° visual angle.

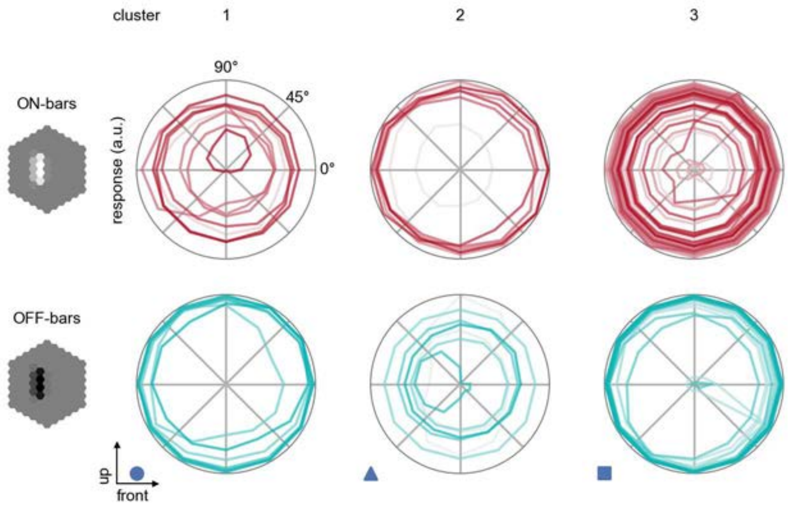

Tm2 - Figure 9: **Peak responses to moving bars.** The top row shows peak responses to moving ON-bars (red), the bottom row shows peak responses to moving OFF-bars (turqoise). The peak responses are averaged over bar-speeds. Bar-stimuli move in different directions from 0 to 360 degrees. The responses from the different models in the different clusters (columns) overlay. Responses from better task-performing models are more saturated.

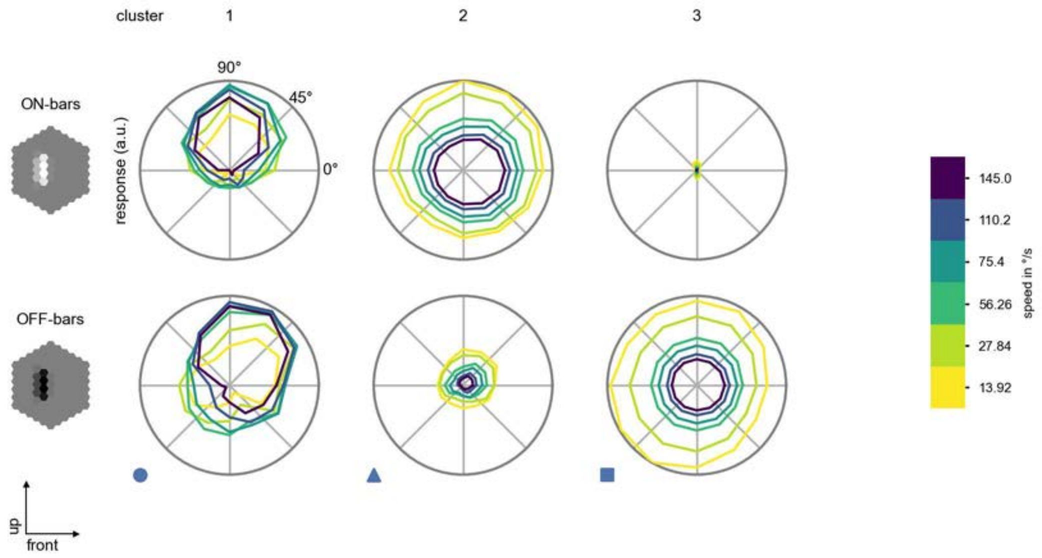

Tm2 - Figure 10: **Peak responses to moving bars from task-optimal models.** The top row shows peak responses to moving ON-bars, the bottom row shows peak responses to moving OFF-bars of varying speeds from 13.92°/s to 145°/s (yellow to dark blue). The bar-stimuli move in different directions from 0 to 360 degrees and at different speeds. Responses from the task-optimal model in the respective cluster.

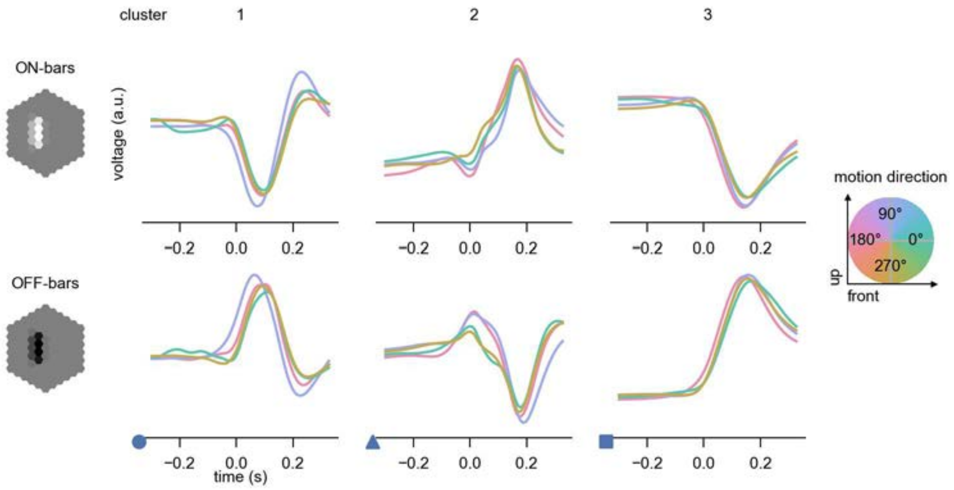

Tm2 - Figure 11: **Responses to moving bars from task-optimal models.** Responses to moving ON-bars (top row) and to moving OFF-bars (bottom row). Bars move in different directions from 0 to 360 degrees and at different speeds. Responses are from the task-optimal model in the respective cluster. Bars moving at 75.4°/s in all cardinal directions (green 0°, blue 90°, red 180°, yellow 270°) from -22.5 to 22.5° visual angle.

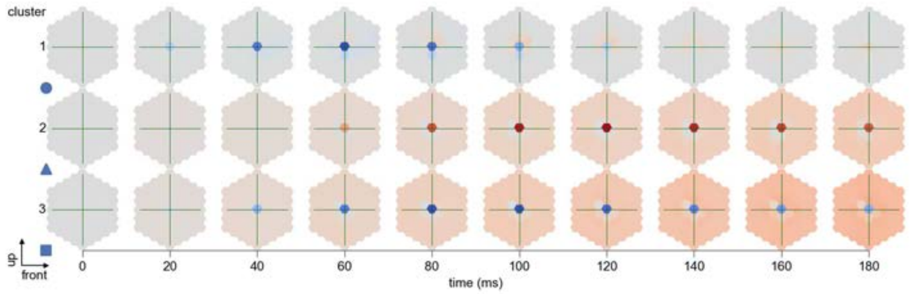

Tm2 - Figure 12: **Spatio-temporal receptive field.** Responses of the central cell to ON-impulses (5 ms) at single-ommatidium flash locations. The flash occurs at second zero. Responses from the task-optimal model of the respective cluster (rows). Red indicates depolarization, blue indicates hyperpolarization.

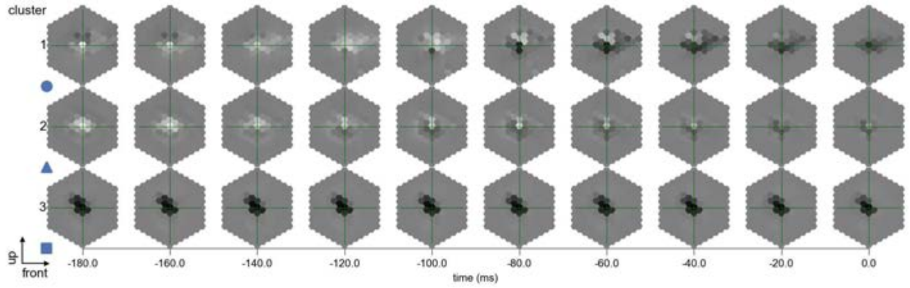

Tm2 - Figure 13: **Maximally excitatory stimuli.** Each row presents the regularized naturalistic-stimulus from the Sintel dataset that maximizes the cell type's central column response at second zero in the task-optimal model of the respective cluster (rows).

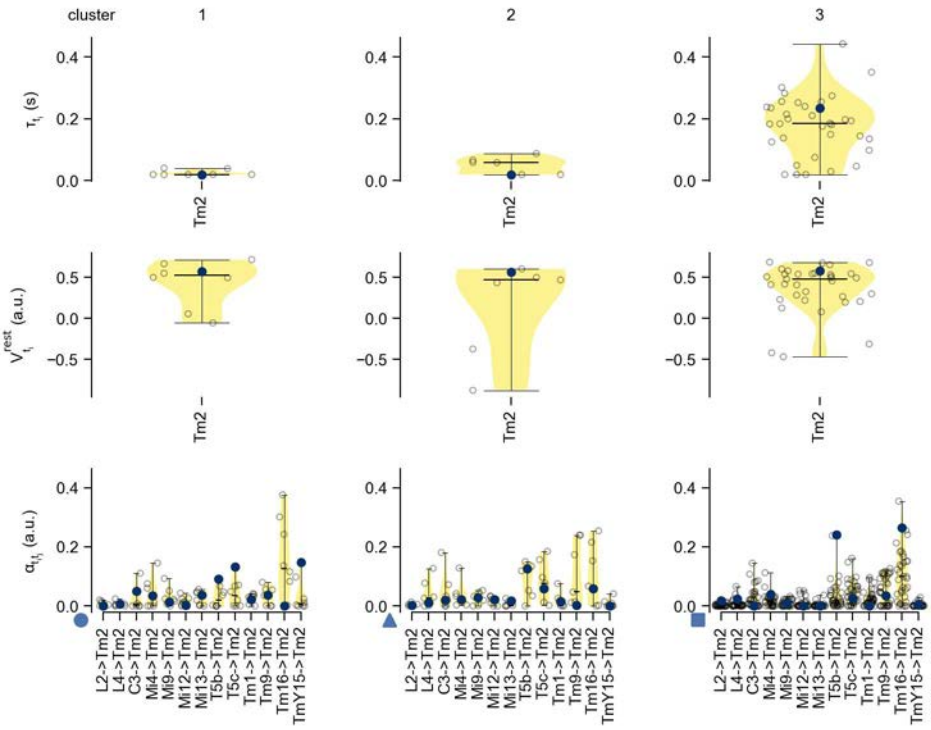

Tm2 - Figure 14: **Task-constrained parameters.** Each column shows the parameters inferred within the respective cluster. First row: learned time constants of the cell type. Second row: resting potentials of the cell type. Third row: scaling factors for the convolutional filters. The blue scatter represents the parameters from the task-optimal model within the cluster.

46 Tm3

← Cell types

Figures

1 Anatomical receptive fields. . . . 330
2 Anatomical projective fields. . . . 331
3 Clustering of the responses to naturalistic stimuli. . . . 331
4 Responses to flashes. . . . 332
5 Cluster-average responses to single-ommatidium flashes. . . . 332
6 Peak responses to moving edges. . . . 333
7 Peak responses to moving edges from task-optimal models. . . . 333
8 Responses to moving edges from task-optimal models. . . . 334
9 Peak responses to moving bars. . . . 334
10 Peak responses to moving bars from task-optimal models. . . . 335
11 Responses to moving bars from task-optimal models. . . . 335
12 Spatio-temporal receptive field. . . . 336
13 Maximally excitatory stimuli. . . . 336
14 Task-constrained parameters. . . . 337

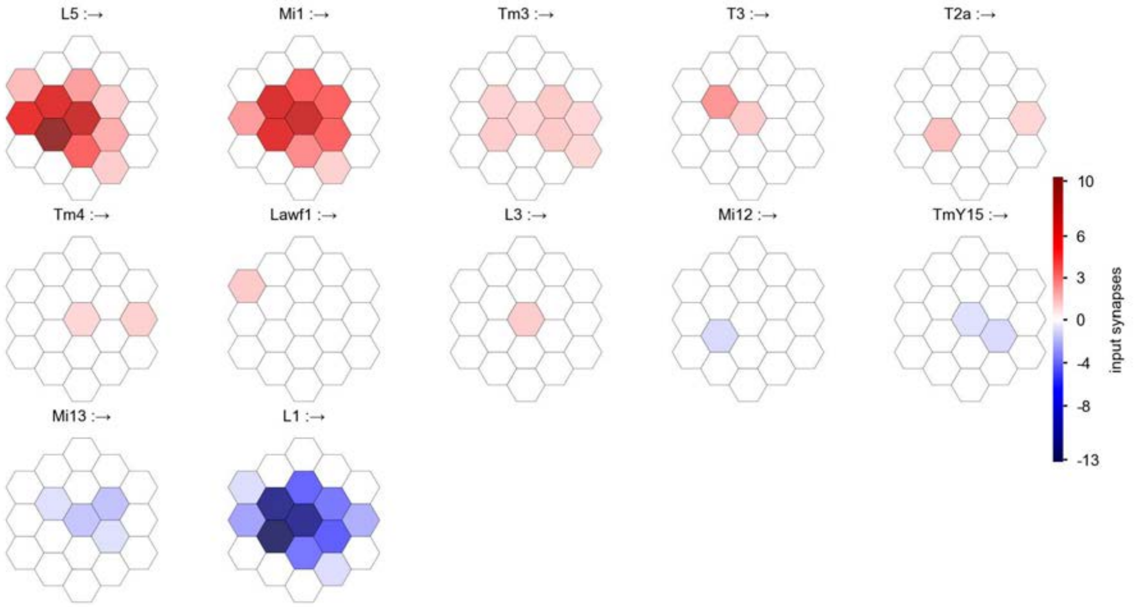

Tm3 - Figure 1: **Anatomical receptive fields.** Each colored hexagon is an input connection, with the connection strength characterized by the average number of synapses that we count from the EM reconstruction. Red indicates excitatory synapses, blue indicates inhibitory synapses from inferred signs. Filters in the order of their total number of synapses.

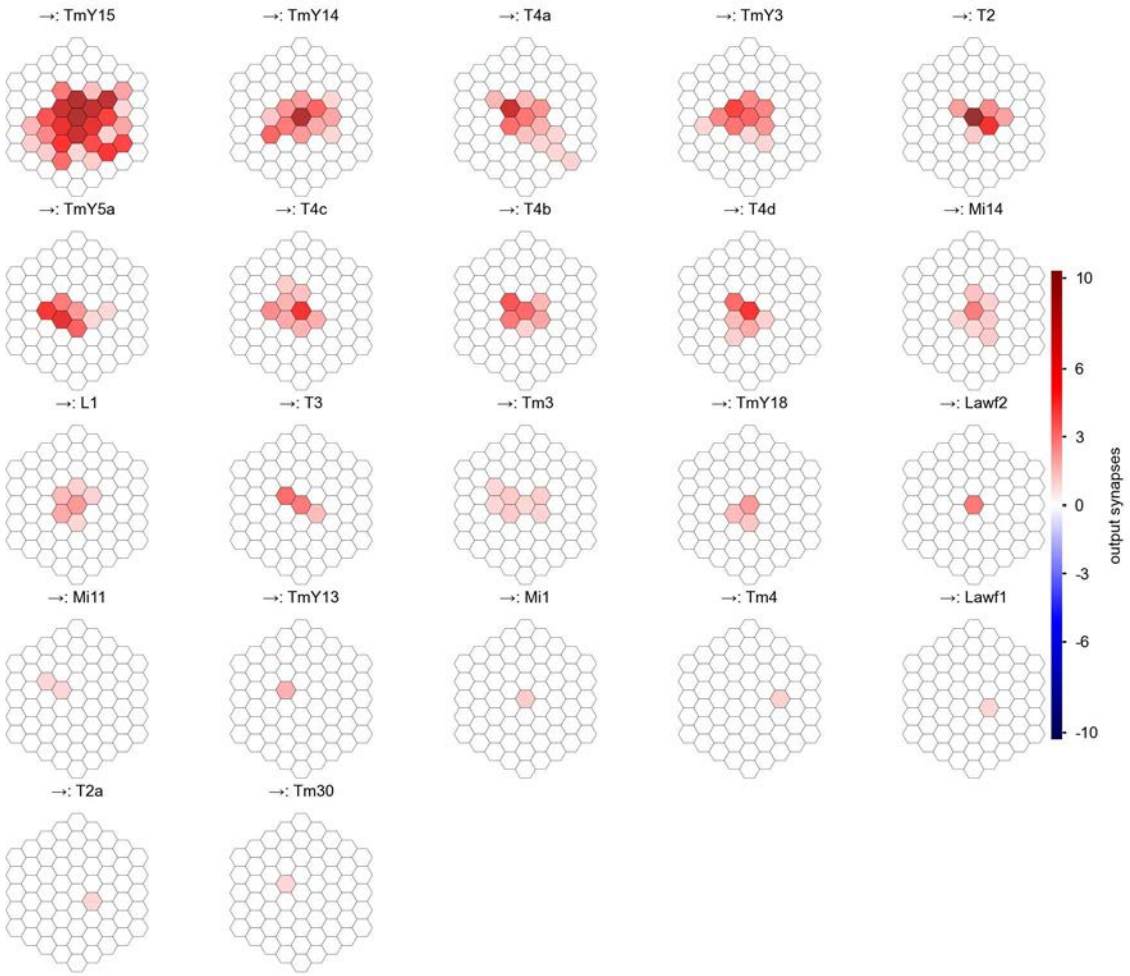

Tm3 - Figure 2: **Anatomical projective fields.** Each colored hexagon is an output connection, with the connection strength characterized by the average number of synapses that we count from the EM reconstruction. Red indicates excitatory synapses, blue indicates inhibitory synapses from inferred signs. Filters in the order of their total number of synapses.

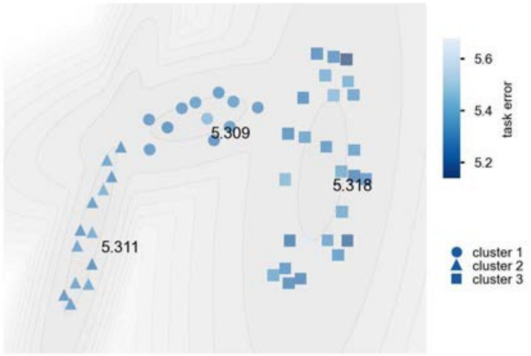

Tm3 - Figure 3: **Clustering of the responses to naturalistic stimuli.** Clustering of the 50 models based on the cell type responses to naturalistic scenes from the Sintel dataset. Scatterpoints represent individual models colored by their task error.

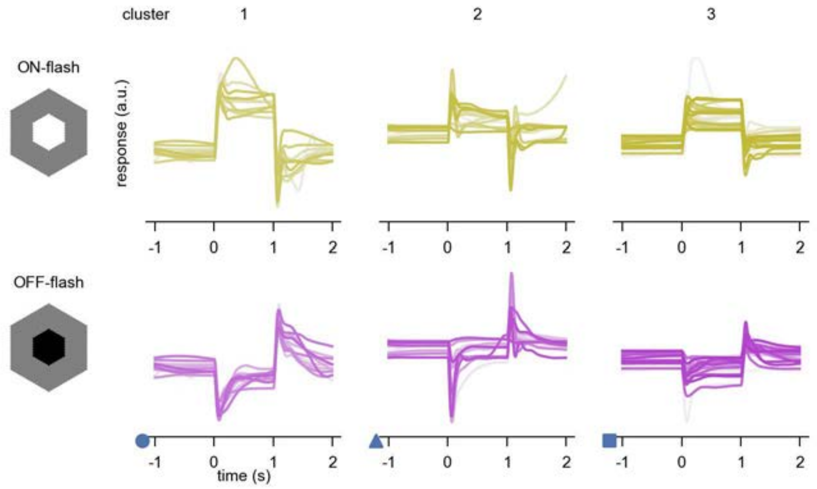

Tm3 - Figure 4: **Responses to flashes.** The top row shows responses to ON-flashes (yellow), the bottom row shows responses to OFF-flashes (magenta). The responses from the 50 different models that are separated into the different clusters (columns) overlay, with better task-performing models on top. Responses from better task-performing models are more saturated. The circular flashes (1s) cover 6 ommatidia in radius and are presented at time zero. Before and after, a grey-stimulus leads to a stationary state of the network.

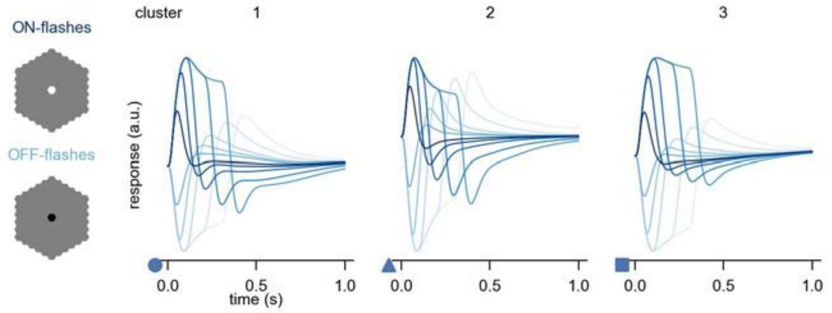

Tm3 - Figure 5: **Cluster-average responses to single-ommatidium flashes.** Responses to single-ommatidium ON-flashes (dark blue shades) and single-ommatidium OFF-flashes (light blue shades) of 20ms, 50ms, 100ms, 200ms, 300ms duration. The flashes occur at second zero.

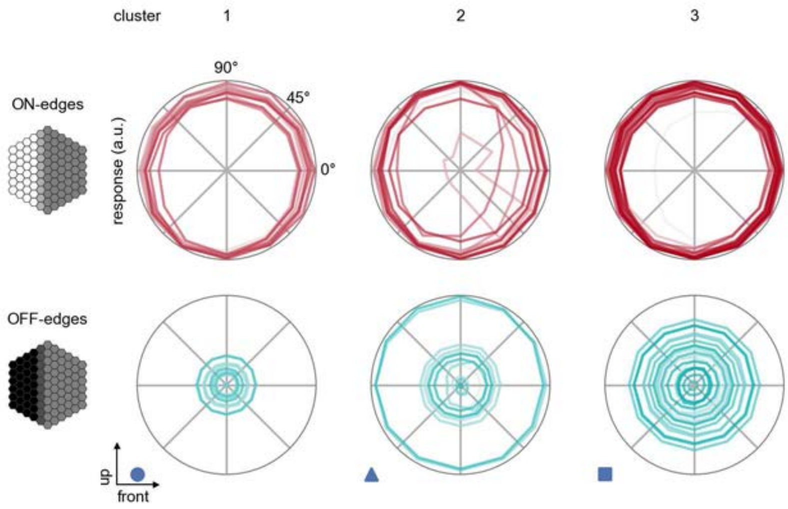

Tm3 - Figure 6: **Peak responses to moving edges.** The top row shows peak responses to moving ON-edges (red), the bottom row shows peak responses to moving OFF-edges (turqoise). The peak responses are averaged over edge-speeds. Edge-stimuli move in different directions from 0 to 360 degrees. The responses from the different models in the different clusters (columns) overlay. Responses from better task-performing models are more saturated.

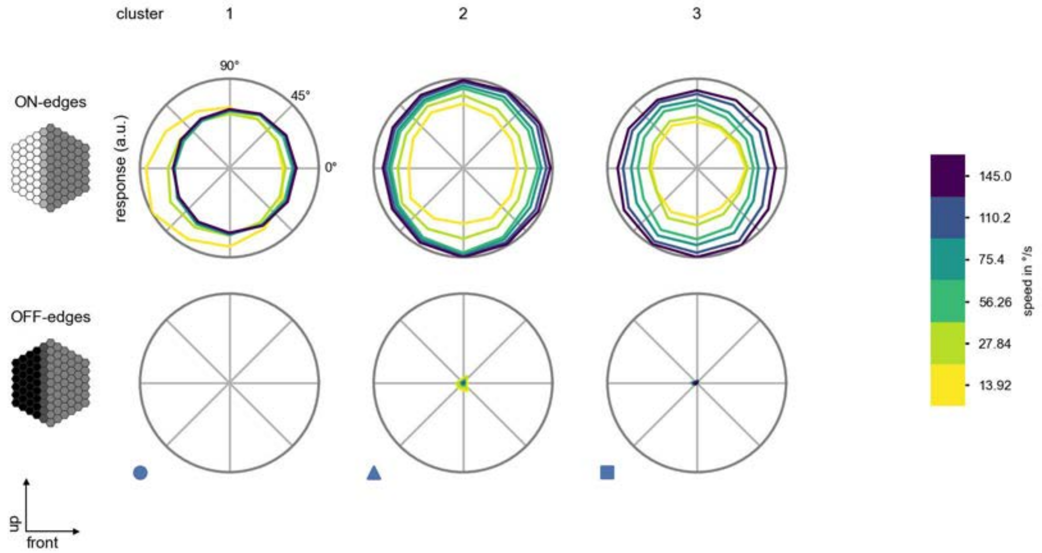

Tm3 - Figure 7: **Peak responses to moving edges from task-optimal models.** The top row shows peak responses to moving ON-edges, the bottom row shows peak responses to moving OFF-edges of varying speeds from 13.92°/s to 145°/s (yellow to dark blue). The edge-stimuli move in different directions from 0 to 360 degrees and at different speeds. Responses from the task-optimal model in the respective cluster.

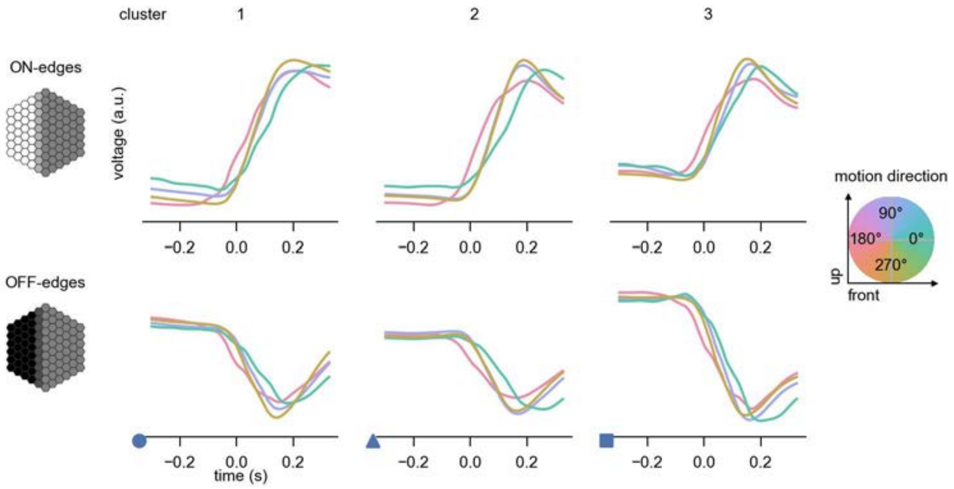

Tm3 - Figure 8: **Responses to moving edges from task-optimal models.** Responses to moving ON-edges (top row) and to moving OFF-edges (bottom row). Edges move in different directions from 0 to 360 degrees and at different speeds. Responses are from the task-optimal model in the respective cluster. Edges moving at 75.4°/s in all cardinal directions (green 0°, blue 90°, red 180°, yellow 270°) from -22.5 to 22.5° visual angle.

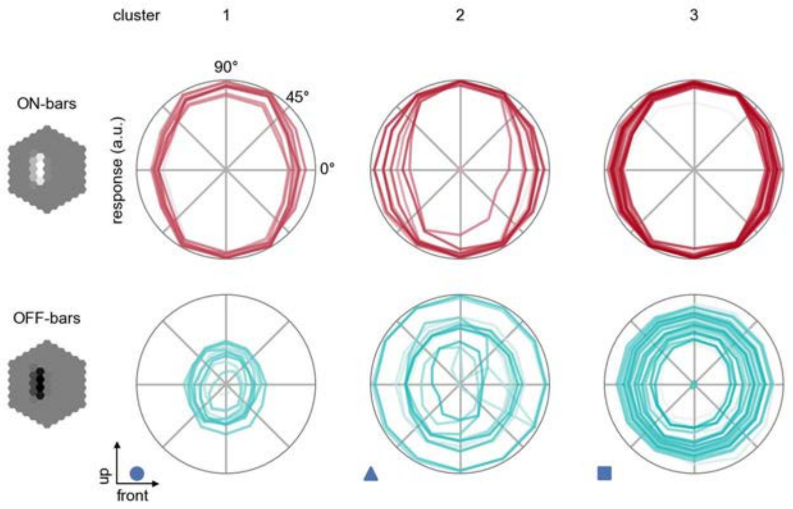

Tm3 - Figure 9: **Peak responses to moving bars.** The top row shows peak responses to moving ON-bars (red), the bottom row shows peak responses to moving OFF-bars (turqoise). The peak responses are averaged over bar-speeds. Bar-stimuli move in different directions from 0 to 360 degrees. The responses from the different models in the different clusters (columns) overlay. Responses from better task-performing models are more saturated.

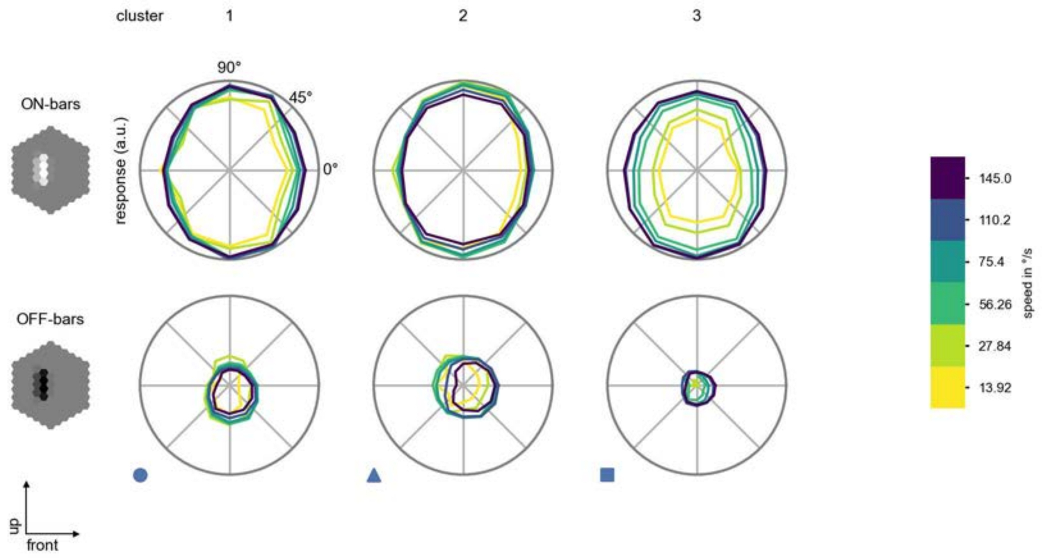

Tm3 - Figure 10: **Peak responses to moving bars from task-optimal models.** The top row shows peak responses to moving ON-bars, the bottom row shows peak responses to moving OFF-bars of varying speeds from 13.92°/s to 145°/s (yellow to dark blue). The bar-stimuli move in different directions from 0 to 360 degrees and at different speeds. Responses from the task-optimal model in the respective cluster.

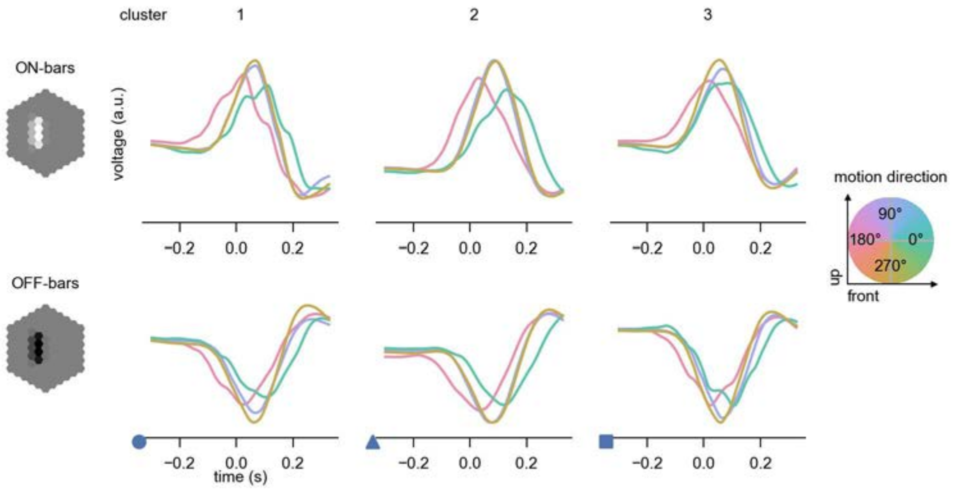

Tm3 - Figure 11: **Responses to moving bars from task-optimal models.** Responses to moving ON-bars (top row) and to moving OFF-bars (bottom row). Bars move in different directions from 0 to 360 degrees and at different speeds. Responses are from the task-optimal model in the respective cluster. Bars moving at 75.4°/s in all cardinal directions (green 0°, blue 90°, red 180°, yellow 270°) from -22.5 to 22.5° visual angle.

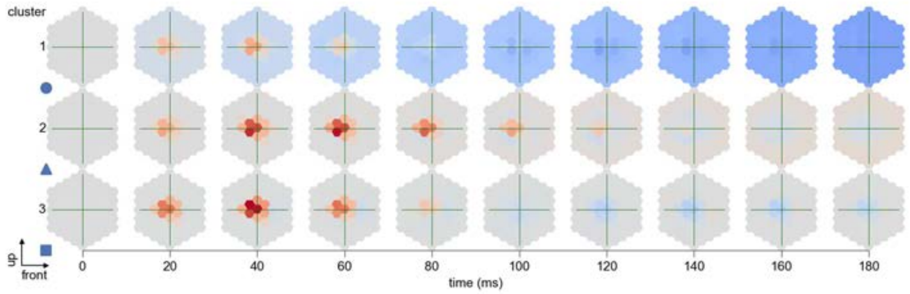

Tm3 - Figure 12: **Spatio-temporal receptive field.** Responses of the central cell to ON-impulses (5 ms) at single-ommatidium flash locations. The flash occurs at second zero. Responses from the task-optimal model of the respective cluster (rows). Red indicates depolarization, blue indicates hyperpolarization.

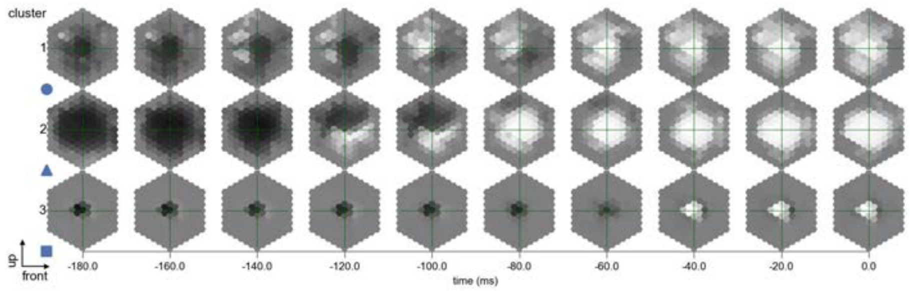

Tm3 - Figure 13: **Maximally excitatory stimuli.** Each row presents the regularized naturalistic-stimulus from the Sintel dataset that maximizes the cell type's central column response at second zero in the task-optimal model of the respective cluster (rows).

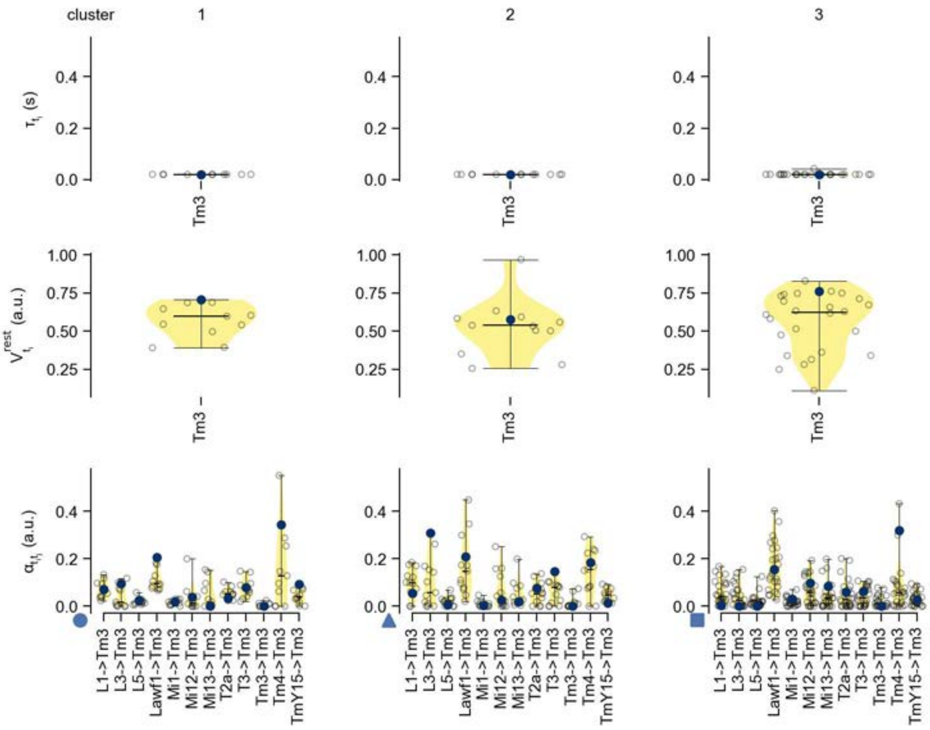

Tm3 - Figure 14: **Task-constrained parameters.** Each column shows the parameters inferred within the respective cluster. First row: learned time constants of the cell type. Second row: resting potentials of the cell type. Third row: scaling factors for the convolutional filters. The blue scatter represents the parameters from the task-optimal model within the cluster.

47 Tm4

← Cell types

Figures

1 Anatomical receptive fields. . . . 338
2 Anatomical projective fields. . . . 339
3 Clustering of the responses to naturalistic stimuli. . . . 339
4 Responses to flashes. . . . 340
5 Cluster-average responses to single-ommatidium flashes. . . . 340
6 Peak responses to moving edges. . . . 341
7 Peak responses to moving edges from task-optimal models. . . . 341
8 Responses to moving edges from task-optimal models. . . . 342
9 Peak responses to moving bars. . . . 342
10 Peak responses to moving bars from task-optimal models. . . . 343
11 Responses to moving bars from task-optimal models. . . . 343
12 Spatio-temporal receptive field. . . . 344
13 Maximally excitatory stimuli. . . . 344
14 Task-constrained parameters. . . . 345

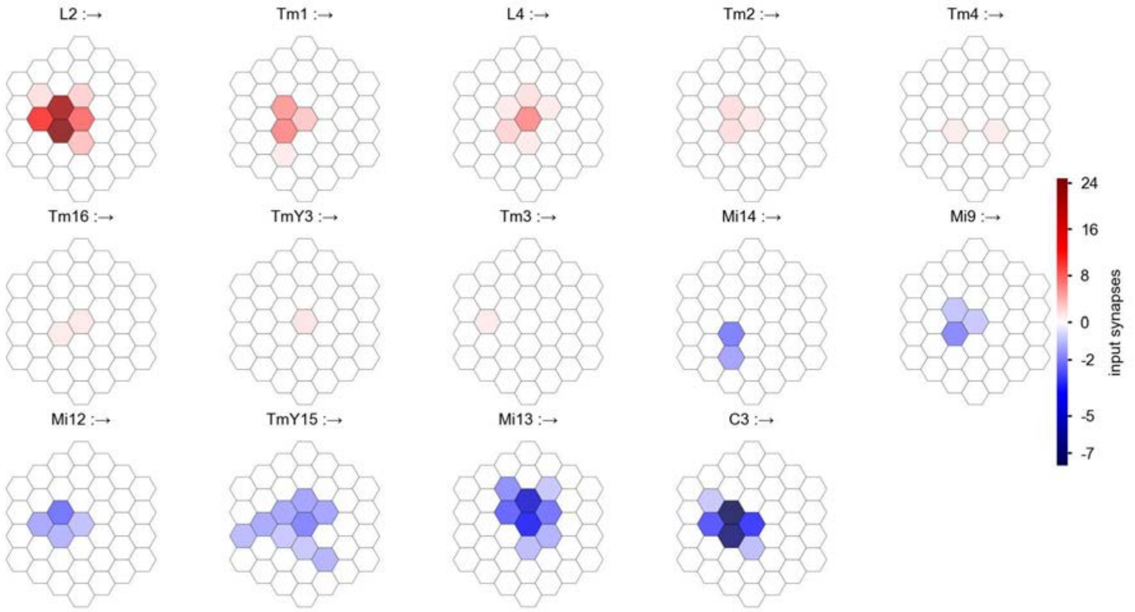

Tm4 - Figure 1: **Anatomical receptive fields.** Each colored hexagon is an input connection, with the connection strength characterized by the average number of synapses that we count from the EM reconstruction. Red indicates excitatory synapses, blue indicates inhibitory synapses from inferred signs. Filters in the order of their total number of synapses.

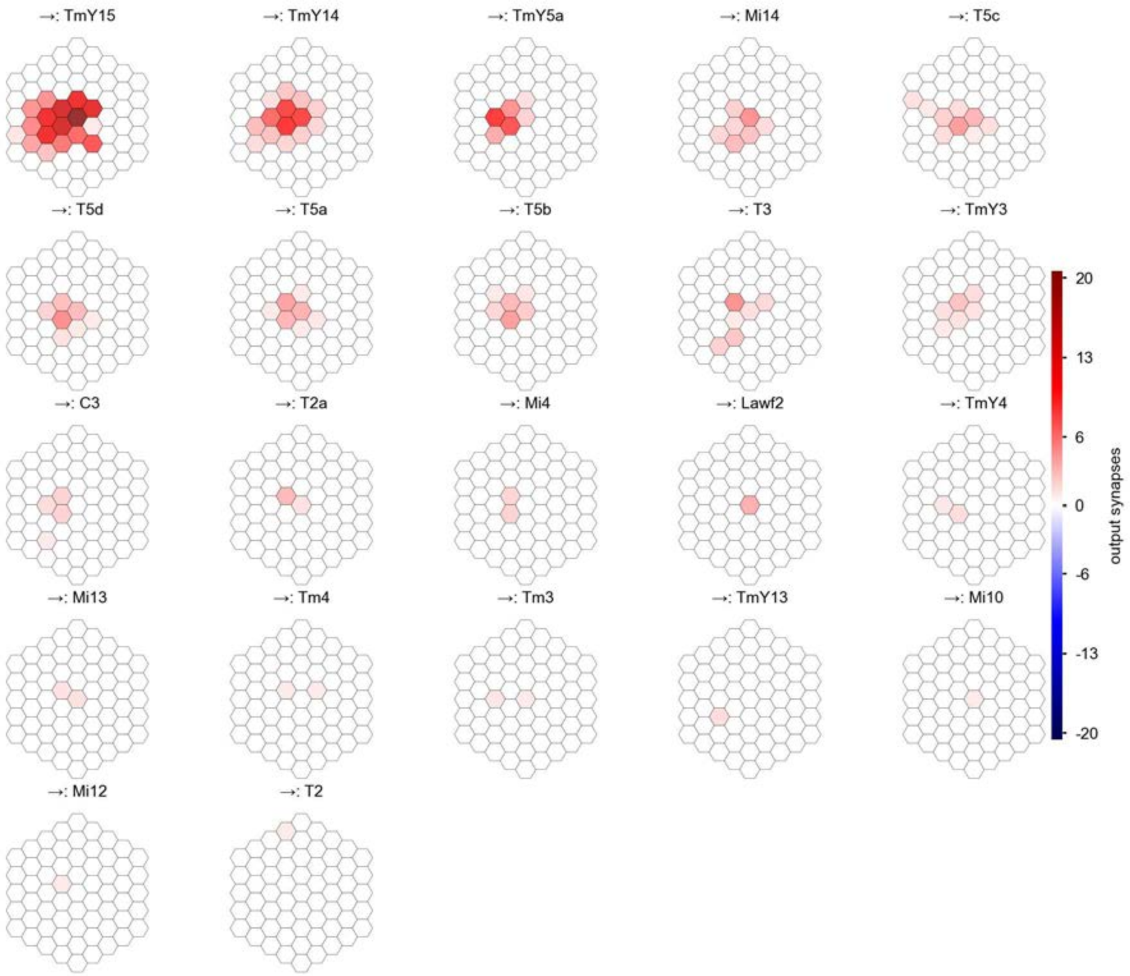

Tm4 - Figure 2: **Anatomical projective fields.** Each colored hexagon is an output connection, with the connection strength characterized by the average number of synapses that we count from the EM reconstruction. Red indicates excitatory synapses, blue indicates inhibitory synapses from inferred signs. Filters in the order of their total number of synapses.

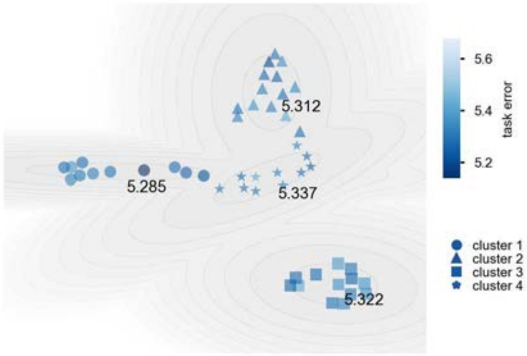

Tm4 - Figure 3: **Clustering of the responses to naturalistic stimuli.** Clustering of the 50 models based on the cell type responses to naturalistic scenes from the Sintel dataset. Scatterpoints represent individual models colored by their task error.

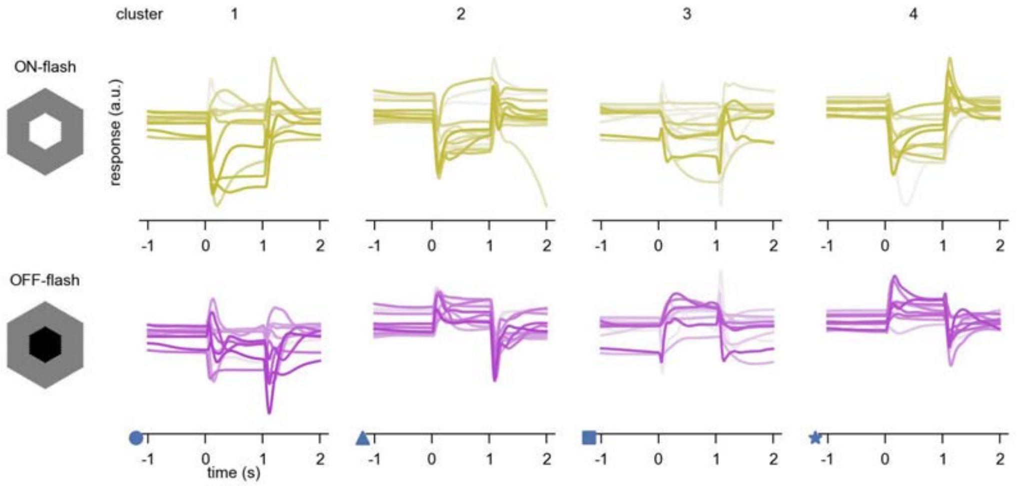

Tm4 - Figure 4: **Responses to flashes.** The top row shows responses to ON-flashes (yellow), the bottom row shows responses to OFF-flashes (magenta). The responses from the 50 different models that are separated into the different clusters (columns) overlay, with better task-performing models on top. Responses from better task-performing models are more saturated. The circular flashes (1s) cover 6 ommatidia in radius and are presented at time zero. Before and after, a grey-stimulus leads to a stationary state of the network.

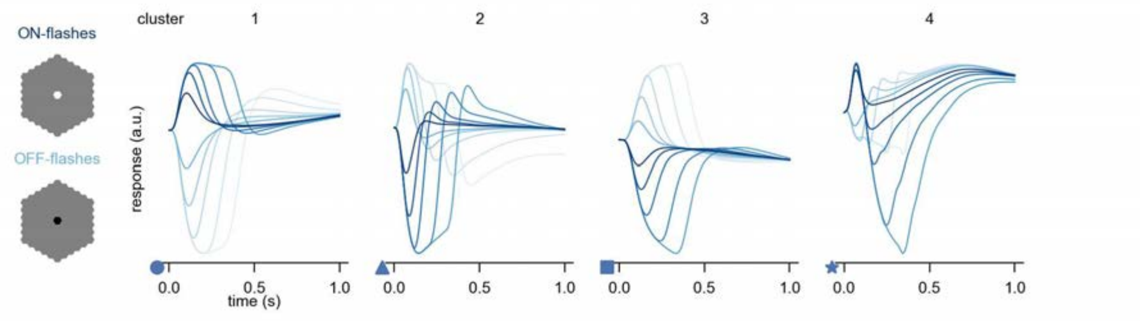

Tm4 - Figure 5: **Cluster-average responses to single-ommatidium flashes.** Responses to single-ommatidium ON-flashes (dark blue shades) and single-ommatidium OFF-flashes (light blue shades) of 20ms, 50ms, 100ms, 200ms, 300ms duration. The flashes occur at second zero.

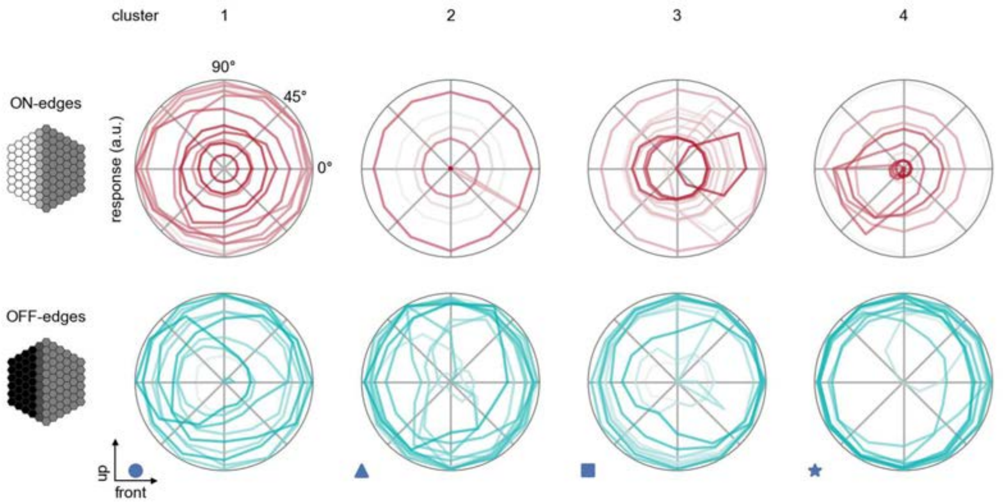

Tm4 - Figure 6: **Peak responses to moving edges.** The top row shows peak responses to moving ON-edges (red), the bottom row shows peak responses to moving OFF-edges (turqoise). The peak responses are averaged over edge-speeds. Edge-stimuli move in different directions from 0 to 360 degrees. The responses from the different models in the different clusters (columns) overlay. Responses from better task-performing models are more saturated.

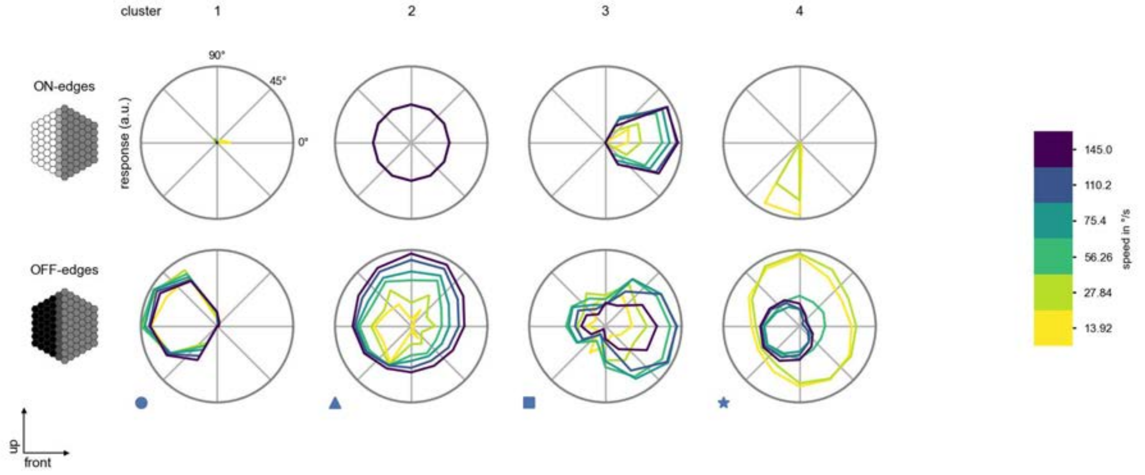

Tm4 - Figure 7: **Peak responses to moving edges from task-optimal models.** The top row shows peak responses to moving ON-edges, the bottom row shows peak responses to moving OFF-edges of varying speeds from 13.92°/s to 145°/s (yellow to dark blue). The edge-stimuli move in different directions from 0 to 360 degrees and at different speeds. Responses from the task-optimal model in the respective cluster.

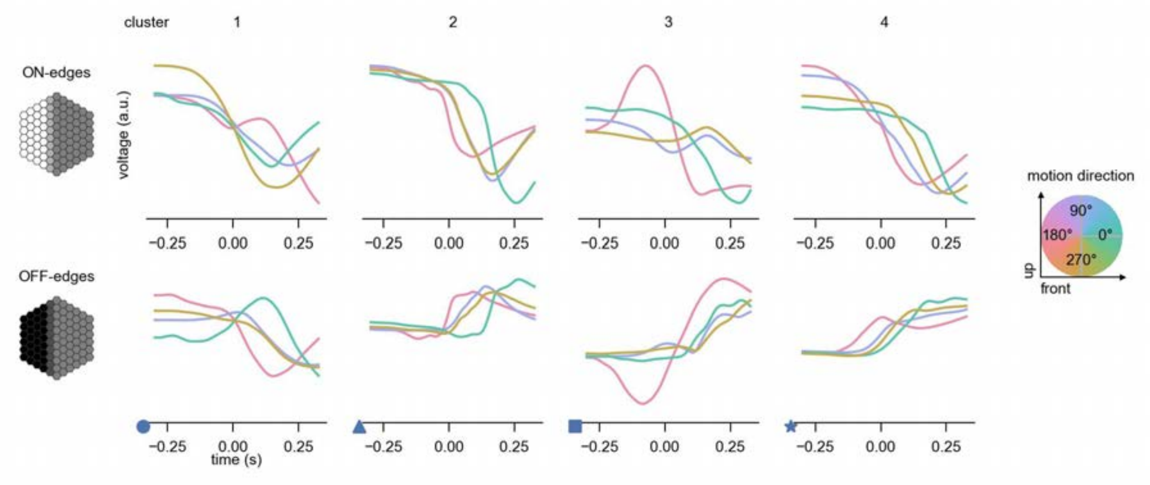

Tm4 - Figure 8: **Responses to moving edges from task-optimal models.** Responses to moving ON-edges (top row) and to moving OFF-edges (bottom row). Edges move in different directions from 0 to 360 degrees and at different speeds. Responses are from the task-optimal model in the respective cluster. Edges moving at 75.4°/s in all cardinal directions (green 0°, blue 90°, red 180°, yellow 270°) from -22.5 to 22.5° visual angle.

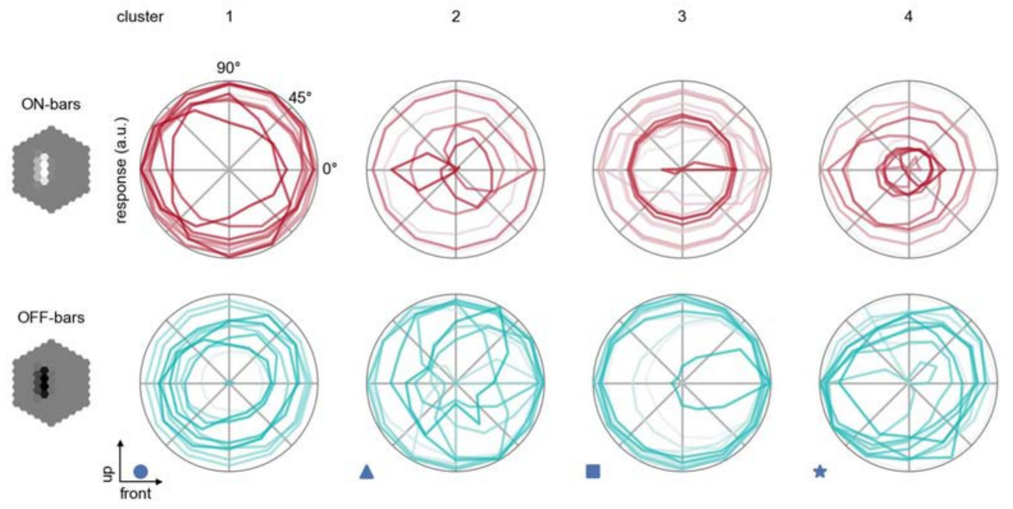

Tm4 - Figure 9: **Peak responses to moving bars.** The top row shows peak responses to moving ON-bars (red), the bottom row shows peak responses to moving OFF-bars (turqoise). The peak responses are averaged over bar-speeds. Bar-stimuli move in different directions from 0 to 360 degrees. The responses from the different models in the different clusters (columns) overlay. Responses from better task-performing models are more saturated.

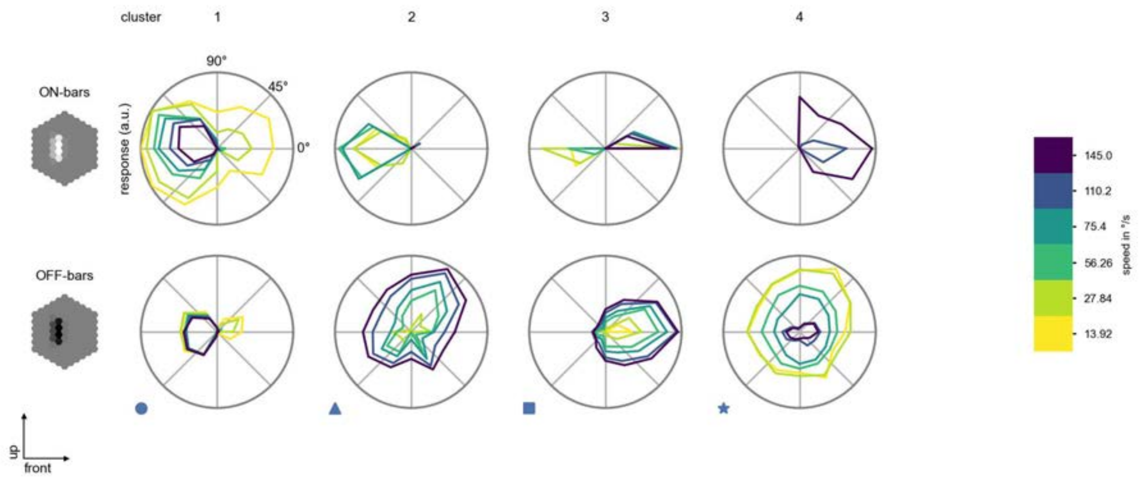

Tm4 - Figure 10: **Peak responses to moving bars from task-optimal models.** The top row shows peak responses to moving ON-bars, the bottom row shows peak responses to moving OFF-bars of varying speeds from 13.92°/s to 145°/s (yellow to dark blue). The bar-stimuli move in different directions from 0 to 360 degrees and at different speeds. Responses from the task-optimal model in the respective cluster.

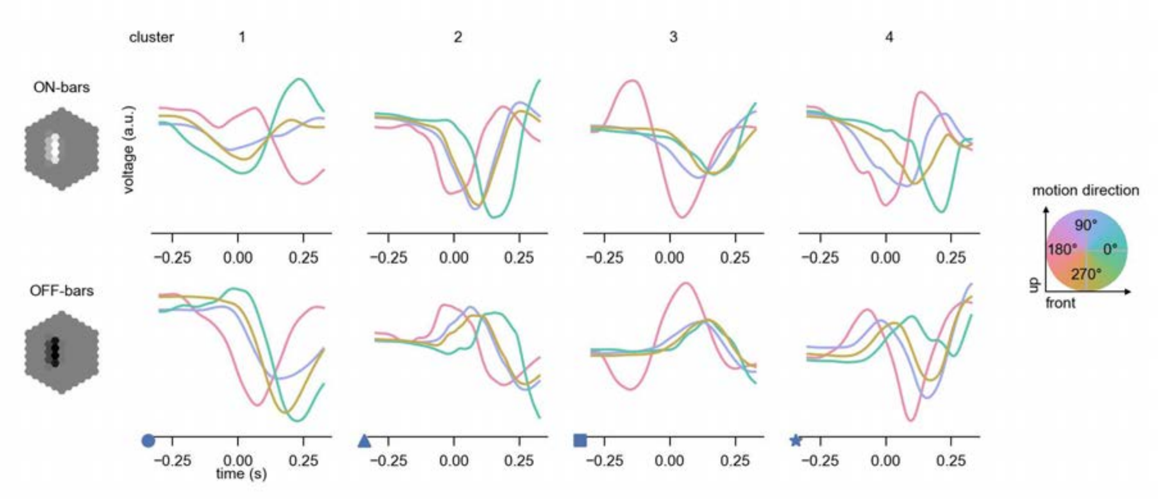

Tm4 - Figure 11: **Responses to moving bars from task-optimal models.** Responses to moving ON-bars (top row) and to moving OFF-bars (bottom row). Bars move in different directions from 0 to 360 degrees and at different speeds. Responses are from the task-optimal model in the respective cluster. Bars moving at 75.4°/s in all cardinal directions (green 0°, blue 90°, red 180°, yellow 270°) from -22.5 to 22.5° visual angle.

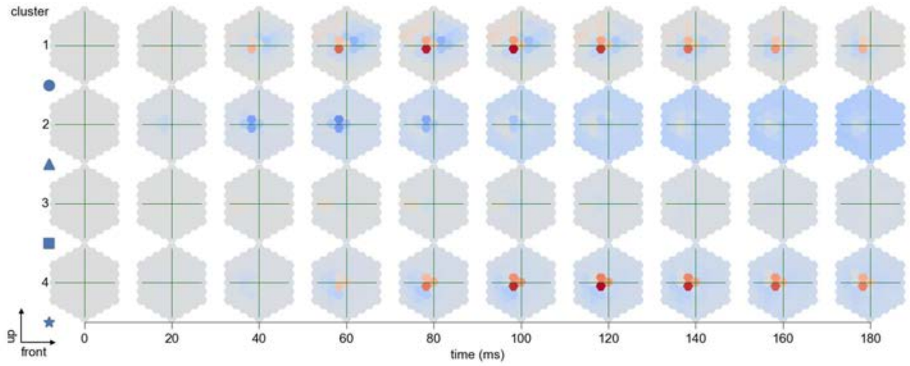

Tm4 - Figure 12: **Spatio-temporal receptive field.** Responses of the central cell to ON-impulses (5 ms) at single-ommatidium flash locations. The flash occurs at second zero. Responses from the task-optimal model of the respective cluster (rows). Red indicates depolarization, blue indicates hyperpolarization.

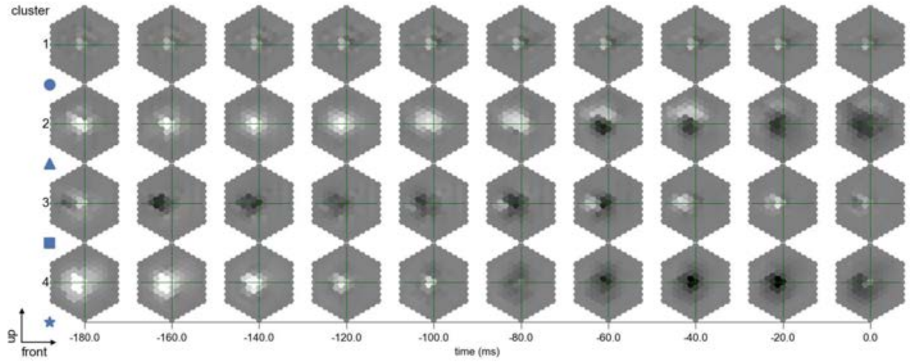

Tm4 - Figure 13: **Maximally excitatory stimuli.** Each row presents the regularized naturalistic-stimulus from the Sintel dataset that maximizes the cell type's central column response at second zero in the task-optimal model of the respective cluster (rows).

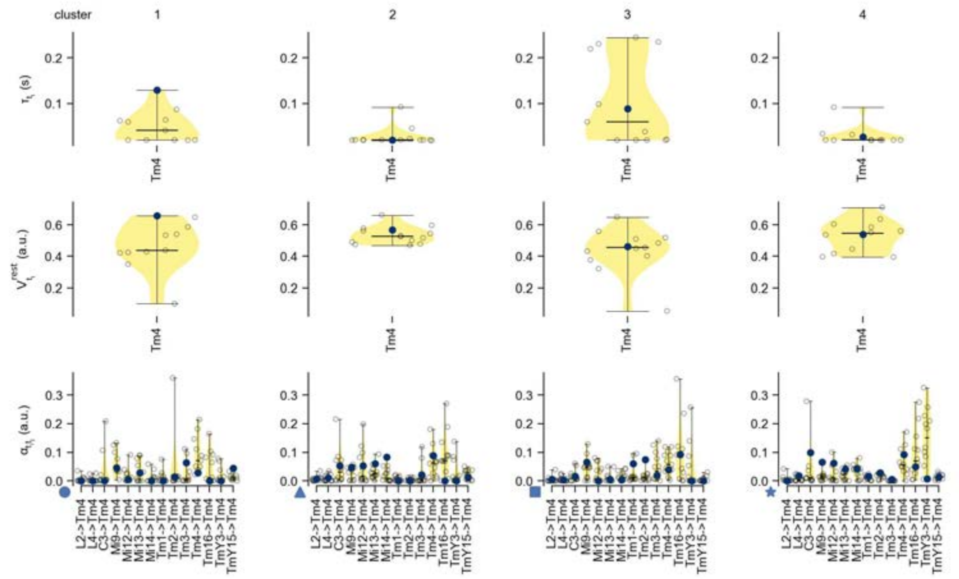

Tm4 - Figure 14: **Task-constrained parameters.** Each column shows the parameters inferred within the respective cluster. First row: learned time constants of the cell type. Second row: resting potentials of the cell type. Third row: scaling factors for the convolutional filters. The blue scatter represents the parameters from the task-optimal model within the cluster.

48 Tm5Y

← Cell types

Figures

1 Anatomical receptive fields. . . . 346
2 Anatomical projective fields. . . . 346
3 Clustering of the responses to naturalistic stimuli. . . . 347
4 Responses to flashes. . . . 347
5 Cluster-average responses to single-ommatidium flashes. . . . 347
6 Peak responses to moving edges. . . . 348
7 Peak responses to moving edges from task-optimal models. . . . 348
8 Responses to moving edges from task-optimal models. . . . 349
9 Peak responses to moving bars. . . . 349
10 Peak responses to moving bars from task-optimal models. . . . 350
11 Responses to moving bars from task-optimal models. . . . 350
12 Spatio-temporal receptive field. . . . 351
13 Maximally excitatory stimuli. . . . 351
14 Task-constrained parameters. . . . 352

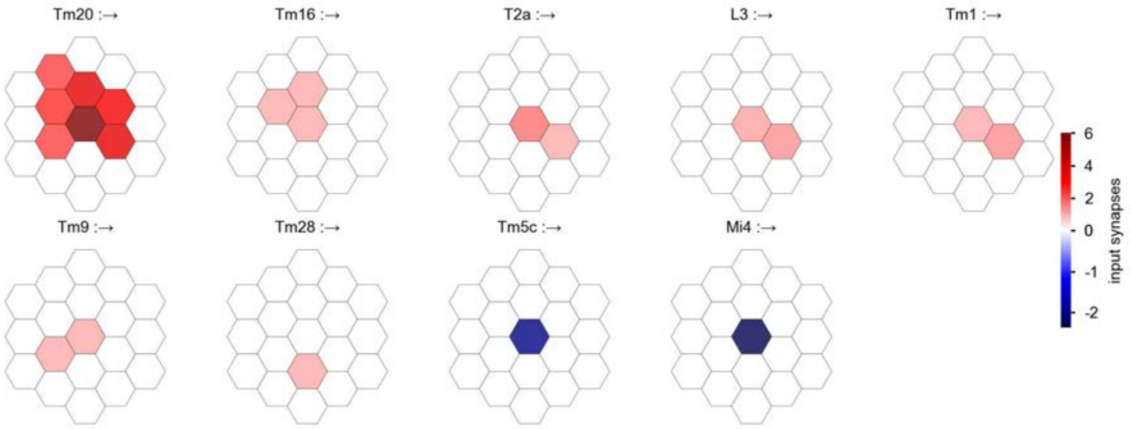

Tm5Y - Figure 1: **Anatomical receptive fields.** Each colored hexagon is an input connection, with the connection strength characterized by the average number of synapses that we count from the EM reconstruction. Red indicates excitatory synapses, blue indicates inhibitory synapses from inferred signs. Filters in the order of their total number of synapses.

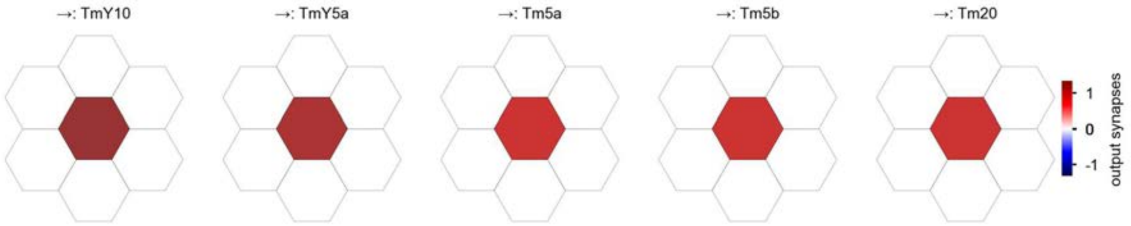

Tm5Y - Figure 2: **Anatomical projective fields.** Each colored hexagon is an output connection, with the connection strength characterized by the average number of synapses that we count from the EM reconstruction. Red indicates excitatory synapses, blue indicates inhibitory synapses from inferred signs. Filters in the order of their total number of synapses.

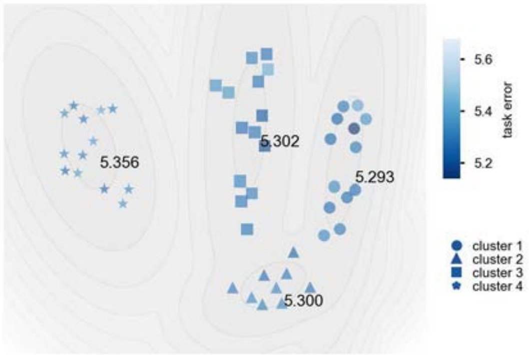

Tm5Y - Figure 3: **Clustering of the responses to naturalistic stimuli.** Clustering of the 50 models based on the cell type responses to naturalistic scenes from the Sintel dataset. Scatterpoints represent individual models colored by their task error.

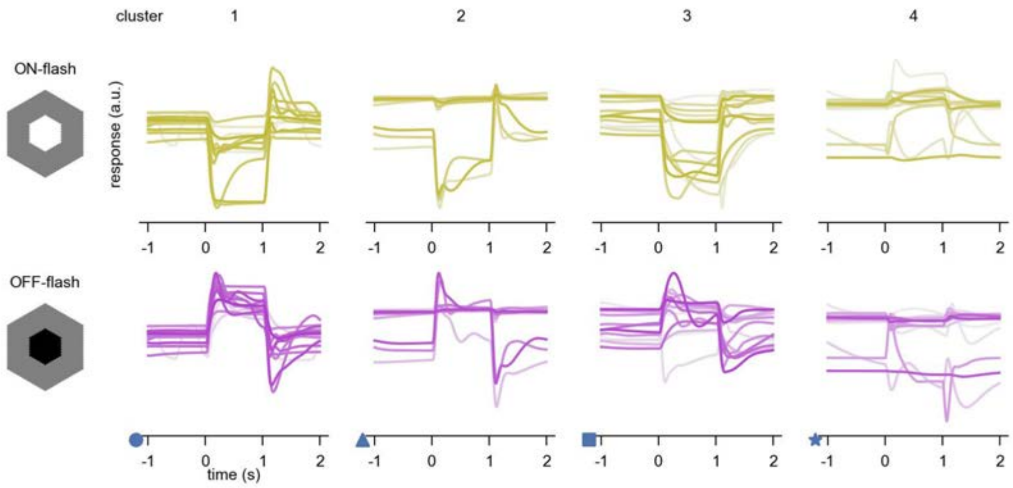

Tm5Y - Figure 4: **Responses to flashes.** The top row shows responses to ON-flashes (yellow), the bottom row shows responses to OFF-flashes (magenta). The responses from the 50 different models that are separated into the different clusters (columns) overlay, with better task-performing models on top. Responses from better task-performing models are more saturated. The circular flashes (1s) cover 6 ommatidia in radius and are presented at time zero. Before and after, a grey-stimulus leads to a stationary state of the network.

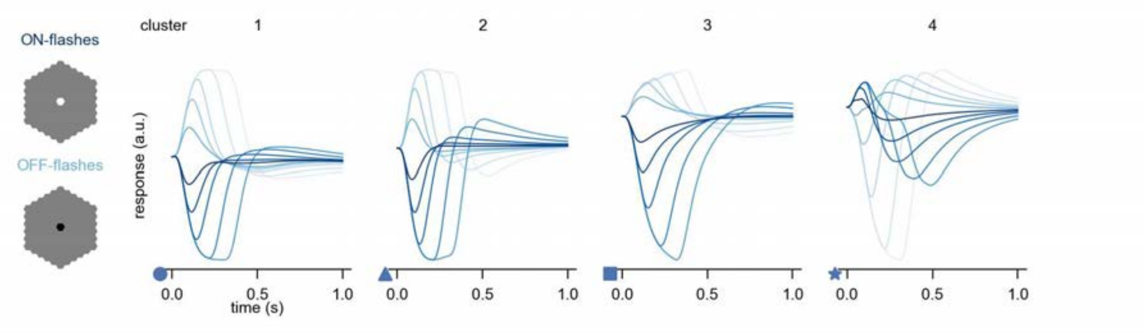

Tm5Y - Figure 5: **Cluster-average responses to single-ommatidium flashes.** Responses to single-ommatidium ON-flashes (dark blue shades) and single-ommatidium OFF-flashes (light blue shades) of 20ms, 50ms, 100ms, 200ms, 300ms duration. The flashes occur at second zero.

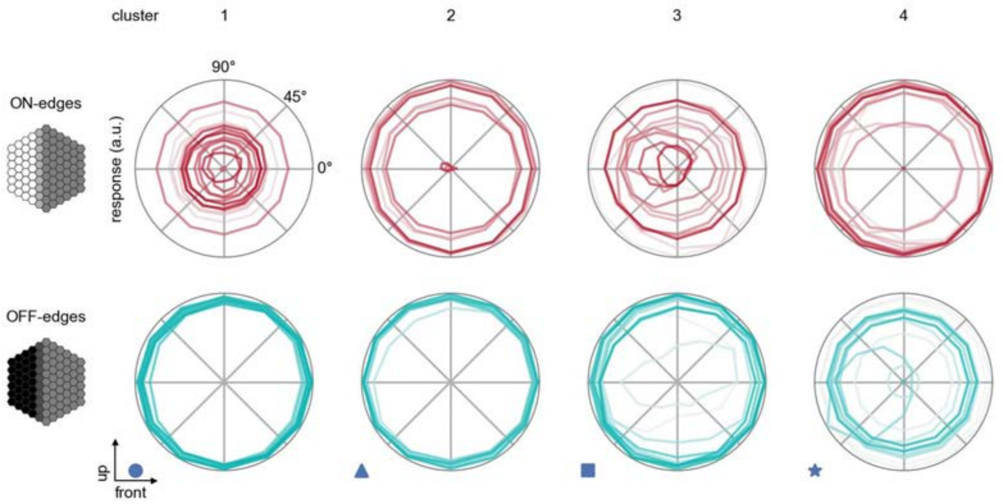

Tm5Y - Figure 6: **Peak responses to moving edges.** The top row shows peak responses to moving ON-edges (red), the bottom row shows peak responses to moving OFF-edges (turqoise). The peak responses are averaged over edge-speeds. Edge-stimuli move in different directions from 0 to 360 degrees. The responses from the different models in the different clusters (columns) overlay. Responses from better task-performing models are more saturated.

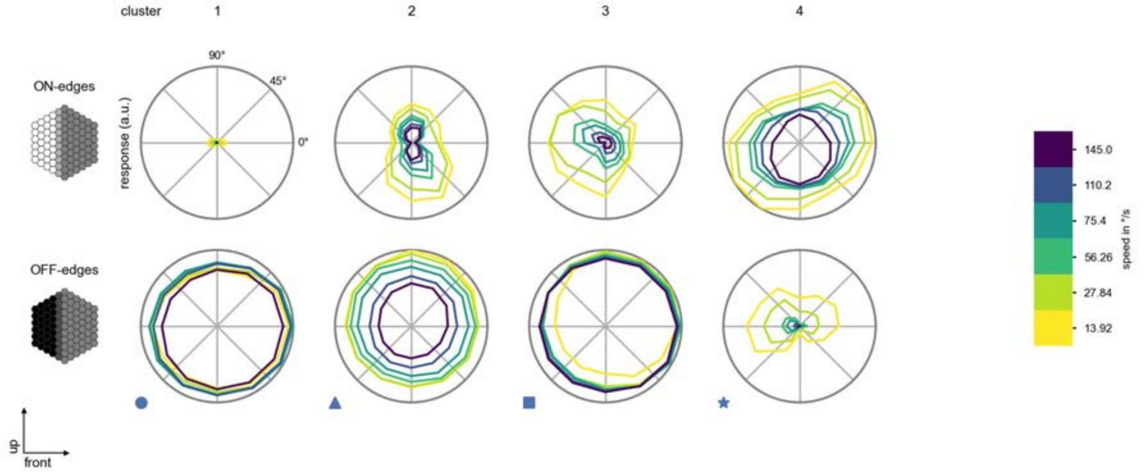

Tm5Y - Figure 7: **Peak responses to moving edges from task-optimal models.** The top row shows peak responses to moving ON-edges, the bottom row shows peak responses to moving OFF-edges of varying speeds from 13.92°/s to 145°/s (yellow to dark blue). The edge-stimuli move in different directions from 0 to 360 degrees and at different speeds. Responses from the task-optimal model in the respective cluster.

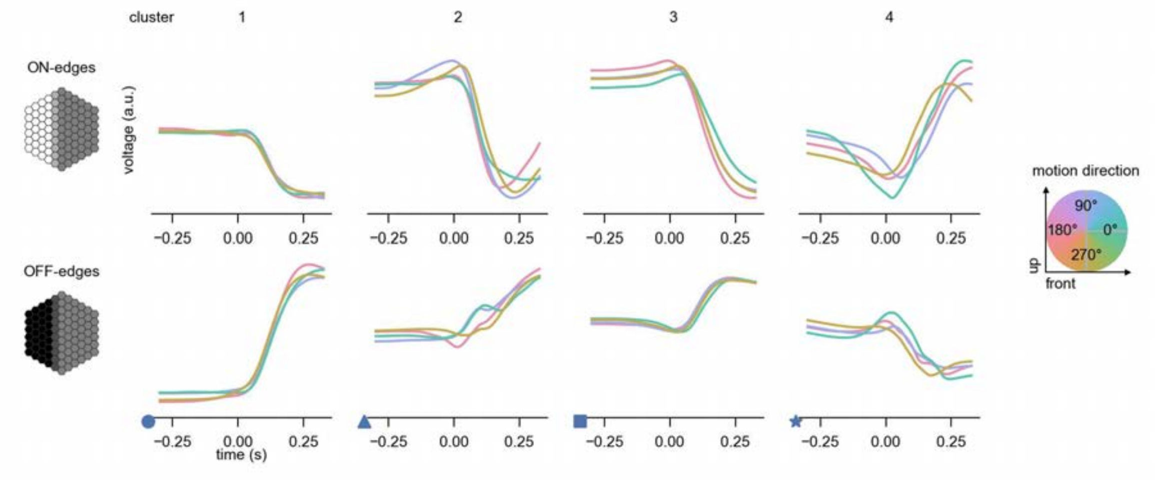

Tm5Y - Figure 8: **Responses to moving edges from task-optimal models.** Responses to moving ON-edges (top row) and to moving OFF-edges (bottom row). Edges move in different directions from 0 to 360 degrees and at different speeds. Responses are from the task-optimal model in the respective cluster. Edges moving at 75.4°/s in all cardinal directions (green 0°, blue 90°, red 180°, yellow 270°) from -22.5 to 22.5° visual angle.

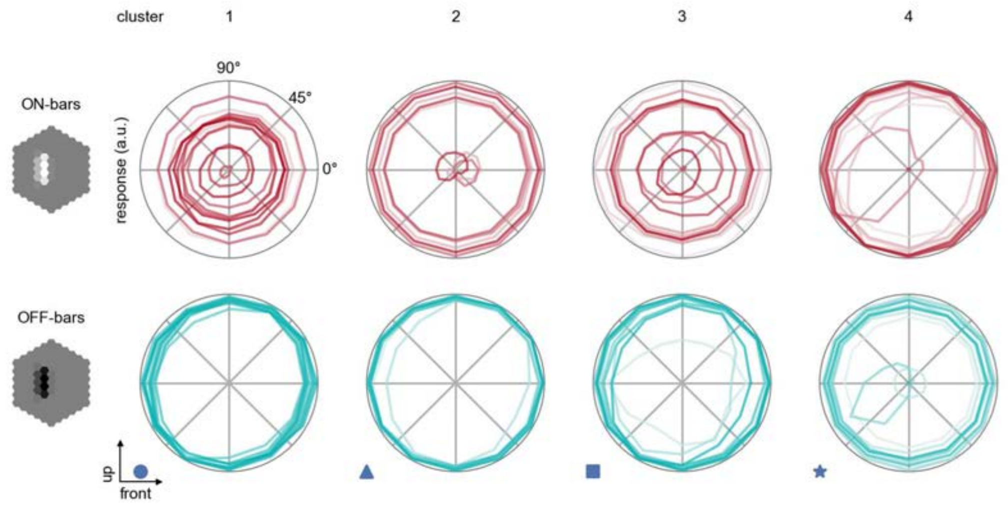

Tm5Y - Figure 9: **Peak responses to moving bars.** The top row shows peak responses to moving ON-bars (red), the bottom row shows peak responses to moving OFF-bars (turqoise). The peak responses are averaged over bar-speeds. Bar-stimuli move in different directions from 0 to 360 degrees. The responses from the different models in the different clusters (columns) overlay. Responses from better task-performing models are more saturated.

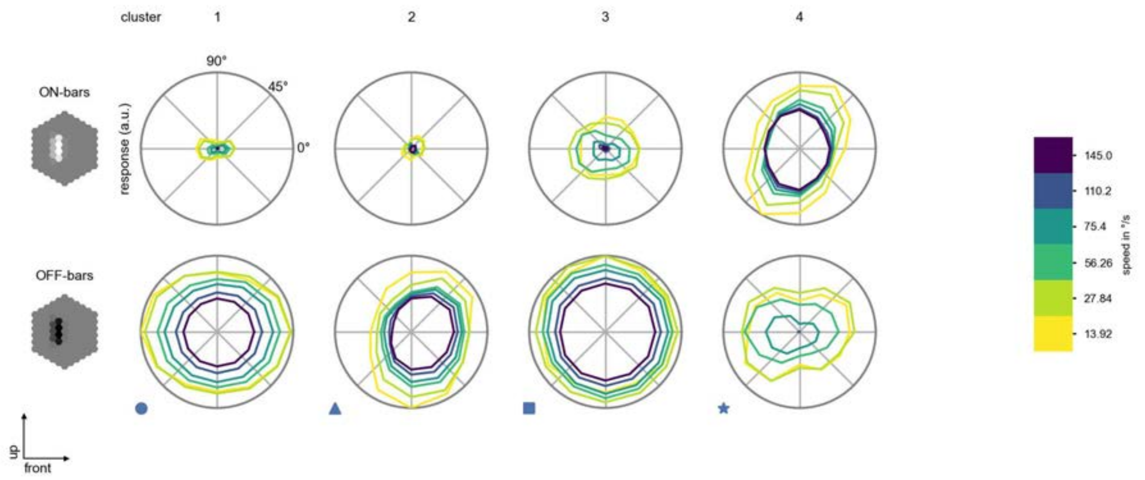

Tm5Y - Figure 10: **Peak responses to moving bars from task-optimal models.** The top row shows peak responses to moving ON-bars, the bottom row shows peak responses to moving OFF-bars of varying speeds from 13.92°/s to 145°/s (yellow to dark blue). The bar-stimuli move in different directions from 0 to 360 degrees and at different speeds. Responses from the task-optimal model in the respective cluster.

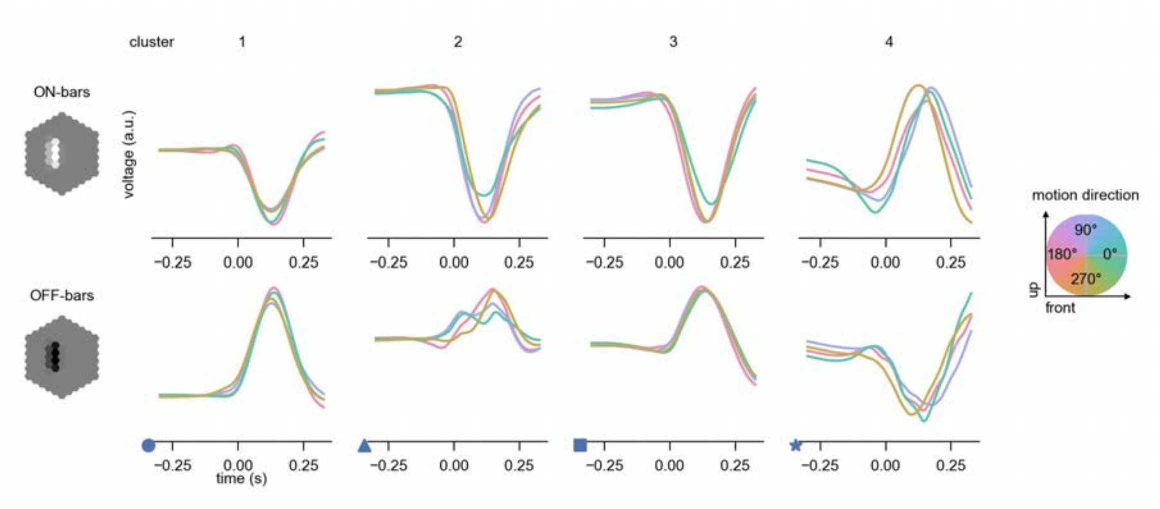

Tm5Y - Figure 11: **Responses to moving bars from task-optimal models.** Responses to moving ON-bars (top row) and to moving OFF-bars (bottom row). Bars move in different directions from 0 to 360 degrees and at different speeds. Responses are from the task-optimal model in the respective cluster. Bars moving at 75.4°/s in all cardinal directions (green 0°, blue 90°, red 180°, yellow 270°) from -22.5 to 22.5° visual angle.

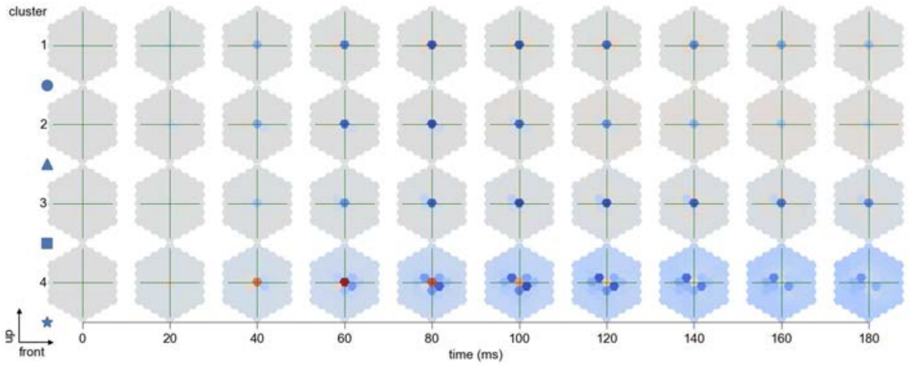

Tm5Y - Figure 12: **Spatio-temporal receptive field.** Responses of the central cell to ON-impulses (5 ms) at single-ommatidium flash locations. The flash occurs at second zero. Responses from the task-optimal model of the respective cluster (rows). Red indicates depolarization, blue indicates hyperpolarization.

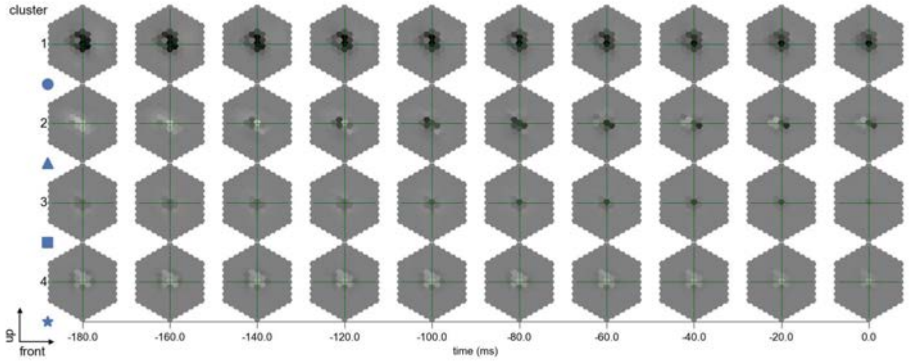

Tm5Y - Figure 13: **Maximally excitatory stimuli.** Each row presents the regularized naturalistic-stimulus from the Sintel dataset that maximizes the cell type's central column response at second zero in the task-optimal model of the respective cluster (rows).

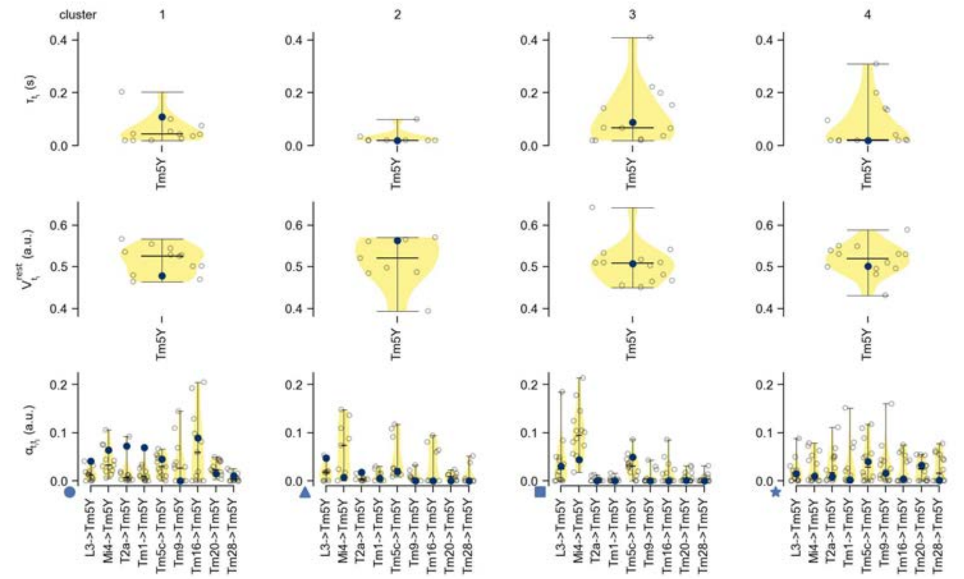

Tm5Y - Figure 14: **Task-constrained parameters.** Each column shows the parameters inferred within the respective cluster. First row: learned time constants of the cell type. Second row: resting potentials of the cell type. Third row: scaling factors for the convolutional filters. The blue scatter represents the parameters from the task-optimal model within the cluster.

49 Tm5a

← Cell types

Figures

1 Anatomical receptive fields. . . . 353
2 Anatomical projective fields. . . . 353
3 Clustering of the responses to naturalistic stimuli. . . . 354
4 Responses to flashes. . . . 354
5 Cluster-average responses to single-ommatidium flashes. . . . 354
6 Peak responses to moving edges. . . . 355
7 Peak responses to moving edges from task-optimal models. . . . 355
8 Responses to moving edges from task-optimal models. . . . 356
9 Peak responses to moving bars. . . . 356
10 Peak responses to moving bars from task-optimal models. . . . 357
11 Responses to moving bars from task-optimal models. . . . 357
12 Spatio-temporal receptive field. . . . 358
13 Maximally excitatory stimuli. . . . 358
14 Task-constrained parameters. . . . 359

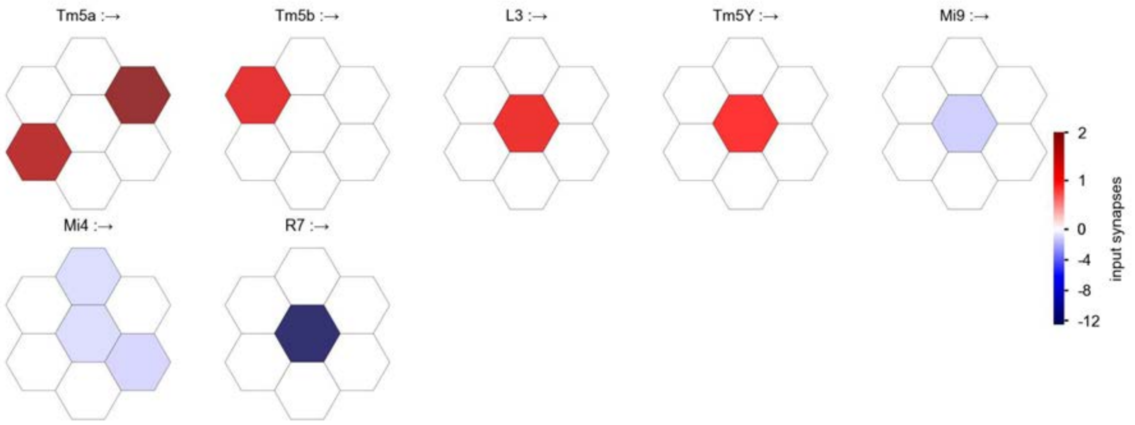

Tm5a - Figure 1: **Anatomical receptive fields.** Each colored hexagon is an input connection, with the connection strength characterized by the average number of synapses that we count from the EM reconstruction. Red indicates excitatory synapses, blue indicates inhibitory synapses from inferred signs. Filters in the order of their total number of synapses.

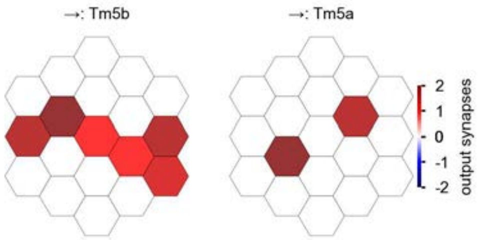

Tm5a - Figure 2: **Anatomical projective fields.** Each colored hexagon is an output connection, with the connection strength characterized by the average number of synapses that we count from the EM reconstruction. Red indicates excitatory synapses, blue indicates inhibitory synapses from inferred signs. Filters in the order of their total number of synapses.

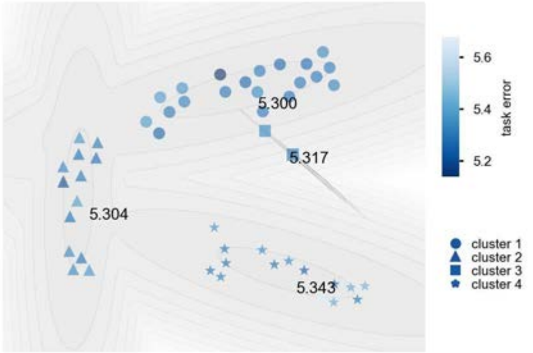

Tm5a - Figure 3: **Clustering of the responses to naturalistic stimuli.** Clustering of the 50 models based on the cell type responses to naturalistic scenes from the Sintel dataset. Scatterpoints represent individual models colored by their task error.

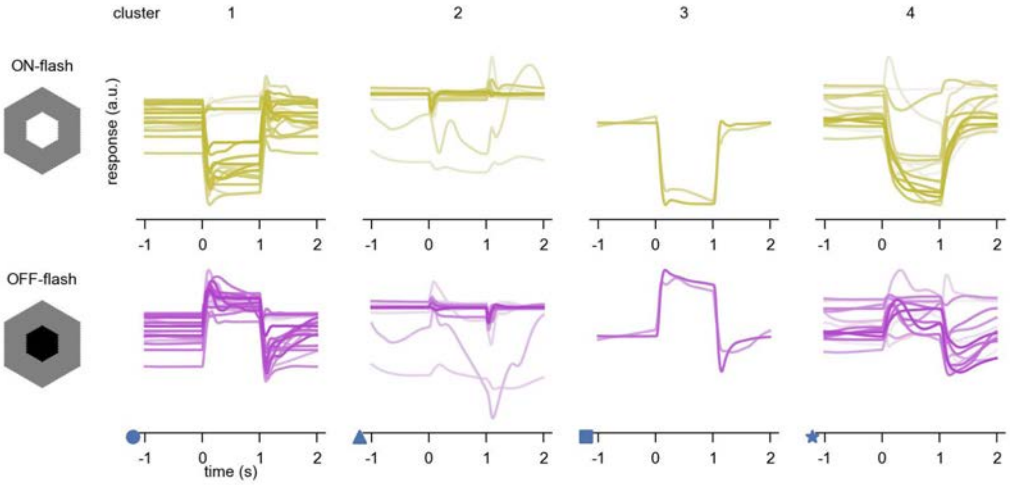

Tm5a - Figure 4: **Responses to flashes.** The top row shows responses to ON-flashes (yellow), the bottom row shows responses to OFF-flashes (magenta). The responses from the 50 different models that are separated into the different clusters (columns) overlay, with better task-performing models on top. Responses from better task-performing models are more saturated. The circular flashes (1s) cover 6 ommatidia in radius and are presented at time zero. Before and after, a grey-stimulus leads to a stationary state of the network.

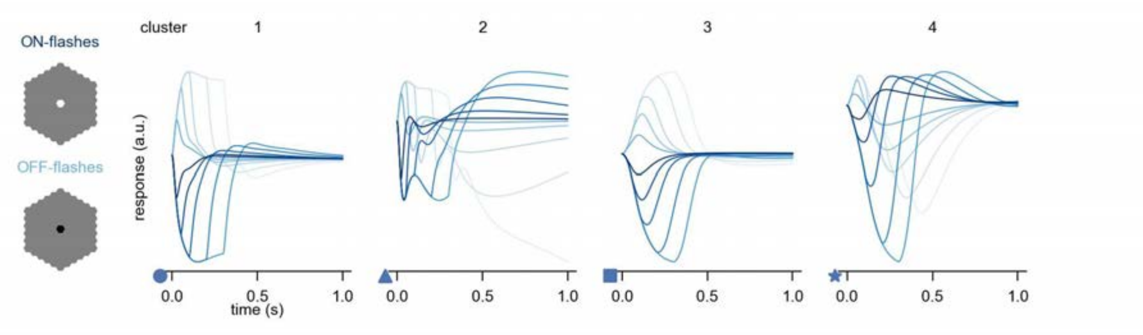

Tm5a - Figure 5: **Cluster-average responses to single-ommatidium flashes.** Responses to single-ommatidium ON-flashes (dark blue shades) and single-ommatidium OFF-flashes (light blue shades) of 20ms, 50ms, 100ms, 200ms, 300ms duration. The flashes occur at second zero.

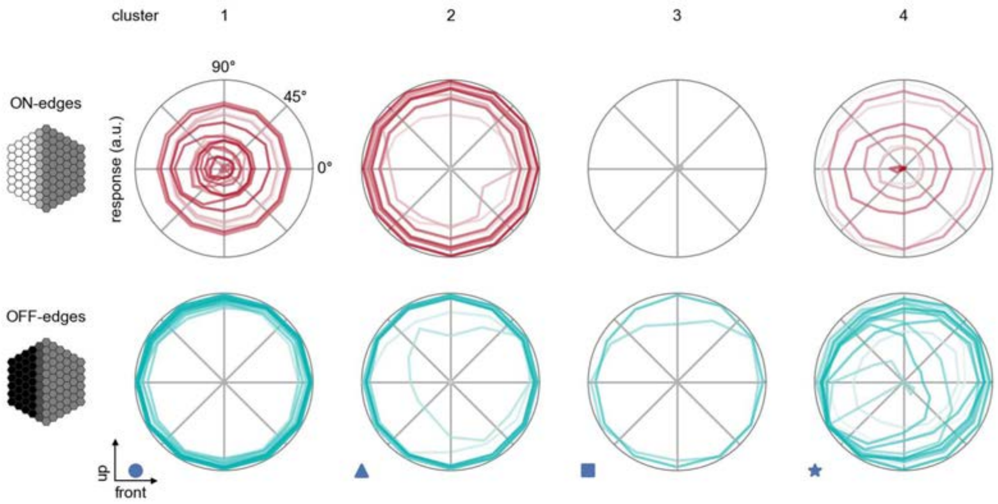

Tm5a - Figure 6: **Peak responses to moving edges.** The top row shows peak responses to moving ON-edges (red), the bottom row shows peak responses to moving OFF-edges (turqoise). The peak responses are averaged over edge-speeds. Edge-stimuli move in different directions from 0 to 360 degrees. The responses from the different models in the different clusters (columns) overlay. Responses from better task-performing models are more saturated.

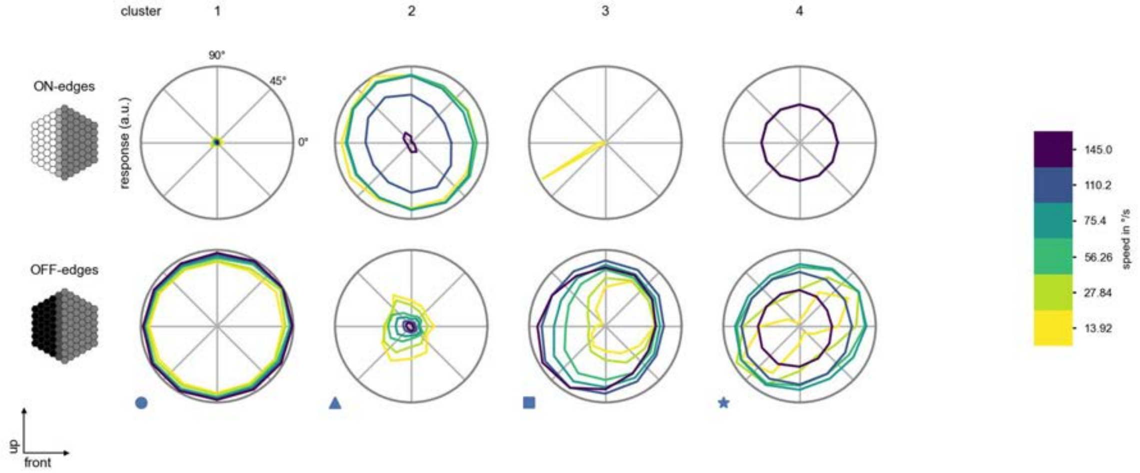

Tm5a - Figure 7: **Peak responses to moving edges from task-optimal models.** The top row shows peak responses to moving ON-edges, the bottom row shows peak responses to moving OFF-edges of varying speeds from 13.92°/s to 145°/s (yellow to dark blue). The edge-stimuli move in different directions from 0 to 360 degrees and at different speeds. Responses from the task-optimal model in the respective cluster.

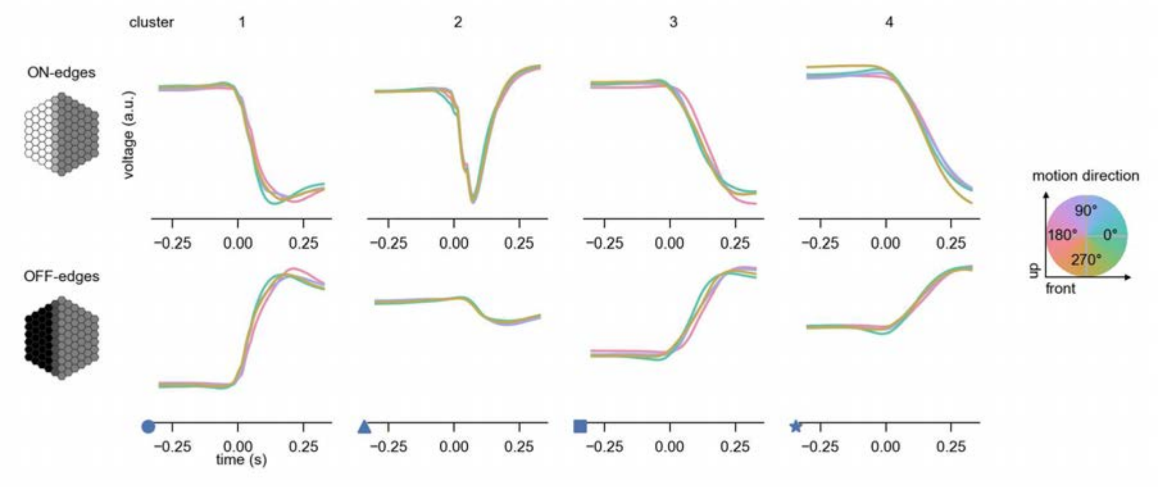

Tm5a - Figure 8: **Responses to moving edges from task-optimal models.** Responses to moving ON-edges (top row) and to moving OFF-edges (bottom row). Edges move in different directions from 0 to 360 degrees and at different speeds. Responses are from the task-optimal model in the respective cluster. Edges moving at 75.4°/s in all cardinal directions (green 0°, blue 90°, red 180°, yellow 270°) from -22.5 to 22.5° visual angle.

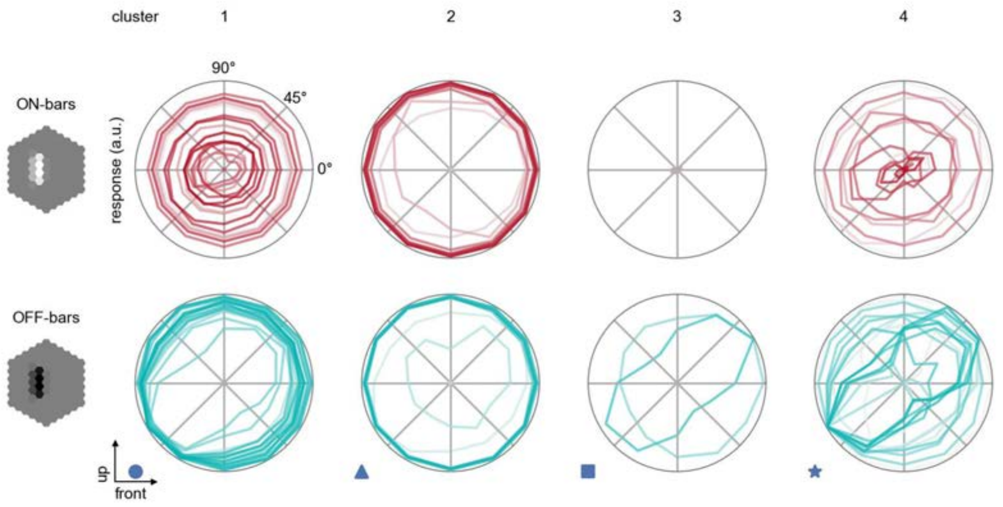

Tm5a - Figure 9: **Peak responses to moving bars.** The top row shows peak responses to moving ON-bars (red), the bottom row shows peak responses to moving OFF-bars (turqoise). The peak responses are averaged over bar-speeds. Bar-stimuli move in different directions from 0 to 360 degrees. The responses from the different models in the different clusters (columns) overlay. Responses from better task-performing models are more saturated.

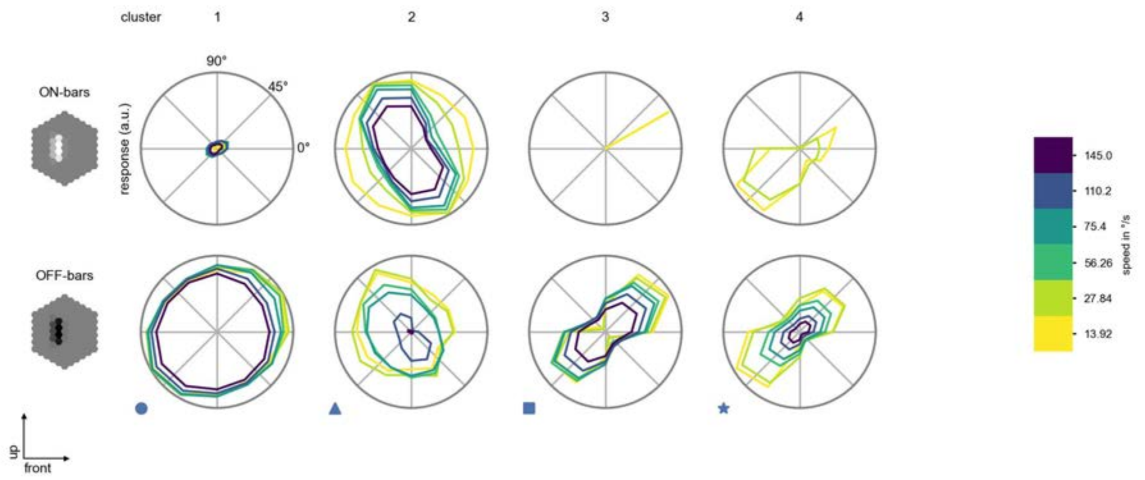

Tm5a - Figure 10: **Peak responses to moving bars from task-optimal models.** The top row shows peak responses to moving ON-bars, the bottom row shows peak responses to moving OFF-bars of varying speeds from 13.92°/s to 145°/s (yellow to dark blue). The bar-stimuli move in different directions from 0 to 360 degrees and at different speeds. Responses from the task-optimal model in the respective cluster.

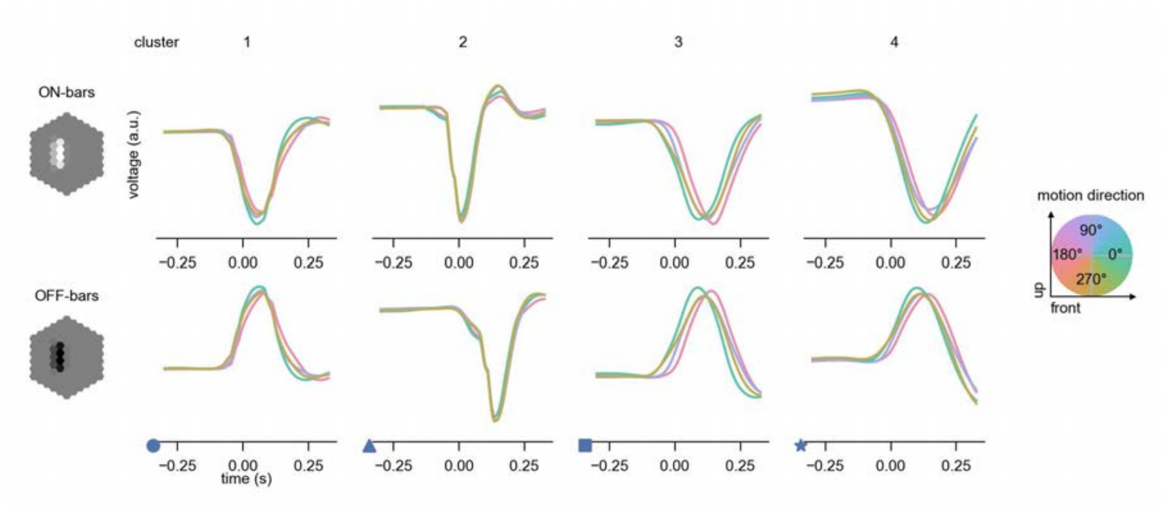

Tm5a - Figure 11: **Responses to moving bars from task-optimal models.** Responses to moving ON-bars (top row) and to moving OFF-bars (bottom row). Bars move in different directions from 0 to 360 degrees and at different speeds. Responses are from the task-optimal model in the respective cluster. Bars moving at 75.4°/s in all cardinal directions (green 0°, blue 90°, red 180°, yellow 270°) from -22.5 to 22.5° visual angle.

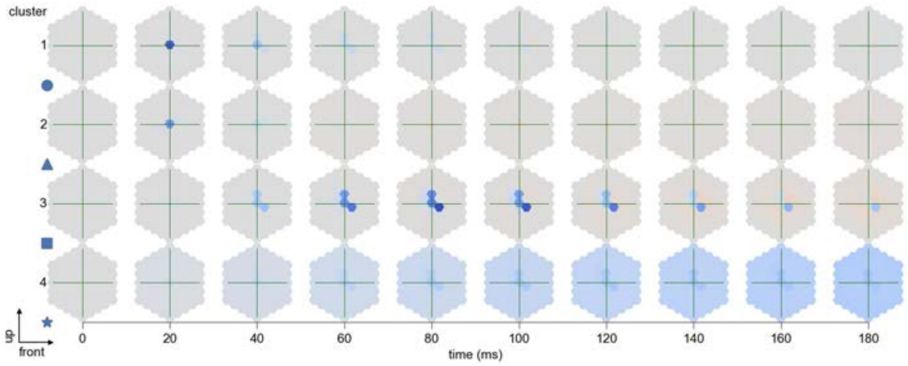

Tm5a - Figure 12: **Spatio-temporal receptive field.** Responses of the central cell to ON-impulses (5 ms) at single-ommatidium flash locations. The flash occurs at second zero. Responses from the task-optimal model of the respective cluster (rows). Red indicates depolarization, blue indicates hyperpolarization.

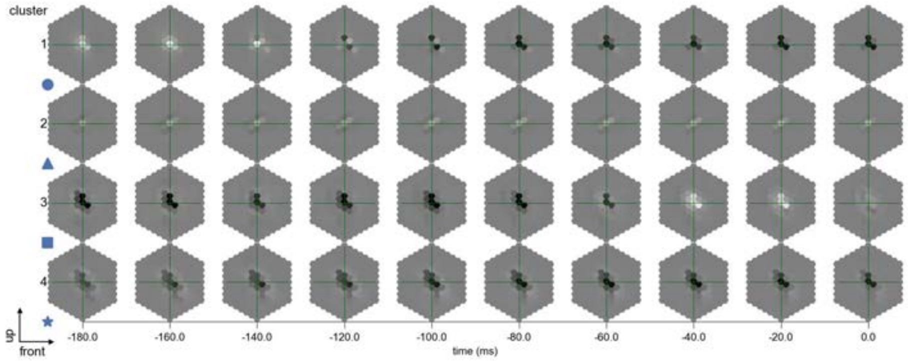

Tm5a - Figure 13: **Maximally excitatory stimuli.** Each row presents the regularized naturalistic-stimulus from the Sintel dataset that maximizes the cell type's central column response at second zero in the task-optimal model of the respective cluster (rows).

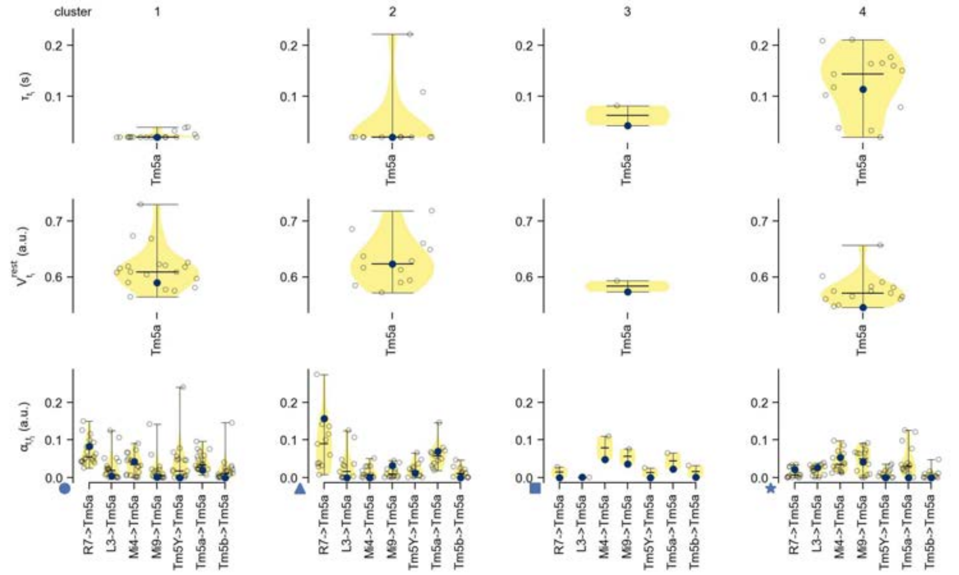

Tm5a - Figure 14: **Task-constrained parameters.** Each column shows the parameters inferred within the respective cluster. First row: learned time constants of the cell type. Second row: resting potentials of the cell type. Third row: scaling factors for the convolutional filters. The blue scatter represents the parameters from the task-optimal model within the cluster.

50 Tm5b

← Cell types

Figures

1 Anatomical receptive fields. 360
2 Anatomical projective fields. 361
3 Clustering of the responses to naturalistic stimuli. 361
4 Responses to flashes. 361
5 Cluster-average responses to single-ommatidium flashes. 362
6 Peak responses to moving edges. 362
7 Peak responses to moving edges from task-optimal models. 363
8 Responses to moving edges from task-optimal models. 363
9 Peak responses to moving bars. 364
10 Peak responses to moving bars from task-optimal models. 364
11 Responses to moving bars from task-optimal models. 365
12 Spatio-temporal receptive field. 365
13 Maximally excitatory stimuli. 365
14 Task-constrained parameters. 366

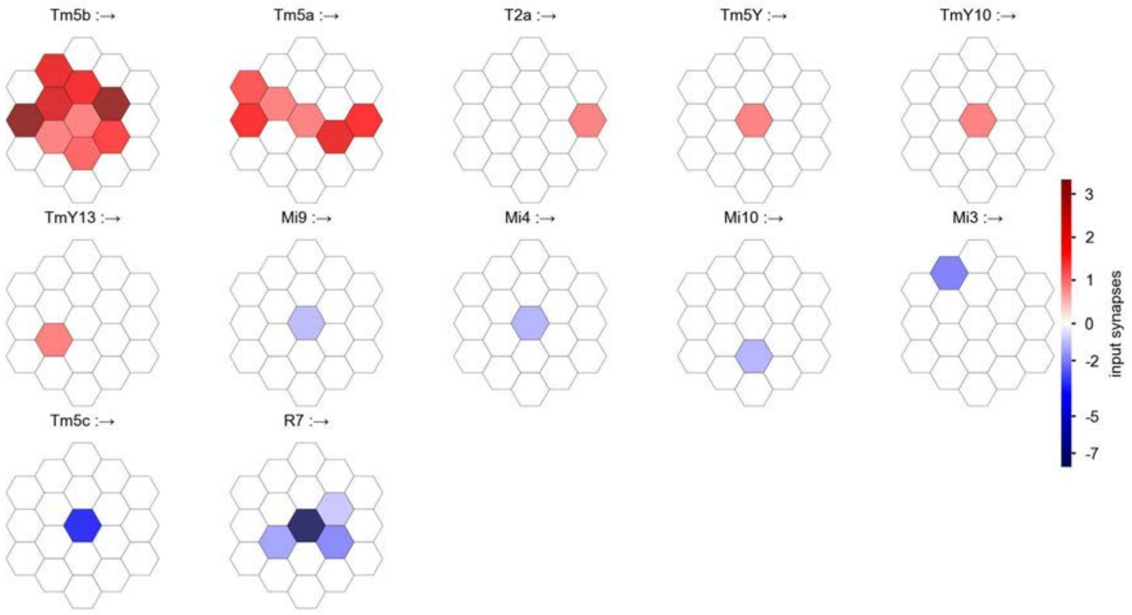

Tm5b - Figure 1: **Anatomical receptive fields.** Each colored hexagon is an input connection, with the connection strength characterized by the average number of synapses that we count from the EM reconstruction. Red indicates excitatory synapses, blue indicates inhibitory synapses from inferred signs. Filters in the order of their total number of synapses.

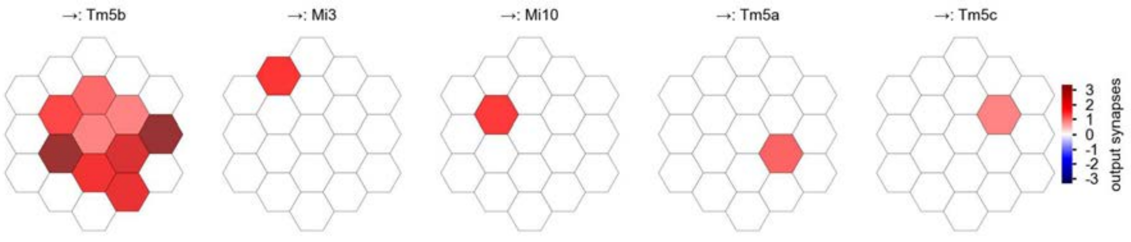

Tm5b - Figure 2: **Anatomical projective fields.** Each colored hexagon is an output connection, with the connection strength characterized by the average number of synapses that we count from the EM reconstruction. Red indicates excitatory synapses, blue indicates inhibitory synapses from inferred signs. Filters in the order of their total number of synapses.

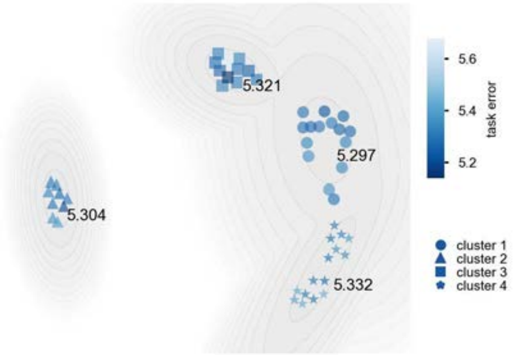

Tm5b - Figure 3: **Clustering of the responses to naturalistic stimuli.** Clustering of the 50 models based on the cell type responses to naturalistic scenes from the Sintel dataset. Scatterpoints represent individual models colored by their task error.

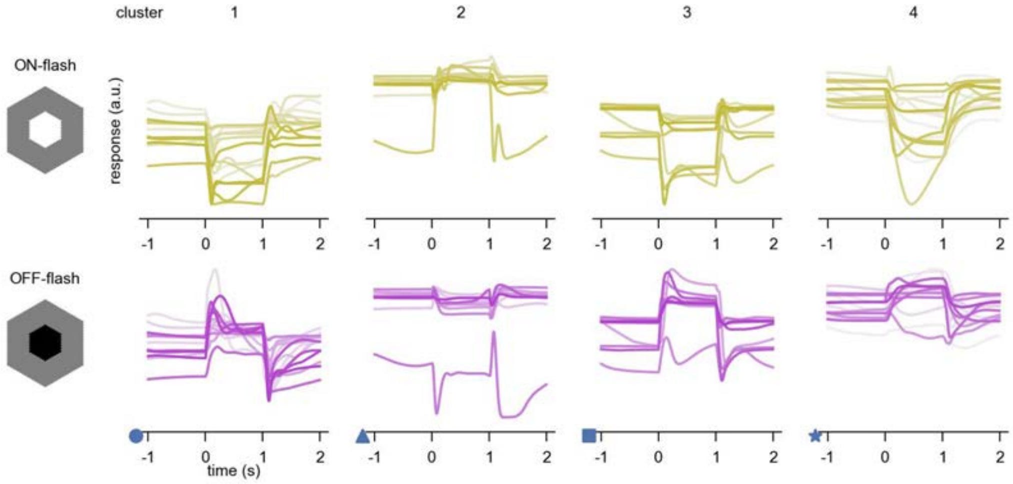

Tm5b - Figure 4: **Responses to flashes.** The top row shows responses to ON-flashes (yellow), the bottom row shows responses to OFF-flashes (magenta). The responses from the 50 different models that are separated into the different clusters (columns) overlay, with better task-performing models on top. Responses from better task-performing models are more saturated. The circular flashes (1s) cover 6 ommatidia in radius and are presented at time zero. Before and after, a grey-stimulus leads to a stationary state of the network.

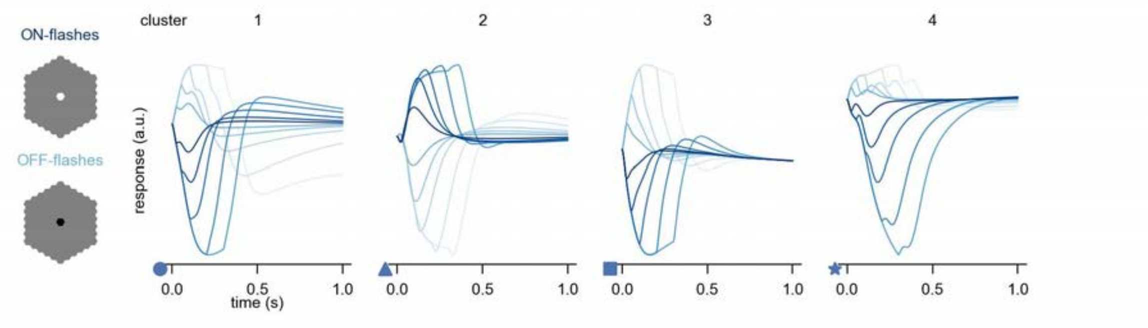

Tm5b - Figure 5: **Cluster-average responses to single-ommatidium flashes.** Responses to single-ommatidium ON-flashes (dark blue shades) and single-ommatidium OFF-flashes (light blue shades) of 20ms, 50ms, 100ms, 200ms, 300ms duration. The flashes occur at second zero.

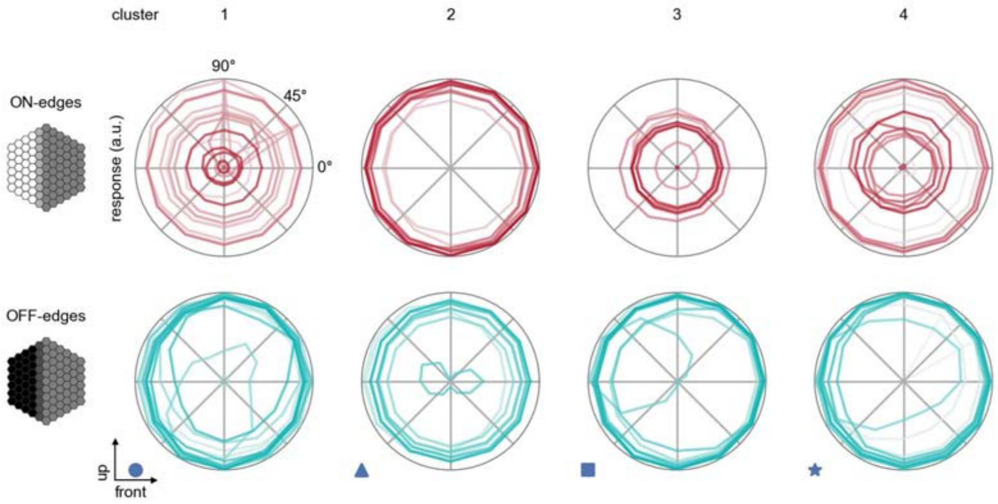

Tm5b - Figure 6: **Peak responses to moving edges.** The top row shows peak responses to moving ON-edges (red), the bottom row shows peak responses to moving OFF-edges (turqoise). The peak responses are averaged over edge-speeds. Edge-stimuli move in different directions from 0 to 360 degrees. The responses from the different models in the different clusters (columns) overlay. Responses from better task-performing models are more saturated.

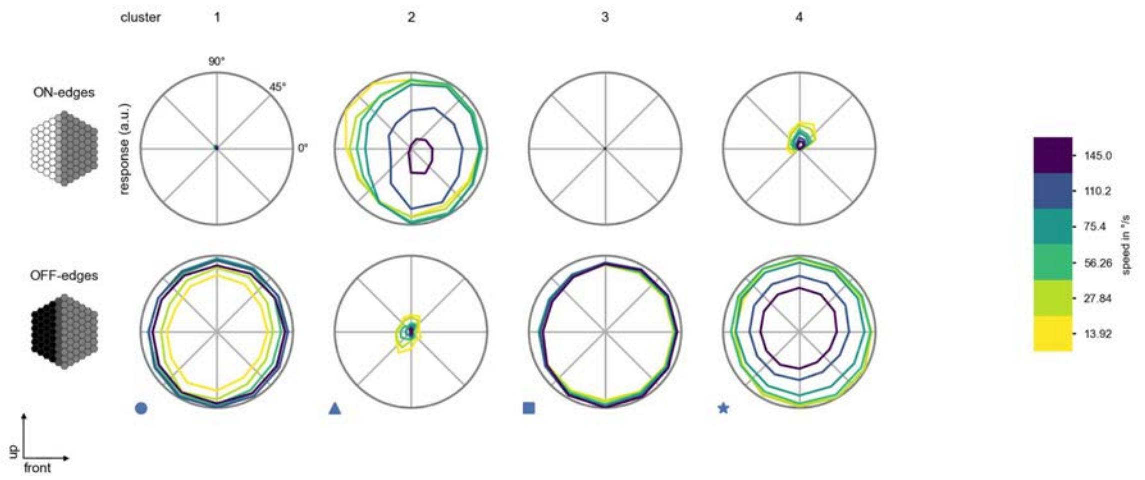

Tm5b - Figure 7: **Peak responses to moving edges from task-optimal models.** The top row shows peak responses to moving ON-edges, the bottom row shows peak responses to moving OFF-edges of varying speeds from 13.92°/s to 145°/s (yellow to dark blue). The edge-stimuli move in different directions from 0 to 360 degrees and at different speeds. Responses from the task-optimal model in the respective cluster.

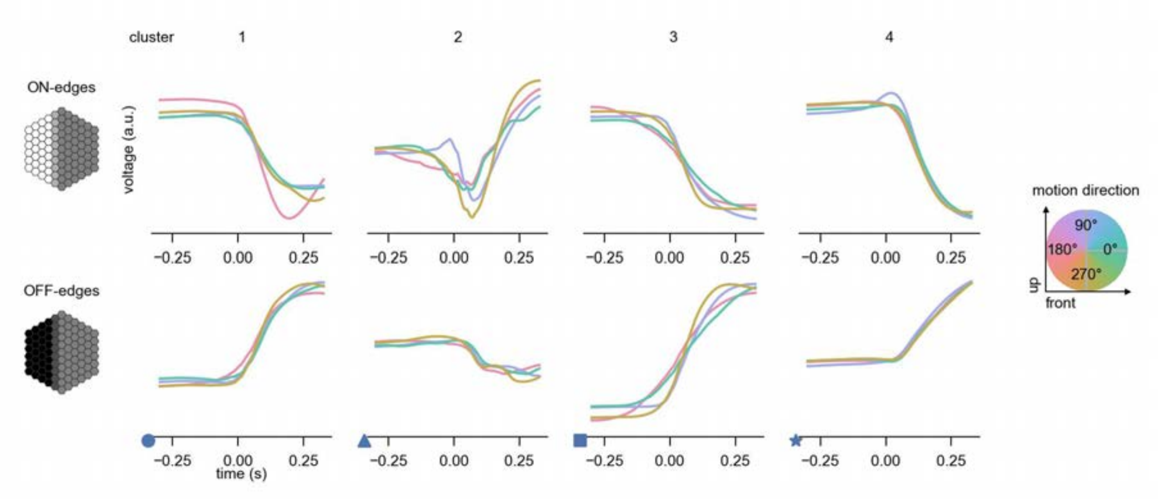

Tm5b - Figure 8: **Responses to moving edges from task-optimal models.** Responses to moving ON-edges (top row) and to moving OFF-edges (bottom row). Edges move in different directions from 0 to 360 degrees and at different speeds. Responses are from the task-optimal model in the respective cluster. Edges moving at 75.4°/s in all cardinal directions (green 0°, blue 90°, red 180°, yellow 270°) from -22.5 to 22.5° visual angle.

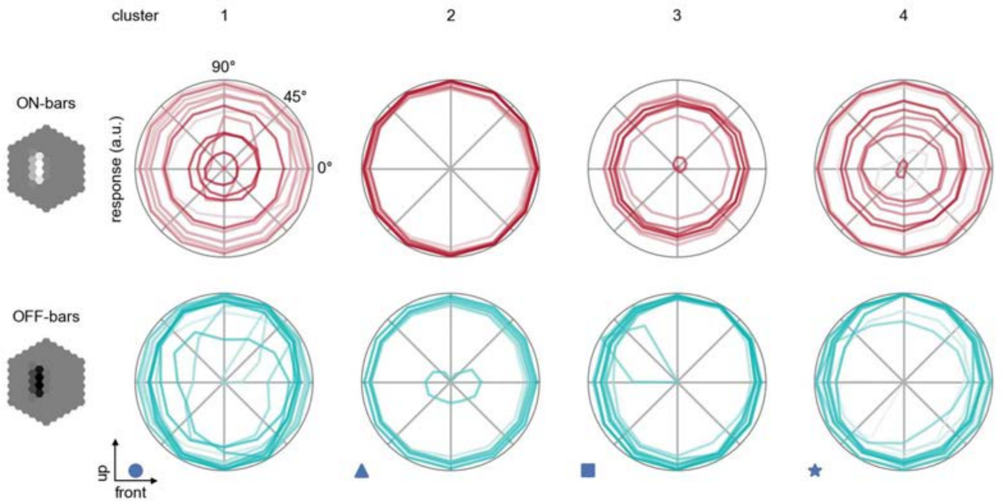

Tm5b - Figure 9: **Peak responses to moving bars.** The top row shows peak responses to moving ON-bars (red), the bottom row shows peak responses to moving OFF-bars (turqoise). The peak responses are averaged over bar-speeds. Bar-stimuli move in different directions from 0 to 360 degrees. The responses from the different models in the different clusters (columns) overlay. Responses from better task-performing models are more saturated.

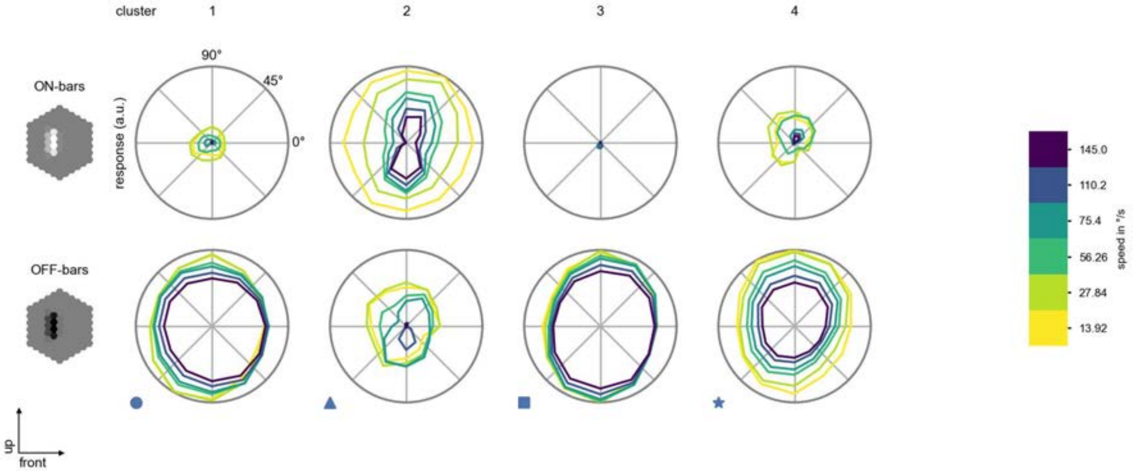

Tm5b - Figure 10: **Peak responses to moving bars from task-optimal models.** The top row shows peak responses to moving ON-bars, the bottom row shows peak responses to moving OFF-bars of varying speeds from 13.92°/s to 145°/s (yellow to dark blue). The bar-stimuli move in different directions from 0 to 360 degrees and at different speeds. Responses from the task-optimal model in the respective cluster.

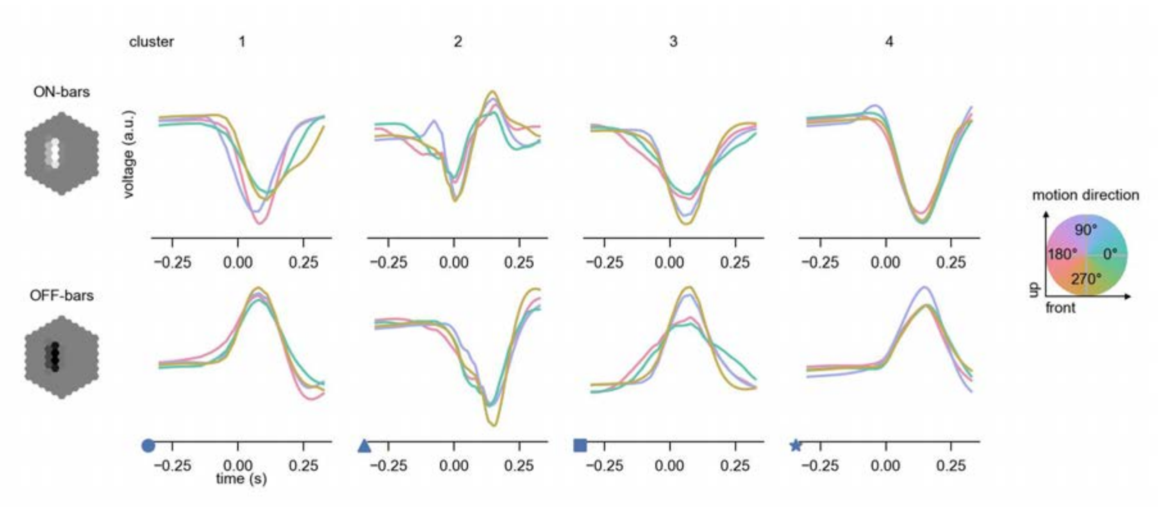

Tm5b - Figure 11: **Responses to moving bars from task-optimal models.** Responses to moving ON-bars (top row) and to moving OFF-bars (bottom row). Bars move in different directions from 0 to 360 degrees and at different speeds. Responses are from the task-optimal model in the respective cluster. Bars moving at 75.4°/s in all cardinal directions (green 0°, blue 90°, red 180°, yellow 270°) from -22.5 to 22.5° visual angle.

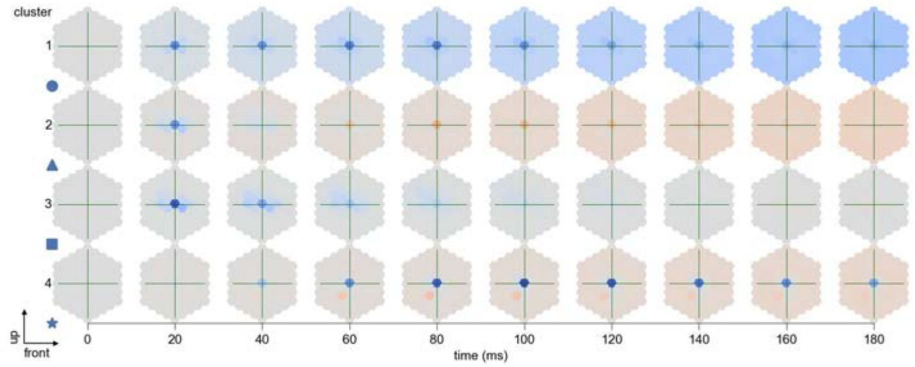

Tm5b - Figure 12: **Spatio-temporal receptive field.** Responses of the central cell to ON-impulses (5 ms) at single-ommatidium flash locations. The flash occurs at second zero. Responses from the task-optimal model of the respective cluster (rows). Red indicates depolarization, blue indicates hyperpolarization.

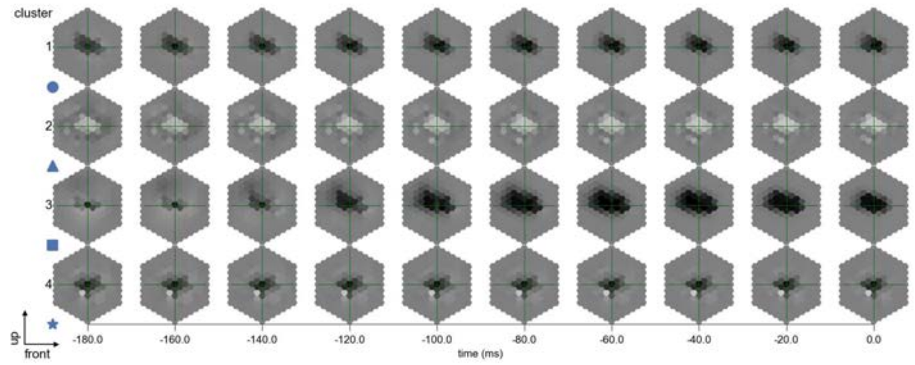

Tm5b - Figure 13: **Maximally excitatory stimuli.** Each row presents the regularized naturalistic-stimulus from the Sintel dataset that maximizes the cell type's central column response at second zero in the task-optimal model of the respective cluster (rows).

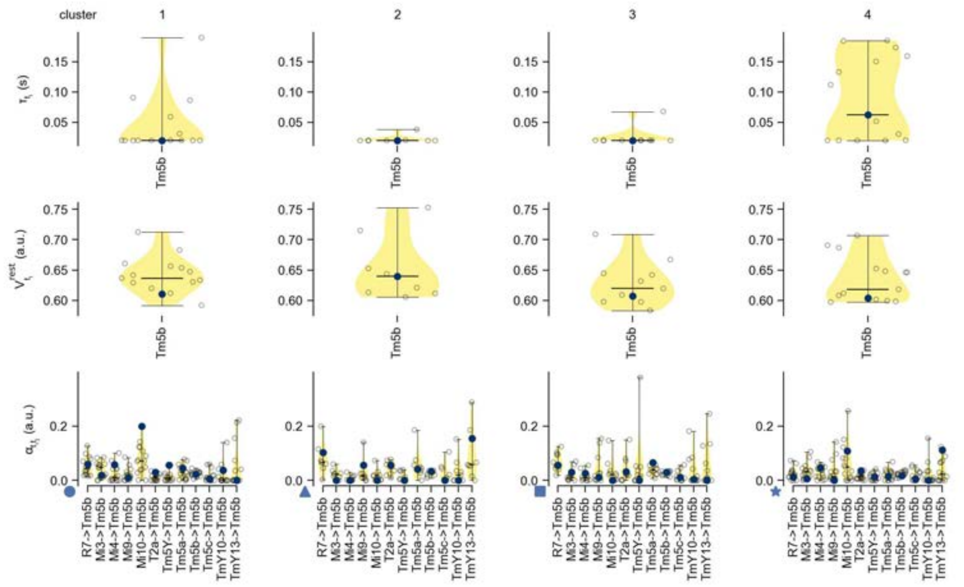

Tm5b - Figure 14: **Task-constrained parameters.** Each column shows the parameters inferred within the respective cluster. First row: learned time constants of the cell type. Second row: resting potentials of the cell type. Third row: scaling factors for the convolutional filters. The blue scatter represents the parameters from the task-optimal model within the cluster.

51 Tm5c

← Cell types

Figures

1 Anatomical receptive fields. . . . 367
2 Anatomical projective fields. . . . 367
3 Clustering of the responses to naturalistic stimuli. . . . 368
4 Responses to flashes. . . . 368
5 Cluster-average responses to single-ommatidium flashes. . . . 368
6 Peak responses to moving edges. . . . 369
7 Peak responses to moving edges from task-optimal models. . . . 369
8 Responses to moving edges from task-optimal models. . . . 370
9 Peak responses to moving bars. . . . 370
10 Peak responses to moving bars from task-optimal models. . . . 371
11 Responses to moving bars from task-optimal models. . . . 371
12 Spatio-temporal receptive field. . . . 371
13 Maximally excitatory stimuli. . . . 372
14 Task-constrained parameters. . . . 372

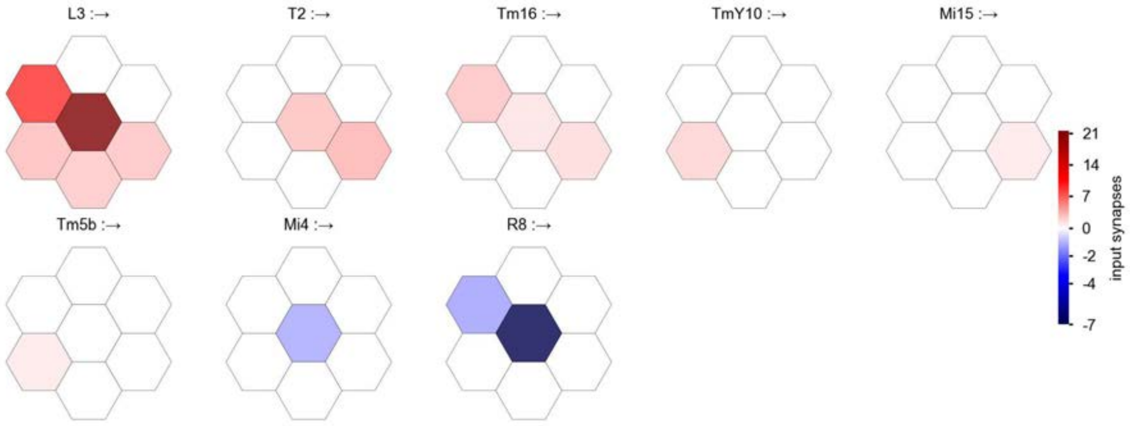

Tm5c - Figure 1: **Anatomical receptive fields.** Each colored hexagon is an input connection, with the connection strength characterized by the average number of synapses that we count from the EM reconstruction. Red indicates excitatory synapses, blue indicates inhibitory synapses from inferred signs. Filters in the order of their total number of synapses.

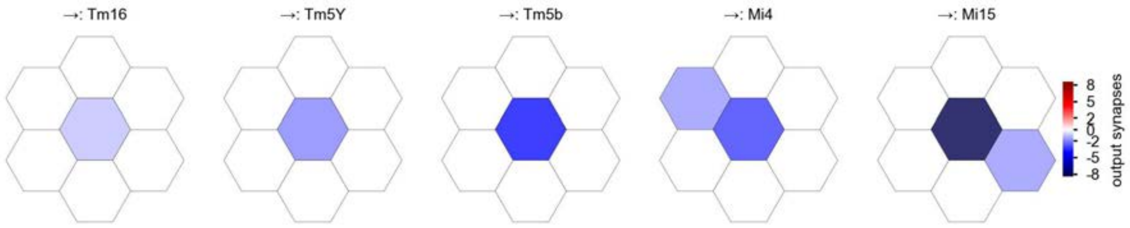

Tm5c - Figure 2: **Anatomical projective fields.** Each colored hexagon is an output connection, with the connection strength characterized by the average number of synapses that we count from the EM reconstruction. Red indicates excitatory synapses, blue indicates inhibitory synapses from inferred signs. Filters in the order of their total number of synapses.

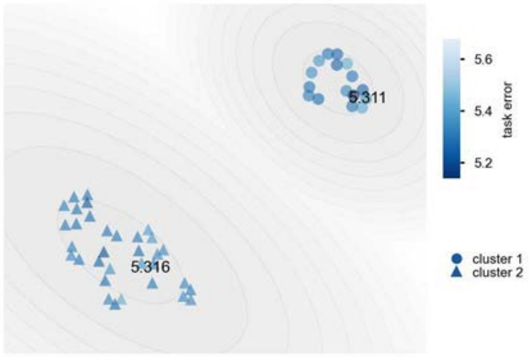

Tm5c - Figure 3: **Clustering of the responses to naturalistic stimuli.** Clustering of the 50 models based on the cell type responses to naturalistic scenes from the Sintel dataset. Scatterpoints represent individual models colored by their task error.

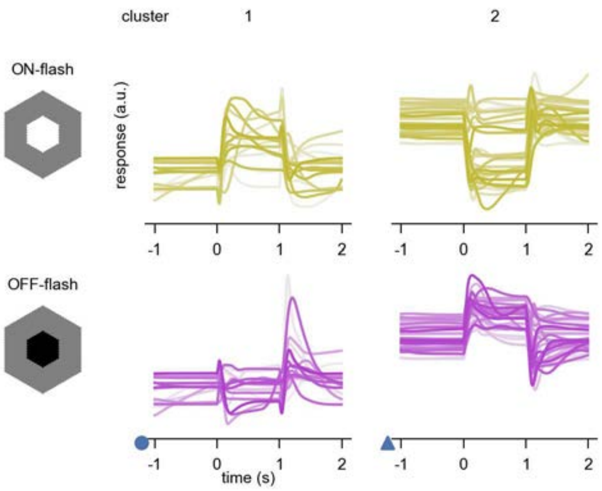

Tm5c - Figure 4: **Responses to flashes.** The top row shows responses to ON-flashes (yellow), the bottom row shows responses to OFF-flashes (magenta). The responses from the 50 different models that are separated into the different clusters (columns) overlay, with better task-performing models on top. Responses from better task-performing models are more saturated. The circular flashes (1s) cover 6 ommatidia in radius and are presented at time zero. Before and after, a grey-stimulus leads to a stationary state of the network.

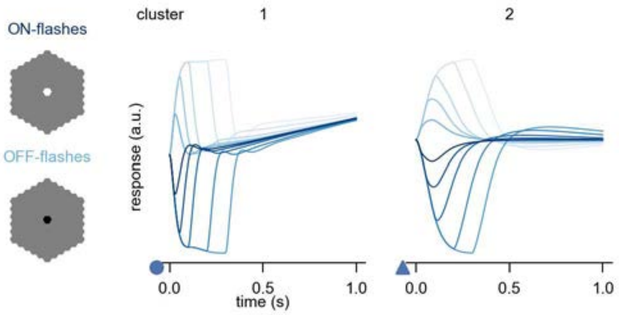

Tm5c - Figure 5: **Cluster-average responses to single-ommatidium flashes.** Responses to single-ommatidium ON-flashes (dark blue shades) and single-ommatidium OFF-flashes (light blue shades) of 20ms, 50ms, 100ms, 200ms, 300ms duration. The flashes occur at second zero.

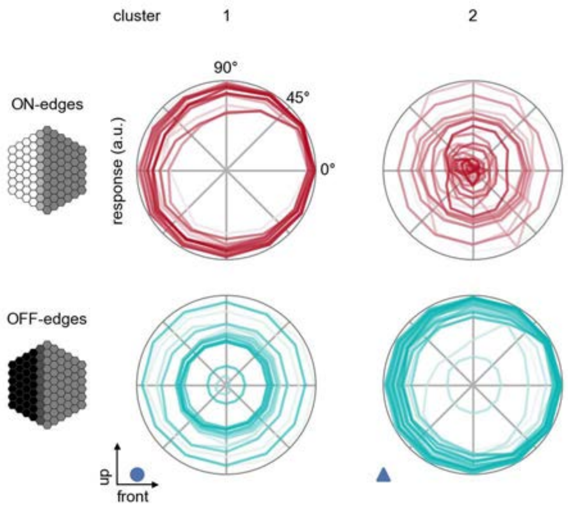

Tm5c - Figure 6: **Peak responses to moving edges.** The top row shows peak responses to moving ON-edges (red), the bottom row shows peak responses to moving OFF-edges (turqoise). The peak responses are averaged over edge-speeds. Edge-stimuli move in different directions from 0 to 360 degrees. The responses from the different models in the different clusters (columns) overlay. Responses from better task-performing models are more saturated.

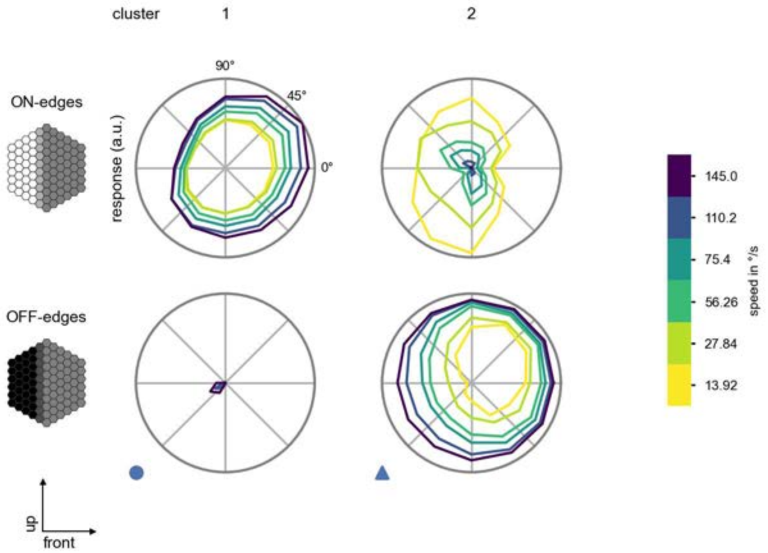

Tm5c - Figure 7: **Peak responses to moving edges from task-optimal models.** The top row shows peak responses to moving ON-edges, the bottom row shows peak responses to moving OFF-edges of varying speeds from 13.92°/s to 145°/s (yellow to dark blue). The edge-stimuli move in different directions from 0 to 360 degrees and at different speeds. Responses from the task-optimal model in the respective cluster.

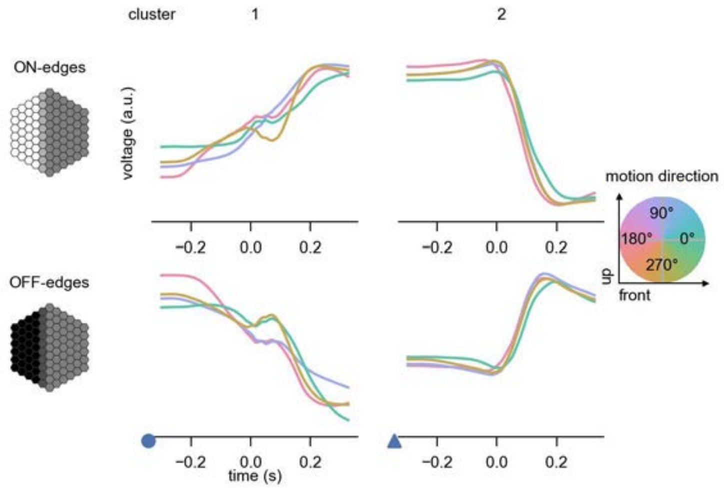

Tm5c - Figure 8: **Responses to moving edges from task-optimal models.** Responses to moving ON-edges (top row) and to moving OFF-edges (bottom row). Edges move in different directions from 0 to 360 degrees and at different speeds. Responses are from the task-optimal model in the respective cluster. Edges moving at 75.4°/s in all cardinal directions (green 0°, blue 90°, red 180°, yellow 270°) from -22.5 to 22.5° visual angle.

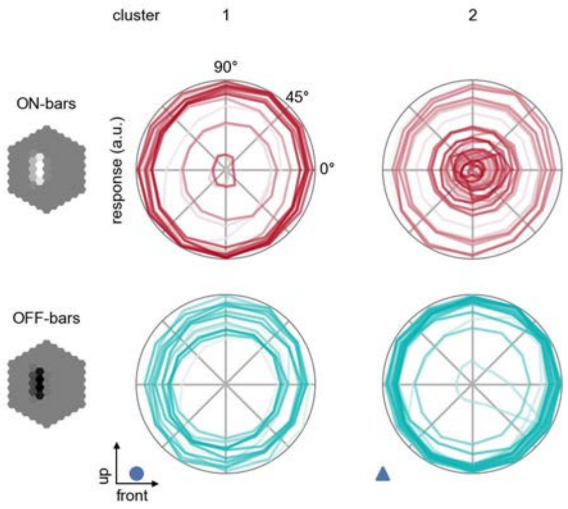

Tm5c - Figure 9: **Peak responses to moving bars.** The top row shows peak responses to moving ON-bars (red), the bottom row shows peak responses to moving OFF-bars (turqoise). The peak responses are averaged over bar-speeds. Bar-stimuli move in different directions from 0 to 360 degrees. The responses from the different models in the different clusters (columns) overlay. Responses from better task-performing models are more saturated.

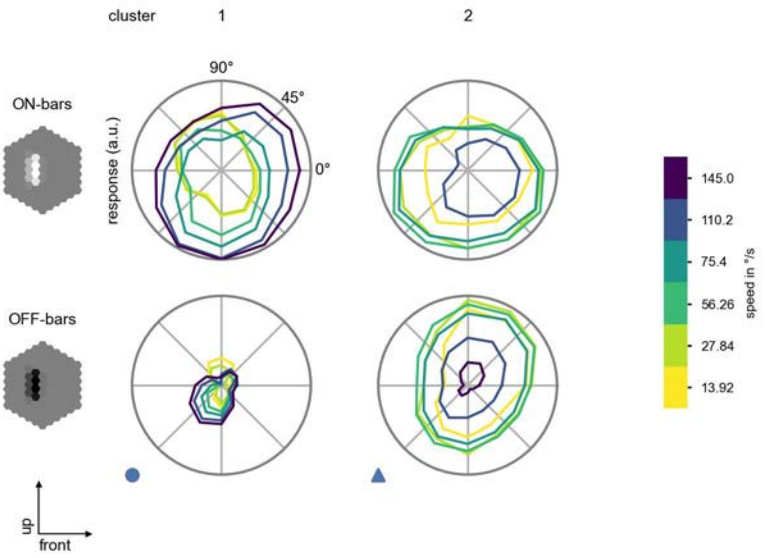

Tm5c - Figure 10: **Peak responses to moving bars from task-optimal models.** The top row shows peak responses to moving ON-bars, the bottom row shows peak responses to moving OFF-bars of varying speeds from 13.92°/s to 145°/s (yellow to dark blue). The bar-stimuli move in different directions from 0 to 360 degrees and at different speeds. Responses from the task-optimal model in the respective cluster.

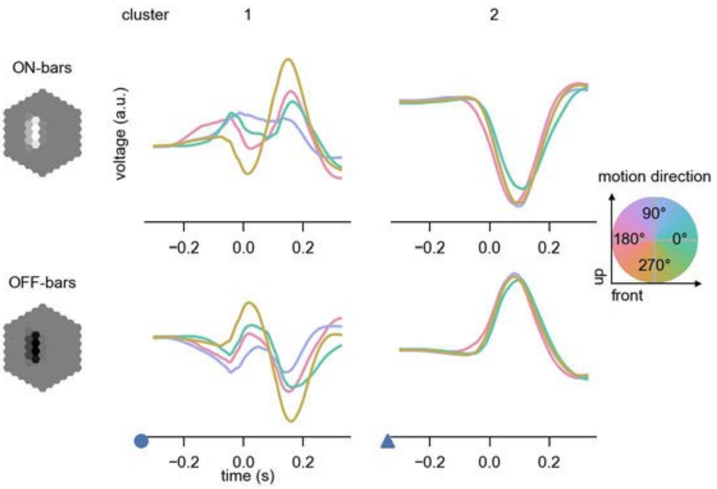

Tm5c - Figure 11: **Responses to moving bars from task-optimal models.** Responses to moving ON-bars (top row) and to moving OFF-bars (bottom row). Bars move in different directions from 0 to 360 degrees and at different speeds. Responses are from the task-optimal model in the respective cluster. Bars moving at 75.4°/s in all cardinal directions (green 0°, blue 90°, red 180°, yellow 270°) from -22.5 to 22.5° visual angle.

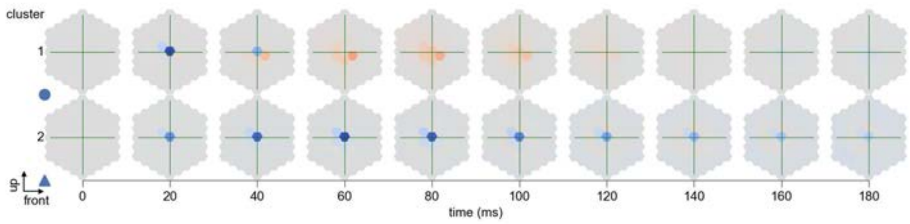

Tm5c - Figure 12: **Spatio-temporal receptive field.** Responses of the central cell to ON-impulses (5 ms) at single-ommatidium flash locations. The flash occurs at second zero. Responses from the task-optimal model of the respective cluster (rows). Red indicates depolarization, blue indicates hyperpolarization.

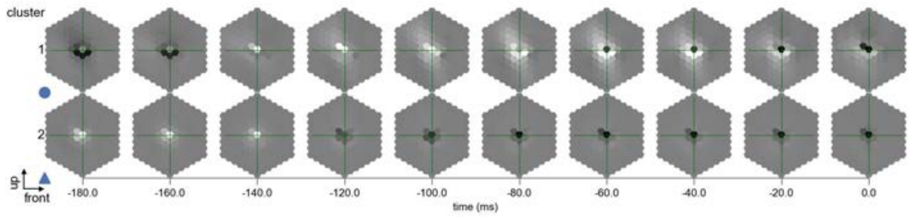

Tm5c - Figure 13: **Maximally excitatory stimuli.** Each row presents the regularized naturalistic-stimulus from the Sintel dataset that maximizes the cell type's central column response at second zero in the task-optimal model of the respective cluster (rows).

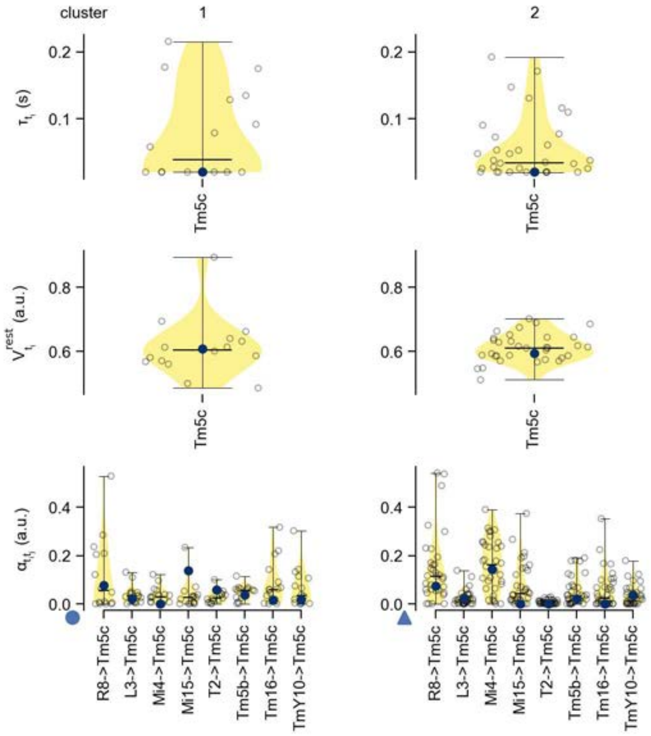

Tm5c - Figure 14: **Task-constrained parameters.** Each column shows the parameters inferred within the respective cluster. First row: learned time constants of the cell type. Second row: resting potentials of the cell type. Third row: scaling factors for the convolutional filters. The blue scatter represents the parameters from the task-optimal model within the cluster.

52 Tm9

← Cell types

Figures

1 Anatomical receptive fields. . . . 373
2 Anatomical projective fields. . . . 374
3 Clustering of the responses to naturalistic stimuli. . . . 374
4 Responses to flashes. . . . 375
5 Cluster-average responses to single-ommatidium flashes. . . . 375
6 Peak responses to moving edges. . . . 376
7 Peak responses to moving edges from task-optimal models. . . . 376
8 Responses to moving edges from task-optimal models. . . . 377
9 Peak responses to moving bars. . . . 377
10 Peak responses to moving bars from task-optimal models. . . . 378
11 Responses to moving bars from task-optimal models. . . . 378
12 Spatio-temporal receptive field. . . . 379
13 Maximally excitatory stimuli. . . . 379
14 Task-constrained parameters. . . . 380

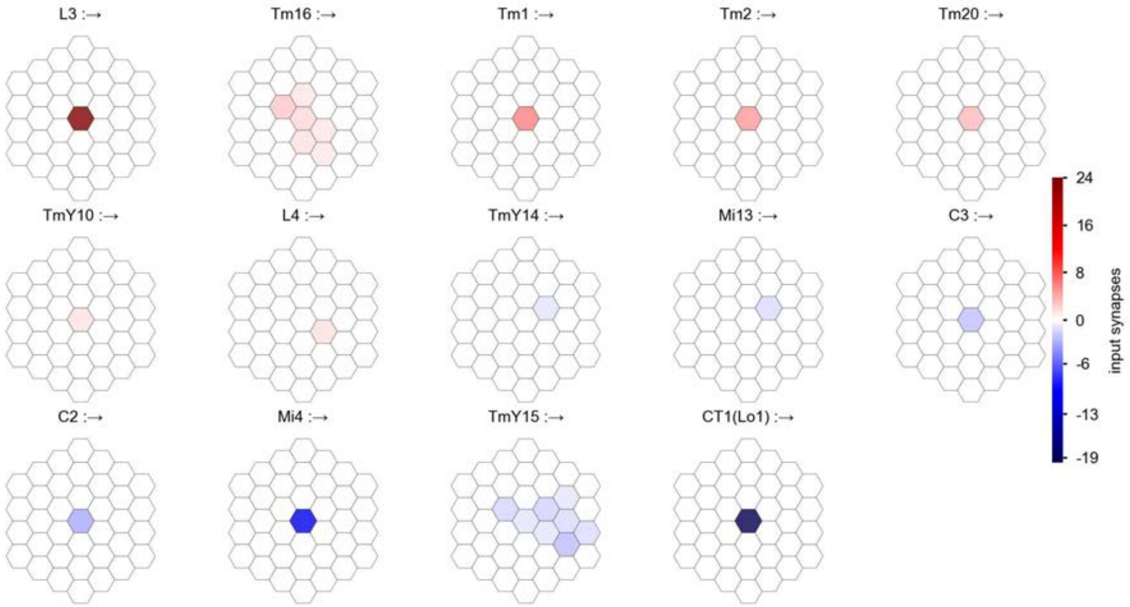

Tm9 - Figure 1: **Anatomical receptive fields.** Each colored hexagon is an input connection, with the connection strength characterized by the average number of synapses that we count from the EM reconstruction. Red indicates excitatory synapses, blue indicates inhibitory synapses from inferred signs. Filters in the order of their total number of synapses.

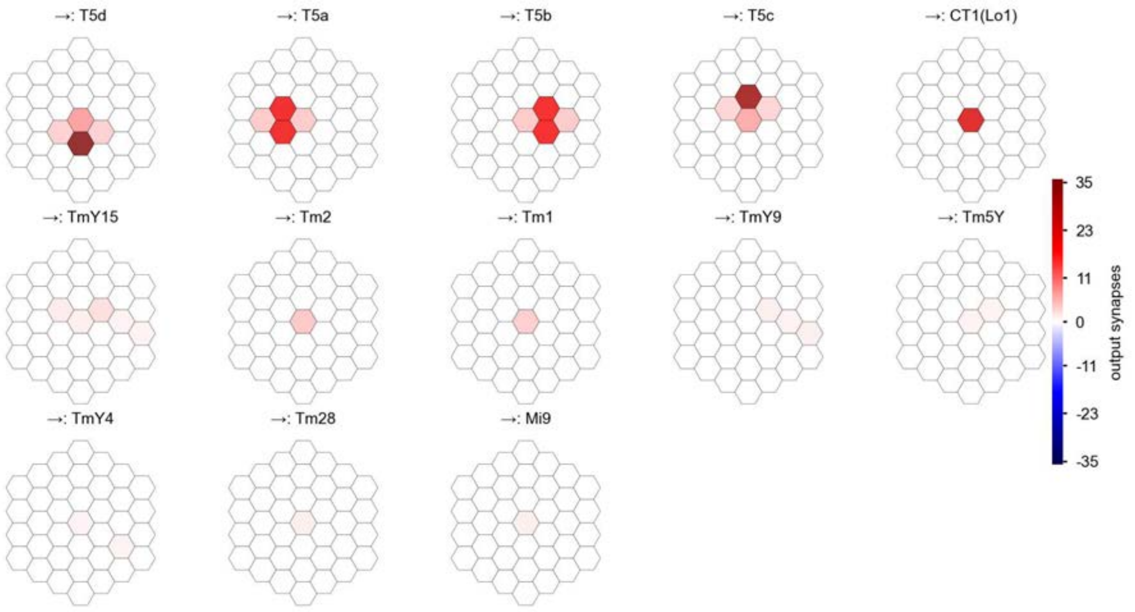

Tm9 - Figure 2: **Anatomical projective fields.** Each colored hexagon is an output connection, with the connection strength characterized by the average number of synapses that we count from the EM reconstruction. Red indicates excitatory synapses, blue indicates inhibitory synapses from inferred signs. Filters in the order of their total number of synapses.

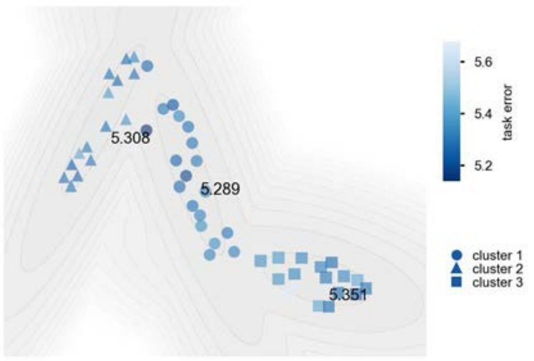

Tm9 - Figure 3: **Clustering of the responses to naturalistic stimuli.** Clustering of the 50 models based on the cell type responses to naturalistic scenes from the Sintel dataset. Scatterpoints represent individual models colored by their task error.

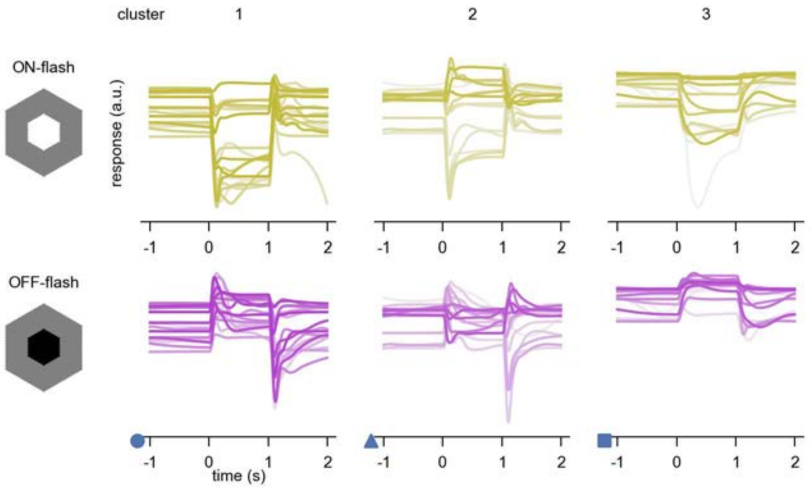

Tm9 - Figure 4: **Responses to flashes.** The top row shows responses to ON-flashes (yellow), the bottom row shows responses to OFF-flashes (magenta). The responses from the 50 different models that are separated into the different clusters (columns) overlay, with better task-performing models on top. Responses from better task-performing models are more saturated. The circular flashes (1s) cover 6 ommatidia in radius and are presented at time zero. Before and after, a grey-stimulus leads to a stationary state of the network.

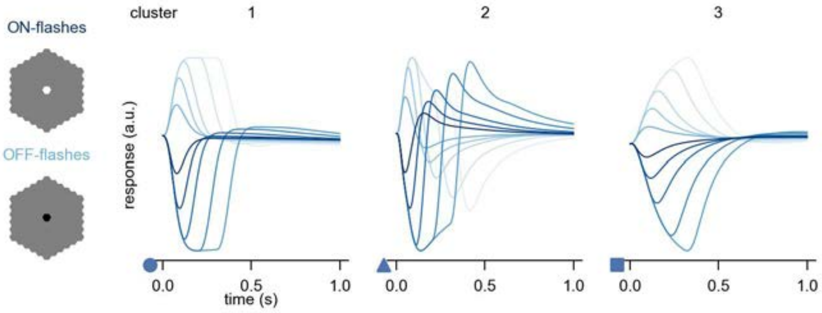

Tm9 - Figure 5: **Cluster-average responses to single-ommatidium flashes.** Responses to single-ommatidium ON-flashes (dark blue shades) and single-ommatidium OFF-flashes (light blue shades) of 20ms, 50ms, 100ms, 200ms, 300ms duration. The flashes occur at second zero.

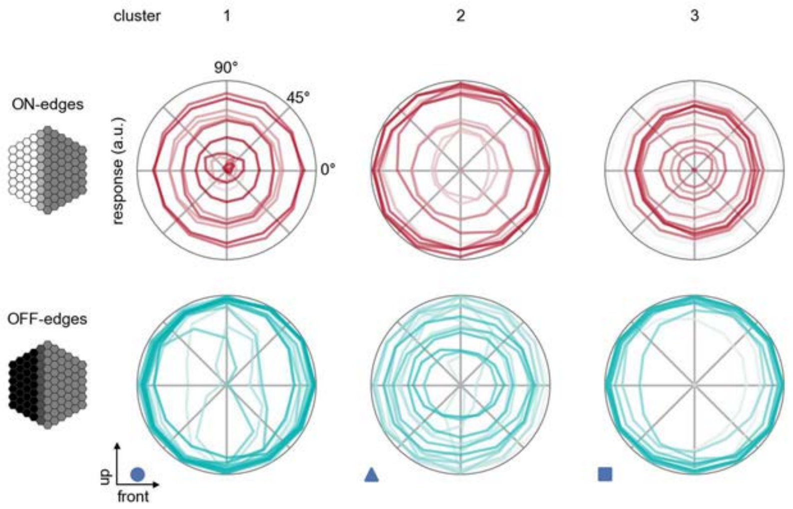

Tm9 - Figure 6: **Peak responses to moving edges.** The top row shows peak responses to moving ON-edges (red), the bottom row shows peak responses to moving OFF-edges (turqoise). The peak responses are averaged over edge-speeds. Edge-stimuli move in different directions from 0 to 360 degrees. The responses from the different models in the different clusters (columns) overlay. Responses from better task-performing models are more saturated.

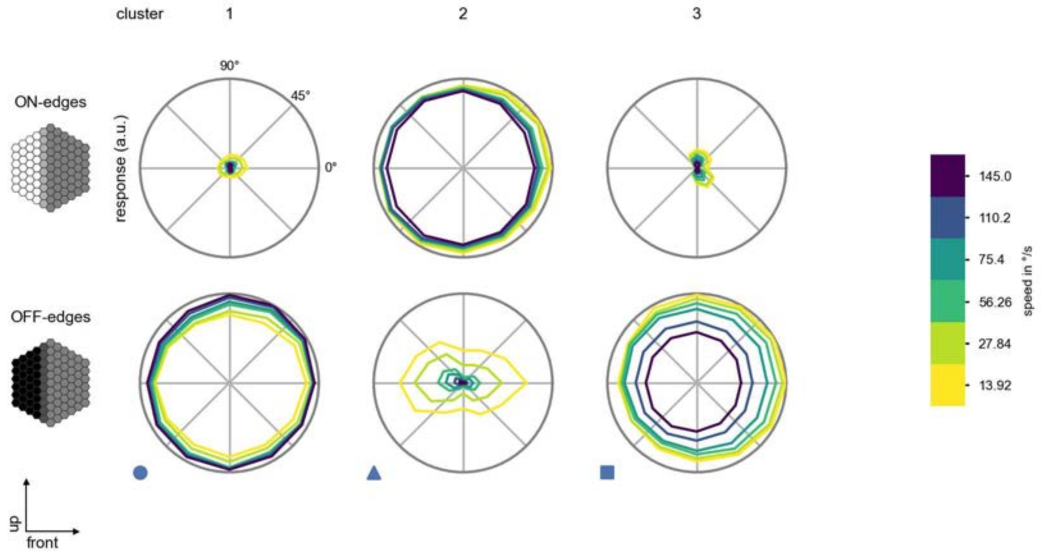

Tm9 - Figure 7: **Peak responses to moving edges from task-optimal models.** The top row shows peak responses to moving ON-edges, the bottom row shows peak responses to moving OFF-edges of varying speeds from 13.92°/s to 145°/s (yellow to dark blue). The edge-stimuli move in different directions from 0 to 360 degrees and at different speeds. Responses from the task-optimal model in the respective cluster.

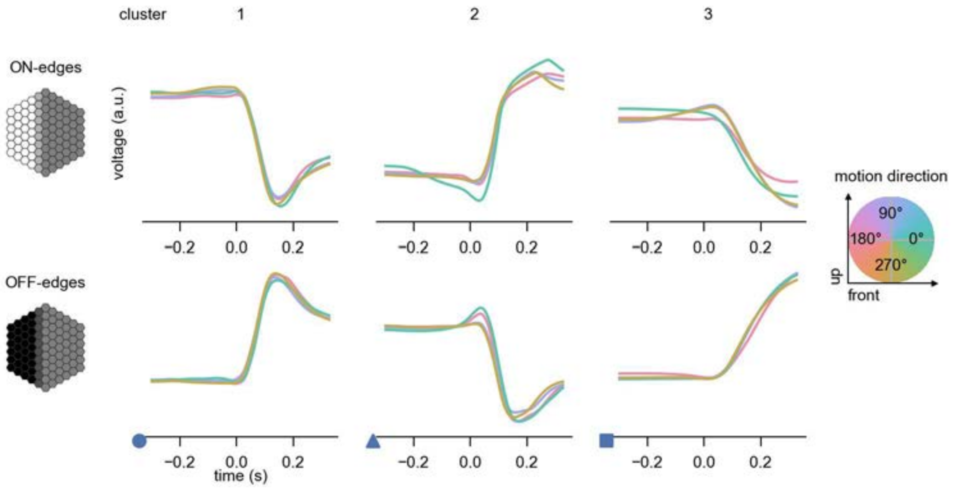

Tm9 - Figure 8: **Responses to moving edges from task-optimal models.** Responses to moving ON-edges (top row) and to moving OFF-edges (bottom row). Edges move in different directions from 0 to 360 degrees and at different speeds. Responses are from the task-optimal model in the respective cluster. Edges moving at 75.4°/s in all cardinal directions (green 0°, blue 90°, red 180°, yellow 270°) from -22.5 to 22.5° visual angle.

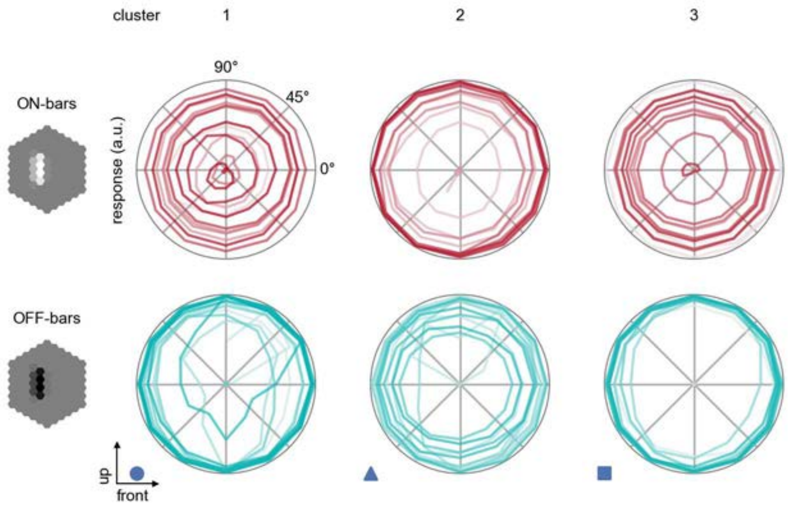

Tm9 - Figure 9: **Peak responses to moving bars.** The top row shows peak responses to moving ON-bars (red), the bottom row shows peak responses to moving OFF-bars (turqoise). The peak responses are averaged over bar-speeds. Bar-stimuli move in different directions from 0 to 360 degrees. The responses from the different models in the different clusters (columns) overlay. Responses from better task-performing models are more saturated.

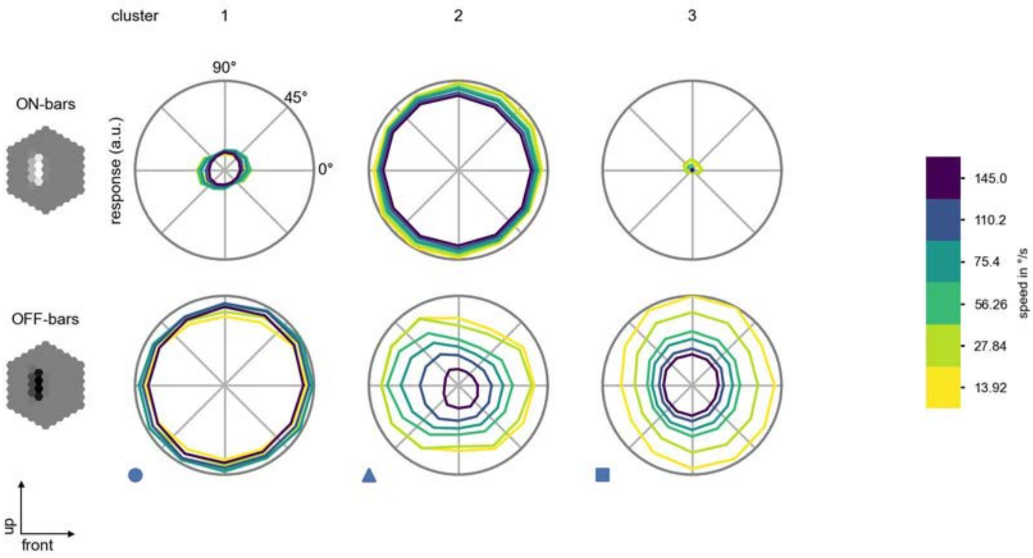

Tm9 - Figure 10: **Peak responses to moving bars from task-optimal models.** The top row shows peak responses to moving ON-bars, the bottom row shows peak responses to moving OFF-bars of varying speeds from 13.92°/s to 145°/s (yellow to dark blue). The bar-stimuli move in different directions from 0 to 360 degrees and at different speeds. Responses from the task-optimal model in the respective cluster.

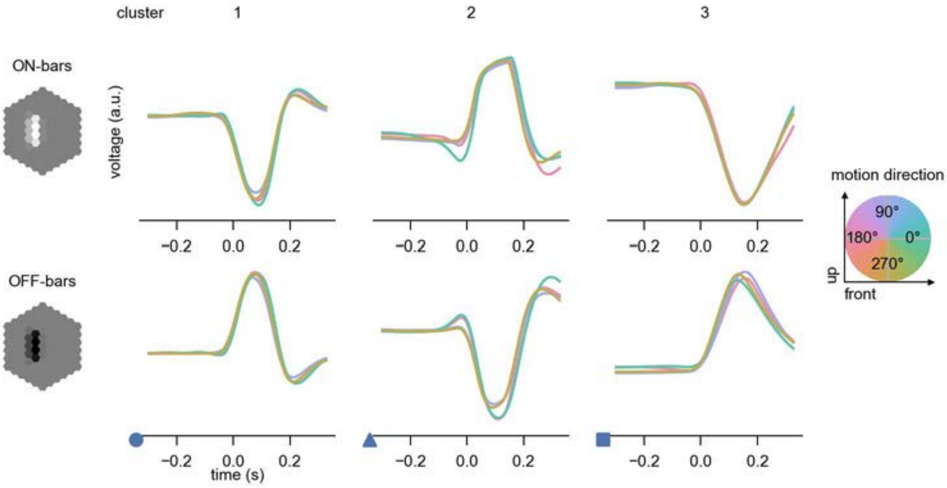

Tm9 - Figure 11: **Responses to moving bars from task-optimal models.** Responses to moving ON-bars (top row) and to moving OFF-bars (bottom row). Bars move in different directions from 0 to 360 degrees and at different speeds. Responses are from the task-optimal model in the respective cluster. Bars moving at 75.4°/s in all cardinal directions (green 0°, blue 90°, red 180°, yellow 270°) from -22.5 to 22.5° visual angle.

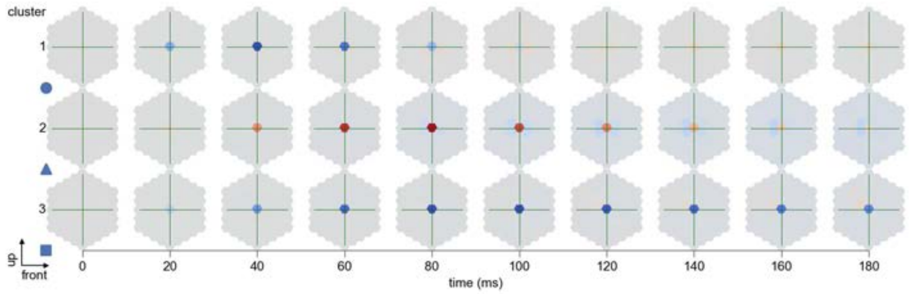

Tm9 - Figure 12: **Spatio-temporal receptive field.** Responses of the central cell to ON-impulses (5 ms) at single-ommatidium flash locations. The flash occurs at second zero. Responses from the task-optimal model of the respective cluster (rows). Red indicates depolarization, blue indicates hyperpolarization.

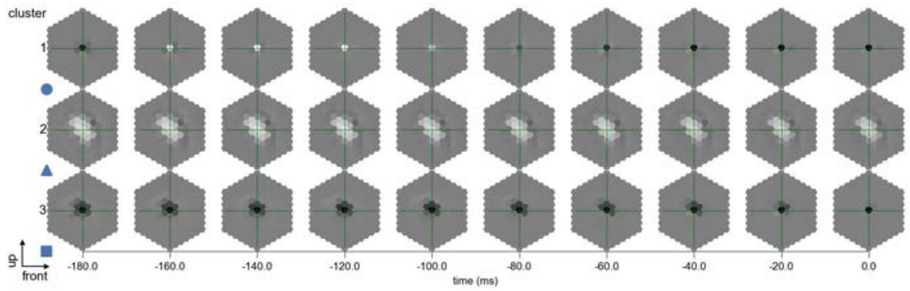

Tm9 - Figure 13: **Maximally excitatory stimuli.** Each row presents the regularized naturalistic-stimulus from the Sintel dataset that maximizes the cell type's central column response at second zero in the task-optimal model of the respective cluster (rows).

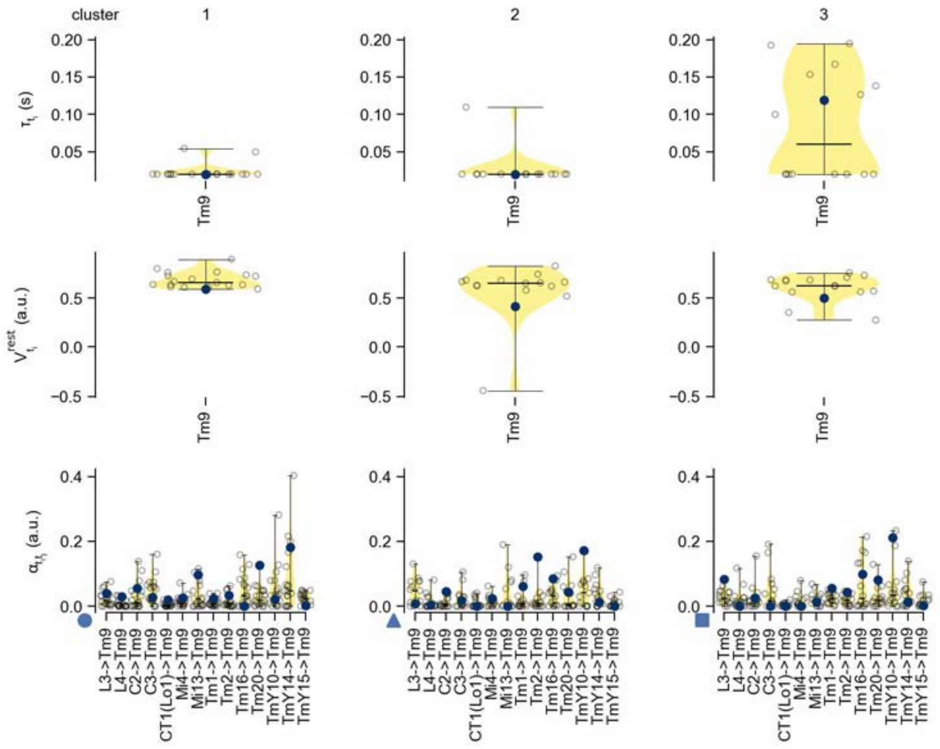

Tm9 - Figure 14: **Task-constrained parameters.** Each column shows the parameters inferred within the respective cluster. First row: learned time constants of the cell type. Second row: resting potentials of the cell type. Third row: scaling factors for the convolutional filters. The blue scatter represents the parameters from the task-optimal model within the cluster.

53 Tm16

← Cell types

Figures

1 Anatomical receptive fields. 381
2 Anatomical projective fields. 382
3 Clustering of the responses to naturalistic stimuli. 382
4 Responses to flashes. 383
5 Cluster-average responses to single-ommatidium flashes. 383
6 Peak responses to moving edges. 384
7 Peak responses to moving edges from task-optimal models. 384
8 Responses to moving edges from task-optimal models. 385
9 Peak responses to moving bars. 385
10 Peak responses to moving bars from task-optimal models. 386
11 Responses to moving bars from task-optimal models. 386
12 Spatio-temporal receptive field. 387
13 Maximally excitatory stimuli. 387
14 Task-constrained parameters. 388

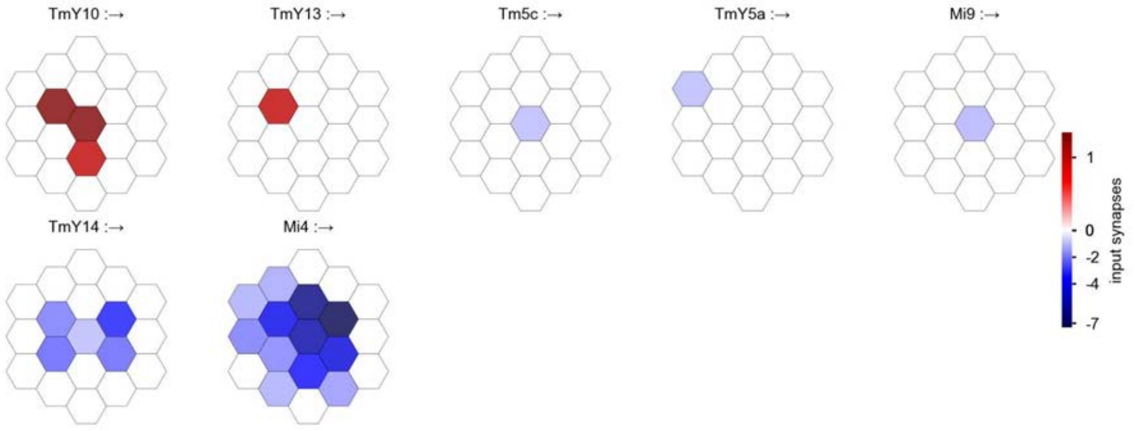

Tm16 - Figure 1: **Anatomical receptive fields.** Each colored hexagon is an input connection, with the connection strength characterized by the average number of synapses that we count from the EM reconstruction. Red indicates excitatory synapses, blue indicates inhibitory synapses from inferred signs. Filters in the order of their total number of synapses.

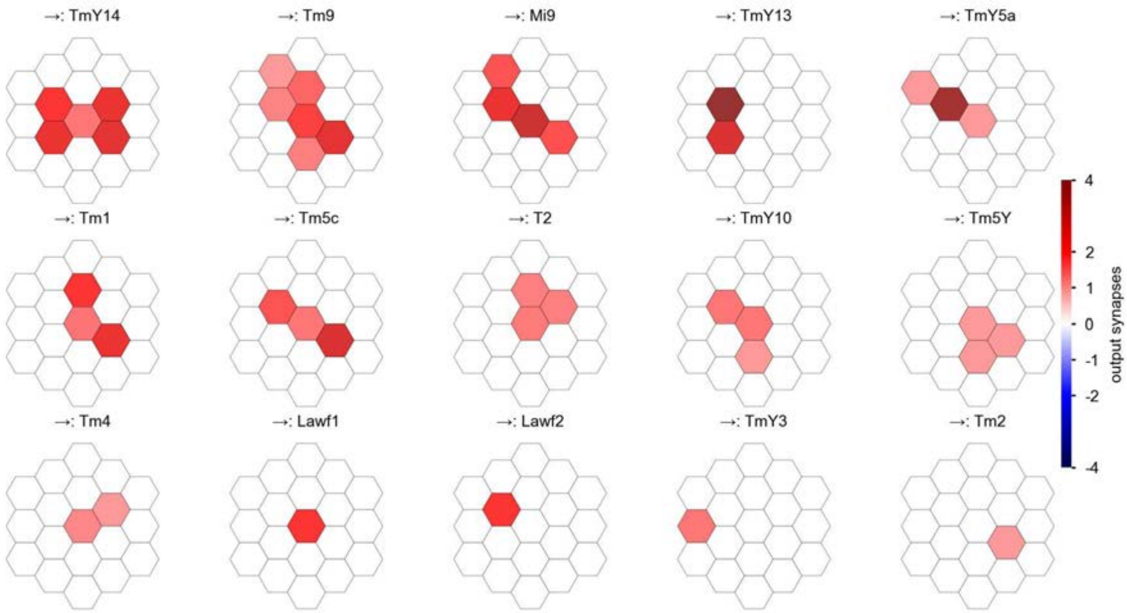

Tm16 - Figure 2: **Anatomical projective fields.** Each colored hexagon is an output connection, with the connection strength characterized by the average number of synapses that we count from the EM reconstruction. Red indicates excitatory synapses, blue indicates inhibitory synapses from inferred signs. Filters in the order of their total number of synapses.

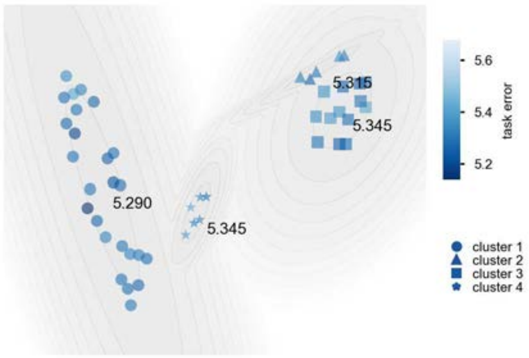

Tm16 - Figure 3: **Clustering of the responses to naturalistic stimuli.** Clustering of the 50 models based on the cell type responses to naturalistic scenes from the Sintel dataset. Scatterpoints represent individual models colored by their task error.

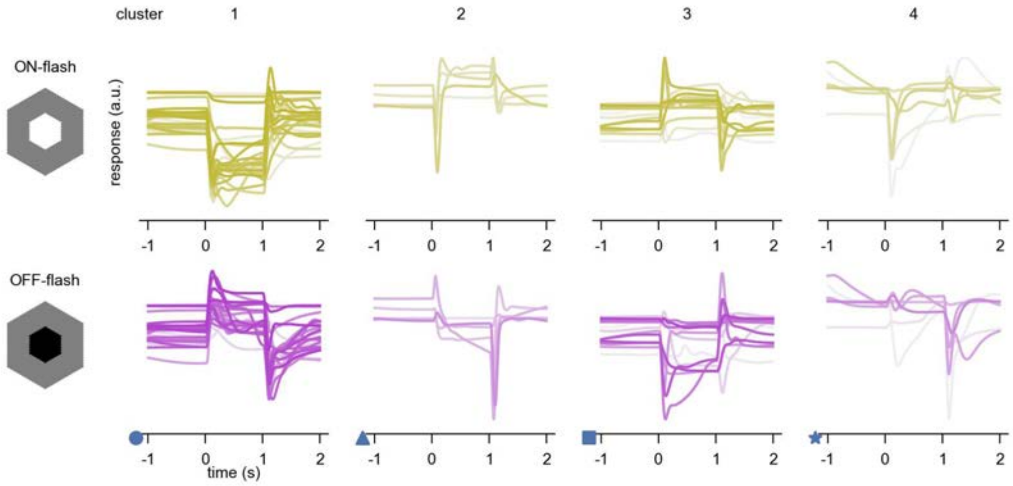

Tm16 - Figure 4: **Responses to flashes.** The top row shows responses to ON-flashes (yellow), the bottom row shows responses to OFF-flashes (magenta). The responses from the 50 different models that are separated into the different clusters (columns) overlay, with better task-performing models on top. Responses from better task-performing models are more saturated. The circular flashes (1s) cover 6 ommatidia in radius and are presented at time zero. Before and after, a grey-stimulus leads to a stationary state of the network.

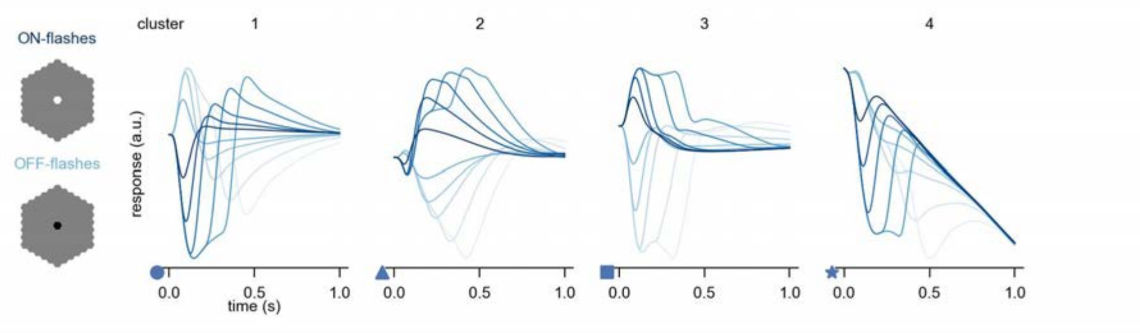

Tm16 - Figure 5: **Cluster-average responses to single-ommatidium flashes.** Responses to single-ommatidium ON-flashes (dark blue shades) and single-ommatidium OFF-flashes (light blue shades) of 20ms, 50ms, 100ms, 200ms, 300ms duration. The flashes occur at second zero.

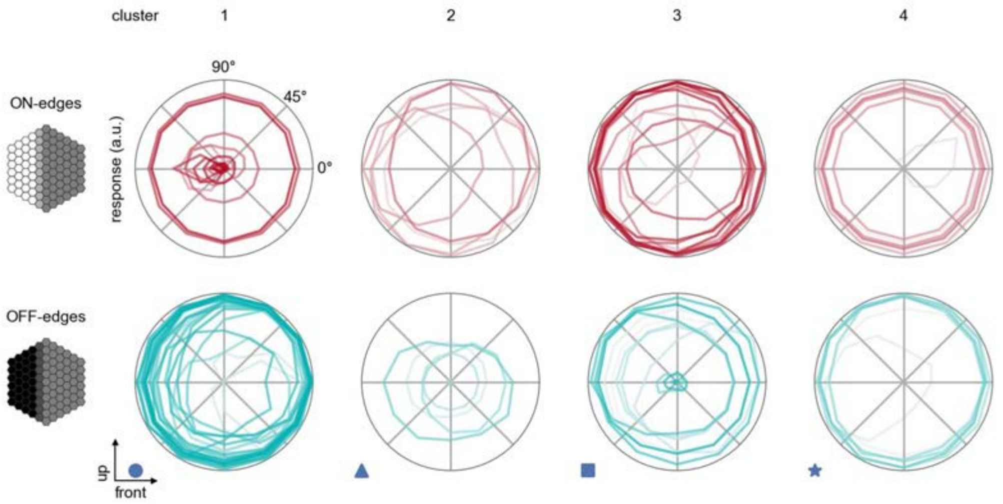

Tm16 - Figure 6: **Peak responses to moving edges.** The top row shows peak responses to moving ON-edges (red), the bottom row shows peak responses to moving OFF-edges (turqoise). The peak responses are averaged over edge-speeds. Edge-stimuli move in different directions from 0 to 360 degrees. The responses from the different models in the different clusters (columns) overlay. Responses from better task-performing models are more saturated.

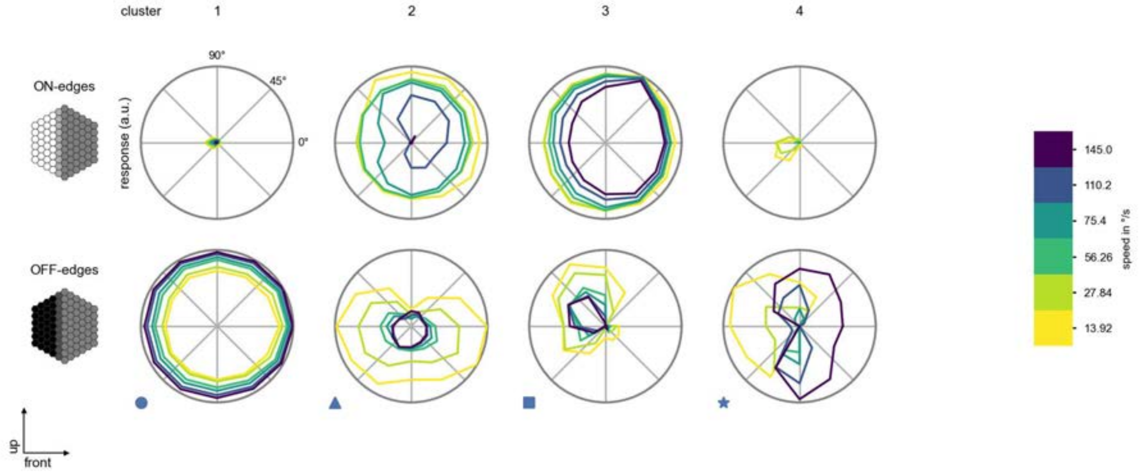

Tm16 - Figure 7: **Peak responses to moving edges from task-optimal models.** The top row shows peak responses to moving ON-edges, the bottom row shows peak responses to moving OFF-edges of varying speeds from 13.92°/s to 145°/s (yellow to dark blue). The edge-stimuli move in different directions from 0 to 360 degrees and at different speeds. Responses from the task-optimal model in the respective cluster.

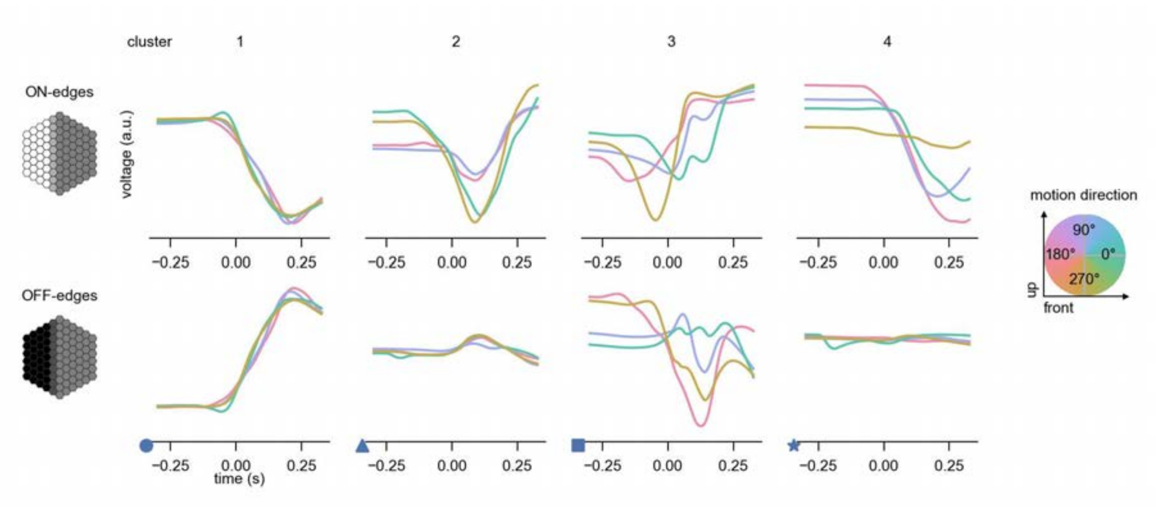

Tm16 - Figure 8: **Responses to moving edges from task-optimal models.** Responses to moving ON-edges (top row) and to moving OFF-edges (bottom row). Edges move in different directions from 0 to 360 degrees and at different speeds. Responses are from the task-optimal model in the respective cluster. Edges moving at 75.4°/s in all cardinal directions (green 0°, blue 90°, red 180°, yellow 270°) from -22.5 to 22.5° visual angle.

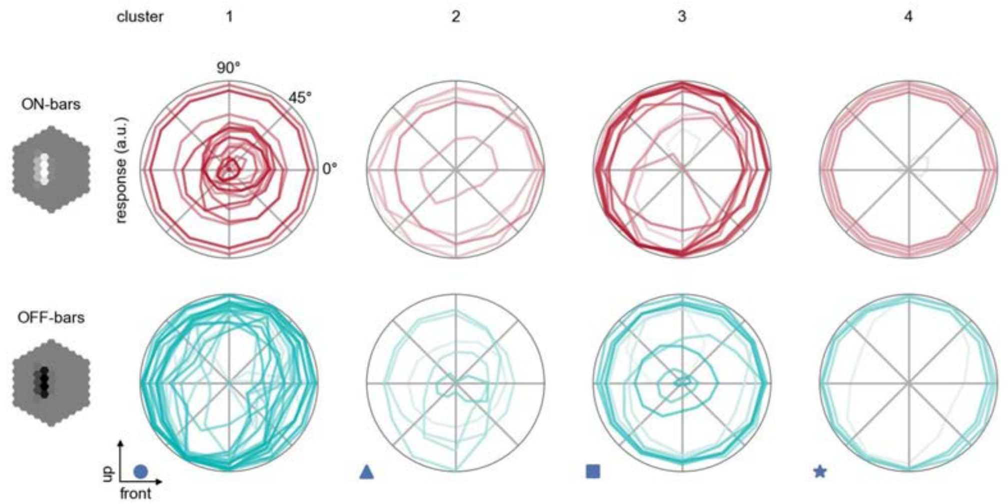

Tm16 - Figure 9: **Peak responses to moving bars.** The top row shows peak responses to moving ON-bars (red), the bottom row shows peak responses to moving OFF-bars (turqoise). The peak responses are averaged over bar-speeds. Bar-stimuli move in different directions from 0 to 360 degrees. The responses from the different models in the different clusters (columns) overlay. Responses from better task-performing models are more saturated.

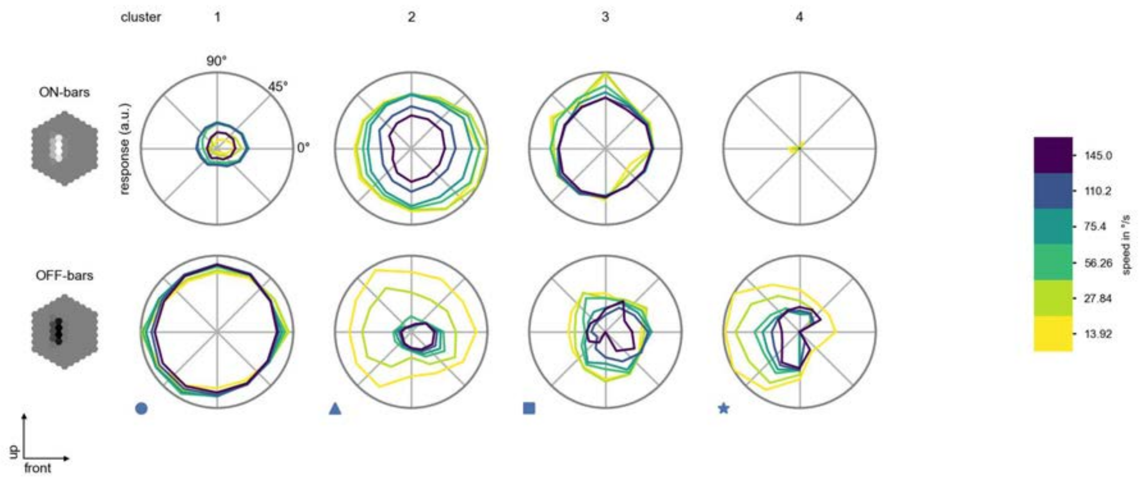

Tm16 - Figure 10: **Peak responses to moving bars from task-optimal models.** The top row shows peak responses to moving ON-bars, the bottom row shows peak responses to moving OFF-bars of varying speeds from 13.92°/s to 145°/s (yellow to dark blue). The bar-stimuli move in different directions from 0 to 360 degrees and at different speeds. Responses from the task-optimal model in the respective cluster.

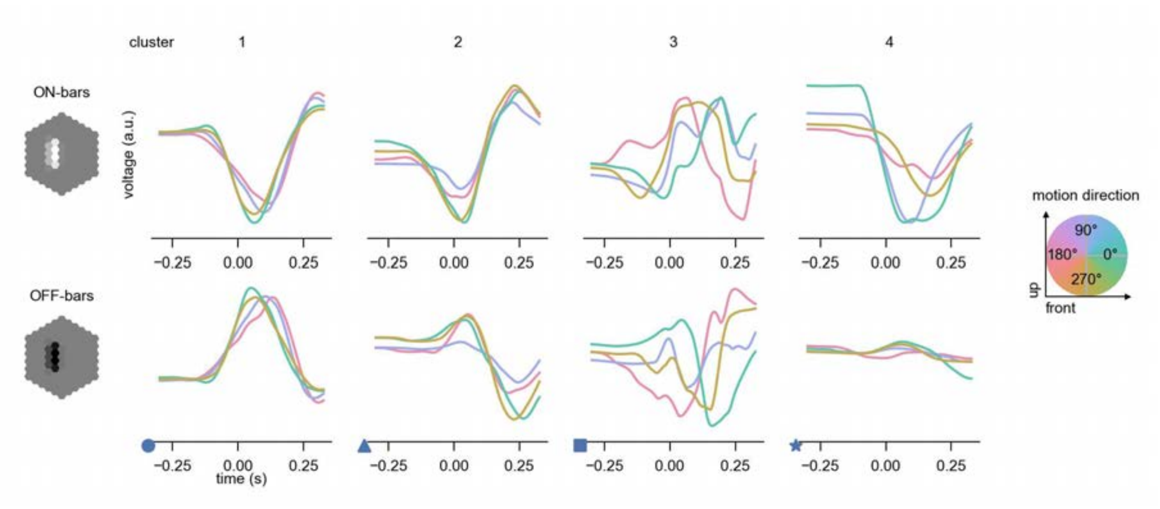

Tm16 - Figure 11: **Responses to moving bars from task-optimal models.** Responses to moving ON-bars (top row) and to moving OFF-bars (bottom row). Bars move in different directions from 0 to 360 degrees and at different speeds. Responses are from the task-optimal model in the respective cluster. Bars moving at 75.4°/s in all cardinal directions (green 0°, blue 90°, red 180°, yellow 270°) from -22.5 to 22.5° visual angle.

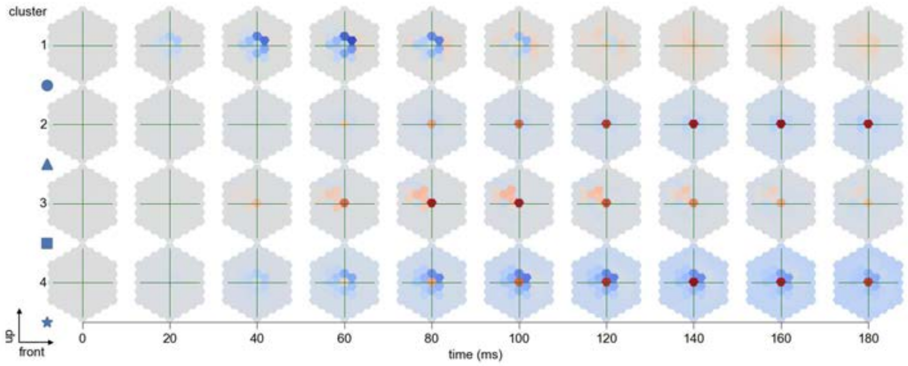

Tm16 - Figure 12: **Spatio-temporal receptive field.** Responses of the central cell to ON-impulses (5 ms) at single-ommatidium flash locations. The flash occurs at second zero. Responses from the task-optimal model of the respective cluster (rows). Red indicates depolarization, blue indicates hyperpolarization.

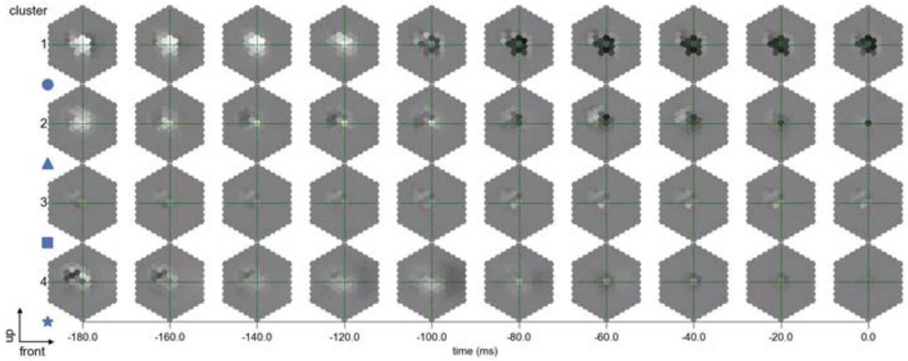

Tm16 - Figure 13: **Maximally excitatory stimuli.** Each row presents the regularized naturalistic-stimulus from the Sintel dataset that maximizes the cell type's central column response at second zero in the task-optimal model of the respective cluster (rows).

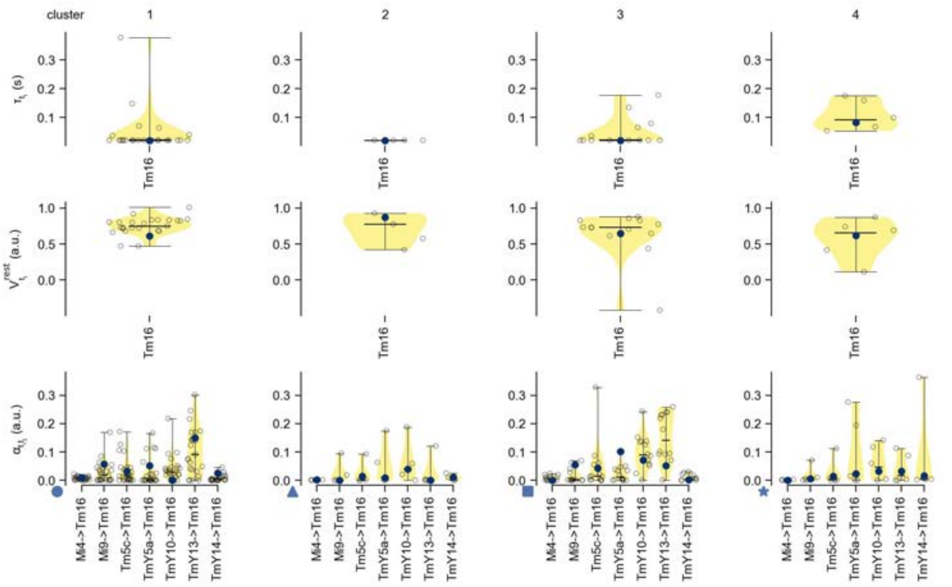

Tm16 - Figure 14: **Task-constrained parameters.** Each column shows the parameters inferred within the respective cluster. First row: learned time constants of the cell type. Second row: resting potentials of the cell type. Third row: scaling factors for the convolutional filters. The blue scatter represents the parameters from the task-optimal model within the cluster.

54 Tm20

← Cell types

Figures

1 Anatomical receptive fields. . . . 389
2 Anatomical projective fields. . . . 390
3 Clustering of the responses to naturalistic stimuli. . . . 390
4 Responses to flashes. . . . 390
5 Cluster-average responses to single-ommatidium flashes. . . . 391
6 Peak responses to moving edges. . . . 391
7 Peak responses to moving edges from task-optimal models. . . . 392
8 Responses to moving edges from task-optimal models. . . . 392
9 Peak responses to moving bars. . . . 393
10 Peak responses to moving bars from task-optimal models. . . . 393
11 Responses to moving bars from task-optimal models. . . . 394
12 Spatio-temporal receptive field. . . . 394
13 Maximally excitatory stimuli. . . . 394
14 Task-constrained parameters. . . . 395

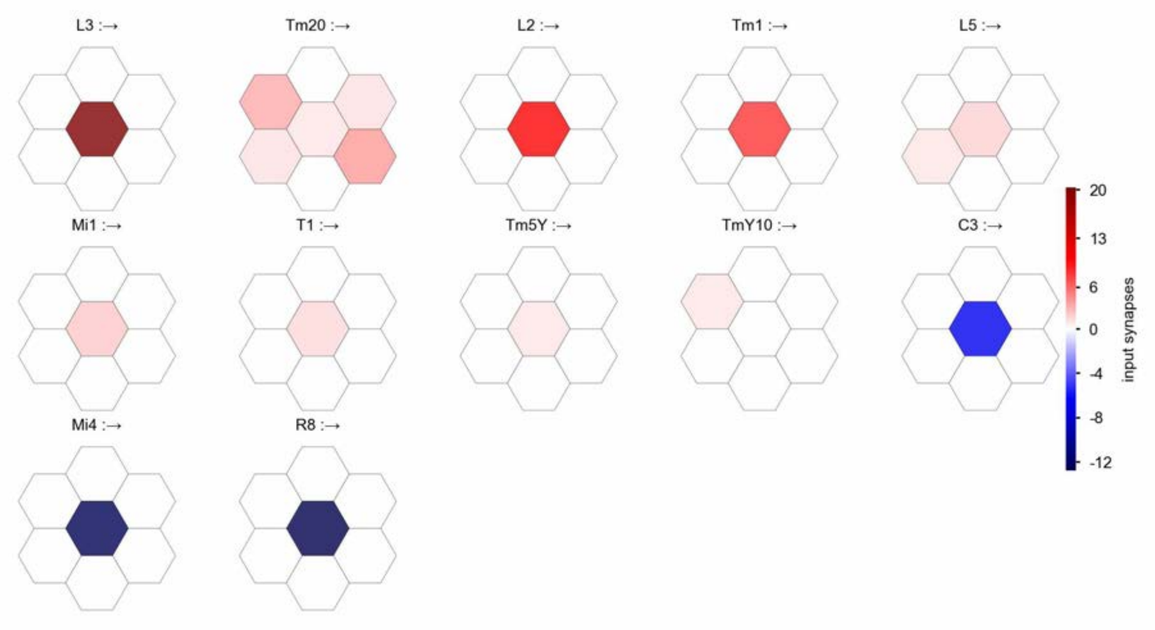

Tm20 - Figure 1: **Anatomical receptive fields.** Each colored hexagon is an input connection, with the connection strength characterized by the average number of synapses that we count from the EM reconstruction. Red indicates excitatory synapses, blue indicates inhibitory synapses from inferred signs. Filters in the order of their total number of synapses.

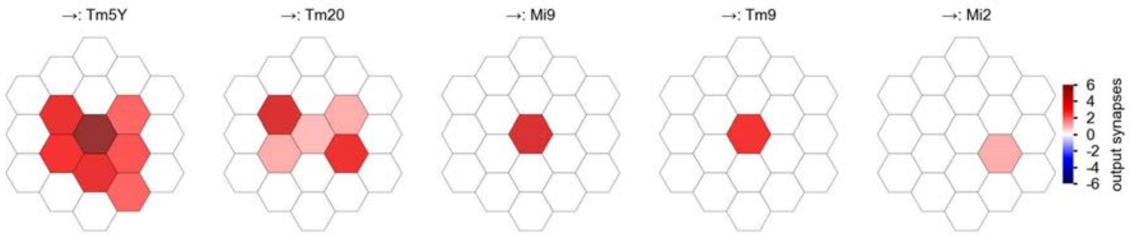

Tm20 - Figure 2: **Anatomical projective fields.** Each colored hexagon is an output connection, with the connection strength characterized by the average number of synapses that we count from the EM reconstruction. Red indicates excitatory synapses, blue indicates inhibitory synapses from inferred signs. Filters in the order of their total number of synapses.

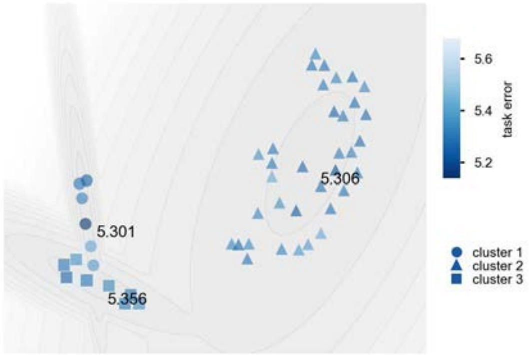

Tm20 - Figure 3: **Clustering of the responses to naturalistic stimuli.** Clustering of the 50 models based on the cell type responses to naturalistic scenes from the Sintel dataset. Scatterpoints represent individual models colored by their task error.

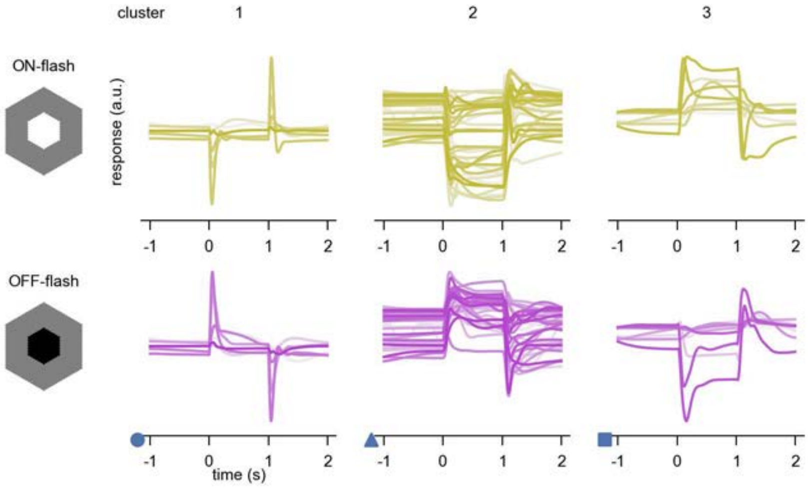

Tm20 - Figure 4: **Responses to flashes.** The top row shows responses to ON-flashes (yellow), the bottom row shows responses to OFF-flashes (magenta). The responses from the 50 different models that are separated into the different clusters (columns) overlay, with better task-performing models on top. Responses from better task-performing models are more saturated. The circular flashes (1s) cover 6 ommatidia in radius and are presented at time zero. Before and after, a grey-stimulus leads to a stationary state of the network.

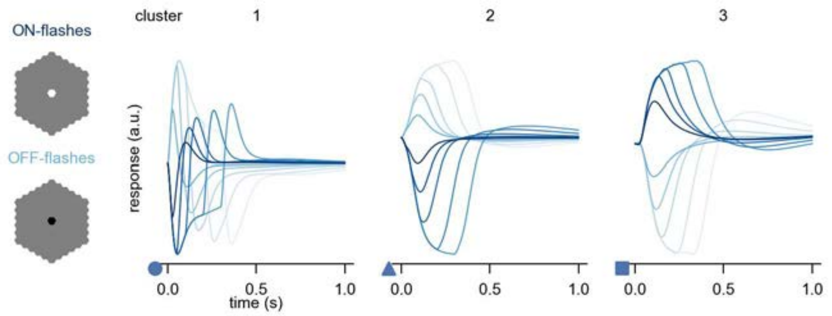

Tm20 - Figure 5: **Cluster-average responses to single-ommatidium flashes.** Responses to single-ommatidium ON-flashes (dark blue shades) and single-ommatidium OFF-flashes (light blue shades) of 20ms, 50ms, 100ms, 200ms, 300ms duration. The flashes occur at second zero.

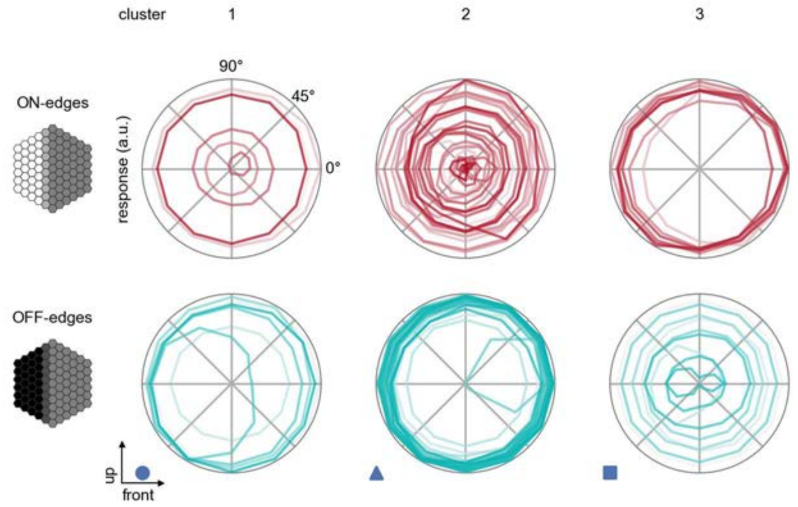

Tm20 - Figure 6: **Peak responses to moving edges.** The top row shows peak responses to moving ON-edges (red), the bottom row shows peak responses to moving OFF-edges (turqoise). The peak responses are averaged over edge-speeds. Edge-stimuli move in different directions from 0 to 360 degrees. The responses from the different models in the different clusters (columns) overlay. Responses from better task-performing models are more saturated.

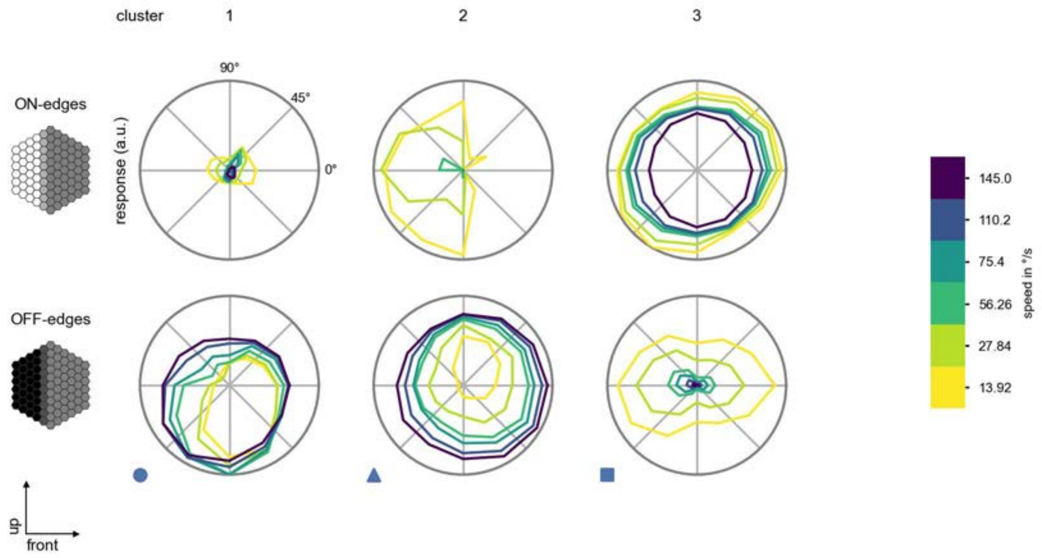

Tm20 - Figure 7: **Peak responses to moving edges from task-optimal models.** The top row shows peak responses to moving ON-edges, the bottom row shows peak responses to moving OFF-edges of varying speeds from 13.92°/s to 145°/s (yellow to dark blue). The edge-stimuli move in different directions from 0 to 360 degrees and at different speeds. Responses from the task-optimal model in the respective cluster.

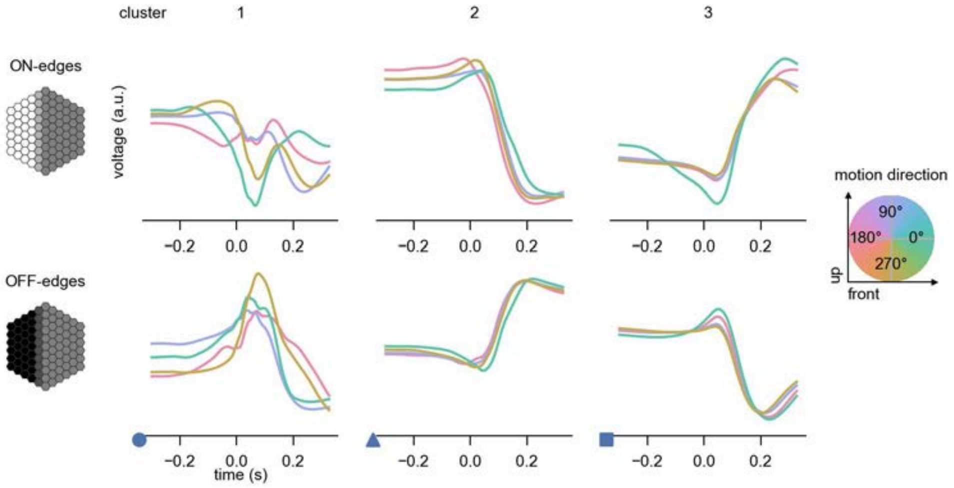

Tm20 - Figure 8: **Responses to moving edges from task-optimal models.** Responses to moving ON-edges (top row) and to moving OFF-edges (bottom row). Edges move in different directions from 0 to 360 degrees and at different speeds. Responses are from the task-optimal model in the respective cluster. Edges moving at 75.4°/s in all cardinal directions (green 0°, blue 90°, red 180°, yellow 270°) from -22.5 to 22.5° visual angle.

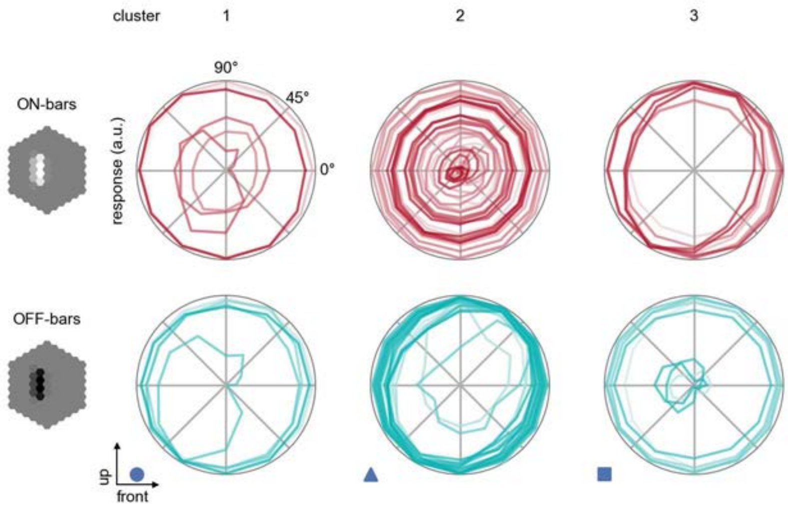

Tm20 - Figure 9: **Peak responses to moving bars.** The top row shows peak responses to moving ON-bars (red), the bottom row shows peak responses to moving OFF-bars (turqoise). The peak responses are averaged over bar-speeds. Bar-stimuli move in different directions from 0 to 360 degrees. The responses from the different models in the different clusters (columns) overlay. Responses from better task-performing models are more saturated.

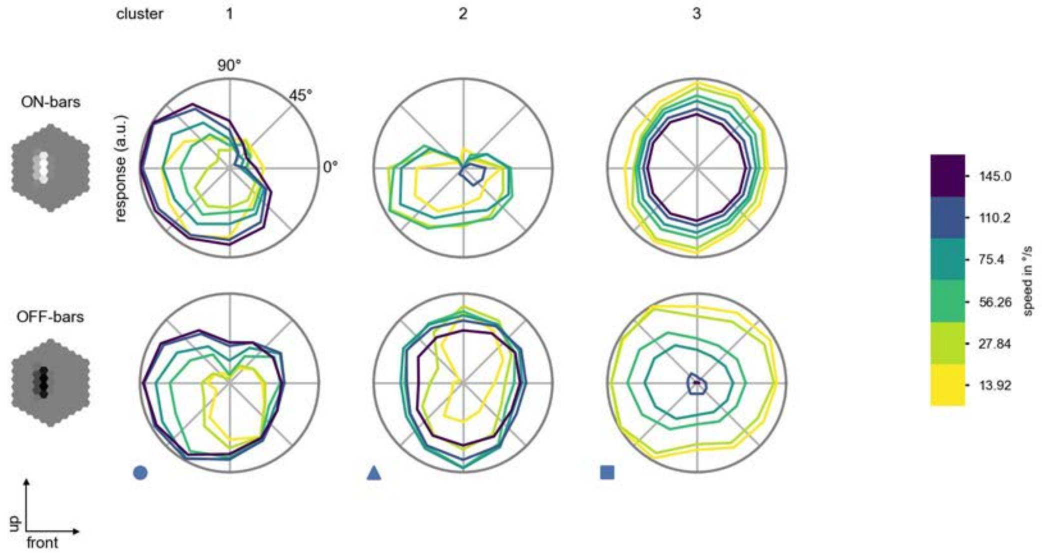

Tm20 - Figure 10: **Peak responses to moving bars from task-optimal models.** The top row shows peak responses to moving ON-bars, the bottom row shows peak responses to moving OFF-bars of varying speeds from 13.92°/s to 145°/s (yellow to dark blue). The bar-stimuli move in different directions from 0 to 360 degrees and at different speeds. Responses from the task-optimal model in the respective cluster.

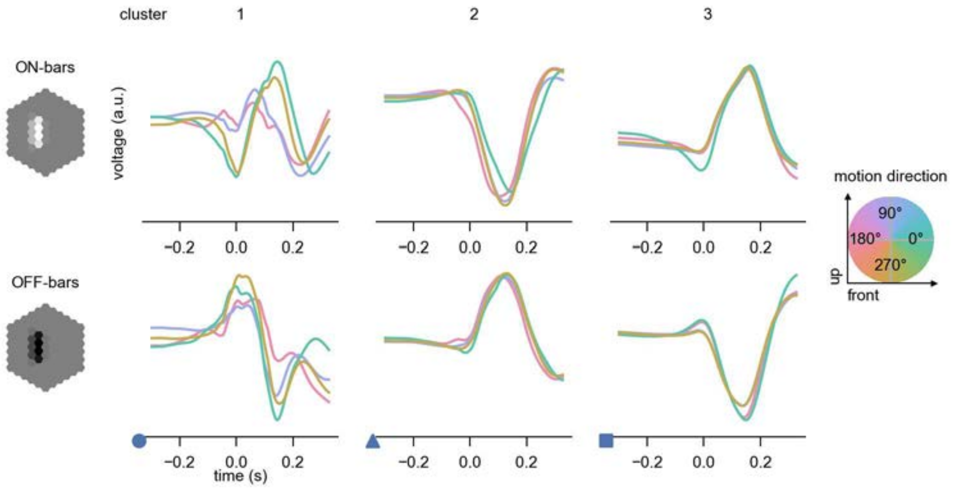

Tm20 - Figure 11: **Responses to moving bars from task-optimal models.** Responses to moving ON-bars (top row) and to moving OFF-bars (bottom row). Bars move in different directions from 0 to 360 degrees and at different speeds. Responses are from the task-optimal model in the respective cluster. Bars moving at 75.4°/s in all cardinal directions (green 0°, blue 90°, red 180°, yellow 270°) from -22.5 to 22.5° visual angle.

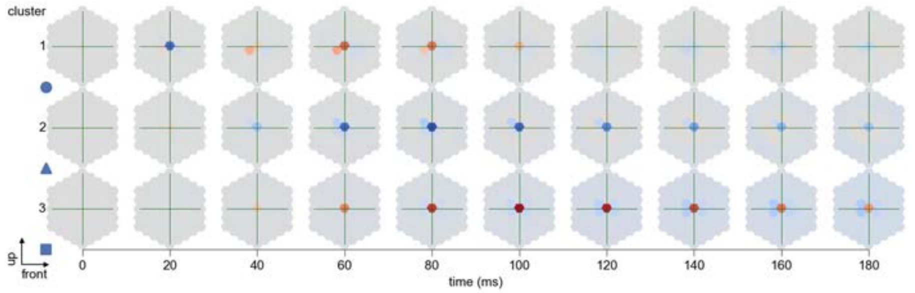

Tm20 - Figure 12: **Spatio-temporal receptive field.** Responses of the central cell to ON-impulses (5 ms) at single-ommatidium flash locations. The flash occurs at second zero. Responses from the task-optimal model of the respective cluster (rows). Red indicates depolarization, blue indicates hyperpolarization.

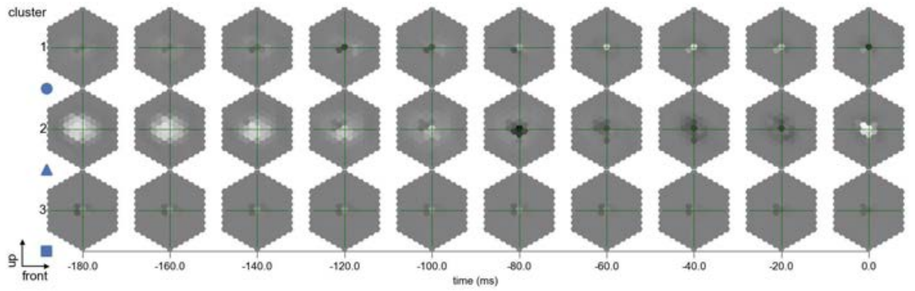

Tm20 - Figure 13: **Maximally excitatory stimuli.** Each row presents the regularized naturalistic-stimulus from the Sintel dataset that maximizes the cell type's central column response at second zero in the task-optimal model of the respective cluster (rows).

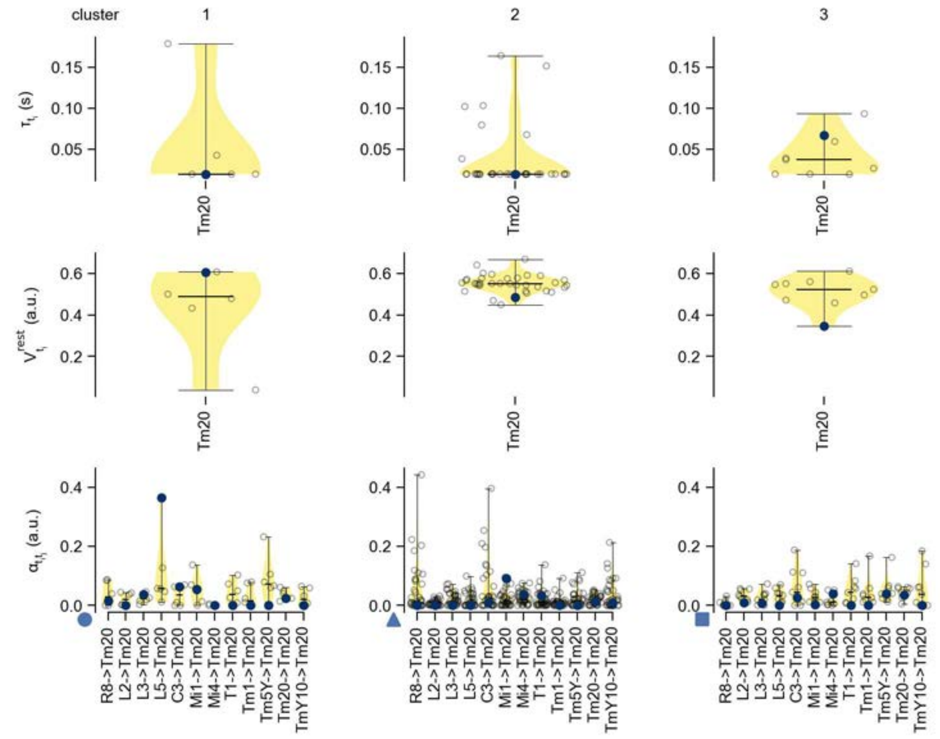

Tm20 - Figure 14: **Task-constrained parameters.** Each column shows the parameters inferred within the respective cluster. First row: learned time constants of the cell type. Second row: resting potentials of the cell type. Third row: scaling factors for the convolutional filters. The blue scatter represents the parameters from the task-optimal model within the cluster.

55 Tm28

← Cell types

Figures

1 Anatomical receptive fields. . . . 396
2 Anatomical projective fields. . . . 396
3 Clustering of the responses to naturalistic stimuli. . . . 397
4 Responses to flashes. . . . 397
5 Cluster-average responses to single-ommatidium flashes. . . . 397
6 Peak responses to moving edges. . . . 398
7 Peak responses to moving edges from task-optimal models. . . . 398
8 Responses to moving edges from task-optimal models. . . . 399
9 Peak responses to moving bars. . . . 399
10 Peak responses to moving bars from task-optimal models. . . . 400
11 Responses to moving bars from task-optimal models. . . . 400
12 Spatio-temporal receptive field. . . . 401
13 Maximally excitatory stimuli. . . . 401
14 Task-constrained parameters. . . . 402

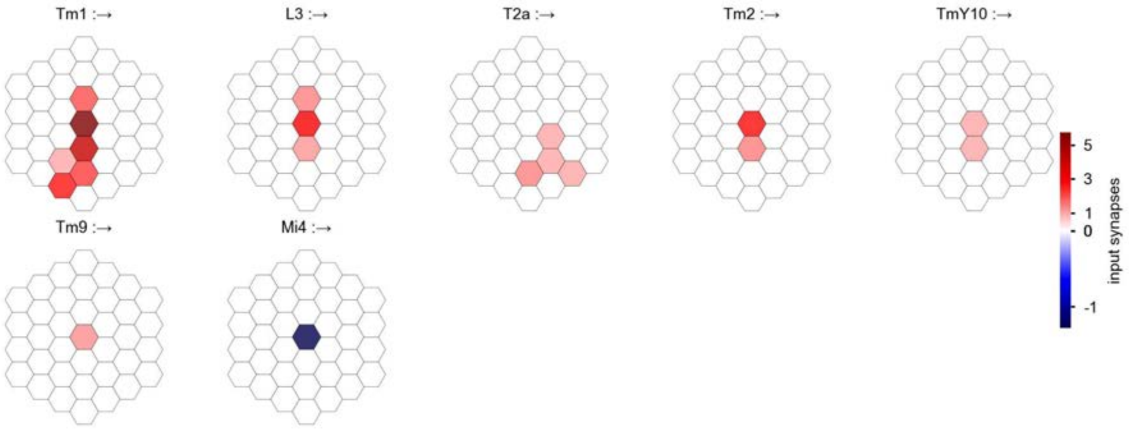

Tm28 - Figure 1: **Anatomical receptive fields.** Each colored hexagon is an input connection, with the connection strength characterized by the average number of synapses that we count from the EM reconstruction. Red indicates excitatory synapses, blue indicates inhibitory synapses from inferred signs. Filters in the order of their total number of synapses.

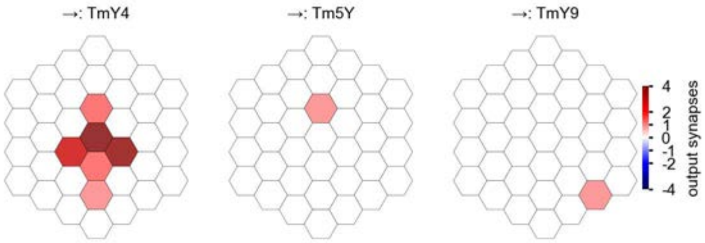

Tm28 - Figure 2: **Anatomical projective fields.** Each colored hexagon is an output connection, with the connection strength characterized by the average number of synapses that we count from the EM reconstruction. Red indicates excitatory synapses, blue indicates inhibitory synapses from inferred signs. Filters in the order of their total number of synapses.

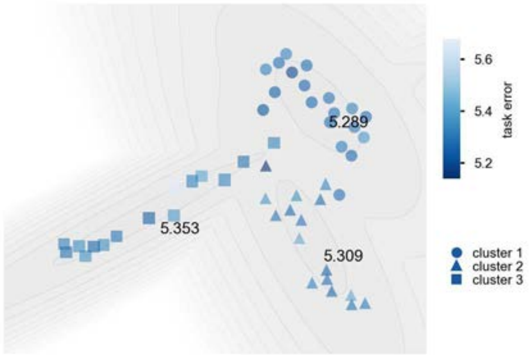

Tm28 - Figure 3: **Clustering of the responses to naturalistic stimuli.** Clustering of the 50 models based on the cell type responses to naturalistic scenes from the Sintel dataset. Scatterpoints represent individual models colored by their task error.

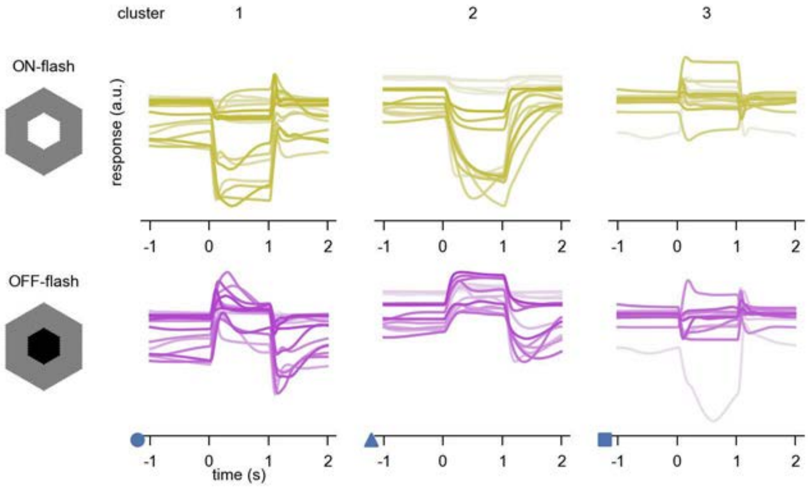

Tm28 - Figure 4: **Responses to flashes.** The top row shows responses to ON-flashes (yellow), the bottom row shows responses to OFF-flashes (magenta). The responses from the 50 different models that are separated into the different clusters (columns) overlay, with better task-performing models on top. Responses from better task-performing models are more saturated. The circular flashes (1s) cover 6 ommatidia in radius and are presented at time zero. Before and after, a grey-stimulus leads to a stationary state of the network.

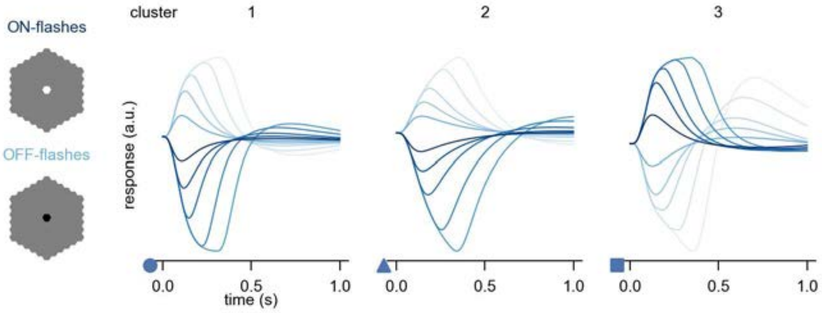

Tm28 - Figure 5: **Cluster-average responses to single-ommatidium flashes.** Responses to single-ommatidium ON-flashes (dark blue shades) and single-ommatidium OFF-flashes (light blue shades) of 20ms, 50ms, 100ms, 200ms, 300ms duration. The flashes occur at second zero.

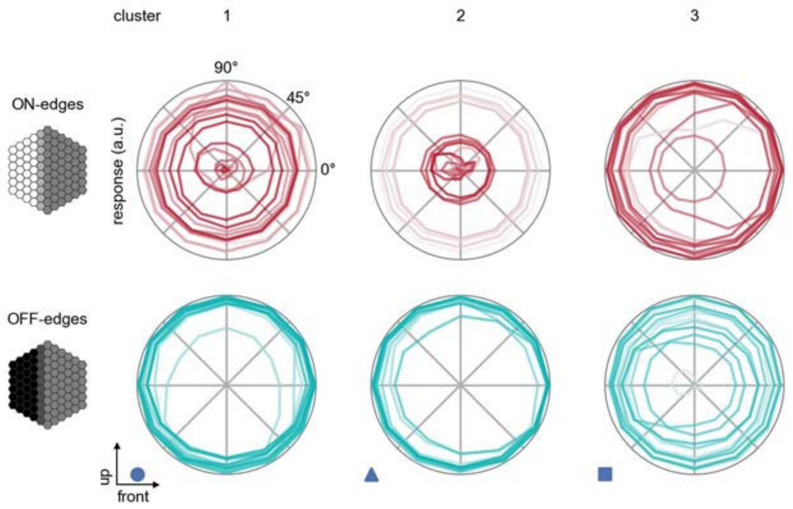

Tm28 - Figure 6: **Peak responses to moving edges.** The top row shows peak responses to moving ON-edges (red), the bottom row shows peak responses to moving OFF-edges (turqoise). The peak responses are averaged over edge-speeds. Edge-stimuli move in different directions from 0 to 360 degrees. The responses from the different models in the different clusters (columns) overlay. Responses from better task-performing models are more saturated.

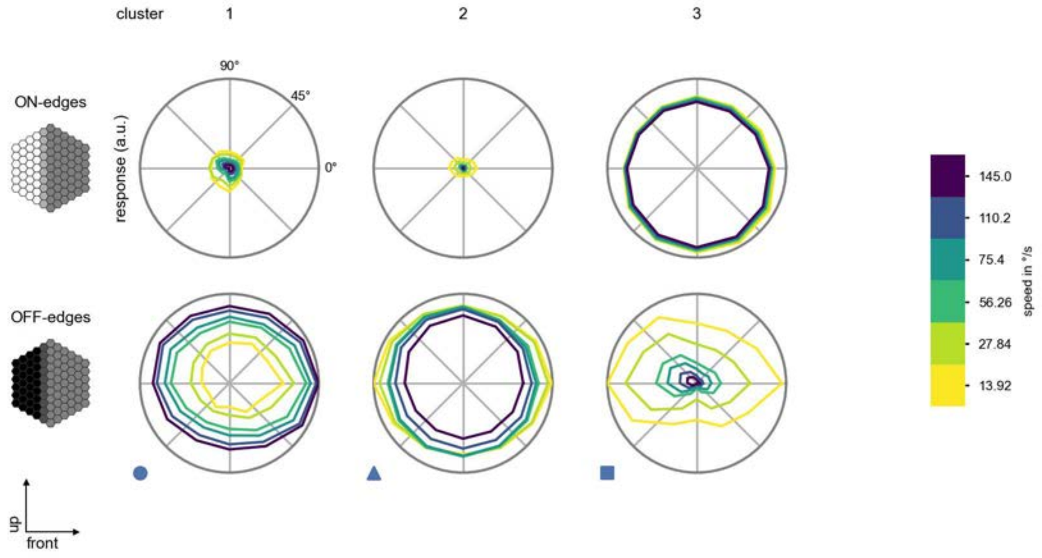

Tm28 - Figure 7: **Peak responses to moving edges from task-optimal models.** The top row shows peak responses to moving ON-edges, the bottom row shows peak responses to moving OFF-edges of varying speeds from 13.92°/s to 145°/s (yellow to dark blue). The edge-stimuli move in different directions from 0 to 360 degrees and at different speeds. Responses from the task-optimal model in the respective cluster.

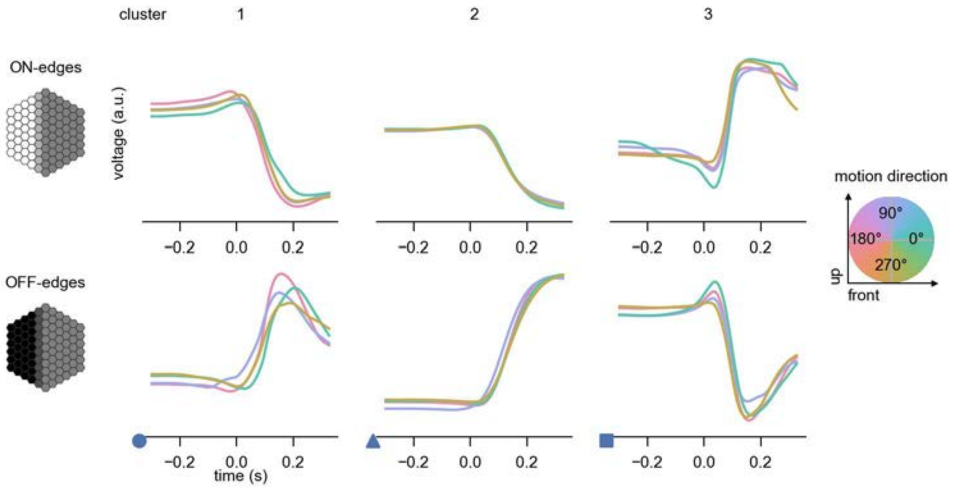

Tm28 - Figure 8: **Responses to moving edges from task-optimal models.** Responses to moving ON-edges (top row) and to moving OFF-edges (bottom row). Edges move in different directions from 0 to 360 degrees and at different speeds. Responses are from the task-optimal model in the respective cluster. Edges moving at 75.4°/s in all cardinal directions (green 0°, blue 90°, red 180°, yellow 270°) from -22.5 to 22.5° visual angle.

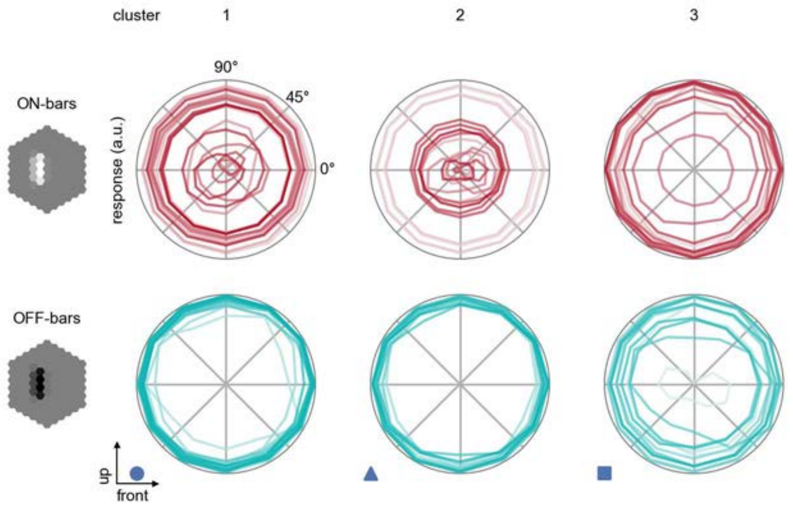

Tm28 - Figure 9: **Peak responses to moving bars.** The top row shows peak responses to moving ON-bars (red), the bottom row shows peak responses to moving OFF-bars (turqoise). The peak responses are averaged over bar-speeds. Bar-stimuli move in different directions from 0 to 360 degrees. The responses from the different models in the different clusters (columns) overlay. Responses from better task-performing models are more saturated.

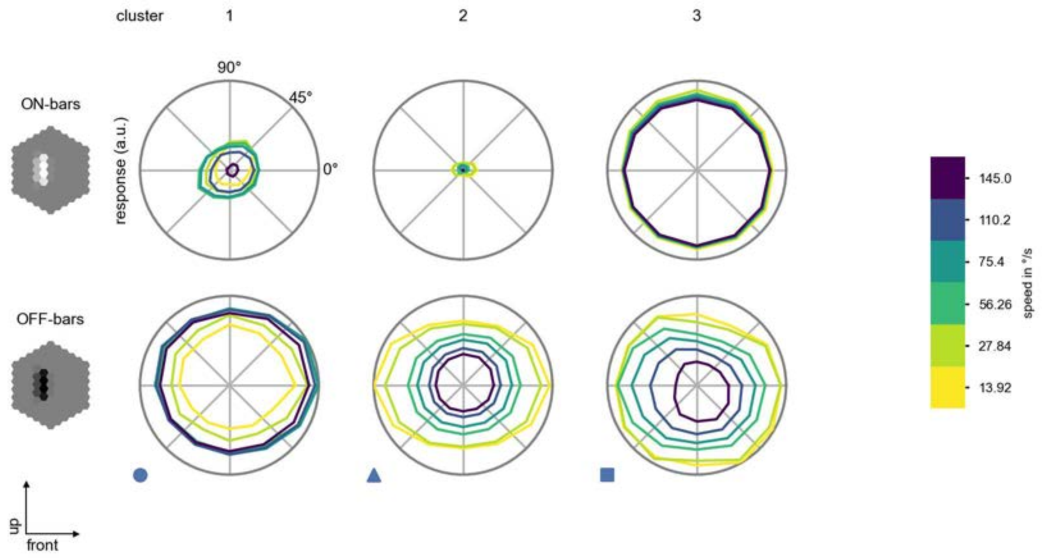

Tm28 - Figure 10: **Peak responses to moving bars from task-optimal models.** The top row shows peak responses to moving ON-bars, the bottom row shows peak responses to moving OFF-bars of varying speeds from 13.92°/s to 145°/s (yellow to dark blue). The bar-stimuli move in different directions from 0 to 360 degrees and at different speeds. Responses from the task-optimal model in the respective cluster.

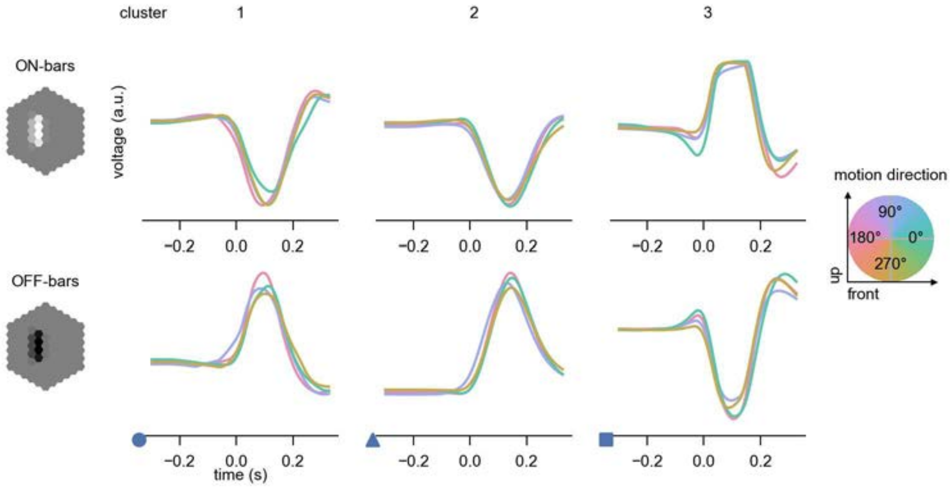

Tm28 - Figure 11: **Responses to moving bars from task-optimal models.** Responses to moving ON-bars (top row) and to moving OFF-bars (bottom row). Bars move in different directions from 0 to 360 degrees and at different speeds. Responses are from the task-optimal model in the respective cluster. Bars moving at 75.4°/s in all cardinal directions (green 0°, blue 90°, red 180°, yellow 270°) from -22.5 to 22.5° visual angle.

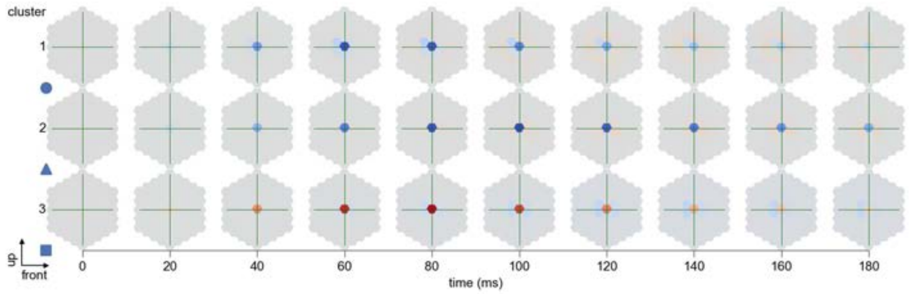

Tm28 - Figure 12: **Spatio-temporal receptive field.** Responses of the central cell to ON-impulses (5 ms) at single-ommatidium flash locations. The flash occurs at second zero. Responses from the task-optimal model of the respective cluster (rows). Red indicates depolarization, blue indicates hyperpolarization.

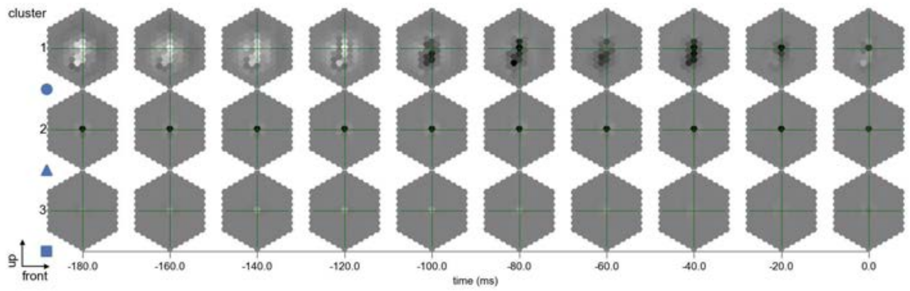

Tm28 - Figure 13: **Maximally excitatory stimuli.** Each row presents the regularized naturalistic-stimulus from the Sintel dataset that maximizes the cell type's central column response at second zero in the task-optimal model of the respective cluster (rows).

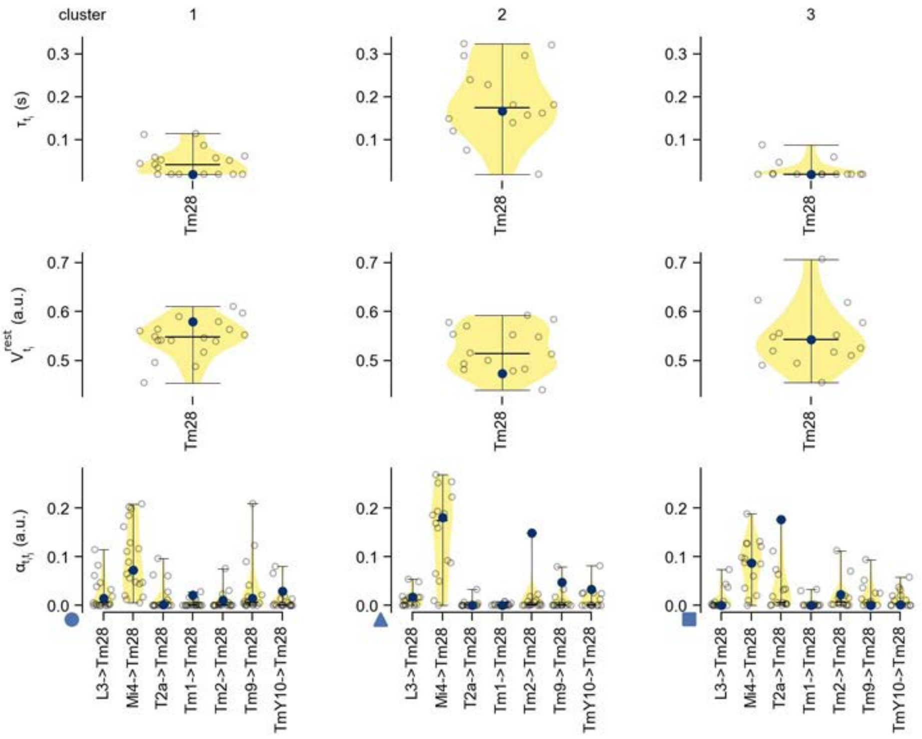

Tm28 - Figure 14: **Task-constrained parameters.** Each column shows the parameters inferred within the respective cluster. First row: learned time constants of the cell type. Second row: resting potentials of the cell type. Third row: scaling factors for the convolutional filters. The blue scatter represents the parameters from the task-optimal model within the cluster.

56 Tm30

← Cell types

Figures

1 Anatomical receptive fields. . . . 403
2 Clustering of the responses to naturalistic stimuli. . . . 403
3 Responses to flashes. . . . 404
4 Cluster-average responses to single-ommatidium flashes. . . . 404
5 Peak responses to moving edges. . . . 405
6 Peak responses to moving edges from task-optimal models. . . . 405
7 Responses to moving edges from task-optimal models. . . . 406
8 Peak responses to moving bars. . . . 406
9 Peak responses to moving bars from task-optimal models. . . . 407
10 Responses to moving bars from task-optimal models. . . . 407
11 Spatio-temporal receptive field. . . . 408
12 Maximally excitatory stimuli. . . . 408
13 Task-constrained parameters. . . . 409

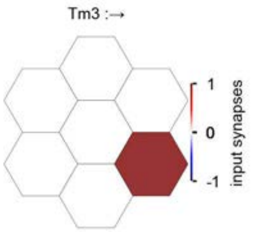

Tm30 - Figure 1: **Anatomical receptive fields.** Each colored hexagon is an input connection, with the connection strength characterized by the average number of synapses that we count from the EM reconstruction. Red indicates excitatory synapses, blue indicates inhibitory synapses from inferred signs. Filters in the order of their total number of synapses.

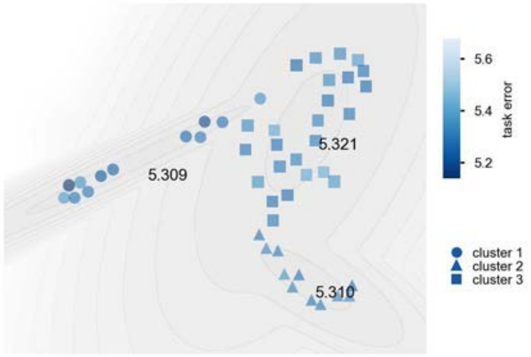

Tm30 - Figure 2: **Clustering of the responses to naturalistic stimuli.** Clustering of the 50 models based on the cell type responses to naturalistic scenes from the Sintel dataset. Scatterpoints represent individual models colored by their task error.

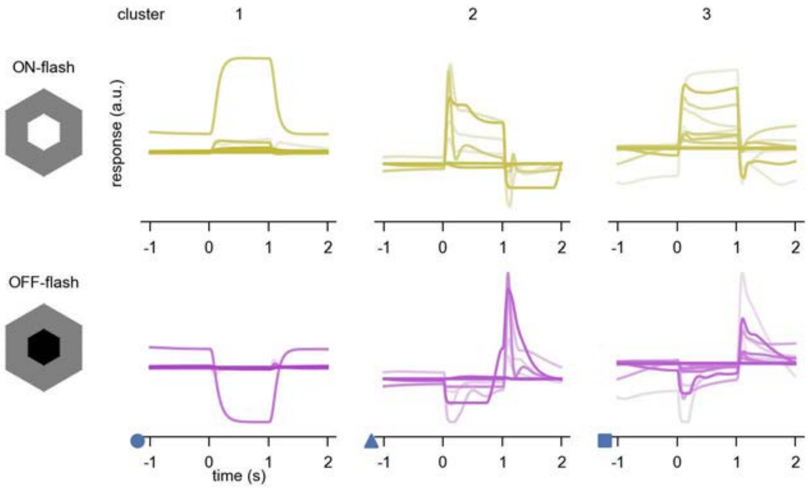

Tm30 - Figure 3: **Responses to flashes.** The top row shows responses to ON-flashes (yellow), the bottom row shows responses to OFF-flashes (magenta). The responses from the 50 different models that are separated into the different clusters (columns) overlay, with better task-performing models on top. Responses from better task-performing models are more saturated. The circular flashes (1s) cover 6 ommatidia in radius and are presented at time zero. Before and after, a grey-stimulus leads to a stationary state of the network.

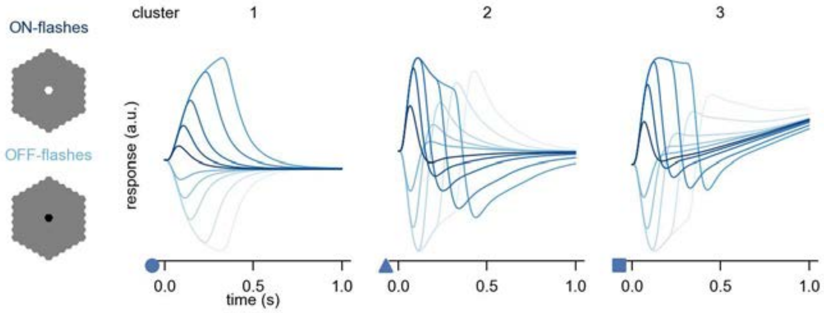

Tm30 - Figure 4: **Cluster-average responses to single-ommatidium flashes.** Responses to single-ommatidium ON-flashes (dark blue shades) and single-ommatidium OFF-flashes (light blue shades) of 20ms, 50ms, 100ms, 200ms, 300ms duration. The flashes occur at second zero.

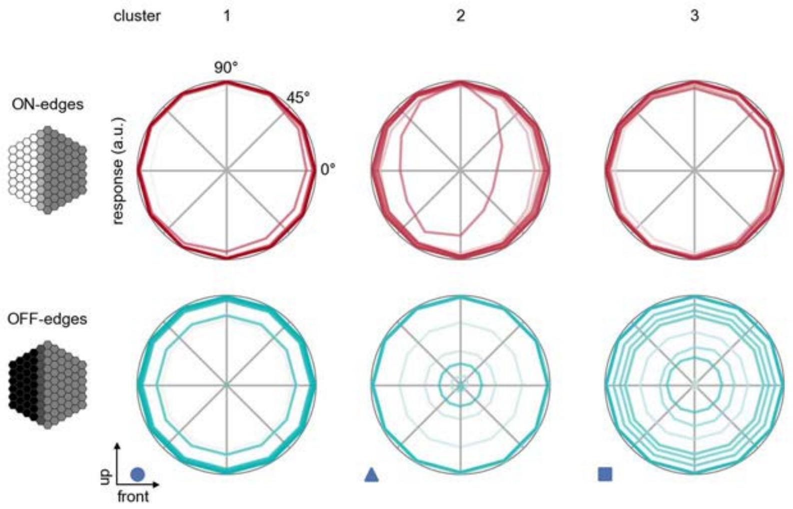

Tm30 - Figure 5: **Peak responses to moving edges.** The top row shows peak responses to moving ON-edges (red), the bottom row shows peak responses to moving OFF-edges (turqoise). The peak responses are averaged over edge-speeds. Edge-stimuli move in different directions from 0 to 360 degrees. The responses from the different models in the different clusters (columns) overlay. Responses from better task-performing models are more saturated.

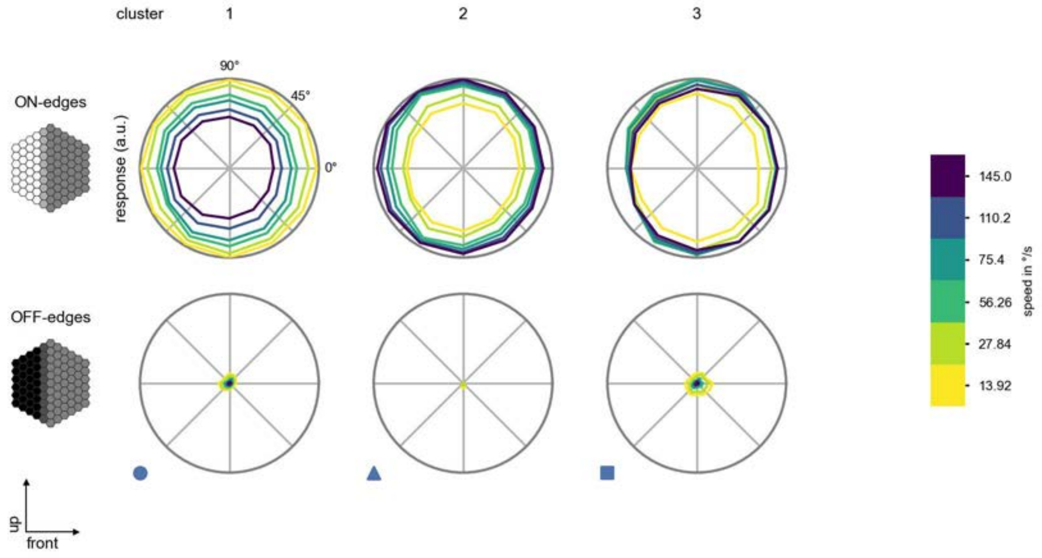

Tm30 - Figure 6: **Peak responses to moving edges from task-optimal models.** The top row shows peak responses to moving ON-edges, the bottom row shows peak responses to moving OFF-edges of varying speeds from 13.92°/s to 145°/s (yellow to dark blue). The edge-stimuli move in different directions from 0 to 360 degrees and at different speeds. Responses from the task-optimal model in the respective cluster.

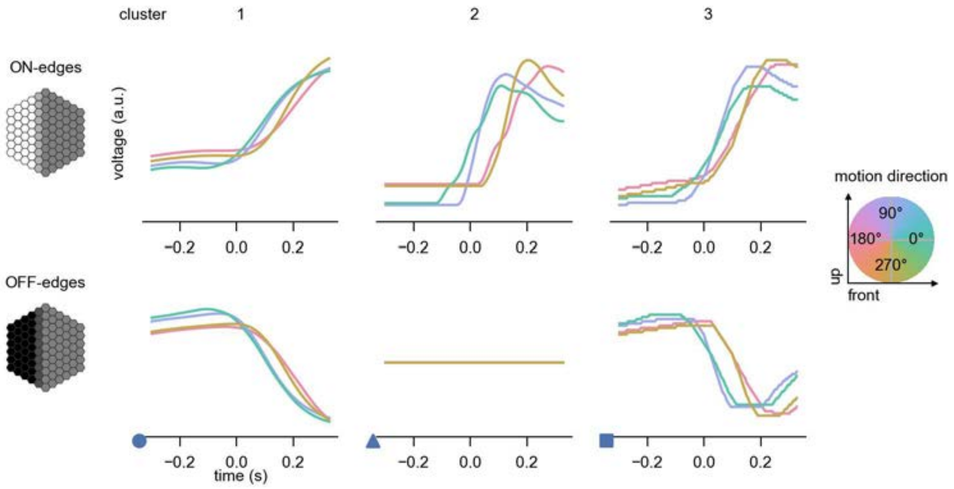

Tm30 - Figure 7: **Responses to moving edges from task-optimal models.** Responses to moving ON-edges (top row) and to moving OFF-edges (bottom row). Edges move in different directions from 0 to 360 degrees and at different speeds. Responses are from the task-optimal model in the respective cluster. Edges moving at 75.4°/s in all cardinal directions (green 0°, blue 90°, red 180°, yellow 270°) from -22.5 to 22.5° visual angle.

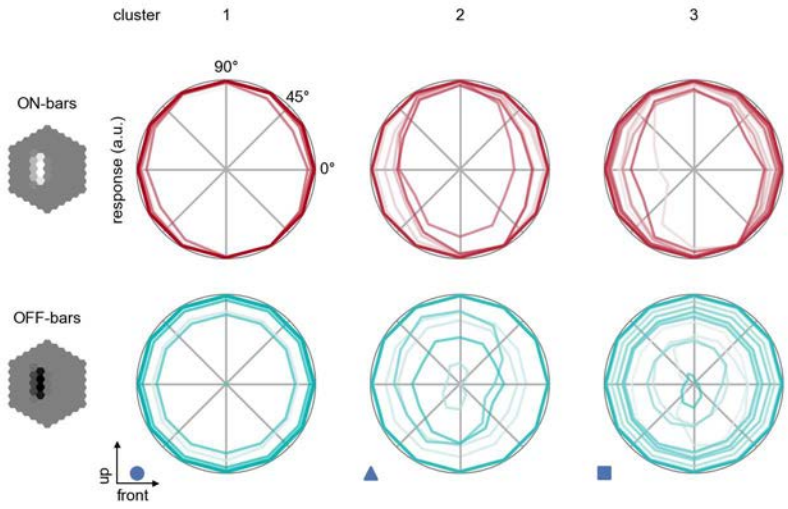

Tm30 - Figure 8: **Peak responses to moving bars.** The top row shows peak responses to moving ON-bars (red), the bottom row shows peak responses to moving OFF-bars (turqoise). The peak responses are averaged over bar-speeds. Bar-stimuli move in different directions from 0 to 360 degrees. The responses from the different models in the different clusters (columns) overlay. Responses from better task-performing models are more saturated.

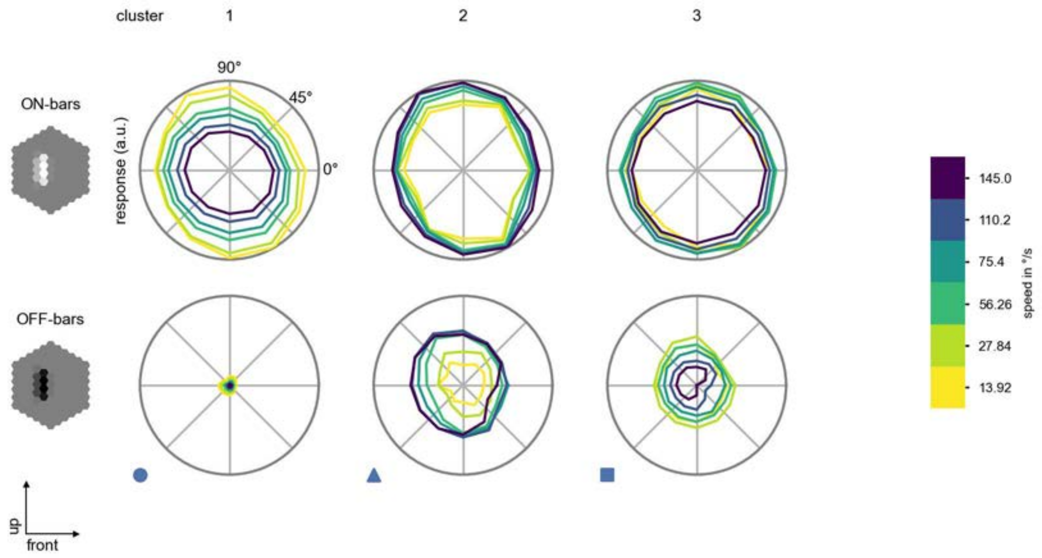

Tm30 - Figure 9: **Peak responses to moving bars from task-optimal models.** The top row shows peak responses to moving ON-bars, the bottom row shows peak responses to moving OFF-bars of varying speeds from 13.92°/s to 145°/s (yellow to dark blue). The bar-stimuli move in different directions from 0 to 360 degrees and at different speeds. Responses from the task-optimal model in the respective cluster.

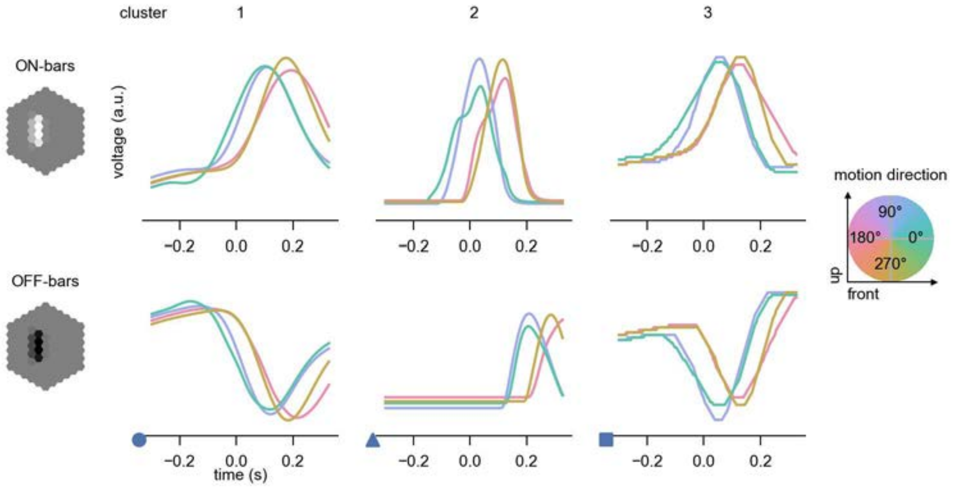

Tm30 - Figure 10: **Responses to moving bars from task-optimal models.** Responses to moving ON-bars (top row) and to moving OFF-bars (bottom row). Bars move in different directions from 0 to 360 degrees and at different speeds. Responses are from the task-optimal model in the respective cluster. Bars moving at 75.4°/s in all cardinal directions (green 0°, blue 90°, red 180°, yellow 270°) from -22.5 to 22.5° visual angle.

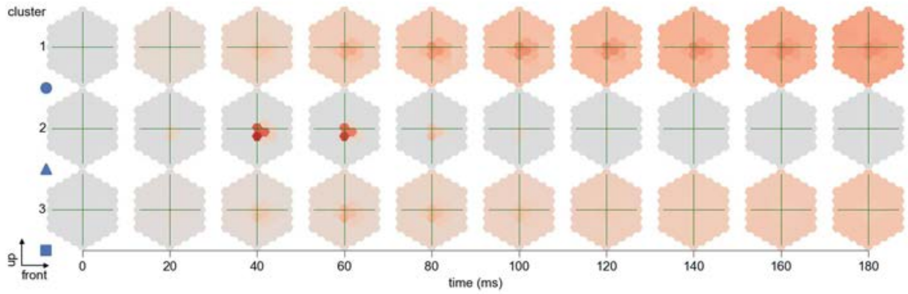

Tm30 - Figure 11: **Spatio-temporal receptive field.** Responses of the central cell to ON-impulses (5 ms) at single-ommatidium flash locations. The flash occurs at second zero. Responses from the task-optimal model of the respective cluster (rows). Red indicates depolarization, blue indicates hyperpolarization.

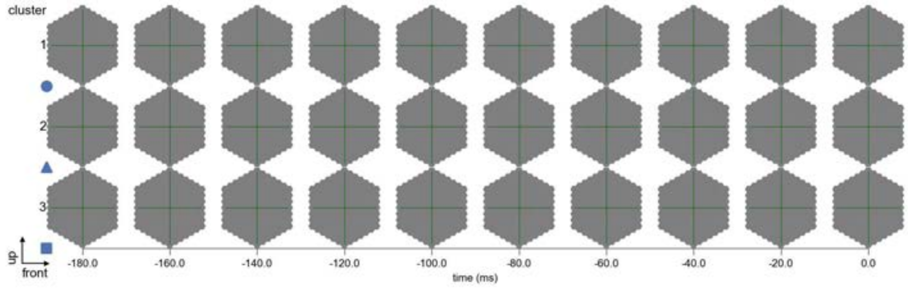

Tm30 - Figure 12: **Maximally excitatory stimuli.** Each row presents the regularized naturalistic-stimulus from the Sintel dataset that maximizes the cell type's central column response at second zero in the task-optimal model of the respective cluster (rows).

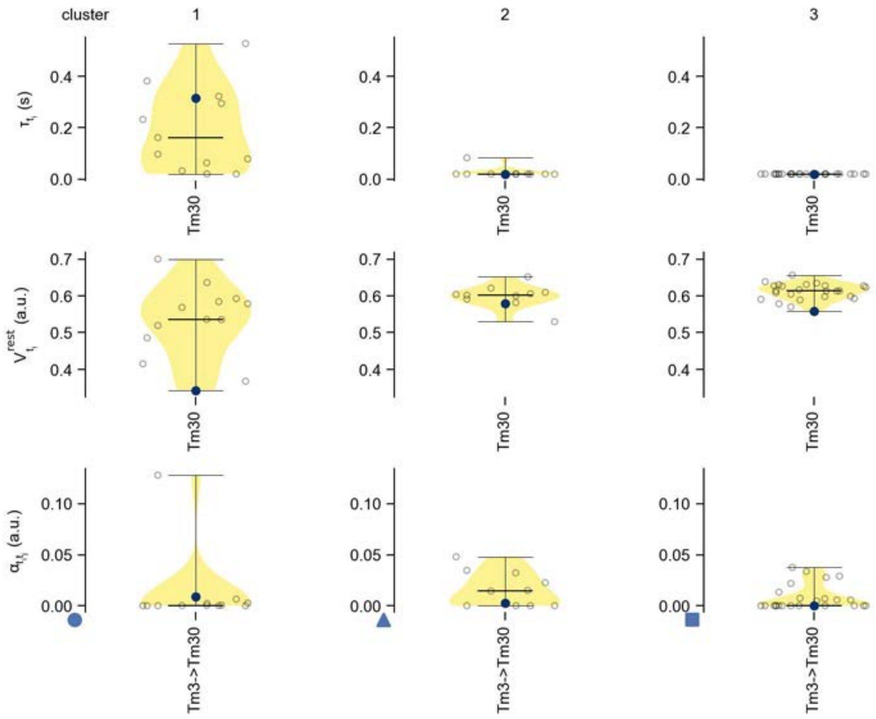

Tm30 - Figure 13: **Task-constrained parameters.** Each column shows the parameters inferred within the respective cluster. First row: learned time constants of the cell type. Second row: resting potentials of the cell type. Third row: scaling factors for the convolutional filters. The blue scatter represents the parameters from the task-optimal model within the cluster.

57 TmY3

← Cell types

Figures

1 Anatomical receptive fields. . . . 410
2 Anatomical projective fields. . . . 411
3 Clustering of the responses to naturalistic stimuli. . . . 411
4 Responses to flashes. . . . 412
5 Cluster-average responses to single-ommatidium flashes. . . . 412
6 Peak responses to moving edges. . . . 413
7 Peak responses to moving edges from task-optimal models. . . . 413
8 Responses to moving edges from task-optimal models. . . . 414
9 Peak responses to moving bars. . . . 414
10 Peak responses to moving bars from task-optimal models. . . . 415
11 Responses to moving bars from task-optimal models. . . . 415
12 Spatio-temporal receptive field. . . . 416
13 Maximally excitatory stimuli. . . . 416
14 Task-constrained parameters. . . . 417

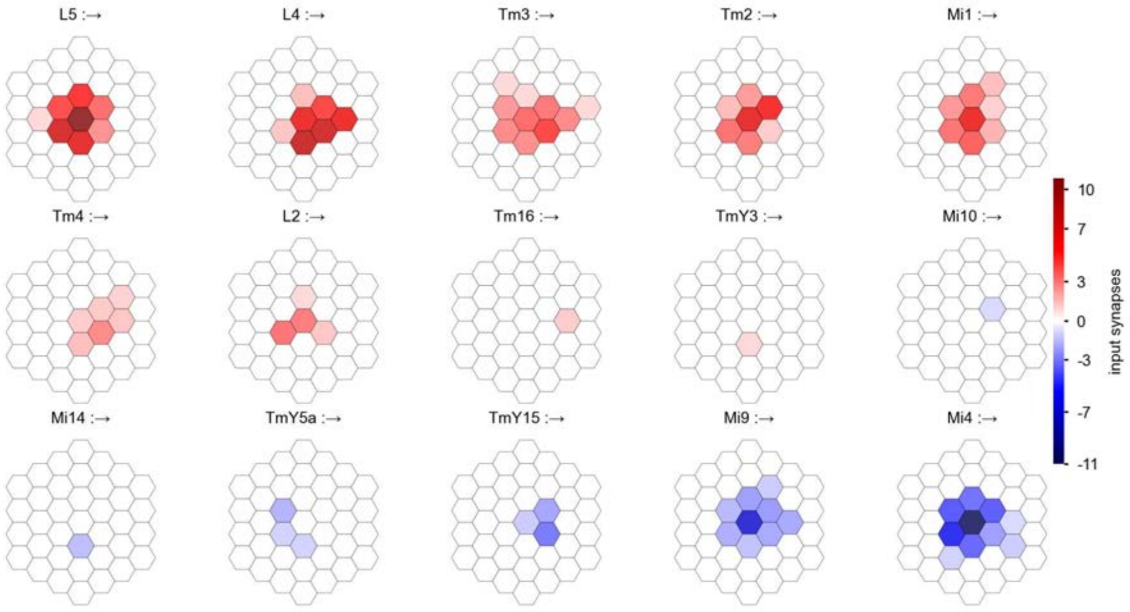

TmY3 - Figure 1: **Anatomical receptive fields.** Each colored hexagon is an input connection, with the connection strength characterized by the average number of synapses that we count from the EM reconstruction. Red indicates excitatory synapses, blue indicates inhibitory synapses from inferred signs. Filters in the order of their total number of synapses.

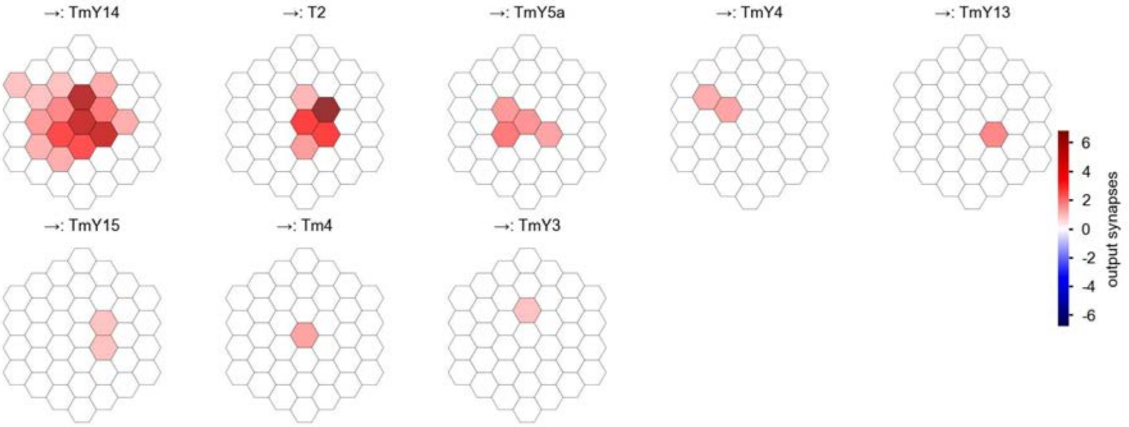

TmY3 - Figure 2: **Anatomical projective fields.** Each colored hexagon is an output connection, with the connection strength characterized by the average number of synapses that we count from the EM reconstruction. Red indicates excitatory synapses, blue indicates inhibitory synapses from inferred signs. Filters in the order of their total number of synapses.

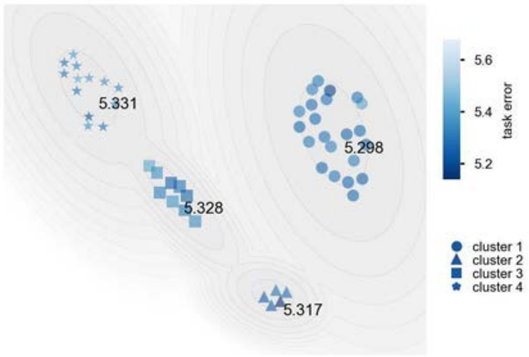

TmY3 - Figure 3: **Clustering of the responses to naturalistic stimuli.** Clustering of the 50 models based on the cell type responses to naturalistic scenes from the Sintel dataset. Scatterpoints represent individual models colored by their task error.

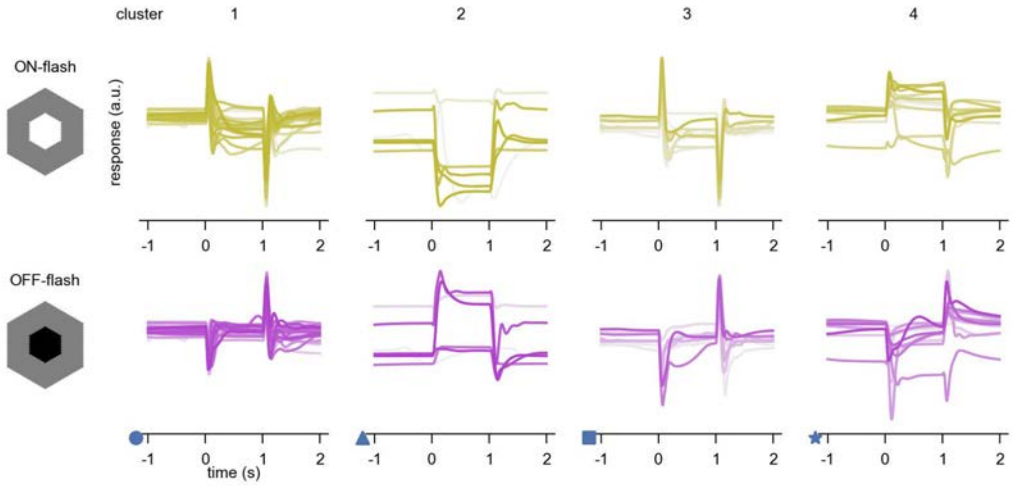

TmY3 - Figure 4: **Responses to flashes.** The top row shows responses to ON-flashes (yellow), the bottom row shows responses to OFF-flashes (magenta). The responses from the 50 different models that are separated into the different clusters (columns) overlay, with better task-performing models on top. Responses from better task-performing models are more saturated. The circular flashes (1s) cover 6 ommatidia in radius and are presented at time zero. Before and after, a grey-stimulus leads to a stationary state of the network.

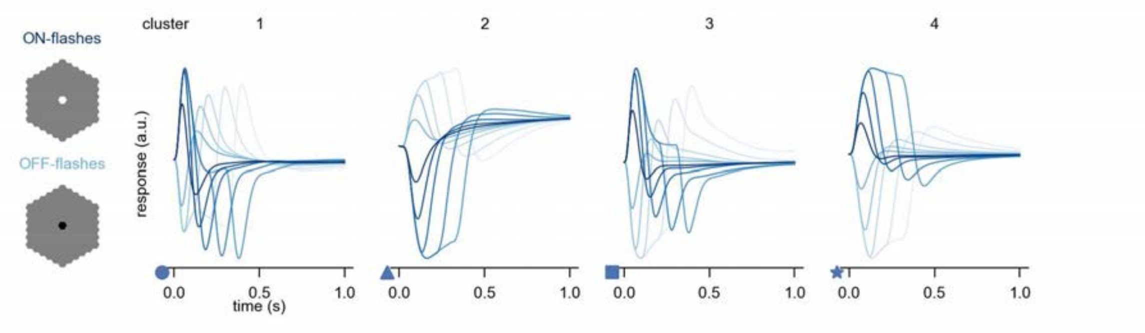

TmY3 - Figure 5: **Cluster-average responses to single-ommatidium flashes.** Responses to single-ommatidium ON-flashes (dark blue shades) and single-ommatidium OFF-flashes (light blue shades) of 20ms, 50ms, 100ms, 200ms, 300ms duration. The flashes occur at second zero.

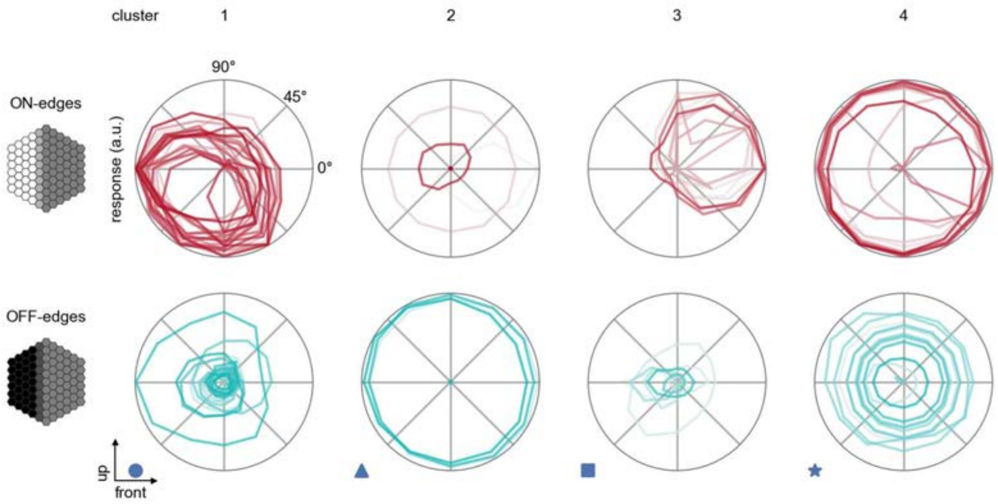

TmY3 - Figure 6: **Peak responses to moving edges.** The top row shows peak responses to moving ON-edges (red), the bottom row shows peak responses to moving OFF-edges (turqoise). The peak responses are averaged over edge-speeds. Edge-stimuli move in different directions from 0 to 360 degrees. The responses from the different models in the different clusters (columns) overlay. Responses from better task-performing models are more saturated.

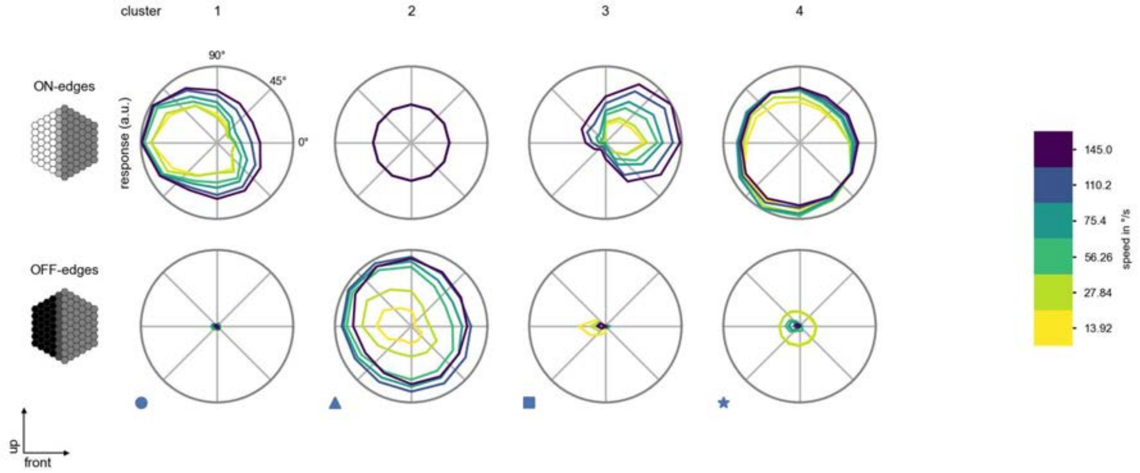

TmY3 - Figure 7: **Peak responses to moving edges from task-optimal models.** The top row shows peak responses to moving ON-edges, the bottom row shows peak responses to moving OFF-edges of varying speeds from 13.92°/s to 145°/s (yellow to dark blue). The edge-stimuli move in different directions from 0 to 360 degrees and at different speeds. Responses from the task-optimal model in the respective cluster.

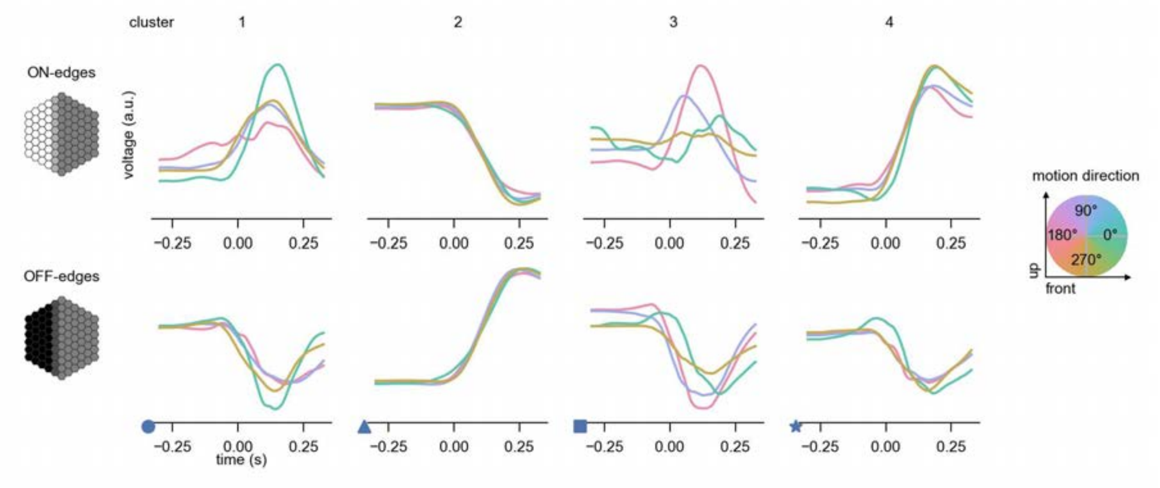

TmY3 - Figure 8: **Responses to moving edges from task-optimal models.** Responses to moving ON-edges (top row) and to moving OFF-edges (bottom row). Edges move in different directions from 0 to 360 degrees and at different speeds. Responses are from the task-optimal model in the respective cluster. Edges moving at 75.4°/s in all cardinal directions (green 0°, blue 90°, red 180°, yellow 270°) from -22.5 to 22.5° visual angle.

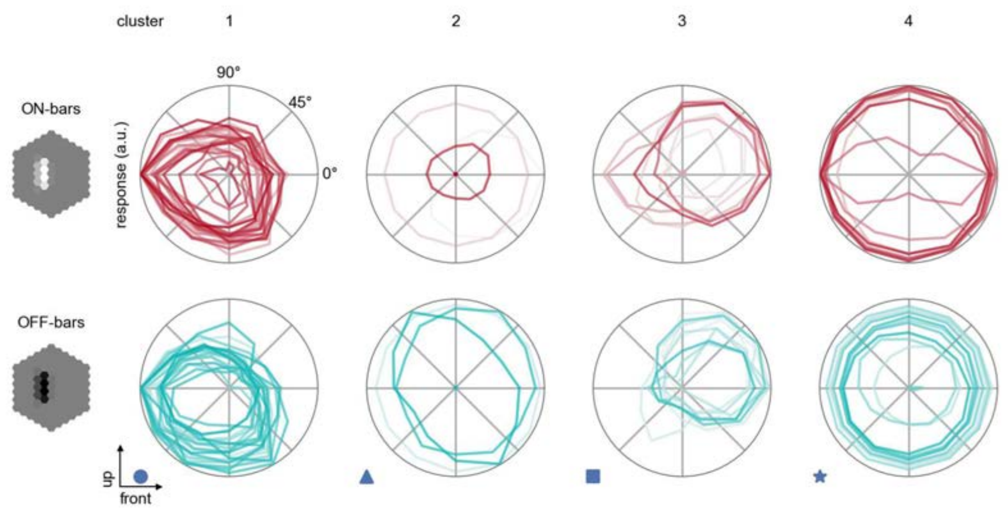

TmY3 - Figure 9: **Peak responses to moving bars.** The top row shows peak responses to moving ON-bars (red), the bottom row shows peak responses to moving OFF-bars (turqoise). The peak responses are averaged over bar-speeds. Bar-stimuli move in different directions from 0 to 360 degrees. The responses from the different models in the different clusters (columns) overlay. Responses from better task-performing models are more saturated.

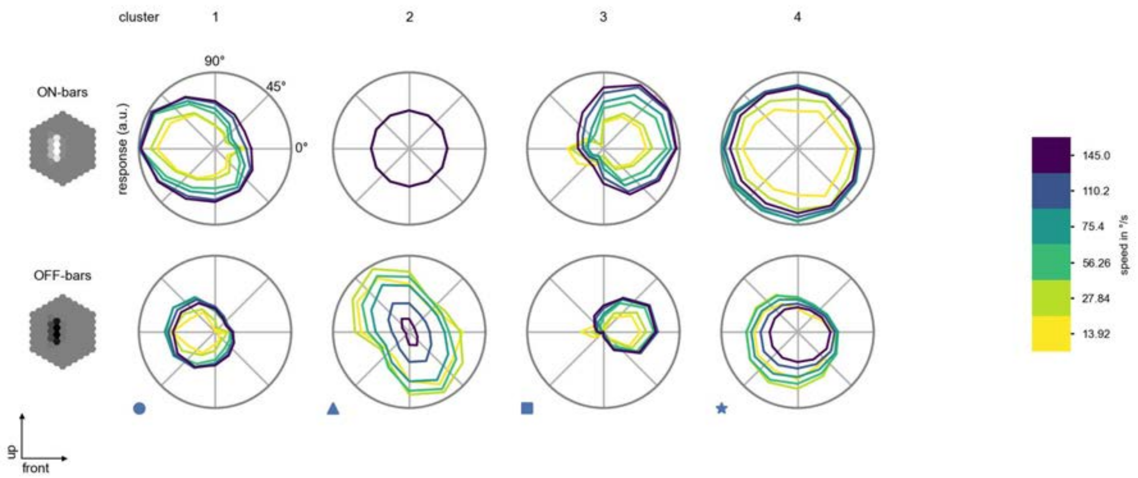

TmY3 - Figure 10: **Peak responses to moving bars from task-optimal models.** The top row shows peak responses to moving ON-bars, the bottom row shows peak responses to moving OFF-bars of varying speeds from 13.92°/s to 145°/s (yellow to dark blue). The bar-stimuli move in different directions from 0 to 360 degrees and at different speeds. Responses from the task-optimal model in the respective cluster.

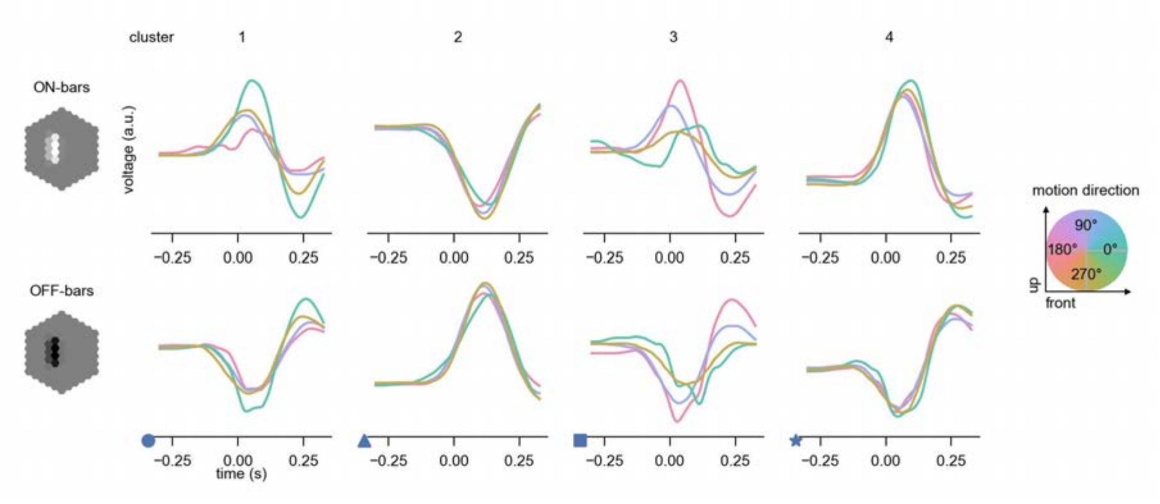

TmY3 - Figure 11: **Responses to moving bars from task-optimal models.** Responses to moving ON-bars (top row) and to moving OFF-bars (bottom row). Bars move in different directions from 0 to 360 degrees and at different speeds. Responses are from the task-optimal model in the respective cluster. Bars moving at 75.4°/s in all cardinal directions (green 0°, blue 90°, red 180°, yellow 270°) from -22.5 to 22.5° visual angle.

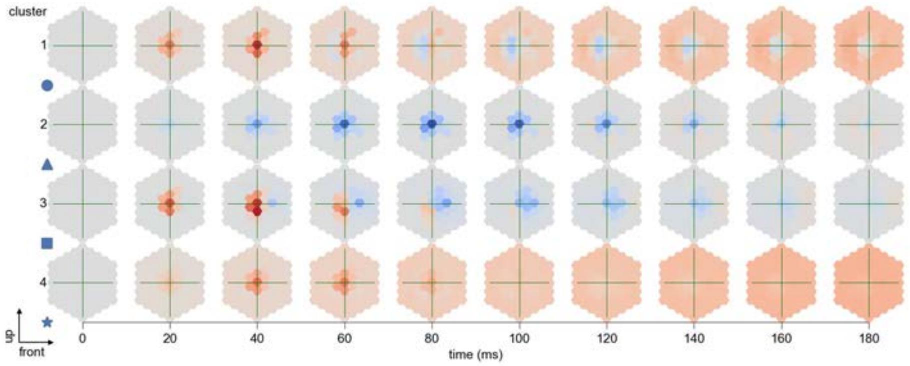

TmY3 - Figure 12: **Spatio-temporal receptive field.** Responses of the central cell to ON-impulses (5 ms) at single-ommatidium flash locations. The flash occurs at second zero. Responses from the task-optimal model of the respective cluster (rows). Red indicates depolarization, blue indicates hyperpolarization.

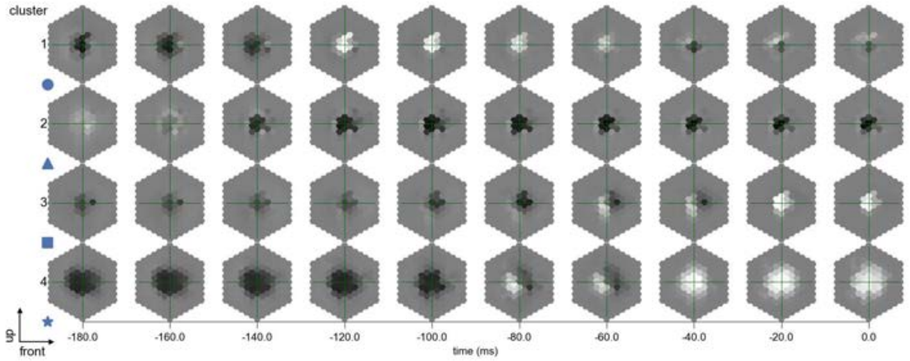

TmY3 - Figure 13: **Maximally excitatory stimuli.** Each row presents the regularized naturalistic-stimulus from the Sintel dataset that maximizes the cell type's central column response at second zero in the task-optimal model of the respective cluster (rows).

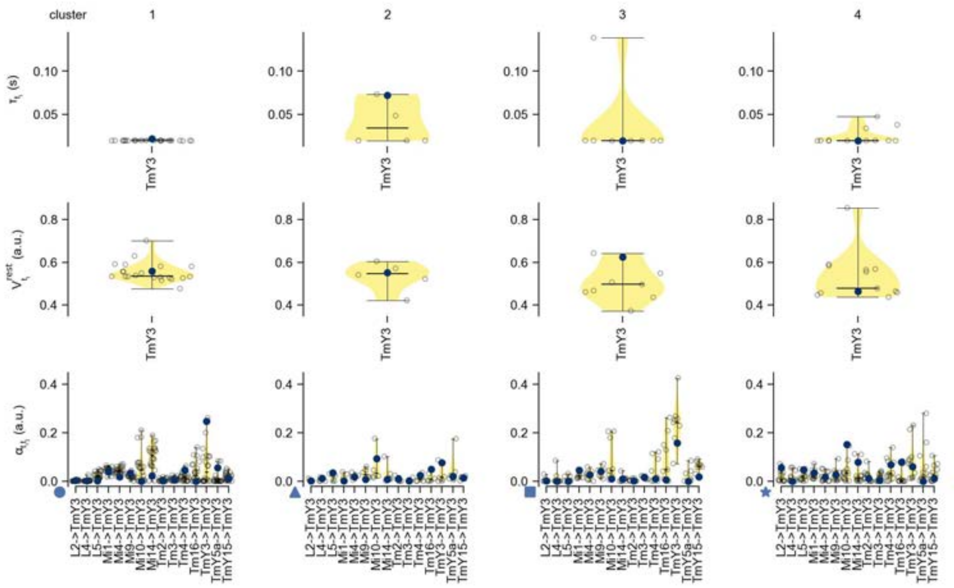

TmY3 - Figure 14: **Task-constrained parameters.** Each column shows the parameters inferred within the respective cluster. First row: learned time constants of the cell type. Second row: resting potentials of the cell type. Third row: scaling factors for the convolutional filters. The blue scatter represents the parameters from the task-optimal model within the cluster.

58 TmY4

← Cell types

Figures

1 Anatomical receptive fields. 418
2 Anatomical projective fields. 419
3 Clustering of the responses to naturalistic stimuli. 419
4 Responses to flashes. 420
5 Cluster-average responses to single-ommatidium flashes. 420
6 Peak responses to moving edges. 421
7 Peak responses to moving edges from task-optimal models. 421
8 Responses to moving edges from task-optimal models. 422
9 Peak responses to moving bars. 422
10 Peak responses to moving bars from task-optimal models. 423
11 Responses to moving bars from task-optimal models. 423
12 Spatio-temporal receptive field. 424
13 Maximally excitatory stimuli. 424
14 Task-constrained parameters. 425

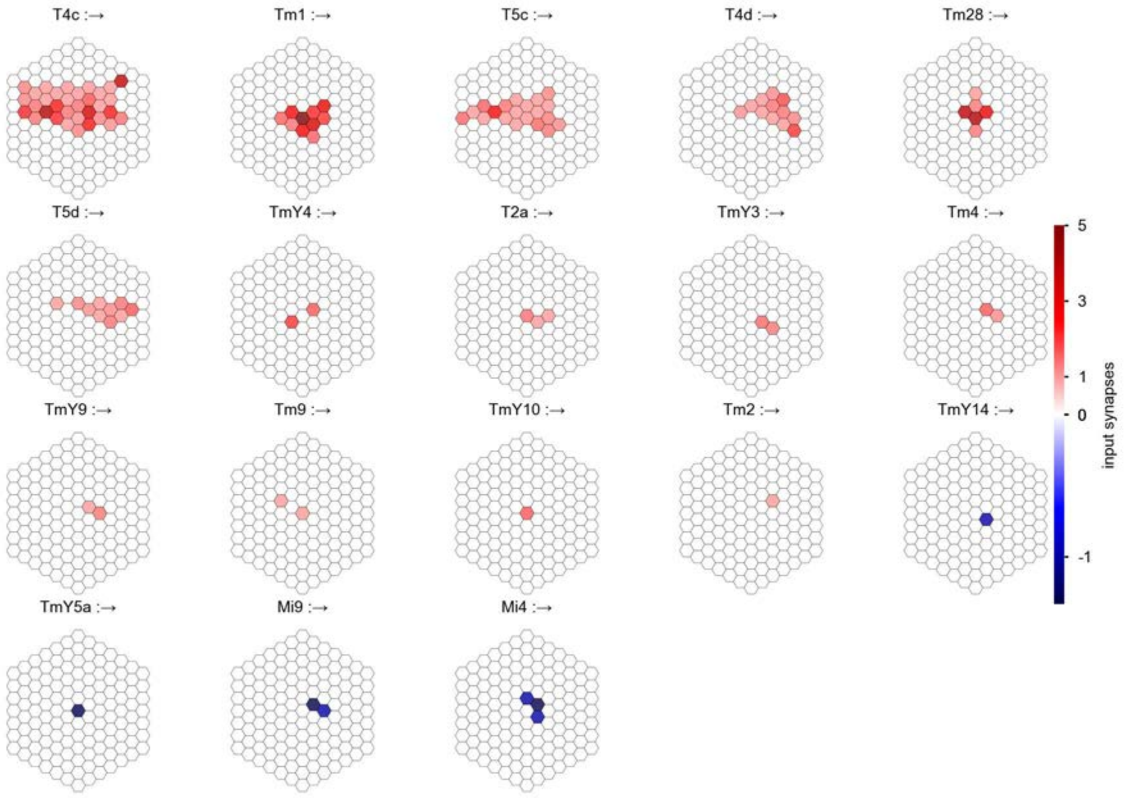

TmY4 - Figure 1: **Anatomical receptive fields.** Each colored hexagon is an input connection, with the connection strength characterized by the average number of synapses that we count from the EM reconstruction. Red indicates excitatory synapses, blue indicates inhibitory synapses from inferred signs. Filters in the order of their total number of synapses.

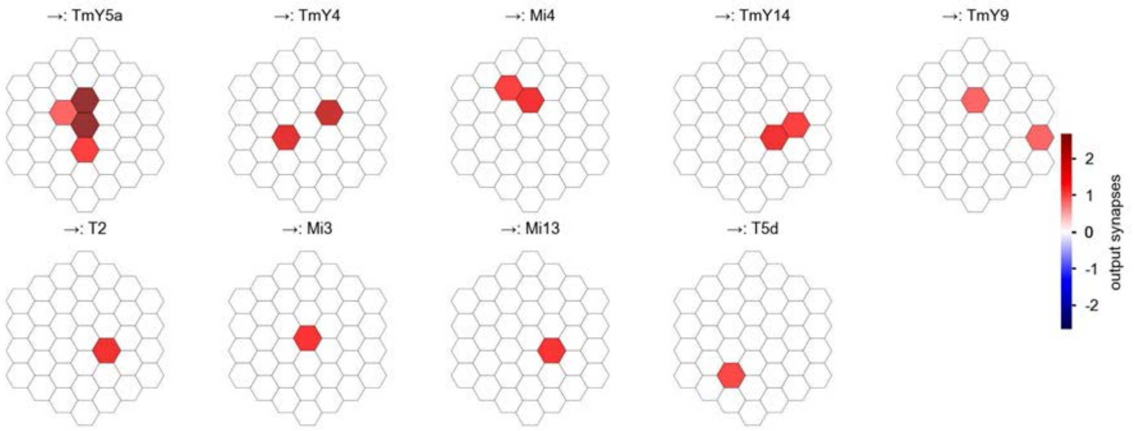

TmY4 - Figure 2: **Anatomical projective fields.** Each colored hexagon is an output connection, with the connection strength characterized by the average number of synapses that we count from the EM reconstruction. Red indicates excitatory synapses, blue indicates inhibitory synapses from inferred signs. Filters in the order of their total number of synapses.

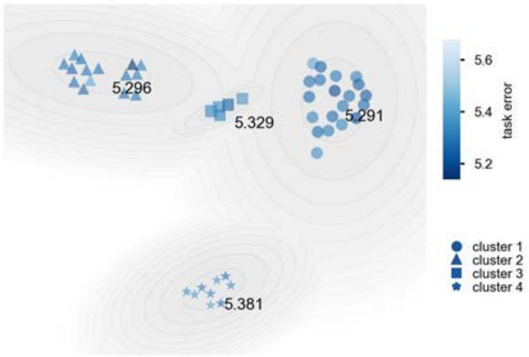

TmY4 - Figure 3: **Clustering of the responses to naturalistic stimuli.** Clustering of the 50 models based on the cell type responses to naturalistic scenes from the Sintel dataset. Scatterpoints represent individual models colored by their task error.

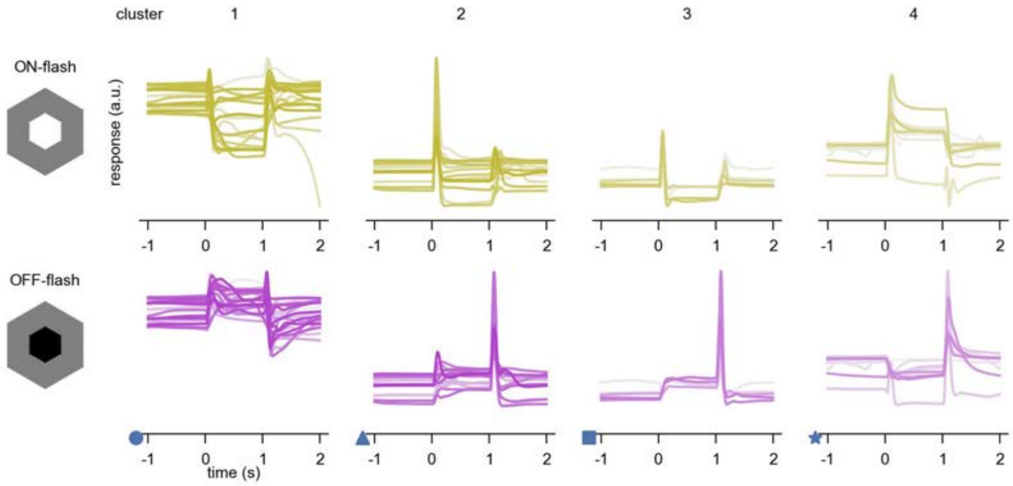

TmY4 - Figure 4: **Responses to flashes.** The top row shows responses to ON-flashes (yellow), the bottom row shows responses to OFF-flashes (magenta). The responses from the 50 different models that are separated into the different clusters (columns) overlay, with better task-performing models on top. Responses from better task-performing models are more saturated. The circular flashes (1s) cover 6 ommatidia in radius and are presented at time zero. Before and after, a grey-stimulus leads to a stationary state of the network.

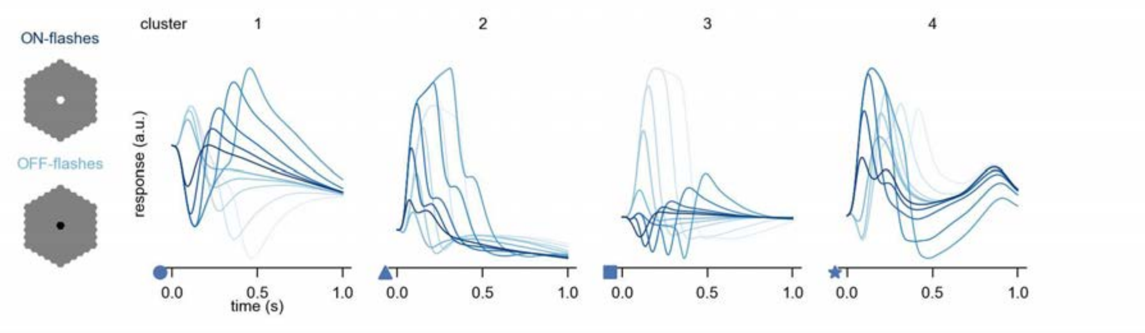

TmY4 - Figure 5: **Cluster-average responses to single-ommatidium flashes.** Responses to single-ommatidium ON-flashes (dark blue shades) and single-ommatidium OFF-flashes (light blue shades) of 20ms, 50ms, 100ms, 200ms, 300ms duration. The flashes occur at second zero.

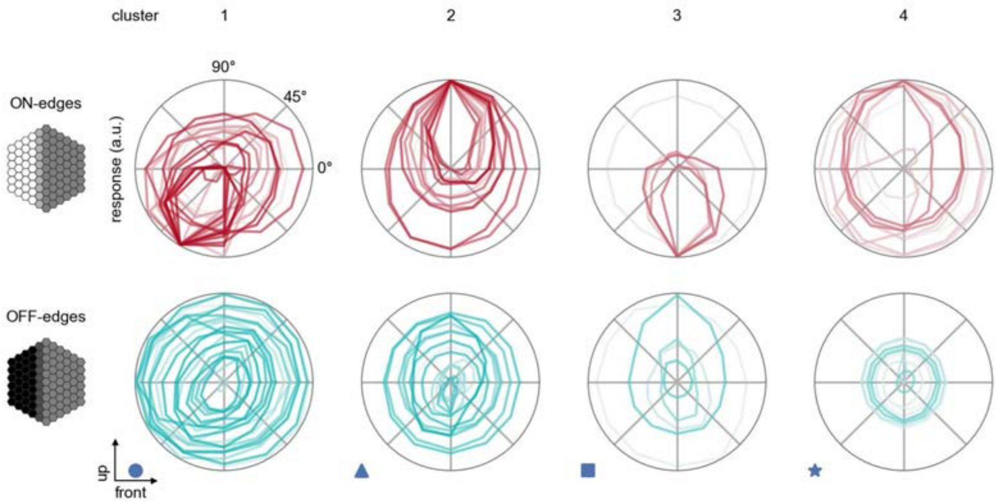

TmY4 - Figure 6: **Peak responses to moving edges.** The top row shows peak responses to moving ON-edges (red), the bottom row shows peak responses to moving OFF-edges (turqoise). The peak responses are averaged over edge-speeds. Edge-stimuli move in different directions from 0 to 360 degrees. The responses from the different models in the different clusters (columns) overlay. Responses from better task-performing models are more saturated.

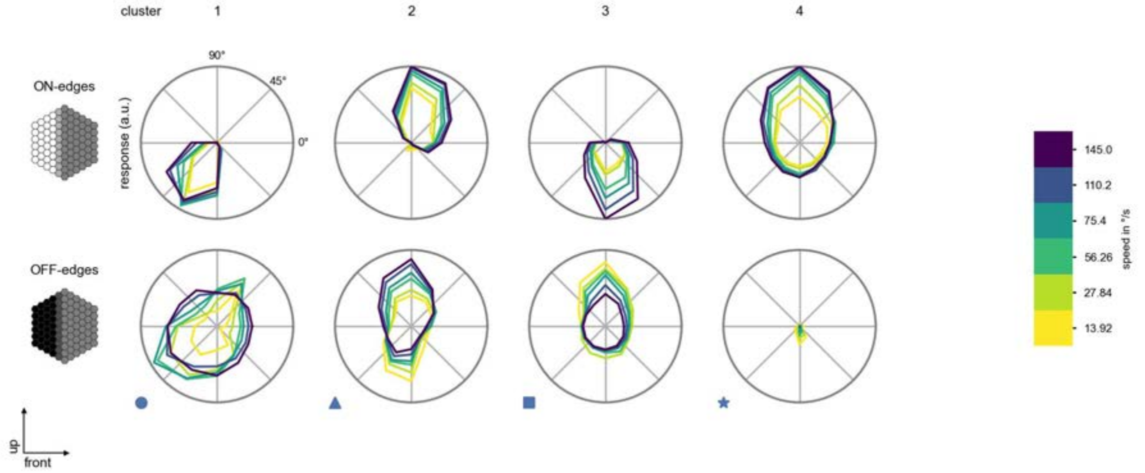

TmY4 - Figure 7: **Peak responses to moving edges from task-optimal models.** The top row shows peak responses to moving ON-edges, the bottom row shows peak responses to moving OFF-edges of varying speeds from 13.92°/s to 145°/s (yellow to dark blue). The edge-stimuli move in different directions from 0 to 360 degrees and at different speeds. Responses from the task-optimal model in the respective cluster.

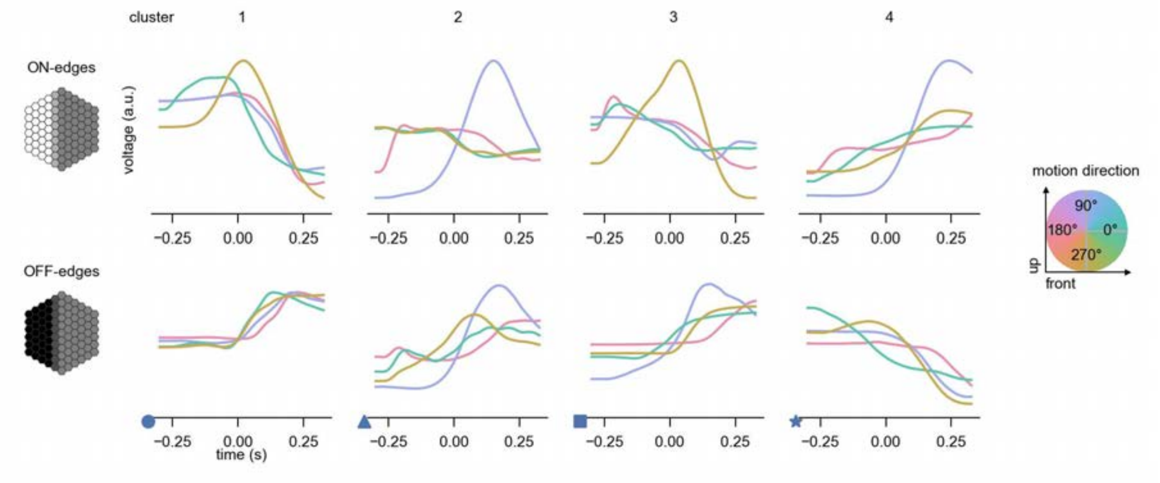

TmY4 - Figure 8: **Responses to moving edges from task-optimal models.** Responses to moving ON-edges (top row) and to moving OFF-edges (bottom row). Edges move in different directions from 0 to 360 degrees and at different speeds. Responses are from the task-optimal model in the respective cluster. Edges moving at 75.4°/s in all cardinal directions (green 0°, blue 90°, red 180°, yellow 270°) from -22.5 to 22.5° visual angle.

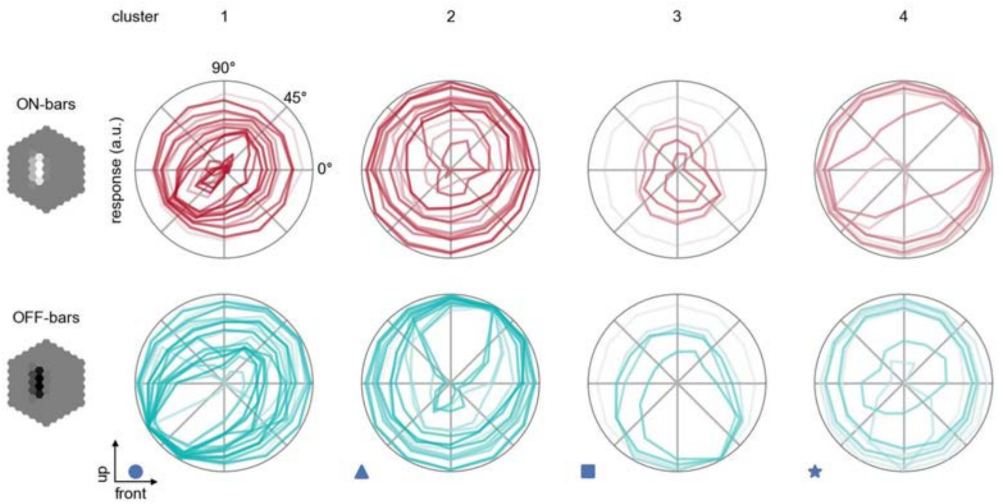

TmY4 - Figure 9: **Peak responses to moving bars.** The top row shows peak responses to moving ON-bars (red), the bottom row shows peak responses to moving OFF-bars (turqoise). The peak responses are averaged over bar-speeds. Bar-stimuli move in different directions from 0 to 360 degrees. The responses from the different models in the different clusters (columns) overlay. Responses from better task-performing models are more saturated.

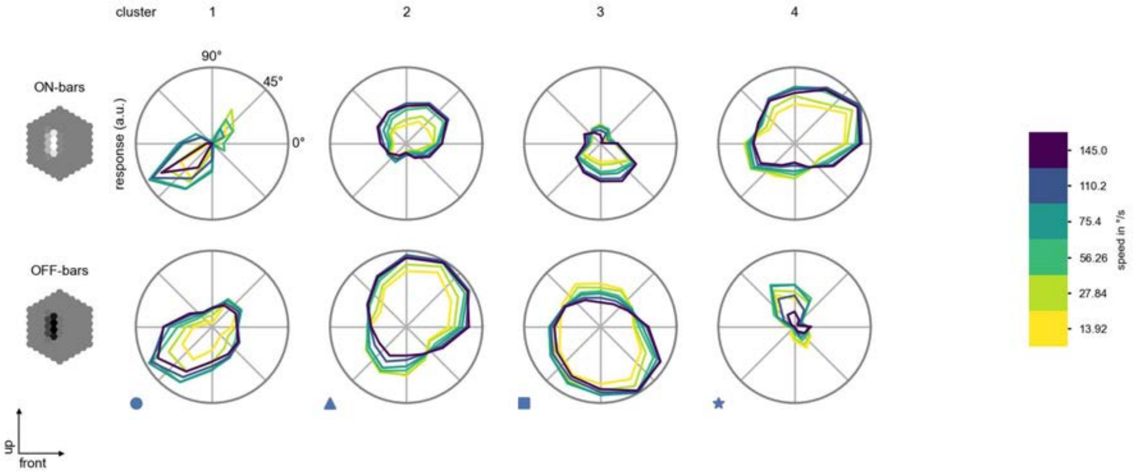

TmY4 - Figure 10: **Peak responses to moving bars from task-optimal models.** The top row shows peak responses to moving ON-bars, the bottom row shows peak responses to moving OFF-bars of varying speeds from 13.92°/s to 145°/s (yellow to dark blue). The bar-stimuli move in different directions from 0 to 360 degrees and at different speeds. Responses from the task-optimal model in the respective cluster.

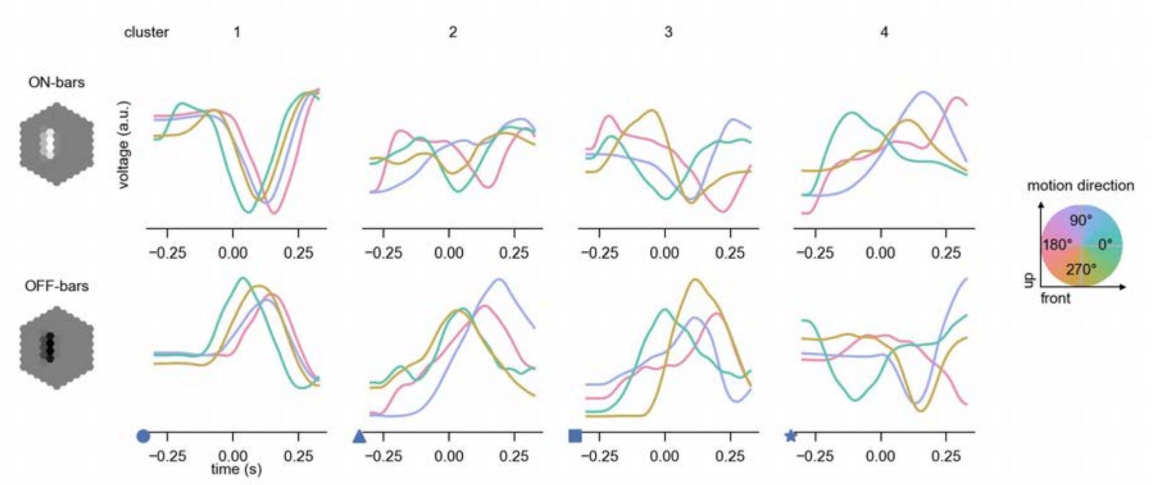

TmY4 - Figure 11: **Responses to moving bars from task-optimal models.** Responses to moving ON-bars (top row) and to moving OFF-bars (bottom row). Bars move in different directions from 0 to 360 degrees and at different speeds. Responses are from the task-optimal model in the respective cluster. Bars moving at 75.4°/s in all cardinal directions (green 0°, blue 90°, red 180°, yellow 270°) from -22.5 to 22.5° visual angle.

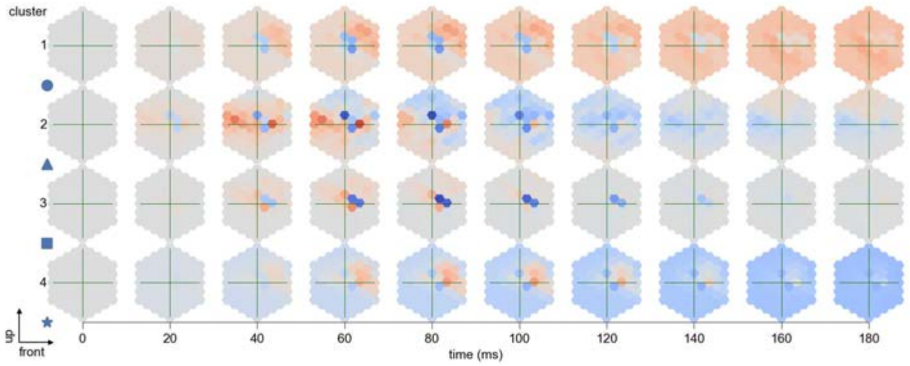

TmY4 - Figure 12: **Spatio-temporal receptive field.** Responses of the central cell to ON-impulses (5 ms) at single-ommatidium flash locations. The flash occurs at second zero. Responses from the task-optimal model of the respective cluster (rows). Red indicates depolarization, blue indicates hyperpolarization.

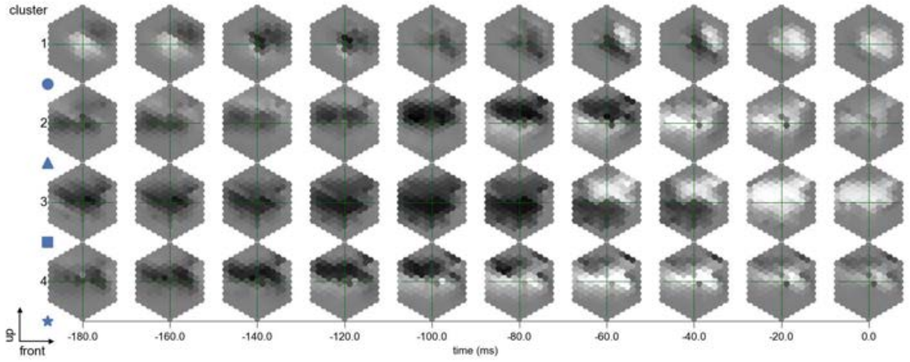

TmY4 - Figure 13: **Maximally excitatory stimuli.** Each row presents the regularized naturalistic-stimulus from the Sintel dataset that maximizes the cell type's central column response at second zero in the task-optimal model of the respective cluster (rows).

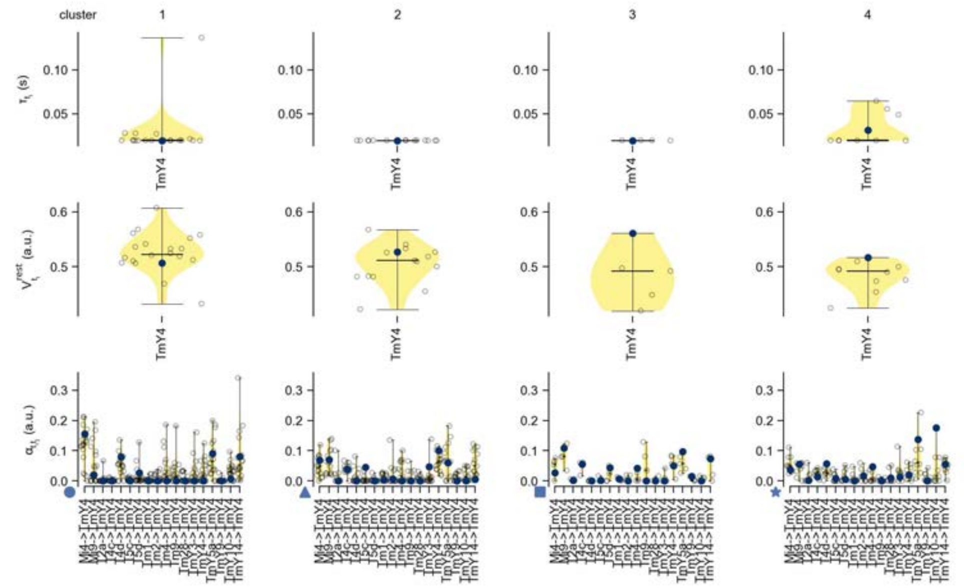

TmY4 - Figure 14: **Task-constrained parameters.** Each column shows the parameters inferred within the respective cluster. First row: learned time constants of the cell type. Second row: resting potentials of the cell type. Third row: scaling factors for the convolutional filters. The blue scatter represents the parameters from the task-optimal model within the cluster.

59 TmY5a

← Cell types

Figures

1 Anatomical receptive fields. . . . 426
2 Anatomical projective fields. . . . 427
3 Clustering of the responses to naturalistic stimuli. . . . 427
4 Responses to flashes. . . . 428
5 Cluster-average responses to single-ommatidium flashes. . . . 428
6 Peak responses to moving edges. . . . 429
7 Peak responses to moving edges from task-optimal models. . . . 429
8 Responses to moving edges from task-optimal models. . . . 430
9 Peak responses to moving bars. . . . 430
10 Peak responses to moving bars from task-optimal models. . . . 431
11 Responses to moving bars from task-optimal models. . . . 431
12 Spatio-temporal receptive field. . . . 432
13 Maximally excitatory stimuli. . . . 432
14 Task-constrained parameters. . . . 433

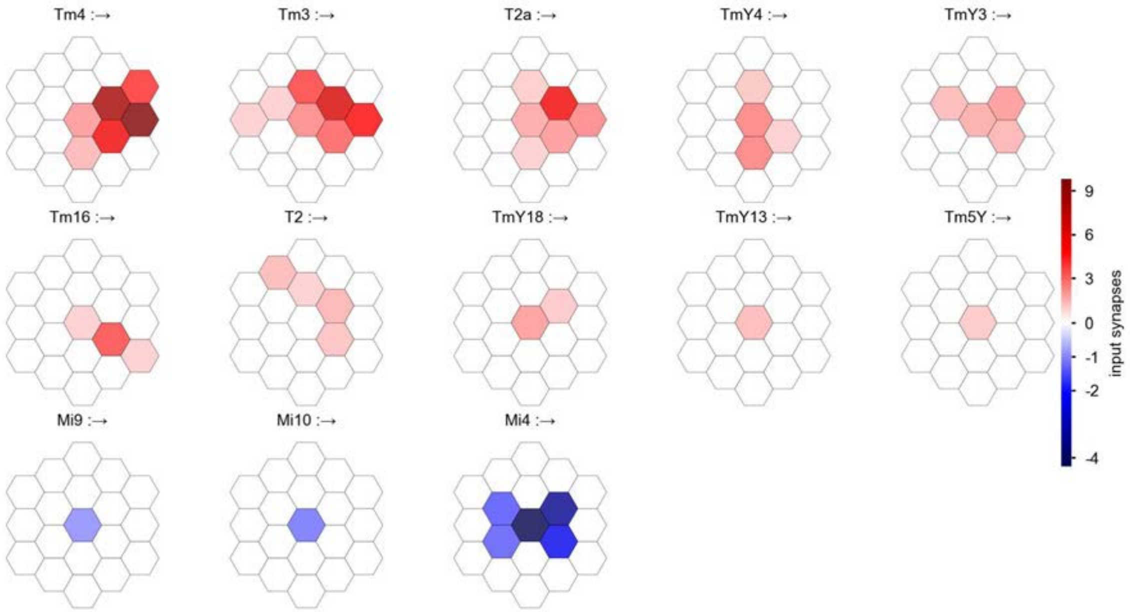

TmY5a - Figure 1: **Anatomical receptive fields.** Each colored hexagon is an input connection, with the connection strength characterized by the average number of synapses that we count from the EM reconstruction. Red indicates excitatory synapses, blue indicates inhibitory synapses from inferred signs. Filters in the order of their total number of synapses.

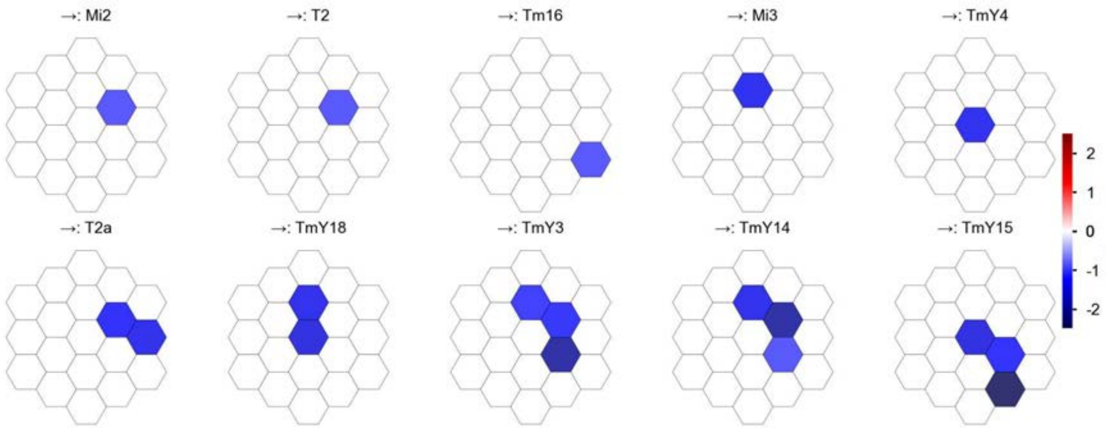

TmY5a - Figure 2: **Anatomical projective fields.** Each colored hexagon is an output connection, with the connection strength characterized by the average number of synapses that we count from the EM reconstruction. Red indicates excitatory synapses, blue indicates inhibitory synapses from inferred signs. Filters in the order of their total number of synapses.

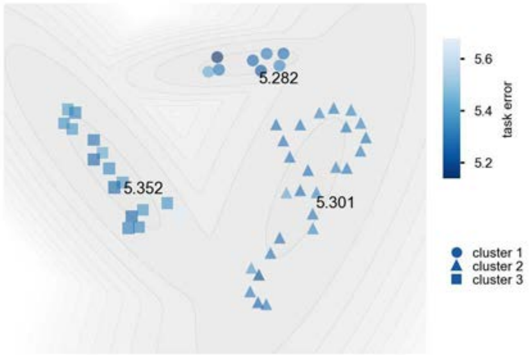

TmY5a - Figure 3: **Clustering of the responses to naturalistic stimuli.** Clustering of the 50 models based on the cell type responses to naturalistic scenes from the Sintel dataset. Scatterpoints represent individual models colored by their task error.

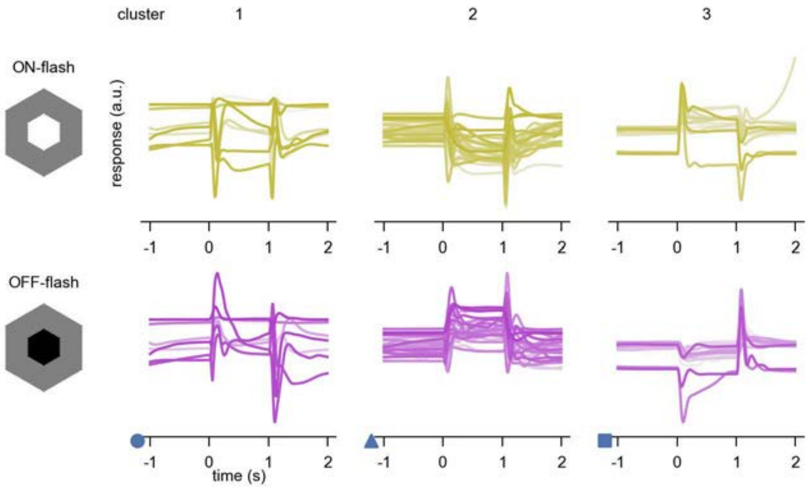

TmY5a - Figure 4: **Responses to flashes.** The top row shows responses to ON-flashes (yellow), the bottom row shows responses to OFF-flashes (magenta). The responses from the 50 different models that are separated into the different clusters (columns) overlay, with better task-performing models on top. Responses from better task-performing models are more saturated. The circular flashes (1s) cover 6 ommatidia in radius and are presented at time zero. Before and after, a grey-stimulus leads to a stationary state of the network.

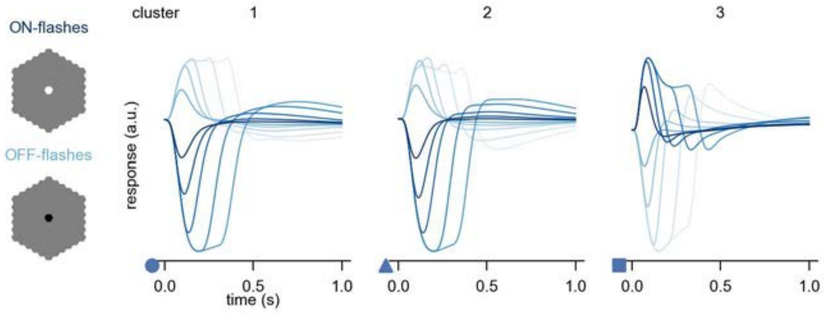

TmY5a - Figure 5: **Cluster-average responses to single-ommatidium flashes.** Responses to single-ommatidium ON-flashes (dark blue shades) and single-ommatidium OFF-flashes (light blue shades) of 20ms, 50ms, 100ms, 200ms, 300ms duration. The flashes occur at second zero.

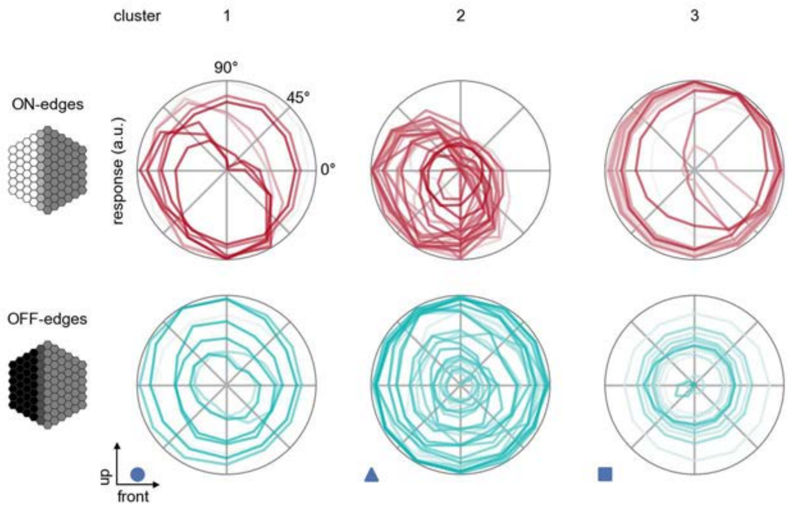

TmY5a - Figure 6: **Peak responses to moving edges.** The top row shows peak responses to moving ON-edges (red), the bottom row shows peak responses to moving OFF-edges (turqoise). The peak responses are averaged over edge-speeds. Edge-stimuli move in different directions from 0 to 360 degrees. The responses from the different models in the different clusters (columns) overlay. Responses from better task-performing models are more saturated.

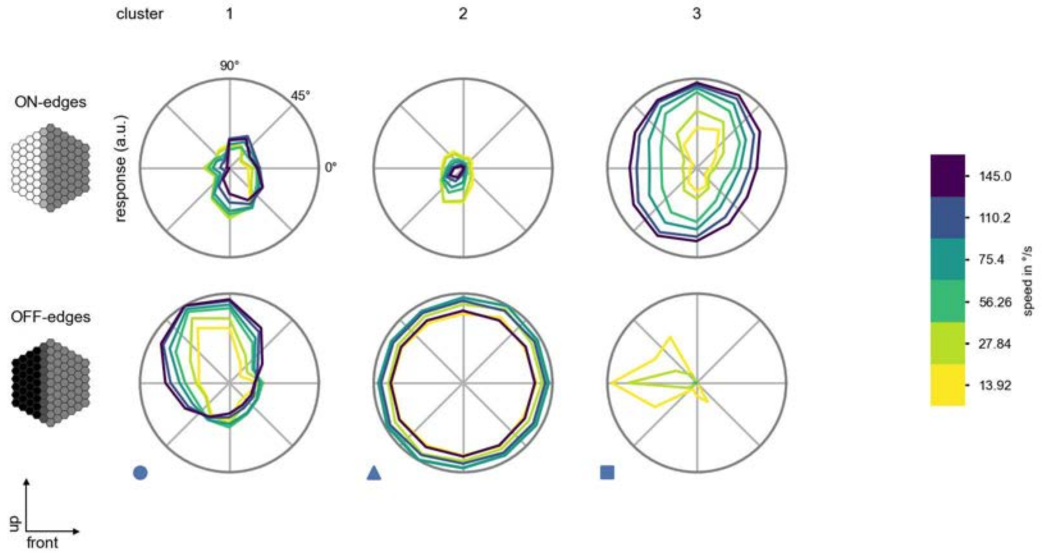

TmY5a - Figure 7: **Peak responses to moving edges from task-optimal models.** The top row shows peak responses to moving ON-edges, the bottom row shows peak responses to moving OFF-edges of varying speeds from 13.92°/s to 145°/s (yellow to dark blue). The edge-stimuli move in different directions from 0 to 360 degrees and at different speeds. Responses from the task-optimal model in the respective cluster.

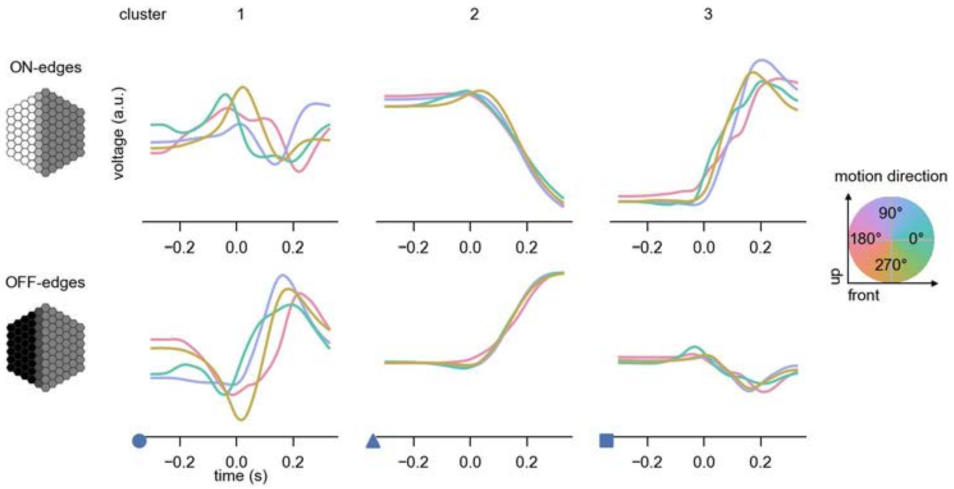

TmY5a - Figure 8: **Responses to moving edges from task-optimal models.** Responses to moving ON-edges (top row) and to moving OFF-edges (bottom row). Edges move in different directions from 0 to 360 degrees and at different speeds. Responses are from the task-optimal model in the respective cluster. Edges moving at 75.4°/s in all cardinal directions (green 0°, blue 90°, red 180°, yellow 270°) from -22.5 to 22.5° visual angle.

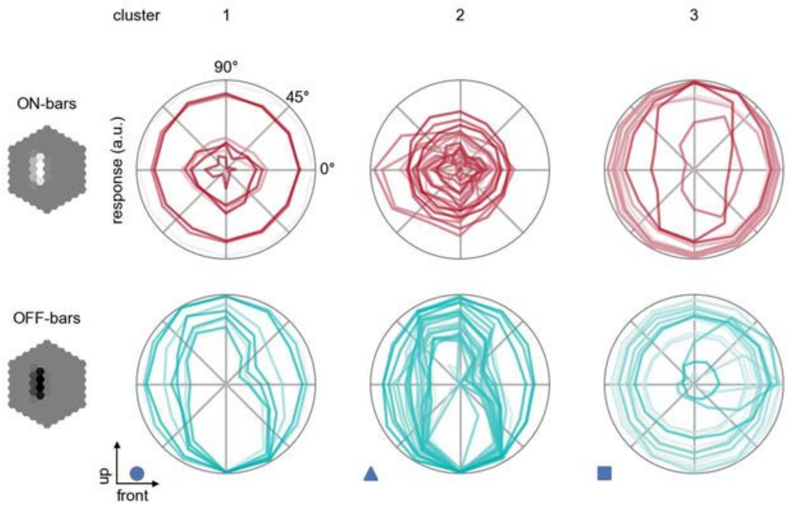

TmY5a - Figure 9: **Peak responses to moving bars.** The top row shows peak responses to moving ON-bars (red), the bottom row shows peak responses to moving OFF-bars (turqoise). The peak responses are averaged over bar-speeds. Bar-stimuli move in different directions from 0 to 360 degrees. The responses from the different models in the different clusters (columns) overlay. Responses from better task-performing models are more saturated.

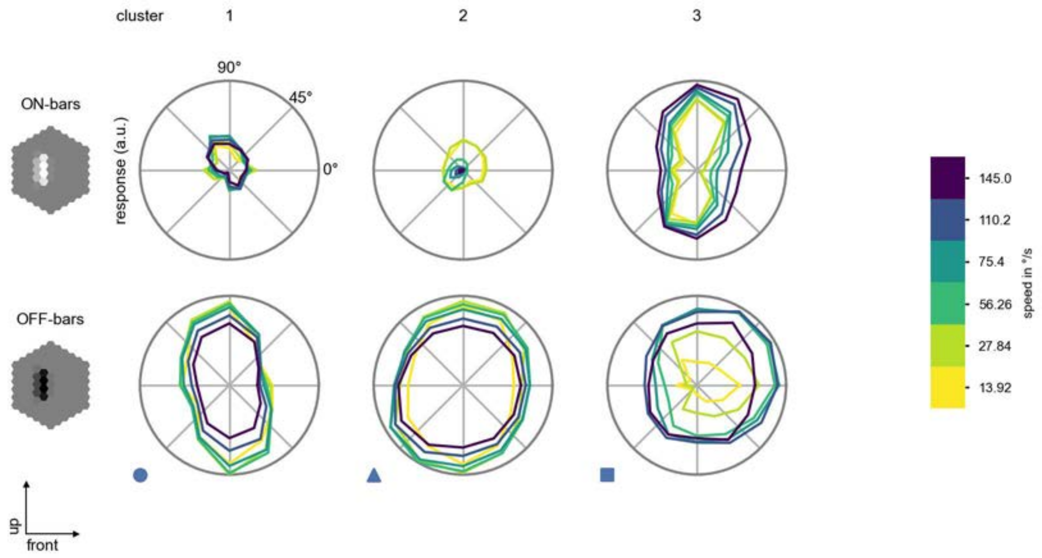

TmY5a - Figure 10: **Peak responses to moving bars from task-optimal models.** The top row shows peak responses to moving ON-bars, the bottom row shows peak responses to moving OFF-bars of varying speeds from 13.92°/s to 145°/s (yellow to dark blue). The bar-stimuli move in different directions from 0 to 360 degrees and at different speeds. Responses from the task-optimal model in the respective cluster.

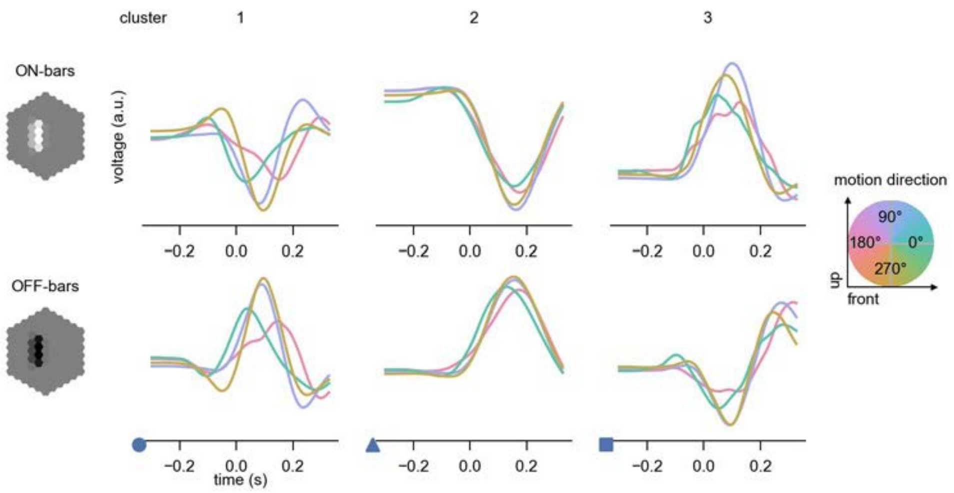

TmY5a - Figure 11: **Responses to moving bars from task-optimal models.** Responses to moving ON-bars (top row) and to moving OFF-bars (bottom row). Bars move in different directions from 0 to 360 degrees and at different speeds. Responses are from the task-optimal model in the respective cluster. Bars moving at 75.4°/s in all cardinal directions (green 0°, blue 90°, red 180°, yellow 270°) from -22.5 to 22.5° visual angle.

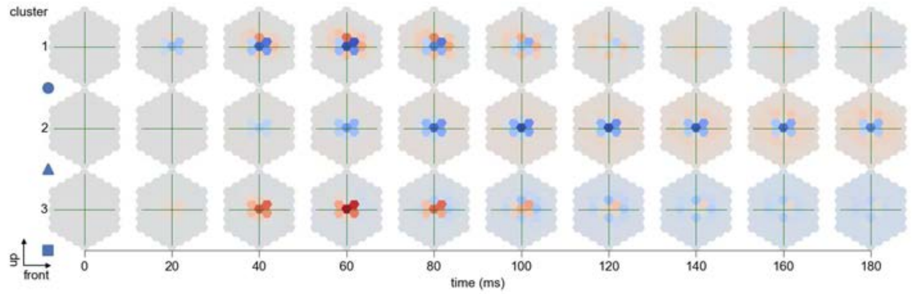

TmY5a - Figure 12: **Spatio-temporal receptive field.** Responses of the central cell to ON-impulses (5 ms) at single-ommatidium flash locations. The flash occurs at second zero. Responses from the task-optimal model of the respective cluster (rows). Red indicates depolarization, blue indicates hyperpolarization.

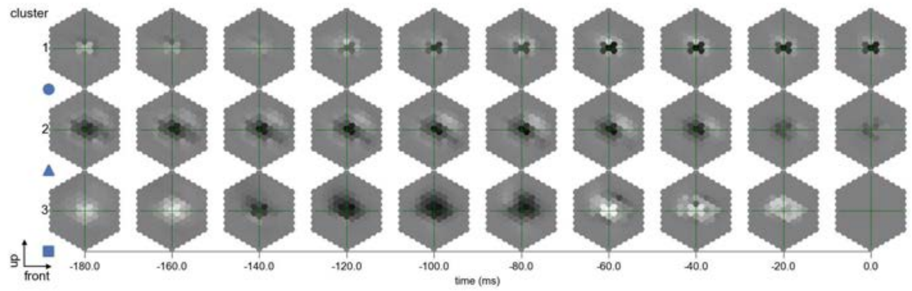

TmY5a - Figure 13: **Maximally excitatory stimuli.** Each row presents the regularized naturalistic-stimulus from the Sintel dataset that maximizes the cell type's central column response at second zero in the task-optimal model of the respective cluster (rows).

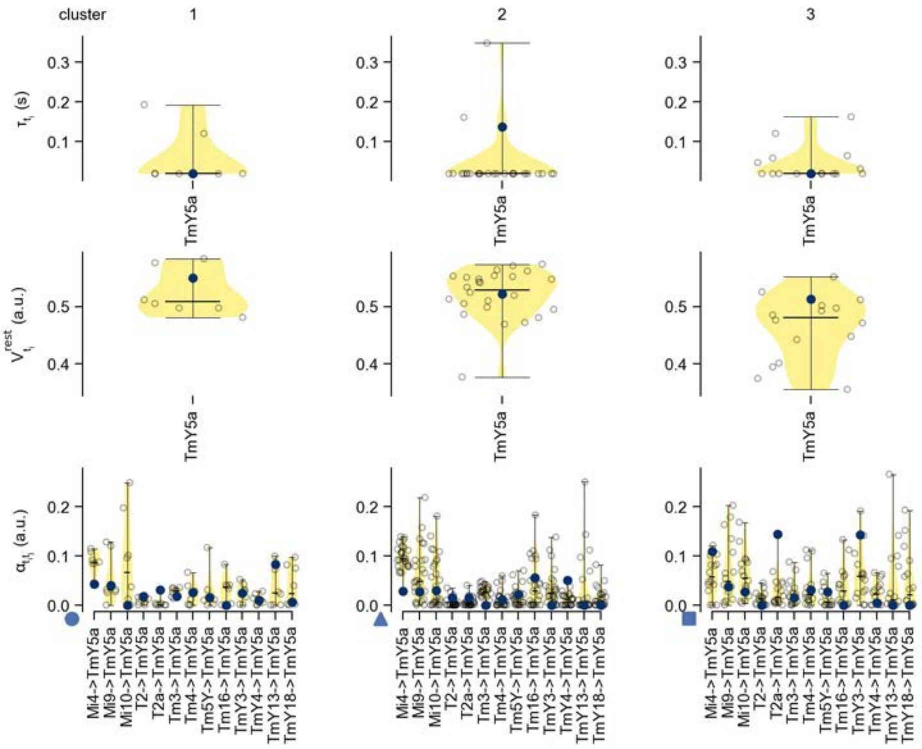

TmY5a - Figure 14: **Task-constrained parameters.** Each column shows the parameters inferred within the respective cluster. First row: learned time constants of the cell type. Second row: resting potentials of the cell type. Third row: scaling factors for the convolutional filters. The blue scatter represents the parameters from the task-optimal model within the cluster.

60 TmY9

← Cell types

Figures

1 Anatomical receptive fields. 434
2 Anatomical projective fields. 435
3 Clustering of the responses to naturalistic stimuli. 435
4 Responses to flashes. 435
5 Cluster-average responses to single-ommatidium flashes. 436
6 Peak responses to moving edges. 436
7 Peak responses to moving edges from task-optimal models. 437
8 Responses to moving edges from task-optimal models. 437
9 Peak responses to moving bars. 438
10 Peak responses to moving bars from task-optimal models. 438
11 Responses to moving bars from task-optimal models. 439
12 Spatio-temporal receptive field. 439
13 Maximally excitatory stimuli. 439
14 Task-constrained parameters. 440

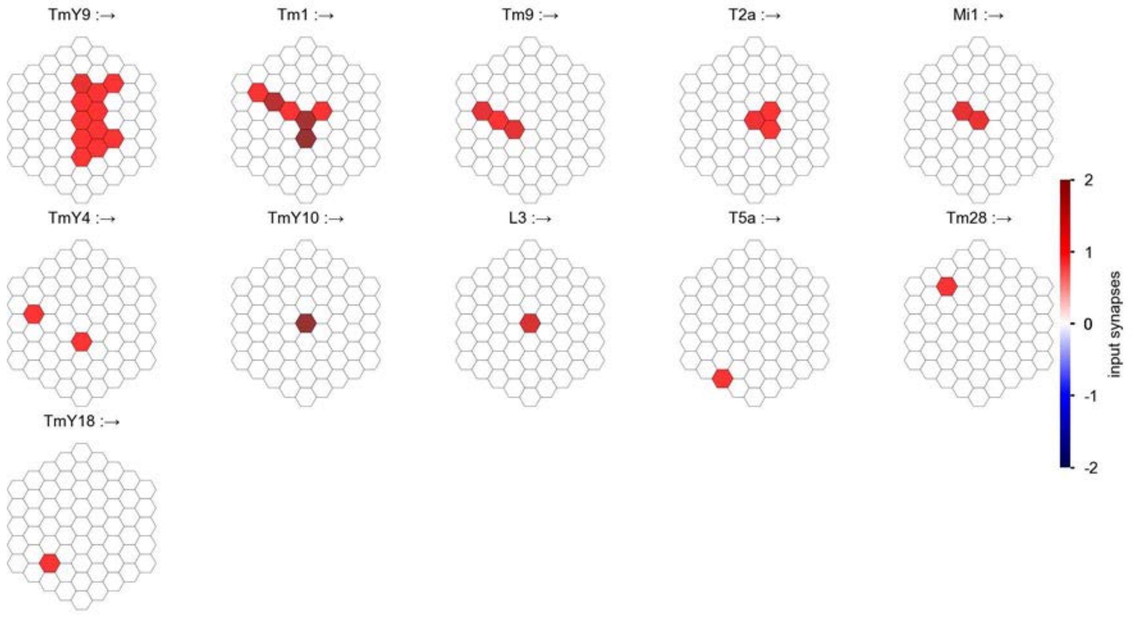

TmY9 - Figure 1: **Anatomical receptive fields.** Each colored hexagon is an input connection, with the connection strength characterized by the average number of synapses that we count from the EM reconstruction. Red indicates excitatory synapses, blue indicates inhibitory synapses from inferred signs. Filters in the order of their total number of synapses.

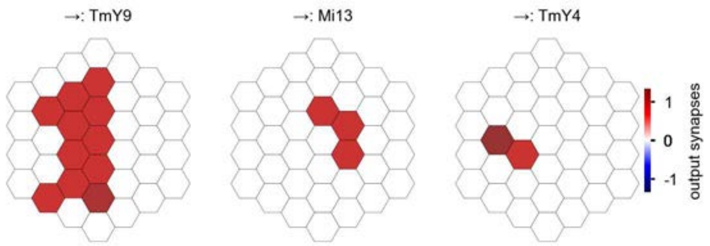

TmY9 - Figure 2: **Anatomical projective fields.** Each colored hexagon is an output connection, with the connection strength characterized by the average number of synapses that we count from the EM reconstruction. Red indicates excitatory synapses, blue indicates inhibitory synapses from inferred signs. Filters in the order of their total number of synapses.

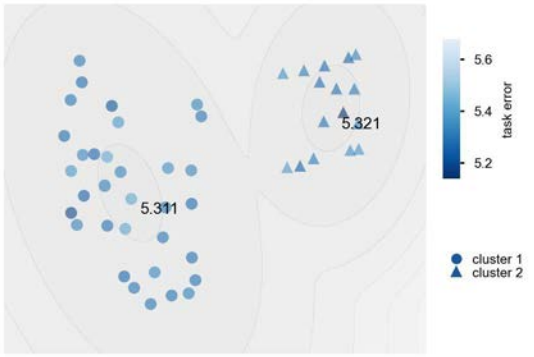

TmY9 - Figure 3: **Clustering of the responses to naturalistic stimuli.** Clustering of the 50 models based on the cell type responses to naturalistic scenes from the Sintel dataset. Scatterpoints represent individual models colored by their task error.

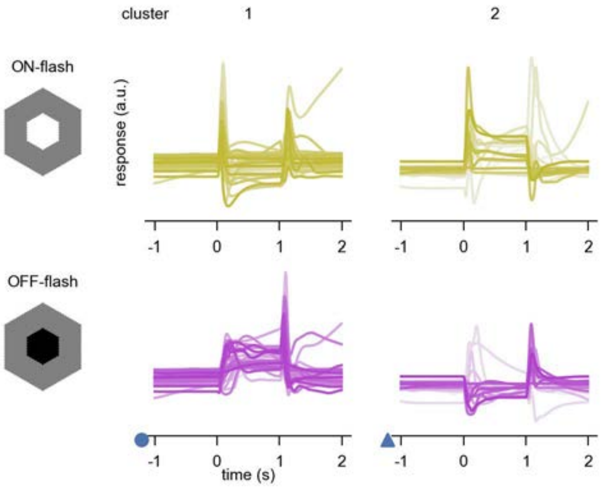

TmY9 - Figure 4: **Responses to flashes.** The top row shows responses to ON-flashes (yellow), the bottom row shows responses to OFF-flashes (magenta). The responses from the 50 different models that are separated into the different clusters (columns) overlay, with better task-performing models on top. Responses from better task-performing models are more saturated. The circular flashes (1s) cover 6 ommatidia in radius and are presented at time zero. Before and after, a grey-stimulus leads to a stationary state of the network.

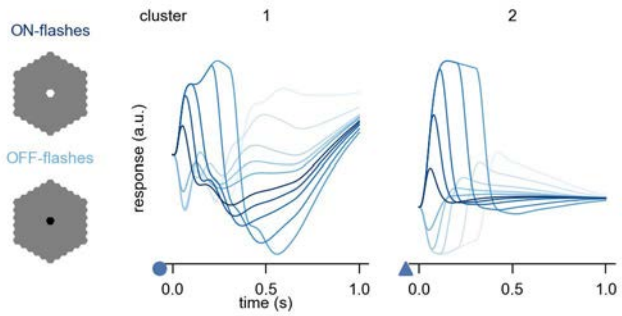

TmY9 - Figure 5: **Cluster-average responses to single-ommatidium flashes.** Responses to single-ommatidium ON-flashes (dark blue shades) and single-ommatidium OFF-flashes (light blue shades) of 20ms, 50ms, 100ms, 200ms, 300ms duration. The flashes occur at second zero.

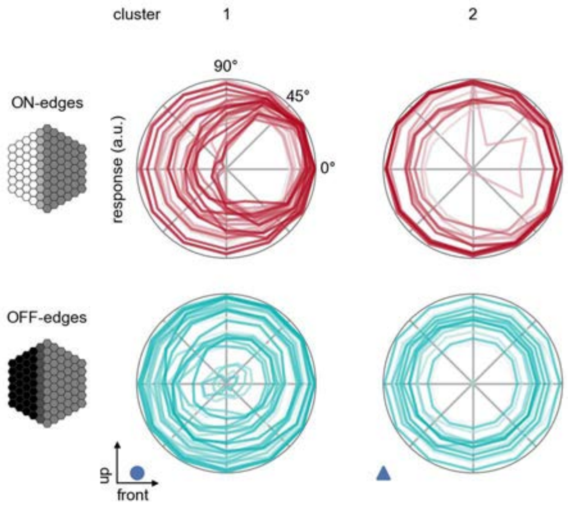

TmY9 - Figure 6: **Peak responses to moving edges.** The top row shows peak responses to moving ON-edges (red), the bottom row shows peak responses to moving OFF-edges (turqoise). The peak responses are averaged over edge-speeds. Edge-stimuli move in different directions from 0 to 360 degrees. The responses from the different models in the different clusters (columns) overlay. Responses from better task-performing models are more saturated.

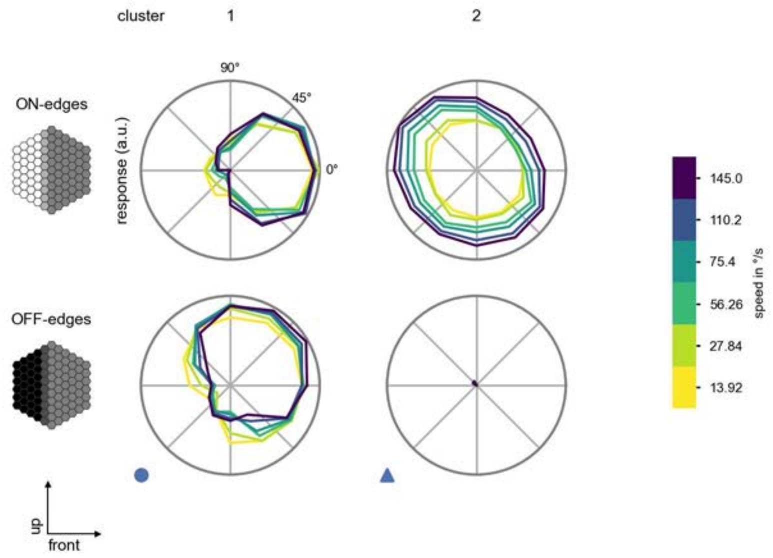

TmY9 - Figure 7: **Peak responses to moving edges from task-optimal models.** The top row shows peak responses to moving ON-edges, the bottom row shows peak responses to moving OFF-edges of varying speeds from 13.92°/s to 145°/s (yellow to dark blue). The edge-stimuli move in different directions from 0 to 360 degrees and at different speeds. Responses from the task-optimal model in the respective cluster.

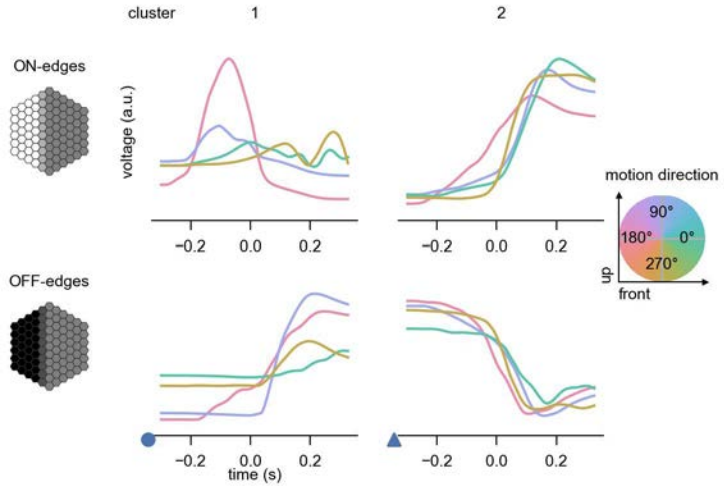

TmY9 - Figure 8: **Responses to moving edges from task-optimal models.** Responses to moving ON-edges (top row) and to moving OFF-edges (bottom row). Edges move in different directions from 0 to 360 degrees and at different speeds. Responses are from the task-optimal model in the respective cluster. Edges moving at 75.4°/s in all cardinal directions (green 0°, blue 90°, red 180°, yellow 270°) from -22.5 to 22.5° visual angle.

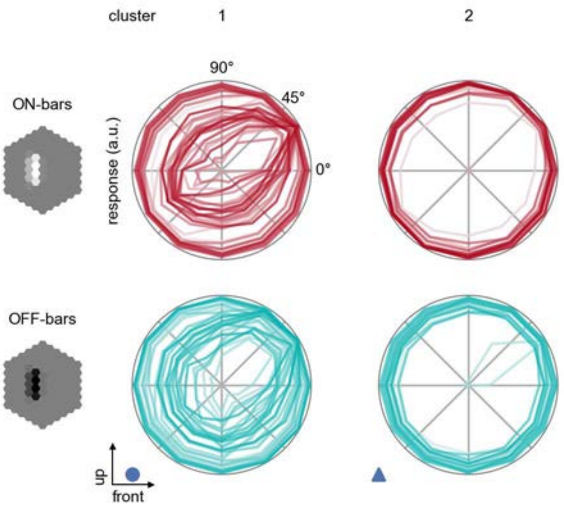

TmY9 - Figure 9: **Peak responses to moving bars.** The top row shows peak responses to moving ON-bars (red), the bottom row shows peak responses to moving OFF-bars (turqoise). The peak responses are averaged over bar-speeds. Bar-stimuli move in different directions from 0 to 360 degrees. The responses from the different models in the different clusters (columns) overlay. Responses from better task-performing models are more saturated.

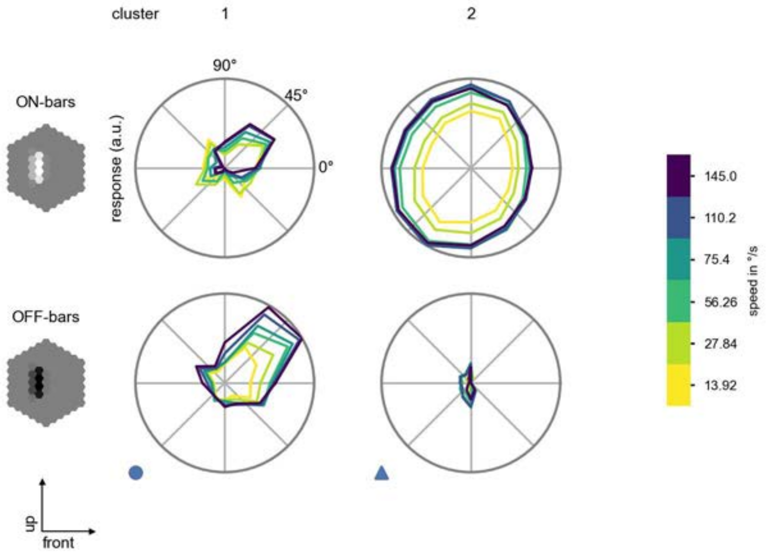

TmY9 - Figure 10: **Peak responses to moving bars from task-optimal models.** The top row shows peak responses to moving ON-bars, the bottom row shows peak responses to moving OFF-bars of varying speeds from 13.92°/s to 145°/s (yellow to dark blue). The bar-stimuli move in different directions from 0 to 360 degrees and at different speeds. Responses from the task-optimal model in the respective cluster.

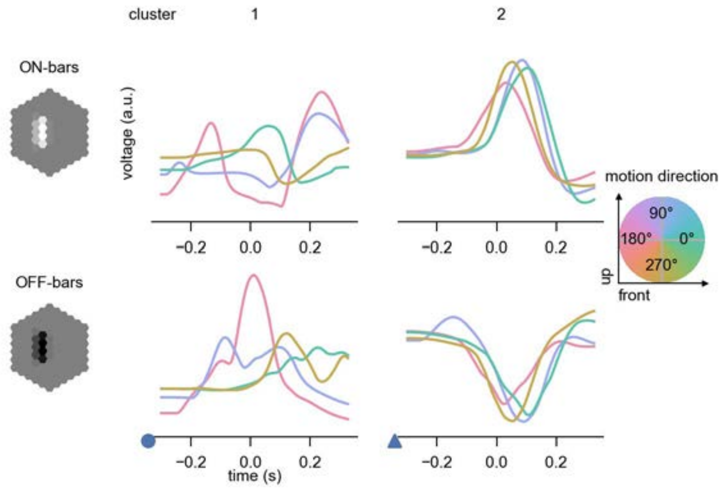

TmY9 - Figure 11: **Responses to moving bars from task-optimal models.** Responses to moving ON-bars (top row) and to moving OFF-bars (bottom row). Bars move in different directions from 0 to 360 degrees and at different speeds. Responses are from the task-optimal model in the respective cluster. Bars moving at 75.4°/s in all cardinal directions (green 0°, blue 90°, red 180°, yellow 270°) from -22.5 to 22.5° visual angle.

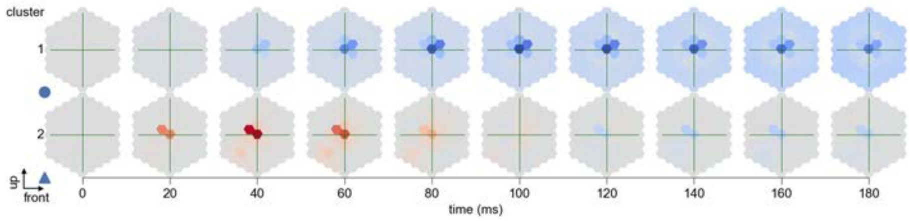

TmY9 - Figure 12: **Spatio-temporal receptive field.** Responses of the central cell to ON-impulses (5 ms) at single-ommatidium flash locations. The flash occurs at second zero. Responses from the task-optimal model of the respective cluster (rows). Red indicates depolarization, blue indicates hyperpolarization.

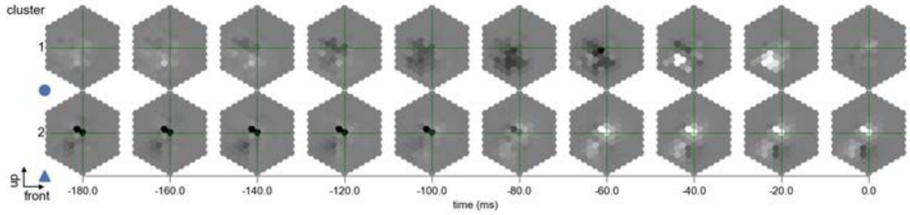

TmY9 - Figure 13: **Maximally excitatory stimuli.** Each row presents the regularized naturalistic-stimulus from the Sintel dataset that maximizes the cell type's central column response at second zero in the task-optimal model of the respective cluster (rows).

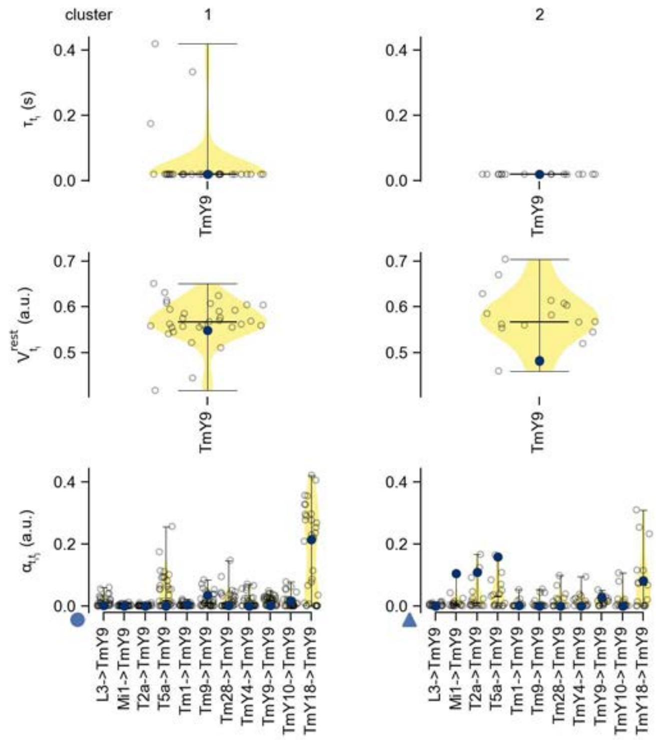

TmY9 - Figure 14: **Task-constrained parameters.** Each column shows the parameters inferred within the respective cluster. First row: learned time constants of the cell type. Second row: resting potentials of the cell type. Third row: scaling factors for the convolutional filters. The blue scatter represents the parameters from the task-optimal model within the cluster.

61 TmY10

← Cell types

Figures

1 Anatomical receptive fields. 441
2 Anatomical projective fields. 441
3 Clustering of the responses to naturalistic stimuli. 442
4 Responses to flashes. 442
5 Cluster-average responses to single-ommatidium flashes. 442
6 Peak responses to moving edges. 443
7 Peak responses to moving edges from task-optimal models. 443
8 Responses to moving edges from task-optimal models. 444
9 Peak responses to moving bars. 444
10 Peak responses to moving bars from task-optimal models. 445
11 Responses to moving bars from task-optimal models. 445
12 Spatio-temporal receptive field. 446
13 Maximally excitatory stimuli. 446
14 Task-constrained parameters. 447

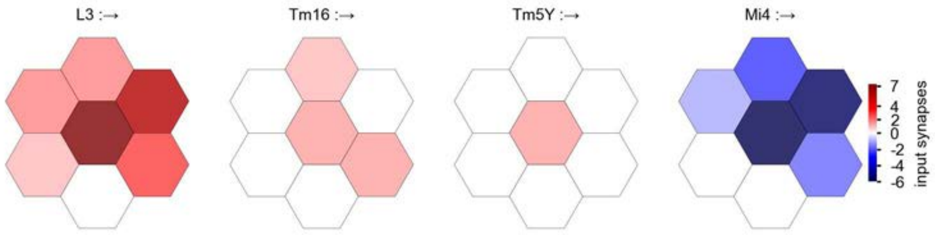

TmY10 - Figure 1: **Anatomical receptive fields.** Each colored hexagon is an input connection, with the connection strength characterized by the average number of synapses that we count from the EM reconstruction. Red indicates excitatory synapses, blue indicates inhibitory synapses from inferred signs. Filters in the order of their total number of synapses.

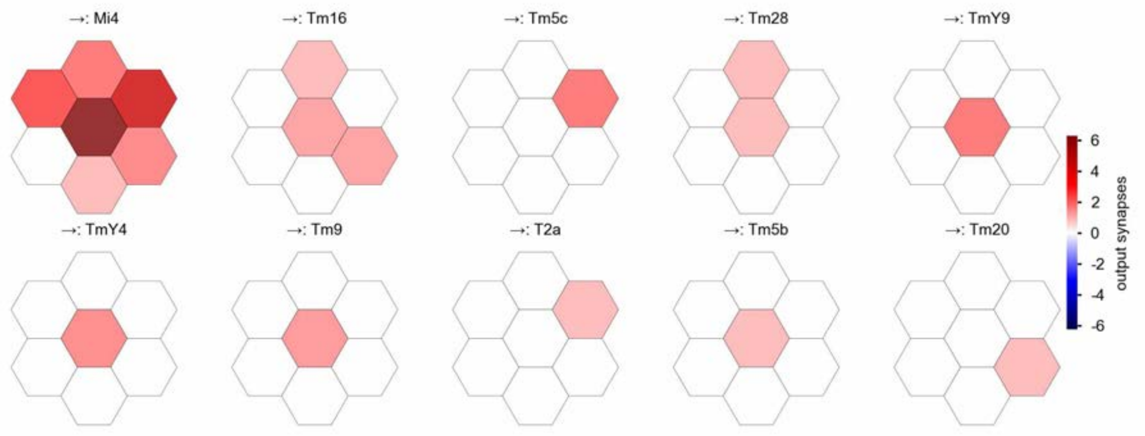

TmY10 - Figure 2: **Anatomical projective fields.** Each colored hexagon is an output connection, with the connection strength characterized by the average number of synapses that we count from the EM reconstruction. Red indicates excitatory synapses, blue indicates inhibitory synapses from inferred signs. Filters in the order of their total number of synapses.

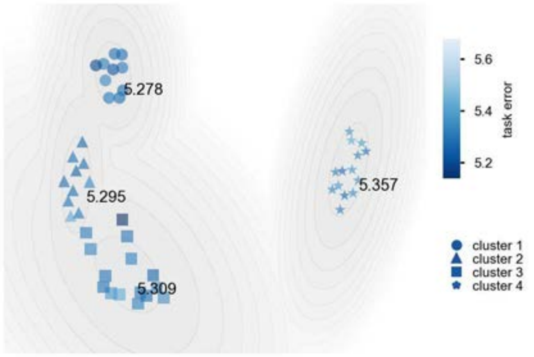

TmY10 - Figure 3: **Clustering of the responses to naturalistic stimuli.** Clustering of the 50 models based on the cell type responses to naturalistic scenes from the Sintel dataset. Scatterpoints represent individual models colored by their task error.

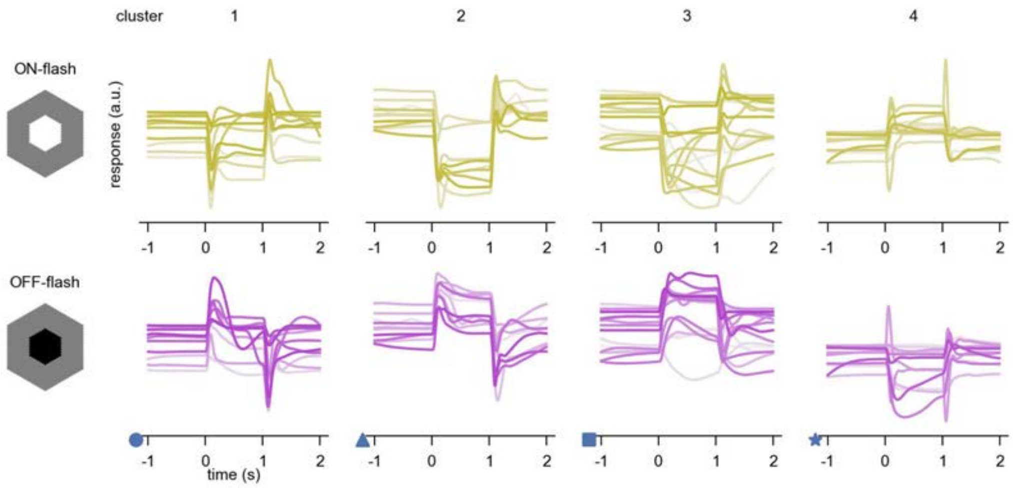

TmY10 - Figure 4: **Responses to flashes.** The top row shows responses to ON-flashes (yellow), the bottom row shows responses to OFF-flashes (magenta). The responses from the 50 different models that are separated into the different clusters (columns) overlay, with better task-performing models on top. Responses from better task-performing models are more saturated. The circular flashes (1s) cover 6 ommatidia in radius and are presented at time zero. Before and after, a grey-stimulus leads to a stationary state of the network.

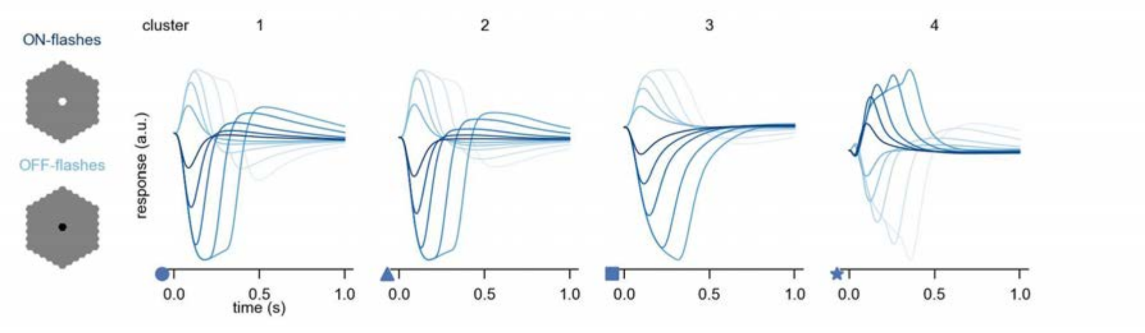

TmY10 - Figure 5: **Cluster-average responses to single-ommatidium flashes.** Responses to single-ommatidium ON-flashes (dark blue shades) and single-ommatidium OFF-flashes (light blue shades) of 20ms, 50ms, 100ms, 200ms, 300ms duration. The flashes occur at second zero.

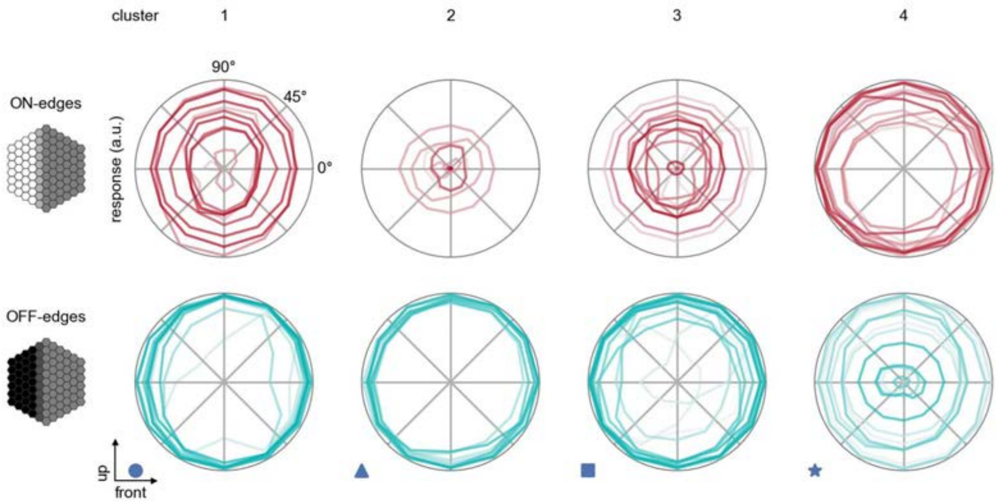

TmY10 - Figure 6: **Peak responses to moving edges.** The top row shows peak responses to moving ON-edges (red), the bottom row shows peak responses to moving OFF-edges (turqoise). The peak responses are averaged over edge-speeds. Edge-stimuli move in different directions from 0 to 360 degrees. The responses from the different models in the different clusters (columns) overlay. Responses from better task-performing models are more saturated.

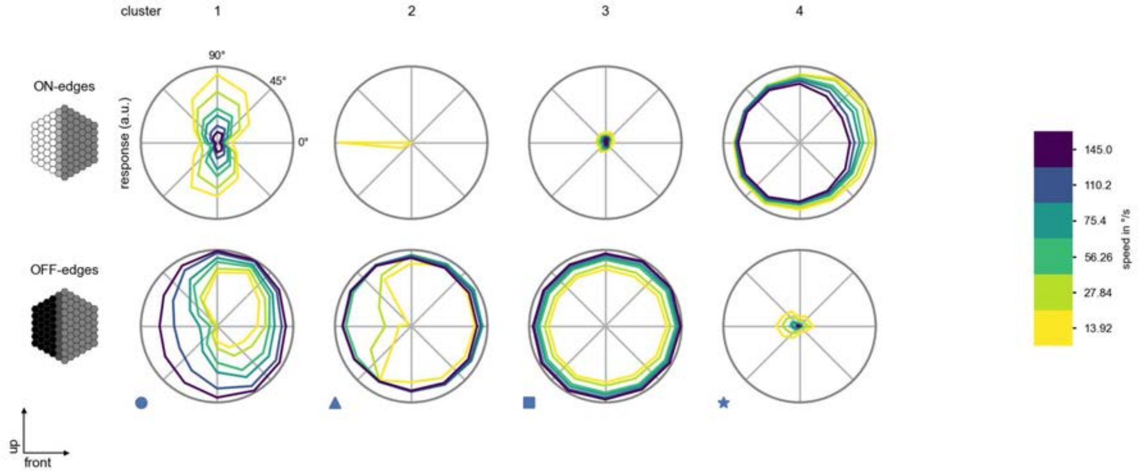

TmY10 - Figure 7: **Peak responses to moving edges from task-optimal models.** The top row shows peak responses to moving ON-edges, the bottom row shows peak responses to moving OFF-edges of varying speeds from 13.92°/s to 145°/s (yellow to dark blue). The edge-stimuli move in different directions from 0 to 360 degrees and at different speeds. Responses from the task-optimal model in the respective cluster.

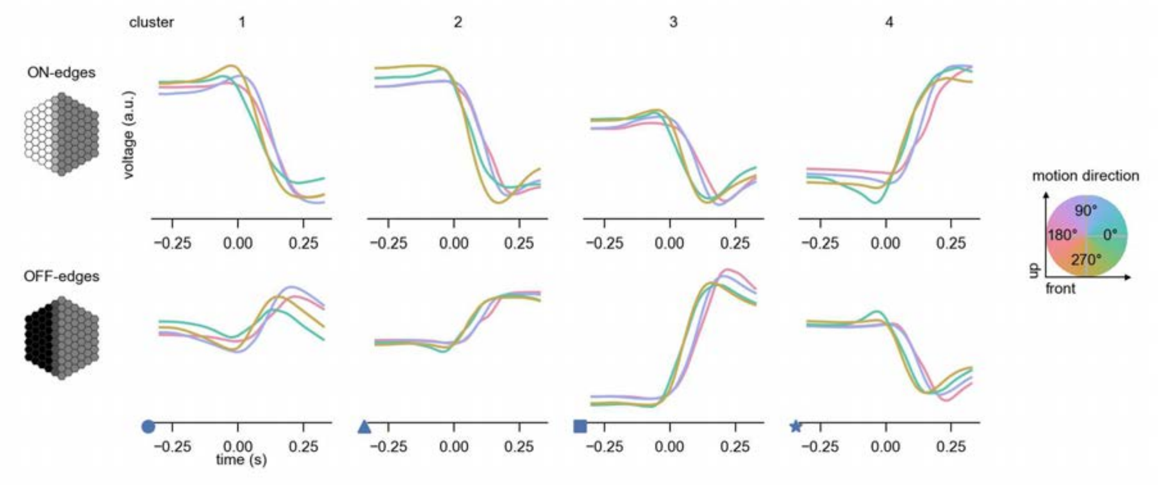

TmY10 - Figure 8: **Responses to moving edges from task-optimal models.** Responses to moving ON-edges (top row) and to moving OFF-edges (bottom row). Edges move in different directions from 0 to 360 degrees and at different speeds. Responses are from the task-optimal model in the respective cluster. Edges moving at 75.4°/s in all cardinal directions (green 0°, blue 90°, red 180°, yellow 270°) from -22.5 to 22.5° visual angle.

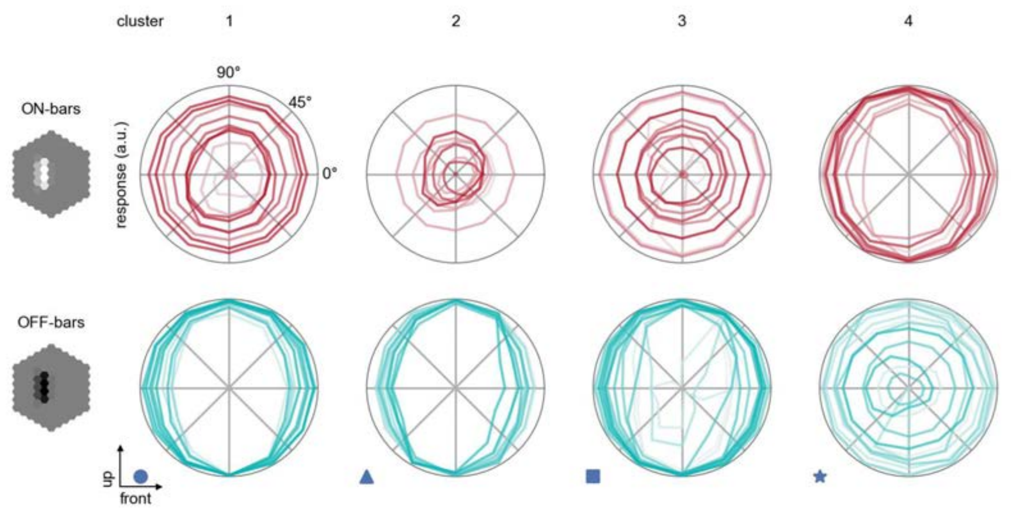

TmY10 - Figure 9: **Peak responses to moving bars.** The top row shows peak responses to moving ON-bars (red), the bottom row shows peak responses to moving OFF-bars (turqoise). The peak responses are averaged over bar-speeds. Bar-stimuli move in different directions from 0 to 360 degrees. The responses from the different models in the different clusters (columns) overlay. Responses from better task-performing models are more saturated.

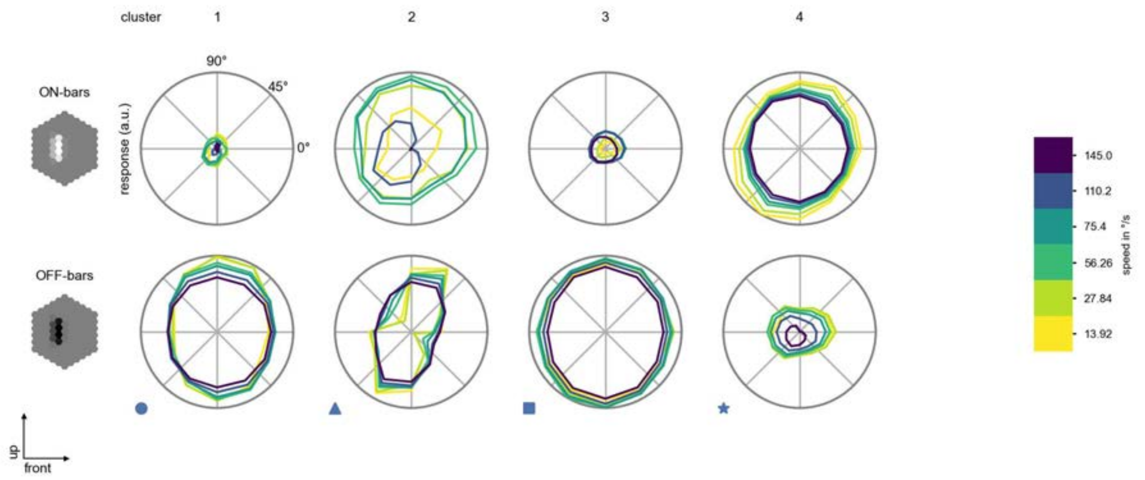

TmY10 - Figure 10: **Peak responses to moving bars from task-optimal models.** The top row shows peak responses to moving ON-bars, the bottom row shows peak responses to moving OFF-bars of varying speeds from 13.92°/s to 145°/s (yellow to dark blue). The bar-stimuli move in different directions from 0 to 360 degrees and at different speeds. Responses from the task-optimal model in the respective cluster.

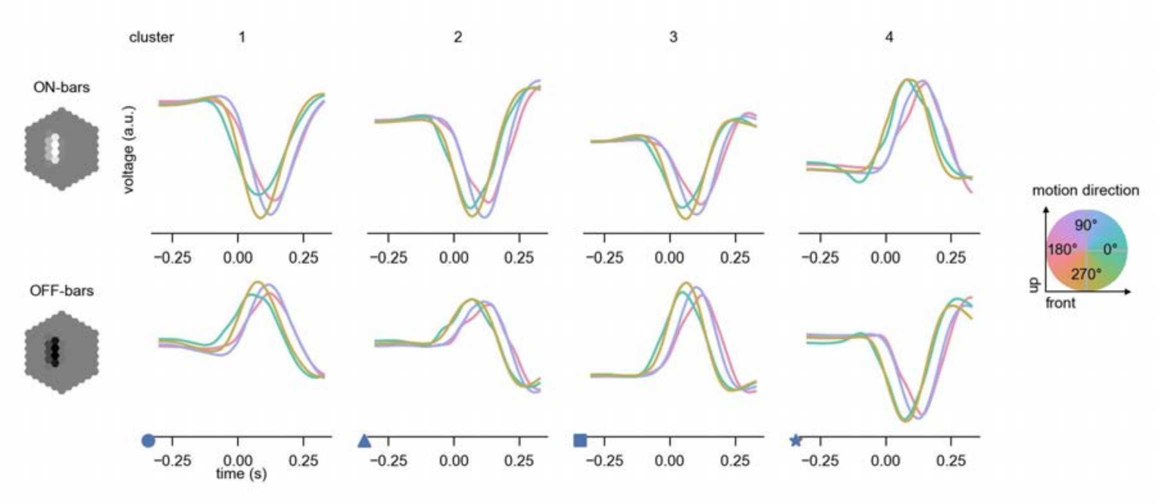

TmY10 - Figure 11: **Responses to moving bars from task-optimal models.** Responses to moving ON-bars (top row) and to moving OFF-bars (bottom row). Bars move in different directions from 0 to 360 degrees and at different speeds. Responses are from the task-optimal model in the respective cluster. Bars moving at 75.4°/s in all cardinal directions (green 0°, blue 90°, red 180°, yellow 270°) from -22.5 to 22.5° visual angle.

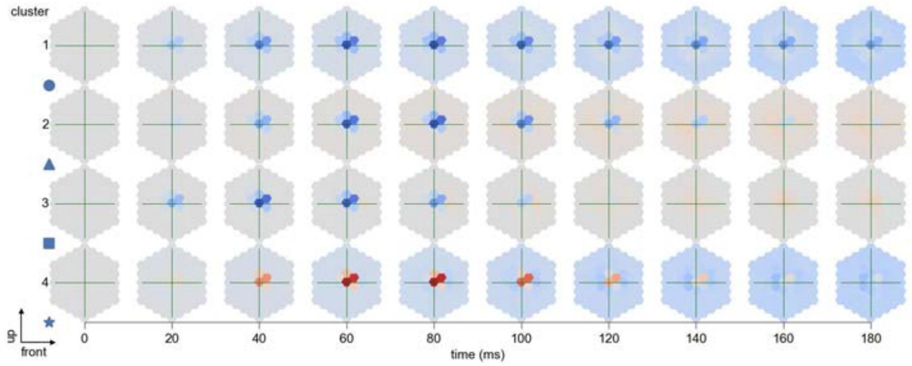

TmY10 - Figure 12: **Spatio-temporal receptive field.** Responses of the central cell to ON-impulses (5 ms) at single-ommatidium flash locations. The flash occurs at second zero. Responses from the task-optimal model of the respective cluster (rows). Red indicates depolarization, blue indicates hyperpolarization.

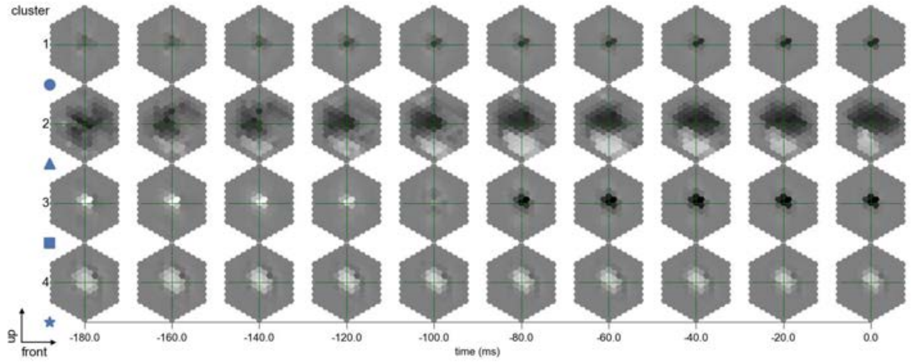

TmY10 - Figure 13: **Maximally excitatory stimuli.** Each row presents the regularized naturalistic-stimulus from the Sintel dataset that maximizes the cell type's central column response at second zero in the task-optimal model of the respective cluster (rows).

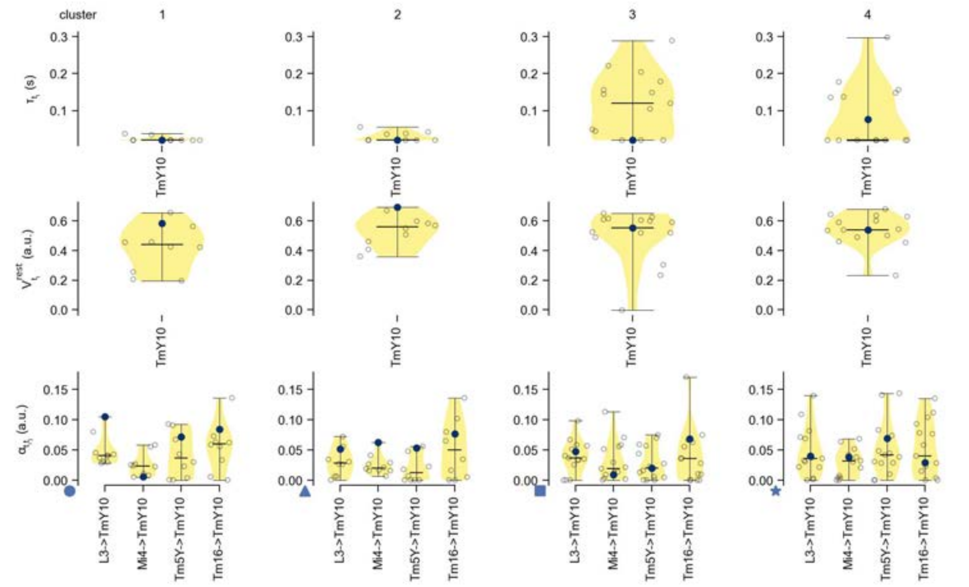

TmY10 - Figure 14: **Task-constrained parameters.** Each column shows the parameters inferred within the respective cluster. First row: learned time constants of the cell type. Second row: resting potentials of the cell type. Third row: scaling factors for the convolutional filters. The blue scatter represents the parameters from the task-optimal model within the cluster.

62 TmY13

← Cell types

Figures

1 Anatomical receptive fields. . . . 448
2 Anatomical projective fields. . . . 448
3 Clustering of the responses to naturalistic stimuli. . . . 449
4 Responses to flashes. . . . 449
5 Cluster-average responses to single-ommatidium flashes. . . . 449
6 Peak responses to moving edges. . . . 450
7 Peak responses to moving edges from task-optimal models. . . . 450
8 Responses to moving edges from task-optimal models. . . . 451
9 Peak responses to moving bars. . . . 451
10 Peak responses to moving bars from task-optimal models. . . . 452
11 Responses to moving bars from task-optimal models. . . . 452
12 Spatio-temporal receptive field. . . . 453
13 Maximally excitatory stimuli. . . . 453
14 Task-constrained parameters. . . . 454

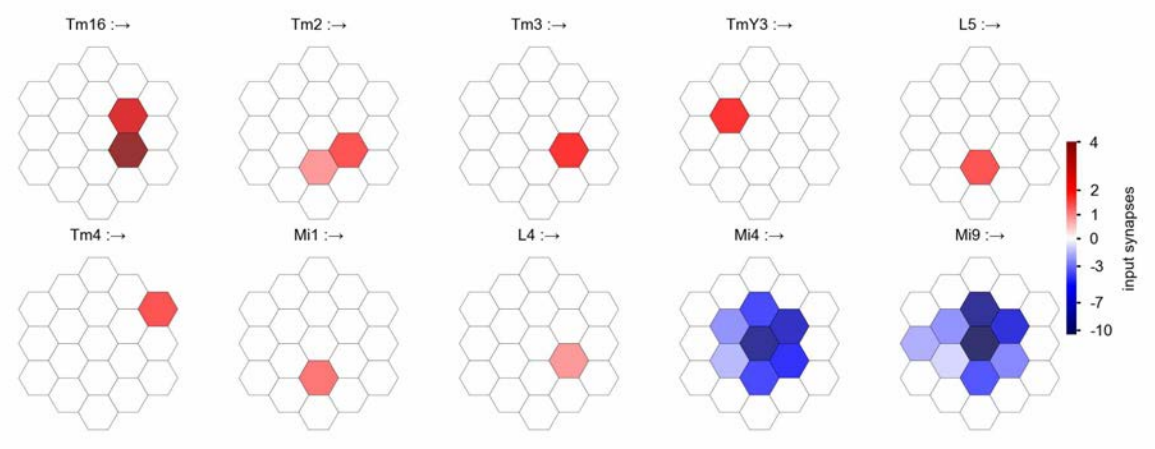

TmY13 - Figure 1: **Anatomical receptive fields.** Each colored hexagon is an input connection, with the connection strength characterized by the average number of synapses that we count from the EM reconstruction. Red indicates excitatory synapses, blue indicates inhibitory synapses from inferred signs. Filters in the order of their total number of synapses.

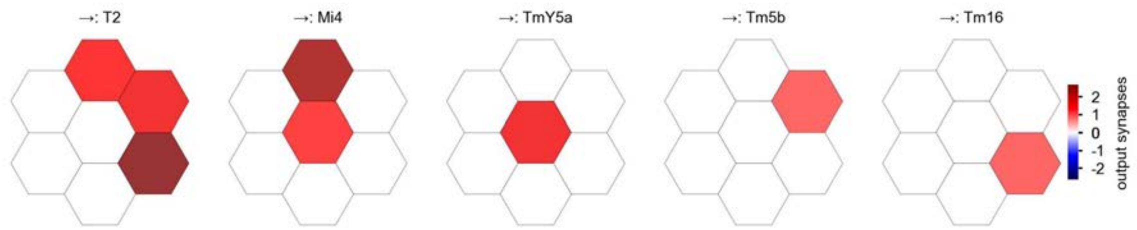

TmY13 - Figure 2: **Anatomical projective fields.** Each colored hexagon is an output connection, with the connection strength characterized by the average number of synapses that we count from the EM reconstruction. Red indicates excitatory synapses, blue indicates inhibitory synapses from inferred signs. Filters in the order of their total number of synapses.

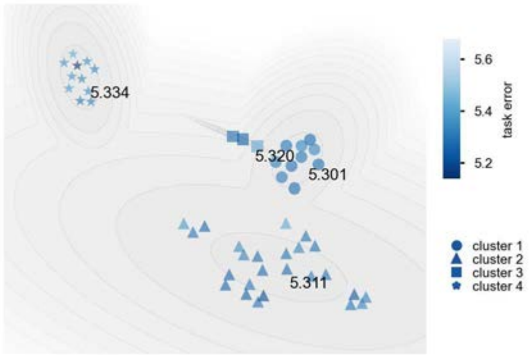

TmY13 - Figure 3: **Clustering of the responses to naturalistic stimuli.** Clustering of the 50 models based on the cell type responses to naturalistic scenes from the Sintel dataset. Scatterpoints represent individual models colored by their task error.

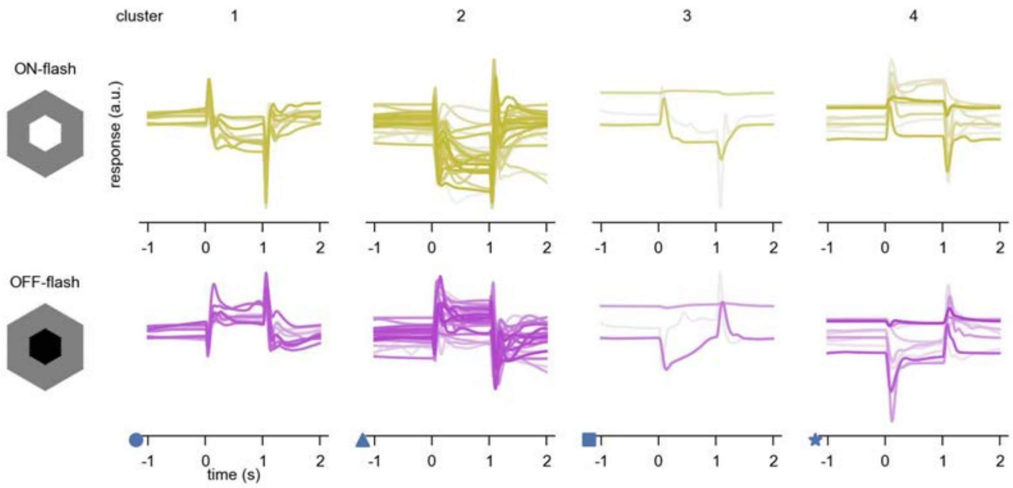

TmY13 - Figure 4: **Responses to flashes.** The top row shows responses to ON-flashes (yellow), the bottom row shows responses to OFF-flashes (magenta). The responses from the 50 different models that are separated into the different clusters (columns) overlay, with better task-performing models on top. Responses from better task-performing models are more saturated. The circular flashes (1s) cover 6 ommatidia in radius and are presented at time zero. Before and after, a grey-stimulus leads to a stationary state of the network.

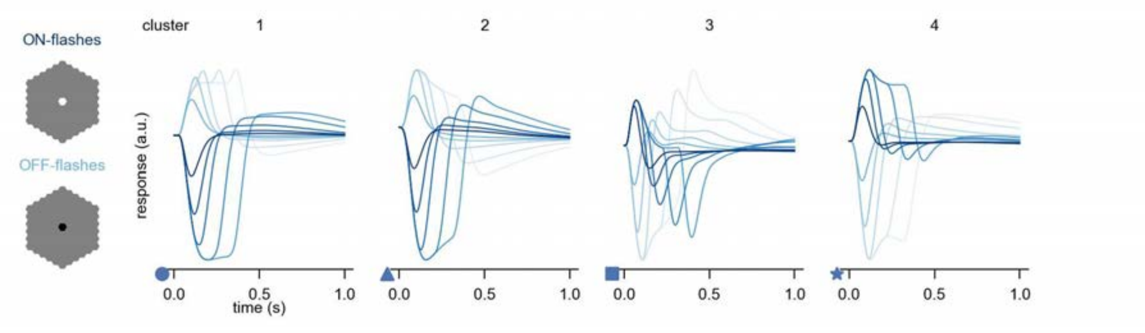

TmY13 - Figure 5: **Cluster-average responses to single-ommatidium flashes.** Responses to single-ommatidium ON-flashes (dark blue shades) and single-ommatidium OFF-flashes (light blue shades) of 20ms, 50ms, 100ms, 200ms, 300ms duration. The flashes occur at second zero.

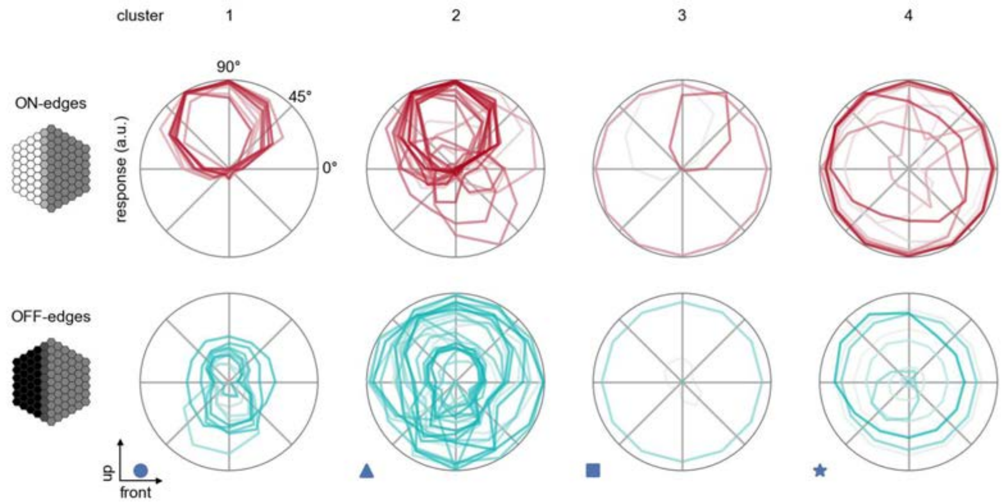

TmY13 - Figure 6: **Peak responses to moving edges.** The top row shows peak responses to moving ON-edges (red), the bottom row shows peak responses to moving OFF-edges (turqoise). The peak responses are averaged over edge-speeds. Edge-stimuli move in different directions from 0 to 360 degrees. The responses from the different models in the different clusters (columns) overlay. Responses from better task-performing models are more saturated.

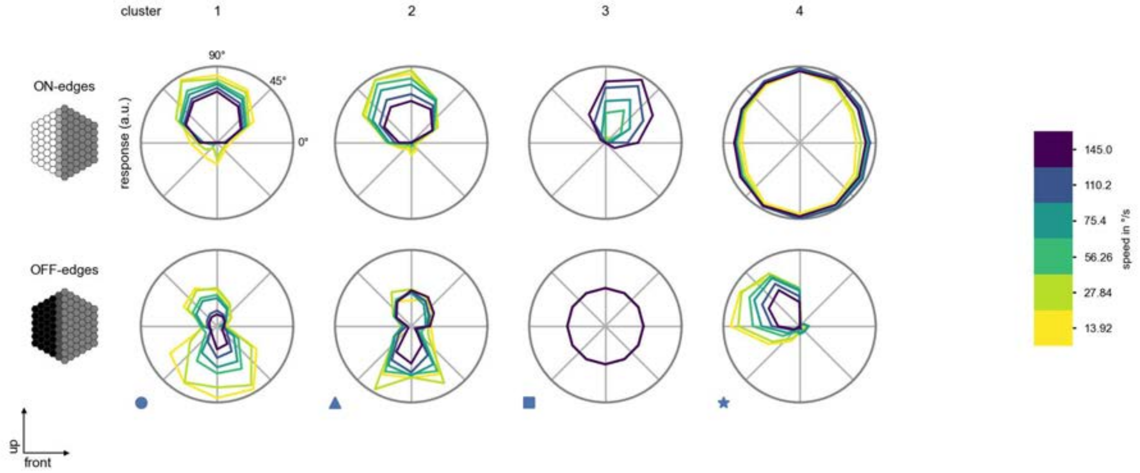

TmY13 - Figure 7: **Peak responses to moving edges from task-optimal models.** The top row shows peak responses to moving ON-edges, the bottom row shows peak responses to moving OFF-edges of varying speeds from 13.92°/s to 145°/s (yellow to dark blue). The edge-stimuli move in different directions from 0 to 360 degrees and at different speeds. Responses from the task-optimal model in the respective cluster.

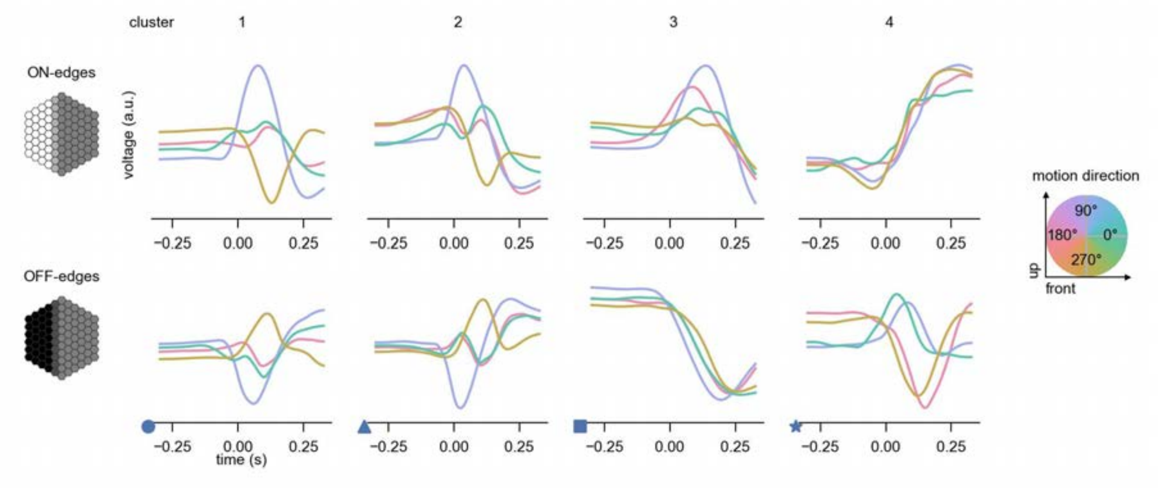

TmY13 - Figure 8: **Responses to moving edges from task-optimal models.** Responses to moving ON-edges (top row) and to moving OFF-edges (bottom row). Edges move in different directions from 0 to 360 degrees and at different speeds. Responses are from the task-optimal model in the respective cluster. Edges moving at 75.4°/s in all cardinal directions (green 0°, blue 90°, red 180°, yellow 270°) from -22.5 to 22.5° visual angle.

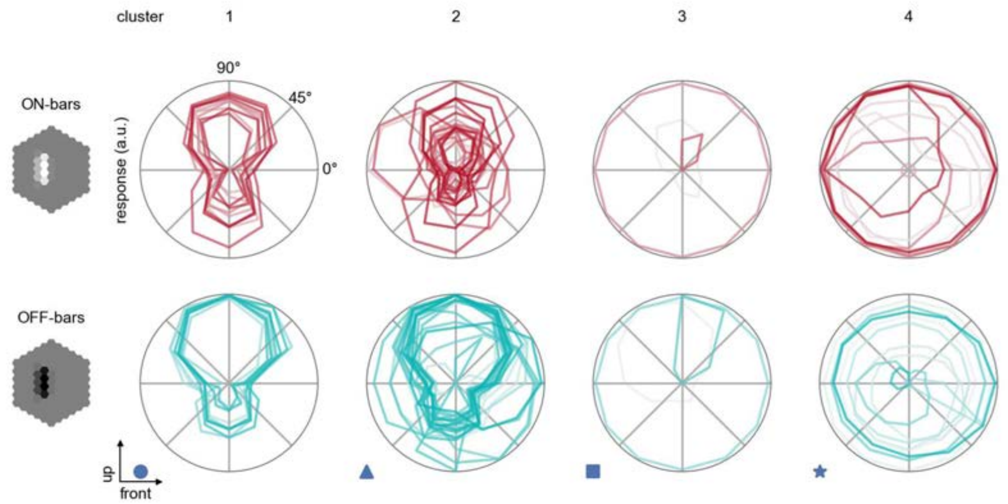

TmY13 - Figure 9: **Peak responses to moving bars.** The top row shows peak responses to moving ON-bars (red), the bottom row shows peak responses to moving OFF-bars (turqoise). The peak responses are averaged over bar-speeds. Bar-stimuli move in different directions from 0 to 360 degrees. The responses from the different models in the different clusters (columns) overlay. Responses from better task-performing models are more saturated.

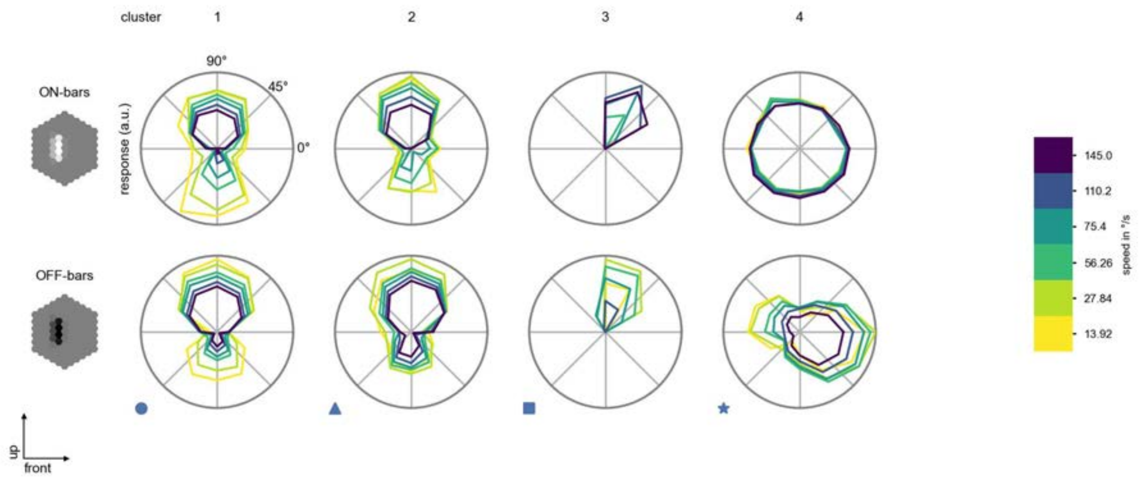

TmY13 - Figure 10: **Peak responses to moving bars from task-optimal models.** The top row shows peak responses to moving ON-bars, the bottom row shows peak responses to moving OFF-bars of varying speeds from 13.92°/s to 145°/s (yellow to dark blue). The bar-stimuli move in different directions from 0 to 360 degrees and at different speeds. Responses from the task-optimal model in the respective cluster.

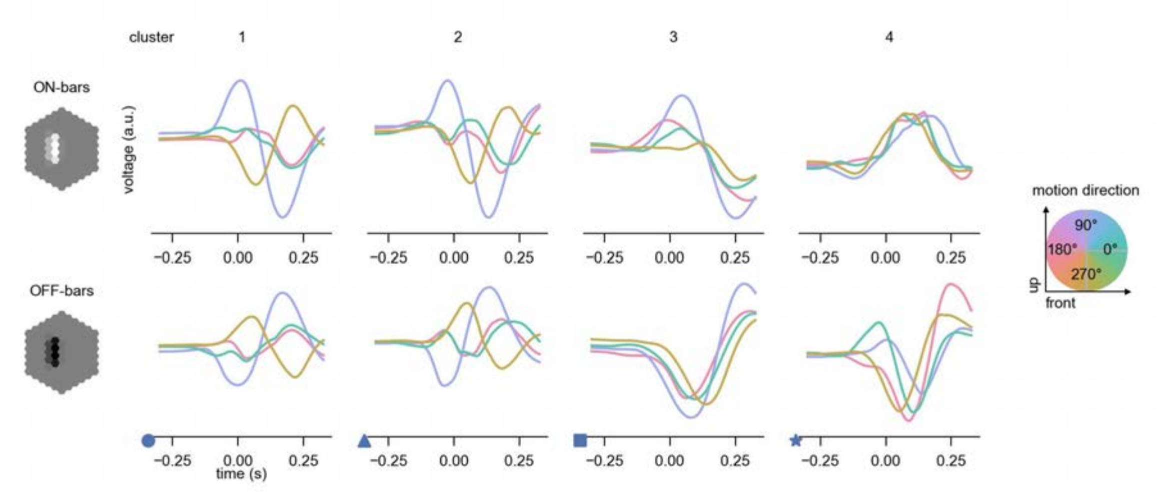

TmY13 - Figure 11: **Responses to moving bars from task-optimal models.** Responses to moving ON-bars (top row) and to moving OFF-bars (bottom row). Bars move in different directions from 0 to 360 degrees and at different speeds. Responses are from the task-optimal model in the respective cluster. Bars moving at 75.4°/s in all cardinal directions (green 0°, blue 90°, red 180°, yellow 270°) from -22.5 to 22.5° visual angle.

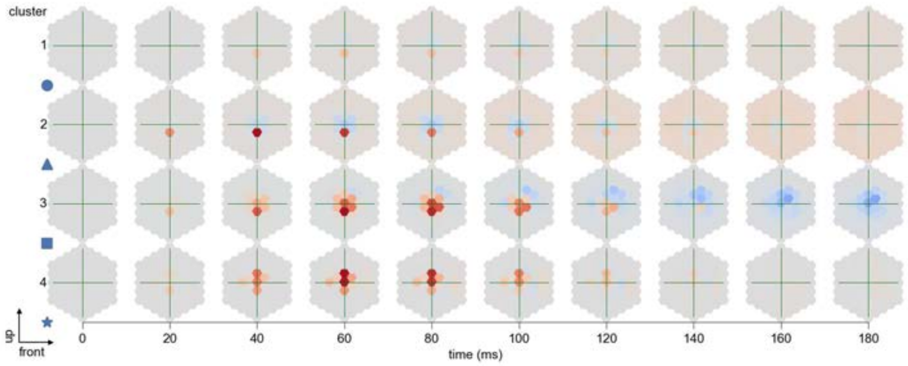

TmY13 - Figure 12: **Spatio-temporal receptive field.** Responses of the central cell to ON-impulses (5 ms) at single-ommatidium flash locations. The flash occurs at second zero. Responses from the task-optimal model of the respective cluster (rows). Red indicates depolarization, blue indicates hyperpolarization.

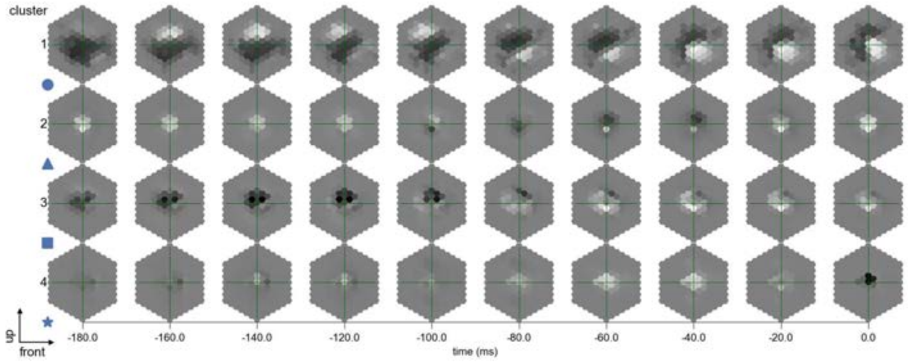

TmY13 - Figure 13: **Maximally excitatory stimuli.** Each row presents the regularized naturalistic-stimulus from the Sintel dataset that maximizes the cell type's central column response at second zero in the task-optimal model of the respective cluster (rows).

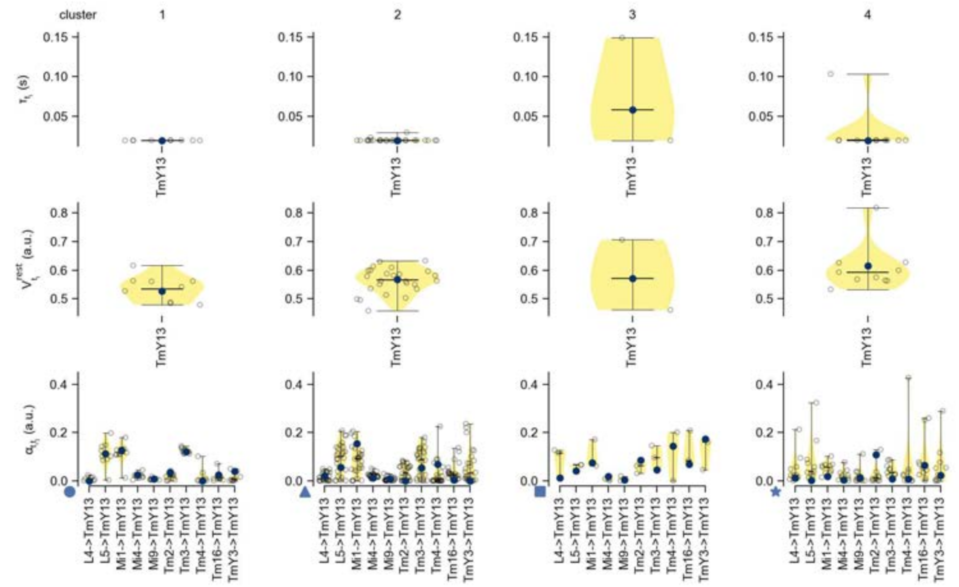

TmY13 - Figure 14: **Task-constrained parameters.** Each column shows the parameters inferred within the respective cluster. First row: learned time constants of the cell type. Second row: resting potentials of the cell type. Third row: scaling factors for the convolutional filters. The blue scatter represents the parameters from the task-optimal model within the cluster.

63 TmY14

← Cell types

Figures

1 Anatomical receptive fields. 455
2 Anatomical projective fields. 456
3 Clustering of the responses to naturalistic stimuli. 456
4 Responses to flashes. 457
5 Cluster-average responses to single-ommatidium flashes. 457
6 Peak responses to moving edges. 458
7 Peak responses to moving edges from task-optimal models. 458
8 Responses to moving edges from task-optimal models. 459
9 Peak responses to moving bars. 459
10 Peak responses to moving bars from task-optimal models. 460
11 Responses to moving bars from task-optimal models. 460
12 Spatio-temporal receptive field. 461
13 Maximally excitatory stimuli. 461
14 Task-constrained parameters. 462

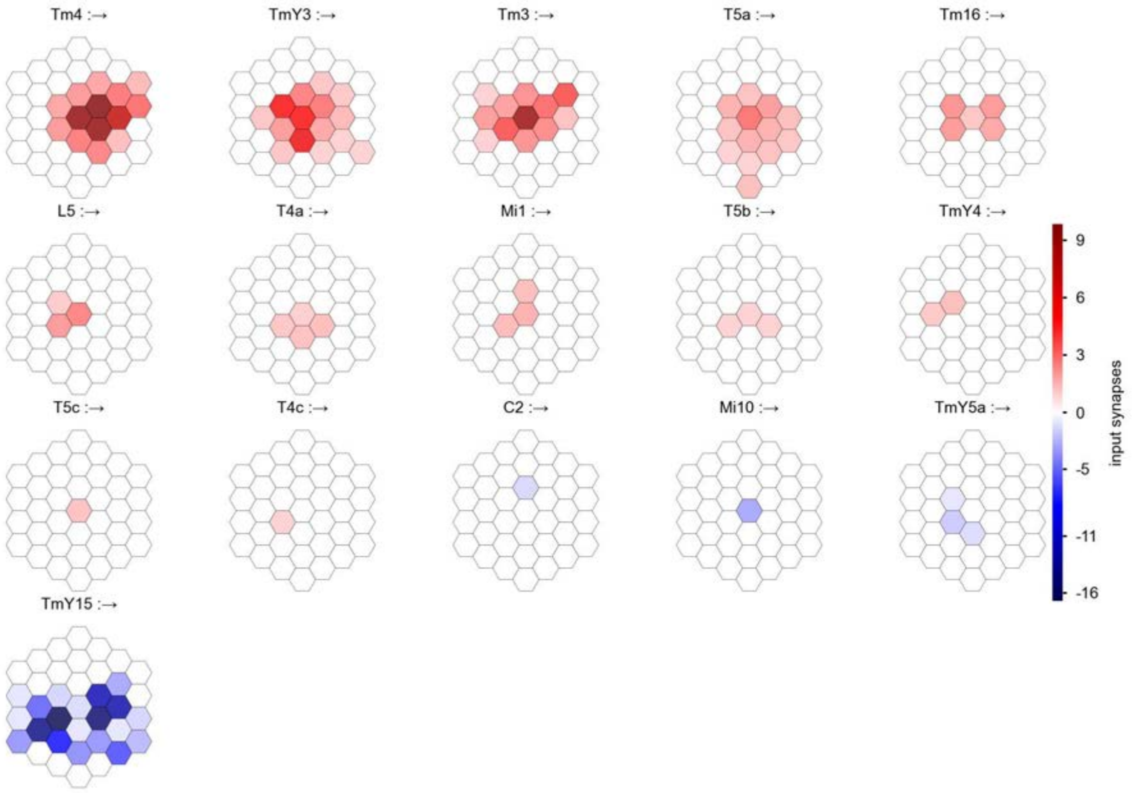

TmY14 - Figure 1: **Anatomical receptive fields.** Each colored hexagon is an input connection, with the connection strength characterized by the average number of synapses that we count from the EM reconstruction. Red indicates excitatory synapses, blue indicates inhibitory synapses from inferred signs. Filters in the order of their total number of synapses.

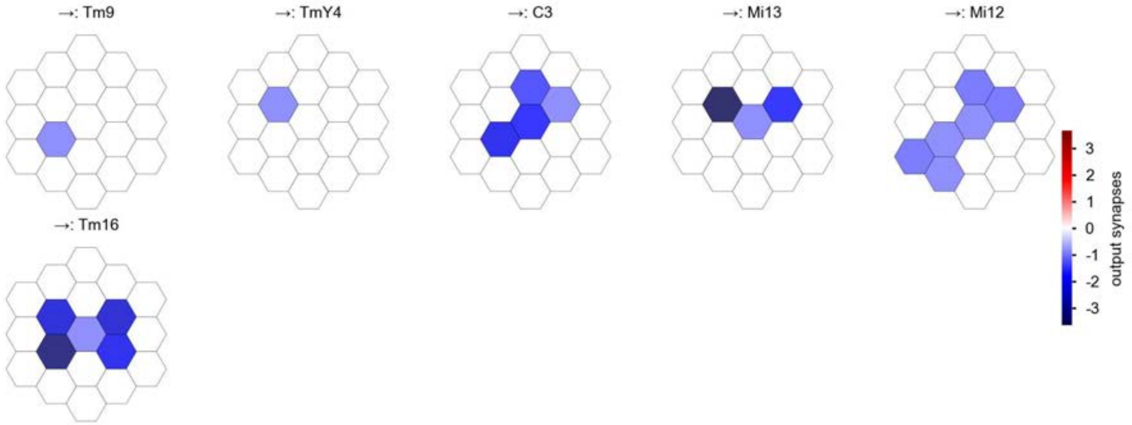

TmY14 - Figure 2: **Anatomical projective fields.** Each colored hexagon is an output connection, with the connection strength characterized by the average number of synapses that we count from the EM reconstruction. Red indicates excitatory synapses, blue indicates inhibitory synapses from inferred signs. Filters in the order of their total number of synapses.

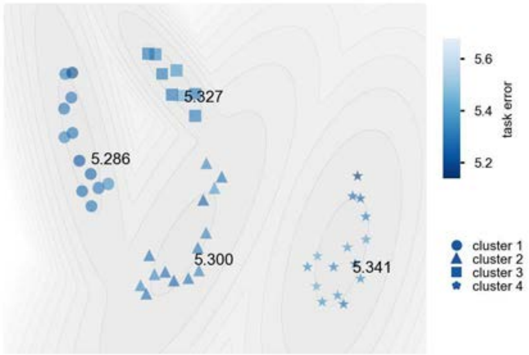

TmY14 - Figure 3: **Clustering of the responses to naturalistic stimuli.** Clustering of the 50 models based on the cell type responses to naturalistic scenes from the Sintel dataset. Scatterpoints represent individual models colored by their task error.

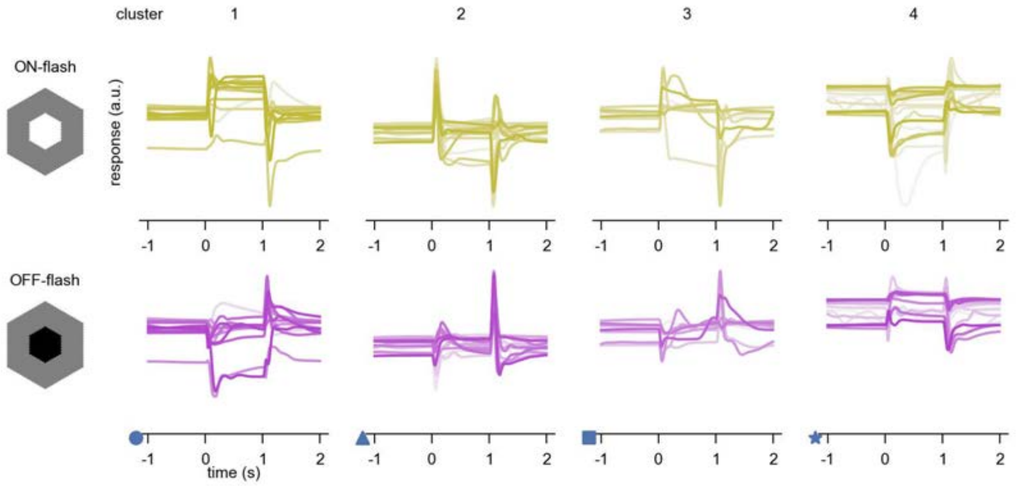

TmY14 - Figure 4: **Responses to flashes.** The top row shows responses to ON-flashes (yellow), the bottom row shows responses to OFF-flashes (magenta). The responses from the 50 different models that are separated into the different clusters (columns) overlay, with better task-performing models on top. Responses from better task-performing models are more saturated. The circular flashes (1s) cover 6 ommatidia in radius and are presented at time zero. Before and after, a grey-stimulus leads to a stationary state of the network.

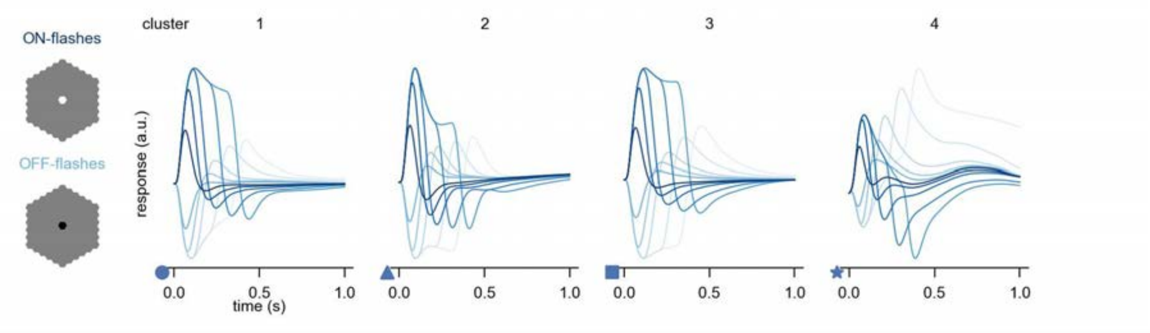

TmY14 - Figure 5: **Cluster-average responses to single-ommatidium flashes.** Responses to single-ommatidium ON-flashes (dark blue shades) and single-ommatidium OFF-flashes (light blue shades) of 20ms, 50ms, 100ms, 200ms, 300ms duration. The flashes occur at second zero.

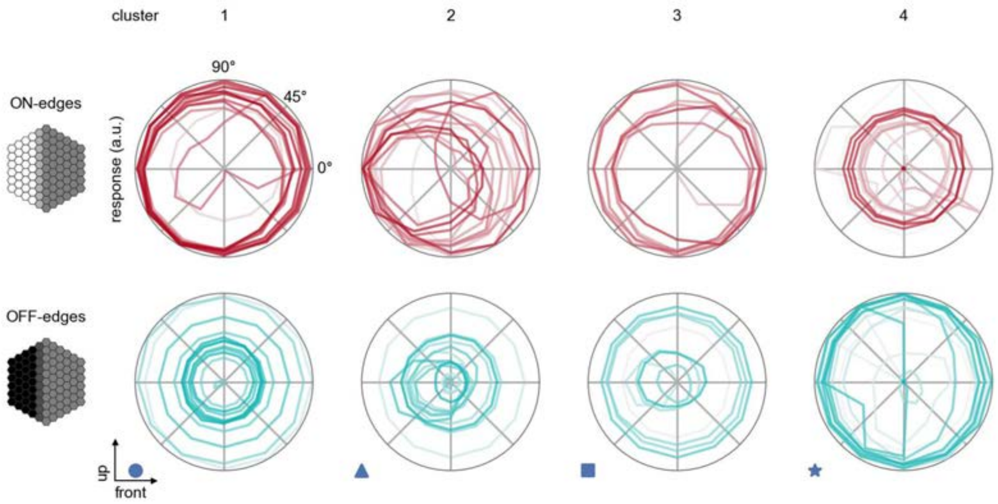

TmY14 - Figure 6: **Peak responses to moving edges.** The top row shows peak responses to moving ON-edges (red), the bottom row shows peak responses to moving OFF-edges (turqoise). The peak responses are averaged over edge-speeds. Edge-stimuli move in different directions from 0 to 360 degrees. The responses from the different models in the different clusters (columns) overlay. Responses from better task-performing models are more saturated.

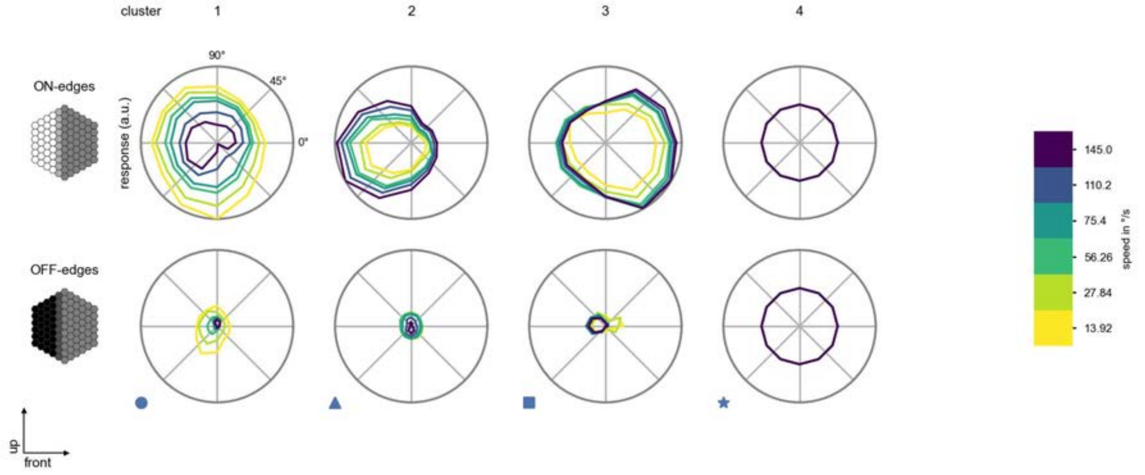

TmY14 - Figure 7: **Peak responses to moving edges from task-optimal models.** The top row shows peak responses to moving ON-edges, the bottom row shows peak responses to moving OFF-edges of varying speeds from 13.92°/s to 145°/s (yellow to dark blue). The edge-stimuli move in different directions from 0 to 360 degrees and at different speeds. Responses from the task-optimal model in the respective cluster.

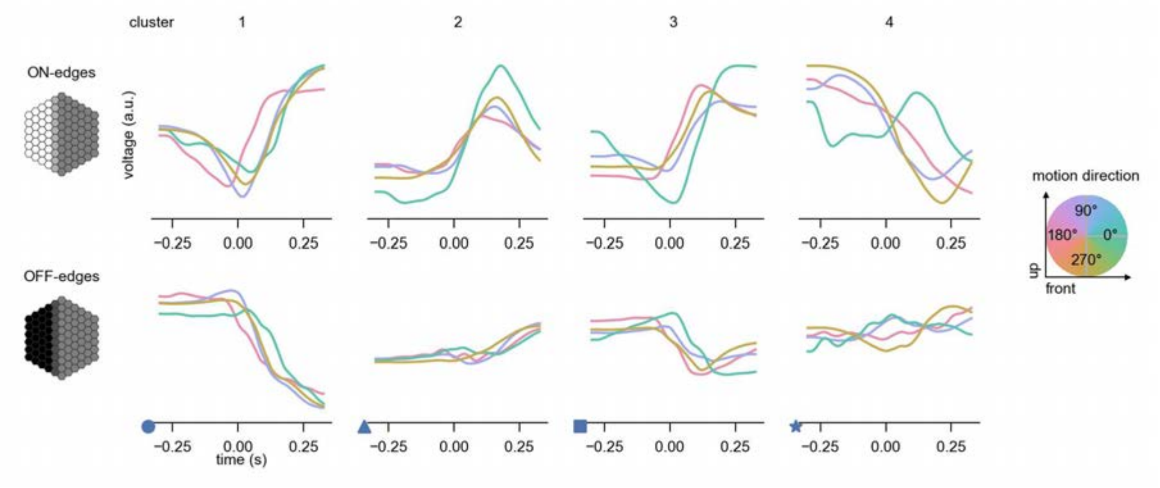

TmY14 - Figure 8: **Responses to moving edges from task-optimal models.** Responses to moving ON-edges (top row) and to moving OFF-edges (bottom row). Edges move in different directions from 0 to 360 degrees and at different speeds. Responses are from the task-optimal model in the respective cluster. Edges moving at 75.4°/s in all cardinal directions (green 0°, blue 90°, red 180°, yellow 270°) from -22.5 to 22.5° visual angle.

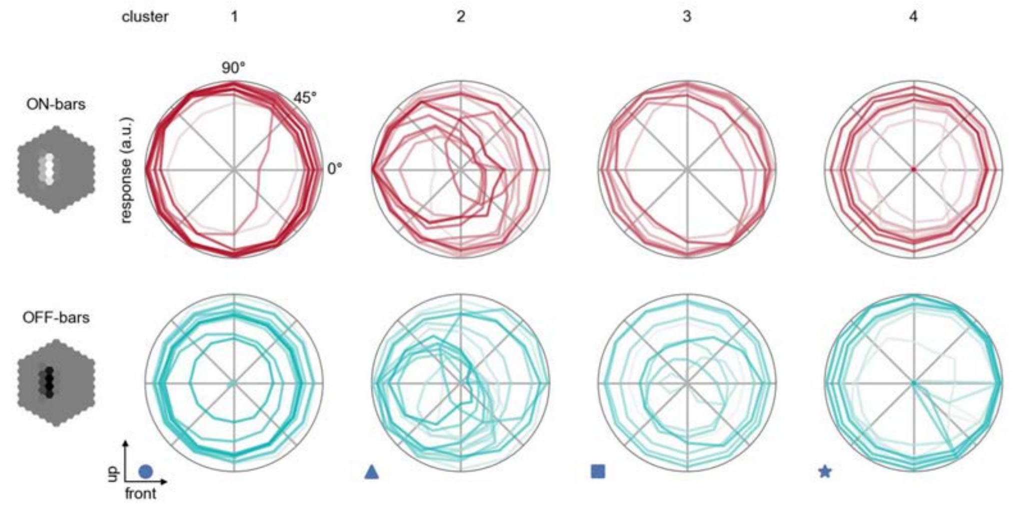

TmY14 - Figure 9: **Peak responses to moving bars.** The top row shows peak responses to moving ON-bars (red), the bottom row shows peak responses to moving OFF-bars (turqoise). The peak responses are averaged over bar-speeds. Bar-stimuli move in different directions from 0 to 360 degrees. The responses from the different models in the different clusters (columns) overlay. Responses from better task-performing models are more saturated.

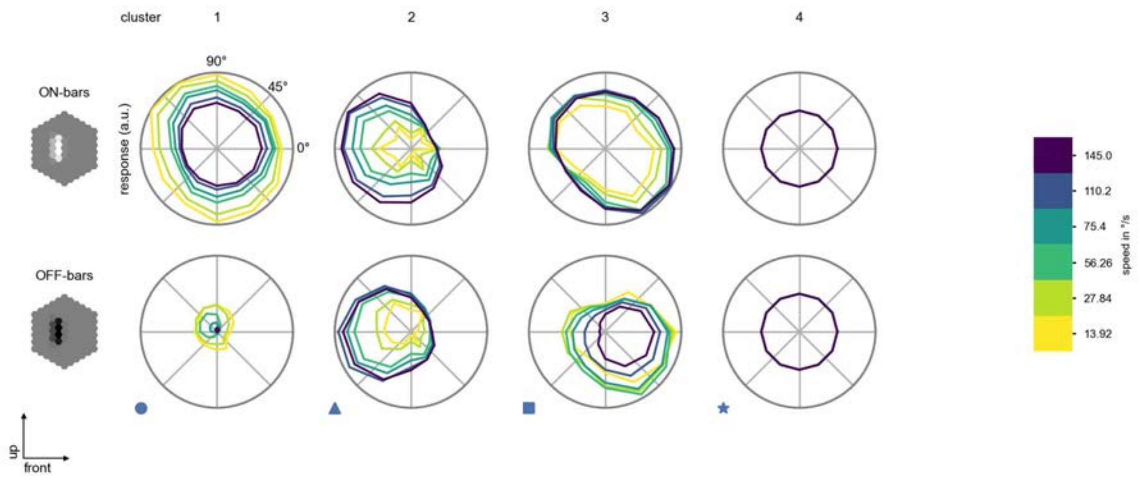

TmY14 - Figure 10: **Peak responses to moving bars from task-optimal models.** The top row shows peak responses to moving ON-bars, the bottom row shows peak responses to moving OFF-bars of varying speeds from 13.92°/s to 145°/s (yellow to dark blue). The bar-stimuli move in different directions from 0 to 360 degrees and at different speeds. Responses from the task-optimal model in the respective cluster.

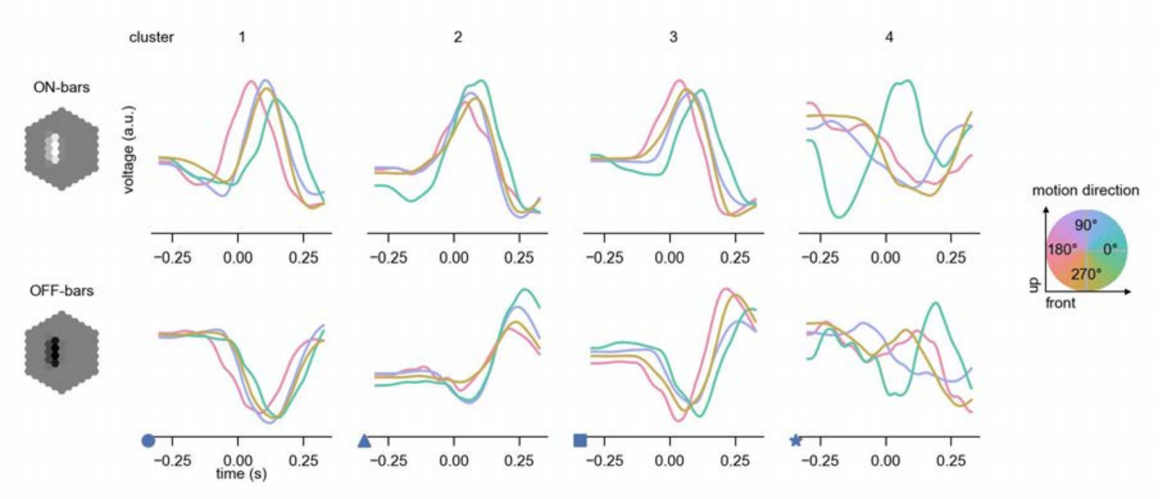

TmY14 - Figure 11: **Responses to moving bars from task-optimal models.** Responses to moving ON-bars (top row) and to moving OFF-bars (bottom row). Bars move in different directions from 0 to 360 degrees and at different speeds. Responses are from the task-optimal model in the respective cluster. Bars moving at 75.4°/s in all cardinal directions (green 0°, blue 90°, red 180°, yellow 270°) from -22.5 to 22.5° visual angle.

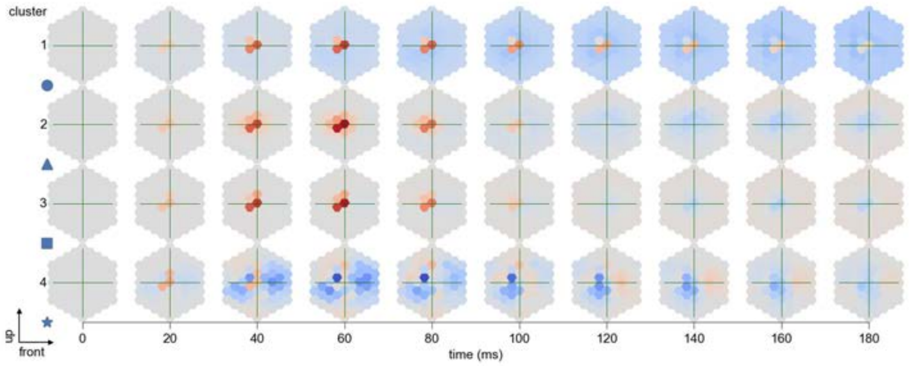

TmY14 - Figure 12: **Spatio-temporal receptive field.** Responses of the central cell to ON-impulses (5 ms) at single-ommatidium flash locations. The flash occurs at second zero. Responses from the task-optimal model of the respective cluster (rows). Red indicates depolarization, blue indicates hyperpolarization.

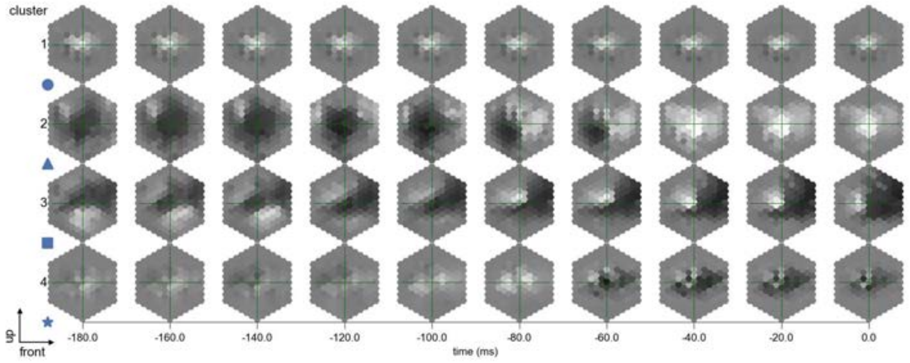

TmY14 - Figure 13: **Maximally excitatory stimuli.** Each row presents the regularized naturalistic-stimulus from the Sintel dataset that maximizes the cell type's central column response at second zero in the task-optimal model of the respective cluster (rows).

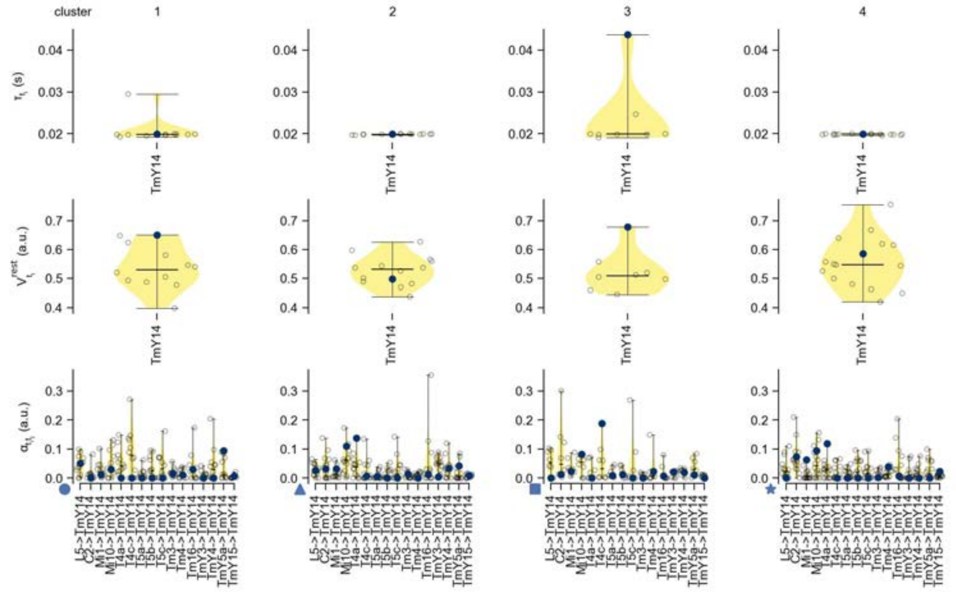

TmY14 - Figure 14: **Task-constrained parameters.** Each column shows the parameters inferred within the respective cluster. First row: learned time constants of the cell type. Second row: resting potentials of the cell type. Third row: scaling factors for the convolutional filters. The blue scatter represents the parameters from the task-optimal model within the cluster.

64 TmY15

← Cell types

Figures

1 Anatomical receptive fields. . . . 463
2 Anatomical projective fields. . . . 464
3 Clustering of the responses to naturalistic stimuli. . . . 464
4 Responses to flashes. . . . 465
5 Cluster-average responses to single-ommatidium flashes. . . . 465
6 Peak responses to moving edges. . . . 466
7 Peak responses to moving edges from task-optimal models. . . . 466
8 Responses to moving edges from task-optimal models. . . . 467
9 Peak responses to moving bars. . . . 467
10 Peak responses to moving bars from task-optimal models. . . . 468
11 Responses to moving bars from task-optimal models. . . . 468
12 Spatio-temporal receptive field. . . . 468
13 Maximally excitatory stimuli. . . . 469
14 Task-constrained parameters. . . . 469

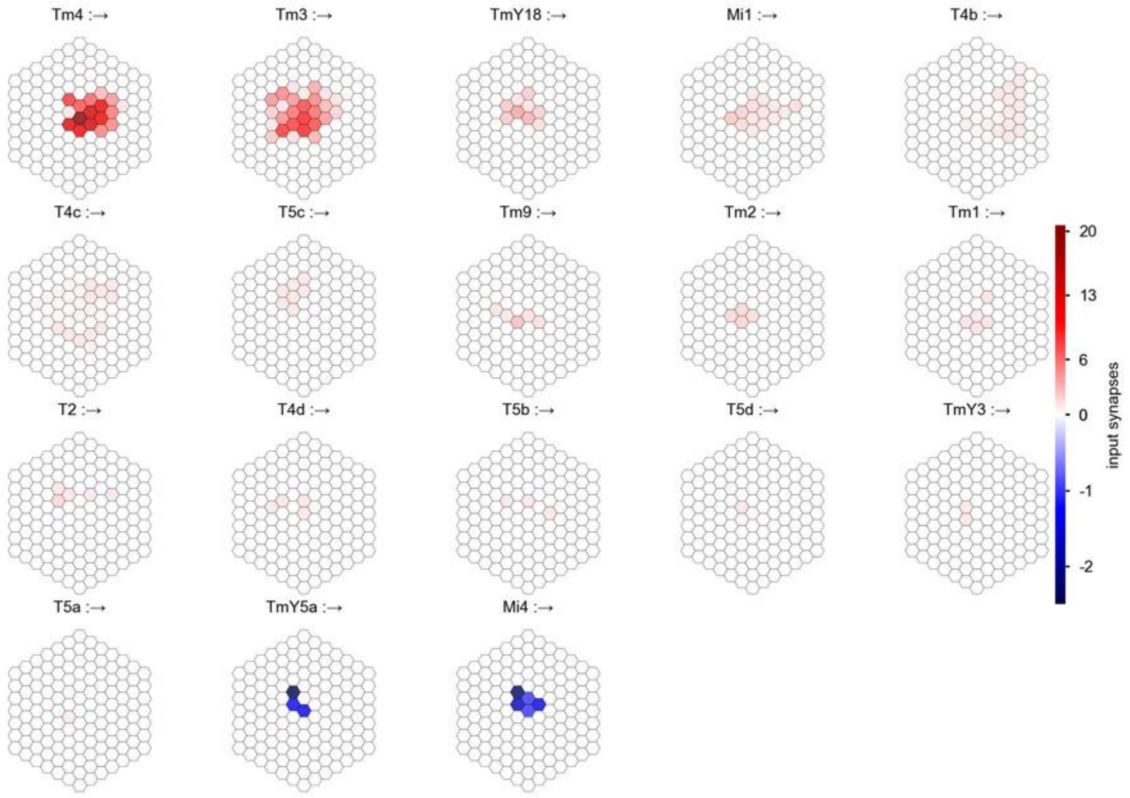

TmY15 - Figure 1: **Anatomical receptive fields.** Each colored hexagon is an input connection, with the connection strength characterized by the average number of synapses that we count from the EM reconstruction. Red indicates excitatory synapses, blue indicates inhibitory synapses from inferred signs. Filters in the order of their total number of synapses.

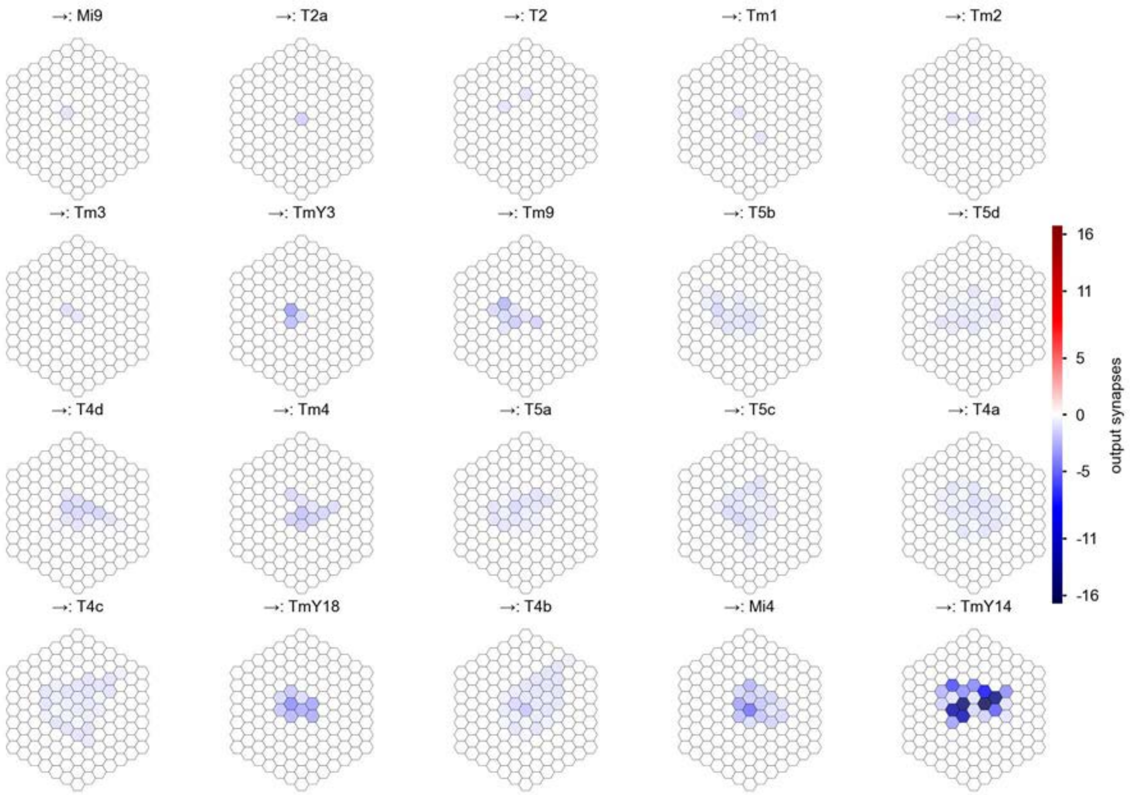

TmY15 - Figure 2: **Anatomical projective fields.** Each colored hexagon is an output connection, with the connection strength characterized by the average number of synapses that we count from the EM reconstruction. Red indicates excitatory synapses, blue indicates inhibitory synapses from inferred signs. Filters in the order of their total number of synapses.

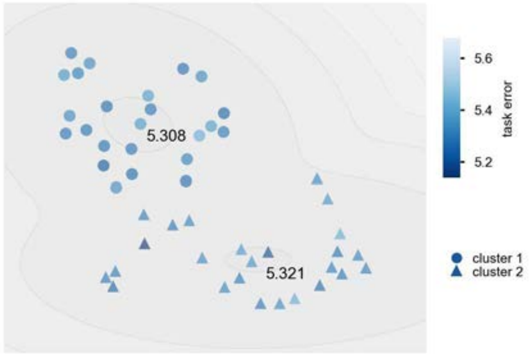

TmY15 - Figure 3: **Clustering of the responses to naturalistic stimuli.** Clustering of the 50 models based on the cell type responses to naturalistic scenes from the Sintel dataset. Scatterpoints represent individual models colored by their task error.

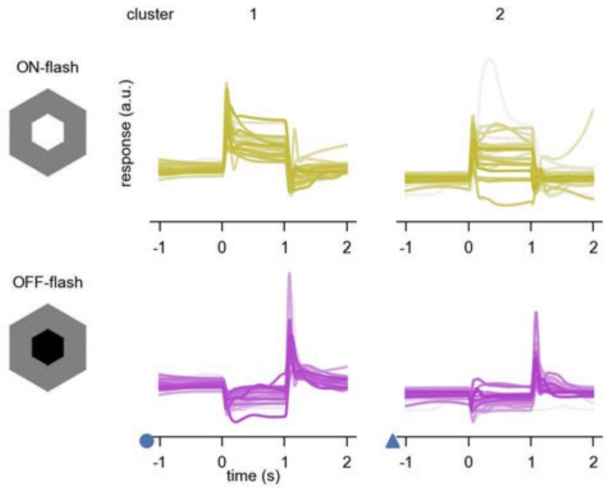

TmY15 - Figure 4: **Responses to flashes.** The top row shows responses to ON-flashes (yellow), the bottom row shows responses to OFF-flashes (magenta). The responses from the 50 different models that are separated into the different clusters (columns) overlay, with better task-performing models on top. Responses from better task-performing models are more saturated. The circular flashes (1s) cover 6 ommatidia in radius and are presented at time zero. Before and after, a grey-stimulus leads to a stationary state of the network.

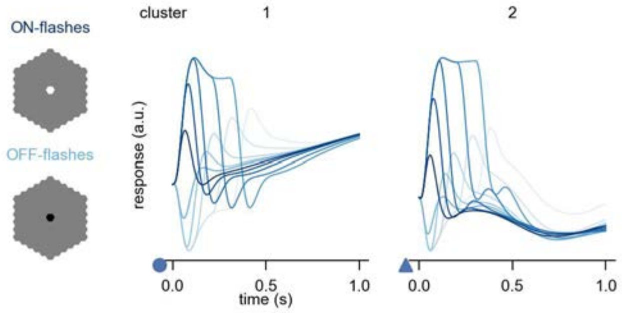

TmY15 - Figure 5: **Cluster-average responses to single-ommatidium flashes.** Responses to single-ommatidium ON-flashes (dark blue shades) and single-ommatidium OFF-flashes (light blue shades) of 20ms, 50ms, 100ms, 200ms, 300ms duration. The flashes occur at second zero.

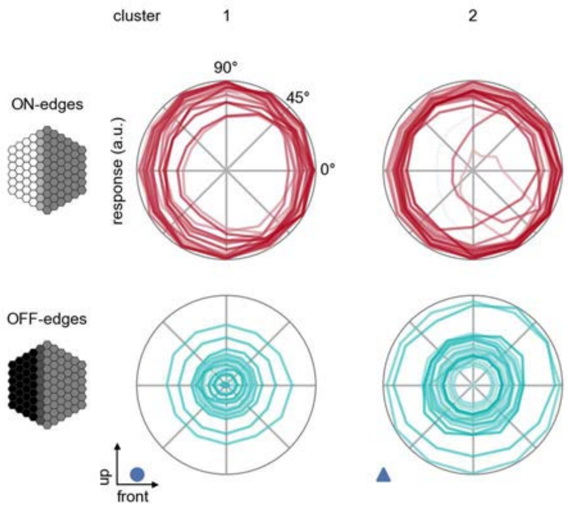

TmY15 - Figure 6: **Peak responses to moving edges.** The top row shows peak responses to moving ON-edges (red), the bottom row shows peak responses to moving OFF-edges (turqoise). The peak responses are averaged over edge-speeds. Edge-stimuli move in different directions from 0 to 360 degrees. The responses from the different models in the different clusters (columns) overlay. Responses from better task-performing models are more saturated.

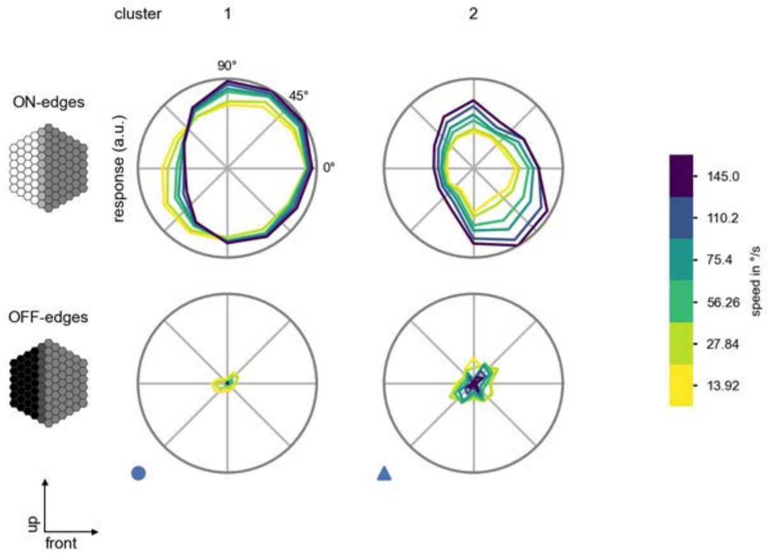

TmY15 - Figure 7: **Peak responses to moving edges from task-optimal models.** The top row shows peak responses to moving ON-edges, the bottom row shows peak responses to moving OFF-edges of varying speeds from 13.92°/s to 145°/s (yellow to dark blue). The edge-stimuli move in different directions from 0 to 360 degrees and at different speeds. Responses from the task-optimal model in the respective cluster.

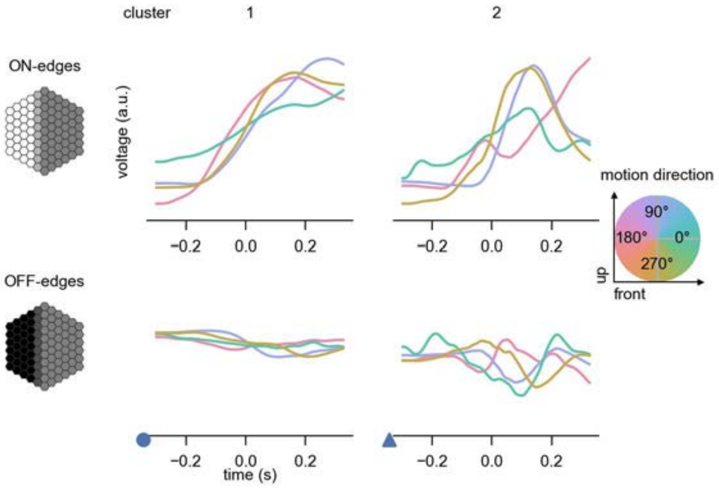

TmY15 - Figure 8: **Responses to moving edges from task-optimal models.** Responses to moving ON-edges (top row) and to moving OFF-edges (bottom row). Edges move in different directions from 0 to 360 degrees and at different speeds. Responses are from the task-optimal model in the respective cluster. Edges moving at 75.4°/s in all cardinal directions (green 0°, blue 90°, red 180°, yellow 270°) from -22.5 to 22.5° visual angle.

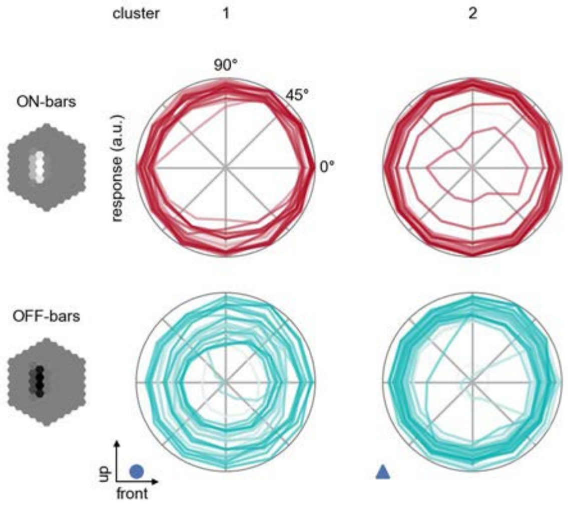

TmY15 - Figure 9: **Peak responses to moving bars.** The top row shows peak responses to moving ON-bars (red), the bottom row shows peak responses to moving OFF-bars (turqoise). The peak responses are averaged over bar-speeds. Bar-stimuli move in different directions from 0 to 360 degrees. The responses from the different models in the different clusters (columns) overlay. Responses from better task-performing models are more saturated.

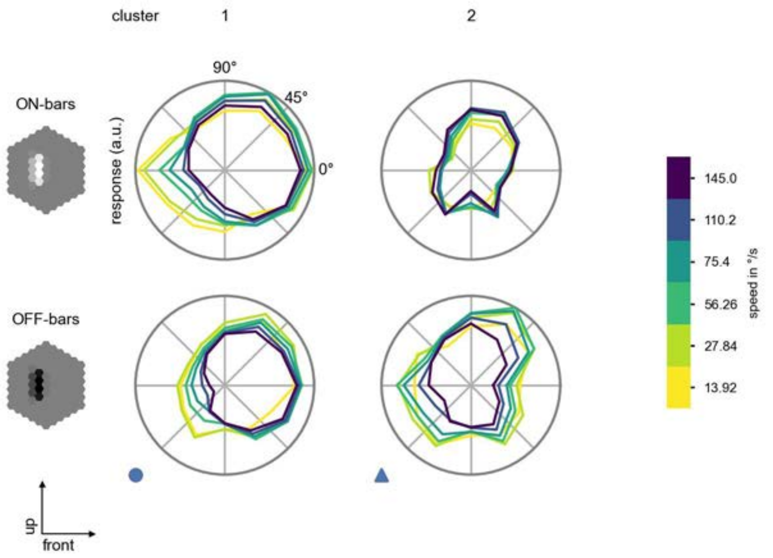

TmY15 - Figure 10: **Peak responses to moving bars from task-optimal models.** The top row shows peak responses to moving ON-bars, the bottom row shows peak responses to moving OFF-bars of varying speeds from 13.92°/s to 145°/s (yellow to dark blue). The bar-stimuli move in different directions from 0 to 360 degrees and at different speeds. Responses from the task-optimal model in the respective cluster.

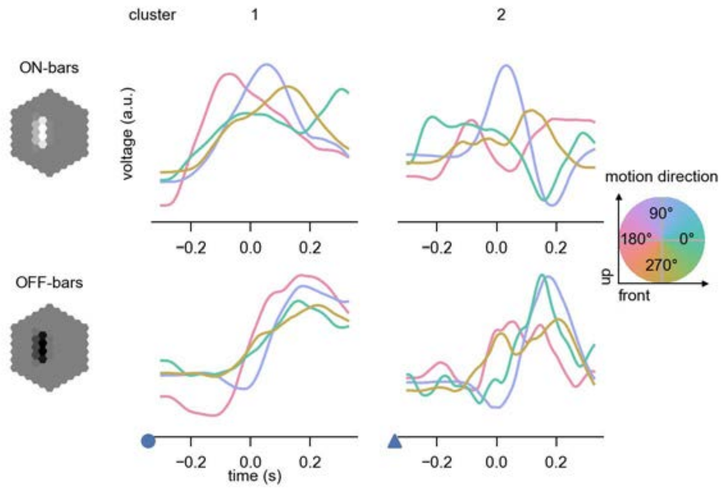

TmY15 - Figure 11: **Responses to moving bars from task-optimal models.** Responses to moving ON-bars (top row) and to moving OFF-bars (bottom row). Bars move in different directions from 0 to 360 degrees and at different speeds. Responses are from the task-optimal model in the respective cluster. Bars moving at 75.4°/s in all cardinal directions (green 0°, blue 90°, red 180°, yellow 270°) from -22.5 to 22.5° visual angle.

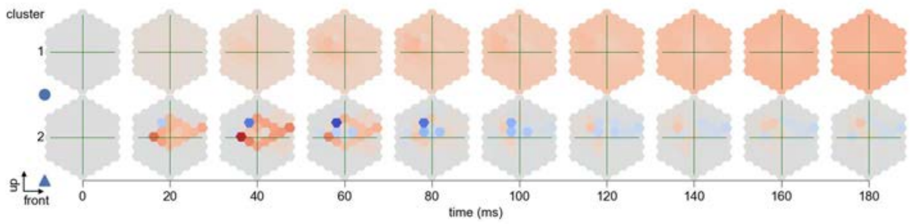

TmY15 - Figure 12: **Spatio-temporal receptive field.** Responses of the central cell to ON-impulses (5 ms) at single-ommatidium flash locations. The flash occurs at second zero. Responses from the task-optimal model of the respective cluster (rows). Red indicates depolarization, blue indicates hyperpolarization.

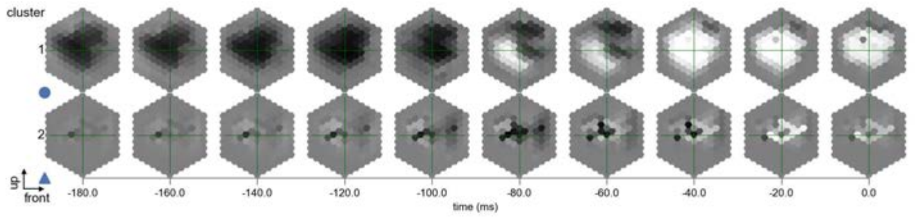

TmY15 - Figure 13: **Maximally excitatory stimuli.** Each row presents the regularized naturalistic-stimulus from the Sintel dataset that maximizes the cell type's central column response at second zero in the task-optimal model of the respective cluster (rows).

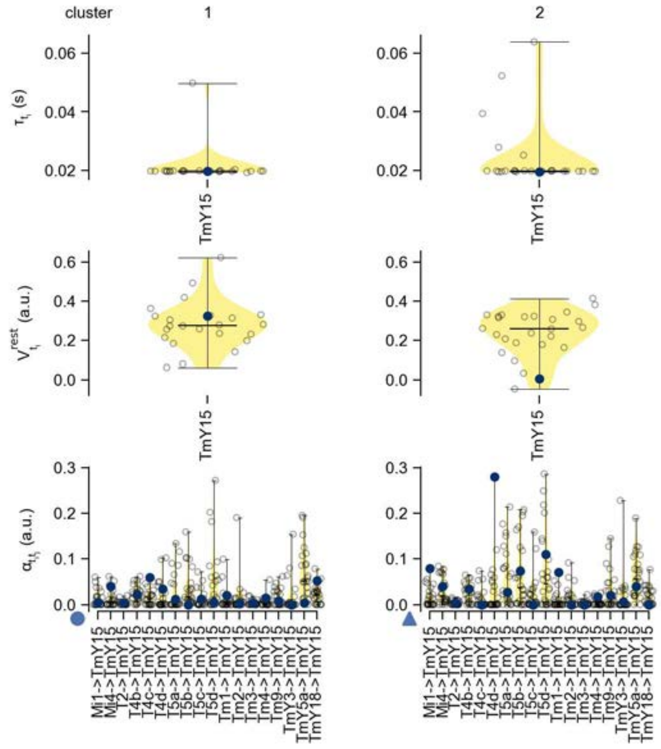

TmY15 - Figure 14: **Task-constrained parameters.** Each column shows the parameters inferred within the respective cluster. First row: learned time constants of the cell type. Second row: resting potentials of the cell type. Third row: scaling factors for the convolutional filters. The blue scatter represents the parameters from the task-optimal model within the cluster.

65 TmY18

← Cell types

Figures

1 Anatomical receptive fields. . . . 470
2 Anatomical projective fields. . . . 471
3 Clustering of the responses to naturalistic stimuli. . . . 471
4 Responses to flashes. . . . 472
5 Cluster-average responses to single-ommatidium flashes. . . . 472
6 Peak responses to moving edges. . . . 473
7 Peak responses to moving edges from task-optimal models. . . . 473
8 Responses to moving edges from task-optimal models. . . . 474
9 Peak responses to moving bars. . . . 474
10 Peak responses to moving bars from task-optimal models. . . . 475
11 Responses to moving bars from task-optimal models. . . . 475
12 Spatio-temporal receptive field. . . . 476
13 Maximally excitatory stimuli. . . . 476
14 Task-constrained parameters. . . . 477

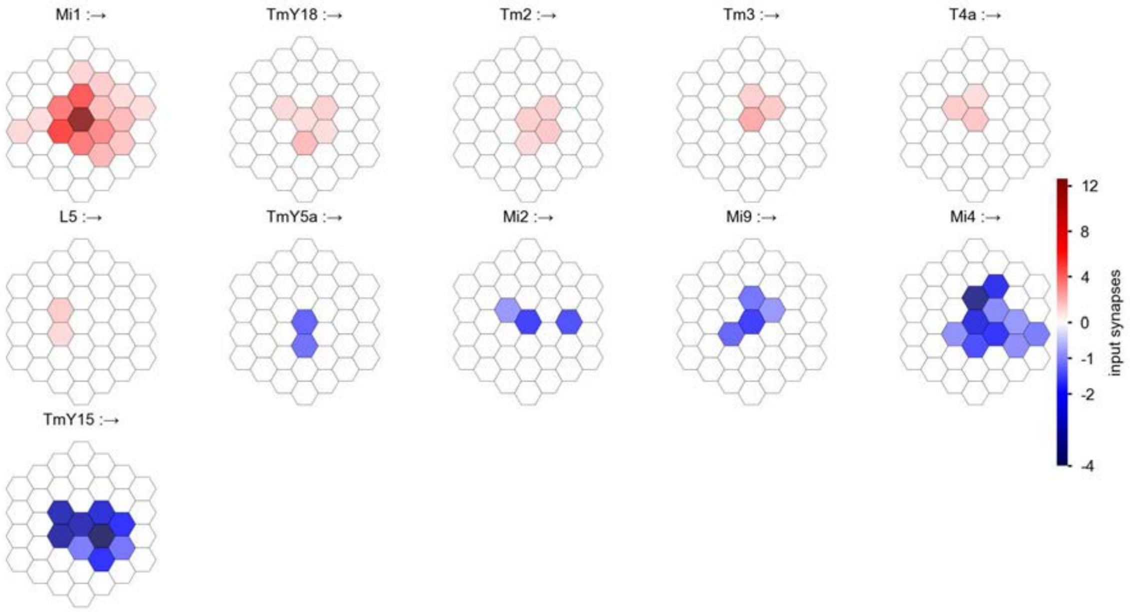

TmY18 - Figure 1: **Anatomical receptive fields.** Each colored hexagon is an input connection, with the connection strength characterized by the average number of synapses that we count from the EM reconstruction. Red indicates excitatory synapses, blue indicates inhibitory synapses from inferred signs. Filters in the order of their total number of synapses.

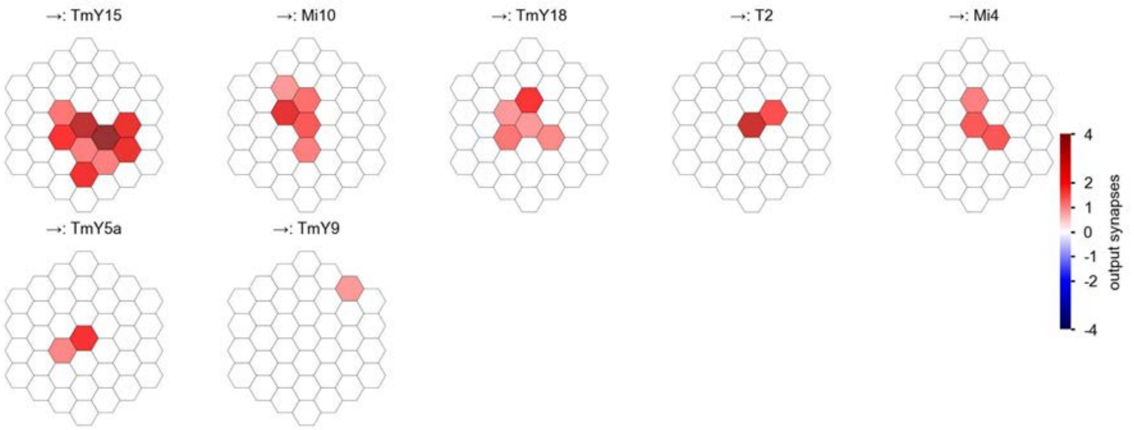

TmY18 - Figure 2: **Anatomical projective fields.** Each colored hexagon is an output connection, with the connection strength characterized by the average number of synapses that we count from the EM reconstruction. Red indicates excitatory synapses, blue indicates inhibitory synapses from inferred signs. Filters in the order of their total number of synapses.

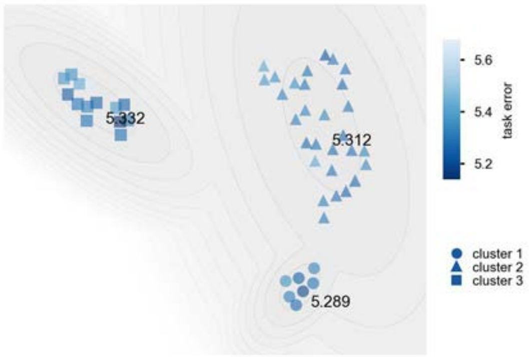

TmY18 - Figure 3: **Clustering of the responses to naturalistic stimuli.** Clustering of the 50 models based on the cell type responses to naturalistic scenes from the Sintel dataset. Scatterpoints represent individual models colored by their task error.

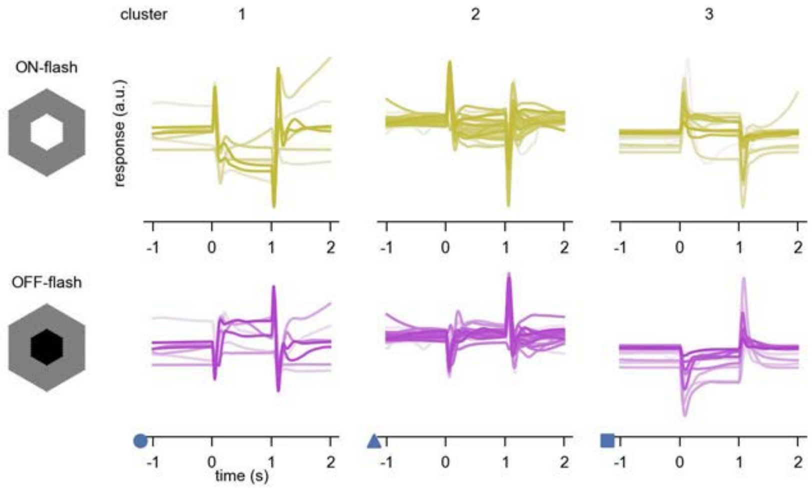

TmY18 - Figure 4: **Responses to flashes.** The top row shows responses to ON-flashes (yellow), the bottom row shows responses to OFF-flashes (magenta). The responses from the 50 different models that are separated into the different clusters (columns) overlay, with better task-performing models on top. Responses from better task-performing models are more saturated. The circular flashes (1s) cover 6 ommatidia in radius and are presented at time zero. Before and after, a grey-stimulus leads to a stationary state of the network.

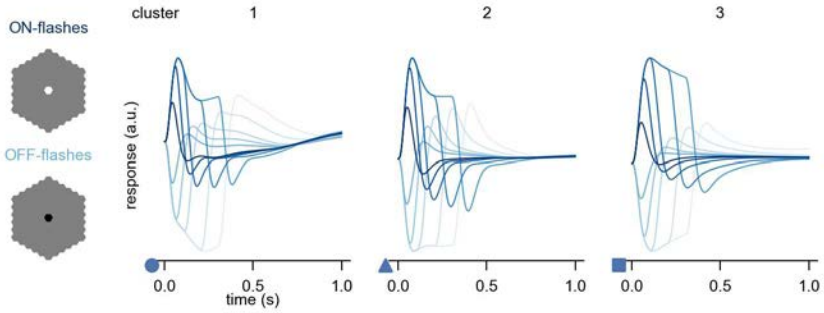

TmY18 - Figure 5: **Cluster-average responses to single-ommatidium flashes.** Responses to single-ommatidium ON-flashes (dark blue shades) and single-ommatidium OFF-flashes (light blue shades) of 20ms, 50ms, 100ms, 200ms, 300ms duration. The flashes occur at second zero.

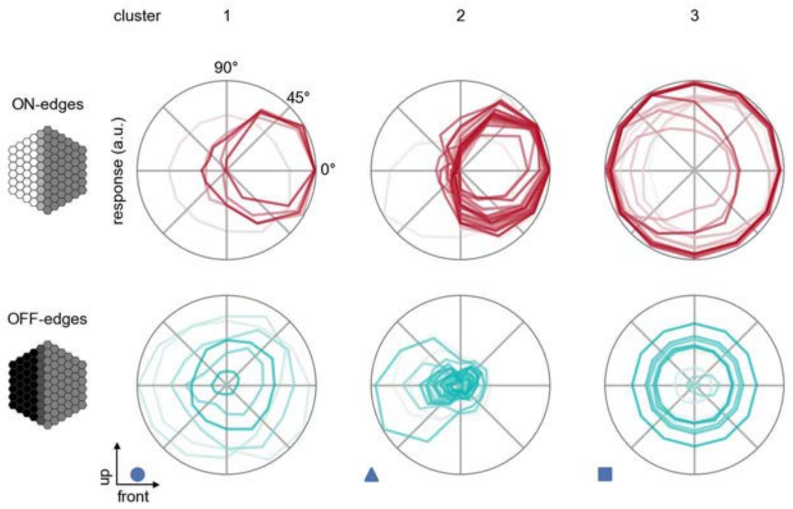

TmY18 - Figure 6: **Peak responses to moving edges.** The top row shows peak responses to moving ON-edges (red), the bottom row shows peak responses to moving OFF-edges (turqoise). The peak responses are averaged over edge-speeds. Edge-stimuli move in different directions from 0 to 360 degrees. The responses from the different models in the different clusters (columns) overlay. Responses from better task-performing models are more saturated.

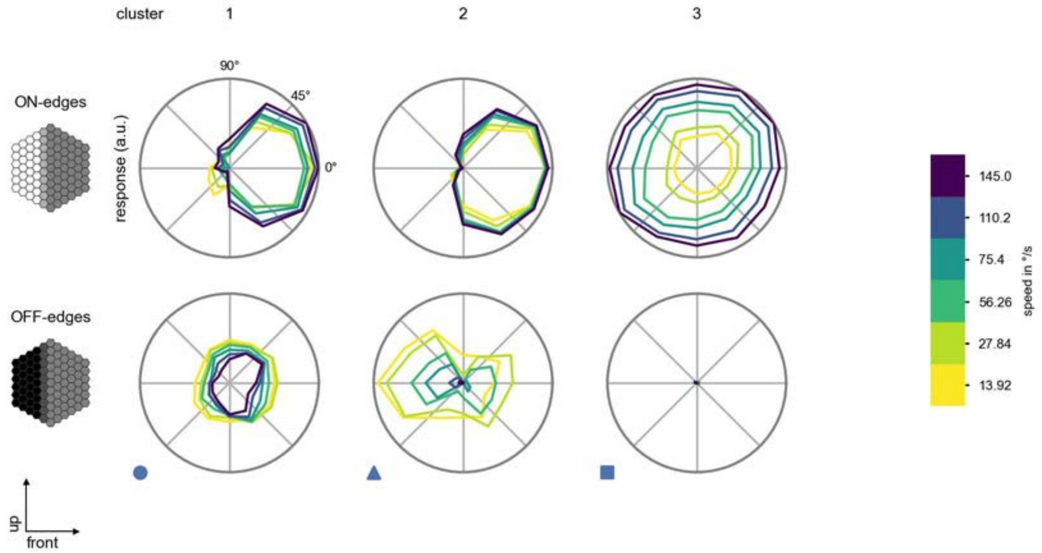

TmY18 - Figure 7: **Peak responses to moving edges from task-optimal models.** The top row shows peak responses to moving ON-edges, the bottom row shows peak responses to moving OFF-edges of varying speeds from 13.92°/s to 145°/s (yellow to dark blue). The edge-stimuli move in different directions from 0 to 360 degrees and at different speeds. Responses from the task-optimal model in the respective cluster.

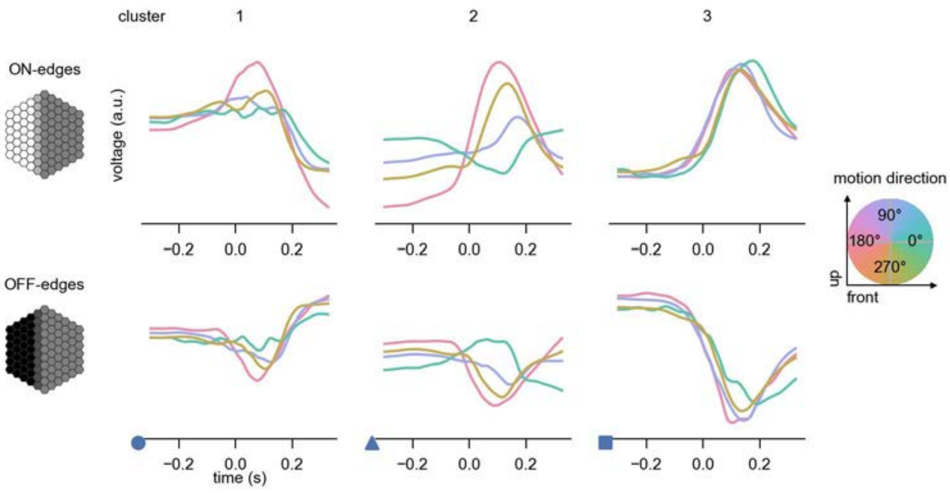

TmY18 - Figure 8: **Responses to moving edges from task-optimal models.** Responses to moving ON-edges (top row) and to moving OFF-edges (bottom row). Edges move in different directions from 0 to 360 degrees and at different speeds. Responses are from the task-optimal model in the respective cluster. Edges moving at 75.4°/s in all cardinal directions (green 0°, blue 90°, red 180°, yellow 270°) from -22.5 to 22.5° visual angle.

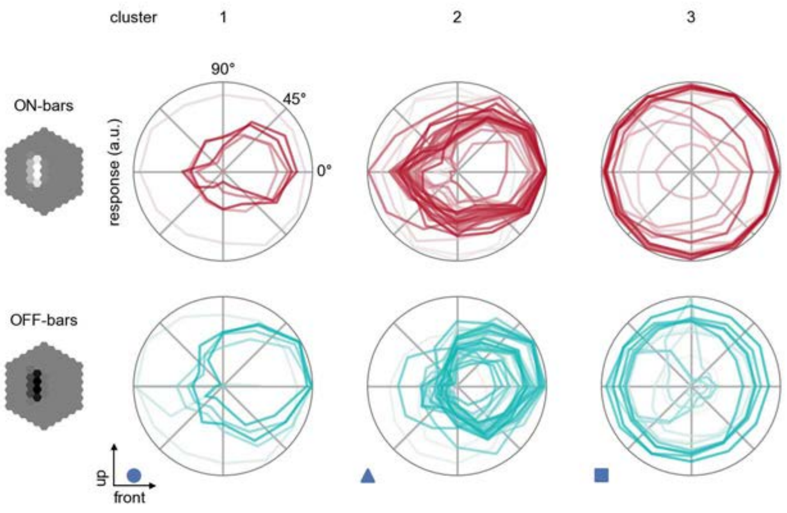

TmY18 - Figure 9: **Peak responses to moving bars.** The top row shows peak responses to moving ON-bars (red), the bottom row shows peak responses to moving OFF-bars (turqoise). The peak responses are averaged over bar-speeds. Bar-stimuli move in different directions from 0 to 360 degrees. The responses from the different models in the different clusters (columns) overlay. Responses from better task-performing models are more saturated.

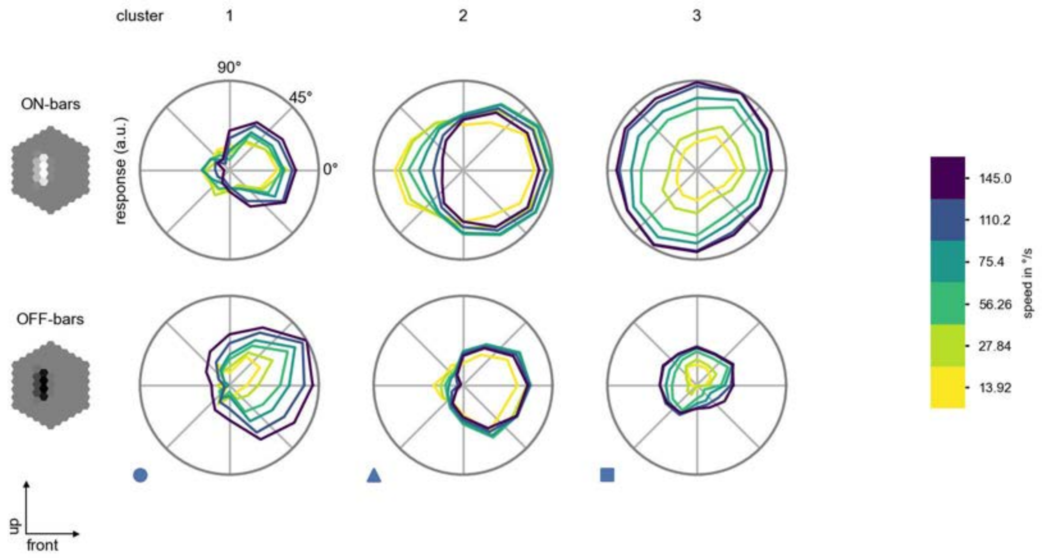

TmY18 - Figure 10: **Peak responses to moving bars from task-optimal models.** The top row shows peak responses to moving ON-bars, the bottom row shows peak responses to moving OFF-bars of varying speeds from 13.92°/s to 145°/s (yellow to dark blue). The bar-stimuli move in different directions from 0 to 360 degrees and at different speeds. Responses from the task-optimal model in the respective cluster.

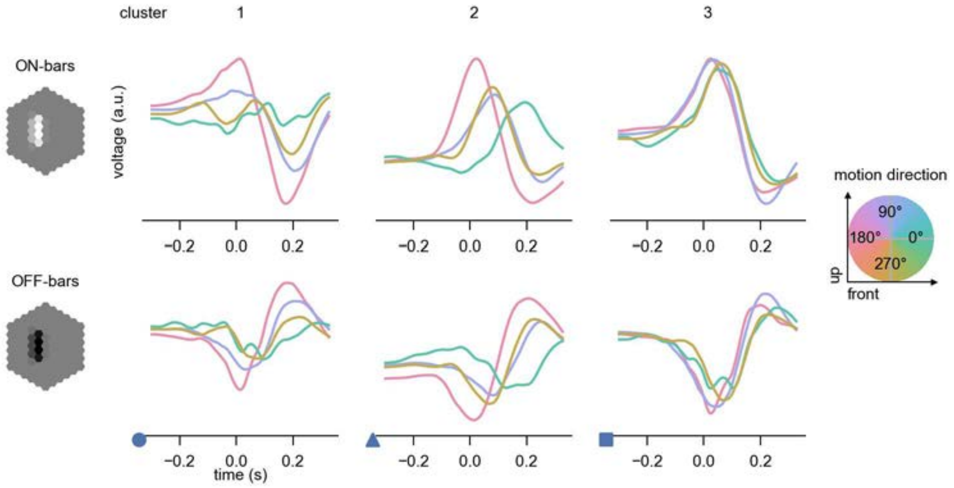

TmY18 - Figure 11: **Responses to moving bars from task-optimal models.** Responses to moving ON-bars (top row) and to moving OFF-bars (bottom row). Bars move in different directions from 0 to 360 degrees and at different speeds. Responses are from the task-optimal model in the respective cluster. Bars moving at 75.4°/s in all cardinal directions (green 0°, blue 90°, red 180°, yellow 270°) from -22.5 to 22.5° visual angle.

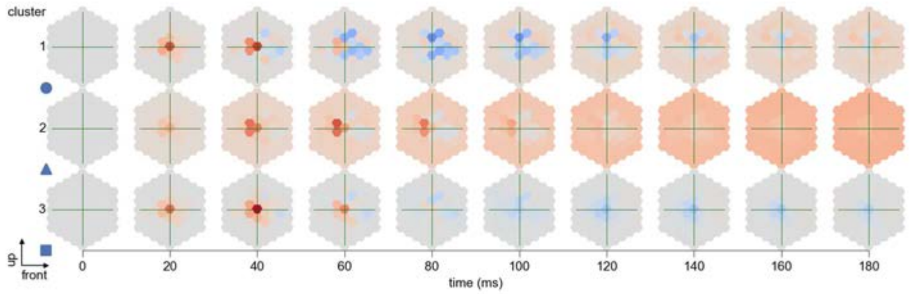

TmY18 - Figure 12: **Spatio-temporal receptive field.** Responses of the central cell to ON-impulses (5 ms) at single-ommatidium flash locations. The flash occurs at second zero. Responses from the task-optimal model of the respective cluster (rows). Red indicates depolarization, blue indicates hyperpolarization.

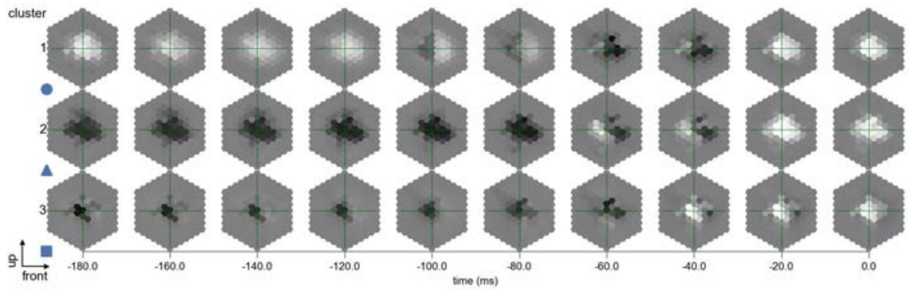

TmY18 - Figure 13: **Maximally excitatory stimuli.** Each row presents the regularized naturalistic-stimulus from the Sintel dataset that maximizes the cell type's central column response at second zero in the task-optimal model of the respective cluster (rows).

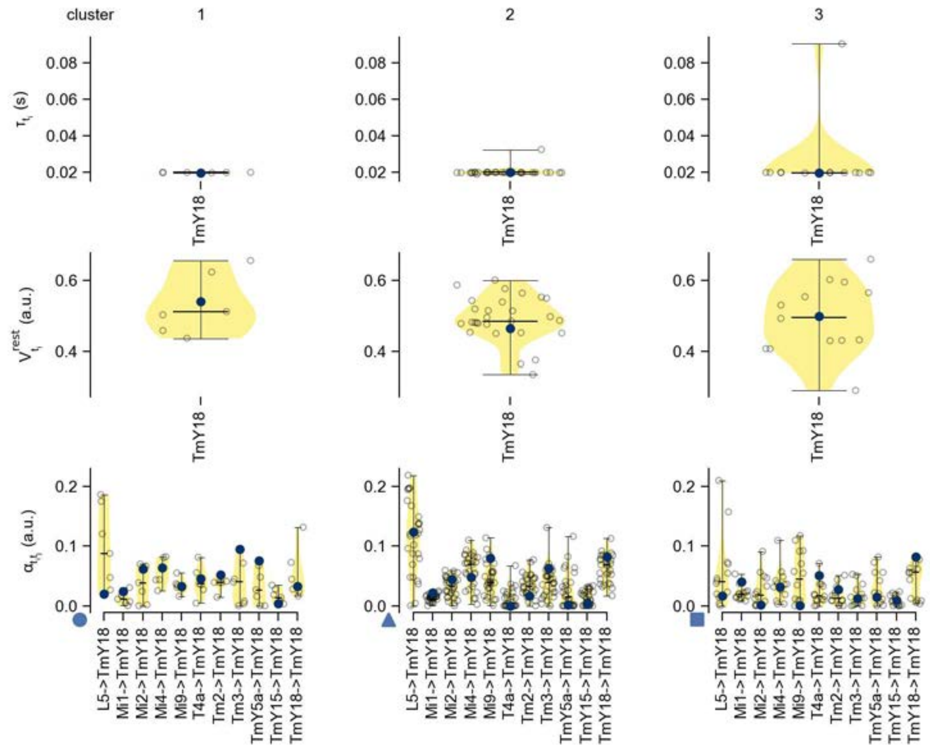

TmY18 - Figure 14: **Task-constrained parameters.** Each column shows the parameters inferred within the respective cluster. First row: learned time constants of the cell type. Second row: resting potentials of the cell type. Third row: scaling factors for the convolutional filters. The blue scatter represents the parameters from the task-optimal model within the cluster.
