# Supplementary material for: Connectome-constrained networks predict neural activity across the fly visual system
Source: Nature. 2024 Sep 11;634(8036):1132–40. doi: 10.1038/s41586-024-07939-3 (PMC11525180; doi:10.1038/s41586-024-07939-3)
Supplement: Supplementary file 3 — This zipped data folder contains seven files: (1) connectome type-to-type filters, average synapse count; (2) source references for the compiled connectome; (3) cell type-to-type connection matrix; (4) single-neuron-resolution connectome for 91 columns; (5) source references to 26 studies reporting neural activity measurements; (6) BibTeX file of 26 neural activity measurement studies; (7) catalogue of neural response predictions for all 64 cell types. [file 41586_2024_7939_MOESM3_ESM.zip › 2023-03-04080C-Supplementary Data/Lappalainen_et_al_SI_Data guide.docx]

# Supplementary information zip contents

1. Lappalainen_et_al_connectome_final_avg_filters.json

Title: Connectome type-to-type filters, average synapse count

Summary: Connectome after aggregating and preprocessing all data sources. This file contains the average filter per connected pair of cell types. From this file, we compile the full cell-to-cell connectivity graph on the hexagonal grid.

1. Lappalainen_et_al_connectome_filter_and_sign_references.xlsx

Title: Source references for the compiled connectome

Summary: Table reporting sources used to compile the connectivity and sign for each cell-type pair in the composite connectome. The table includes columns: presynaptic (cell type), postsynaptic (cell type), filter reference, dataset reference, sign reference, number of cells of presynaptic type, number of cells of postsynaptic type.

1. Lappalainen_et_al_cell_type_connectivity.xlsx
   Title: Cell type-to-type connection matrix

Summary: Cell type connectivity in total synapse count between each pair of cell types, aggregated per filter over all columns as in Fig. 1c.

1. Lappalainen_et_al_single_neuron_connectivity_91_columns.parquet
   Title: Single-neuron resolution connectome for 91 columns

Summary: Compiled single neuron-to-neuron connectivity across 91 columns of the eye model, accounting for 5,759 cells. Data corresponding to Extended Data Fig. 1.

1. Lappalainen_et_al_neural_activity_references.xlsx
   Title: Source references to 26 studies reporting neural activity measurements

Summary: References to 26 studies reporting neural activity measurements of cell

types contained in our model. The table includes columns: Title, Author, Year, Journal, Measured neuron type, Depolarizes to light increment (ON) or light decrement (OFF), Figure, Putative motion detection pathway, Description of putative function, Measurement type, Stimulus, Neuropil.

1. Lappalainen_et_al_neural_activity_references.bib

Title: BibTeX file of 26 neural activity measurement studies

Summary: BibTeX-entries for each of the 26 neural activity measurement studies used to validate our model predictions.

1. Lappalainen_et_al_all_cell_type_neural_activity_predictions.pdf

Title: Catalog of neural response predictions for all 64 cell types

Summary: For each cell type: anatomical receptive fields, anatomical projective fields, clustering of the responses to naturalistic stimuli, cluster responses to full-field flashes, single-ommatidium flashes, moving edges and bars, spatio-temporal receptive fields, maximally excitatory stimuli, task-constrained parameters.
